# Dynamic Catalytic Highly Enantioselective 1,3‐Dipolar Cycloadditions

**DOI:** 10.1002/anie.202108072

**Published:** 2021-08-03

**Authors:** Okan Yildirim, Michael Grigalunas, Lukas Brieger, Carsten Strohmann, Andrey P. Antonchick, Herbert Waldmann

**Affiliations:** ^1^ Max Planck Institute of Molecular Physiology Department of Chemical Biology Otto-Hahn-Strasse 11 44227 Dortmund Germany; ^2^ Technichal University Dortmund Faculty of Chemistry Chemical Biology Otto-Hahn-Strasse 6 44221 Dortmund Germany; ^3^ Technichal University Dortmund Faculty of Chemistry Inorganic Chemistry Otto-Hahn-Strasse 6 44221 Dortmund Germany; ^4^ Nottingham Trent University Department of Chemistry and Forensics Cifton Lane NG11 8NS Nottingham UK

**Keywords:** asymmetric synthesis, diastereodivergent synthesis, dynamic covalent chemistry, enantiodivergent synthesis, pseudo-natural products, reversible cycloaddition

## Abstract

In dynamic covalent chemistry, reactions follow a thermodynamically controlled pathway through equilibria. Reversible covalent‐bond formation and breaking in a dynamic process enables the interconversion of products formed under kinetic control to thermodynamically more stable isomers. Notably, enantioselective catalysis of dynamic transformations has not been reported and applied in complex molecule synthesis. We describe the discovery of dynamic covalent enantioselective metal‐complex‐catalyzed 1,3‐dipolar cycloaddition reactions. We have developed a stereodivergent tandem synthesis of structurally and stereochemically complex molecules that generates eight stereocenters with high diastereo‐ and enantioselectivity through asymmetric reversible bond formation in a dynamic process in two consecutive Ag‐catalyzed 1,3‐dipolar cycloadditions of azomethine ylides with electron‐poor olefins. Time‐dependent reversible dynamic covalent‐bond formation gives enantiodivergent and diastereodivergent access to structurally complex double cycloadducts with high selectivity from a common set of reagents.

## Graphical abstract

A highly enantioselectively catalyzed dynamic covalent one‐pot‐tandem 1,3‐dipolar cycloaddition of azomethine ylides to α′‐alkylidene‐2‐cyclopentenones is described. Time‐dependend enantio‐ and diastereodivergent synthesis was achieved by using the same set of reagents. A reversible cycloaddition of the kinetically favored product enables the interconversion to its more stable thermodynamically favored product through a chiral complex‐mediated process.

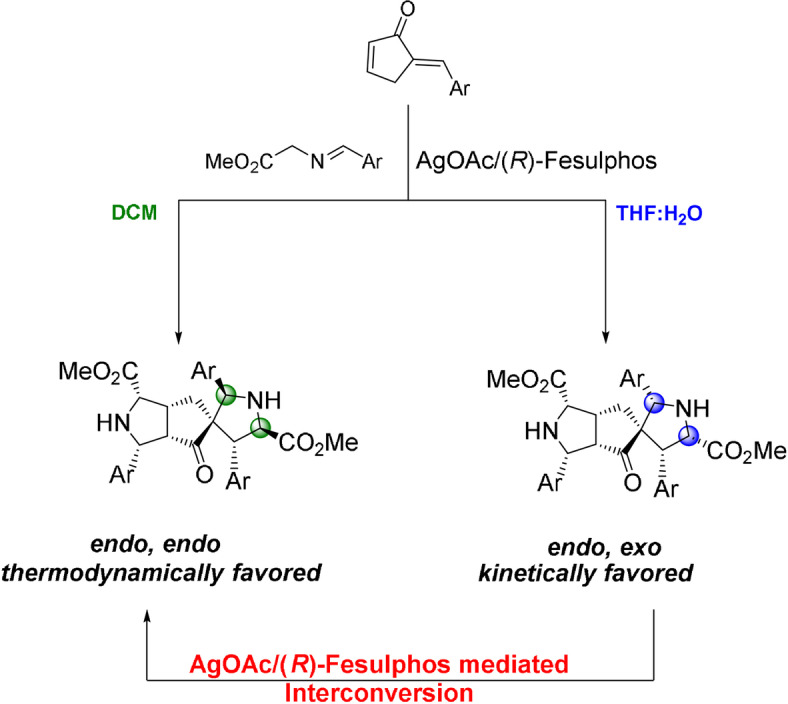

## Introduction

The synthesis of organic compounds is dominated by kinetically controlled reactions, which enables irreversible formation of strong covalent bonds.[^1^, ^2^] The irreversible nature of the reaction guarantees that, once the particular product is formed, it is stable and will not be reformed or converted into another product.^[1]^

**Scheme 1:**
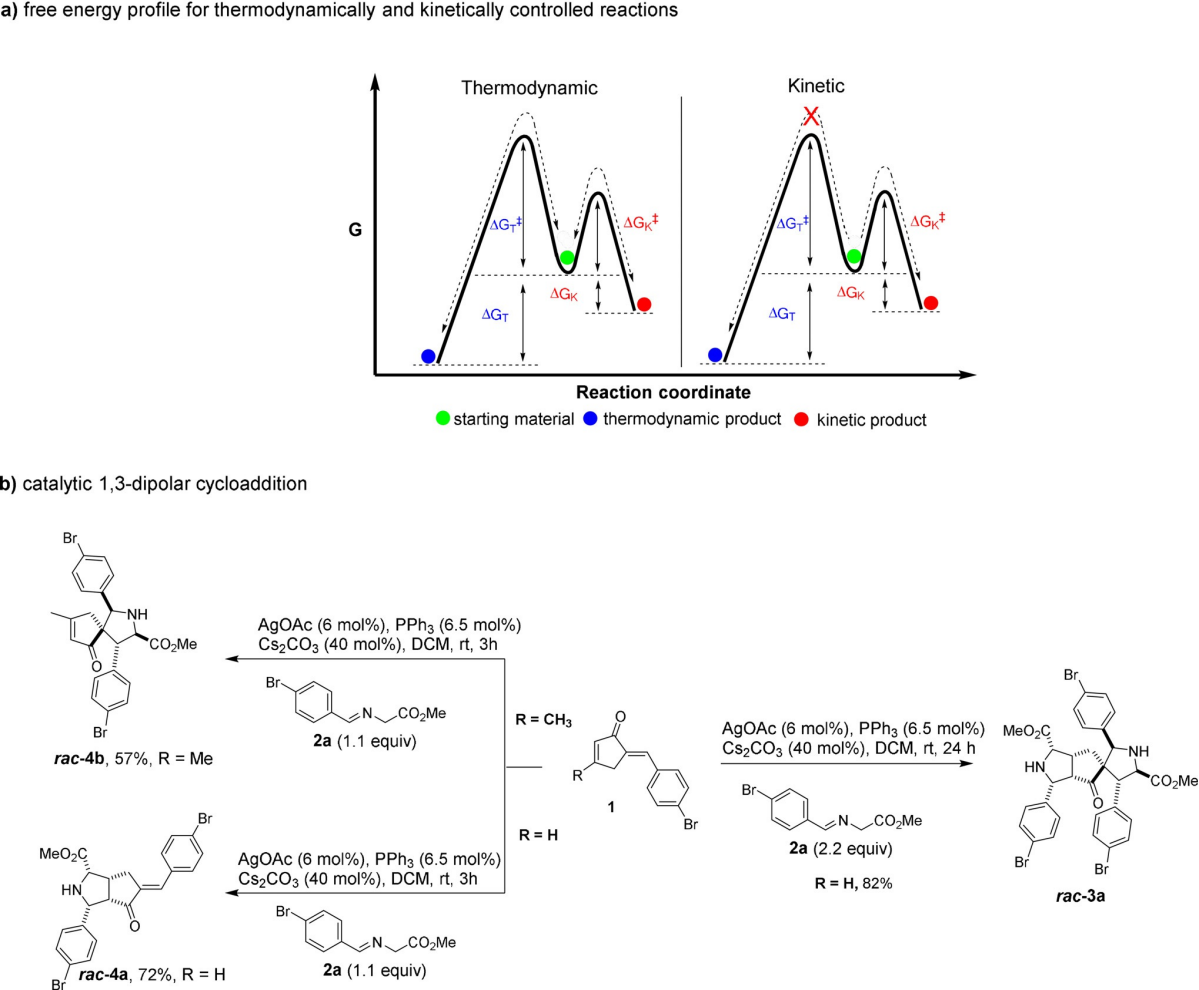
a) Free energy profile illustrating thermodynamically and kinetically controlled reactions. Double ended black arrows represent reversible reactions. b) Establishment of the enantioselectively catalyzed, double 1,3‐dipolar cycloaddition of enones and azomethine ylide derived from amino acid ester imine **2 a**.

As an alternative to selective product formation, Rowan et al. introduced the concept of dynamic covalent chemistry (DCC).^[2]^ In DCC covalent bonds can be formed and broken reversibly in a fast equilibrium and under conditions in which equilibrium control leads to efficient formation of products under thermodynamic control.^[2]^ DCC has been proposed primarily in the context of supramolecular chemistry including applications in combinatorial chemistry.[^3^, ^4^] The reversible nature of reactions permits “error checking” and “proof reading” for interconverting components to access the thermodynamically most stable adduct.[^4^, ^5^, ^6^, ^7^] However, while in supramolecular chemistry weak noncovalent interactions dominate, for DCC more robust covalent bonds are relevant with slower kinetics of bond cleavage and formation. In DCC the relative stability of the products (i.e., thermodynamic parameters) determines the product distribution rather than the relative magnitudes of energy barriers of each pathway (i.e., kinetic parameters) (Scheme 1 a). Since both the thermodynamic and the kinetic parameters are functions of reaction parameters, the outcome is highly dependent on reaction conditions such as temperature, catalyst and reaction time required to reach an equilibrium.[^7^, ^8^] Turner et al. employed DCC in enzyme catalysis. They used an aldolase for preparation of a dynamic combinatorial library through stereoselective carbon‐carbon bond formation and enabled change in equilibrium in product distribution in presence of a thermodynamic trap.^[9]^

DCC was also observed for non‐enzymatic aldol/retro‐aldol reactions,[^5^, ^10^] for Diels–Alder cycloadditions at higher temperature,^[11]^ and for additional transformations including Michael additions,^[12]^ alkene cross‐metathesis.^[13]^ and [2+2] cycloaddition.^[14]^ However, non‐enzymatic enantioselective catalysis of DCC has not been reported and DCC has not been observed for dipolar cycloadditions yet. Herein we describe the discovery of catalytic and highly enantioselective dynamic covalent chemistry in metal complex‐catalyzed 1,3‐dipolar cycloaddition reactions, that is, a reaction type which belongs to the most important and widely used transformation in organic synthesis.

In the course of a program aimed at the synthesis of pseudo‐natural products,[^15^, ^16^, ^17^, ^18^, ^19^, ^20^, ^21^] which contain two or more natural product (NP) derived fragments in novel and structurally complex arrangements, we intended to combine two pyrrolidine fragments in an unprecedented manner. To this end, it was envisaged to subject cyclopentenones containing both an endocyclic and an exocyclic conjugated double bond (Scheme 1 b), to a sequence of two subsequent enantioselectively catalyzed 1,3‐dipolar cycloadditions with azomethine ylides generated in situ from imines.[^21^, ^22^, ^23^, ^24^, ^25^, ^26^]

## Results and Discussion

To establish the reaction sequence initial screening studies were conducted with cyclic enone (R=H) and glycine methyl ester imine **2 a** in dichloromethane in the presence of base (Scheme 1 b, Supplementary Table 1). We found that the double 1,3‐dipolar cycloaddition can be catalyzed efficiently with AgOAc to yield cycloadduct ***rac*** **‐3 a** with 82 % yield and high diastereoselectivity (*d.r*. >20:1). The regioselectivity of the first addition was investigated by treating cyclic enone (R=H) with 1.1 equiv of imine **2 a** under the same reaction conditions which resulted in the regioselective formation of ***rac*** **‐4 a** with high diastereoselectivity (>20:1) in 72 % yield (Scheme 1 b). A change in regioselectivity was observed when a methyl substituent was introduced at the endocyclic double bond of the dipolarophile (R=CH_3_). Monocycloaddition of cyclic enone and the ylide derived from glycine methyl ester imine **2 a** resulted in formation of spirocycle ***rac*** **‐4 b** in 57 % yield and with good diastereoselectivity (10:1). The product ***rac*** **‐4 a** reacts with glycine methyl ester imine **2 a** to form ***rac*** **‐3 a** with high diastereoselectivity (>20:1) and 75 % yield under the same reaction conditions.

**Table 1:** Results of the enantioselective synthesis of the chiral compounds **3**.^[a]^ 
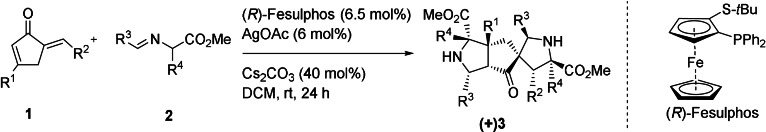

Product	R^1^	R^2^	R^3^	R^4^	Yield [%]^[b]^	*ee* ^[c]^
**3 a**	H	4‐Br‐C_6_H_4_	4‐Br‐C_6_H_4_	H	81	99
**3 b**	H	4‐Br‐C_6_H_4_	3‐Br‐C_6_H_4_	H	72	91
**3 c**	H	4‐Br‐C_6_H_4_	2‐Br‐C_6_H_4_	H	65	96
**3 d**	H	4‐Br‐C_6_H_4_	4‐Cl‐C_6_H_4_	H	81	98
**3 e**	H	4‐Br‐C_6_H_4_	4‐MeO‐C_6_H_4_	H	60	98
**3 f**	H	4‐Br‐C_6_H_4_	4‐F‐C_6_H_4_	H	75	98
**3 g**	H	4‐Br‐C_6_H_4_	4‐Me‐C_6_H_4_	H	63	93
**3 h**	H	4‐Br‐C_6_H_4_	2‐naphtyl	H	66	97
**3 i**	H	4‐Br‐C_6_H_4_	3‐furyl	H	40	95
**3 j**	H	4‐Br‐C_6_H_4_	2‐furyl	H	45	95
**3 k**	H	4‐Br‐C_6_H_4_	4‐Br‐C_6_H_4_	Ph	21	91
**3 l**	H	4‐NO_2_‐C_6_H_4_	4‐F‐C_6_H_4_	H	80	95
**3 m**	H	4‐NO_2_‐C_6_H_4_	3‐Br‐C_6_H_4_	H	72	91
**3 n**	H	4‐MeO‐C_6_H_4_	4‐F‐C_6_H_4_	H	65	98
**3 o**	H	4‐Me‐C_6_H_4_	4‐F‐C_6_H_4_	H	60	98
**3 p**	H	2‐naphtyl	4‐F‐C_6_H_4_	H	65	95
**3 q**	H	3‐Br‐C_6_H_4_	4‐Br‐C_6_H_4_	H	81	91
**3 r**	H	3‐Br‐C_6_H_4_	2‐Br‐C_6_H_4_	H	60	91
**3 s**	H	2‐Br‐C_6_H_4_	4‐Br‐C_6_H_4_	H	80	95
**3 t**	H	3‐Br‐C_6_H_4_	4‐MeO‐C_6_H_4_	H	55	91
**3 u**	H	4‐Cl‐C_6_H_4_	4‐Cl‐C_6_H_4_	H	80	95
**3 v**	H	^t^Butyl	4‐Br‐C_6_H_4_	H	21	93
**3 w**	H	3‐Br‐C_6_H_4_	4‐F‐C_6_H_4_	H	75	95
**3 x**	Me	4‐Br‐C_6_H_4_	4‐Br‐C_6_H_4_	H	40	93
**3 y**	H	4‐Br‐C_6_H_4_	[Fe(η^5^‐C_5_H_5_)( η^5^‐C_5_H_4_)]	H	48	94

[a] For General Procedure **A**, see Supplementary Methods. [b] Isolated yields of the pure major enantiomer after column chromatography. Diastereomeric ratio for all products is >20:1. [c] Determined by HPLC analysis using a chiral stationary phase. Me, methyl; Ph, phenyl.

In order to explore the enantioselective synthesis of tricyclic compound **3 a**, different chiral ligands, metal catalysts, bases and solvents were investigated (Supplementary Table 2). Optimization experiments indicated that the best result could be obtained using AgOAc (6 mol %) as a Lewis acid in combination with the *S*,*P*‐ferrocenyl ligand (*R*)‐Fesulphos, (**L1** 6.5 mol %) in dichloromethane using cesium carbonate (Cs_2_CO_3_; 40 mol %) as a base at room temperature after 24 h. Under these conditions, tricyclic compound **3 a** was obtained in 81 % yield with high diastereoselectivity (*d.r*. >20:1) and excellent enantioselectivity (99 % *ee*; Table 1). Notably, this reaction created eight stereocenters including one quaternary center in a one‐pot transformation. The absolute configuration of product **3 a** was determined by crystal structure analysis (Supporting Information, Crystal Data), which revealed that product **3 a** is formed by double *endo*‐selective cycloaddition.

**Table 2:** Results of the enantioselective synthesis of the tandem cycloadditions with two different imines.^[a]^ 
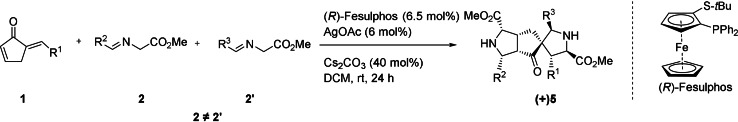

Product	R^1^	R^2^	R^3^	Yield [%]^[b]^	*ee* ^[c]^
5 a	4‐Cl‐C_6_H_4_	4‐Cl‐C_6_H_4_	4‐Br‐C_6_H_4_	77	85
5 b	4‐Cl‐C_6_H_4_	4‐Cl‐C_6_H_4_	4‐Me‐C_6_H_4_	65	82
5 c	4‐Cl‐C_6_H_4_	4‐Cl‐C_6_H_4_	4‐MeO‐C_6_H_4_	56	84
5 d	4‐Br‐C_6_H_4_	4‐Me‐C_6_H_4_	2‐naphtyl	75	89
5 e	4‐Cl‐C_6_H_4_	4‐Me‐C_6_H_4_	4‐MeO‐C_6_H_4_	70	85
5 f	4‐Cl‐C_6_H_4_	4‐Me‐C_6_H_4_	4‐Br‐C_6_H_4_	65	90
5 g	4‐Br‐C_6_H_4_	4‐Me‐C_6_H_4_	2‐Br‐C_6_H_4_	56	90
5 h	4‐Br‐C_6_H_4_	4‐Me‐C_6_H_4_	4‐MeO‐C_6_H_4_	69	89
5 i	4‐Br‐C_6_H_4_	4‐Me‐C_6_H_4_	4‐F‐C_6_H_4_	78	90
5 j	4‐Br‐C_6_H_4_	4‐Me‐C_6_H_4_	4‐Br‐C_6_H_4_	72	87

[a] General procedure B can be found in Supplementary Methods. [b] Isolated yields of the pure major enantiomer after column chromatography. Diastereomer ratio for all products is >20:1. [c] Determined by HPLC analysis using a chiral stationary phase. Me, methyl; Ph, phenyl.

The scope of the tandem reaction is wide. Regardless of the substitution pattern of the aromatic ring, enones **1** react with various azomethine ylides derived from imines **2** with moderate to good yields of 40–81 % with high diastereo‐ (>20:1) and enantioselectivity (91–99 % *ee*, **3 a**–**3 y** in Table 1). In the presence of electron‐donating substituents such as methyl‐ or methoxy groups (**3 e** and **3 g**, respectively), the yield was reduced to 60–63 % whereas electron‐neutral or ‐withdrawing substituents such as bromine or fluorine (**3 a** and **3 f**) led to higher yields. Heterocycle‐containing azomethine ylides reacted to provide products (**3 i** and **3 j**) in moderate yield and with high enantioselectivity (95 % *ee*). In general, the enantioselectivity of the double cycloaddition remained consistent regardless of substitution pattern. When an enone with a sterically hindering *tert‐butyl* substituent on the exocyclic olefin was employed, the double cycloaddition product (**3 v**) was obtained in 21 % yield, and high enantioselectivity was still observed (93 % *ee*). Furthermore, introduction of a methyl substituent to the endocyclic double bond of the enone yielded **3 x** with two quaternary centers with high enantioselectivity (93 % *ee)*.

A more hindered α‐phenyl substituted ylide, afforded product **3 k** in 21 % yield but with high enantioselectivity (91 % *ee*), thereby inducing formation of three quaternary centers. For all of examples shown in Table 1, the products were formed as single diastereomers (*d.r*. >20/1) after 24 h.

To rapidly expand the chemical space accessible via the double cycloaddition, we explored whether a sequential multicomponent transformation could give access to cycloadducts formed from two different imines. Following optimization, a sequence was established in which cyclic enones **1** were treated with 1.1 equiv of an iminoester **2** in the presence of (*R*)‐Fesulphos (**L1**, 6.5 mol %), AgOAc (6 mol %) and Cs_2_CO_3_ (40 mol %) in dichloromethane for 3 h (Supplementary Methods, procedure B) followed by treatment with a second, different iminoester **2′** for 24 h. Based on the different reactivity of the double bonds, the first cycloaddition occurs with the endocyclic double and the second cycloaddition targets the exocyclic double bond, which results in selective formation of mixed double cycloaddition products **5** by means of a sequential one‐pot, three‐component reaction (**5 a**–**5 j** in Table 2). The mixed double cycloaddition products **5** were obtained with high diastereoselectivity but, unexpectedly, with lower enantioselectivity (82–91 % *ee*) relative to the two‐component double‐cycloaddition products **3**, for which the products were formed with up to 99 % *ee*.

To explain the findings we hypothesized that both cycloadditions, that is, formation of **3** and **4**, may be the result of an enantioselectively catalyzed dynamic covalent process. In this process the double cycloaddition first proceeds reversibly through a kinetically controlled pathway due to the lower energy barrier of the transition state followed by an interconversion to the thermodynamically more stable product by a fast equilibrium process. To validate this mechanistic hypothesis changes in enantioselectivity of the double cycloaddition were investigated stepwise (Table 3, Table 5). Upon treatment of cyclic enone **1 a** with 1.1 equiv of iminoester **2 a** in the presence of (*R*)‐Fesulphos **L1** (6.5 mol %), AgOAc (6 mol %) and Cs_2_CO_3_ (40 mol %) in DCM, monoadduct **4 a** can be formed with varying enantioselectivity in the range of 70–95 % *ee* (Table 3, Supporting Information, Experimental studies). The lower and varying enantioselectivity for formation of monoadduct **4 a** in comparison to double cycloaddition product **3 a** (99 % *ee*, Table 1) indicates that, indeed, enantioselective retro‐cycloaddition catalyzed by the Ag/(*R*)‐Fesulphos complex may occur, which changes the enantiomeric ratio during the reaction. The decrease in enantioselectivity for formation of monoadduct **4 a** is even more obvious when the ratio of AgOAc and (*R*)‐Fesulphos is changed (Table 3). Treatment of cyclic enone **1 a** with 1.1 equiv of iminoester **2 a** in the presence of 12 mol % instead of 6.5 mol %(*R*)‐Fesulphos **L1,** AgOAc (6 mol %) and Cs_2_CO_3_ (40 mol %) in DCM, leads to formation of monoadduct **4 a** with 90–95 % *ee* after 3 h but with only 80 % *ee* after 16 h (Table 3, Supporting Information, Experimental studies).

**Table 3:** Results of the time dependent enantioselective synthesis of chiral mono‐adducts (±)**4**. 
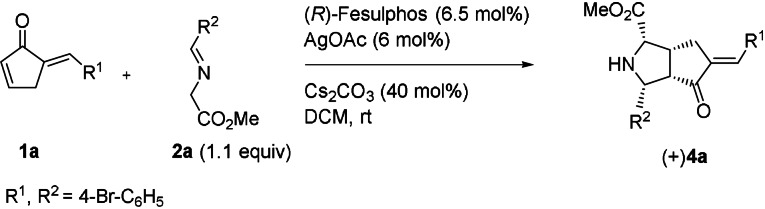

Entry	*t* [h]	Yield [%]	*ee* ^[b]^
1	3	59	95
2	12	59	87
3	24	59	84
4^[a]^	16	58	80
5^[a]^	24	57	70

[a] 12 mol % of (*R*)‐Fesulphos was used. [b] Determined by HPLC analysis using a chiral stationary phase.

**Scheme 2:**
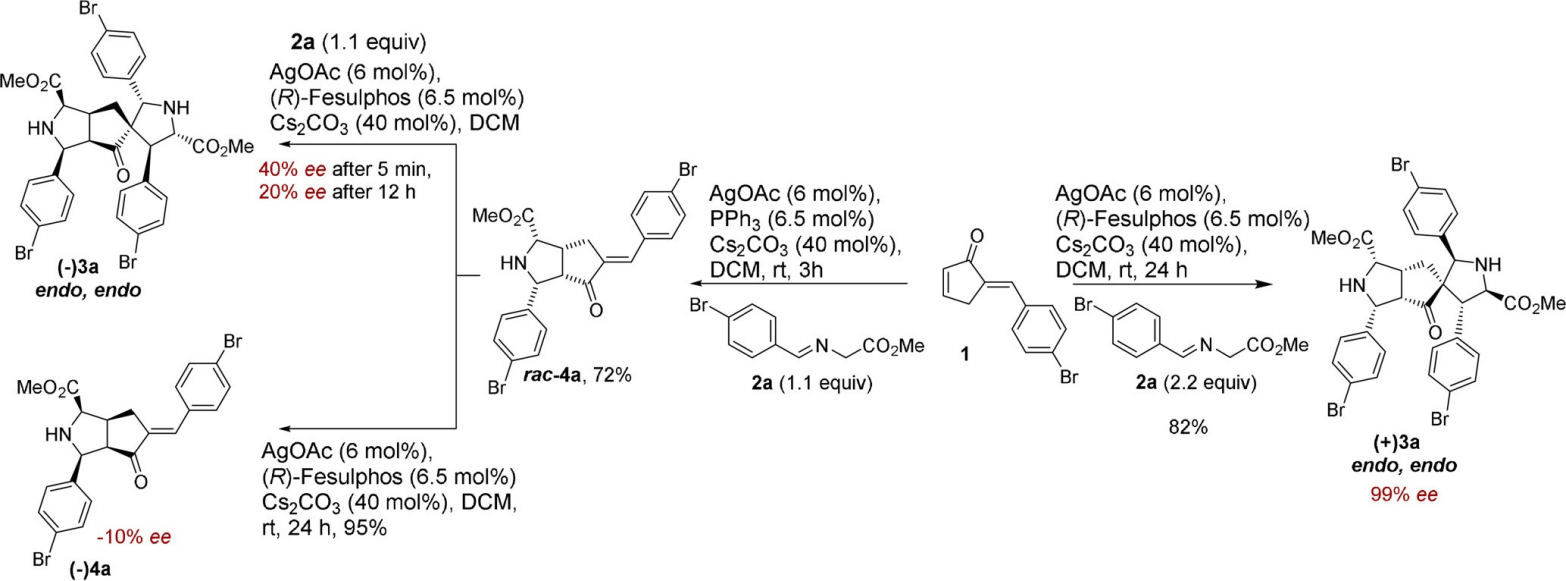
Establishment of stereodivergent synthesis in the enantioselectively catalyzed, double 1,3‐dipolar cycloaddition.

Cycloaddition product **3 a** is formed with 99 % *ee* regardless of the AgOAc/(*R*)‐Fesulphos ratio. These results indicate that one enantiomer of monoadduct **4 a** is involved in a retrocycloaddition as it matches with the chiral environment of the Ag/(*R*)‐Fesulphos complex in a selective manner. In order to confirm that retrocycloaddition is an enantioselective process, enantioenriched mono adduct **4 a** (81 % *ee*) was subjected to reaction with (*R*)‐Fesulphos **L1** (12 mol %), AgOAc (6 mol %) and Cs_2_CO_3_ (40 mol %) in DCM and a decrease of enantiomeric excess for the recovered mono adduct **4 a** was observed (74 % *ee*) (Table 4, Supporting Information, Experimental studies). Furthermore, racemic mono adduct **4 a** was treated under identical conditions and was recovered with −10 % *ee* (Table 4, Scheme 2).

**Table 4:** Treatment of mono‐adduct **4** using chiral catalyst. 
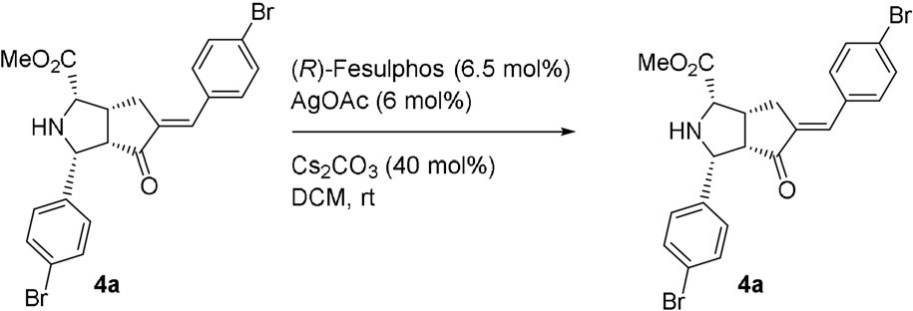

Entry	*ee* of **4 a** before reaction	*t* [h]	Yield [%]	*ee*^[a]^ of **4 a** after reaction
1	81	24	94	74
2	0 (*rac*)	24	95	−10

[a] Determined by HPLC analysis using a chiral stationary phase.

These results indicate that the mono addition reaction in the presence of Ag/(*R*)‐Fesulphos is a reversible process in which the forward cycloaddition is enantioselective whereas the retrocycloaddition undergoes kinetic resolution. Similarly investigations with enone **1 a** and imine **2 g** further confirmed a dynamic covalent mono cycloaddition (Supporting Information, Experimental studies).

For examination of the second cycloaddition in the sequence from **4 a** to **3 a** enantioenriched mono addition product **4 a** (70 % *ee*) was treated with 1 equiv of iminoester **2 a** under racemic conditions. No change in enantioselectivity was observed for the double cycloaddition product **3 a** (70 % *ee* Table 5, Supporting Information, Experimental studies), thereby confirming that the stereochemistry is already set in the first step of the reaction sequence. However, employing enantioenriched **4 a** (70 % *ee*) in the presence of a chiral catalyst led to a decrease in enantioselectivity for the double cycloaddition product **3 a** (60 % *ee*, Table 5; Supporting Information, Experimental studies).

**Table 5:** Catalytic 1,3‐dipolar cycloaddition of enantioenriched/racemic mono‐adduct **4**. 
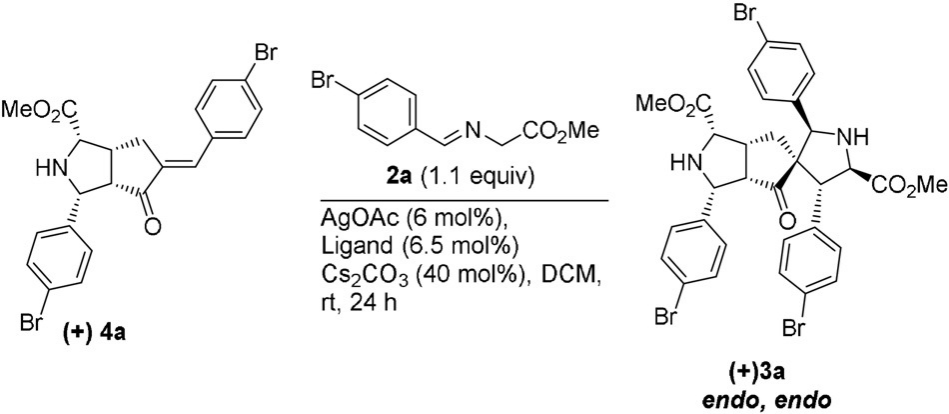

Entry	Ligand	*ee* of **4 a**	Yield [%]	*ee*^[a]^ of **3 a**
1	(*R*)‐Fesulphos	70	71	60
2	PPh_3_	70	75	70
3	(*R*)‐Fesulphos	0 (*rac*)	72	−20

[a] Determined by HPLC analysis using a chiral stationary phase.

Furthermore, when racemic mono addition product ***rac*** **‐4 a** was subjected to the cycloaddition in the presence of (*R*)‐FeSulphos and AgOAc with 1 equiv of iminoester **2 a**, double cycloaddition product **3 a** was obtained as an opposite enantiomer (−)**3 a** with 20 % *ee* (Table 5, Scheme 2) and was confirmed by crystal structure analysis (Supporting Information, Experimental studies, Crystal Data). The decreased enantioselectivity observed for **3 a** employing a chiral catalyst may be due the enantioselective retrocycloaddition of **4 a** as discussed above (Table 5).

**Scheme 3:**
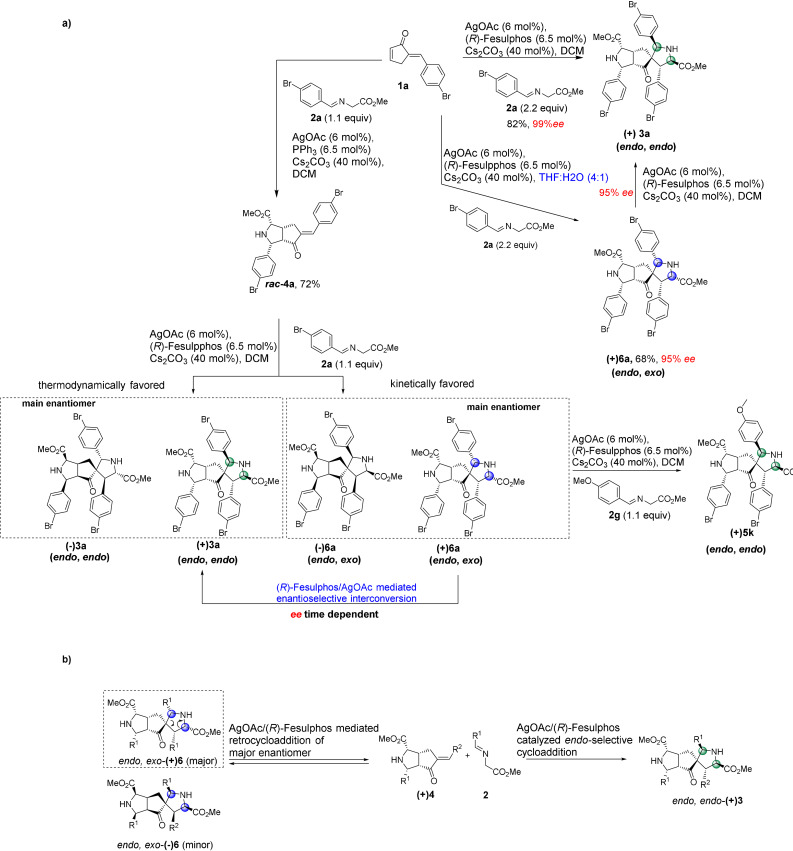
a) Establishment of the stereodivergent catalyzed, double 1,3‐dipolar cycloaddition of enone **1 a** and selected azomethine ylides and control of enantioselectivity. b) Proposed mechanism and intermediates for the AgOAc/(*R*)‐Fesulphos mediated retrocycloaddition and interconversion.

To further elucidate the reaction pathway, we performed a kinetic experiment by reacting ***rac*** **‐4 a** with imine **2 a** employing chiral catalyst (Supporting Information, Experimental studies, Supplementary Table 3). In the early stages of the reaction, **4 a** is rapidly converted into the *endo*, *endo*‐product (−)**3 a** and an *endo*, *exo*‐diastereomer **(+)6 a** (Scheme 3 a, Supporting Information, Experimental studies, Supplementary Table 3). X‐Ray analysis of the stereoisomeric *endo, exo*‐product **(+)6 a** revealed that it is formed through an *exo*‐selective cycloaddition of iminoester **2 a** to the *endo* monoaddition product **(+)4 a** (Supporting Information, Crystal Data). After three minutes, (−)**3 a** and (+)**6 a** are formed with noticeable *ee* (40 % and 37 % *ee*, respectively) (Supporting Information, Experimental studies, Supplementary Table 3). Over the course of the reaction, the *ee* of both products ((−)**3 a** and (+)**6 a**) decreases to 13 % and −13 % *ee*, respectively. Furthermore, the product ratio changes throughout the course of the reaction with more *endo*, *endo*‐product **3 a** being formed over time while the yield of the *endo*, *exo*‐product **6 a** decreases over time (Supporting Information, Experimental studies, Supplementary Table 3). A similar trend was observed by performing the kinetic experiment by subjecting racemic mono adduct ***rac*** **‐4 a** to cycloaddition with iminoester **2 g** under chiral conditions (Supporting Information, Experimental studies, Supplementary Table 4).

Since ***rac*** **‐4 a** is converted into two stereoisomers (−)**3 a** and (+)**6 a** with varying ratios and *ee*’s over time, it implies the formation of kinetically and thermodynamically controlled products during an enantioselective dynamic covalent process resulting in interconversion of those stereoisomers. (Scheme 3 a, Supporting Information Figure 1). The *endo*, *exo*‐stereoisomer (+)**6 a** is the kinetically favored product and is formed faster than the thermodynamically more stable product (−)**3 a**.

To explain the decrease in enantioselectivity and the change in product ratio over time, in accordance to the observation in Scheme 2, we investigated the stability of **(+)6 a**. Compound **(+)6 a** (37 % *ee*) was subjected to AgOAc (6 mol %), (*R*)‐Fesulphos (6.5 mol %) and Cs_2_CO_3_ (40 mol %) and was partially converted to the more stable and thermodynamically favored *endo*, *endo*‐product **(+)3 a** with 59 % *ee* (37 % yield) while **(+)6 a** was recovered with a lower *ee* of 20 % (51 % yield) (Scheme 3 a, Supporting Information, Experimental studies). This result provides further evidence that (+)**6 a** is labile under the reaction conditions and that the change of enantioselectivity is due to an enantioselective dynamic process in which the main enantiomer of the kinetically favored product ((+)**6 a**) is converted to the minor enantiomer ((+)**3 a**) of the thermodynamically stable product (Scheme 3 a/b). Furthermore, highly enantiopure *endo*, *exo*‐product **(+)6 a** (95 % *ee*) was treated under identical conditions and was converted to the stable thermodynamically favored *endo*, *endo*‐product (+)**3 a** (95 % *ee*, 92 % yield) with retention of enantioselectivity (Scheme 3 a).

To clarify through which intermediate the stereoisomeric *endo*, *exo*‐product (+)**6 a** is converted to product (+)**3 a**, compound **(+)6 a** (37 % *ee*) was treated with iminoester **2 g** (2 equiv) in the presence of AgOAc (6 mol %), (*R*)‐Fesulphos (6.5 mol %) and Cs_2_CO_3_ (40 mol %) in DCM. An imine exchange reaction was observed on the spirocycle affording product **5 k** in moderate yield (65 %) and 33 % *ee* (Scheme 3 a, Supporting Information, Experimental studies). The imine exchange on the spirocycle indicates that the transformation of *endo*, *exo*‐product (+)**6 a** to **5 k** proceeds through monoaddition product (+)**4 a** as an intermediate. Therefore, the thermodynamically less stable *endo*, *exo*‐product **6 a** is converted to mono addition product **4 a** through a retro cycloaddition followed by an endo‐selective cycloaddition with iminoester **2 g** to regenerate the spirocycle (Scheme 3 a, Supporting Information, Experimental studies). A chiral (Ag‐/(*R*)‐Fesulphos) mediated dynamic covalent process is occurring in which covalent bonds are cleaved through a retro cycloaddition and reformed again by cycloaddition.

We also investigated whether *endo*, *endo*‐product **3 a** can be converted to its stereoisomer *endo*, *exo*‐product **6 a**. Racemic *endo*, *endo* **3 a** was treated with AgOAc (6 mol %), (*R*)‐Fesulphos (6.5 mol %) and Cs_2_CO_3_ (40 mol %) with and without iminoester **2 a**. Compound **3 a** was not converted to product **6 a** which confirms the stability of *endo*, *endo*‐product **3 a** compared to its stereoisomeric *endo*, *exo*‐analog **6 a** under the reaction conditions (Supporting Information, Experimental studies).

These findings suggest that the double cycloaddition of enone **1** with imine **2** is in general a dynamic covalent process which can be divided in two cycles (Supporting Information, Figure 2). First, a Ag‐/(*R*)‐Fesulphos‐catalyzed mono addition occurs to generate the chiral mono addition product **4** in which the chirality is set. The initial enantiopurity of the mono addition is >95 % *ee*.

Over time, a selective retro‐cycloaddition for the major enantiomer of mono adduct **4** is catalyzed by Ag‐/(*R*)‐Fesulphos which induces its decomposition and results in a decreased *ee* of monoaddition product **4** from >95 % *ee* to 70–80 % *ee* (Table 3, Supporting Information, Figure 2). The main enantiomer of mono adduct **4** matches with the chiral environment of the Ag‐/(*R*)‐Fesulphos complex as its decomposition is faster compared to its enantiomer, resulting in decrease of *ee* for mono adduct. In a second cycle starting from the enantioenriched mono addition product **4**, a second cycloaddition takes place to generate a double cycloaddition product. Here, two diastereomers are formed, the kinetically favored non‐stable *endo, exo* product **6** and the thermodynamically controlled stable *endo*, *endo* product **3** (Scheme 3 a). Through a dynamic covalent process, enantioselective retro‐cycloaddition of non‐stable kinetic product *endo*, *exo* **‐6 a** occurs generating mono addition product **4**, which is then converted to the thermodynamically favored and stable *endo*, *endo* product **3** (Scheme 3 b, Supporting Information, Figure 2).

Finially we investigated whether changes in reaction conditions would enable a selective synthesis of kinetic diastereomer **6**. To this end, a solvent screen revealed that in the aqueous system THF:H_2_O (4:1) a switch in diastereoselctivity occurs. Treatment of enone **1 a** with 2.2 equiv of iminoester **2 a** in the presence of (*R*)‐Fesulphos **L1** (6.5 mol %) AgOAc (6 mol %) and Cs_2_CO_3_ (40 mol %) in this aqueous solvent system yields the *endo*, *exo*‐product **6 a** (95 % *ee*, 60 % yield) as the main diastereomer relative to *endo*, *endo* product (+)**3 a** (*d.r*. 4:1) (Table 6).

**Table 6:** Results of the enantioselective synthesis of the chiral endo‐exo adducts **6**.^[a]^ 
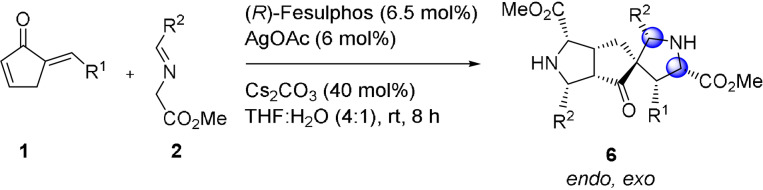

Product	R^1^	R^2^	Yield [%]^[b]^	*ee* ^[c]^	*d.r*.
**6 a**	4‐Br‐C_6_H_4_	4‐Br‐C_6_H_4_	65	95	4:1
**6 b**	4‐Br‐C_6_‐H_4_	4‐MeO‐C_6_H_4_	60	93	7:1
**6 c**	4‐Br‐C_6_H_4_	4‐F‐C_6_H_4_	51	97	4:1
**6 d**	4‐Br‐C_6_H_4_	2‐furyl	49	95	7:1
**6 e**	4‐Br‐C_6_H_4_	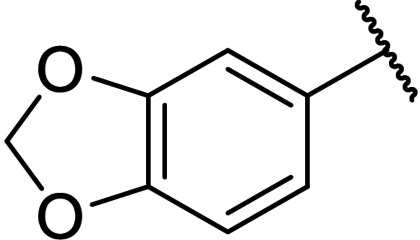	48	95	7:1
**6 f**	4‐Br‐C_6_H_4_	3‐Me‐C_6_H_4_	46	89	3:1
**6 g**	4‐Br‐C_6_H_4_	3‐furyl	52	95	6:1

[a] For General Procedure **A**, see Supplementary Methods. [b] Isolated yields of the pure major enantiomer after column chromatography. [c] Determined by HPLC analysis using a chiral stationary phase. Me, methyl; Ph, phenyl.

**Scheme 4:**
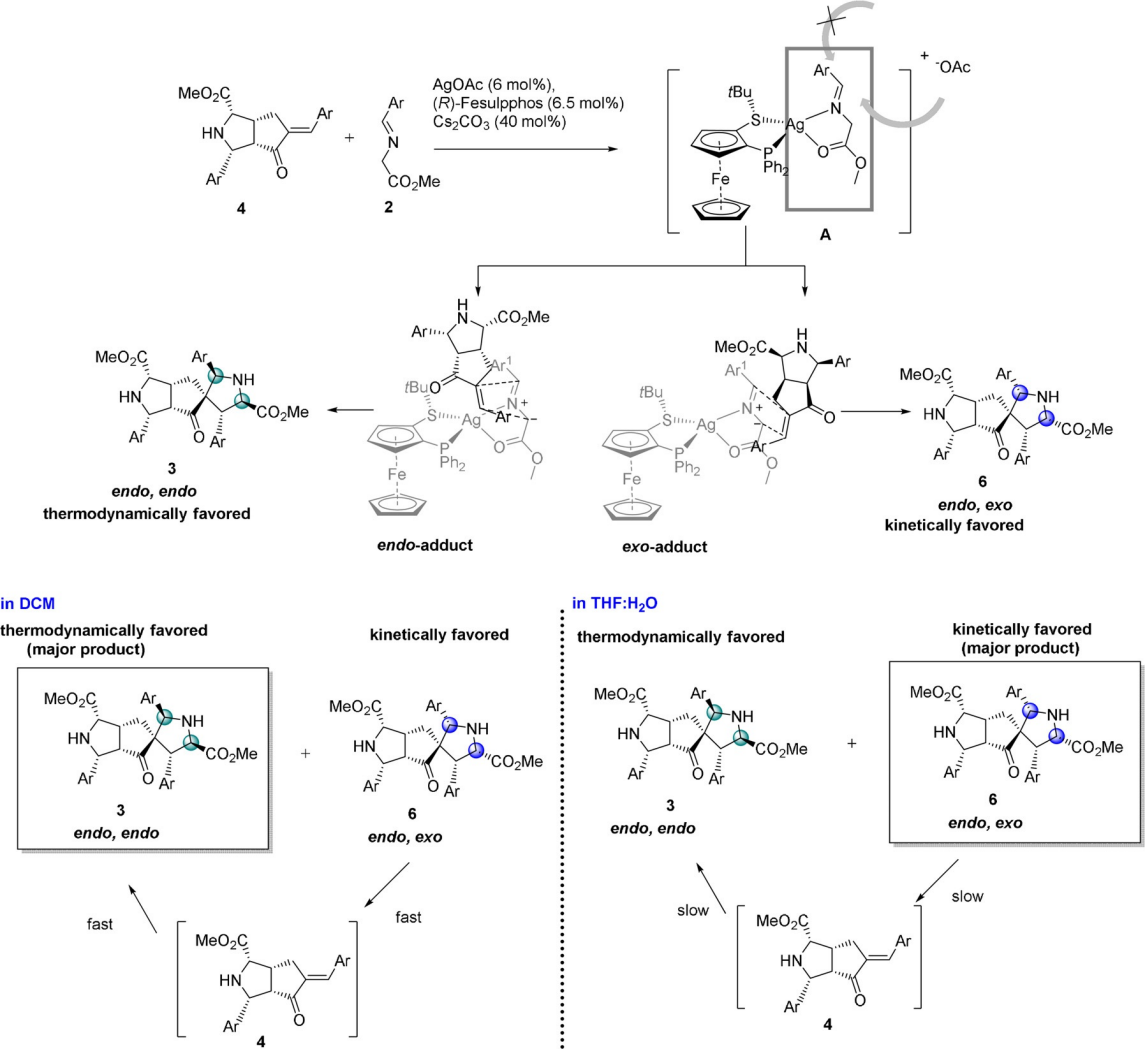
Proposed intermediates and transition states for formation of kinetically and thermodynamically favoured products **3** and **6** and differences in DCM and THF:H_2_O.

In contrast, in DCM the *endo*, *endo*‐product **3 a** is selectively obtained. The inversion in diastereoselectivity may be due to a more favorable *exo*‐selective transition state in the aqueous environment. Alternatively interconversion of **6 a** to **3 a** is much slower in the aqueous solvent system relative to DCM. The interconversion of the less stable kinetically favored *endo*, *exo* product **6** to the thermodynamically controlled product **3** might be a slow equilibrium process which results in the kinetically controlled *endo*, *exo* product as major diastereomer in THF:H_2_O (Scheme 4). Thus the speed of isomerization of the stereoisomers is decreased. In DCM the interconversion to reach the thermodynamically controlled equilibrium may proceed faster, since the retro cycloaddition from kinetically favored *endo, exo* product **6** to mono adduct **4** is more accelerated (Scheme 4). This was confirmed by analysis of the interconversion of the non‐stable kinetic product *endo*, *exo* **‐6 a** to its thermodynamically more stable *endo*, *endo* product **3 a** in both solvent systems. Subjecting Compound **(+)6 a** (95 % *ee*) to AgOAc (6 mol %), (*R*)‐Fesulphos (6.5 mol %) and Cs_2_CO_3_ (40 mol %) in DCM afforded **(+)3 a** with 37 % yield after 1 h, while only traces were observed in aqueous conditions after 1 h (Table 7). Expansion of the substrate scope confirmed the diastereoselectivity trend favoring **6** over its thermodynamically more stable stereoisomer **3** when an aqueous solvent system (THF:H_2_O) was employed (Table 6).

**Table 7:** Results of the time dependent enantioselective interconversion of enantioenriched endo, exo **(±)6** **a to** endo, endo **(±)3** **a**. 
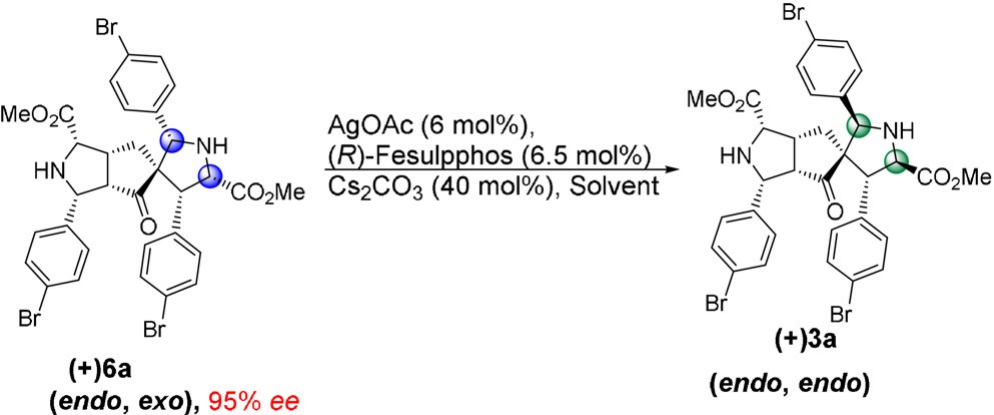

Entry	*t* [h]	Solvent	Yield [%]	*ee* ^[a]^
1	0.5	DCM	13	95
2	1	DCM	37	95
3	0.5	THF:H_2_O	no conversion	n.d.
4	1	THF:H_2_O	traces	n.d.

[a] Determined by HPLC analysis using a chiral stationary phase. n.d.=not detected.

## Conclusion

In summary, we have discovered an enantioselectively catalyzed dynamic covalent one‐pot‐tandem cycloaddition of azomethine ylides to α′‐alkylidene‐2‐cyclopentenones. High diastereo‐ and enantioselectivity was obtained for the formation of the double cycloaddition products in a thermodynamically controlled equilibrium process. A stereodivergent synthesis was achieved since a switch in diastereoselectivity is observed in aqueous solvent system (THF:H_2_O) and an enantiodivergent selectivity is recorded for reactions starting from racemic intermediate. Owing to differences in the reactivity of the *endo*‐ and *exo*cyclic double bonds of the α′‐alkylidene‐2‐cyclopentenones, two different dipoles could be successfully used in a one‐pot process, yielding structurally complex molecular frameworks with up to 8 stereocenters.

## Conflict of interest

The authors declare no conflict of interest.

## Supporting information

As a service to our authors and readers, this journal provides supporting information supplied by the authors. Such materials are peer reviewed and may be re‐organized for online delivery, but are not copy‐edited or typeset. Technical support issues arising from supporting information (other than missing files) should be addressed to the authors.

Supporting Information
