# Supplementary material for: Dynamic Catalytic Highly Enantioselective 1,3‐Dipolar Cycloadditions
Source: Angew Chem Int Ed Engl. 2021 Aug 3;60(36):20012–20. doi: 10.1002/anie.202108072 (PMC8456807; doi:10.1002/anie.202108072)
Supplement: Supplementary file 1 — Supporting Information [file ANIE-60-20012-s001.pdf]

Angewandte Chemie
Eine Zeitschrift der Gesellschaft Deutscher Chemiker

Supporting Information

Dynamic Catalytic Highly Enantioselective 1,3-Dipolar Cycloadditions

Okan Yildirim, Michael Grigalunas, Lukas Brieger, Carsten Strohmann, Andrey P. Antonchick, and Herbert Waldmann**

anie_202108072_sm_miscellaneous_information.pdf

Table of Contents

Supplementary Tables *2*

Supplementary Table 1 2

Supplementary Table 2. 3

Supplementary Table 3 5

Supplementary Table 4 6

Supplementary Figures *7*

Supplementary Figure 1 7

Supplementary Figure 2 8

Supplementary Methods *9*

Synthesis of cyclic enone 1 10

Synthesis of iminoesters 2 15

General procedures for 1,3-dipolar cycloadditions 20

Procedure A 20

Procedure B 21

Procedure C 22

Procedure D 23

Characterization of cycloaddition products 3-6. .... *24*

Experimental studies on the enantioselectivity of cycloaddition *71*

Experimental studies on racemic mono adduct rac-4a *99*

Crystal data and structure for (+)3a *121*

Crystal data and structure for (+)3e *122*

Crystal data and structure for (-)3e *123*

Crystal data and structure for 6a *124*

Crystal data and structure for 6b *125*

References *126*

Copies of spectral data *127*

Supplementary Tables

Supplementary Table 1

Optimization of the reaction conditions for the catalytic 1,3-dipolar cycloaddition of enone **1a** with azomethine ylide **2a**.*

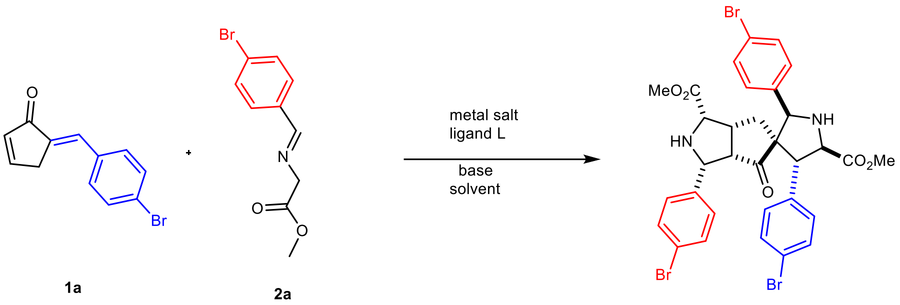

3a-rac

Entry	Catalyst	Catalyst (mol%)	Ligand	Ligand (mol%)	Base (40 mol%)	Solvent	Time (h)	d.r. †	Yield‡ (%)
1	$Cu(CH_3CN)_4PF_6$	6	PPh_3	6.5	Et_3N	DCM	24	>20:1	25
2	$Cu(CH_3CN)_4PF_6$	6	-	6.5	Et_3N	DCM	24	>20:1	20
3	$Cu(CH_3CN)_4PF_6$	6	PPh_3	6.5	Et_3N	THF	48	n.d.	trace
4	$Cu(CH_3CN)_4PF_6$	6	PPh_3	6.5	Cs_2CO_3	DCM	24	n.d.	trace
5	$Cu(CH_3CN)_4PF_6$	6	PPh_3	6.5	Et_3N	PhMe	12	>20:1	17
6	AgOAc	6	PPh_3	6.5	Cs_2CO_3	DCM	24	>20:1	81
7	AgOAc	6	PPh_3	6.5	K_2CO_3	DCM	24	n.d.	trace
8	$Cu(CH_3CN)_4BF_4$	6	PPh_3	6.5	Cs_2CO_3	DCM	24	>20:1	15
9	AgOTf	6	PPh_3	6.5	Cs_2CO_3	DCM	48	n.d.	n.d.
10	AgOAc	6	PPh_3	6.5	Cs_2CO_3	Et_2O	24	>20:1	30
11	$AgSbPF_6$	6	PPh_3	6.5	Cs_2CO_3	Et_2O	24	n.d.	n.d.

*Reaction conditions: ligand PPh_3, catalyst, base (40 mol%, 0.048 mmol), iminoester **2a** (2.2 equiv., 0.26 mmol) and cyclic enone **1a** (1 equiv., 0.12 mmol), ambient temperature. †Determined by 1H NMR spectroscopy. ‡Isolated yields of **3a-rac** after column chromatography. †d.r. – diastereomer ratio determined by ^{1}H-NMR spectroscopy, n.d. – not determined.

Supplementary Table 2.

Optimization of the reaction conditions for the enantioselective 1,3-dipolar cycloaddition of enone **1a** with azomethine ylide **2a**.*

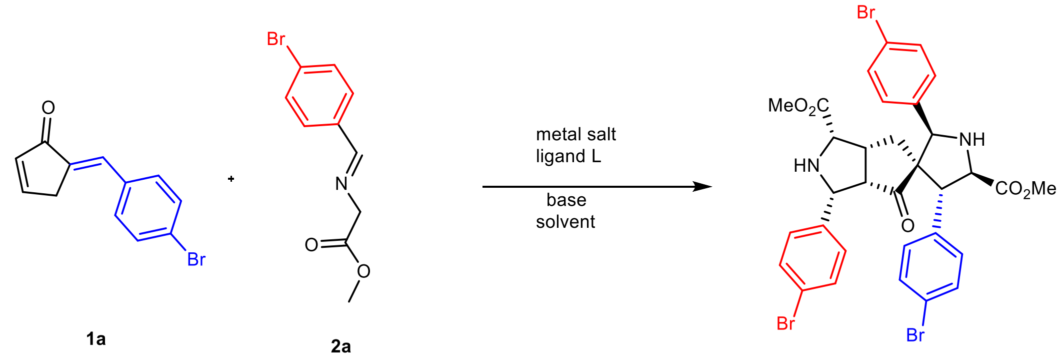

1a **2a** **3a**

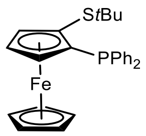

L1

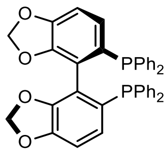

L2

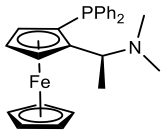

L3

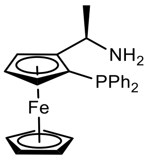

L4

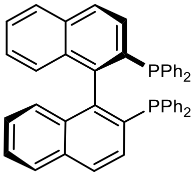

L5

Entry	Catalyst	Catalyst (mol%)	Ligand	Ligand (mol%)	Base (40 mol%)	Solvent	Time (h)	d.r. †	Yield ‡ (%)	e.e.§
1	$Cu(CH_3CN)_4PF_6$	6	**L1**	6.5	Et_3N	DCM	24	>20:1	33	81
2	$Cu(CH_3CN)_4PF_6$	6	**L2**	6.5	Et_3N	DCM	24	>20:1	20	55
3	$Cu(CH_3CN)_4PF_6$	6	**L3**	6.5	Et_3N	DCM	48	n.d.	trace	n.d.
4	$Cu(CH_3CN)_4PF_6$	6	**L4**	6.5	Et_3N	DCM	24	>20:1	40	70
5	$Cu(CH_3CN)_4PF_6$	6	**L5**	6.5	Et_3N	DCM	24	>20:1	25	30
6	AgOAc	6	**L1**	6.5	Et_3N	DCM	24	>20:1	27	65
**7**	**AgOAc**	**6**	**L1**	**6.5**	**Cs_2CO_3**	**DCM**	**24**	**>20:1**	**81**	**99**
8	AgOAc	6	**L1**	6.5	K_2CO_3	DCM	24	n.d.	trace	n.d.
9	$Cu(CH_3CN)_4BF_4$	6	**L1**	6.5	Cs_2CO_3	DCM	24	>20:1	15	7
10	AgOTf	6	**L1**	6.5	Cs_2CO_3	DCM	48	n.d.	trace	n.d.
11	$Cu(CH_3CN)_4PF_6$	6	**L1**	6.5	Cs_2CO_3	DCM	24	>20:1	27	20
12	$AgSbPF_6$	6	**L1**	6.5	Cs_2CO_3	DCM	24	n.d.	trace	n.d.
13	AgOAc	6	**L1**	6.5	Cs_2CO_3	THF	16	>20:1	30	80
14	AgOAc	6	**L1**	6.5	Cs_2CO_3	Et_2O	16	>20:1	37	11
15	AgOAc	6	**L1**	6.5	Cs_2CO_3	PhMe	16	>20:1	20	9

*Reaction conditions: ligand **L1-L5**, catalyst, base (40 mol%, 0.048 mmol), iminoester **2a** (2.2 equiv., 0.26 mmol) and cyclic enone **1a** (1 equiv., 0.12 mmol), ambient temperature. †Determined by ^{1}H NMR spectroscopy. ‡Isolated yields of **3a** after column chromatography. §Determined by HPLC analysis on chiral phase. †d.r. – diastereomer ratio determined by ^{1}H-NMR spectroscopy, n.d. –not determined.

Supplementary Table 3
Enantioselective 1,3-dipolar cycloaddition of racemic mono adduct ***rac*-4a** with azomethine ylide **2a** and time-related changes in enantioselectivity and diastereoselectivity.

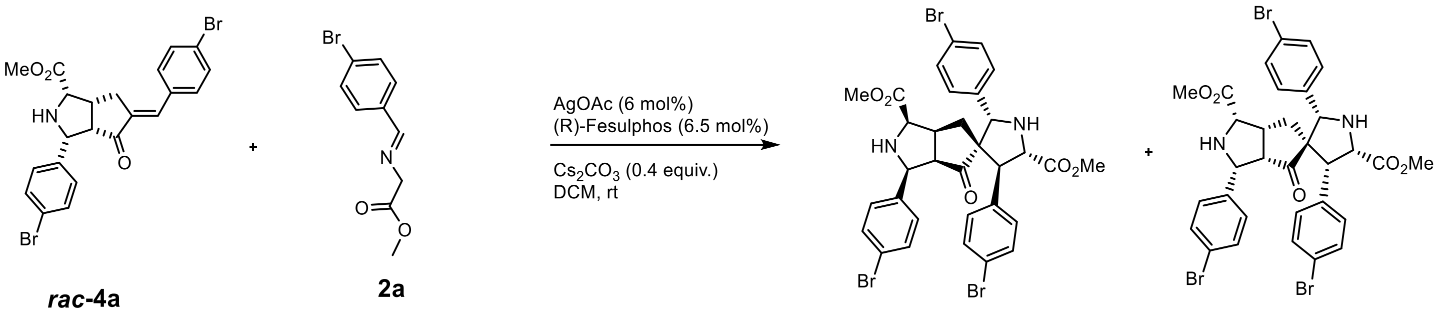

rac*-4a** (0.2 mmol) + **2a** 1.1 equiv. → **(-)3a** ***endo, endo + **6a** ***endo, exo***

time	**(-)3a** e.e. § (%)	**(-)3a**Yield‡ (%)	**6a** e.e. § (%)	**6a** Yield‡ (%)	Ratio **(-)3a/6a**
3 min	40	29	37	47	1/1.5
7 min	33	34	31	43	1/1.2
15 min	30	45	29	42	1/1.2
1 h	13	66	-13	15	1/0.3

Reaction was performed in 6 ml of DCM and 0.2 mmol of SM. Each time 1.5 ml of the reaction mixture was taken, purified and analyzed. ‡Isolated yields of (-)**3a** and **6a** after column chromatography based on **0.05 (**4 aliquots were taken on specific time points from 0.2 mmol SM) mmol SM. §Determined by HPLC analysis on chiral phase.

Supplementary Table 4

Enantioselective 1,3-dipolar cycloaddition of racemic mono adduct ***rac*-4c** with azomethine ylide **2g** and time-related changes in enantioselectivity and diastereoselectivity.

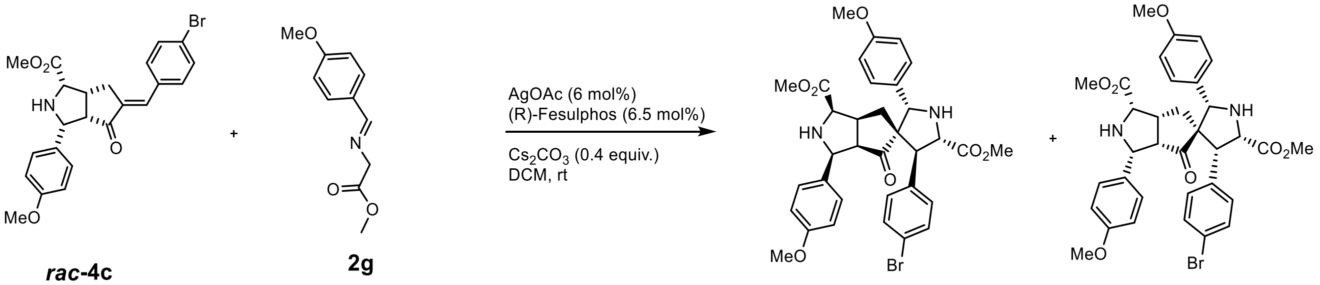

rac*-4c** (0.22 mmol) + **2g** 1.1 equiv. → **(-)3e** ***endo, endo + **6b** ***endo, exo***

time	**(-)3e** e.e. § (%)	**(-)3e**Yield‡ (%)	**6b** e.e. § (%)	**6b** Yield‡ (%)	Ratio **(-)3e/6b**
3 min	55	31	55	47	1/1.5
7 min	43	34	35	44	1/1.2
15 min	40	34	35	44	1/1.2
30 min	35	44	31	37	1.8/1.0
1 h	11	80	----	----	>20:1

Reaction was performed in 7.5 ml of DCM and 0.22 mmol of SM. Each time 1.5 ml of the reaction mixture was taken, purified and analyzed. ‡Isolated yields of (-)**3a** and **6a** after column chromatography based on **0.04** mmol SM. §Determined by HPLC analysis on chiral phase.

Supplementary Figures

Supplementary Figure 1

Kinetic experiment for enantioselective 1,3-dipolar cycloaddition of racemic monoadduct **4a**

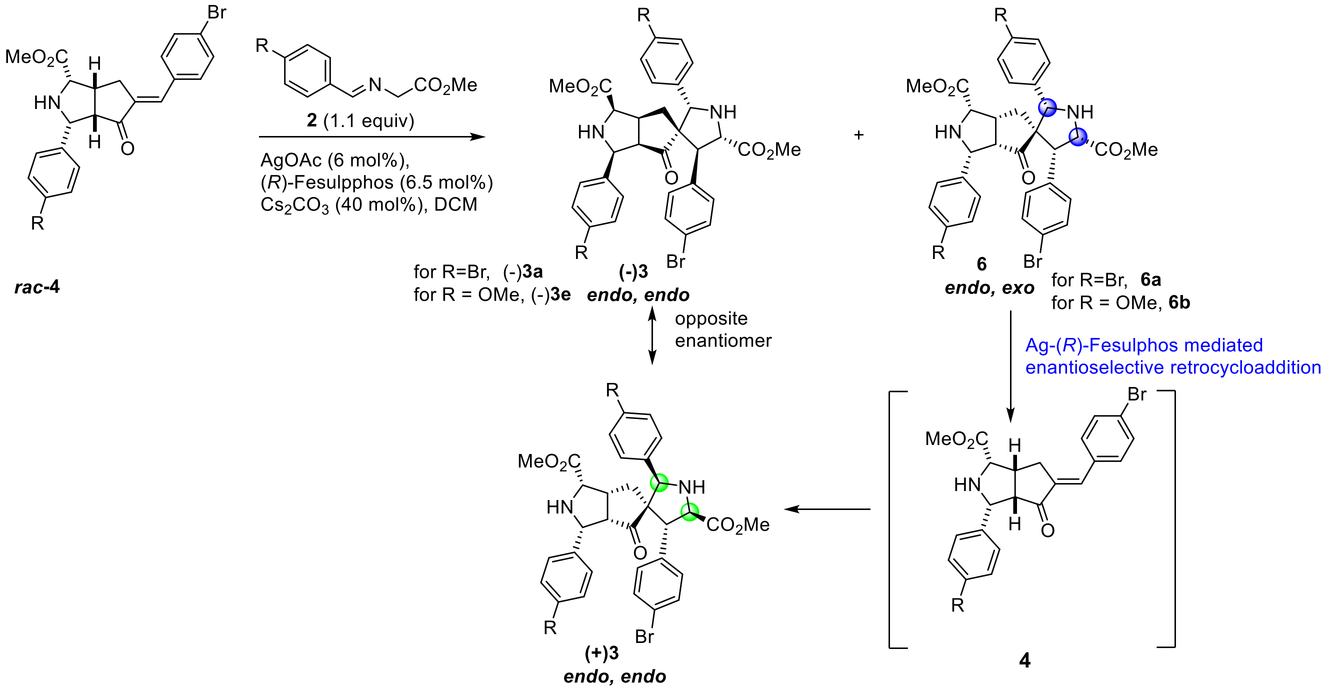

Treating racemic mono adduct **4** under chiral conditions (AgOAc/(*R*)-Fesulphos) leads to formation of two stereoisomers, *endo, endo* product **(-)3** (opposite enantiomer and *endo, exo* product **6** with time dependent enantioselectivity and varying ratio. The *endo, exo* product **6** is the kinetically favored product and is converted during course of reaction through a AgOAc-(*R*)-Fesulphos mediated catalyzed reaction to the thermodynamically stable stereoisomer *endo, endo* product **(+)3** by retro cycloaddition and formation of intermediate product **4**. The conversion of *endo, exo* product **6** to its stereoisomer **(+)3** also explains the decrease in ee overt time of *endo, endo* product **(-)3**.

Supplementary Figure 2

Proposed mechanism for the stereochemical course for the enantioselective double 1,3 dipolar cycloaddition of enone **1** and azomethine ylide **2**.

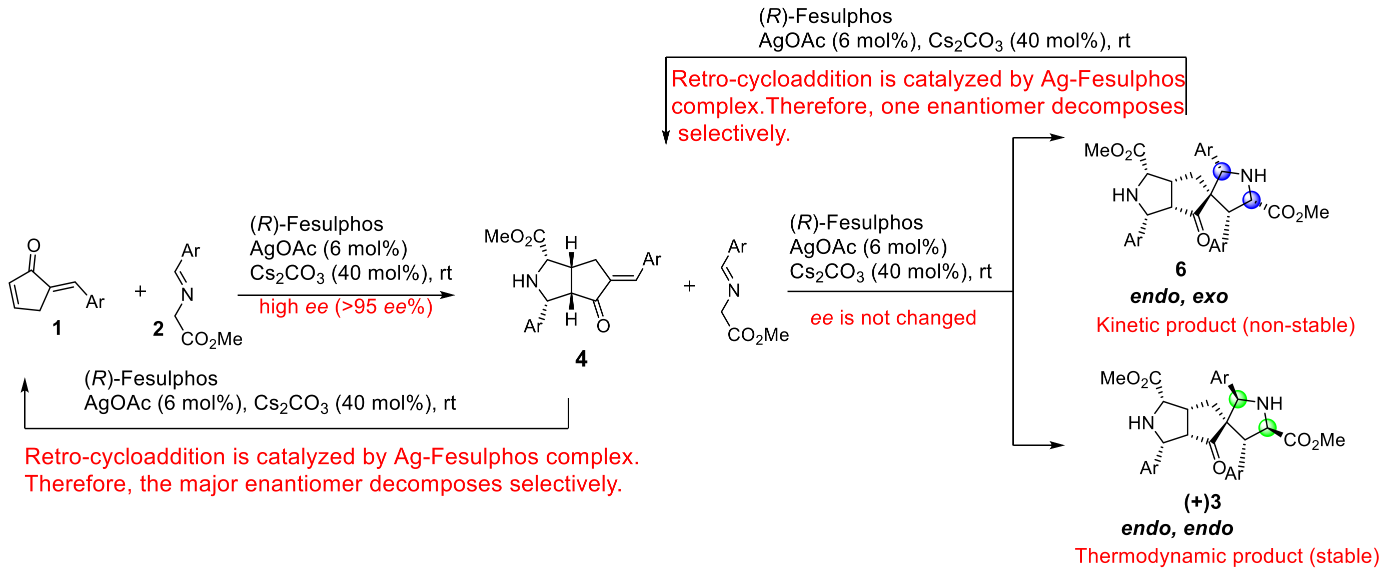

Supplementary Methods

Unless otherwise noted, all commercially available compounds were used as provided without further purifications. Dry solvents (THF, toluene, DCM) were used as commercially available. Solvents for chromatography were technical grade.

Analytical thin-layer chromatography (TLC) was performed on *Merck silica gel aluminium plates* with F-254 indicator. Compounds were visualized by irradiation with UV light or potassium permanganate staining. Column chromatography was performed using *silica gel Merck 60* (particle size 0.040-0.063 mm). Solvent mixtures are understood as volume/volume.

^{1}H-NMR and ^{13}C-NMR were recorded on a *Bruker DRX400* (400 MHz), *Bruker DRX500* (500 MHz) and *INOVA500* (500 MHz) using $CDCl_3$ or CD_2Cl_2 as solvent. Data are reported in the following order: chemical shift (δ) values are reported in ppm with the solvent resonance as internal standard ($CDCl_3$: δ = 7.26 ppm for 1H, δ = 77.16 ppm for ^{13}C; CD_2Cl_2: δ = 5.32 ppm for ^{1}H, δ = 53.8 ppm for ^{13}C); multiplicities are indicated br s (broadened singlet), s (singlet), d (doublet), t (triplet), q (quartet) m (multiplet); coupling constants (*J*) are given in Hertz (Hz).

High resolution mass spectra were recorded on a *LTQ Orbitrap* mass spectrometer coupled to an *Acceka HPLC*-System (HPLC column: *Hypersyl GOLD*, 50 mm x 1 mm, particle size 1.9 µm, ionization method: electron spray ionization). Fourier transform infrared spectroscopy (FT-IR) spectra were obtained with a *Bruker Tensor 27* spectrometer (ATR, neat) and are reported in terms of frequency of absorption (cm^{-1}). Optical rotations were measured in a *Schmidt + Haensch Polartronic HH8* polarimeter.

The enantiomeric excesses were determined by HPCL analysis using a chiral stationary phase column (column: CHIRALCEL IC, eluent: (*iso*-hexane/ *iso*-propanol). The chiral HPLC methods were calibrated with the corresponding racemic mixtures. The ratio of diastereomers was determined by ^{1}H-NMR analysis via integration of characteristic signals of methyl esters. Chemical yields refer to pure isolated substances. Yields and enantiomeric excesses, diastereoselectivity are given in the tables. The chemicals and solvents were purchased from the companies Sigma-Aldrich, Acros Organic, ABCR and Alfa Aesar. (*Rp*)-2-(*tert*-Butylthio)-1-(diphenyl-phosphino)ferrocene (purity: 98%), Tetrakis(acetonitrile)copper(I) hexafluorophosphate (purity; 97%) and Tetrakis(acetonitrile)copper(I) tetrafluoroborate (purity: 97%) were purchased from Sigma-Aldrich.

Synthesis of cyclic enone 1

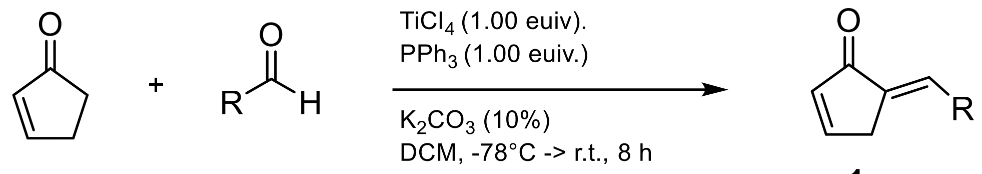

1

To a solution of the cyclic enone (1.0 equiv.) and triphenylphosphine (1.0 equiv.) in DCM (15 ml) was added titanium tetrachloride (1 equiv.) at -78 °C. After 15 min, the mixture was treated with RCHO (1.0-3.0 equiv.). The mixture was allowed to warm to room temperature over 8 h and then treated with 10% aqueous K_2CO_3. The organic layer was concentrated in vacuum and the residue was purified by flash chromatography on silica gel (petroleum ether/EtOAc) to give alkylidene cyclic enone **1**.[1, 2]

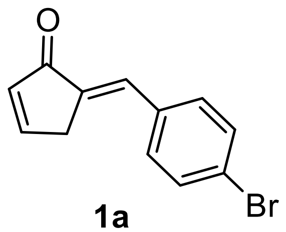

1a

(*E*)-5-(4-bromobenzylidene)cyclopent-2-en-1-one[1, 2]

90% yield, **^{1}H NMR** (400 MHz, CD_2Cl_2): δ 7.66-7.70 (m, 1H), 7.55 (d, *J* = 8.5 Hz, 2H), 7.43 (d, *J* = 8.5 Hz, 2H), 7.33 (s, 1H), 6.48 (m, 1H), 3.54 (d, *J* = 6.5 Hz, 2H);

^{13}C NMR (126 MHz, CD_2Cl_2) δ 196.83, 157.39, 135.12, 134.26, 133.28, 132.05, 131.78, 129.98, 123.61, 34.21.

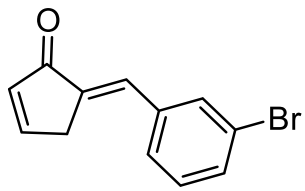

1b

(*E*)-5-(3-bromobenzylidene)cyclopent-2-en-1-one

72% yield**,** ^{1}**H NMR** (500 MHz, CD_2Cl_2) δ 7.76 – 7.70 (m, 2H), 7.58 – 7.48 (m, 2H), 7.33 (d, *J* = 7.9 Hz, 1H), 7.26 (d, *J* = 7.9 Hz, 1H), 6.45 – 6.42 (m, 1H), 3.56 (d, *J* = 6.3 Hz, 2H);

^{13}**C NMR** (126 MHz, CD_2Cl_2) δ 197.23, 158.10, 137.96, 135.62, 134.45, 133.32, 132.71, 130.95, 130.14, 129.51, 123.34, 34.68.

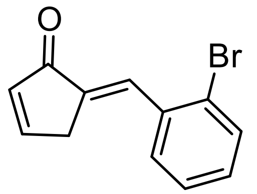

1c

(*E*)-5-(2-bromobenzylidene)cyclopent-2-en-1-one

56% yield, ^{1}**H NMR** (500 MHz, CD_2Cl_2) δ 7.69 – 7.58 (m, 3H), 7.33 – 7.29 (m, 3H), 6.44-6.40 (m, 1H), 3.49 (d, *J* = 6.5 Hz, 2H);

^{13}**C NMR** (126 MHz, CD_2Cl_2) δ 205.56, 158.46, 150.79, 138.25, 135.75, 133.92, 130.95, 130.42, 130.31, 128.10, 127.96, 34.09.

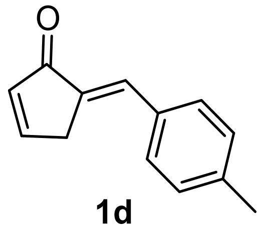

1d

(*E*)-5-(4-methylbenzylidene)cyclopent-2-en-1-one[1]

79% yield, ^{1}**H NMR** (500 MHz, CD_2Cl_2) δ 7.68 (s, 1H), 7.51 (d, *J* = 8.2 Hz, 2H), 7.31 (d, *J* = 6.2 Hz, 1H), 7.25 (d, *J* = 8.2 Hz, 2H), 6.42-6.38 (m, 1H), 3.56 (d, *J* = 6.2 Hz, 2H), 2.38 (s, 3H);

^{13}**C NMR** (126 MHz, CD_2Cl_2) δ 197.65, 157.67, 140.60, 134.13, 132.97, 132.27, 131.86, 130.95, 130.14, 34.94, 21.71.

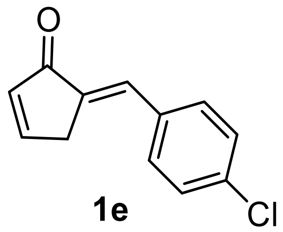

1e

(*E*)-5-(4-chlorobenzylidene)cyclopent-2-en-1-one[2]

84% yield, ^{1}**H NMR** (500 MHz, CD_2Cl_2) δ 7.73 – 7.67 (m, 1H), 7.54 (d, *J* = 8.6 Hz, 2H), 7.41 (d, *J* = 8.6 Hz, 2H), 7.29 (s, 1H), 6.42-6.39 (m, 1H), 3.54 (d, *J* = 6.3 Hz, 2H);

^{13}**C NMR** (126 MHz, CD_2Cl_2) δ 197.34, 157.89, 135.65, 134.39, 133.66, 132.10, 130.43, 129.59, 34.73.

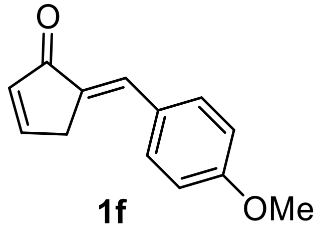

1f

(*E*)-5-(4-methoxybenzylidene)cyclopent-2-en-1-one

75% yield, ^{1}**H NMR** (500 MHz, CD_2Cl_2) δ 7.83(d, *J* = 8.4 Hz, 1H), 7.67 (s, 1H), 7.57 (d, *J* = 8.3 Hz, 2H), 6.96 (d, *J* = 8.3 Hz, 2H), 6.41 – 6.38 (m, 1H), 3.84 (s, 3H), 3.55 (d, *J* = 6.5 Hz, 2H);

^{13}**C NMR** (126 MHz, CD_2Cl_2) δ 197.64, 157.34, 135.73, 132.69, 132.31, 131.61, 128.42, 114.86, 114.80, 55.91, 34.93.

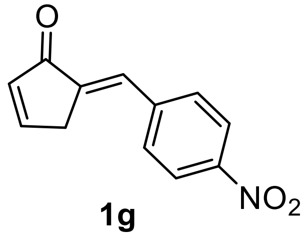

1g

(*E*)-5-(4-nitrobenzylidene)cyclopent-2-en-1-one

53% yield, ^{1}**H NMR** (500 MHz, CD_2Cl_2) δ 8.26 (d, *J* = 8.8 Hz, 2H), 7.75 (m, 3H), 7.39 (s, 1H), 6.50 – 6.43 (m, 1H), 3.61 (d, *J* = 6.3 Hz, 2H);

^{13}**C NMR** (126 MHz, CD_2Cl_2) δ 196.94, 158.44, 148.20, 142.18, 136.69, 135.64, 131.33, 129.14, 124.47, 34.66.

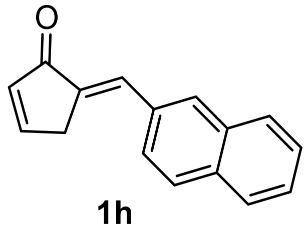

1h

(*E*)-5-(naphthalen-2-ylmethylene)cyclopent-2-en-1-one

66% yield, ^{1}**H NMR** (500 MHz, CD_2Cl_2) δ 8.00 – 7.85 (m, 4H), 7.77 – 7.70 (m, 2H), 7.63 – 7.47 (m, 3H), 6.45-6.40 (m, 1H), 3.69 (d, *J* = 6.2 Hz, 2H);

^{13}**C NMR** (126 MHz, CD_2Cl_2) δ 197.54, 157.91, 135.72, 134.28, 133.87, 133.44, 133.39, 131.95, 131.77, 129.03, 128.99, 128.16, 127.79, 127.20, 54.22, 35.02.

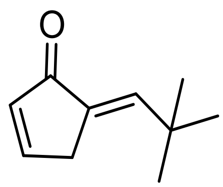

1i

(E)-5-(2,2-dimethylpropylidene)cyclopent-2-en-1-one[2]

48% yield, **^{1}H NMR** (500 MHz, CD_2Cl_2) δ 7.69 (s, 1H), 6.55 (d, J = 6.0 Hz, 1H), 6.33-6.31 (m, 1H), 3.37 (d, J = 6.2 Hz, 2H), 1.18 (s, 9H);

^{13}C NMR (126 MHz, CD_2Cl_2) δ 198.19, 158.46, 145.76, 135.40, 130.67, 83.02, 33.46, 29.93.

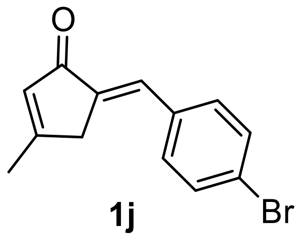

1j

(E)-5-(4-bromobenzylidene)-3-methylcyclopent-2-en-1-one

57% yield, **^{1}H NMR** (500 MHz, CD_2Cl_2) δ 7.56 (d, J = 8.5 Hz, 2H), 7.45 (d, J = 8.5 Hz, 2H), 7.19 (s, 1H), 6.15 (s, 1H), 3.43 (s, 2H), 2.22 (s, 3H);

^{13}C NMR (126 MHz, CD_2Cl_2) δ 197.22, 172.37, 135.60, 135.02, 132.51, 132.17, 131.77, 128.65, 123.77, 38.85, 19.45.

Synthesis of iminoesters 2

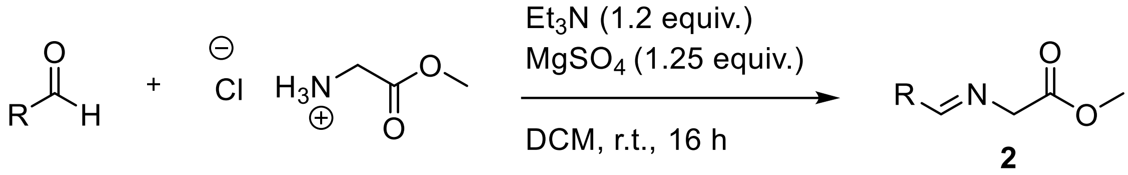

To a suspension of amino acid ester hydrochloride (1.2 equiv., 12 mmol) and $MgSO_4$ (1.25 equiv., 12.5 mmol) in DCM (15 mL) was added Et_3N (1.2 equiv., 12 mmol). The mixture was stirred at ambient temperature for 1h. Then the corresponding aldehyde (1 equiv., 10 mmol) was added and the mixture was allowed to stir at ambient temperature overnight. The precipitate was removed by filtration and the filtrate was washed with water (15 mL). The aqueous phase was extracted two times with DCM (10 mL) and the combined organic layer was washed once with brine (15 mL), dried over $MgSO_4$ and concentrated. The iminoesters were used for 1,3-dipolar cycloadditions and NMR studies without further purification.[3]

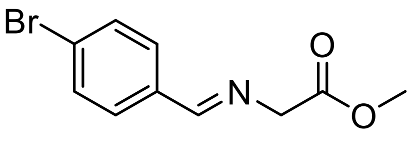

2a

Methyl (*E*)-2-((4-bromobenzylidene)amino)acetate[4]

93% yield, **^{1}H NMR** (500 MHz, CD_2Cl_2) δ 8.24 (t, *J* = 1.4 Hz, 1H), 7.65 (d, *J* = 8.5 Hz, 2H), 7.58 (d, *J* = 8.5 Hz, 2H), 4.37 (d, *J* = 1.4 Hz, 2H), 3.74 (s, 3H);

^{13}C NMR (126 MHz, CD_2Cl_2) δ 170.82, 164.41, 135.24, 132.42, 130.30, 126.04, 62.21, 52.49.

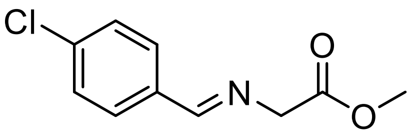

2b

Methyl (*E*)-2-((4-chlorobenzylidene)amino)acetate[4]

90% yield, **^{1}H NMR** (500 MHz, CD_2Cl_2) δ 8.25 (t, *J* = 1.4 Hz, 1H), 7.72 (d, *J* = 8.5 Hz, 2H), 7.42 (d, *J* = 8.5 Hz, 2H), 4.38 (d, *J* = 1.4 Hz, 2H), 3.74 (s, 3H);

^{13}C NMR (126 MHz, CD_2Cl_2) δ 170.87, 164.29, 137.55, 134.85, 130.11, 129.45, 62.21, 52.49.

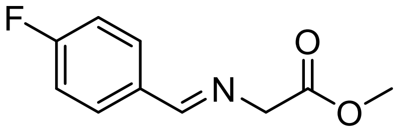

2c

Methyl (*E*)-2-((4-fluorobenzylidene)amino)acetate[5]

87% yield, **^{1}H NMR** (700 MHz, CD_2Cl_2) δ 8.25 (t, *J* = 1.5 Hz, 1H), 7.78 (d, *J* = 8.7, Hz, 2H), 7.13 (d, *J* = 8.7 Hz, 2H), 4.37 (d, *J* = 1.5 Hz, 2H), 3.74 (s, 3H);

^{13}C NMR (176 MHz, CD_2Cl_2) δ 170.99, 165.87 (d, *J* = 250.7 Hz), 164.44, 164.18, 132.78 (d, *J* = 3.0 Hz), 130.92 (d, *J* = 8.8 Hz), 116.19 (d, *J* = 22.0 Hz), 62.21, 52.46.

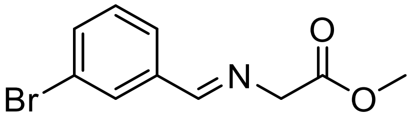

2d

Methyl (*E*)-2-((3-bromobenzylidene)amino)acetate[5]

82% yield, ^{1}**H NMR** (700 MHz, CD_2Cl_2) δ 8.23 (t, *J* = 1.4 Hz ,1H), 7.96 (s, 1H), 7.68-7.63 (m, 1H), 7.61 – 7.59 (m, 1H), 7.33 (t, *J* = 7.8 Hz, 1H), 4.39 (d, *J* = 1.4 Hz, 2H), 3.75 (s, 3H);

^{13}**C NMR** (176 MHz, CD_2Cl_2) δ 170.77, 164.06, 138.36, 134.55, 131.38, 130.83, 127.80, 123.37, 62.20, 52.52.

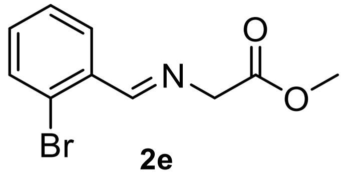

2e

Methyl (*E*)-2-((2-bromobenzylidene)amino)acetate

80% yield, ^{1}**H NM**R (700 MHz, CD_2Cl_2) δ 8.66 (d, *J* = 1.5 Hz, 1H), 8.05 (dd, *J* = 8.0, 1.8 Hz, 1H), 7.60 (dd, *J* = 8.0, 1.8 Hz, 1H), 7.40 – 7.37 (m, 1H), 7.32-7.28 (m, 1H), 4.44 (d, *J* = 1.5 Hz, 2H), 3.75 (s, 3H);

^{13}**C NMR** (176 MHz, CD_2Cl_2) δ 170.79, 164.54, 134.70, 133.66, 132.93, 129.36, 128.28, 125.83, 62.29, 52.51.

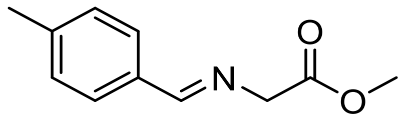

2f

Methyl (*E*)-2-((4-methylbenzylidene)amino)acetate[4]

86% yield, ^{1}**H NMR** (700 MHz, CD_2Cl_2) δ 8.24 (t, *J* = 1.4 Hz, 1H), 7.65 (d, *J* = 8.2 Hz, 2H), 7.24 (d, *J* = 8.2 Hz, 2H), 4.36 (d, *J* = 1.4 Hz, 2H), 3.74 (s, 3H), 2.39 (s, 3H);

^{13}**C NMR** (176 MHz, CD_2Cl_2) δ 171.18, 165.52, 142.30, 133.76, 129.92, 128.83, 62.39, 52.42, 21.81.

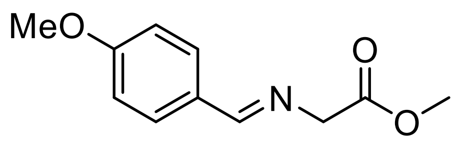

2g

Methyl (*E*)-2-((4-methoxybenzylidene)amino)acetate[5]

78% yield, **^{1}H NMR** (700 MHz, CD_2Cl_2) δ 8.24 (d, *J* = 1.4 Hz, 1H), 7.74 (d, *J* = 8.8 Hz, 2H), 6.98 (d, *J* = 8.8 Hz, 2H), 4.38 (d, *J* = 1.4 Hz, 2H), 3.88 (s, 3H), 3.78 (s, 3H);

^{13}C NMR (176 MHz, CD_2Cl_2) δ 171.30, 164.85, 162.71, 130.48, 129.27, 114.56, 62.33, 55.93, 52.39.

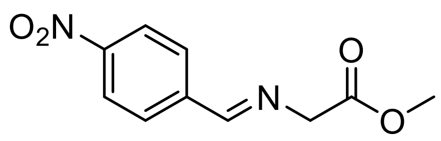

2h

Methyl (*E*)-2-((4-nitrobenzylidene)amino)acetate

77% yield, **^{1}H NMR** (700 MHz, CD_2Cl_2) δ 8.39 (t, *J* = 1.4 Hz, 1H), 8.27 (d, *J* = 8.8 Hz, 2H), 7.96 (d, *J* = 8.8 Hz, 2H), 4.46 (d, *J* = 1.4 Hz, 2H), 3.76 (s, 3H);

^{13}C NMR (176 MHz, CD_2Cl_2) δ 170.46, 163.56, 141.70, 129.66, 124.41, 62.27, 52.60.

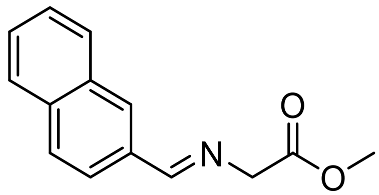

2i

Methyl (*E*)-2-((naphthalen-2-ylmethylene)amino)acetate

75% yield, ^{1}**H NMR** (700 MHz, CD_2Cl_2) δ 8.44 (t, *J* = 1.4 Hz, 1H), 8.10 – 8.09 (m, 1H), 8.02 (dd, *J* = 8.5, 1.7 Hz, 1H), 7.94 – 7.92 (m, 1H), 7.89-7.84 (m, 2H), 7.59 – 7.53 (m, 2H), 4.45 (d, *J* = 1.4 Hz, 2H), 3.77 (s, 3H);

^{13}**C NMR** (176 MHz, CD_2Cl_2) δ 171.10, 165.66, 135.50, 134.06, 133.61, 131.11, 129.22, 129.07, 128.39, 128.00, 127.15, 124.15, 62.45, 52.48.

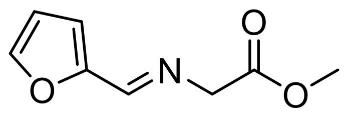

2j

Methyl (*E*)-2-((furan-2-ylmethylene)amino)acetate[6]

80% yield, ^{1}**H NMR** (400 MHz, CD_2Cl_2): δ 8.07 (t, *J* = 1.3 Hz, 1H), 7.56 (d, *J* = 5.8 Hz, 1H), 6.85 (d, *J* = 5.5 Hz, 1H), 6.52 (dd, *J* = 5.8, 5.5 Hz, 1H), 4.33 (d, *J* = 1.3 Hz, 2H), 3.74 (s, 3H);

^{13}**C NMR** (101 MHz, (CD2Cl2): δ 170.87, 153.79, 151.98, 145.83, 115.54, 112.31, 62.23, 52.48.

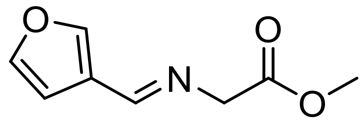

2k

Methyl (*E*)-2-((furan-3-ylmethylene)amino)acetate[6]

76% yield, ^{1}**H NMR** (700 MHz, CD_2Cl_2) δ 8.21 (s, *J* = 1.4 Hz, 1H), 7.78 (d, *J* = 4.1 Hz, 1H), 7.49 – 7.44 (m, 1H), 6.84 – 6.79 (m, 1H), 4.30 (d, *J* = 1.4 Hz, 2H), 3.73 (s, 3H);

^{13}**C NMR** (176 MHz, CD_2Cl_2) δ 171.07, 157.31, 146.58, 144.85, 125.87, 108.23, 62.42, 52.42.

General procedures for 1,3-dipolar cycloadditions

Procedure A for the synthesis of the double cycloaddition product

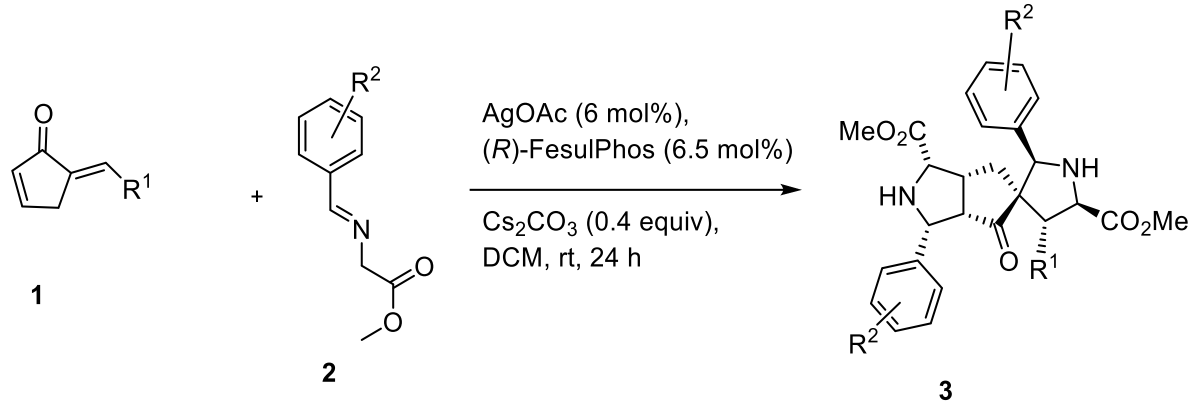

1 **2** **3**

(*Rp*)-2-(*tert*-Butylthio)-1-(diphenylphosphino)ferrocene (6.5 mol%, 7.8 μmol) and silver acetate (6 mol%, 7.2 μmol) were dissolved in DCM stirred at ambient temperature for 15 min. To the resulting solution iminoester **2** (2.2 equiv., 0.26 mmol), Cs_2CO_3 (40 mol%, 48 μmol) and enone **1** (1 equiv., 0.12 mmol) were added and the mixture was allowed to stired at ambient temperature for at least 24 h (until all *endo, exo* diastereomer is converted to *endo, endo* isomer). The crude mixture was directly charged onto silica gel and the product was isolated using *n*-pentane / acetone as eluent.

Procedure B for the synthesis of double addition products with two different imines

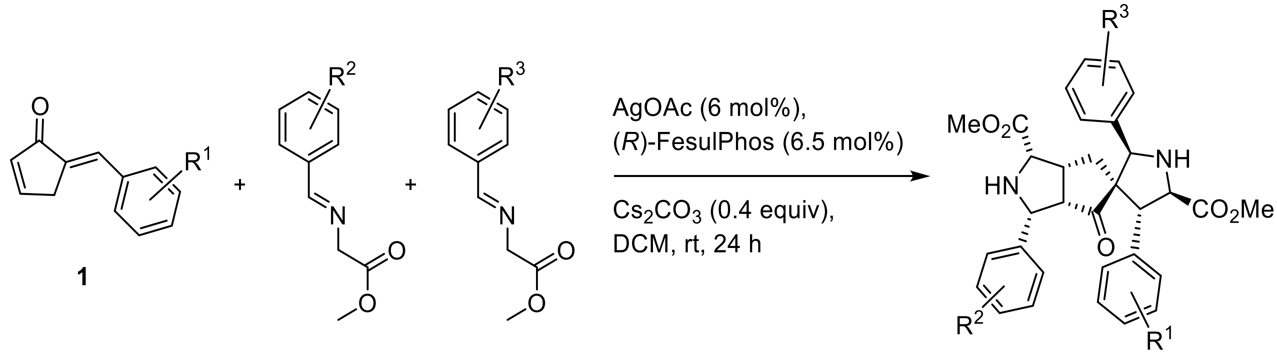

1 **2** **2'** **5**

(Rp)-2-(tert-Butylthio)-1-(diphenylphosphino)ferrocene (6.5 mol%, 7.8 µmol) and silver acetate (6 mol%, 7.2 µmol) were dissolved in DCM stirred at ambient temperature for 15 min. To the resulting solution iminoester 2 (1.1 equiv., 0.13 mmol), Cs_2CO_3 (40 mol%, 48 µmol) and enone 1 (1 equiv., 0.12 mmol) were added and the mixture was allowed to stir at ambient temperature for 3 h followed by addition of second iminoester 2 (1.1 equiv., 0.13 mmol). The reaction mixture was stirred at ambient temperature for at least 24 h. The crude mixture was directly charged onto silica gel and the product was isolated using n-pentane / acetone as eluent.

Procedure C for the synthesis of mono addition products

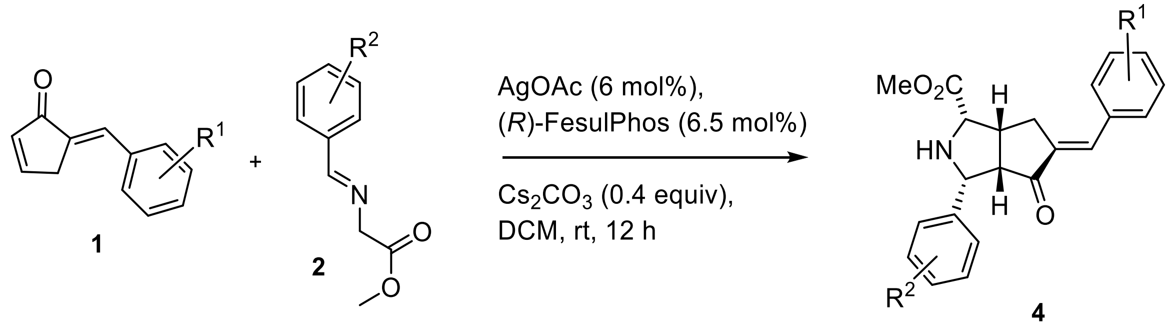

(*Rp*)-2-(*tert*-Butylthio)-1-(diphenylphosphino)ferrocene (6.5 mol%, 7.8 µmol) and silver acetate (6 mol%, 7.2 µmol) were dissolved in DCM stirred at ambient temperature for 15 min. To the resulting solution were added iminoester **2** (1.1 equiv., 0.13 mmol), Cs_2CO_3 (40 mol%, 48 µmol) and enone **1** (1.00 equiv., 0.12 mmol) were added and the mixture was allowed to stir at ambient temperature for 12 h. The crude mixture was directly charged onto silica gel and the product was isolated using *n*-pentane / acetone as eluent.

Procedure D for the synthesis of the double cycloaddition product (*endo, exo*) in THF:H_2O

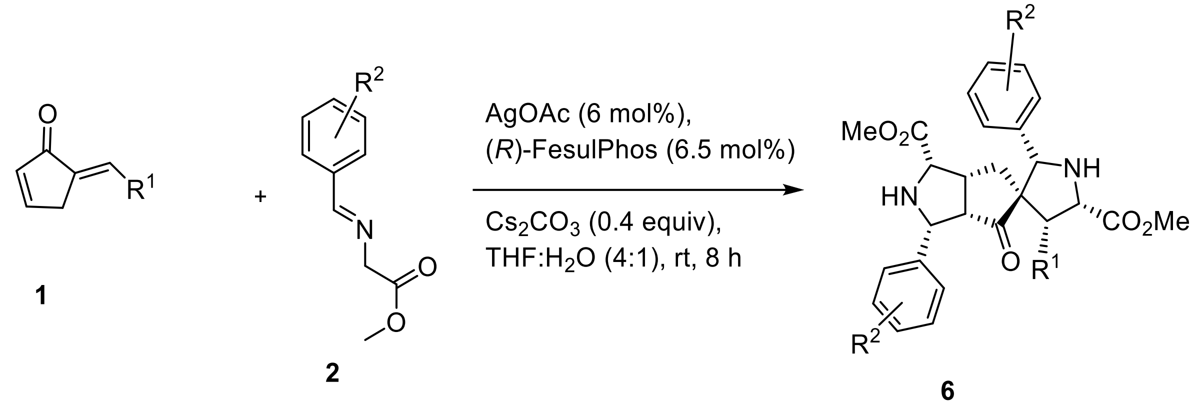

(Rp)-2-(tert-Butylthio)-1-(diphenylphosphino)ferrocene (6.5 mol%, 7.8 μmol) and silver acetate (6 mol%, 7.2 μmol) were dissolved in THF:H_2O (4:1) (2.5 mL total volume) stirred at ambient temperature for 15 min. To the resulting solution iminoester 2 (2.2 equiv., 0.26 mmol), Cs_2CO_3 (40 mol%, 48 μmol) and enone 1 (1 equiv., 0.12 mmol) were added and the mixture was allowed to stir at ambient temperature for 8 h. The crude mixture was directly charged onto silica gel and the product was isolated using *n*-pentane / acetone as eluent.

Characterization of cycloaddition products 3-6.

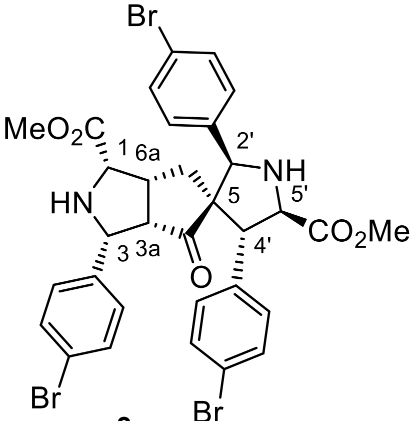

3a

Dimethyl (1*S*,2'*R*,3*R*,3a*S*,4'*R*,5*R*,5'*R*,6a*R*)-2',3,4'-tris(4-bromophenyl)-4-oxohexahydro-1*H*-spiro[cyclopenta[*c*]pyrrole-5,3'-pyrrolidine]-1,5'-dicarboxylate

yellow amorphous solid, 81% yield, **^{1}H NMR** (700 MHz, CD_2Cl_2) δ 7.53 (d, *J* = 7.5 Hz, 2H), 7.47 (d, *J* = 7.5 Hz, 2H), 7.42 (d, *J* = 8.0 Hz, 2H), 7.15 (d, *J* = 8.0 Hz, 2H), 7.05 (d, *J* = 7.9 Hz, 2H), 6.97 (d, *J* = 7.9 Hz, 2H), 4.25 (s, 1H), 4.14 (d, *J* = 10.7 Hz, 1H), 3.97 (d, *J* = 7.1 Hz, 1H), 3.75 (d, *J* = 7.6 Hz, 1H), 3.66 (s, 3H), 3.60 (s, 3H), 3.43 (d, *J* = 7.1 Hz, 1H), 2.63 – 2.59 (m, 1H), 2.03-1.99 (m, 1H), 1.75 (dd, *J* = 13.6, 7.6 Hz, 1H), 1.59 (dd, *J* = 13.6, 7.6 Hz, 1H); **^{13}C NMR** (176 MHz, CD_2Cl_2) δ 216.65, 173.27, 171.55, 138.97, 138.88, 137.94, 132.34, 132.20, 131.47, 130.20, 130.05, 122.86, 121.72, 121.52, 74.72, 68.28, 66.97, 63.99, 63.12, 55.87, 55.32, 52.66, 52.26, 40.28, 34.47;

HRMS: calcd. for $[M+H]^+$ $C_{32}H_{29}{}^{79}Br_3N_2O_5$ = 758.97127, found: 758.96994; calcd. for $[M+H]^+$ $C_{32}H_{29}{}^{79}Br^{81}Br_2N_2O_5$ = 762.96703, found: 762.96584; calcd. for $[M+H]^+$ $C_{32}H_{29}{}^{79}Br_2{}^{81}BrN_2O_5$ = 760.96902, found: 760.96789; calcd. for $[M+H]^+$ $C_{32}H_{29}{}^{81}Br_3N_2O_5$ = 764.96554, found: 764.96380;

HPLC conditions: CHIRALPAK IC column, *iso*-propanol/ *iso*-hexane = 30/70, flow rate = 0.5 mL min^{-1}, minor enantiomer: t_R = 19.79 min; major enantiomer: t_R = 26.32 min; (99% e.e.); CHIRALPAK IA column, *iso*-propanol/ *iso*-hexane = 15/85, flow rate = 0.5 mL min^{-1}, minor enantiomer: t_R = 86.05 min; major enantiomer: t_R = 51.16 min; (99% e.e.);

$[\alpha]^{20}_{D}$= + 41.1° (c = 0.19, $CHCl_3$).

FT-IR: $\tilde{\nu}$ 3431, 2884, 2765, 2521, 2315, 1644, 1615, 1461, 1092, 1156, 1075 cm^{-1}.

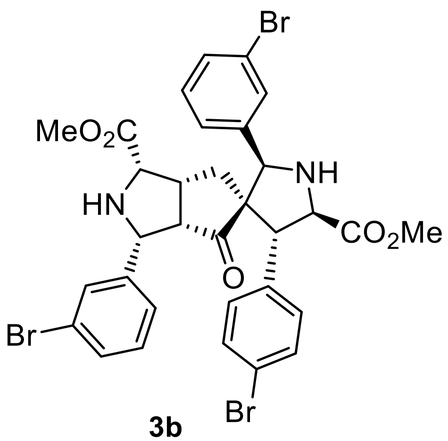

3b

Dimethyl*(1S,2'R,3R,3aS,4'R,5R,5'R,6aR)*-2',3-bis(3-bromophenyl)-4'-(4-bromophenyl)-4-oxohexahydro-1*H*-spiro[cyclopenta[*c*]pyrrole-5,3'-pyrrolidine]-1,5'-dicarboxylate

white amorphous solid, 72% yield, ^{1}**H NMR** (400 MHz, (CD_2Cl_2): δ 7.55 (d, *J* = 8.4 Hz, 2H), 7.46 (d, *J* = 8.4 Hz, 1H), 7.43-7.42 (m, 1H), 7.41 – 7.40 (m, 1H), 7.32 (d, *J* = 7.5 Hz, 1H), 7.24 – 7.17 (m, 3H), 7.11 (d, *J* = 7.5 Hz, 2H), 7.01 (d, *J* = 7.5 Hz, 1H), 4.23 (s, 1H), 4.14 (d, *J* = 10.7 Hz, 1H), 3.99 (d, *J* = 7.1 Hz, 1H), 3.75 (d, *J* = 8.9 Hz, 1H), 3.66 (s, 3H), 3.60 (s, 3H), 3.48 (d, *J* = 7.1 Hz, 1H), 2.63 – 2.58 (m, 1H), 2.05 (dd, *J* = 13.7, 8.9 Hz, 1H), 1.59 (dd, *J* = 13.7, 10.6 Hz, 1H), 1.28 – 1.24 (m, 1H); ^{13}**C NMR** (101 MHz, (CD_2Cl_2): δ 216.59, 173.20, 171.45, 142.31, 141.24, 139.12, 132.30, 132.12, 131.49, 131.45, 131.04, 130.92, 130.87, 130.10, 127.47, 127.15, 123.11, 122.61, 121.69, 74.78,, 68.28, 66.99, 64.04, 63.17, 55.97, 55.38, 52.61, 52.26, 40.16, 34.53;

HRMS: calcd. for $[M+H]^+$ $C_{32}H_{29}{}^{79}Br_3N_2O_5$ = 758.97121, found: 758.96994; calcd. for $[M+H]^+$ $C_{32}H_{29}{}^{79}Br^{81}Br_2N_2O_5$ = 762.96571, found: 762.96584; calcd. for $[M+H]^+$ $C_{32}H_{29}{}^{79}Br_2{}^{81}BrN_2O_5$ = 760.96842, found: 760.96789; calcd. for $[M+H]^+$ $C_{32}H_{29}{}^{81}Br_3N_2O_5$ = 764.96422, found: 764.96380;

HPLC conditions: CHIRALPAK IC column, *iso*-propanol/ *iso*-hexane = 30/70, flow rate = 0.5 mL min^{-1}, minor enantiomer: t_R = 22.14 min; major enantiomer: t_R = 86.39 min; (91% e.e.);

$[\alpha]^{20}_D$= + 136.2° (c = 0.2, $CHCl_3$).

FT-IR: $\tilde{\nu}$ 3457**,** 2952, 2361, 1732, 1489, 1434, 1362, 1206, 1159, 1073, 1023cm^{-1}.

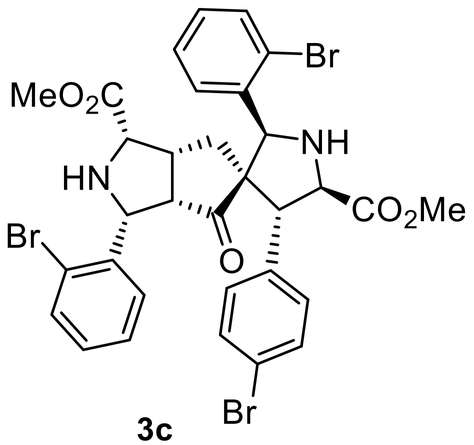

3c

Dimethyl (1*S*,2'*S*,3*R*,3a*S*,4'*R*,5*R*,5'*R*,6a*R*)-2',3-bis(2-bromophenyl)-4'-(4-bromophenyl)-4-oxohexahydro-1*H*-spiro[cyclopenta[*c*]pyrrole-5,3'-pyrrolidine]-1,5'-dicarboxylate

white amorphous solid, 65% yield, **^{1}H NMR** (400 MHz, (CD_2Cl_2): δ 7.61 (dd, *J* = 8.5, 1.3 Hz, 1H), 7.54 – 7.49 (m, 3H), 7.35 – 7.30 (m, 1H), 7.29 – 7.23 (m, 2H), 7.21 – 7.12 (m, 3H), 7.02 (d, *J* = 8.5 Hz, 2H), 4.95 (s, 1H), 4.38 (d, *J* = 10.6 Hz, 1H), 3.99 (d, *J* = 7.7 Hz, 1H), 3.77 (d, *J* = 5.8 Hz, 1H), 3.65 (s, 3H), 3.57 (s, 3H), 3.44 (d, *J* = 7.7 Hz, 1H), 2.87 – 2.81 (m, 1H), 2.68 (bs, 1H), 2.35 – 2.27 (m, 1H), 2.05 – 1.92 (m, 2H), 1.44 (dd, *J* = 13.4, 10.6 Hz, 1H); **^{13}C NMR** (101 MHz, (CD_2Cl_2): δ 216.17, 173.19, 171.61, 139.28, 139.10, 137.85, 133.70, 132.52, 132.13, 131.45, 130.21, 129.99, 129.14, 128.58, 128.53, 127.70, 125.02, 124.92, 121.53, 71.13, 68.87, 66.67, 63.46, 62.89, 55.22, 52.93, 52.58, 52.16, 39.76, 33.79,

HRMS: calcd. for $[M+H]^+$ $C_{32}H_{29}{}^{79}Br_3N_2O_5$ = 758.97121, found: 758.96994; calcd. for $[M+H]^+$ $C_{32}H_{29}{}^{79}Br^{81}Br_2N_2O_5$ = 762.96571, found: 762.96584; calcd. for $[M+H]^+$ $C_{32}H_{29}{}^{79}Br_2{}^{81}BrN_2O_5$ = 760.96842, found: 760.96789; calcd. for $[M+H]^+$ $C_{32}H_{29}{}^{81}Br_3N_2O_5$ = 764.96422, found: 764.96380;

HPLC conditions: CHIRALPAK IC column, *iso*-propanol/ *iso*-hexane = 40/60, flow rate = 0.5 mL min^{-1}, minor enantiomer: t_R = 23.47 min; major enantiomer: t_R = 28.45 min**;** (98% e.e.);

[α]$^{20}_{D}$= + 136.1° (c = 0.3, $CHCl_3$).

FT-IR: $\tilde{\nu}$ 3447, 2877, 2758, 2359, 1771, 1733,1653, 1206, 1181, 1129, 1119 cm^{-1}.

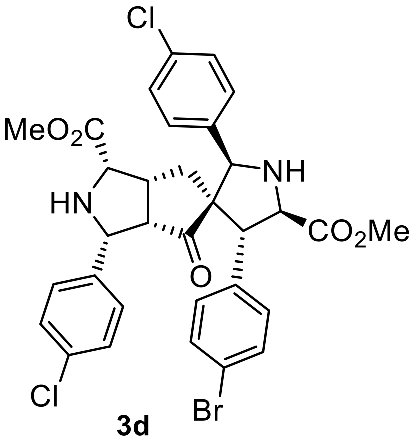

3d

Dimethyl (1S,2'*R*,3*R*,3*aS*,4'*R*,5*R*,5'*R*,6*aR*)-4'-(4-bromophenyl)-2',3-bis(4-chlorophenyl)-4-oxohexahydro-1*H*-spiro[cyclopenta[*c*]pyrrole-5,3'-pyrrolidine]-1,5'-dicarboxylate

white amorphous solid, 81% yield, **^{1}H NMR** (400 MHz, (CD_2Cl_2): δ 7.53 (d, *J* = 8.4 Hz, 2H), 7.31 (d, *J* = 8.5 Hz, 2H), 7.26 (d, *J* = 8.5 Hz, 2H), 7.21 (d, *J* = 8.4 Hz, 2H), 7.06 (d, *J* = 8.4 Hz, 2H), 7.02 (d, *J* = 8.4 Hz, 2H), 4.26 (s, 1H), 4.15 (d, *J* = 10.8 Hz, 1H), 3.97 (d, *J* = 7.2 Hz, 1H), 3.75 (d, *J* = 8.2 Hz, 1H), 3.66 (s, 3H), 3.59 (s, 3H), 3.44 (d, *J* = 7.2 Hz, 1H), 2.64 – 2.58 (m, 1H), 2.05 – 2.00 (m, 1H), 1.78-1.73 (m, 1H), 1.59 (dd, *J* = 13.5, 8.2 Hz, 1H); **^{13}C NMR** (101 MHz, (CD_2Cl_2): δ 216.67, 173.27, 171.55, 139.00, 138.37, 137.45, 134.66, 133.38, 132.18, 131.46, 129.88, 129.70, 129.36, 128.49, 121.70, 74.70, 68.31, 66.99, 63.95, 63.11, 55.90, 55.34, 52.60, 52.24, 40.28, 34.38;

HRMS: calcd. for $[M+H]^+$ $C_{32}H_{29}{}^{79}Br^{35}Cl_2N_2O_5$ = 671.07186, found: 671.07097; calcd. for $[M+H]^+$ $C_{32}H_{29}{}^{81}Br^{35}Cl_2N_2O_5$ = 673.06864, found: 673.06892; calcd. for $[M+H]^+$ $C_{32}H_{29}{}^{81}Br^{35}Cl^{37}ClN_2O_5$ = 675.06609, found: 675.06597;

HPLC conditions: CHIRALPAK IC column, *iso*-propanol/ *iso*-hexane = 30/70, flow rate = 0.5 mL min^{-1}, minor enantiomer: t_R = 18.25 min; major enantiomer: t_R = 25.29 min; (99% e.e.);

$[\alpha]^{20}_D$= + 131.1° (c = 0.2, $CHCl_3$).

FT-IR: $\tilde{\nu}$ 3651, 2525, 2159, 2029, 1976, 1788, 1589, 1425, 1236, 1123, 1023 cm^{-1}.

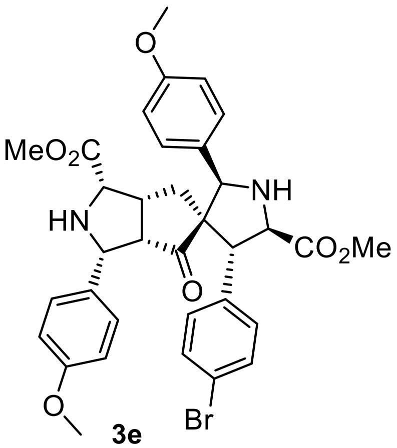

3e

Dimethyl (1*S*,2'*R*,3*R*,3a*S*,4'*R*,5*R*,5'*R*,6a*R*)-4'-(4-bromophenyl)-2',3-bis(4-methoxyphenyl)-4-oxohexahydro-1*H*-spiro[cyclopenta[*c*]pyrrole-5,3'-pyrrolidine]-1,5'-dicarboxylate

white amorphous solid, 60% yield, **^{1}H NMR** (400 MHz, CD_2Cl_2): δ 7.54 (d, *J* = 8.4 Hz, 2H), 7.16 (d, *J* = 8.6 Hz, 2H), 7.09 (d, *J* = 8.4 Hz, 2H), 6.96 (d, *J* = 8.6 Hz, 2H), 6.86 (d, *J* = 17.4, 8.6 Hz, 2H), 6.84 (d, *J* = 8.6 Hz, 2H) 4.20 (s, 1H), 4.10 (d, *J* = 10.5 Hz, 1H), 3.93 (d, *J* = 7.3 Hz, 1H), 3.82 (s, 3H), 3.80 (s, 3H), 3.76 (d, *J* = 7.3 Hz, 1H), 3.70 (d, *J* = 5.8 Hz, 1H), 3.64 (s, 3H), 3.59 (s, 3H), 3.44 (d, *J* = 7.3 Hz, 1H), 2.61 – 2.55 (m, 1H), 2.13 (d, *J* = 5.8 Hz, 1H), 1.96 (m, 1H), 1.75 -1.70 (m, 1H), 1.56 (dd, *J* = 13.5, 10.5 Hz, 1H); **^{13}C NMR** (101 MHz, (CD_2Cl_2): δ 217.03, 172.75, 171.27, 159.67, 158.98, 139.10, 131.95, 131.56, 131.04, 130.92, 128.95, 128.80, 120.96, 113.91, 113.12, 74.89, 67.76, 66.88, 63.94, 62.59, 55.64, 55.37, 55.20, 55.14, 51.93, 51.59, 40.15, 34.04;

HRMS: calcd. for $[M+H]^+$ $C_{34}H_{35}{}^{79}BrN_2O_7$ = 663.16997, found: 663.17004; calcd. for $[M+H]^+$ $C_{34}H_{35}{}^{81}BrN_2O_7$ = 665.16801, found: 665.16799;

HPLC conditions: CHIRALPAK IA column, *iso*-propanol/ *iso*-hexane = 60/40, flow rate = 0.5 mL min^{-1}, minor enantiomer: t_R = 31.99 min; major enantiomer: t_R = 23.59 min; (98% e.e.);

$[\alpha]^{20}_D$= + 157.6° (c = 0.2, $CHCl_3$).

FT-IR: $\tilde{\nu}$ 3475, 2439, 2379, 2342, 1640, 1544, 1303, 1288 ,1278, 1246, 1033 cm^{-1}.

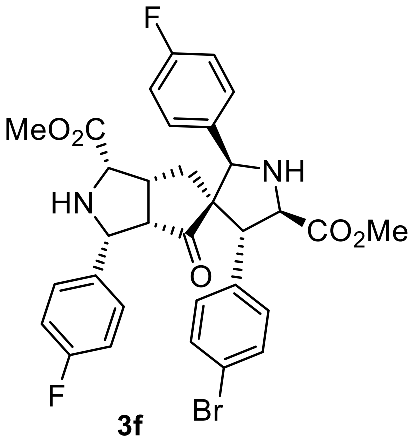

3f

Dimethyl (1*S*,2'*R*,3*R*,3a*S*,4'*R*,5*R*,5'*R*,6a*R*)-4'-(4-bromophenyl)-2',3-bis(4-fluorophenyl)-4-oxohexahydro-1*H*-spiro[cyclopenta[*c*]pyrrole-5,3'-pyrrolidine]-1,5'-dicarboxylate

white amorphous solid, 75% yield, **^{1}H NMR** (400 MHz, (CD_2Cl_2): δ 7.53 (d, *J* = 8.0 Hz, 2H), 7.25 (d, *J* = 8.0, 2H), 7.08 – 7.02 (m, 6H), 6.99-6.96 (m, 2H), 4.27 (s, 1H), 4.15 (d, *J* = 10.6 Hz, 1H), 3.97 (d, *J* = 7.3 Hz, 1H), 3.74 (d, *J* = 7.6 Hz, 1H), 3.66 (s, 3H), 3.59 (s, 3H), 3.44 (d, *J* = 7.3 Hz, 1H), 2.63 - 2.59 (m, 1H), 2.00 -1.97 (m, 1H), 1.77 (dd, *J* = 13.5, 7.6 Hz, 1H), 1.60 (dd, *J* = 13.5, 10.6 Hz, 1H); **^{13}C NMR** (101 MHz, (CD_2Cl_2): δ 216.94, 173.27, 171.63, 163.92 (d, *J* = 246.8 Hz), 162.52 (d, *J* = 246.4 Hz), 139.10, 135.46 (d, *J* = 3 Hz), 134.58 (d, *J* = 3.1 Hz), 132.18, 131.49, 130.20 (d, *J* = 8.1 Hz), 129.88 (d, *J* = 8 Hz), 121.69, 116.15 (d, *J* = 21.5 Hz), 115.20 (d, *J* = 21.4 Hz), 74.76, 68.26, 67.04, 64.00, 63.11, 55.90, 55.46, 52.58, 52.24, 40.36, 34.55; **^{19}F NMR** (565 MHz, CD_2Cl_2) δ -113.6 (m), -115.4 (m);

HRMS: calcd. for $[M+H]^+$ $C_{32}H_{29}{}^{79}BrF_2N_2O_5$ = 639.12962, found: 639.13007; calcd. for $[M+H]^+$ $C_{32}H_{29}{}^{81}BrF_2N_2O_5$ = 641.12732, found: 641.12802;

HPLC conditions: CHIRALPAK IC column, *iso*-propanol/ *iso*-hexane = 30/70, flow rate = 0.5 mL min^{-1}, minor enantiomer: t_R = 18.43 min; major enantiomer: t_R = 27.01 min**;** (98% e.e.);

$[\alpha]^{20}_{D}$= + 144.1° (c = 0.1, $CHCl_3$).

FT-IR: $\tilde{\nu}$ 3458, 2922, 2385, 1732, 1604, 1508, 1218, 1145, 1089, 1010 cm^{-1}.

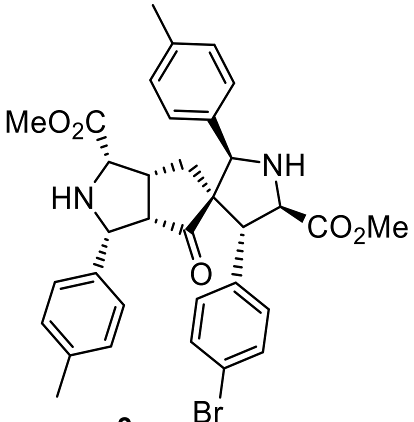

3g

Dimethyl (1*S*,2'*R*,3*R*,3a*S*,4'*R*,5*R*,5'*R*,6a*R*)-4'-(4-bromophenyl)-4-oxo-2',3-di-*p*-tolylhexahydro-1*H*-spiro[cyclopenta[*c*]pyrrole-5,3'-pyrrolidine]-1,5'-dicarboxylate

white amorphous solid, 63% yield, **^{1}H NMR** (400 MHz, (CD_2Cl_2): δ 7.46 (d, *J* = 8.4 Hz, 2H), 7.06 – 6.99 (m, 8H), 6.83 (d, *J* = 8.0 Hz, 2H), 4.12 (s, 1H), 4.01 (d, *J* = 10.8 Hz, 1H), 3.86 (d, *J* = 7.4 Hz, 1H), 3.61 (d, *J* = 5.9 Hz, 1H), 3.56 (s, 3H), 3.50 (s, 3H), 3.34 (d, *J* = 7.4 Hz, 1H), 2.54 – 2.46 (m, 1H), 2.28 (s, 3H), 2.25 (s, 3H), 1.91 – 1.86 (m, 1H), 1.67 – 1.63 (m, 1H), 1.48 (dd, *J* = 13.4, 10.8 Hz, 1H);

^{13}C NMR (101 MHz, (CD_2Cl_2): δ 217.41, 173.30, 171.83, 139.66, 138.91, 137.68, 136.60, 135.17, 132.14, 131.52, 129.85, 129.09, 128.27, 128.25, 121.54, 75.81, 68.55, 67.56, 64.87, 63.24, 56.36, 56.06, 52.50, 52.18, 40.79, 34.55, 29.65, 21.41;

HRMS: calcd. for $[M+H]^+$ $C_{34}H_{35}{}^{79}BrN_2O_5$ = 631.18013, found: 631.18021; calcd. for $[M+H]^+$ $C_{34}H_{35}{}^{81}BrN_2O_5$ = 633.17791, found: 633.17816;

HPLC conditions: CHIRALPAK IC column, *iso*-propanol/ *iso*-hexane = 80/20, flow rate = 0.5 mL min^{-1}, minor enantiomer: t_R = 23.13 min; major enantiomer: t_R = 66.36 min; (93% e.e.);

$[\alpha]^{20}_D$= + 95.9° (c = 0.22, $CHCl_3$).

FT-IR: $\tilde{\nu}$ 3384, 2966, 2867, 2161, 2032, 1735, 1587, 1436, 1206, 1106, 1011 cm^{-1}.

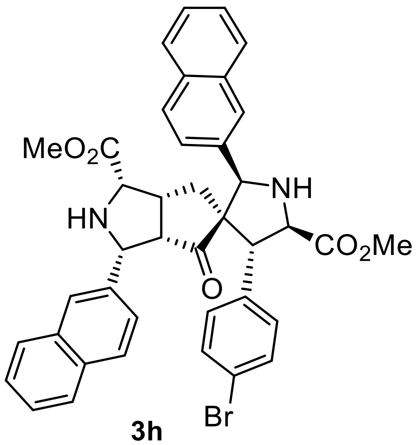

3h

Dimethyl (1*S*,2'*R*,3*R*,3a*S*,4'*R*,5*R*,5'*R*,6a*R*)-4'-(4-bromophenyl)-2',3-di(naphthalen-2-yl)-4-oxohexahydro-1*H*-spiro[cyclopenta[*c*]pyrrole-5,3'-pyrrolidine]-1,5'-dicarboxylate

white amorphous solid, 66% yield, **^{1}H NMR** (400 MHz, CD_2Cl_2): δ 7.87 – 7.85 (m, 3H), 7.83 (d, *J* = 8.5 Hz, 1H), 7.80 (d, *J* = 8.0 Hz, 1H), 7.77 (d, *J* = 8.5 Hz, 1H), 7.74 (s, 1H), 7.60 (s, 1H), 7.57 (d, *J* = 8.0 Hz, 2H), 7.53 – 7.47 (m, 4H), 7.35 (dd, *J* = 8.4, 1.8 Hz, 1H), 7.12 – 7.10 (m, 3H), 4.44 (s, 1H), 4.25 (d, *J* = 10.6 Hz, 1H), 3.98 (d, *J* = 7.0 Hz, 1H), 3.72 (d, *J* = 5.9 Hz, 1H), 3.69 (s, 3H), 3.44 (d, *J* = 7.0 Hz, 1H), 3.36 (s, 3H), 2.66 – 2.59 (m, 1H), 2.02 (dd, *J* = 13.5, 8.7 Hz, 1H), 1.90 -1.84 (m, 1H), 1.73 (dd, *J* = 13.5, 10.6 Hz, 1H); **^{13}C NMR** (176 MHz, (CD_2Cl_2): δ 216.47, 172.67, 171.21, 138.97, 136.91, 135.41, 133.30, 133.13, 133.01, 131.65, 130.93, 128.41, 128.05, 127.73, 127.66, 127.62, 127.13, 127.03, 126.49, 126.39, 126.01, 125.72, 125.63, 125.53, 121.05, 75.25, 67.97, 66.81, 64.21, 62.70, 55.68, 55.29, 51.74, 51.67, 40.07, 34.06;

HRMS: calcd. for $[M+H]^+$ $C_{40}H_{35}{}^{79}BrN_2O_5$ = 703.18101, found: 703.18021; calcd. for $[M+H]^+$ $C_{40}H_{35}{}^{81}BrN_2O_5$ = 705.17876, found: 705.17816;

HPLC conditions: CHIRALPAK IA column, *iso*-propanol/ *iso*-hexane = 60/40, flow rate = 0.5 mL min^{-1}, minor enantiomer: t_R = 70.97 min; major enantiomer: t_R = 42.85 min; (96% e.e.);

$[\alpha]^{20}{}_D$= + 168.8° (c = 0.22, $CHCl_3$).

FT-IR: $\tilde{\nu}$ 3364, 2956, 2867, 2159, 2031, 1731, 1489, 1208, 1088, 1012 cm^{-1}.

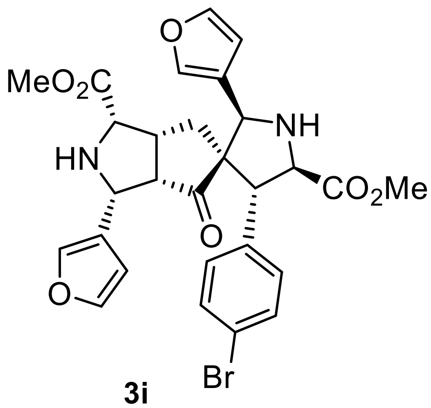

3i

Dimethyl (1*S*,2'*S*,3*R*,3a*S*,4'*R*,5*R*,5'*R*,6a*R*)-4'-(4-bromophenyl)-2',3-di(furan-2-yl)-4-oxohexahydro-1*H*-spiro[cyclopenta[*c*]pyrrole-5,3'-pyrrolidine]-1,5'-dicarboxylate

white amorphous solid, 40% yield **^{1}H NMR** (400 MHz, CD_2Cl_2): δ 7.51 – 7.46 (m, 3H), 7.40 (m, 1H), 7.23 – 7.22 (m, 2H), 6.99 (d, *J* = 8.5 Hz, 1H), 6.30 (dd, *J* = 1.9, 0.9 Hz, 2H), 6.11 (dd, *J* = 1.9, 0.9 Hz, 1H), 4.24 (d, *J* = 10.7 Hz, 1H), 4.20 (s, 1H), 3.90 (d, *J* = 7.1 Hz, 1H), 3.76 (d, *J* = 5.7 Hz, 1H), 3.64 (s, 3H), 3.63 (s, 3H), 3.34 (d, *J* = 7.1 Hz, 1H), 2.76 - 2.72 (m, 1H), 2.44 -2.39 (m, 1H), 1.65 -1.61 (m, 1H), 1.49 (dd, *J* = 13.6, 10.7 Hz, 1H);

^{13}C NMR (126 MHz, CD_2Cl_2) δ 218.09, 173.09, 171.76, 144.31, 143.32, 141.46, 140.34, 139.57, 132.15, 131.33, 125.31, 123.13, 121.54, 110.98, 109.74, 67.87, 67.56, 67.25, 63.23, 56.80, 56.30, 56.15, 52.61, 52.23, 41.37, 34.16;

HRMS: calcd. for $[M+H]^+$ $C_{28}H_{27}{}^{79}BrN_2O_7$ = 583.10702, found: 583.10744; calcd. for $[M+H]^+$ $C_{28}H_{27}{}^{81}BrN_2O_7$ = 585.10472, found: 585.10539;

HPLC conditions: CHIRALPAK IC column, *iso*-propanol/ *iso*-hexane = 50/50, flow rate = 0.5 mL min^{-1}, minor enantiomer: t_R = 31.04 min; major enantiomer: t_R = 67.54 min; (96% e.e.);

$[\alpha]^{20}_{D}$= + 78.5° (c = 0.12, $CHCl_3$).

FT-IR: $\tilde{\nu}$ 3458, 2932, 2885, 2159, 2029,1765, 1532, 1302, 1056 cm^{-1}.

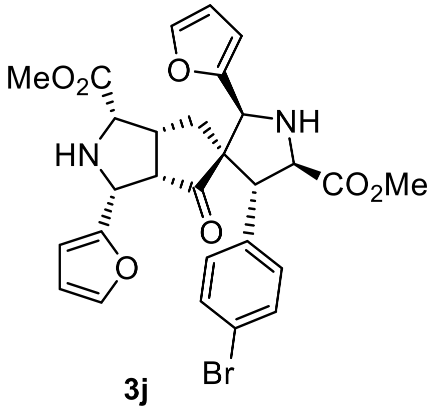

3j

Dimethyl (1*S*,2'*R*,3*R*,3a*S*,4'*R*,5*R*,5'*R*,6a*R*)-4'-(4-bromophenyl)-2',3-di(furan-3-yl)-4-oxohexahydro-1*H*-spiro[cyclopenta[*c*]pyrrole-5,3'-pyrrolidine]-1,5'-dicarboxylate

white amorphous solid, 45% yield ^{1}H NMR (600 MHz, CD_2Cl_2) δ 7.52 (d, *J* = 8.4 Hz, 2H), 7.39 – 7.38 (m, 1H), 7.36 – 7.34 (m, 1H), 7.11 (d, *J* = 8.4 Hz, 2H), 6.37 (dd, *J* = 3.4, 1.8 Hz, 1H), 6.34 (dd, *J* = 3.4, 1.8 Hz, 1H), 6.30 (d, *J* = 3.3 Hz, 1H), 6.15 (d, *J* = 3.3 Hz, 1H), 4.34-4.32 (m, 2H) 3.95 (d, *J* = 6.5 Hz, 1H), 3.76 (d, *J* = 5.7 Hz, 1H), 3.64 (s, 6H), 3.55 (d, *J* = 6.5 Hz, 1H), 2.72 – 2.67 (m, 1H), 2.51 – 2.47 (m, 1H), 1.62 – 1.55 (m, 2H).

^{13}C NMR (151 MHz, CD_2Cl_2) δ 216.64, 173.18, 171.46, 151.93, 151.60, 142.91, 142.46, 139.70, 132.17, 131.25, 121.60, 111.38, 110.93, 108.96, 108.75, 68.15, 67.16, 67.09, 63.45, 58.95, 55.89, 55.78, 52.63, 52.38, 42.25, 32.66.

HRMS: calcd. for $[M+H]^+$ $C_{28}H_{27}{}^{79}BrN_2O_7$ = 583.10769, found: 583.10744; calcd. for $[M+H]^+$ $C_{28}H_{27}{}^{81}BrN_2O_7$ = 585.10504, found: 585.10539;

HPLC conditions: CHIRALPAK IC column, *iso*-propanol/ *iso*-hexane = 50/50, flow rate = 0.5 mL min^{-1}, minor enantiomer: t_R = 27.54 min; major enantiomer: t_R = 74.1 min; (95% e.e.);

$[\alpha]^{20}_{D}$= + 41.1° (c = 0.21, $CHCl_3$).

FT-IR: $\tilde{\nu}$ 3449, 2942, 2863, 2158, 2049,1766, 1529, 1299, 1184, 1056, cm^{-1}.

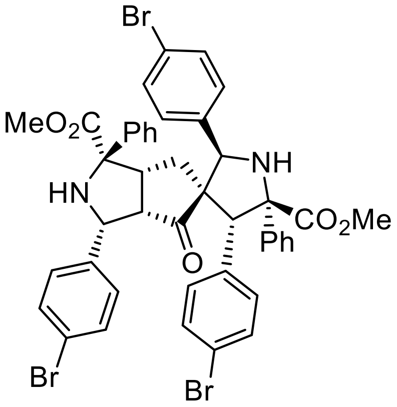

3k

Dimethyl (1*R*,2'*R*,3*R*,3*aS*,4'*R*,5*R*,5'*S*,6*aR*)-2',3,4'-tris(4-bromophenyl)-4-oxo-1,5'-diphenylhexahydro-1*H*-spiro[cyclopenta[*c*]pyrrole-5,3'-pyrrolidine]-1,5'-dicarboxylate

white amorphous solid, 21% yield,**^{1}H NMR** (700 MHz, CD_2Cl_2) δ 7.66 (d, *J* = 8.4 Hz, 2H), 7.61 (d, *J* = 7.2 Hz, 2H), 7.58 (d, *J* = 8.4 Hz, 2H), 7.34 – 7.22 (m, 10H), 6.98 (d, *J* = 7.2 Hz, 2H), 6.94 (d, *J* = 8.5 Hz, 2H), 6.51 (d, *J* = 8.5 Hz, 2H), 4.26 (s, 1H), 4.10 (d, *J* = 12.0 Hz, 1H), 3.94 – 3.89 (m, 2H), 3.67 (s, 3H), 3.31 (s, 3H), 2.53 – 2.50 (m, 1H), 2.39 (m, 1H), 2.30 (m, 1H), 1.75 (dd, *J* = 13.4, 12.0 Hz, 1H), 1.65 (m, 1H);

^{13}C NMR (176 MHz, CD_2Cl_2) δ 217.20, 173.93, 172.38, 144.25, 139.95, 137.92, 137.08, 136.68, 133.14, 132.47, 131.96, 131.54, 129.78, 129.30, 128.95, 128.57, 128.05, 127.79, 126.78, 126.14, 123.22, 122.21, 121.22, 75.05, 73.95, 71.37, 69.96, 66.14, 61.76, 55.36, 53.17, 52.82, 45.25, 31.62;

HRMS: calcd. for $[M+H]^+$ $C_{44}H_{38}{}^{79}Br{}^{81}Br_2N_2O_5$ = 915.02844, found: 915.02915; calcd. for $[M+H]^+$ $C_{44}H_{38}{}^{81}Br_3N_2O_5$ = 917.02640, found: 917.02760;

HPLC conditions: CHIRALPAK IC column, *iso*-propanol/ *iso*-hexane = 3/97, flow rate = 0.5 mL min^{-1}, minor enantiomer: t_R = 26.61 min; major enantiomer: t_R = 18.76 min; (91% e.e.);

$[\alpha]^{20}_{D}$= + 76.1° (c = 0.21, $CHCl_3$).

FT-IR: $\tilde{\nu}$ 3651, 2523, 2159, 2030, 1976, 1733, 1510, 1508, 1221, 1010 cm^{-1}

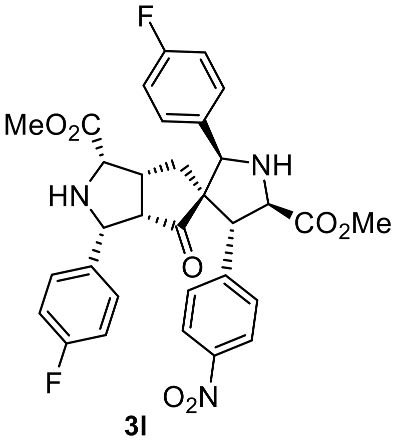

3l

Dimethyl (1*S*,2'*R*,3*R*,3a*S*,4'*R*,5*R*,5'*R*,6a*R*)-2',3-bis(4-fluorophenyl)-4'-(4-nitrophenyl)-4-oxohexahydro-1*H*-spiro[cyclopenta[*c*]pyrrole-5,3'-pyrrolidine]-1,5'-dicarboxylate

yellow amorphous solid, 80% yield, ^{1}**H NMR** (600 MHz, CD_2Cl_2) δ 8.30 – 8.22 (m, 2H), 7.39 – 7.34 (m, 2H), 7.30 – 7.24 (m, 2H), 7.11 – 6.98 (m, 6H), 4.32 (s, 1H), 4.17 (dd, *J* = 10.8, 3.7 Hz, 1H), 4.04-4.02 (m, 1H), 3.75-3.73 (m, 1H), 3.63 (s, 3H), 3.62 – 3.60 (m, 1H), 3.60 (s, 3H), 2.82 (m, 1H), 2.67 – 2.60 (m, 1H), 2.06 (bs, 1H), 2.02 – 1.97 (m, 1H), 1.83 – 1.79 (m, 1H), 1.60 – 1.52 (m, 1H);

^{13}**C NMR** (151 MHz, CD_2Cl_2) δ 215.81, 172.35, 170.92, 163.52 (d, *J* = 247 Hz), 162.91(d, *J* = 244.9 Hz), 147.20, 147.03, 134.82 (d, *J* = 2.5 Hz), 133.82 (d, *J* = 3.0 Hz), 130.10, 129.70 (d, *J* = 8.5 Hz), 129.22 (d, *J* = 7.9 Hz), 123.67, 115.67 (d, *J* = 21.5 Hz), 114.66 (d, *J* = 21.4 Hz), 74.24, 67.60, 66.27, 63.28, 62.41, 55.03, 54.95, 52.09, 51.68, 39.65, 33.99;

^{19}**F NMR** (565 MHz, CD_2Cl_2) δ -113.9 (m), -115.7 (m);

HRMS: calcd. for $[M+H]^+$ $C_{32}H_{29}F_2N_3O_7$ = 606.20604, found: 606.20463;

HPLC conditions: CHIRALPAK IC column, *iso*-propanol/ *iso*-hexane = 80/20, flow rate = 0.5 mL min^{-1}, minor enantiomer: t_R = 24.63 min; major enantiomer: t_R = 34.95 min; (95% e.e.);

$[\alpha]^{20}_{D}$= + 152.3° (c = 0.13, $CHCl_3$).

FT-IR: $\tilde{\nu}$ 3651, 2529, 2159, 2030, 1976, 1725, 1509, 1345, 1218, 1137, 1052 cm^{-1}.

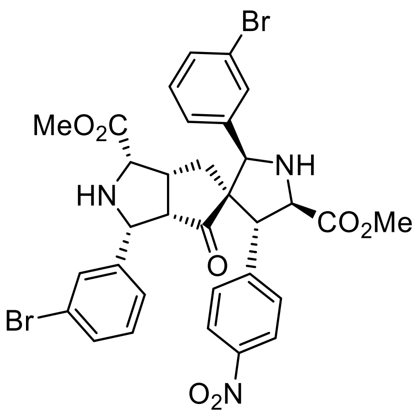

3m

Dimethyl (1*S*,2'*R*,3*R*,3a*S*,4'*R*,5*R*,5'*R*,6a*R*)-2',3-bis(3-bromophenyl)-4'-(4-nitrophenyl)-4-oxohexahydro-1*H*-spiro[cyclopenta[*c*]pyrrole-5,3'-pyrrolidine]-1,5'-dicarboxylate

white amorphous solid, 72%, **^{1}H NMR** (600 MHz, CD_2Cl_2) δ 8.26 (d, *J* = 8.7 Hz, 2H), 7.48 – 7.39 (m, 5H), 7.31 (d, *J* = 1.9 Hz, 1H), 7.26 – 7.17 (m, 3H), 7.02 (dd, *J* = 8.7, 1.9 Hz, 1H), 4.29 (s, 1H), 4.16 (d, *J* = 10.6 Hz, 1H), 4.06 (d, *J* = 7.2 Hz, 1H), 3.76 (d, *J* = 5.7 Hz, 1H), 3.63 (s, 3H), 3.60 (s, 3H), 2.68 – 2.60 (m, 1H), 2.07 (d, *J* = 7.2 Hz, 1H), 1.81 (dd, *J* = 13.6, 10.6 Hz, 1H), 1.58 – 1.51 (m, 2H);

^{13}C NMR (151 MHz, CD_2Cl_2) δ 215.98, 172.82, 171.32, 147.76, 147.47, 142.23, 141.08, 132.27, 131.55, 131.10, 130.99, 130.71, 130.67, 130.13, 127.47, 127.21, 124.39, 123.17, 122.66, 74.80, 68.24, 66.77, 63.89, 63.02, 55.67, 55.41, 52.71, 52.29, 40.06, 34.56;

HRMS: calcd. for $[M+H]^+$ $C_{32}H_{29}{}^{79}Br_2N_3O_7$ = 726.04689, found: 726.04450; calcd. for $[M+H]^+$ $C_{32}H_{29}{}^{79}Br^{81}BrN_3O_7$ = 728.04469, found: 728.04246; calcd. for $[M+H]^+$ $C_{32}H_{29}{}^{81}Br_2N_3O_7$ = 730.04311, found: 730.04041;

HPLC conditions: CHIRALPAK IA column, *iso*-propanol/ *iso*-hexane = 30/70, flow rate = 0.5 mL min^{-1}, minor enantiomer: t_R = 66.37 min; major enantiomer: t_R = 45.82 min; (91% e.e.);

$[\alpha]^{20}_D$= + 130.1° (c = 0.23, $CHCl_3$).

FT-IR: $\tilde{\nu}$ 3587, 2524, 2159, 2030, 1733, 1648, 1518, 1345, 1205, 1153, 1085 cm^{-1}.

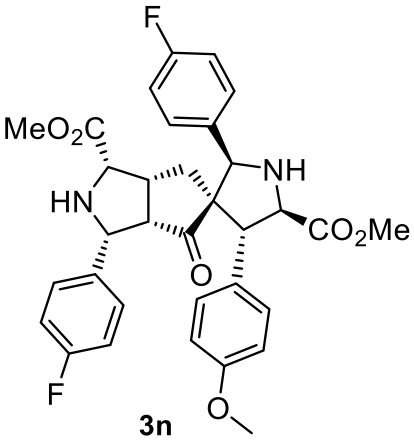

3n

Dimethyl (1*S*,2'*R*,3*R*,3a*S*,4'*R*,5*R*,5'*R*,6a*R*)-2',3-bis(4-fluorophenyl)-4'-(4-methoxyphenyl)-4-oxohexahydro-1*H*-spiro[cyclopenta[*c*]pyrrole-5,3'-pyrrolidine]-1,5'-dicarboxylate

white amorphous solid, 65%, ^{1}**H NMR** (700 MHz, CD_2Cl_2) δ 7.25 (d, *J* = 8.6, 2H), 7.09 (d, *J* = 8.6 Hz, 2H), 7.05 – 6.98 (m, 6H), 6.93 (d, *J* = 8.6 Hz, 2H), 4.27 (s, 1H), 4.14 (d, *J* = 10.8 Hz, 1H), 3.96 (d, *J* = 7.4 Hz, 1H), 3.84 (s, 3H), 3.73 (d, *J* = 5.8 Hz, 1H), 3.65 (s, 3H), 3.59 (s, 3H), 3.42 (d, *J* = 7.4 Hz, 1H), 2.63 – 2.57 (m, 1H), 2.00 – 1.97 (m, 1H), 1.80 – 1.75 (m, 1H), 1.63 (dd, *J* = 13.6, 10.8 Hz, 1H);

^{13}**C NMR** (176 MHz, CD_2Cl_2) δ 217.48, 173.58, 171.71, 163.86 (d, *J* = 246.5 Hz), 163.32 (d, *J* = 244.7 Hz), 159.42, 135.54 (d, *J* = 3.1 Hz), 134.82 (d, *J* = 3.0 Hz), 131.92, 130.72, 130.19 (d, *J* = 8.1 Hz), 130.02 (d, *J* = 8.0 Hz), 116.07 (d, *J* = 21.5 Hz), 115.18 (d, *J* = 21.4 Hz), 114.37, 74.91, 68.66, 67.47, 64.12, 63.18, 56.13, 55.79, 55.58, 52.48, 52.18, 40.44, 34.66; HRMS: calcd. for $[M+H]^+$ $C_{33}H_{32}F_2N_2O_6$ = 591.23147, found: 591.23012;

^{19}**F NMR** (565 MHz, CD_2Cl_2) δ -114.4 (m), -115.9 (m);

HPLC conditions: CHIRALPAK IC column, *iso*-propanol/ *iso*-hexane = 30/70, flow rate = 0.5 mL min^{-1}, minor enantiomer: t_R = 24.52 min; major enantiomer: t_R = 50.30 min; (98% e.e.);

[α]$^{20}_{D}$= + 146.7° (c = 0.1, $CHCl_3$).

FT-IR: $\tilde{\nu}$ 3527, 2511, 2477, 1755, 1585, 1558, 1297, 1166, 1097, 1012 cm^{-1}.

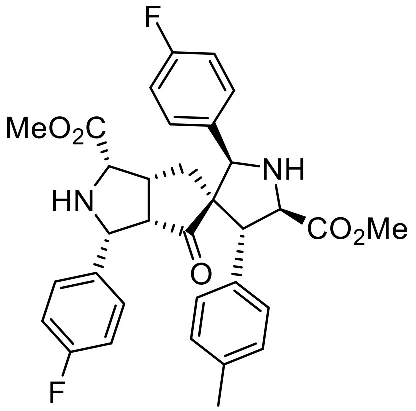

3o

Dimethyl *(*1*S*,2'*R*,3*R*,3*aS*,4'*R*,5*R*,5'*R*,6*aR*)-2',3-bis(4-fluorophenyl)-4-oxo-4'-(*p*-tolyl)hexahydro-1*H*-spiro[cyclopenta[*c*]pyrrole-5,3'-pyrrolidine]-1,5'-dicarboxylate

white amorphous solid, 60%, **^{1}H NMR** (600 MHz, CD_2Cl_2) δ 7.27 – 7.24 (m, 2H), 7.22 (d, *J* = 7.9 Hz, 2H), 7.08 – 6.96 (m, 8H), 4.27 (s, 1H), 4.14 (d, *J* = 10.7 Hz, 1H), 3.99 (d, *J* = 7.3 Hz, 1H), 3.72 (d, *J* = 5.7 Hz, 1H), 3.64 (s, 3H), 3.62 (d, *J* = 7.3Hz, 1H), 3.59 (s, 3H), 2.61 -2.53 (m, 1H), 2.39 (s, 3H), 2.01 -1.95 (m, 1H), 1.75 (m, 1H), 1.62 (dd, *J* = 13.6, 10.7 Hz, 1H);

^{13}C NMR (151 MHz, CD_2Cl_2) δ 217.52, 173.54, 171.71, 163.97 (d, *J* = 246.5 Hz), 163.42 (d, *J* = 244.7 Hz), 137.59, 136.94, 135.52 (d, *J* = 2.9 Hz), 134.71 (d, *J* = 3.2 Hz), 130.18 (d, *J* = 8.1 Hz), 130.02 (d, *J* = 8.0 Hz), 129.73, 129.56, 116.08 (d, *J* = 21.4 Hz), 115.19 (d, *J* = 21.4 Hz), 74.93, 68.57, 67.41, 64.13, 63.16, 56.14, 55.93, 52.48, 52.16, 40.44, 34.62, 21.39;

^{19}F NMR (565 MHz, CD_2Cl_2) δ -114.3 (m), -115.9 (m);

HRMS: calcd. for $[M+H]^+$ $C_{33}H_{32}F_2N_2O_5$ = 575.23641, found: 575.23520;

HPLC conditions: CHIRALPAK IC column, *iso*-propanol/ *iso*-hexane = 30/70, flow rate = 0.5 mL min^{-1}, minor enantiomer: t_R = 18.23 min; major enantiomer: t_R = 143.3 min; (98% e.e.);

$[\alpha]^{20}_D$= + 41.1° (c = 0.15, $CHCl_3$).

FT-IR: $\tilde{\nu}$ 3498, 2511, 2159, 2049, 2022, 1731, 1508, 1218, 1035, 1011 cm^{-1}.

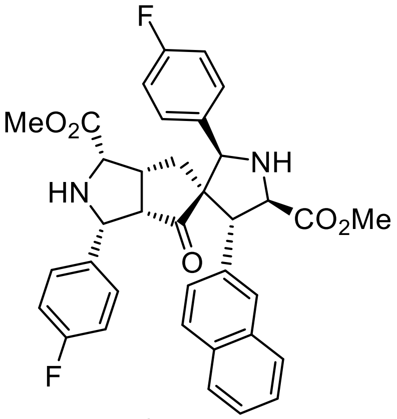

3p

Dimethyl (1*S*,2'*R*,3*R*,3a*S*,4'*R*,5*R*,5'*R*,6a*R*)-2',3-bis(4-fluorophenyl)-4'-(naphthalen-2-yl)-4-oxohexahydro-1*H*-spiro[cyclopenta[*c*]pyrrole-5,3'-pyrrolidine]-1,5'-dicarboxylate

brown amorphous solid, 65%, **^{1}H NMR** (500 MHz, CD_2Cl_2) δ 7.92 – 7.88 (m, 3H), 7.61 (d, *J* = 1.9 Hz, 1H), 7.58 – 7.50 (m, 2H), 7.34 (dd, *J* = 8.5, 1.9 Hz, 1H), 7.31 – 7.27 (m, 2H), 7.09 – 6.94 (m, 6H), 4.39 (s, 1H), 4.16-4.14 (m, 2H), 3.71 (d, *J* = 5.7 Hz, 1H), 3.66 (d, *J* = 7.3 Hz, 1H), 3.57 (s, 3H), 3.52 (s, 3H), 2.83 (bs, 1H), 2.66 – 2.57 (m, 1H), 2.03 – 1.98 (m, 1H), 1.83 (m, 1H), 1.67 (dd, *J* = 13.6, 10.7 Hz, 1H);

^{13}C NMR (126 MHz, CD_2Cl_2) δ 217.24, 173.54, 171.60, 163.15 (d, *J* = 246.5 Hz), 162.57 (d, *J* = 244.6 Hz), 137.35, 135.44 (d, *J* = 3.0 Hz), 134.73 (d, *J* = 3.2 Hz), 133.91, 133.14, 130.21 (d, *J* = 8.1 Hz), 129.96 (d, *J* = 8.0 Hz), 128.71, 128.61, 128.32, 128.20, 127.50, 126.89, 126.56, 116.12 (d, *J* = 21.5 Hz), 115.19 (d, *J* = 21.4 Hz), 74.92, 68.66, 67.13, 64.05, 63.08, 56.33, 55.93, 52.54, 52.08, 40.45, 34.62;

^{19}F NMR (565 MHz, CD_2Cl_2) δ -114.3 (m), -115.9 (m);

HRMS: calcd. for $[M+H]^+$ $C_{36}H_{32}F_2N_2O_5$ = 611.23648, found: 611.23520;

HPLC conditions: CHIRALPAK IC column, *iso*-propanol/ *iso*-hexane = 30/70, flow rate = 0.5 mL min^{-1}, minor enantiomer: t_R = 20.03 min; major enantiomer: t_R = 44.11 min; (95% e.e.);

$[\alpha]^{20}_D$= + 168.4° (c = 0.17, $CHCl_3$).

FT-IR: $\tilde{\nu}$ 3402, 2460, 2108, 1680, 1588, 1398, 1212, 1187, 1078, 1019 cm^{-1}**.**

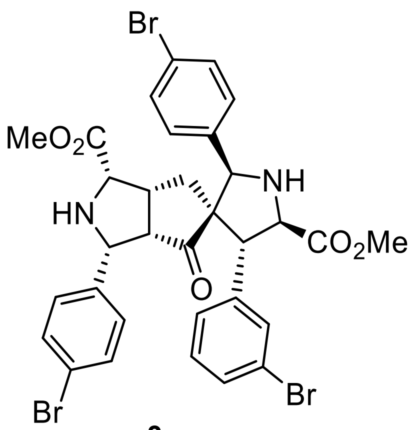

3q

Dimethyl (1*S*,2'*R*,3*R*,3a*S*,4'*R*,5*R*,5'*R*,6a*R*)-4'-(3-bromophenyl)-2',3-bis(4-bromophenyl)-4-oxohexahydro-1*H*-spiro[cyclopenta[*c*]pyrrole-5,3'-pyrrolidine]-1,5'-dicarboxylate

white amorphous solid, 81% yield, **^{1}H NMR** (400 MHz, CD_2Cl_2) δ 7.50 – 7.44 (m, 5H), 7.33 – 7.27 (m, 2H), 7.18 – 7.11 (m, 3H), 6.97 (d, *J* = 8.4 Hz, 2H), 4.24 (s, 1H), 4.13 (d, *J* = 10.2 Hz, 1H), 3.99 (d, *J* = 7.2 Hz, 1H), 3.78 – 3.74 (m, 1H), 3.66 (s, 3H), 3.61 (s, 3H), 3.45 (d, *J* = 7.2 Hz, 1H), 2.75 (s, 1H), 2.68 – 2.58 (m, 1H), 2.00 (dd, *J* = 13.5, 10.2 Hz, 1H), 1.78 (m, 1H), 1.61 – 1.52 (m, 2H);

^{13}C NMR (101 MHz, CD_2Cl_2) δ 216.49, 173.14, 171.57, 142.24, 138.82, 137.91, 132.64, 132.33, 131.52, 131.02, 130.68, 130.26, 130.07, 128.45, 123.10, 122.87, 121.55, 74.87, 68.29, 66.84, 64.07, 63.17, 55.75, 55.52, 52.68, 52.23, 40.25, 34.73;

HRMS: calcd. for $[M+H]^+$ $C_{32}H_{29}{}^{79}Br_3N_2O_5$ = 758.97254, found: 758.96994; calcd. for $[M+H]^+$ $C_{32}H_{29}{}^{79}Br^{81}Br_2N_2O_5$ = 762.96848, found: 762.96584; calcd. for $[M+H]^+$ $C_{32}H_{29}{}^{79}Br_2{}^{81}BrN_2O_5$ = 760.97050, found: 760.96789; calcd. for $[M+H]^+$ $C_{32}H_{29}{}^{81}Br_3N_2O_5$ = 764.96625, found: 764.96380;

HPLC conditions: CHIRALPAK IC column, *iso*-propanol/ *iso*-hexane = 30/70, flow rate = 0.5 mL min^{-1}, minor enantiomer: t_R = 18.90min; major enantiomer: t_R = 32.07 min; (91% e.e.);

$[\alpha]^{20}{}_D$= + 107.8° (c = 0.24, $CHCl_3$).

FT-IR: $\tilde{\nu}$ 3488, 2511, 2159, 2057, 2014, 1732, 1659, 1428, 1211, 1142, 1089 cm^{-1}.

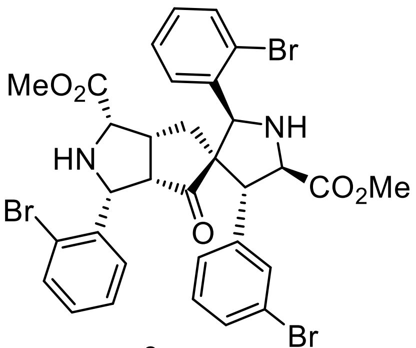

3r

Dimethyl (1*S*,2'*S*,3*R*,3a*S*,4'*R*,5*R*,5'*R*,6a*R*)-2',3-bis(2-bromophenyl)-4'-(3-bromophenyl)-4-oxohexahydro-1*H*-spiro[cyclopenta[*c*]pyrrole-5,3'-pyrrolidine]-1,5'-dicarboxylate

white amorphous solid, 60% yield, **^{1}H NMR** (700 MHz, CD_2Cl_2) δ 7.61 (dd, *J* = 8.0, 1.3 Hz, 1H), 7.53 (dd, *J* = 8.0, 1.3 Hz, 1H), 7.48 – 7.46 (m, 1H), 7.35 – 7.32 (m, 2H), 7.29 – 7.15 (m, 6H), 7.11 (m, 1H), 4.93 (s, 1H), 4.37 (d, *J* = 10.2 Hz, 1H), 4.01 (d, *J* = 8.0 Hz, 1H), 3.76 (d, *J* = 6.0 Hz, 1H), 3.65 (s, 3H), 3.58 (s, 3H), 3.46 (d, *J* = 8.0 Hz, 1H), 2.89 – 2.84 (m, 1H), 2.30 – 2.24 (m, 1H), 2.05 – 2.01 (m, 1H), 1.40 (dd, *J* = 13.4, 10.2 Hz, 1H); **^{13}C NMR** (176 MHz, CD_2Cl_2) δ 216.03, 173.00, 171.65, 142.33, 139.26, 137.78, 133.74, 132.81, 132.55, 130.88, 130.67, 130.25, 130.14, 129.18, 128.68, 128.56, 128.31, 127.84, 125.08, 124.89, 122.98, 71.42, 68.95, 66.50, 63.55, 62.92, 55.50, 52.80, 52.62, 52.14, 39.74, 34.15;

HRMS: calcd. for $[M+H]^+$ $C_{32}H_{30}{}^{79}Br_2{}^{81}BrN_2O_5$ = 759.97549, found: 759.97264; calcd. for $[M+H]^+$ $C_{32}H_{30}{}^{79}Br^{81}Br_2N_2O_5$ = 761.97320, found: 761.97059; calcd. for $[M+H]^+$ $C_{32}H_{30}{}^{81}Br_3N_2O_5$ = 763.97095, found: 763.96855;

HPLC conditions: CHIRALPAK IC column, *iso*-propanol/ *iso*-hexane = 30/70, flow rate = 0.5 mL min^{-1}, minor enantiomer: t_R = 19.44 min; major enantiomer: t_R = 39.94 min; (91% e.e.);

$[\alpha]^{20}_{D}$= + 131.2° (c = 0.35, $CHCl_3$).

FT-IR: $\tilde{\nu}$ 3476, 2508, 2129, 2156, 1998, 1734, 1642, 1208, 1112, 1021 cm^{-1}.

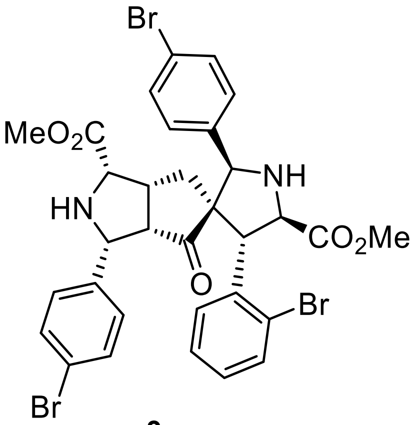

3s

Dimethyl (1*S*,2'*S*,3*R*,3a*S*,4'*R*,5*R*,5'*R*,6a*R*)-2',3-bis(2-bromophenyl)-4'-(3-bromophenyl)-4-oxohexahydro-1*H*-spiro[cyclopenta[*c*]pyrrole-5,3'-pyrrolidine]-1,5'-dicarboxylate

white amorphous solid, 80% yield, **^{1}H NMR** (600 MHz, CD_2Cl_2) δ 7.66 (dd, *J* = 8.1, 1.3 Hz, 1H), 7.49 (d, *J* = 8.4 Hz, 2H), 7.46 (d, *J* = 8.1 Hz, 1H), 7.43 (d, *J* = 8.4 Hz, 2H), 7.41 – 7.38 (m, 1H), 7.22 – 7.15 (m, 5H), 4.30 (s, 1H), 4.21 (d, *J* = 4.8 Hz, 1H), 4.04 (d, *J* = 10.2 Hz, 1H), 3.88 (d, *J* = 4.8 Hz, 1H), 3.66 (m, 4H), 3.58 (s, 3H), 2.90 (bs, 1H), 2.50 – 2.43 (m, 1H), 2.10 (bs, 1H), 1.81 – 1.78 (m, 1H), 1.68 – 1.60 (m, 2H);

^{13}C NMR (151 MHz, CD_2Cl_2) δ 216.98, 173.62, 171.50, 140.91, 138.27, 137.11, 133.62, 132.48, 131.35, 130.35, 130.13, 130.07, 129.33, 128.63, 126.10, 123.02, 121.50, 74.99, 68.33, 64.61, 63.63, 55.99, 52.75, 52.07, 40.17, 33.90;

HRMS: calcd. for $[M+H]^+$ $C_{32}H_{29}{}^{79}Br_3N_2O_5$ = 758.96994, found: 758.97286; calcd. for $[M+H]^+$ $C_{32}H_{30}{}^{79}Br_2{}^{81}BrN_2O_5$ = 760.96789, found: 760.97090; calcd. for $[M+H]^+$ $C_{32}H_{20}{}^{81}Br_3N_2O_5$ = 764.96380, found: 764.96625**;**

HPLC conditions: CHIRALPAK IC column, *iso*-propanol/ *iso*-hexane = 30/70, flow rate = 0.5 mL min^{-1}, minor enantiomer: t_R = 21.32 min; major enantiomer: t_R = 39.24 min; (95% e.e.);

$[\alpha]^{20}_{D}$= + 22.1° (c = 0.1, $CHCl_3$).

FT-IR: $\tilde{\nu}$ 3335, 2512, 2187, 2156, 2011, 1728, 1648, 1203, 1098 cm^{-1}.

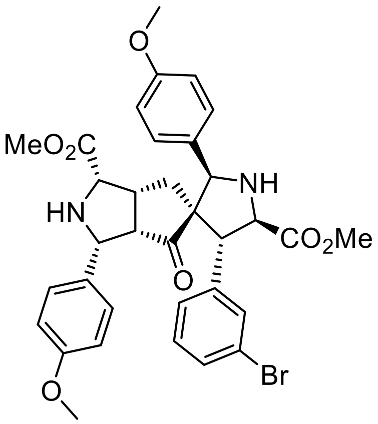

3t

Dimethyl (1*S*,2'*R*,3*R*,3a*S*,4'*R*,5*R*,5'*R*,6a*R*)-4'-(3-bromophenyl)-2',3-bis(4-methoxyphenyl)-4-oxohexahydro-1*H*-spiro[cyclopenta[*c*]pyrrole-5,3'-pyrrolidine]-1,5'-dicarboxylate

white amorphous solid, 55% yield, **^{1}H NMR** (600 MHz, CD_2Cl_2) δ 7.49 – 7.47 (m, 1H), 7.35 (d, *J* = 10.3 Hz, 1H), 7.30 (m, 1H), 7.16-7.12 (m, 3H), 6.95 (d, *J* = 8.6 Hz, 2H), 6.86 (dd, *J* = 10.3, 8.6 Hz, 4H), 4.19 (s, 1H), 4.09 (d, *J* = 10.7 Hz, 1H), 3.95 (d, *J* = 7.5 Hz, 1H), 3.82 (s, 3H), 3.80 (s, 3H), 3.70 (d, *J* = 5.7 Hz, 1H), 3.65 (s, 3H), 3.60 (s, 3H), 3.44 (d, *J* = 7.5 Hz, 1H), 2.75 (bs, 1H), 2.64 – 2.57 (m, 1H), 2.01 (bs, 1H), 1.94 (dd, *J* = 10.7, 8.9 Hz, 1H), 1.78 – 1.74 (m, 1H), 1.55 – 1.51 (m, 1H);

^{13}C NMR (151 MHz, CD_2Cl_2) δ 217.44, 173.17, 171.87, 160.23, 159.55, 142.83, 132.74, 131.54, 130.86, 130.63, 130.06, 129.61, 129.41, 128.45, 122.99, 114.46, 113.73, 75.62, 68.36, 67.32, 64.54, 63.17, 56.16, 56.09, 55.77, 55.73, 52.55, 52.15, 40.73, 34.88;

HRMS: calcd. for $[M+H]^+$ $C_{34}H_{35}{}^{79}BrN_2O_7$ = 663.17181, found: 663.17004; calcd. for $[M+H]^+$ $C_{34}H_{35}{}^{81}BrN_2O_7$ = 665.17000, found: 665.16799;

HPLC conditions: CHIRALPAK IC column, *iso*-propanol/ *iso*-hexane = 80/20, flow rate = 0.5 mL min^{-1}, minor enantiomer: t_R = 27.41 min; major enantiomer: t_R = 51.85 min; (91% e.e.)

$[\alpha]^{20}_D$= + 128.2° (c = 0.12, $CHCl_3$).

FT-IR: $\tilde{\nu}$ 3412, 2512, 2167, 2114, 1766, 1598, 1225, 1181, 1103, 1036 cm^{-1}.

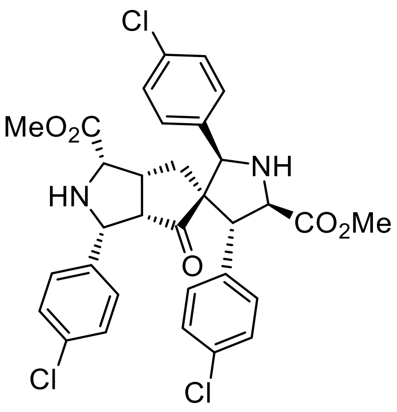

3u

Dimethyl (1*S*,2'*R*,3*R*,3*aS*,4'*R*,5*R*,5'*R*,6*aR*)-2',3,4'-tris(4-chlorophenyl)-4-oxohexahydro-1*H*-spiro[cyclopenta[*c*]pyrrole-5,3'-pyrrolidine]-1,5'-dicarboxylate

white amorphous solid, 80% yield, **^{1}H NMR** (500 MHz, CD_2Cl_2) δ 7.38 (d, *J* = 8.4 Hz, 2H), 7.31 (d, *J* = 8.5 Hz, 2H), 7.27 (d, *J* = 8.4 Hz, 2H), 7.21 (d, *J* = 8.5 Hz, 2H), 7.12 (d, *J* = 8.5 Hz, 2H), 7.02 (d, *J* = 8.5 Hz, 2H), 4.26 (s, 1H), 4.15 (d, *J* = 10.7 Hz, 1H), 3.97 (d, *J* = 7.1 Hz, 1H), 3.75 (d, *J* = 5.7 Hz, 1H), 3.65 (s, 3H), 3.59 (s, 3H), 3.45 (d, *J* = 7.1 Hz, 1H), 2.75 (bs, 1H), 2.63 – 2.57 (m, 1H), 2.07 – 1.99 (m, 1H), 1.78 – 1.73 (m, 1H), 1.59 (dd, *J* = 13.5, 10.7 Hz, 1H);

^{13}C NMR (126 MHz, CD_2Cl_2) δ 216.74, 173.28, 171.54, 138.47, 138.35, 137.38, 134.62, 133.54, 133.34, 131.08, 129.85, 129.68, 129.34, 129.19, 128.47, 74.66, 68.34, 67.02, 63.90, 63.07, 55.87, 55.26, 52.60, 52.24, 40.23, 34.45;

HRMS: calcd. for $[M+H]^+$ $C_{32}H_{30}{}^{35}Cl_3N_2O_5$ = 627.12301, found: 627.12148; calcd. for $[M+H]^+$ $C_{32}H_{30}{}^{35}Cl_2{}^{37}ClN_2O_5$ = 629.12023, found: 629.11853; calcd. for $[M+H]^+$ $C_{32}H_{30}{}^{35}Cl^{37}Cl_2N_2O_5$ = 631.11700, found: 631.11558;

HPLC conditions: CHIRALPAK IC column, *iso*-propanol/ *iso*-hexane = 30/70, flow rate = 0.5 mL min^{-1}, minor enantiomer: t_R = 35.56 min; major enantiomer: t_R = 56.67 min; (95% e.e.);

$[\alpha]^{20}_{D}$= + 95.6° (c = 0.18, $CHCl_3$).

FT-IR: $\tilde{\nu}$ 3357, 2360, 2238, 1732, 1491, 1434, 1207, 1108, 1089, 1013, cm^{-1}.

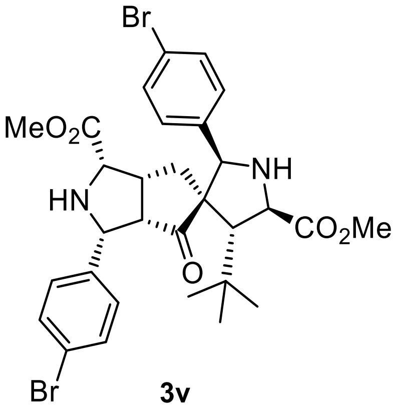

3v

Dimethyl (1*S*,2'*R*,3*R*,3a*S*,4'*R*,5*R*,5'*R*,6a*R*)-2',3-bis(4-bromophenyl)-4'-(tert-butyl)-4-oxohexahydro-1*H*-spiro[cyclopenta[*c*]pyrrole-5,3'-pyrrolidine]-1,5'-dicarboxylate

white amorphous solid, 21% yield, **^{1}H NMR** (700 MHz, CD_2Cl_2) δ 7.44 (d, *J* = 8.5 Hz, 2H), 7.40 (d, *J* = 8.4 Hz, 2H), 7.14 (d, *J* = 8.4 Hz, 2H), 7.10 (d, *J* = 8.5 Hz, 2H), 4.16 (d, *J* = 10.3 Hz, 1H), 4.03 (s, 1H), 3.78 (s, 3H), 3.70 (d, *J* = 7.1 Hz, 1H), 3.66 (s, 3H), 2.58 – 2.51 (m, 1H), 2.46 (dd, *J* = 13.0, 10.3 Hz, 1H), 2.36 (d, *J* = 7.1 Hz, 1H), 2.16 (d, *J* = 7.0 Hz, 1H), 2.14 – 2.07 (m, 1H), 1.91-1.89 (m, 1H), 0.98 (s, 9H);

^{13}C NMR (176 MHz, CD_2Cl_2) δ 216.64, 175.27, 172.00, 138.81, 138.44, 132.23, 131.23, 130.34, 129.83, 122.73, 121.33, 75.49, 68.56, 64.03, 63.60, 58.94, 54.79, 52.52, 52.33, 40.39, 34.42, 33.74, 30.10;

HRMS: calcd. for $[M+H]^+$ $C_{30}H_{35}{}^{79}Br_2N_2O_5$ = 661.09072, found: 661.09165; calcd. for $[M+H]^+$ $C_{30}H_{35}{}^{79}Br^{81}BrN_2O_5$ = 663.08868, found: 663.08948; calcd. for $[M+H]^+$ $C_{30}H_{35}{}^{81}Br_2N_2O_5$ = 665.08663, found: 665.08771**;**

HPLC conditions: CHIRALPAK IC column, *iso*-propanol/ *iso*-hexane = 30/70, flow rate = 0.5 mL min^{-1}, minor enantiomer: t_R = 40.02 min; major enantiomer: t_R = 40.02 min; (94% e.e.);

$[\alpha]^{20}_D$= - 15.0° (c = 0.1, $CHCl_3$).

FT-IR: $\tilde{\nu}$ 3185, 2535, 2441, 2031, 1617, 1534, 1211, 1189, 1089 cm^{-1}.

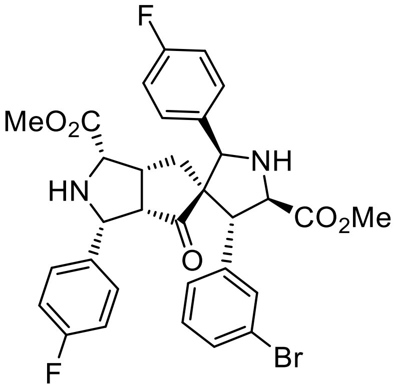

3w

Dimethyl (1*S*,2'*R*,3*R*,3a*S*,4'*R*,5*R*,5'*R*,6a*R*)-4'-(3-bromophenyl)-2',3-bis(4-fluorophenyl)-4-oxohexahydro-1*H*-spiro[cyclopenta[*c*]pyrrole-5,3'-pyrrolidine]-1,5'-dicarboxylate

white solid, 75%, **^{1}H NMR** (600 MHz, CD_2Cl_2) δ 7.48 (d, *J* = 8.6 Hz, 1H), 7.33-7.31 (m, 1H), 7.30-7.28 (m, 1H), 7.28 – 7.24 (m, 2H), 7.14 – 7.13 (m, 1H), 7.06 – 7.01 (m, 6H), 4.27 (s, 1H), 4.14 (d, *J* = 10.6 Hz, 1H), 3.99 (d, *J* = 7.4 Hz, 1H), 3.74 (d, *J* = 5.8 Hz, 1H), 3.66 (s, 3H), 3.60 (s, 3H), 3.46 (d, *J* = 7.4 Hz, 1H), 2.75 (s, 1H), 2.65 – 2.59 (m, 1H), 2.05 (bs, 1H), 1.97 – 1.94 (m, 1H), 1.79 (dd, *J* = 13.6, 10.6 Hz 1H), 1.59-1.54 (m, 1H);

^{13}C NMR (151 MHz, CD_2Cl_2) δ 216.79, 173.14, 171.65, 164.02 (d, *J* = 246.7 Hz), 163.44 (d, *J* = 244.7 Hz), 142.36, 135.40 (d, *J* = 2.9 Hz), 134.56 (d, *J* = 3.1 Hz), 132.67, 130.98, 130.66, 130.28 (d, *J* = 8.2 Hz), 129.90 (d, *J* = 8.0 Hz), 128.45, 123.06, 116.14 (d, *J* = 21.5 Hz), 115.26 (d, *J* = 21.4 Hz), 74.93, 68.26, 66.91, 64.05, 63.14, 55.76, 55.66, 52.60, 52.21, 40.33, 34.83;

^{19}F NMR (565 MHz, CD2Cl2) δ -114.1 (m), -114.8 (m);

HRMS: calcd. for $[M+H]^+$ $C_{32}H_{30}{}^{79}BrF_2N_2O_5$ = 639.13182, found: 639.13007; calcd. for $[M+H]^+$ $C_{32}H_{30}{}^{81}BrF_2N_2O_5$ = 641.13007, found: 641.12802;

HPLC conditions: CHIRALPAK IC column, *iso*-propanol/ *iso*-hexane = 30/70, flow rate = 0.5 mL min^{-1}, minor enantiomer: t_R = 22.39 min; major enantiomer: t_R = 41.27 min; (95% e.e.);

[α]$^{20}_{D}$= + 138.5° (c = 0.2, $CHCl_3$).

FT-IR: $\tilde{\nu}$ 3312, 2412, 2067, 2014, 1734, 1558, 1223, 1103, 1025 cm^{-1}.

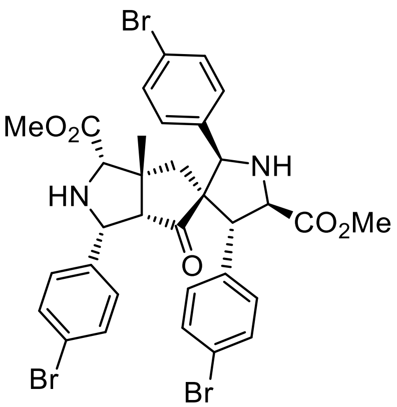

3x

Dimethyl (1R,2'R,3S,3aR,4'R,5R,5'R,6aS)-1,2',4'-tris(4-bromophenyl)-3a-methyl-6-oxohexahydro-1H-spiro[cyclopenta[c]pyrrole-5,3'-pyrrolidine]-3,5'-dicarboxylate

white amorphous solid, 40% yield; ^{1}**H NMR** (700 MHz, CD_2Cl_2) δ 7.53 (d, *J* = 8.5 Hz, 2H), 7.45-7.42 (m, 4H), 7.16 – 7.09 (m, 6H), 4.21 (s, 1H), 4.19 (d, *J* = 9.1 Hz, 1H), 4.02 (d, *J* = 5.5 Hz, 1H), 3.60 (s, 3H), 3.57 (s, 3H), 3.54 (d, *J* = 5.5 Hz, 1H), 3.44 (s, 1H), 2.00 (d, *J* = 9.1 Hz, 1H), 1.88 (d, *J* = 14.8 Hz, 1H), 1.54 (s, 2H), 1.43 (d, *J* = 14.8 Hz, 1H), 0.98 (s, 3H);

^{13}**C NMR** (176 MHz, CD_2Cl_2) δ 218.44, 173.66, 171.60, 139.40, 138.09, 137.65, 132.24, 132.07, 131.94, 131.43, 130.81, 129.58, 122.88, 121.69, 121.39, 74.26, 72.00, 66.94, 66.65, 65.10, 64.26, 55.99, 52.86, 52.09, 46.81, 40.22, 26.90;

HRMS: calcd. for $[M+H]^+$ $C_{33}H_{32}{}^{79}Br_2{}^{81}BrN_2O_5$ = 774.98354, found: 774.98395; calcd. for $[M+H]^+$ $C_{33}H_{32}{}^{79}Br^{81}Br_2N_2O_5$ = 776.98149, found: 776.98137;

HPLC conditions: CHIRALPAK IC column, *iso*-propanol/ *iso*-hexane = 15/85, flow rate = 0.5 mL min^{-1}, minor enantiomer: t_R = 37.47 min; major enantiomer: t_R = 50.97 min; (94% e.e.);

$[\alpha]^{20}_{D}$= + 125.8° (c = 0.18, $CHCl_3$).

FT-IR: $\tilde{\nu}$ 3332, 2360, 2159, 2030, 1715, 1655, 1453, 1215, 1122, 1095, cm^{-1}.

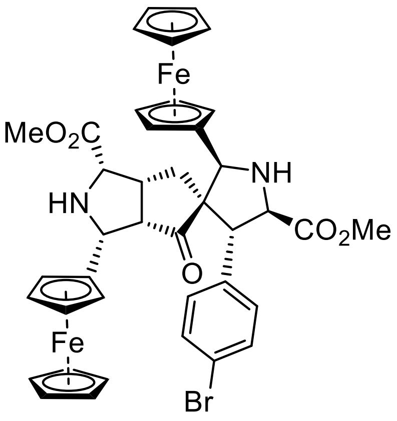

3y

Dimethyl (1*S*,2'*R*,3*R*,3a*S*,4'*R*,5*R*,5'*R*,6a*R*)-2',3,4'-tris(4-chlorophenyl)-4-oxohexahydro-1*H*-spiro[cyclopenta[*c*]pyrrole-5,3'-pyrrolidine]-1,5'-dicarboxylate

white amorphous solid, 48% yield; ^{1}**H NMR** (500 MHz, CD_2Cl_2) δ 7.46 (d, *J* = 8.5 Hz, 2H), 7.01 (d, *J* = 8.5 Hz, 2H), 4.21 (s, 5H), 4.17 (dd, *J* = 2.5, 1.3 Hz, 1H), 4.14 (dd, *J* = 2.7, 1.3 Hz, 1H), 4.13 – 4.12 (m, 1H), 4.11 – 4.10 (m, 3H), 4.08 (s, 5H), 4.03 (dd, *J* = 2.7, 1.3 Hz, 1H), 3.90 (s, 1H), 3.81 (d, *J* = 6.2 Hz, 1H), 3.78 (d, *J* = 2.5 Hz, 1H), 3.75 – 3.74 (m, 1H), 3.66 (s, 3H), 3.60 (s, 3H), 3.17 (d, *J* = 6.2 Hz, 1H), 2.77 – 2.72 (m, 1H), 2.09 – 2.06 (m, 1H), 1.47 – 1.37 (m, 2H).

^{13}**C NMR** (126 MHz, CD_2Cl_2) δ 217.04, 173.95, 172.32, 139.95, 131.95, 131.33, 121.22, 87.66, 85.56, 70.52, 69.77, 69.45, 69.42, 69.11, 69.03, 68.93, 68.50, 68.08, 67.81, 67.43, 67.09, 66.09, 65.61, 63.06, 60.83, 56.73, 55.99, 52.47, 52.26, 42.33, 33.48.

HRMS: calcd. for $[M+H]^+$ $C_{40}H_{40}{}^{79}Br^{56}Fe_2N_2O_5$ = 819.08140, found: 819.08073; calcd. for $[M+H]^+$ $C_{40}H_{40}{}^{79}Br^{54}Fe^{56}FeN_2O_5$ = 817.08607, found: 817.08544; calcd. for $[M+H]^+$ $C_{40}H_{40}{}^{81}Br^{54}Fe^{57}FeN_2O_5$ = 820.08448, found: 820.08390; calcd. for $[M+H]^+$ $C_{40}H_{40}{}^{81}Br^{56}Fe_2N_2O_5$ = 821.07935, found: 821.07887; calcd. for $[M+H]^+$ $C_{40}H_{40}{}^{79}Br^{57}Fe^{58}FeN_2O_5$ = 822.08019, found: 822.08132;

HPLC conditions: CHIRALPAK IC column, *iso*-propanol/ *iso*-hexane = 15/85, flow rate = 0.5 mL min^{-1}, minor enantiomer: t_R = 37.47 min; major enantiomer: t_R = 50.97 min; (94% e.e.);

$[\alpha]^{20}_{D}$= + 98.5° (c = 0.18, $CHCl_3$).

FT-IR: $\tilde{\nu}$ 3344, 2947, 2847, 1732, 1488, 1436, 1245, 1220, 1151, 1104, 1007, 967 cm^{-1}.

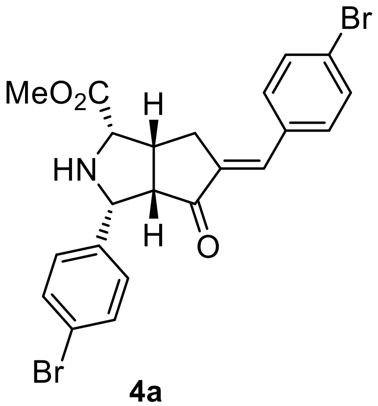

4a

Methyl (1*S*,3*R*,3a*S*,6a*R*)-5-((*E*)-4-bromobenzylidene)-3-(4-bromophenyl)-4-oxooctahydrocyclopenta[*c*]pyrrole-1-carboxylate

white amorphous solid, 72% yield, **^{1}H NMR** (500 MHz, CD_2Cl_2) δ 7.56 (d, *J* = 8.5 Hz, 2H), 7.41 – 7.35 (m, 4H), 7.20 (d, *J* = 8.5 Hz, 2H), 7.01-6.97 (m, 1H), 4.53 (d, *J* = 10.4 Hz, 1H), 4.14 (d, *J* = 5.5 Hz, 1H), 3.83 (s, 3H), 3.31 – 3.07 (m, 3H), 2.78 (m, 1H), 2.41 (bs, 1H); **^{13}C NMR** (126 MHz, CD_2Cl_2) δ 206.19, 172.11, 138.99, 138.01, 134.86, 132.71, 132.45, 131.58, 131.44, 129.93, 124.22, 121.58, 64.99, 64.54, 56.31, 52.51, 40.72, 31.95; **HRMS**: calcd. for $[M+H]^+$ $C_{22}H_{20}{}^{79}Br_2NO_3$ = 503.98039, found: 503.98045; calcd. for $[M+H]^+$ $C_{22}H_{20}{}^{79}Br^{81}BrNO_3$ = 505.97827, found: 505.97840; calcd. for $[M+H]^+$ $C_{22}H_{20}{}^{81}Br_2NO_3$ = 507.97602, found: 507.97635;

HPLC conditions: CHIRALPAK IC column, *iso*-propanol/ *iso*-hexane = 30/70, flow rate = 0.5 mL min^{-1}, minor enantiomer: t_R = 40.63 min; major enantiomer: t_R = 28.16min; (81% e.e.); CHIRALPAK IA column, *iso*-propanol/ *iso*-hexane = 15/85, flow rate = 0.5 mL min^{-1}, minor enantiomer: t_R = 36.68 min; major enantiomer: t_R = 93.25 min;

$[\alpha]^{20}_D$= + 251.5° (c = 0.2, $CHCl_3$).

FT-IR: $\tilde{\nu}$ 3378, 2514, 2058, 1744, 1525, 1488, 1221, 1155, 1093, 1008 cm^{-1}.

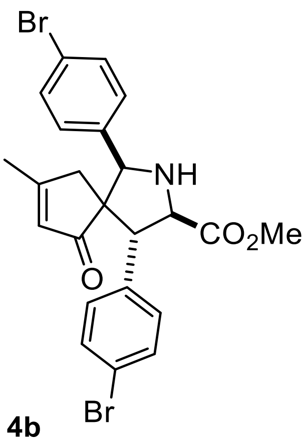

4b

rac-Methyl (1*R*,3*R*,4*R*,5*R*)-1,4-bis(4-bromophenyl)-8-methyl-6-oxo-2-azaspiro[4.4]non-7-ene-3-carboxylate

white amorphous solid, 57% yield, **^{1}H NMR** (600 MHz, CD_2Cl_2) δ 7.50 (d, *J* = 8.4 Hz, 2H), 7.41 (d, *J* = 8.4 Hz, 2H), 7.13 (d, *J* = 8.4 Hz, 4H), 5.36 (s, 1H), 4.30 (s, 1H), 4.14 (d, *J* = 7.0 Hz, 1H), 3.75 (s, 3H), 3.73 (d, *J* = 7.0 Hz, 1H), 3.21 (s, 1H), 2.34-2.31 (m, 2H), 1.81 (s, 3H);

^{13}C NMR (151 MHz, CD_2Cl_2) δ 209.95, 176.94, 173.23, 140.54, 137.13, 132.42, 131.81, 131.14, 129.18, 128.99, 122.32, 121.55, 73.05, 67.66, 64.79, 55.57, 52.76, 42.62, 19.40;

HRMS: calcd. for $[M+H]^+$ $C_{23}H_{22}{}^{79}Br_2NO_3$ = 517.99610, found: 517.99637; calcd. for $[M+H]^+$ $C_{22}H_{20}{}^{79}Br^{81}BrNO_3$ = 519.99405, found: 519.99406.

FT-IR: $\tilde{\nu}$ 3558, 2508, 2025, 1987, 1756, 1548, 1234, 1198, 1081, 1036 cm^{-1}.

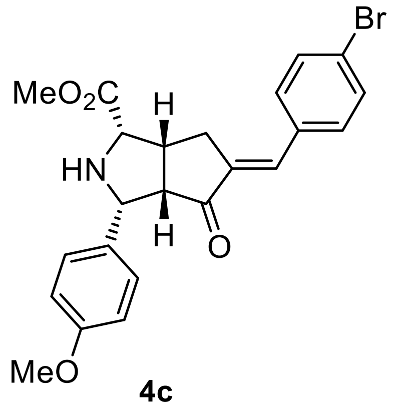

4c

Methyl (1*S*,3*R*,3a*S*,6a*R*)-5-((*E*)-4-bromobenzylidene)-3-(4-methoxyphenyl)-4-oxooctahydrocyclopenta[*c*]pyrrole-1-carboxylate

yellow amorphous solid, 61% yield, **^{1}H NMR** (500 MHz, CD_2Cl_2) δ 7.56 (d, *J* = 8.5 Hz, 2H), 7.38 (d, *J* = 8.5 Hz, 2H), 7.18 (d, *J* = 8.7 Hz, 2H), 6.99 (t, *J* = 3.0 Hz, 1H), 6.79 (d, *J* = 8.7 Hz, 2H), 4.51 (d, *J* = 10.5 Hz, 1H), 4.12 (d, *J* = 5.4 Hz, 1H), 3.83 (s, 3H), 3.76 (s, 3H), 3.27 – 3.21 (m, 1H), 3.16 – 3.08 (m, 2H), 2.79 (ddd, *J* = 18.1, 7.0, 3.4 Hz, 1H).

^{13}C NMR (126 MHz, CD_2Cl_2) δ 206.61, 172.31, 159.50, 138.30, 134.97, 132.67, 132.43, 131.54, 131.06, 129.23, 124.09, 113.79, 65.51, 64.49, 56.70, 55.62, 52.48, 41.07, 31.84.
HRMS: calcd. for $[M+H]^+$ $C_{23}H_{23}{}^{79}BrNO_4$ = 456.08010, found: 456.08050; calcd. for $[M+H]^+$ $C_{23}H_{23}{}^{81}BrNO_4$ = 458.07800, found: 458.07845;
HPLC conditions: CHIRALPAK IC column, *iso*-propanol/ *iso*-hexane = 50/50, flow rate = 0.5 mL min^{-1}, minor enantiomer: t_R = 66.88 min; major enantiomer: t_R = 49.92 min; (69% e.e.);
[α]$^{20}_{D}$= + 166.2° (c = 0.1, $CHCl_3$).
FT-IR: $\tilde{\nu}$ 2926, 2835, 1737, 1707, 1614, 1511, 1487, 1303, 1244, 1172, 1132, 1031 cm^{-1}.

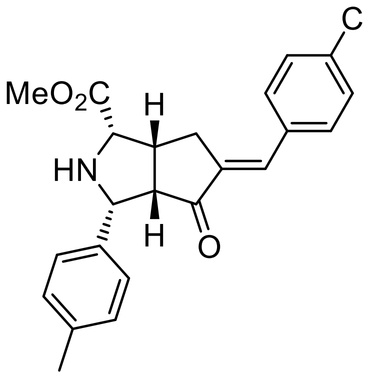

4d

Methyl (1*S*,3*R*,3a*S*,6a*R*)-5-((*E*)-4-chlorobenzylidene)-4-oxo-3-(*p*-tolyl)octahydrocyclopenta[*c*]pyrrole-1-carboxylate

white amorphous solid, 74% yield, **^{1}H NMR** (500 MHz, CD_2Cl_2) δ 7.48 – 7.37 (m, 4H), 7.15 (d, *J* = 7.9 Hz, 2H), 7.08 (d, *J* = 7.9 Hz, 2H), 7.02-6.99 (m, 1H), 4.52 (d, *J* = 10.4 Hz, 1H), 4.12 (d, *J* = 5.5 Hz, 1H), 3.83 (s, 3H), 3.29 – 3.08 (m, 3H), 2.86 – 2.78 (m, 1H), 2.30 (s, 3H);

^{13}C NMR (126 MHz, CD_2Cl_2) δ 206.47, 172.28, 138.13, 137.74, 136.52, 135.67, 134.57, 132.46, 130.99, 129.44, 129.24, 128.06, 65.88, 64.59, 56.73, 52.48, 41.14, 31.79, 21.39;

HRMS: calcd. for $[M+H]^+$ $C_{23}H_{23}{}^{35}ClNO_3$ = 396.13610, found: 396.13610; calcd. for $[M+H]^+$ $C_{23}H_{23}{}^{37}ClNO_3$ = 398.13316, found: 398.13315;

HPLC conditions: CHIRALPAK IC column, *iso*-propanol/ *iso*-hexane = 30/70, flow rate = 0.5 mL min^{-1}, minor enantiomer: t_R = 39.63 min; major enantiomer: t_R = 40.63 min; (84% e.e.);

$[\alpha]^{20}_{D}$= + 265.2° (c = 0.1, $CHCl_3$).

FT-IR: $\tilde{\nu}$ 3458, 2514, 2058, 1723, 1511, 1487, 1221, 1123, 1088, 1025 cm^{-1}.

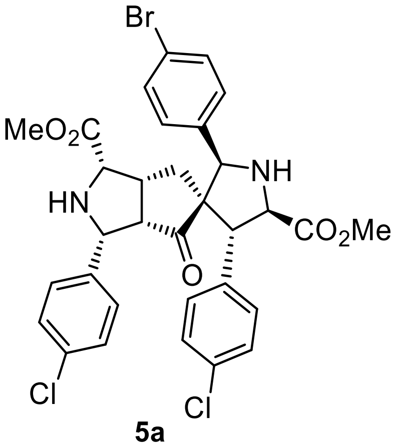

5a

Dimethyl (1*S*,2'*R*,3*R*,3a*S*,4'*R*,5*R*,5'*R*,6a*R*)-2'-(4-bromophenyl)-3,4'-bis(4-chlorophenyl)-4-oxohexahydro-1*H*-spiro[cyclopenta[*c*]pyrrole-5,3'-pyrrolidine]-1,5'-dicarboxylate

white amorphous solid, 77% yield, **^{1}H NMR** (400 MHz, CD_2Cl_2) δ 7.47 (d, *J* = 8.4 Hz, 2H), 7.38 (d, *J* = 8.5 Hz, 2H), 7.27 (d, *J* = 8.5 Hz, 2H), 7.13-7.11 (m, 4H), 7.02 (d, *J* = 8.4 Hz, 2H), 4.25 (s, 1H), 4.15 (d, *J* = 10.6 Hz, 1H), 3.95 (d, *J* = 7.2 Hz, 1H), 3.75 (d, *J* = 5.6 Hz, 1H), 3.65 (s, 3H), 3.59 (s, 3H), 3.45 (d, *J* = 7.2 Hz, 1H), 2.77 (s, 1H), 2.66 – 2.59 (m, 1H), 2.09 – 1.99 (m, 2H), 1.76 (m, 1H), 1.59 (*J* = 13.6, 10.6 Hz, 1H):

^{13}C NMR (101 MHz, CD_2Cl_2) δ 216.68, 173.29, 171.55, 138.48, 138.37, 137.98, 133.59, 133.38, 132.33, 131.11, 130.20, 129.71, 129.21, 128.49, 122.84, 74.76, 68.35, 67.05, 63.96, 63.11, 55.91, 55.28, 52.60, 52.24, 40.28, 34.50;

HRMS: calcd. for $[M+H]^+$ $C_{32}H_{29}{}^{79}Br^{35}Cl_2N_2O_5$ = 671.07191, found: 671.07097; calcd. for $[M+H]^+$ $C_{32}H_{29}{}^{81}Br^{35}Cl\ N_2O_5$ = 673.06915, found: 673.06892; calcd. for $[M+H]^+$ $C_{32}H_{29}{}^{81}Br^{37}Cl^{35}ClN_2O_5$ = 675.06689, found: 675.06597; **HPLC** conditions: CHIRALPAK IC column, *iso*-propanol/ *iso*-hexane = 40/60, flow rate = 0.5 mL min^{-1}, minor enantiomer: t_R = 15.28 min; major enantiomer: t_R = 21.02 min; (85% e.e.);

$[\alpha]^{20}_D$= + 65.6° (c = 0.2, $CHCl_3$).

FT-IR: $\tilde{\nu}$ 3423**,** 2528 ,2159, 2030, 1955, 1578, 1496, 1215, 1105, 1032 cm^{-1}.

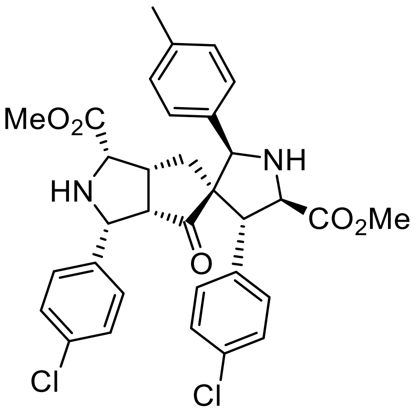

5b

Dimethyl (1*S*,2'*R*,3*R*,3*aS*,4'*R*,5*R*,5'*R*,6*aR*)-3,4'-bis(4-chlorophenyl)-4-oxo-2'-(*p*-tolyl)hexahydro-1*H*-spiro[cyclopenta[*c*]pyrrole-5,3'-pyrrolidine]-1,5'-dicarboxylate

white amorphous solid, 65% yield, **^{1}H NMR** (600 MHz, CD_2Cl_2) δ 7.38 (d, *J* = 8.4 Hz, 2H), 7.27 (d, *J* = 8.4 Hz, 2H), 7.15 – 7.11 (m, 6H), 7.03 (d, *J* = 8.4 Hz, 2H), 4.22 (s, 1H), 4.13 (d, *J* = 10.7 Hz, 1H), 3.98 (s, 1H), 3.95 (d, *J* = 7.1 Hz, 1H), 3.73 (d, *J* = 5.8 Hz, 1H), 3.65 (s, 3H), 3.60 (s, 3H), 3.42 (d, *J* = 7.1 Hz, 1H), 2.63 – 2.57 (m, 1H), 2.33 (s, 3H), 2.03 – 1.97 (m, 1H), 1.76 – 1.73 (m, 1H), 1.56 (dd, *J* = 13.4, 10.7 Hz, 1H);

^{13}C NMR (151 MHz, CD_2Cl_2) δ 217.14, 173.36, 171.66, 138.95, 138.90, 138.57, 135.22, 133.47, 133.33, 131.09, 129.88, 129.73, 129.17, 128.47, 128.21, 75.51, 68.47, 67.29, 63.92, 63.12, 55.96, 55.75, 52.56, 52.21, 40.36, 34.52, 21.41;

HRMS: calcd. for $[M+H]^+$ $C_{33}H_{32}{}^{35}Cl_2N_2O_5$ = 607.17639, found: 607.17610; calcd. for $[M+H]^+$ $C_{33}H_{32}{}^{35}Cl^{37}ClN_2O_5$ = 609.17367, found: 609.17315; calcd. for $[M+H]^+$ $C_{33}H_{32}{}^{37}Cl_2N_2O_5$ = 611.17036, found: 611.17020;

HPLC conditions: CHIRALPAK IC column, *iso*-propanol/ *iso*-hexane = 40/60, flow rate = 0.5 mL min^{-1}, minor enantiomer: t_R = 21.61 min; major enantiomer: t_R = 55.35 min; (82% e.e.);

$[\alpha]^{20}_D$= + 57.9° (c = 0.2, $CHCl_3$).

FT-IR: $\tilde{\nu}$ 3325, 2958, 2315, 2087, 1734, 1505, 1487, 1148, 1091, 1013, cm^{-1}.

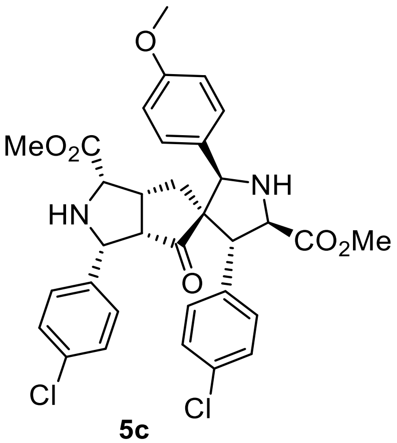

5c

Dimethyl (1*S*,2'*R*,3*R*,3a*S*,4'*R*,5*R*,5'*R*,6a*R*)-3,4'-bis(4-chlorophenyl)-2'-(4-methoxyphenyl)-4-oxohexahydro-1*H*-spiro[cyclopenta[*c*]pyrrole-5,3'-pyrrolidine]-1,5'-dicarboxylate

white amorphous solid, 55% yield, **^{1}H NMR** (600 MHz, CD_2Cl_2) δ 7.38 (d, *J* = 8.4 Hz, 2H), 7.27 (d, *J* = 8.4 Hz, 2H), 7.16 (d, *J* = 8.6 Hz, 2H), 7.12 (d, *J* = 8.4 Hz, 2H), 7.03 (d, *J* = 8.4 Hz, 2H), 6.85 (d, *J* = 8.6 Hz, 2H), 4.21 (s, 1H), 4.13 (d, *J* = 10.6 Hz, 1H), 3.94 (d, *J* = 7.1 Hz, 1H), 3.79 (s, 3H), 3.73 (d, *J* = 5.8 Hz, 1H), 3.64 (s, 3H), 3.60 (s, 3H), 3.42 (d, *J* = 7.1 Hz, 1H), 2.77 (bs, 1H), 2.64 – 2.58 (m, 1H), 1.99 (dd, *J* = 13.5, 10.6 Hz, 1H), 1.77– 1.71 (m, 1H), 1.55 (dd, *J* = 13.5, 10.6 Hz, 1H);

^{13}C NMR (151 MHz, CD_2Cl_2) δ 217.30, 173.38, 171.66, 160.24, 138.96, 138.59, 133.44, 133.33, 131.07, 130.11, 129.74, 129.48, 129.17, 128.47, 114.50, 75.29, 68.29, 67.26, 63.92, 63.14, 55.93, 55.77, 55.71, 52.55, 52.20, 40.35, 34.62;

HRMS: calcd. for $[M+H]^+$ $C_{33}H_{33}{}^{35}Cl_2N_2O_6$ = 623.18971 found: 623.18958; calcd. for $[M+H]^+$ $C_{33}H_{33}{}^{35}Cl^{37}ClN_2O_6$ = 625.26857 found: 625.26765;

HPLC conditions: CHIRALPAK IC column, *iso*-propanol/ *iso*-hexane = 40/60, flow rate = 0.5 mL min^{-1}, minor enantiomer: t_R = 20.96 min; major enantiomer: t_R = 26.49 min; (83% e.e.);

$[\alpha]^{20}_{D}$= + 123.8° (c = 0.2, $CHCl_3$).

FT-IR: $\tilde{\nu}$ 3496, 2475, 2437, 1827, 1754, 1542, 1228, 1165, 1102, 102 5cm^{-1}.

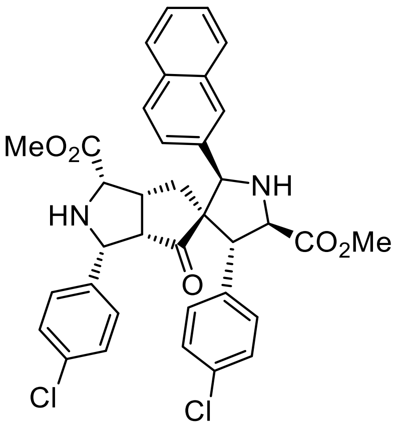

5d

Dimethyl (1*S*,2'*R*,3*R*,3a*S*,4'*R*,5*R*,5'*R*,6a*R*)-3,4'-bis(4-chlorophenyl)-2'-(naphthalen-2-yl)-4-oxohexahydro-1*H*-spiro[cyclopenta[*c*]pyrrole-5,3'-pyrrolidine]-1,5'-dicarboxylate

white amorphous solid, 63% yield, **^{1}H NMR** (600 MHz, CD_2Cl_2) δ 7.87 – 7.81 (m, 3H), 7.75 – 7.74 (m, 1H), 7.53 – 7.48 (m, 2H), 7.40 (d, *J* = 8.5 Hz, 2H), 7.36 (dd, *J* = 8.5, 1.9 Hz, 1H), 7.25 (d, *J* = 8.4 Hz, 2H), 7.16 (d, *J* = 8.4 Hz, 2H), 6.99 (d, *J* = 8.4 Hz, 2H), 4.45 (s, 1H), 4.07 – 4.02 (m, 2H), 3.68 – 3.65 (m, 4H), 3.63 (s, 3H), 3.53 (d, *J* = 7.3 Hz, 1H), 2.68 – 2.62 (m, 1H), 1.94 - 1.90 (m, 1H), 1.89 -1.85 (m, 1H), 1.64 – 1.54 (m, 2H);

^{13}C NMR (151 MHz, CD_2Cl_2) δ 216.83, 173.36, 171.62, 138.69, 138.45, 136.12, 133.84, 133.66, 133.54, 133.32, 131.17, 129.73, 129.20, 128.98, 128.59, 128.45, 128.18, 127.57, 126.97, 126.06, 75.72, 68.69, 67.28, 63.91, 63.09, 55.86, 55.69, 52.61, 52.23, 40.34, 34.64;

HRMS: calcd. for $[M+H]^+$ $C_{36}H_{32}{}^{35}Cl_2N_2O_5$ = 643.17790, found: 643.17610; calcd. for $[M+H]^+$ $C_{36}H_{32}{}^{35}Cl^{37}ClN_2O_5$ = 645.17539, found: 645.17315; calcd. for $[M+H]^+$ $C_{36}H_{32}{}^{37}Cl_2N_2O_5$ = 647.17179, found: 647.17020;

HPLC conditions: CHIRALPAK IC column, *iso*-propanol/ *iso*-hexane = 60/40, flow rate = 0.5 mL min^{-1}, minor enantiomer: t_R = 18.98 min; major enantiomer: t_R = 30.45 min; (89% e.e.);

$[\alpha]^{20}_D$ = + 102.7° (c = 0.22, $CHCl_3$).

FT-IR: $\tilde{\nu}$ 3415, 2348, 2109, 1735, 1732, 1496, 1211, 1172, 1097, 1048, cm^{-1}.

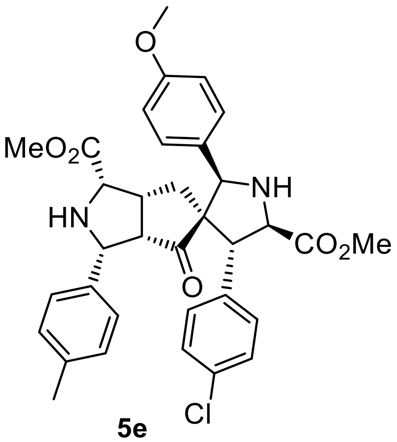

5e

Dimethyl (1*S*,2'*R*,3*R*,3a*S*,4'*R*,5*R*,5'*R*,6a*R*)-4'-(4-chlorophenyl)-2'-(4-methoxyphenyl)-4-oxo-3-(*p*-tolyl)hexahydro-1*H*-spiro[cyclopenta[*c*]pyrrole-5,3'-pyrrolidine]-1,5'-dicarboxylate

yellow amorphous solid, 70% yield, **^{1}H NMR** (600 MHz, CD_2Cl_2) δ 7.39 (d, *J* = 8.4 Hz, 2H), 7.15 (dd, *J* = 8.7 Hz, 2H), 7.14 (d, *J* = 8.4 Hz, 2H) 7.12 (d, *J* = 8.4 Hz, 2H), 6.92 (d, *J* = 8.7 Hz, 2H), 6.85 (d, *J* = 8.7 Hz, 2H), 4.19 (s, 1H), 4.10 (d, *J* = 10.7 Hz, 1H), 3.94 (d, *J* = 7.3 Hz, 1H), 3.80 (s, 3H), 3.70 (d, *J* = 5.8 Hz, 1H), 3.64 (s, 3H), 3.59 (s, 3H), 3.44 (d, *J* = 7.3 Hz, 1H), 2.61 – 2.56 (m, 1H), 2.36 (s, 3H), 1.98-1.96 (m, 1H), 1.73-1.69 (m, 1H), 1.55 (dd, *J* = 13.7, 10.7 Hz, 1H);

^{13}C NMR (151 MHz, CD_2Cl_2) δ 217.65, 173.30, 171.82, 160.23, 139.18, 137.69, 136.60, 133.40, 131.13, 129.98, 129.53, 129.16, 129.09, 128.25, 114.47, 75.50, 68.39, 67.54, 64.86, 63.24, 56.34, 55.93, 55.77, 52.49, 52.16, 40.76, 34.62, 21.46;

HRMS: calcd. for $[M+H]^+$ $C_{34}H_{35}{}^{35}ClN_2O_6$ = 603.22708, found: 603.22564; calcd. for $[M+H]^+$ $C_{34}H_{35}{}^{37}ClN_2O_6$ = 605.22458, found: 605.22269;

HPLC conditions: CHIRALPAK IC column, *iso*-propanol/ *iso*-hexane = 80/20, flow rate = 0.5 mL min^{-1}, minor enantiomer: t_R = 24.02 min; major enantiomer: t_R = 57.97 min; (89% e.e.);

$[\alpha]^{20}_{D}$= + 114.1° (c = 0.22, $CHCl_3$).

FT-IR: $\tilde{\nu}$ 3339, 2448, 2021, 1735, 1589, 1496, 1235, 1115, 1078, 1023 cm^{-1}.

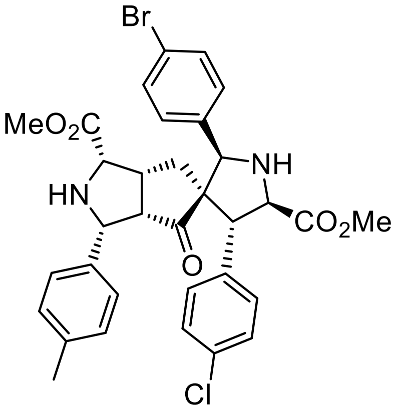

5f

Dimethyl (1*S*,2'*R*,3*R*,3a*S*,4'*R*,5*R*,5'*R*,6a*R*)-2'-(4-bromophenyl)-4'-(4-chlorophenyl)-4-oxo-3-(*p*-tolyl)hexahydro-1*H*-spiro[cyclopenta[*c*]pyrrole-5,3'-pyrrolidine]-1,5'-dicarboxylate

white amorphous solid, 65% yield, **^{1}H NMR** (400 MHz, CD_2Cl_2) δ 7.47 (d, *J* = 8.5 Hz, 2H), 7.39 (d, *J* = 8.5 Hz, 2H), 7.17 – 7.09 (m, 6H), 6.92 (d, *J* = 8.0 Hz, 2H), 4.23 (s, 1H), 4.12 (d, *J* = 10.6 Hz, 1H), 3.98 (d, *J* = 7.3 Hz, 1H), 3.72 (d, *J* = 5.7 Hz, 1H), 3.65 (s, 3H), 3.58 (s, 3H), 3.47 (d, *J* = 7.3 Hz, 1H), 2.78 (bs, 1H), 2.64 – 2.50 (m, 1H), 2.36 (s, 3H), 2.08 – 1.93 (m, 2H), 1.79 – 1.73 (m, 1H), 1.59 (dd, *J* = 13.7, 10.6 Hz, 1H);

^{13}C NMR (101 MHz, CD_2Cl_2) δ 217.00, 173.22, 171.72, 138.72, 137.86, 137.73, 136.40, 133.53, 132.29, 131.17, 130.26, 129.20, 129.10, 128.22, 122.82, 75.00, 68.45, 67.33, 64.89, 63.21, 56.30, 55.52, 52.53, 52.19, 40.66, 34.51, 21.46;

HRMS: calcd. for $[M+H]^+$ $C_{33}H_{32}{}^{79}Br^{35}ClN_2O_5$ = 651.12765, found: 651.12559; calcd. for $[M+H]^+$ $C_{33}H_{32}{}^{81}Br^{35}ClN_2O_5$ = 653.12541, found: 653.12354; calcd. for $[M+H]^+$ $C_{33}H_{32}{}^{81}Br^{37}ClN_2O_5$ = 655.12220, found: 655.12059;

HPLC conditions: CHIRALPAK IC column, *iso*-propanol/ *iso*-hexane = 30/70, flow rate = 0.5 mL min^{-1}, minor enantiomer: t_R = 24.85 min; major enantiomer: t_R = 55.27 min; (90% e.e.);

$[\alpha]^{20}_D$= + 78.5° (c = 0.2, $CHCl_3$).

FT-IR: $\tilde{\nu}$ 3520, 2485, 2415, 1871, 1745, 1663, 1453, 1295, 1081, 1022 cm^{-1}.

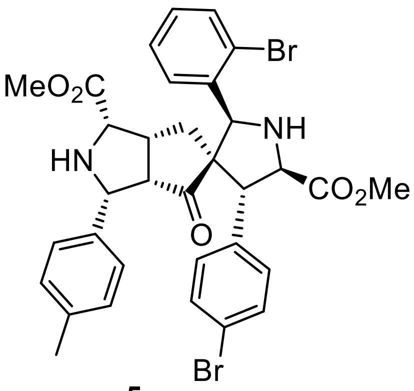

5g

Dimethyl (1*S*,2'*S*,3*R*,3a*S*,4'*R*,5*R*,5'*R*,6a*R*)-2'-(2-bromophenyl)-4'-(4-bromophenyl)-4-oxo-3-(*p*-tolyl)hexahydro-1*H*-spiro[cyclopenta[*c*]pyrrole-5,3'-pyrrolidine]-1,5'-dicarboxylate

white amorphous solid, 55% yield, **^{1}H NMR** (500 MHz, CD_2Cl_2) δ 7.60 (dd, *J* = 8.0, 1.3 Hz, 1H), 7.55 (d, *J* = 8.5 Hz, 2H), 7.37 – 7.27 (m, 2H), 7.24 – 7.15 (m, 2H), 7.11 (d, *J* = 8.5 Hz, 3H), 6.89 (d, *J* = 8.0 Hz, 2H), 4.96 (s, 1H), 4.13 (d, *J* = 10.7 Hz, 1H), 3.99-3.97 (m, 2H), 3.73 (d, *J* = 5.7 Hz, 1H), 3.66 (s, 3H), 3.59 (s, 3H), 3.47 (d, *J* = 7.7 Hz, 1H), 2.81 – 2.73 (m, 1H), 2.36 (s, 3H), 2.15 – 1.99 (m, 1H), 1.59 (dd, *J* = 13.6, 10.7 Hz , 1H);

^{13}C NMR (176 MHz, CD_2Cl_2) δ 216.54, 172.59, 171.18, 138.74, 137.36, 137.15, 135.93, 133.16, 131.63, 131.04, 129.58, 129.47, 128.55, 127.98, 127.66, 124.65, 121.10, 70.77, 68.38, 66.78, 64.37, 62.57, 55.87, 55.03, 51.97, 51.59, 40.26, 33.26, 20.89;

HRMS: calcd. for $[M+H]^+$ $C_{33}H_{32}{}^{79}Br_2N_2O_5$ = 695.07740, found: 695.07507; calcd. for $[M+H]^+$ $C_{33}H_{32}{}^{79}Br^{81}BrN_2O_6$ = 697.07539, found: 697.07303; calcd. for $[M+H]^+$ $C_{33}H_{32}{}^{81}Br_2N_2O_6$ = 699.07348, found: 699.07098;

HPLC conditions: CHIRALPAK IA column, *iso*-propanol/ *iso*-hexane = 15/85, flow rate = 0.5 mL min^{-1}, minor enantiomer: t_R = 41.58 min; major enantiomer: t_R = 45.36 min; (90% e.e.);

$[\alpha]^{20}_D$= + 44.6° (c = 0.18, $CHCl_3$).

FT-IR: $\tilde{\nu}$ 3368, 2360, 2355, 1732, 1492, 1434, 1207, 1152, 1090, 1010, cm^{-1}.

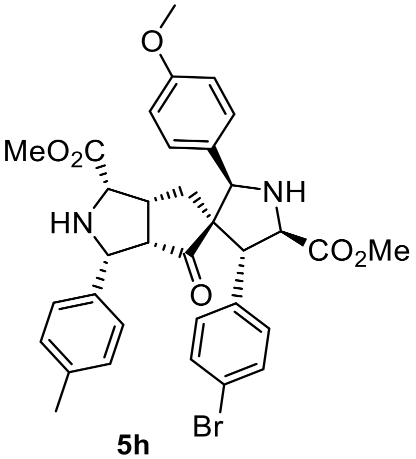

5h

Dimethyl (1*S*,2'*R*,3*R*,3a*S*,4'*R*,5*R*,5'*R*,6a*R*)-4'-(4-bromophenyl)-2'-(4-methoxyphenyl)-4-oxo-3-(*p*-tolyl)hexahydro-1*H*-spiro[cyclopenta[*c*]pyrrole-5,3'-pyrrolidine]-1,5'-dicarboxylate

white amorphous solid, 59% yield, **^{1}H NMR** (400 MHz, CD_2Cl_2) δ 7.54 (d, *J* = 8.7 Hz, 2H), 7.15 (d, *J* = 8.7 Hz, 2H), 7.12-7.09 (m, 4H), 6.92 (d, *J* = 8.8 Hz, 2H), 6.85 (d, *J* = 8.8 Hz, 2H), 4.19 (s, 1H), 4.10 (d, *J* = 10.7 Hz, 1H), 3.93 (d, *J* = 7.3 Hz, 1H), 3.80 (s, 3H), 3.70 (d, *J* = 5.7 Hz, 1H), 3.64 (s, 3H), 3.59 (s, 3H), 3.42 (d, *J* = 7.3 Hz, 1H), 2.78 (bs, 1H), 2.63 – 2.54 (m, 1H), 2.36 (s, 3H), 2.03 – 1.94 (m, 1H), 1.73 (m, 1H), 1.56 (dd, *J* = 13.7, 10.7 Hz, 1H);

^{13}C NMR (101 MHz, CD_2Cl_2) δ 217.61, 173.29, 171.82, 160.24, 139.70, 137.69, 136.61, 132.14, 131.50, 130.01, 129.53, 129.09, 128.25, 121.53, 114.48, 75.53, 68.35, 67.50, 64.86, 63.25, 56.34, 56.00, 55.77, 52.49, 52.17, 40.78, 34.62, 21.46;

HRMS: calcd. for $[M+H]^+$ $C_{34}H_{35}{}^{79}BrN_2O_6$ = 647.17683, found: 647.17513; calcd. for $[M+H]^+$ $C_{34}H_{35}{}^{81}BrN_2O_6$ = 649.17514, found: 649.17308;

HPLC conditions: CHIRALPAK IA column, *iso*-propanol/ *iso*-hexane = 20/80, flow rate = 0.5 mL min^{-1}, minor enantiomer: t_R = 53.22 min; major enantiomer: t_R = 47.58 min; (89% e.e.);

$[\alpha]^{20}_D$= + 127.5° (c = 0.2, $CHCl_3$).

FT-IR: $\tilde{\nu}$ 3652, 2534 ,2159, 2030, 1976, 1733, 1513, 1489, 1434, 1205, 1121, 1074, 1010 cm^{-1}.

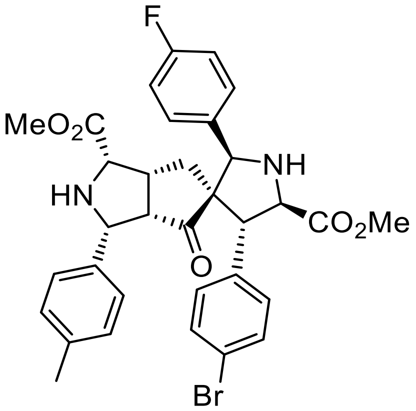

5i

Dimethyl (1*S*,2'*R*,3*R*,3a*S*,4'*R*,5*R*,5'*R*,6a*R*)-4'-(4-bromophenyl)-2'-(4-fluorophenyl)-4-oxo-3-(*p*-tolyl)hexahydro-1*H*-spiro[cyclopenta[*c*]pyrrole-5,3'-pyrrolidine]-1,5'-dicarboxylate

white amorphous solid, 78% yield, **^{1}H NMR** (600 MHz, CD_2Cl_2) δ 7.54 (d, *J* = 8.4 Hz, 2H), 7.24 (dd, *J* = 8.4, 2H), 7.12 – 7.08 (m, 4H), 7.03 (d, *J* = 8.1 Hz, 2H), 6.92 (d, *J* = 8.1 Hz, 2H), 4.25 (s, 1H), 4.11 (d, *J* = 10.7 Hz, 1H), 3.96 (d, *J* = 7.3 Hz, 1H), 3.71 (d, *J* = 5.7 Hz, 1H), 3.65 (s, 3H), 3.58 (s, 3H), 3.45 (d, *J* = 7.3 Hz, 1H), 2.77 (bs, 1H), 2.62 – 2.55 (m, 1H), 2.36 (s, 3H), 2.03 (bs, 1H), 2.01 – 1.95 (m, 1H), 1.78 – 1.74 (m, 1H), 1.59 (dd, *J* = 13.5, 10.7 Hz, 1H);

^{13}C NMR (151 MHz, CD_2Cl_2) δ 216.63, 172.67, 171.17, 163.45 (d, *J* = 246.7 Hz), 138.78, 137.16, 135.87, 133.87 (d, *J* = 3.1 Hz), 131.60, 130.95, 129.67 (d, *J* = 8.1 Hz), 128.53, 127.66, 121.06, 115.55 (d, *J* = 21.5 Hz), 74.45, 67.74, 66.73, 64.30, 62.66, 55.72, 55.09, 51.96, 51.64, 40.10, 33.98, 20.89;

^{19}F NMR (565 MHz, CD_2Cl_2) δ -114.2 (m);

HRMS: calcd. for $[M+H]^+$ $C_{33}H_{33}{}^{79}BrFN_2O_5$ = 635.15514, found: 635.15578; calcd. for $[M+H]^+$ $C_{33}H_{33}{}^{81}BrFN_2O_5$= 637.15309, found: 637.15414;

HPLC conditions: CHIRALPAK IC column, *iso*-propanol/ *iso*-hexane = 30/70, flow rate = 0.5 mL min^{-1}, minor enantiomer: t_R = 18.50 min; major enantiomer: t_R = 44.05 min; (90% e.e.);

$[\alpha]^{20}_{D}$= + 115.5° (c = 0.2, $CHCl_3$).

FT-IR: $\tilde{\nu}$ 3448, 2515, 2159, 2030, 1732, 1513, 1435, 1248, 1183, 1031 cm^{-1}.

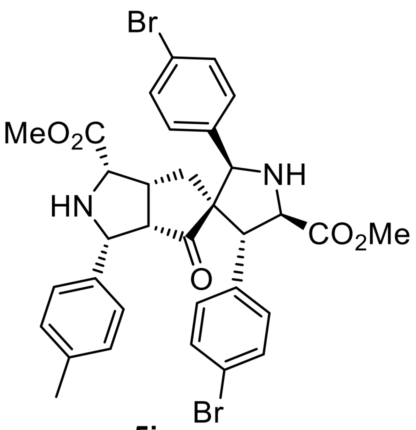

5j

Dimethyl (1*S*,2'*R*,3*R*,3a*S*,4'*R*,5*R*,5'*R*,6a*R*)-2',4'-bis(4-bromophenyl)-4-oxo-3-(*p*-tolyl)hexahydro-1*H*-spiro[cyclopenta[*c*]pyrrole-5,3'-pyrrolidine]-1,5'-dicarboxylate

white amorphous solid, 72% yield, **^{1}H NMR** (700 MHz, CD_2Cl_2) δ 7.54 (d, *J* = 8.4 Hz, 2H), 7.46 (d, *J* = 8.4 Hz, 2H), 7.15 (d, *J* = 8.0 Hz, 2H), 7.12-7.08 (m, 4H), 6.92 (d, *J* = 8.0 Hz, 2H), 4.23 (s, 1H), 4.12 (d, *J* = 10.8 Hz, 1H), 3.99 (s, 1H), 3.97 (d, *J* = 7.3 Hz, 1H), 3.72 (d, *J* = 5.8 Hz, 1H), 3.65 (s, 3H), 3.58 (s, 3H), 3.45 (d, *J* = 7.3 Hz, 1H), 2.62 – 2.57 (m, 1H), 2.36 (s, 3H), 2.03 – 1.98 (m, 1H), 1.78 – 1.74 (m, 1H), 1.59 (dd, *J* = 13.6, 10.8 Hz, 1H);

^{13}C NMR (176 MHz, CD_2Cl_2) δ 216.98, 173.18, 171.71, 139.21, 137.80, 137.73, 136.39, 132.30, 132.18, 131.52, 130.25, 129.11, 128.22, 122.84, 121.67, 74.97, 68.39, 67.26, 64.89, 63.22, 56.30, 55.56, 52.55, 52.21, 40.67, 34.48, 21.46;

HRMS: calcd. for $[M+H]^+$ $C_{33}H_{32}{}^{79}Br_2N_2O_5$ = 695.07740, found: 695.07507; calcd. for $[M+H]^+$ $C_{33}H_{32}{}^{79}Br^{81}BrN_2O_5$ = 697.06780, found: 697.07531; calcd. for $[M+H]^+$ $C_{33}H_{32}{}^{81}Br_2$N2O5 = 699.07358, found: 699.07098;

HPLC conditions: CHIRALPAK IC column, *iso*-propanol/ *iso*-hexane = 30/70, flow rate = 0.5 mL min^{-1}, minor enantiomer: t_R = 25.90 min; major enantiomer: t_R = 58.02 min; (87% e.e.);

$[\alpha]^{20}_D$= + 74.7° (c = 0.36, $CHCl_3$).

FT-IR: $\tilde{\nu}$ 3378, 2514, 2159, 2058, 1733, 1515, 1455, 1221, 1109, 1015 cm^{-1}.

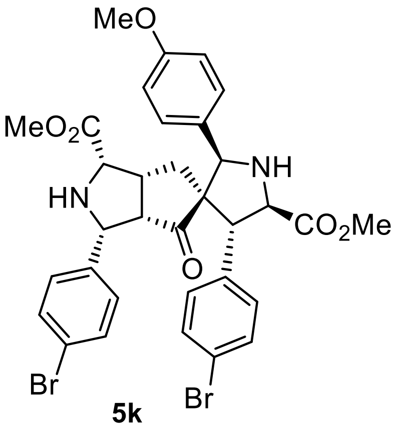

5k

Dimethyl (1*S*,2'*R*,3*R*,3*aS*,4'*R*,5*R*,5'*R*,6*aR*)-3,4'-bis(4-bromophenyl)-2'-(4-methoxyphenyl)-4-oxohexahydro-1*H*-spiro[cyclopenta[*c*]pyrrole-5,3'-pyrrolidine]-1,5'-dicarboxylatewhite amorphous solid, 37% yield, ^{1}H NMR (600 MHz, $CDCl_3$) δ 7.46 (d, *J* = 7.4 Hz, 2H), 7.36 (d, *J* = 8.4 Hz, 2H), 7.07-7.10 (m, 4H), 6.99 (d, *J* = 8.4 Hz, 2H), 6.78 (d, *J* = 7.6 Hz, 2H), 4.28 (s, 1H), 4.13 (d, *J* = 10.8 Hz, 1H), 4.06 – 4.03 (m, 1H), 3.74 (s, 3H), 3.69 (d, *J* = 5.5 Hz, 1H), 3.61 (s, 3H), 3.59 (s, 3H), 3.34 (d, *J* = 4.8 Hz, 1H), 2.41-2.48 (m, 1H), 2.09 – 2.06 (m, 1H), 1.63 – 1.56 (m, 1H).

^{13}C NMR (151 MHz, $CDCl_3$) δ 217.46, 172.26, 171.15, 160.11, 138.29, 138.07, 132.09, 131.30, 130.75, 129.38, 129.12, 121.74, 121.47, 114.54, 67.09, 66.11, 63.48, 62.80, 55.98, 55.45, 52.88, 52.20, 39.92, 32.07, 29.85.

HRMS: calcd. for $[M+H]^+$ $C_{33}H_{33}{}^{79}Br_2N_2O_6$ = 711.06998, found: 711.06999; calcd. for $[M+H]^+$ $C_{33}H_{33}{}^{79}Br^{81}BrN_2O_6$ = 713.06794, found: 713.06794; calcd. for $[M+H]^+$ $C_{33}H_{33}{}^{81}Br_2N_2O_6$ = 715.06616, found: 715.06590;

HPLC conditions: CHIRALPAK IA column, *iso*-propanol/ *iso*-hexane = 40/60, flow rate = 0.5 mL min^{-1}, minor enantiomer: t_R = 25.78 min; major enantiomer: t_R = 33.09 min; (33% e.e.); (through Imin exchange reaction, see experimental studies).

FT-IR: $\tilde{\nu}$ 3344, 2922, 2851, 1731, 1611, 1514, 1488, 1435, 1408, 1284, 1207, 1073, 964 cm^{-1}.

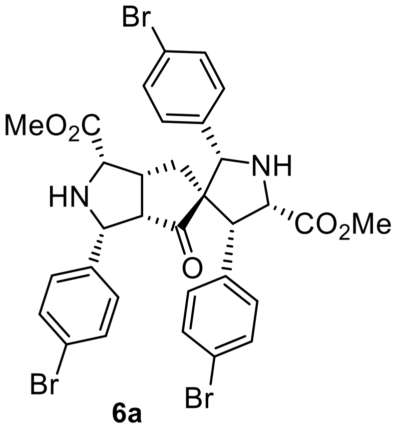

6a

Dimethyl (1*S*,2'*S*,3*R*,3*aS*,4'*R*,5*R*,5'*S*,6*aR*)-2',3,4'-tris(4-bromophenyl)-4-oxohexahydro-1*H*-spiro[cyclopenta[*c*]pyrrole-5,3'-pyrrolidine]-1,5'-dicarboxylate

white amorphous solid, 65% yield, **^{1}H NMR** (600 MHz, $CDCl_3$) δ 7.52 (d, *J* = 7.7 Hz, 2H), 7.45 (d, *J* = 8.4 Hz, 2H), 7.37 (d, *J* = 7.8 Hz, 2H), 7.28 (d, *J* = 7.8 Hz, 2H), 7.00 (d, *J* = 8.4 Hz, 2H), 6.83 (d, *J* = 8.0 Hz, 2H), 4.20 (d, *J* = 10.0 Hz, 2H), 4.04 (s, 1H), 3.76 (d, *J* = 9.3 Hz, 1H), 3.63 (s, 3H), 3.36 (s, 3H), 2.68-2.61 (m, 1H), 1.93 (dd, *J* = 13.9, 7.5 Hz, 1H), 1.65-1.62 (m, 1H), 1.60-1.53 (m, 2H).

^{13}C NMR (151 MHz, $CDCl_3$) δ 219.28, 172.27, 170.98, 137.18, 136.61, 131.96, 131.89, 131.50, 131.35, 131.33, 129.28, 129.20, 122.77, 121.67, 121.50, 70.86, 66.53, 64.34, 63.77, 63.20, 55.56, 54.28, 52.03, 51.87, 39.62, 30.71.

HRMS: calcd. for $[M+H]^+$ $C_{32}H_{30}{}^{79}Br_3N_2O_5$ = 758.97018, found: 758.96994; calcd. for $[M+H]^+$ $C_{32}H_{30}{}^{79}Br^{81}Br_2N_2O_5$ = 762.96627, found: 762.96584;

HPLC conditions: CHIRALPAK IC column, *iso*-propanol/ *iso*-hexane = 30/70, flow rate = 0.5 mL min^{-1}, minor enantiomer: t_R = 19.89 min; major enantiomer: t_R = 29.13 min; (95% e.e.); CHIRALPAK IA column, *iso*-propanol/ *iso*-hexane = 15/85, flow rate = 0.5 mL min^{-1}, minor enantiomer: t_R = 72.52 min; major enantiomer: t_R = 90.37 min; (95% e.e.);

$[\alpha]^{20}_D$= + 187.6° (c = 0.2, $CHCl_3$).

FT-IR: $\tilde{\nu}$ 3329, 2921, 2851, 1738, 1724, 1485, 1441, 1367, 1206, 1125, 1072, 1009 cm^{-1}.

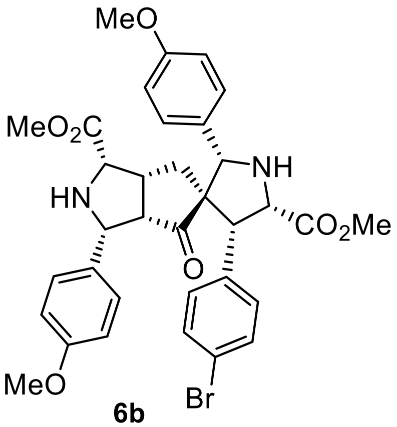

6b

Dimethyl (1*S*,2'*S*,3*R*,3*aS*,4'*R*,5*R*,5'*S*,6*aR*)-4'-(4-bromophenyl)-2',3-bis(4-methoxyphenyl)-4-oxohexahydro-1*H*-spiro[cyclopenta[*c*]pyrrole-5,3'-pyrrolidine]-1,5'-dicarboxylate

white amorphous solid, 60% yield, **^{1}H NMR** (600 MHz, CD_2Cl_2) δ 7.47 (d, *J* = 8.4 Hz, 2H), 7.32 (d, *J* = 8.1 Hz, 2H), 7.11 (d, *J* = 8.5 Hz, 2H), 6.92 (d, *J* = 8.9 Hz, 2H), 6.90 – 6.83 (m, 2H), 6.80 (d, *J* = 8.7 Hz, 2H), 4.16 (dd, *J* = 12.0, 9.8 Hz, 2H), 4.02 (s, 1H), 3.84 (s, 3H), 3.83 (s, 3H), 3.71 (d, *J* = 9.2 Hz, 1H), 3.57 (s, 3H), 3.32 (s, 3H), 2.65 – 2.60 (m, 2H), 1.84 (ddd, *J* = 13.9, 7.8, 1.2 Hz, 1H), 1.35 – 1.26 (m, 2H).

^{13}C NMR (151 MHz, CD_2Cl_2) δ 220.61, 172.58, 171.75, 160.35, 159.42, 138.29, 132.46, 131.51, 131.32, 130.33, 129.30, 129.15, 121.56, 114.29, 113.73, 71.42, 66.95, 65.16, 64.58, 63.55, 56.41, 55.83, 55.72, 54.67, 52.03, 51.88, 40.45, 31.57.

HRMS: calcd. for $[M+H]^+$ $C_{34}H_{36}{}^{79}BrN_2O_7$ = 663.16971, found: 663.17004; calcd. for $[M+H]^+$ $C_{34}H_{36}{}^{81}BrN_2O_7$ = 665.16804, found: 665.16799;

HPLC conditions: CHIRALPAK IA column, *iso*-propanol/ *iso*-hexane = 80/20, flow rate = 0.5 mL min^{-1}, minor enantiomer: t_R = 36.50 min; major enantiomer: t_R = 64.39 min; (93% e.e.);

$[\alpha]^{20}_{D}$= + 197.6° (c = 0.2, $CHCl_3$).

FT-IR: $\tilde{\nu}$ 2923, 2851, 1733, 1567, 1508, 1489, 1210, 1154 ,1075, 1037, 1009, 965 cm^{-1}.

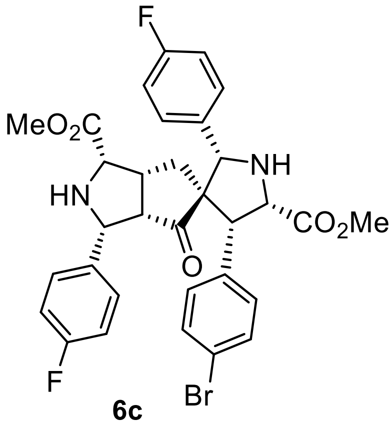

6c

Dimethyl (1S,2'S,3R,3aS,4'R,5R,5'S,6aR)-4'-(4-bromophenyl)-2',3-bis(4-fluorophenyl)-4-oxohexahydro-1H-spiro[cyclopenta[c]pyrrole-5,3'-pyrrolidine]-1,5'-dicarboxylate

white amorphous solid, 51% yield, **^{1}H NMR** (600 MHz, $CDCl_3$) δ 7.46 (d, *J* = 8.4 Hz, 2H), 7.41 – 7.37 (m, 2H), 7.09 (t, *J* = 8.2 Hz, 2H), 7.03 (d, *J* = 8.4 Hz, 2H), 6.97 – 6.90 (m, 4H), 4.25-4.20 (m, 2H), 4.08 (s, 1H), 3.76 (d, *J* = 9.4 Hz, 1H), 3.63 (s, 3H), 3.36 (s, 3H), 2.66-2.62 (m, 1H), 1.92 (dd, *J* = 13.7, 7.4 Hz, 1H), 1.56-150 (m, 1H), 1.46 – 1.41 (m, 1H), 1.28-1.23 (m, 1H).

^{13}C NMR (151 MHz, $CDCl_3$) δ 219.47, 172.24, 170.99, 162.99 (d, *J* = 248.0 Hz), 162.22 (d, *J* = 246.0 Hz), 136.73, 133.74, 133.20, 131.90, 131.77, 131.35, 129.14 (d, *J* = 7.8 Hz), 121.64, 115.80 (d, *J* = 21.3 Hz), 115.14 (d, *J* = 21.3 Hz), 71.55, 70.72, 66.54, 64.31, 63.80, 63.12, 55.63, 54.19, 52.02, 39.66, 30.77.

HRMS: calcd. for $[M+H]^+$ $C_{32}H_{30}{}^{79}BrF_2N_2O_5$ = 639.13007, found: 639.13014; calcd. for $[M+H]^+$ $C_{32}H_{30}{}^{81}BrF_2N_2O_5$ = 641.12818, found: 641.12811.

HPLC conditions: CHIRALPAK IC column, *iso*-propanol/ *iso*-hexane = 30/70, flow rate = 0.5 mL min^{-1}, minor enantiomer: t_R = 17.01 min; major enantiomer: t_R = 21.26 min; (97% e.e.);

$[\alpha]^{20}_D$= + 166.6° (c = 0.2, $CHCl_3$).

FT-IR: $\tilde{\nu}$ 3337, 2850, 1729, 1605, 1509, 1486, 1437, 1375, 1222, 1156 , 1128, 1076, 1037, 1030, 1012 cm^{-1}.

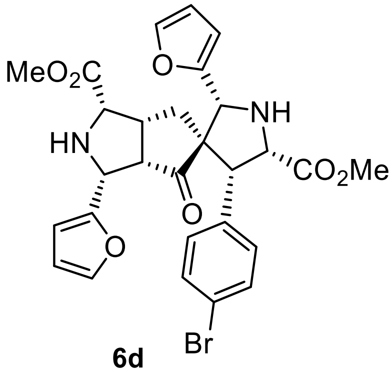

6d

Dimethyl (1*S*,2'*R*,3*R*,3*aS*,4'*R*,5*R*,5'*S*,6*aR*)-4'-(4-bromophenyl)-2',3-di(furan-2-yl)-4-oxohexahydro-1*H*-spiro[cyclopenta[*c*]pyrrole-5,3'-pyrrolidine]-1,5'-dicarboxylate

white amorphous solid, 49% yield, **^{1}H NMR** (700 MHz, $CDCl_3$) δ 7.46 – 7.41 (m, 3H), 7.29 (dd, *J* = 1.8, 0.8 Hz, 1H), 7.15 (d, *J* = 8.5 Hz, 2H), 6.44 – 6.41 (m, 2H), 6.32 (dd, *J* = 3.2, 1.9 Hz, 1H), 6.11 – 6.09 (m, 1H), 4.46 (d, *J* = 11.0 Hz, 1H), 4.30 (d, *J* = 8.7 Hz, 1H), 4.24 (s, 1H), 3.76 (d, *J* = 6.0 Hz, 1H), 3.72 (d, *J* = 8.7 Hz, 1H), 3.63 (s, 3H), 3.36 (s, 3H), 3.01 – 2.97 (m, 1H), 2.13 – 2.08 (m, 1H), 1.77 (ddd, *J* = 13.9, 7.6, 1.5 Hz, 1H), 1.39 (dd, *J* = 13.9, 11.0 Hz, 1H).

^{13}C NMR (176 MHz, $CDCl_3$) δ 218.91, 171.86, 170.59, 151.69, 150.43, 142.49, 142.30, 136.95, 131.83, 131.23, 121.52, 111.07, 110.76, 108.92, 108.69, 66.46, 64.52, 64.39, 63.50, 59.06, 55.66, 54.67, 52.20, 51.85, 41.63, 30.72.

HRMS: calcd. for $[M+H]^+$ $C_{28}H_{28}{}^{79}BrN_2O_7$ = 583.10744, found: 583.10718; calcd. for $[M+H]^+$ $C_{28}H_{28}{}^{81}BrN_2O_7$ = 585.10539, found: 585.10522;

HPLC conditions: CHIRALPAK IC column, *iso*-propanol/ *iso*-hexane = 50/50, flow rate = 0.5 mL min^{-1}, minor enantiomer: t_R = 69.21 min; major enantiomer: t_R = 29.62 min; (95% e.e.);

$[\alpha]^{20}_{D}$= + 197.6° (c = 0.2, $CHCl_3$).

FT-IR: $\tilde{\nu}$ 2951, 2159, 2034, 1730, 1505, 1485, 1435, 1363, 1205 ,1182, 1148, 1119, 1072, 953 cm^{-1}.

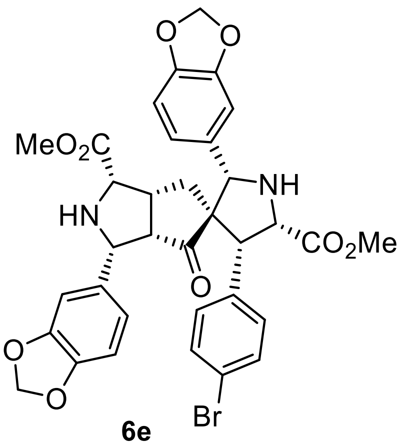

6e

Dimethyl (1*S*,2'*S*,3*R*,3*aS*,4'*R*,5*R*,5'*S*,6*aR*)-2',3-bis(benzo[*d*][1,3]dioxol-5-yl)-4'-(4-bromophenyl)-4-oxohexahydro-1*H*-spiro[cyclopenta[*c*]pyrrole-5,3'-pyrrolidine]-1,5'-dicarboxylate

white amorphous solid, 48% yield, **^{1}H NMR** (700 MHz, $CDCl_3$) δ 7.44 (d, *J* = 8.5 Hz, 2H), 7.10 (d, *J* = 8.5 Hz, 2H), 6.94 (s, 1H), 6.81 – 6.72 (m, 3H), 6.53 (dd, *J* = 8.0, 1.7 Hz, 1H), 6.47 (d, *J* = 1.7 Hz, 1H), 6.05 – 5.96 (m, 4H), 4.19 (dd, *J* = 10.0, 8.8 Hz, 2H), 4.00 (s, 1H), 3.74 (d, *J* = 9.2 Hz, 1H), 3.62 (d, *J* = 6.0 Hz, 1H), 3.59 (s, 3H), 3.32 (s, 3H), 2.71 – 2.65 (m, 1H), 1.88 (dd, *J* = 14.6, 8.4 Hz, 1H), 1.73 – 1.65 (m, 1H), 1.31 – 1.28 (m, 1H).

^{13}C NMR (176 MHz, $CDCl_3$) δ 220.39, 172.01, 171.15, 148.05, 147.90, 147.67, 147.10, 137.53, 132.33, 131.81, 131.35, 130.87, 121.41, 120.95, 120.90, 108.42, 108.12, 108.02, 107.99, 101.43, 101.23, 71.29, 66.57, 64.91, 64.17, 63.15, 55.99, 54.02, 51.91, 51.70, 40.00, 31.59.

HRMS: calcd. for $[M+H]^+$ $C_{34}H_{32}{}^{79}BrN_2O_9$ = 691.12857, found: 691.12859; calcd. for $[M+H]^+$ $C_{34}H_{32}{}^{81}BrN_2O_9$ = 693.12652, found: 693.12693;

HPLC conditions: CHIRALPAK IC column, *iso*-propanol/ *iso*-hexane = 80/20, flow rate = 0.5 mL min^{-1}, minor enantiomer: t_R = 30.53 min; major enantiomer: t_R = 59.65 min; (95% e.e.);

$[\alpha]^{20}_D$= + 147.6° (c = 0.2, $CHCl_3$).

FT-IR: $\tilde{\nu}$ 2886, 2160, 2035, 1727, 1502, 1485, 1442, 1388, 1238, 1205, 1118, 1035, 1010, 931 cm^{-1}.

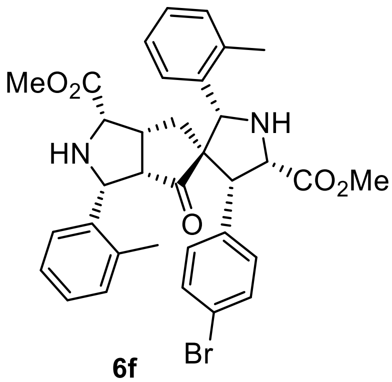

6f

Dimethyl (1*S*,2'*S*,3*R*,3*aS*,4'*R*,5*R*,5'*S*,6*aR*)-4'-(4-bromophenyl)-4-oxo-2',3-di-*o*-tolylhexahydro-1*H*-spiro[cyclopenta[*c*]pyrrole-5,3'-pyrrolidine]-1,5'-dicarboxylate

white amorphous solid, 46% yield, **^{1}H NMR** (700 MHz, $CDCl_3$) δ 7.80 – 7.74 (m, 1H), 7.36 (d, *J* = 8.4 Hz, 2H), 7.28 – 7.24 (m, 1H), 7.12 (dd, *J* = 4.0, 1.4 Hz, 2H), 7.09 (d, *J* = 7.5 Hz, 1H), 7.05 – 7.00 (m, 1H), 6.99 (d, *J* = 8.5 Hz, 2H), 6.89 (d, *J* = 7.6 Hz, 1H), 4.37 (s, 1H), 4.34 (d, *J* = 10.8 Hz, 1H), 4.11 (d, *J* = 8.9 Hz, 1H), 3.57 (d, *J* = 5.7 Hz, 1H), 3.53 (s, 3H), 3.48 (d, *J* = 8.5 Hz, 1H), 3.26 (s, 3H), 2.79-2.76 (m, 1H), 2.29 (s, 3H), 2.12 (s, 3H), 1.86 (dd, *J* = 14.0, 7.5 Hz, 1H), 1.63 – 1.58 (m, 1H), 1.29 – 1.24 (m, 1H).

^{13}C NMR (176 MHz, $CDCl_3$) δ 220.14, 172.07, 171.09, 136.89, 136.40, 135.80, 131.97, 131.84, 131.25, 131.21, 130.81, 130.08, 128.26, 128.21, 127.45, 126.38, 126.14, 125.18, 121.36, 65.97, 65.37, 64.08, 62.94, 61.41, 54.43, 53.77, 51.91, 51.73, 40.34, 31.24, 19.86, 19.57.

HRMS: calcd. for $[M+H]^+$ $C_{34}H_{36}{}^{79}BrN_2O_5$ = 631.18021, found: 631.18000; calcd. for $[M+H]^+$ $C_{34}H_{36}{}^{81}BrN_2O_5$ = 633.17816, found: 633.17805;

HPLC conditions: CHIRALPAK IC column, *iso*-propanol/ *iso*-hexane = 80/20, flow rate = 0.5 mL min^{-1}, minor enantiomer: t_R = 28.40 min; major enantiomer: t_R = 22.09 min; (89% e.e.);

$[\alpha]^{20}_{D}$= + 199.7° (c = 0.2, $CHCl_3$).

FT-IR: $\tilde{\nu}$ 3341, 2950, 1731, 1698, 1681, 1486, 1434, 1376, 1209 ,1073, 1036, 1029, 1011 cm^{-1}.

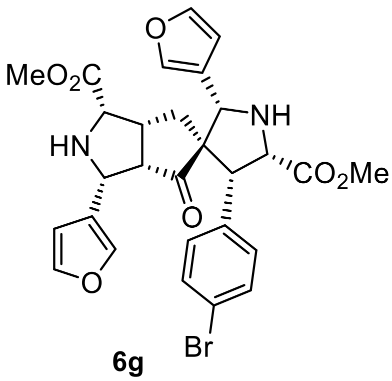

6g

Dimethyl (1S,2'S,3R,3aS,4'R,5R,5'S,6aR)-4'-(4-bromophenyl)-2',3-di(furan-3-yl)-4-oxohexahydro-1H-spiro[cyclopenta[c]pyrrole-5,3'-pyrrolidine]-1,5'-dicarboxylate

white amorphous solid, 52% yield, ^{1}**H NMR** (700 MHz, $CDCl_3$) δ 7.48 – 7.47 (m, 2H), 7.40 (d, *J* = 8.5 Hz, 2H), 7.36 (t, *J* = 1.7 Hz, 1H), 6.94 – 6.92 (m, 3H), 6.45 (dd, *J* = 1.9, 0.9 Hz, 1H), 6.06 (dd, *J* = 1.8, 0.9 Hz, 1H), 4.28 (d, *J* = 11.8 Hz, 1H), 4.21 (d, *J* = 9.3 Hz, 1H), 4.09 (s, 1H), 3.73 – 3.70 (m, 2H), 3.65 (s, 3H), 3.35 (s, 3H), 2.81 – 2.78 (m, 1H), 2.28 – 2.21 (m, 1H), 1.93 – 1.87 (m, 1H), 1.44 – 1.40 (m, 1H).

^{13}**C NMR** (176 MHz, $CDCl_3$) δ 219.64, 172.32, 171.11, 143.92, 142.98, 140.61, 139.66, 136.55, 131.78, 131.27, 123.38, 122.56, 121.59, 110.31, 109.75, 66.14, 64.00, 63.91, 63.38, 57.42, 55.73, 54.79, 52.11, 51.86, 41.20, 30.47.

HRMS: calcd. for $[M+H]^+$ $C_{28}H_{28}{}^{79}BrN_2O_7$ = 583.10744, found: 583.10712; calcd. for $[M+H]^+$ $C_{28}H_{28}{}^{81}BrN_2O_7$ = 585.10539, found: 585.10539;

HPLC conditions: CHIRALPAK IC column, *iso*-propanol/ *iso*-hexane = 50/50, flow rate = 0.5 mL min^{-1}, minor enantiomer: t_R = 27.54 min; major enantiomer: t_R = 40.68 min; (95% e.e.);

$[\alpha]^{20}_D$= + 205.6° (c = 0.2, $CHCl_3$).

FT-IR: $\tilde{\nu}$ 2951, 1726, 1501, 1485, 1437, 1364, 1206, 1158, 1073, 1036, 1021, 1010, 873 cm^{-1}.

Experimental studies on the enantioselectivity of cycloaddition

Experimental studies with enone 1a and Imin 2a

Double cycloaddition to obtain product **3a** was performed stepwise. Enone **1a** was treated with 1.1 equiv. of iminoester **2a** and after 3 h second addition of iminoester **2a** (1.1 equiv.) followed. The product was isolated and enantiomeric excess was determined by HPLC analysis on chiral phase.

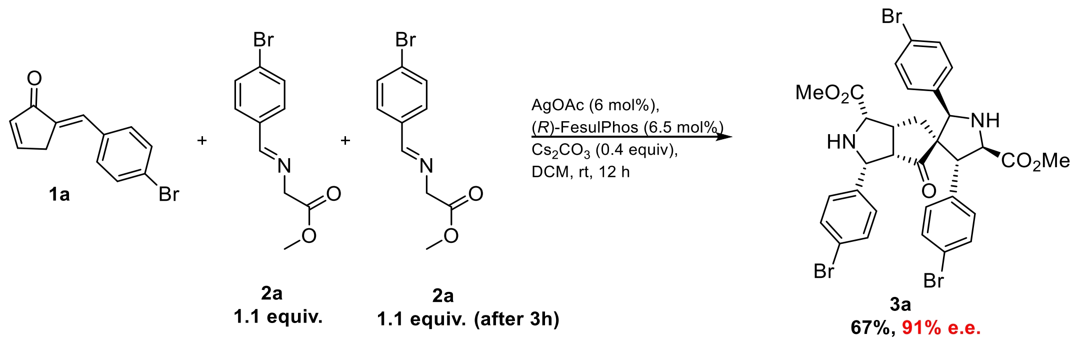

HPLC traces for **3a**: racemic top, enantiomer 1 bottom

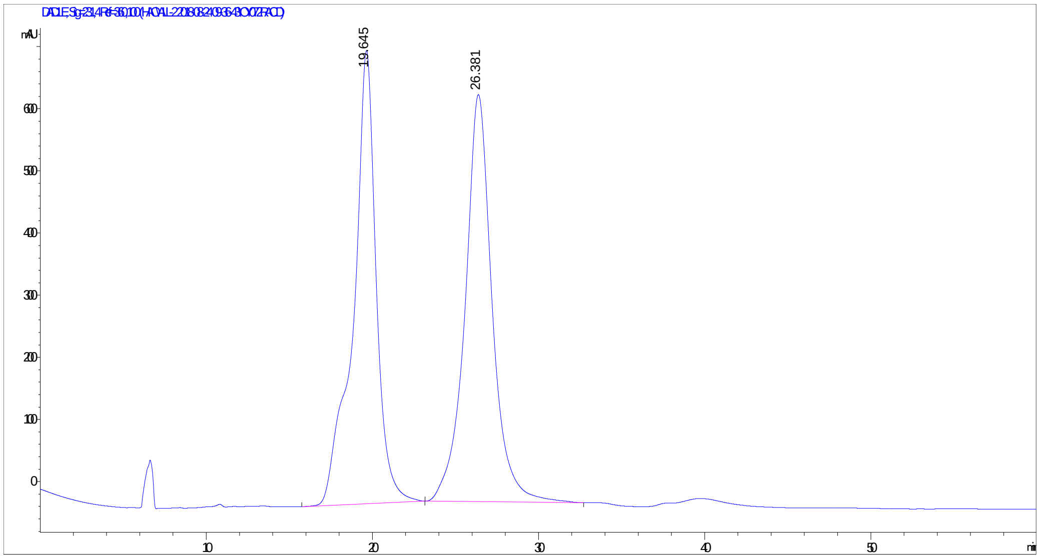

#	Time	Area	Height	Width	Area%	Symmetry
1	19.645	69686.1	729.6	1.3464	48.371	1.34
2	26.381	74380.5	655.4	1.656	51.629	0.932

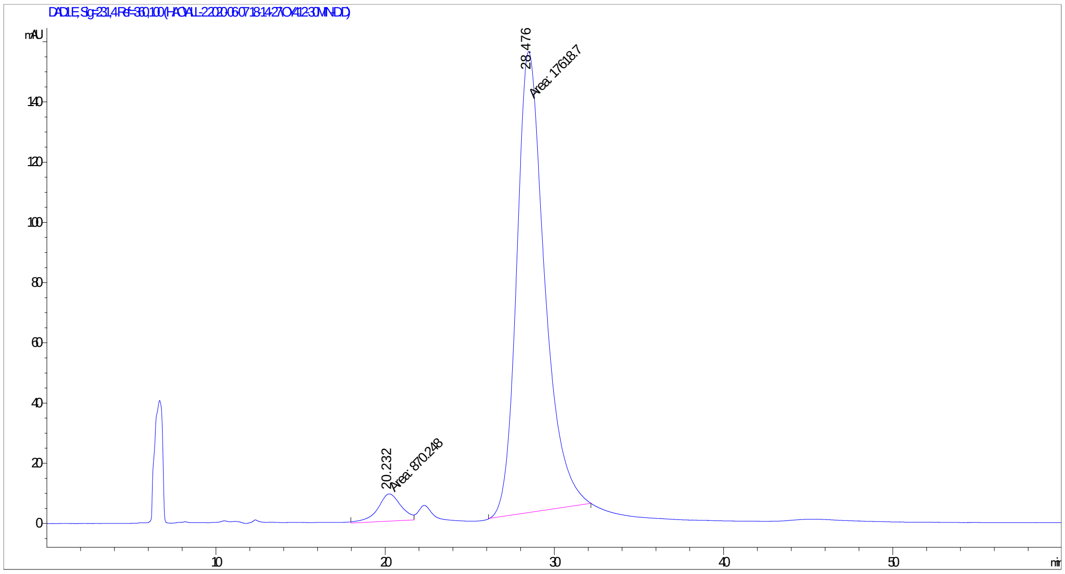

#	Time	Area	Height	Width	Area%	Symmetry
1	20.232	870.2	9.1	1.6025	4.707	0.96
2	28.476	17618.7	153.1	1.918	95.293	0.713

Double cycloaddition to obtain product **3a** was performed stepwise. Enone **1a** was treated with 1.1 equiv. of iminoester **2a** with ratio of AgOAc/(*R*)-Fesulphos (1:2) and after 3 h second addition of iminoester **2a** (1.1 equiv.) followed. The product was isolated and enantiomeric excess was determined by HPLC analysis on chiral phase.

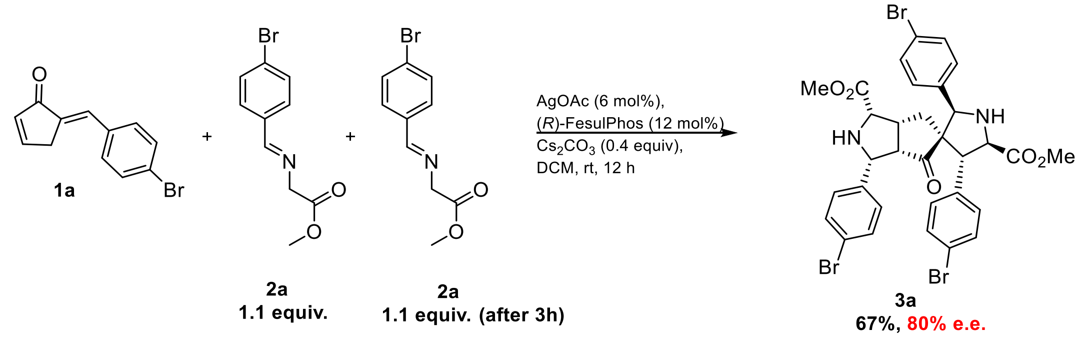

HPLC traces for **3a**: racemic top, enantiomer 1 bottom

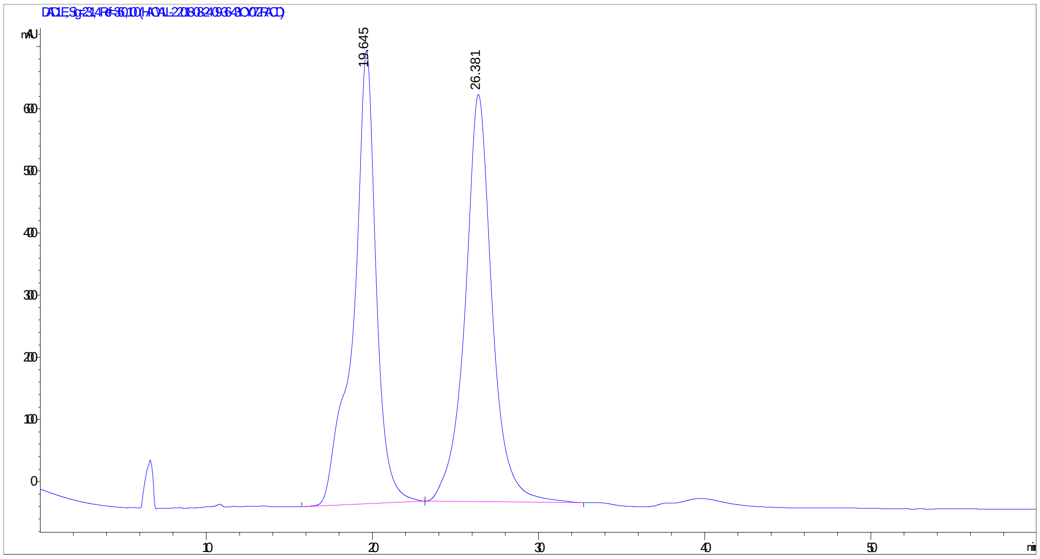

#	Time	Area	Height	Width	Area%	Symmetry
1	19.645	69686.1	729.6	1.3464	48.371	1.34
2	26.381	74380.5	655.4	1.656	51.629	0.932

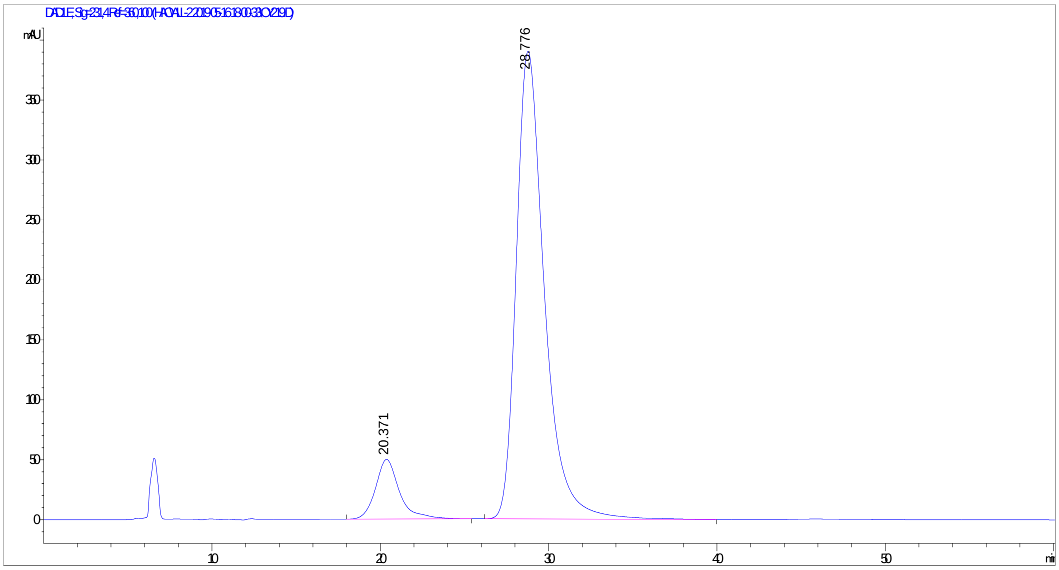

#	Time	Area	Height	Width	Area%	Symmetry
1	20.371	4912.9	49.7	1.4393	9.964	0.799
2	28.776	44392.6	389.2	1.9011	90.036	0.703

Mono cycloaddition of enone **1a** was performed. Enone **1a** was treated with 1.1 equiv. of iminoester **2a** with ratio of AgOAc/(*R*)-Fesulphos 1:2. The product was isolated after 3 h and enantiomeric excess was determined by HPLC analysis on chiral phase.

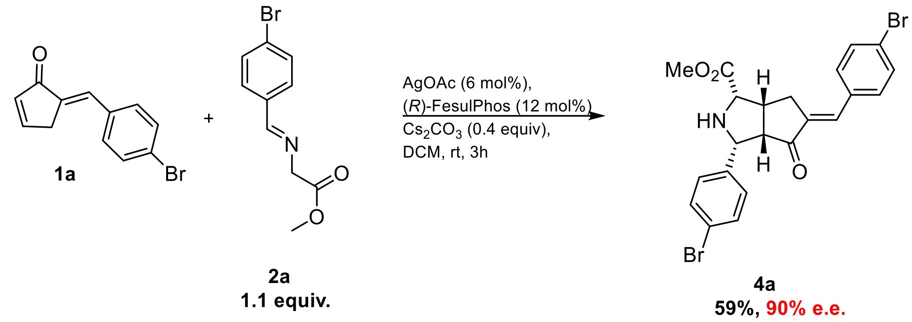

HPLC traces for **4a on chiral IA column**: racemic top, enantiomer 1 bottom

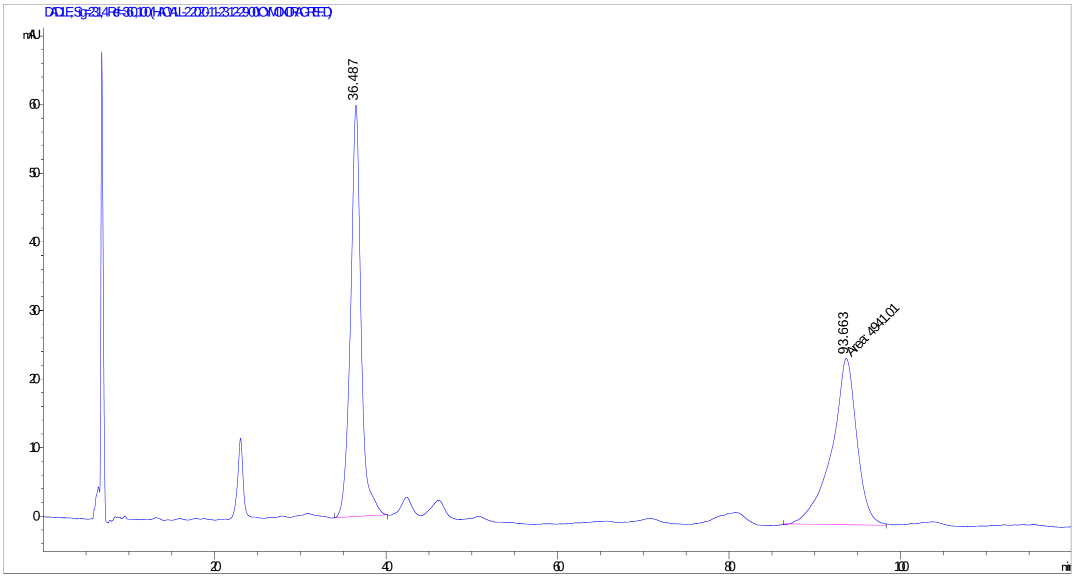

#	Time	Area	Height	Width	Area%	Symmetry
1	36.487	5068.9	59.9	1.2247	50.639	1.011
2	93.663	4941	24.2	3.3981	49.361	1.32

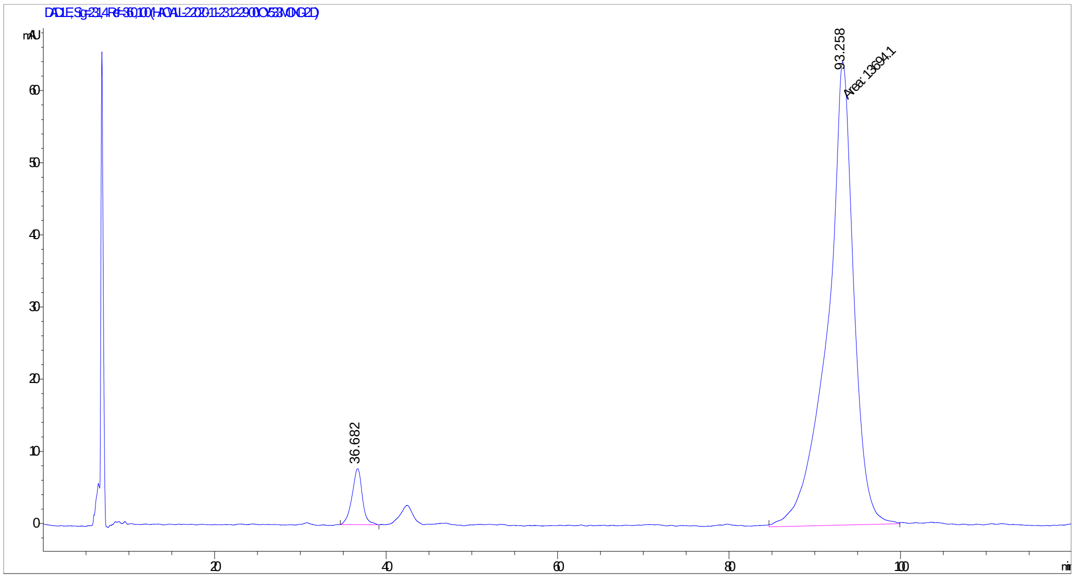

#	Time	Area	Height	Width	Area%	Symmetry
1	36.682	647.9	7.8	0.9873	4.518	1.142
2	93.258	13694.1	64.2	3.555	95.482	1.34

Mono cycloaddition of enone **1a** was performed. Enone **1a** was treated with 1.1 equiv. of iminoester **2a** with ratio of AgOAc/(*R*)-Fesulphos 1:2. The product was isolated after 16 h and enantiomeric excess was determined by HPLC analysis on chiral phase.

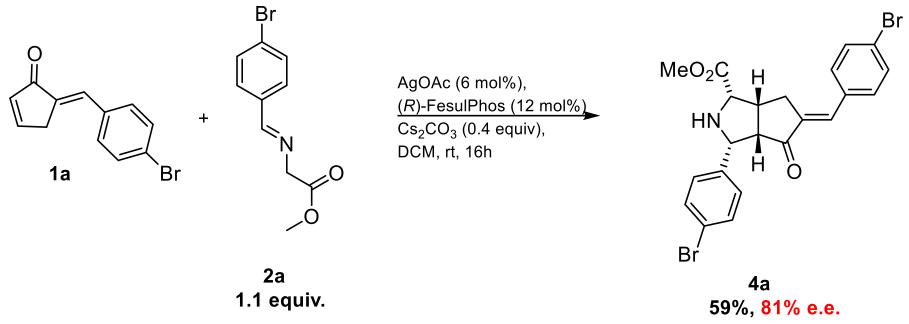

HPLC traces for **4a on chiral IA column**: racemic top, enantiomer 1 bottom

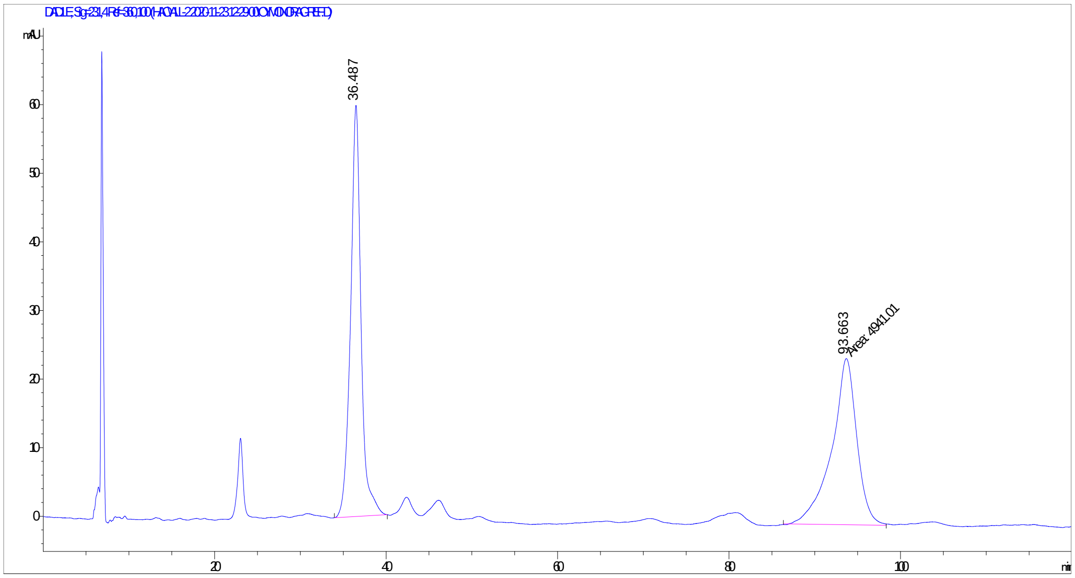

#	Time	Area	Height	Width	Area%	Symmetry
1	36.487	5068.9	59.9	1.2247	50.639	1.011
2	93.663	4941	24.2	3.3981	49.361	1.32

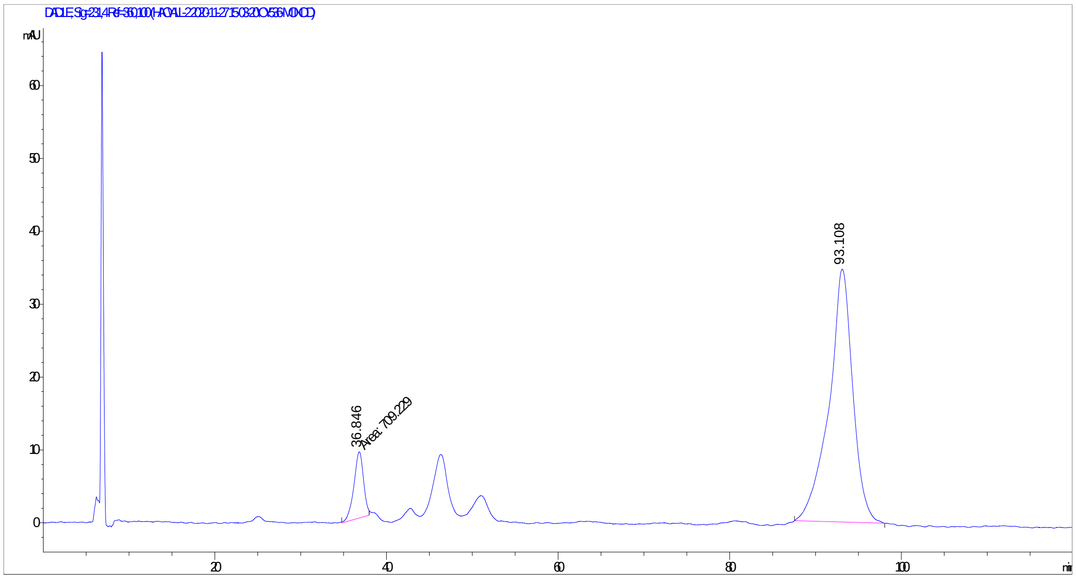

#	Time	Area	Height	Width	Area%	Symmetry
1	36.846	709.2	9.1	1.3	9.594	1.301
2	93.108	6683	34.7	2.2694	90.406	1.212

Mono cycloaddition of enone **1a** was performed. Enone **1a** was treated with 1.1 equiv. of iminoester **2a** with ratio of AgOAc/(*R*)-Fesulphos 1:1 and reaction was run for 3 h. The product was isolated and enantiomeric excess was determined by HPLC analysis on chiral phase.

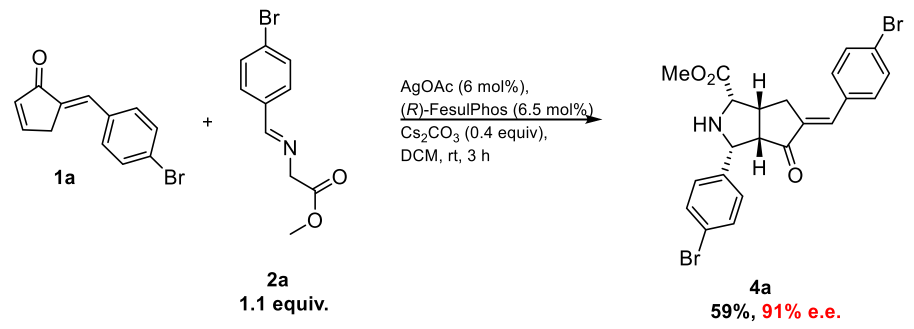

HPLC traces or **4a on chiral IC column**: racemic top, enantiomer 1 bottom

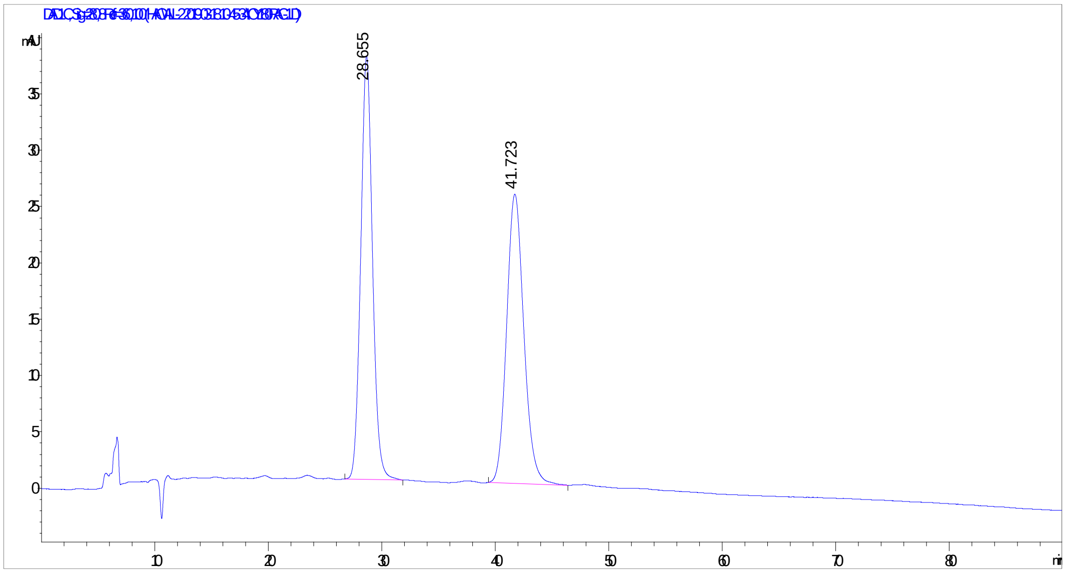

#	Time	Area	Height	Width	Area%	Symmetry
1	28.655	2699.8	37.6	1.0637	49.914	0.93
2	41.723	2709.2	25.7	1.5346	50.086	0.867

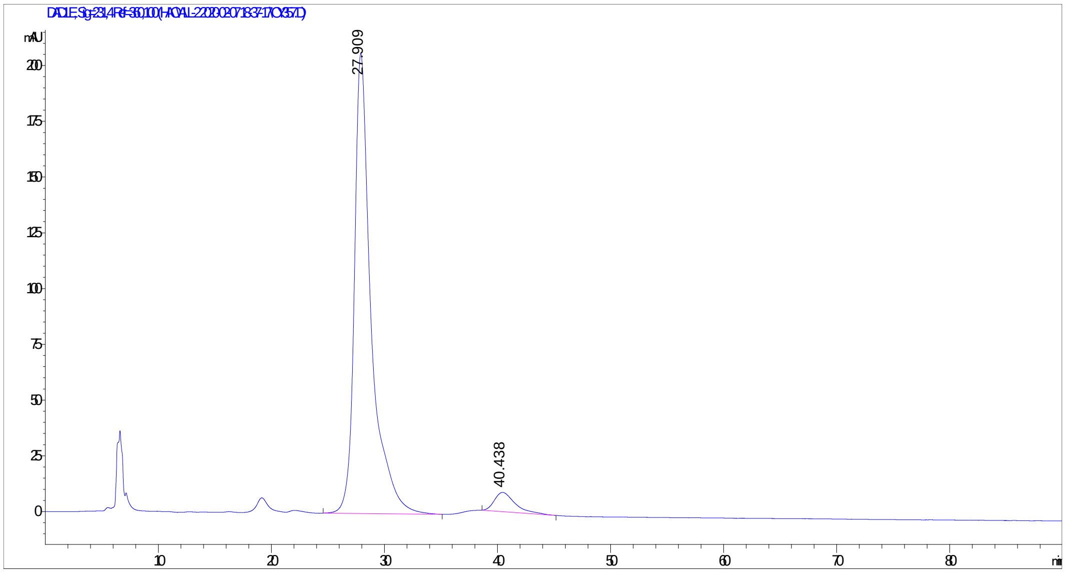

#	Time	Area	Height	Width	Area%	Symmetry
1	27.909	21239.8	206.4	1.4566	95.100	0.585
2	40.438	1094.5	8.6	1.4977	4.900	0.614

Mono cycloaddition of enone **1a** was performed. Enone **1a** was treated with 1.1 equiv. of iminoester **2a** with ratio of AgOAc/(*R*)-Fesulphos 1:1 and reaction was run for 12 h. The product was isolated and enantiomeric excess was determined by HPLC analysis on chiral phase.

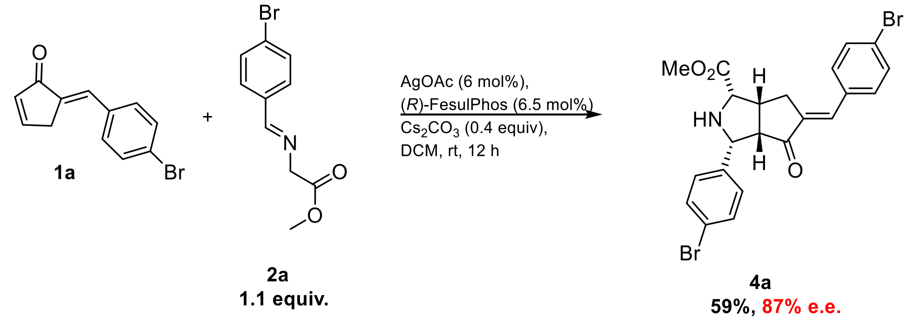

HPLC traces for **4a on chiral IC column**: racemic top, enantiomer 1 bottom

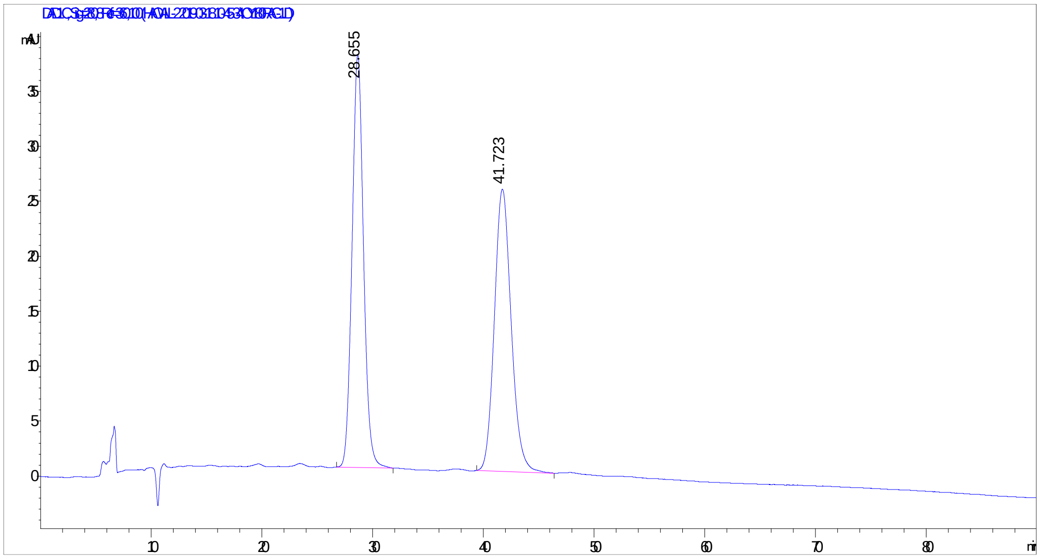

#	Time	Area	Height	Width	Area%	Symmetry
1	28.655	2699.8	37.6	1.0637	49.914	0.93
2	41.723	2709.2	25.7	1.5346	50.086	0.867

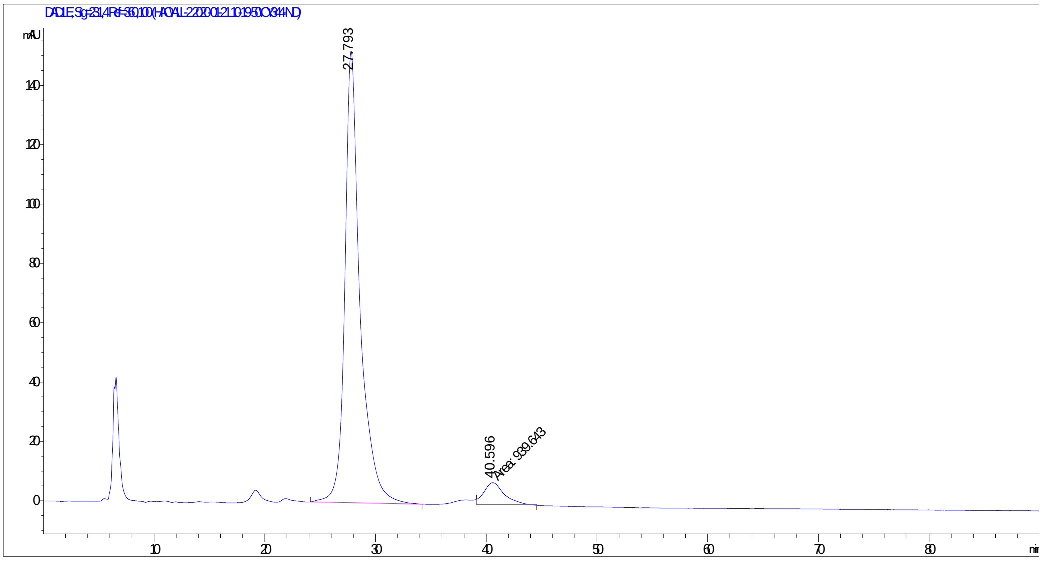

#	Time	Area	Height	Width	Area%	Symmetry
1	27.793	14550.7	152.3	1.3729	93.934	0.657
2	40.596	939.6	7.4	2.1229	6.066	0.773

Mono cycloaddition of enone **1a** was performed. Enone **1a** was treated with 1.1 equiv. of iminoester **2a** with ratio of AgOAc/(*R*)-Fesulphos 1:1 and reaction was run for 24 h. The product was isolated and enantiomeric excess was determined by HPLC analysis on chiral phase.

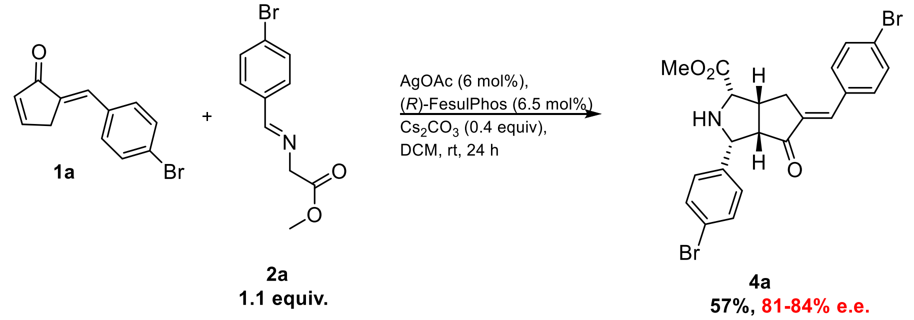

2a
1.1 equiv.

4a
57%, 81-84% e.e.

HPLC traces for **4a on chiral IC column**: racemic top, enantiomer 1 bottom

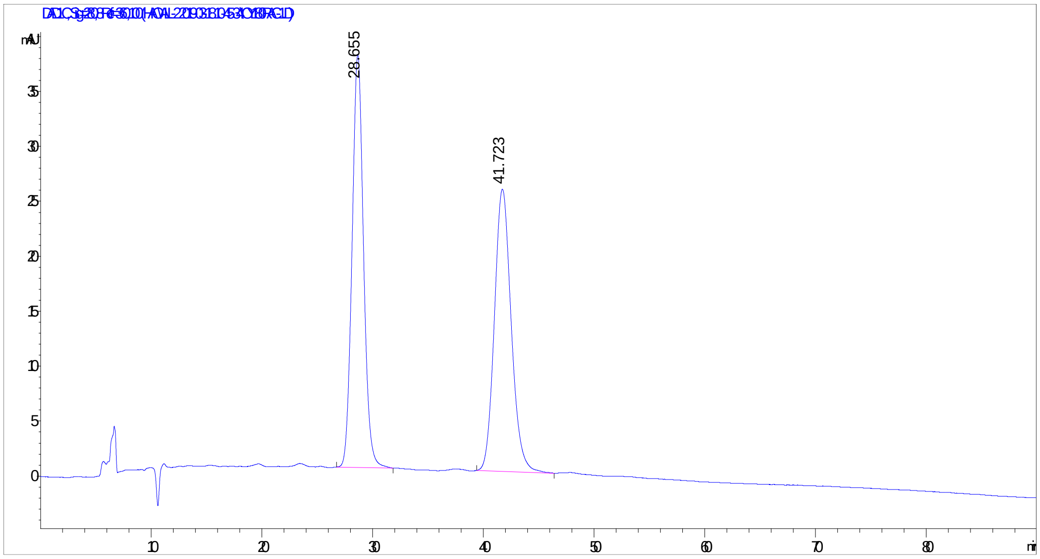

#	Time	Area	Height	Width	Area%	Symmetry
1	28.655	2699.8	37.6	1.0637	49.914	0.93
2	41.723	2709.2	25.7	1.5346	50.086	0.867

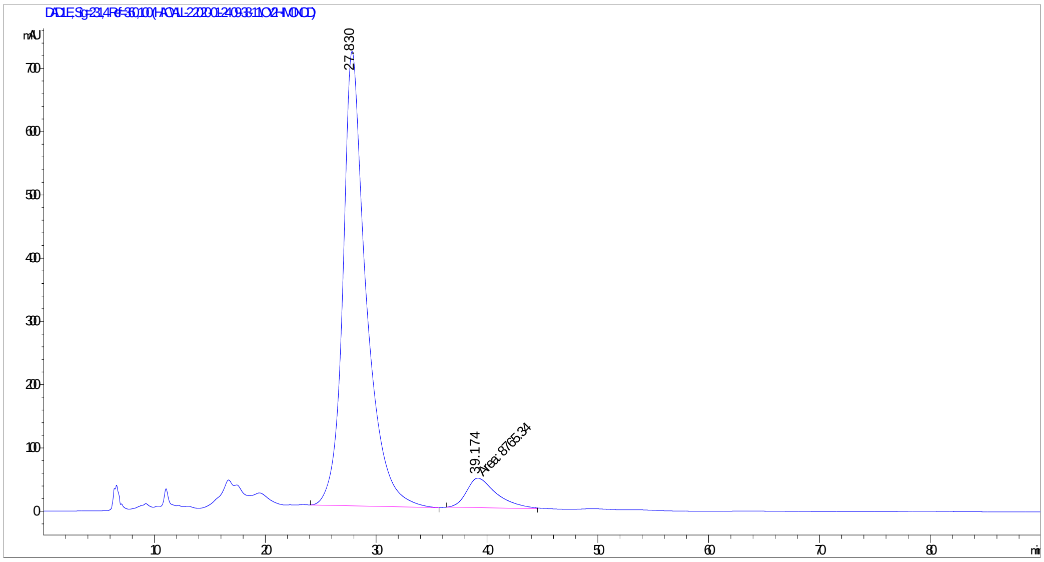

#	Time	Area	Height	Width	Area%	Symmetry
1	27.83	104904.6	718.4	2.0277	92.289	0.628
2	39.174	8765.3	46.6	3.134	7.711	0.573

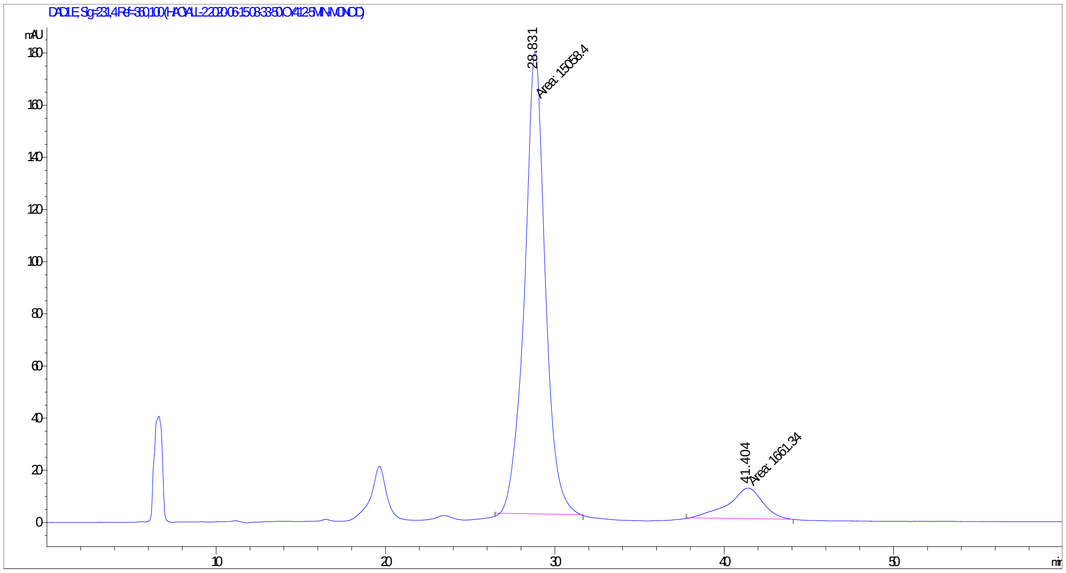

#	Time	Area	Height	Width	Area%	Symmetry
1	28.831	15058.4	177.4	1.4146	90.064	1.003
2	41.404	1661.3	11.7	2.3604	9.936	1.318

Racemic mono cycloaddition product **4a** was subjected to chiral conditions and was treated with AgOAc, (R)-Fesulphos and Cs_2CO_3. . The product was isolated and enantiomeric excess was determined by HPLC analysis on chiral phase. Mono cycloaddition product **4a** was recovered as opposite enantiomer, racemization occurs.

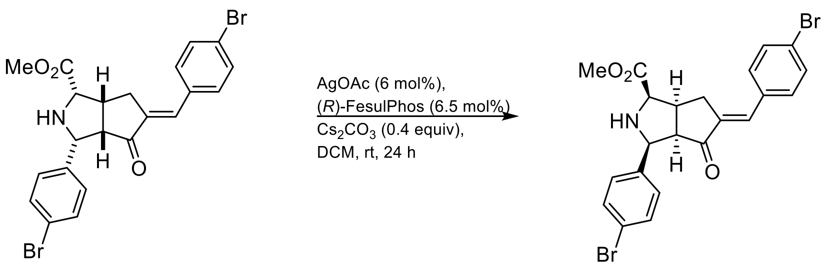

4a ***racemic*** → **4a** **94%, -10% e.e.**

HPLC traces for **4a** on **chiral IA column**: racemic top, enantiomer 1 bottom

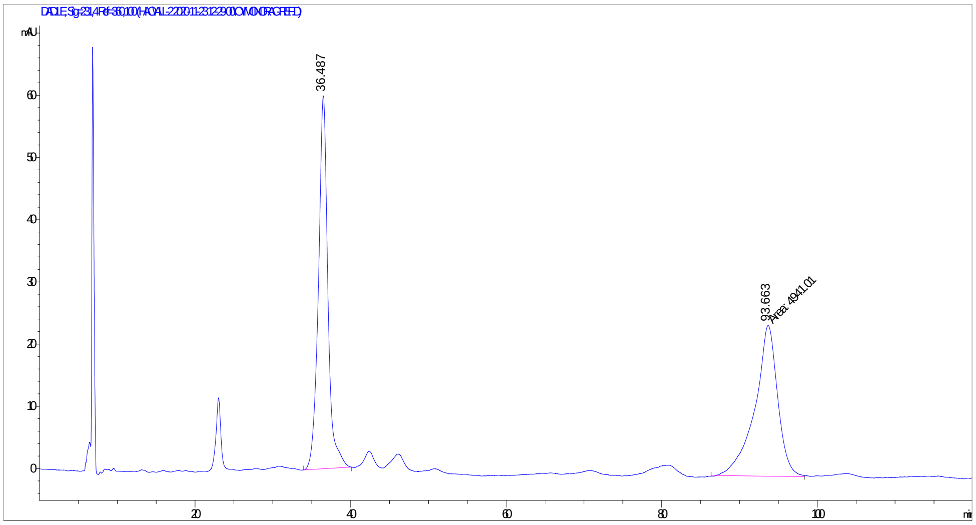

#	Time	Area	Height	Width	Area%	Symmetry
1	36.487	5068.9	59.9	1.2247	50.639	1.011
2	93.663	4941	24.2	3.3981	49.361	1.32

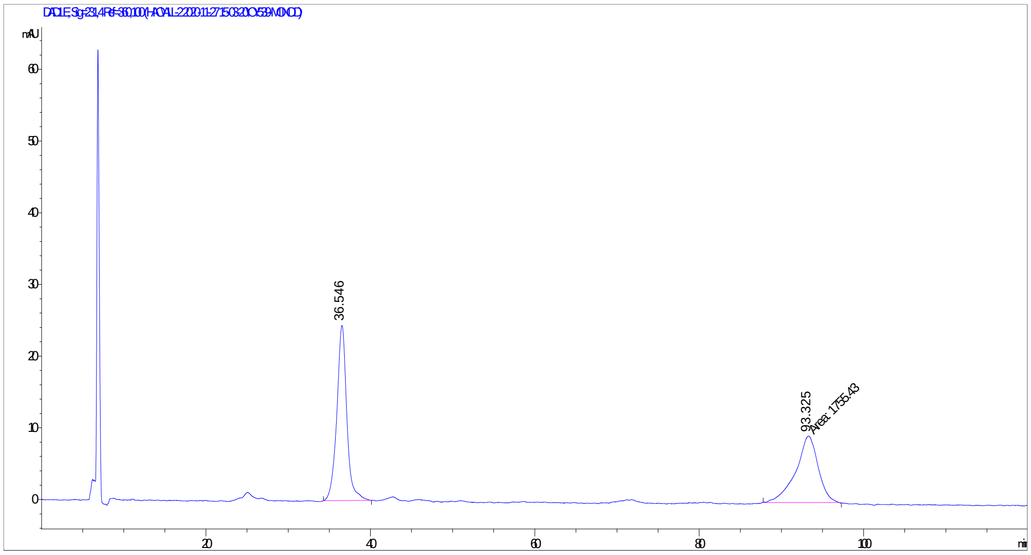

#	Time	Area	Height	Width	Area%	Symmetry
1	36.546	2061.6	24.4	1.1919	54.011	1.016
2	93.325	1755.4	9.3	3.1535	45.989	1.336

Enantioenriched mono cycloaddition product **4a** was subjected to chiral conditions and was treated with AgOAc, (R)-Fesulphos and Cs_2CO_3. . The product was isolated and enantiomeric excess was determined by HPLC analysis on chiral phase. Mono cycloaddition product **4a** was recovered with decreased enantiomeric excess, racemization occurs.

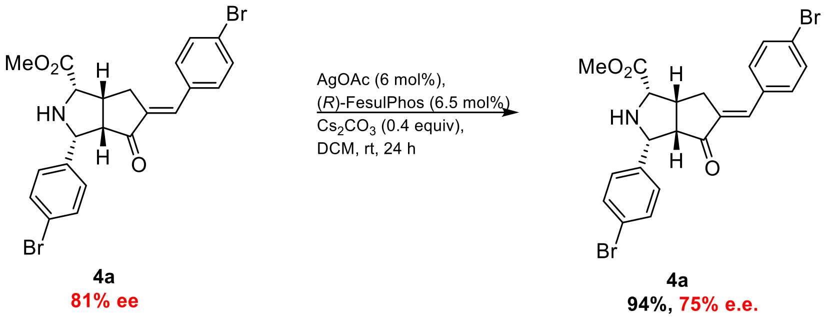

HPLC traces for **4a** on **chiral IA column**: racemic top, enantiomer 1 bottom

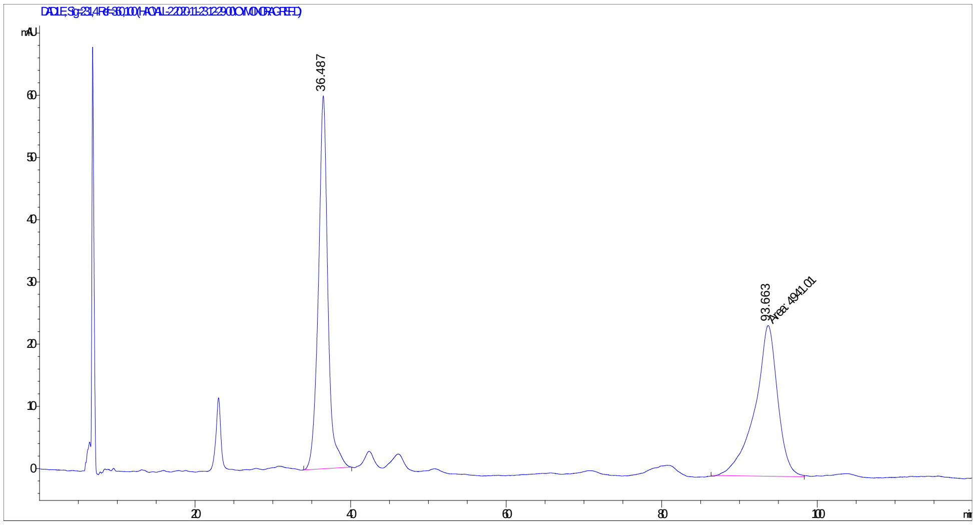

#	Time	Area	Height	Width	Area%	Symmetry
1	36.487	5068.9	59.9	1.2247	50.639	1.011
2	93.663	4941	24.2	3.3981	49.361	1.32

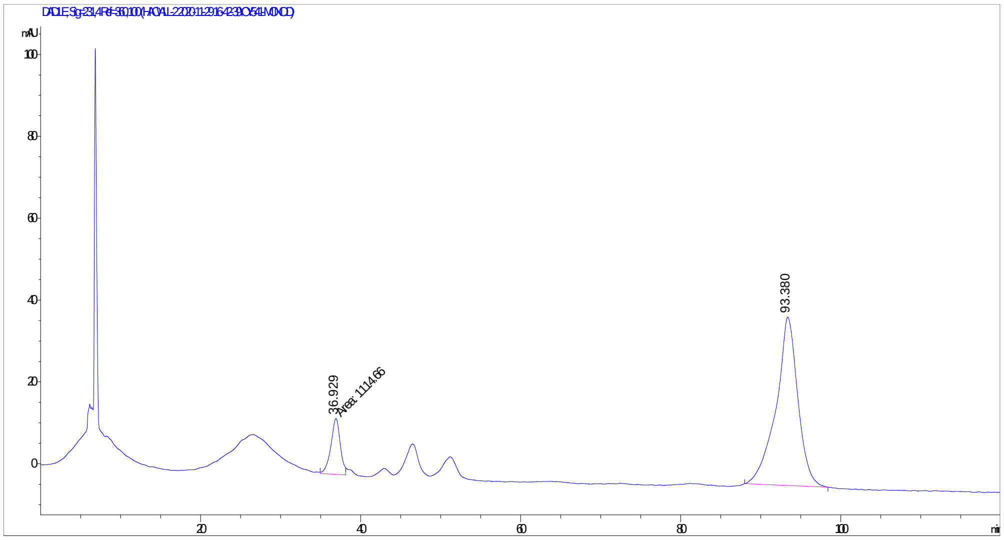

#	Time	Area	Height	Width	Area%	Symmetry
1	36.929	1114.7	13.6	1.3612	12.536	1.176
2	93.38	7777.4	41.1	2.2254	87.464	1.145

Enantioenriched mono cycloaddition product **4a** was treated with 1.1 equiv. of iminoester **2a**. The product was isolated and enantiomeric excess was determined by HPLC analysis on chiral phase.

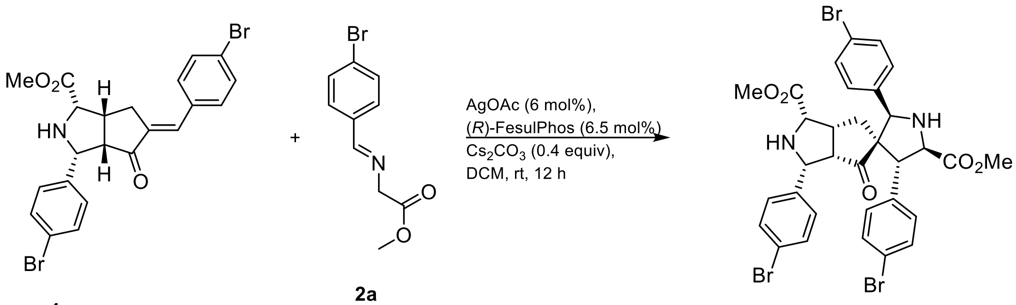

4a **70% e.e.**

2a **1.1 equiv.**

3a **59%, 60% e.e.**

HPLC traces for **3a**: racemic top, enantiomer 1 bottom

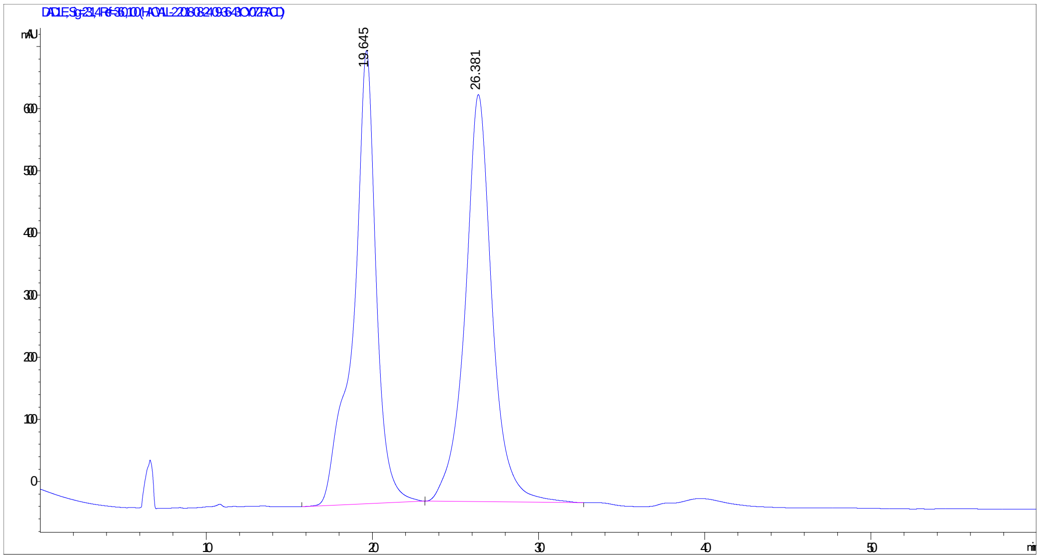

#	Time	Area	Height	Width	Area%	Symmetry
1	19.645	69686.1	729.6	1.3464	48.371	1.34
2	26.381	74380.5	655.4	1.656	51.629	0.932

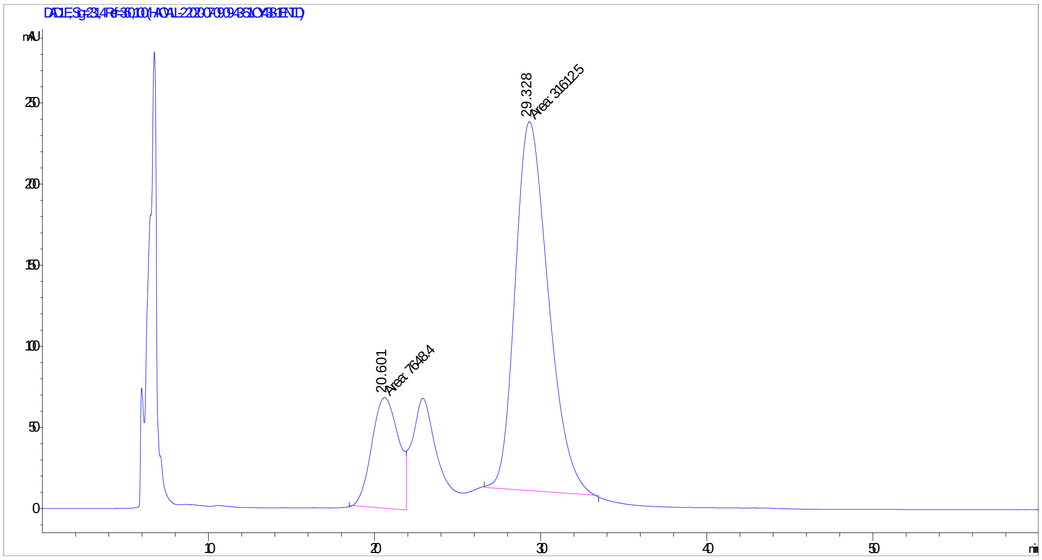

#	Time	Area	Height	Width	Area%	Symmetry
1	20.601	7648.4	68.2	1.8703	19.481	0.868
2	29.328	31612.5	227.5	2.3159	80.519	0.725

Enantioenriched mono cycloaddition product **4a** was treated with 1.1 equiv. of iminoester **2a** using achiral ligand PPh_3. The product was isolated and enantiomeric excess was determined by HPLC analysis on chiral phase.

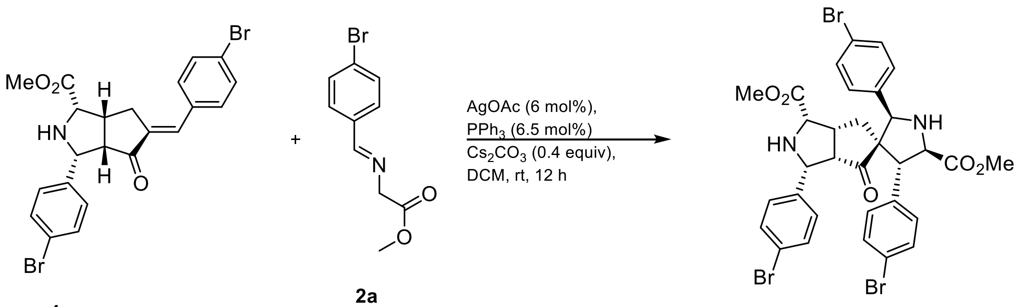

4a 70% e.e.

2a 1.1 equiv.

3a 59%, 70% e.e.

HPLC traces for **3a**: racemic top, enantiomer 1 bottom

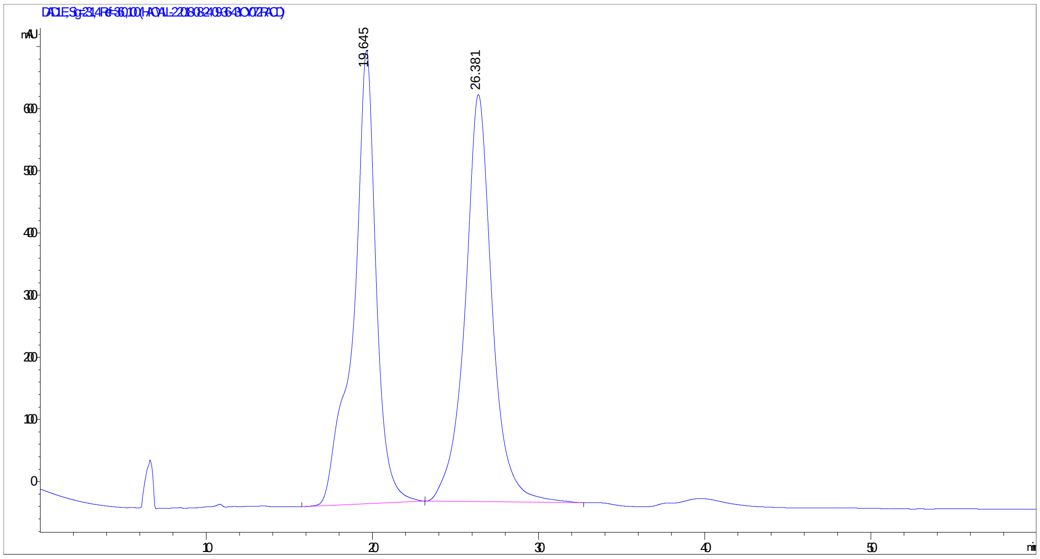

#	Time	Area	Height	Width	Area%	Symmetry
1	19.645	69686.1	729.6	1.3464	48.371	1.34
2	26.381	74380.5	655.4	1.656	51.629	0.932

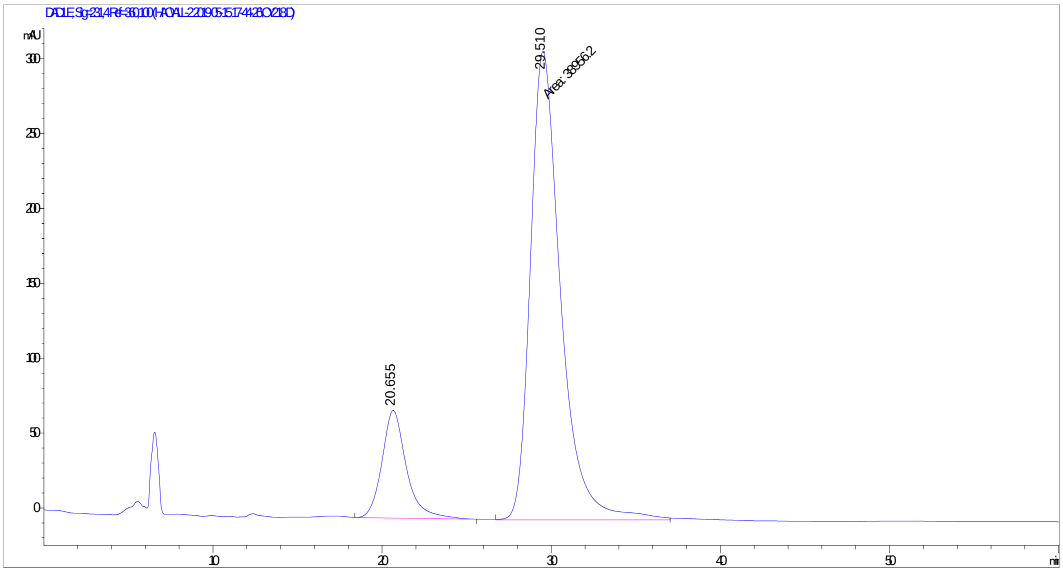

#	Time	Area	Height	Width	Area%	Symmetry
1	20.655	7067.4	72	1.6358	15.671	0.889
2	29.51	38029.9	311.4	2.0356	84.329	0.688

Experimental studies with enone 1a and Imin 2g.

Mono cycloaddition of enone **1a** was performed. Enone **1a** was treated with 1.1 equiv. of iminoester **2g**. Product **4c** was isolated and enantiomeric excess was determined by HPLC analysis on chiral phase.

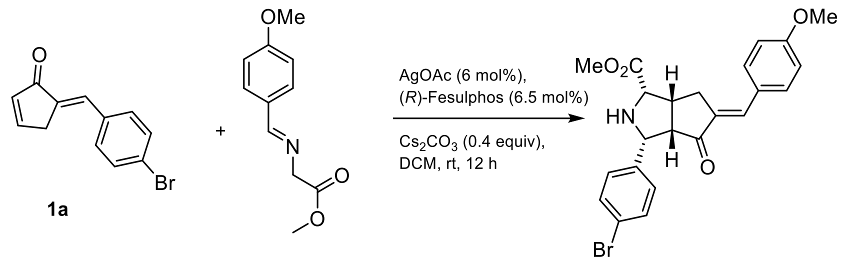

2g
1.1 equiv.

4c
61% yield, 70% e.e.

HPLC traces for **4c**: racemic top, enantiomer 1 bottom

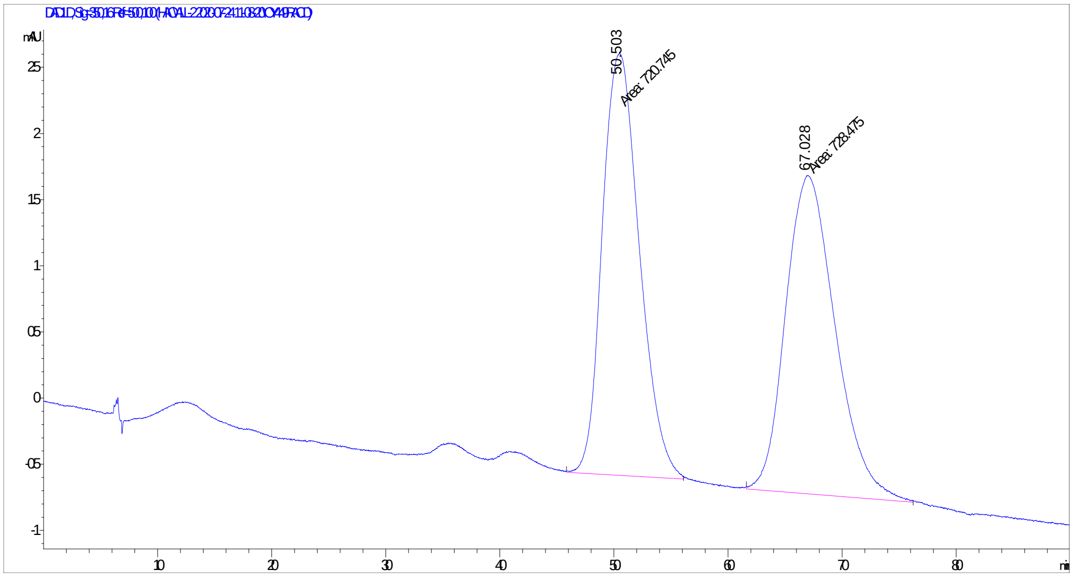

#	Time	Area	Height	Width	Area%	Symmetry
1	50.503	720.7	3.2	3.7647	49.733	0.839
2	67.028	728.5	2.4	5.0412	50.267	0.795

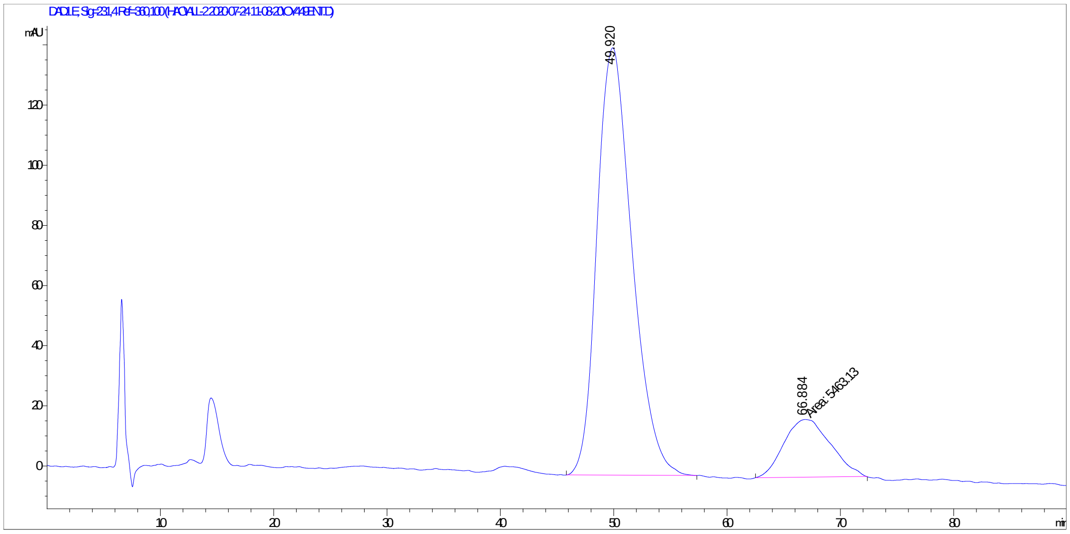

#	Time	Area	Height	Width	Area%	Symmetry
1	49.92	30572.2	142.1	2.6904	84.840	0.798
2	66.884	5463.1	19.2	4.7532	15.160	0.814

Enantioenriched mono cycloaddition product **4c** was treated with 1.1 equiv. of iminoester **2g**. The product was isolated and enantiomeric excess was determined by HPLC analysis on chiral phase.

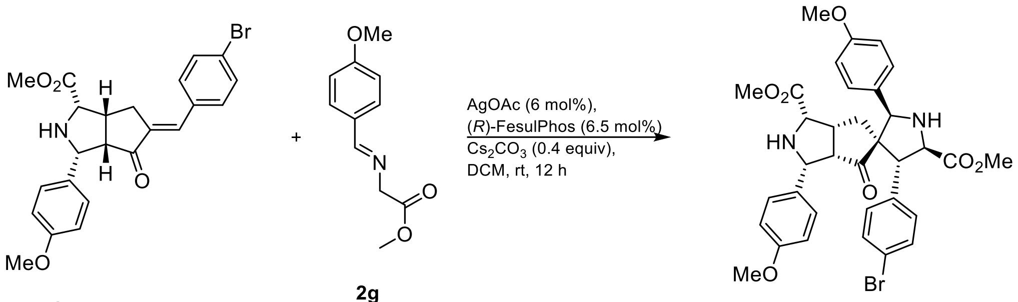

4a **70% e.e.**

2g **1.1 equiv.**

3e **64%, 60% e.e.**

HPLC traces for racemic **3e**

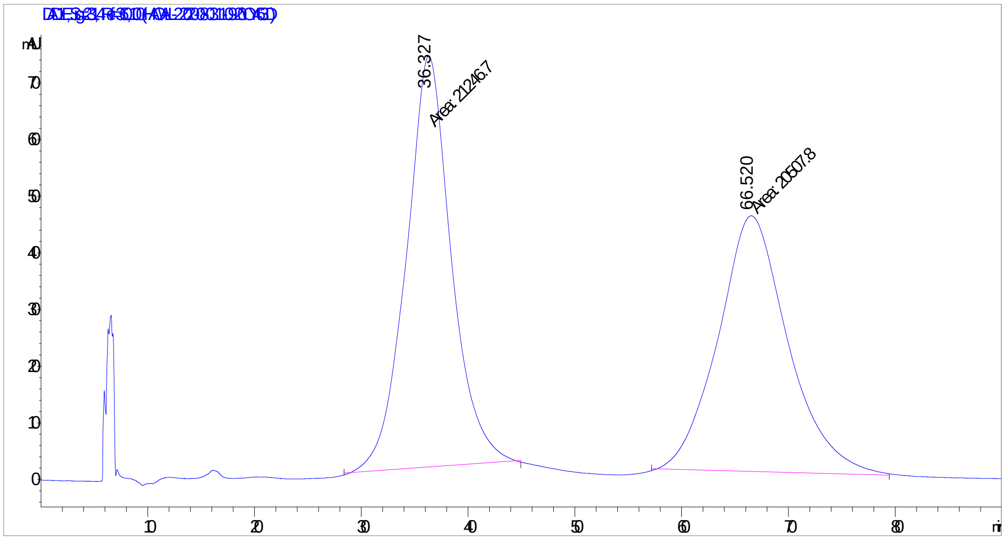

#	Time	Area	Height	Width	Area%	Symmetry
1	36.327	21246.7	72.5	4.8815	50.885	0.979
2	66.52	20507.8	45.1	7.5753	49.115	0.869

HPLC traces for enantiomer **3e**

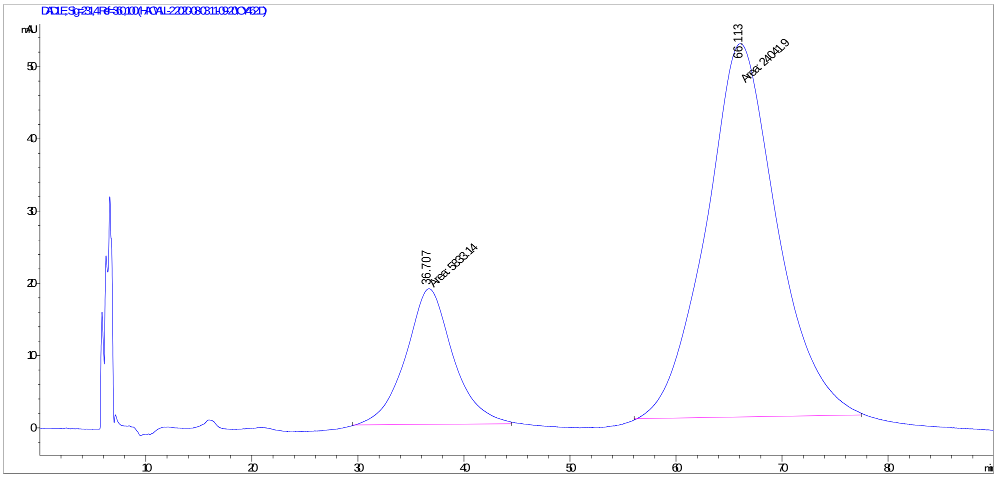

#	Time	Area	Height	Width	Area%	Symmetry
1	36.707	5833.1	18.8	5.1751	19.525	0.943
2	66.113	24041.9	51.7	7.7531	80.475	0.936

Enantioenriched mono cycloaddition product **4c** was treated with 1.1 equiv. of iminoester **2g** under racemic coditions. The product was isolated and enantiomeric excess was determined by HPLC analysis on chiral phase.

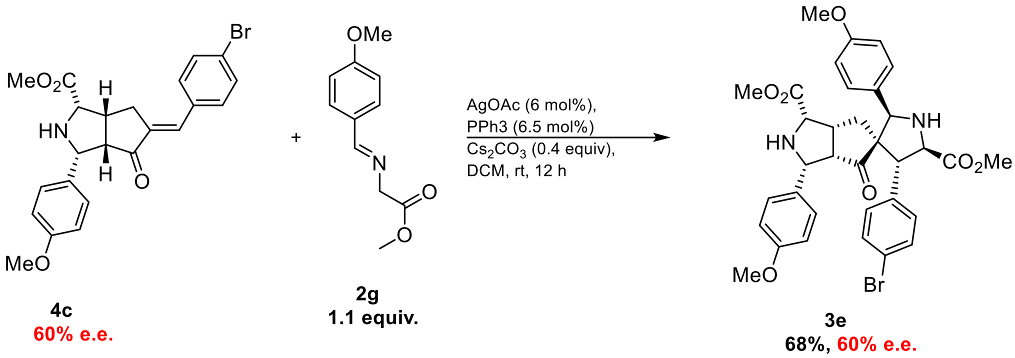

4c
60% e.e.

2g
1.1 equiv.

3e
68%, 60% e.e.

HPLC traces for enantiomer **3e**

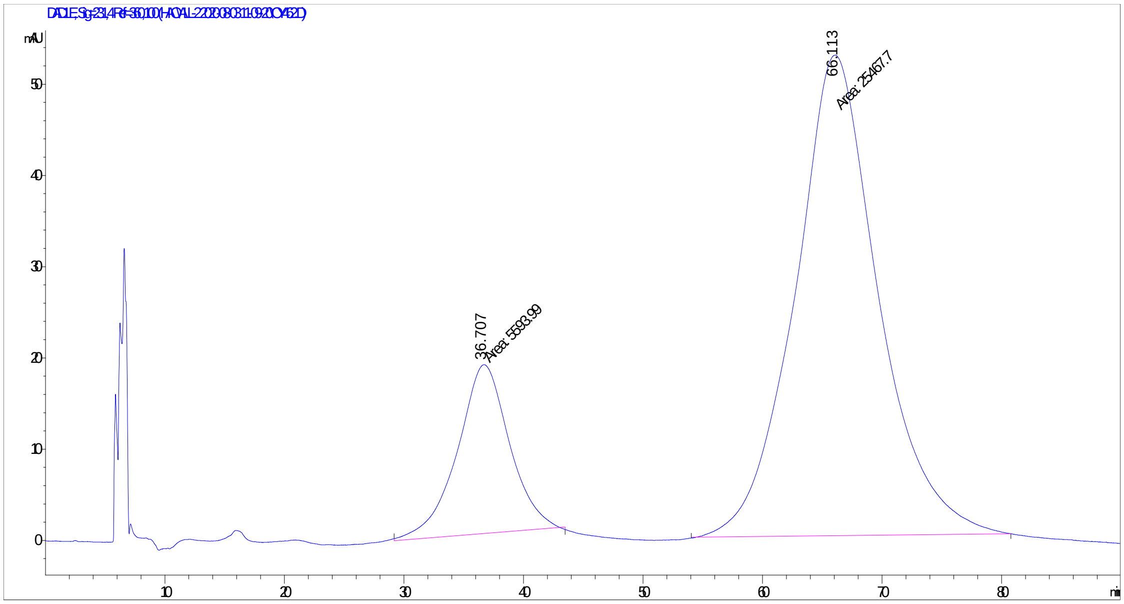

#	Time	Area	Height	Width	Area%	Symmetry
1	36.707	5594	18.5	5.0391	18.009	1.044
2	66.113	25467.7	52.7	8.0581	81.991	0.922

Experimental studies on racemic mono adduct *rac*-4a

Racemic mono cycloaddition product **4a** was treated with 1.1 equiv. of iminoester **2a**. The product was isolated and enantiomeric excess was determined by HPLC analysis on chiral phase. Opposite enantiomer compared double cycloaddition with enone **1a** was obtained.

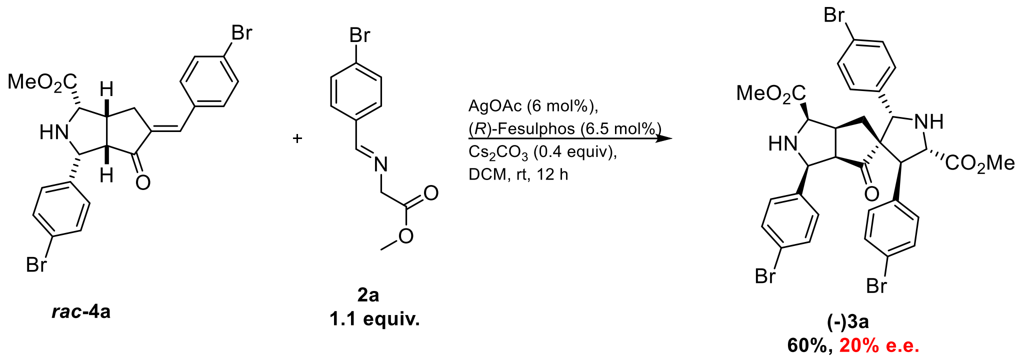

HPLC traces for (-)**3a**: racemic top, enantiomer 1 bottom

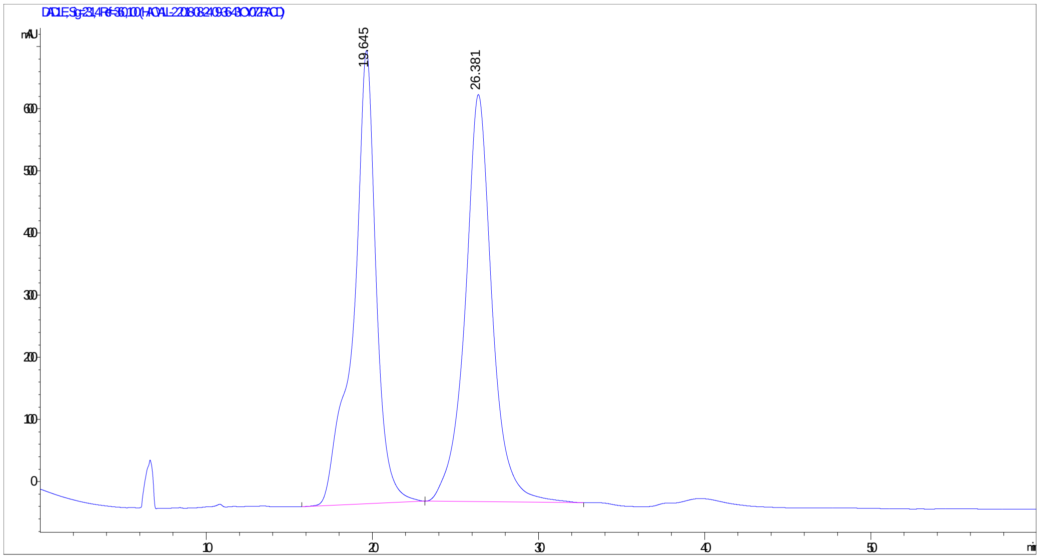

#	Time	Area	Height	Width	Area%	Symmetry
1	19.645	69686.1	729.6	1.3464	48.371	1.34
2	26.381	74380.5	655.4	1.656	51.629	0.932

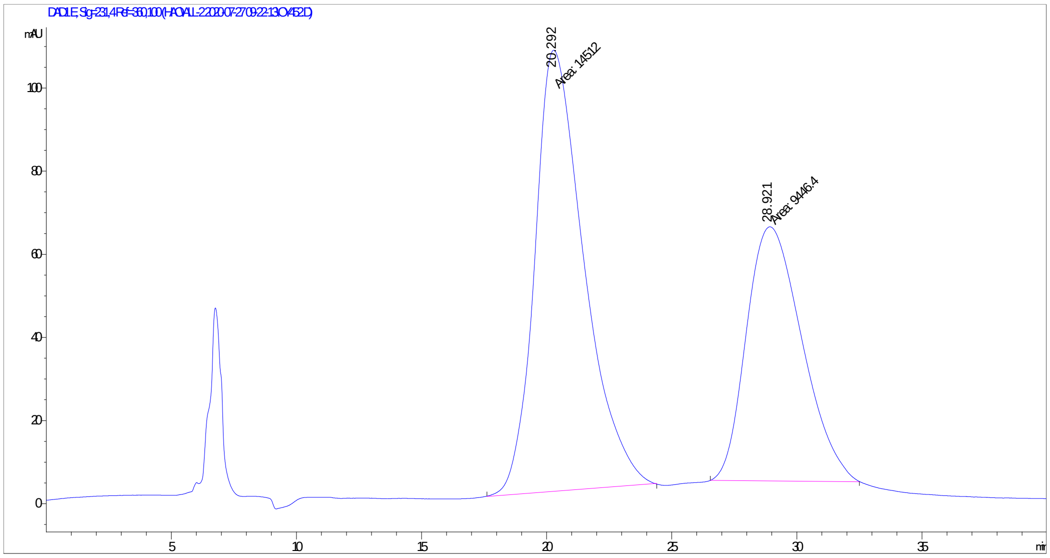

#	Time	Area	Height	Width	Area%	Symmetry
1	20.292	14512	106.1	2.2794	60.572	0.631
2	28.921	9446.4	61.1	2.5748	39.428	0.711

Racemic mono cycloaddition product ***rac*-4a** was treated with 1.1 equiv. of iminoester **2a**. The course of reaction was observed over time. Two different products were observed, opposite enantiomer *endo, endo* **(-)3a** and diastereomer *endo, exo* **6a** with varying ratio and change in enantioselectivity over time. Both products were isolated after certain time points and enantiomeric excess was determined by HPLC analysis on chiral phase.

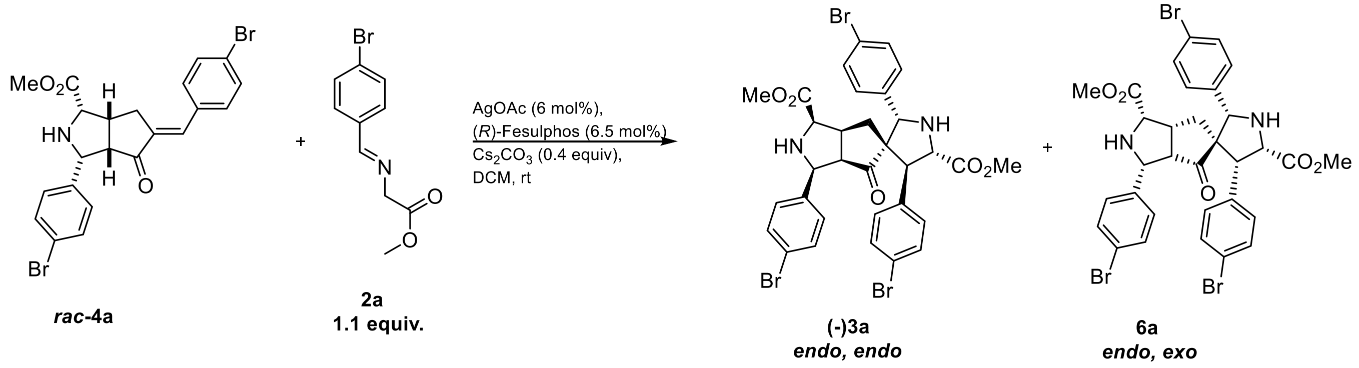

HPLC traces of *endo, endo* **(-)3a** on chiral **IC-column** after 3 min

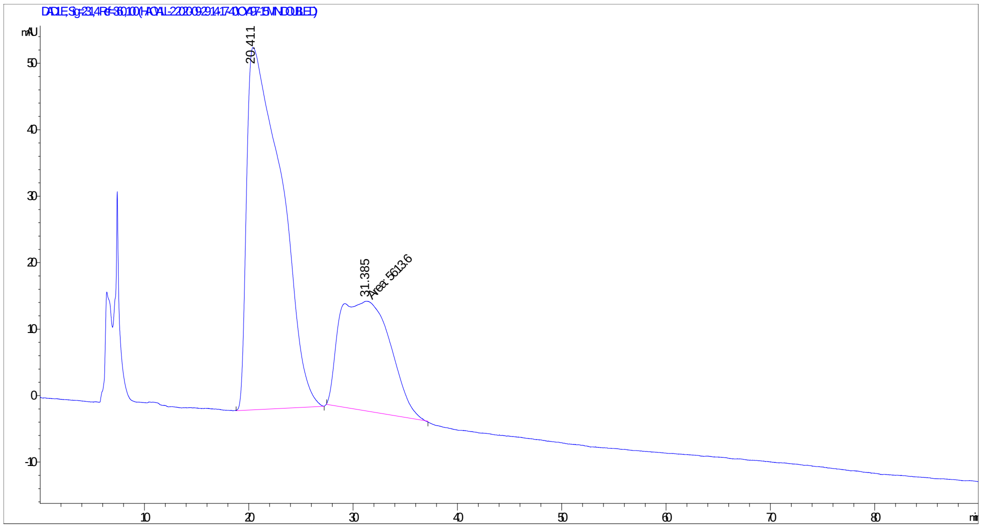

#	Time	Area	Height	Width	Area%	Symmetry
1	20.411	12518.4	54.5	2.7252	69.040	0.223
2	31.385	5613.6	16.5	5.6548	30.960	1.114

HPLC traces of *endo, exo* **6a** on chiral **IC-column** after 3 min

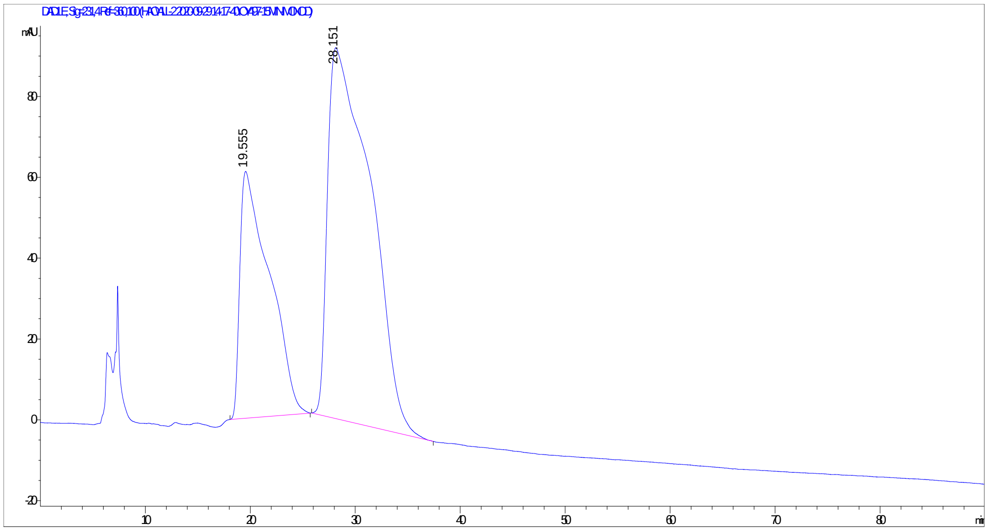

#	Time	Area	Height	Width	Area%	Symmetry
1	19.555	11910.5	61.1	2.4313	31.283	0.224
2	28.151	26163.2	91.8	3.3898	68.717	0.227

HPLC traces of *endo, endo* **(-)3a** on chiral **IC-column** after 7 min

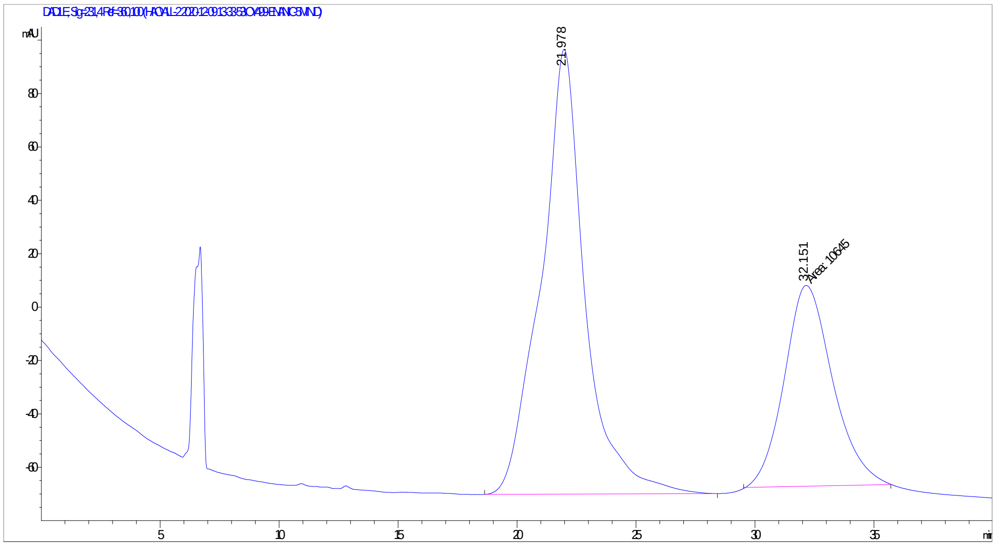

#	Time	Area	Height	Width	Area%	Symmetry
1	21.978	21403.6	166.7	1.7563	66.785	1.049
2	32.151	10645	75.2	2.3589	33.215	0.806

HPLC traces of *endo, exo* **6a** on chiral **IC-column** after 7 min

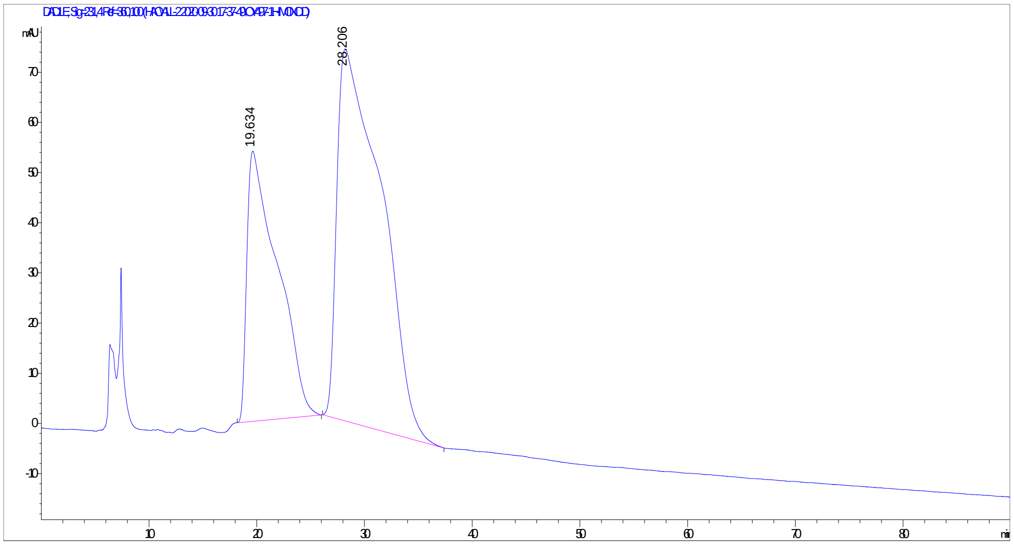

#	Time	Area	Height	Width	Area%	Symmetry
1	19.634	10609.3	53.9	2.5014	33.273	0.223
2	28.206	21276.3	74.1	3.3663	66.727	0.225

HPLC traces of *endo, endo* **(-)3a** on chiral **IC-column** after 15 min

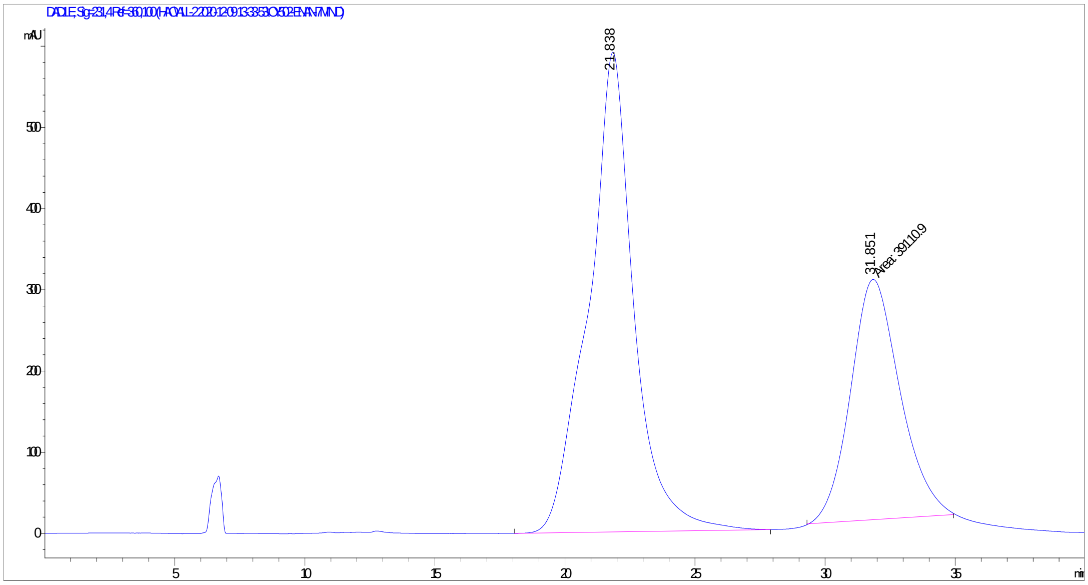

#	Time	Area	Height	Width	Area%	Symmetry
1	21.838	71459.1	590.8	1.679	64.628	1.098
2	31.851	39110.9	295.9	2.2028	35.372	0.829

HPLC traces of *endo, exo* **6a** on chiral **IC-column** after 15 min

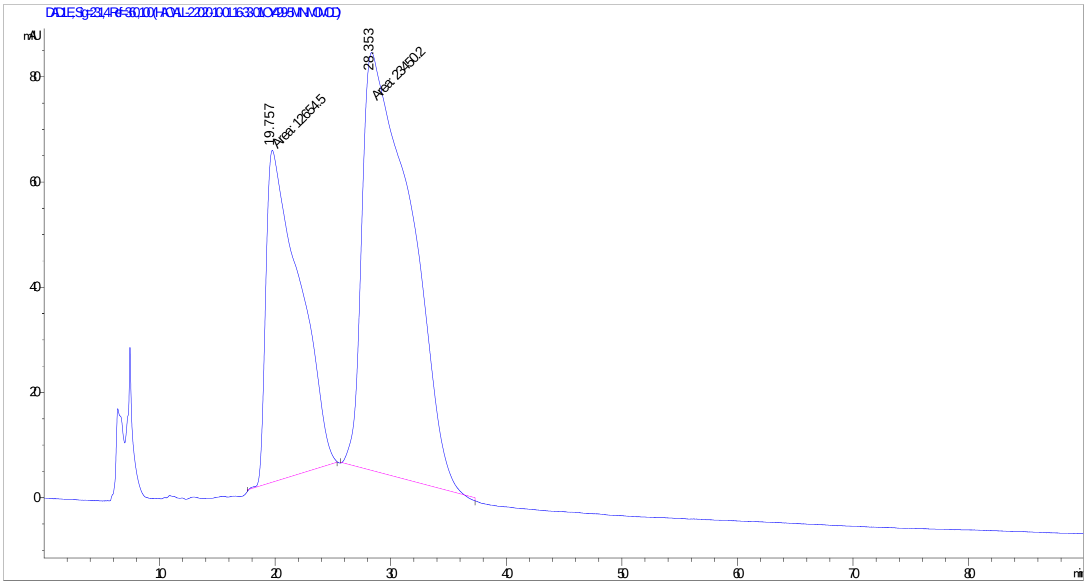

#	Time	Area	Height	Width	Area%	Symmetry
1	19.757	12654.5	63.1	3.3429	35.050	0.237
2	28.353	23450.2	79.4	4.9199	64.950	0.249

HPLC traces of *endo, endo* **(-)3a** on chiral **IC-column** after 1h

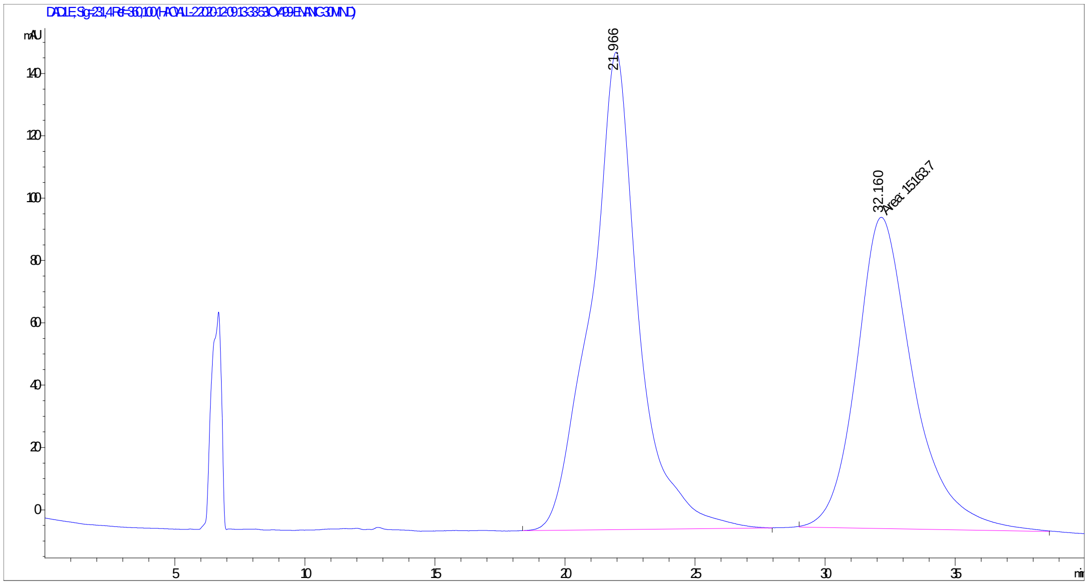

#	Time	Area	Height	Width	Area%	Symmetry
1	21.966	19428.5	153.2	1.7457	56.164	1.067
2	32.16	15163.7	99.9	2.531	43.836	0.742

HPLC traces of *endo, exo* **6a** on chiral **IC-column** after 1h

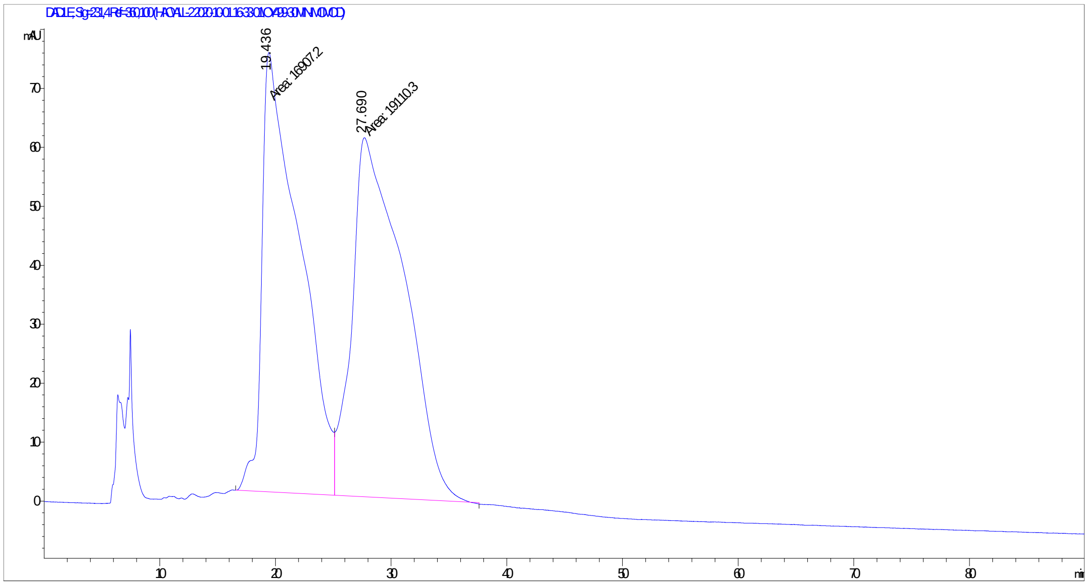

#	Time	Area	Height	Width	Area%	Symmetry
1	19.436	16907.2	74.5	3.7804	46.942	0
2	27.69	19110.3	60.9	5.2288	53.058	0.327

Racemic mono cycloaddition product **4c** was treated with 1.1 equiv. of iminoester **2g**. The product was isolated and enantiomeric excess was determined by HPLC analysis on chiral phase. Opposite enantiomer compared double cycloaddition with enone **1a** was obtained.

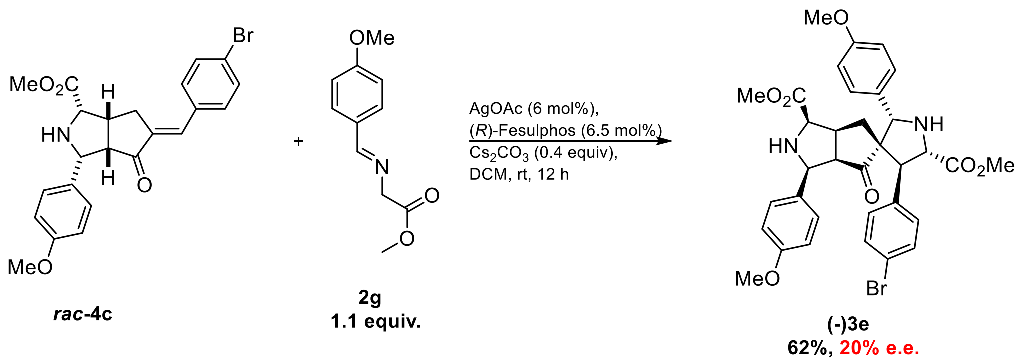

HPLC traces of **(-)3e,** racemic top, enantiomer 1 bottom

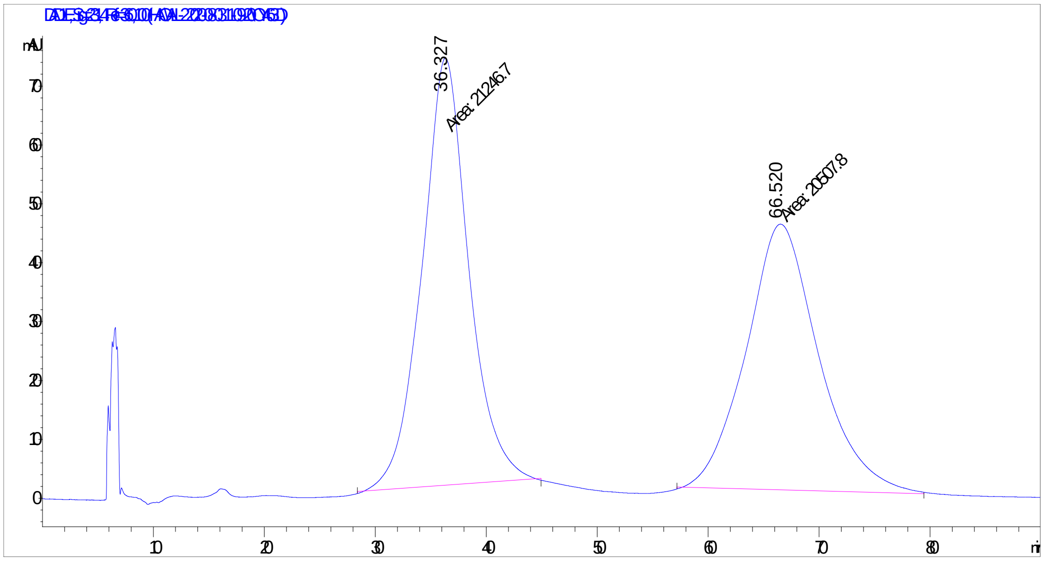

#	Time	Area	Height	Width	Area%	Symmetry
1	36.327	21246.7	72.5	4.8815	50.885	0.979
2	66.52	20507.8	45.1	7.5753	49.115	0.869

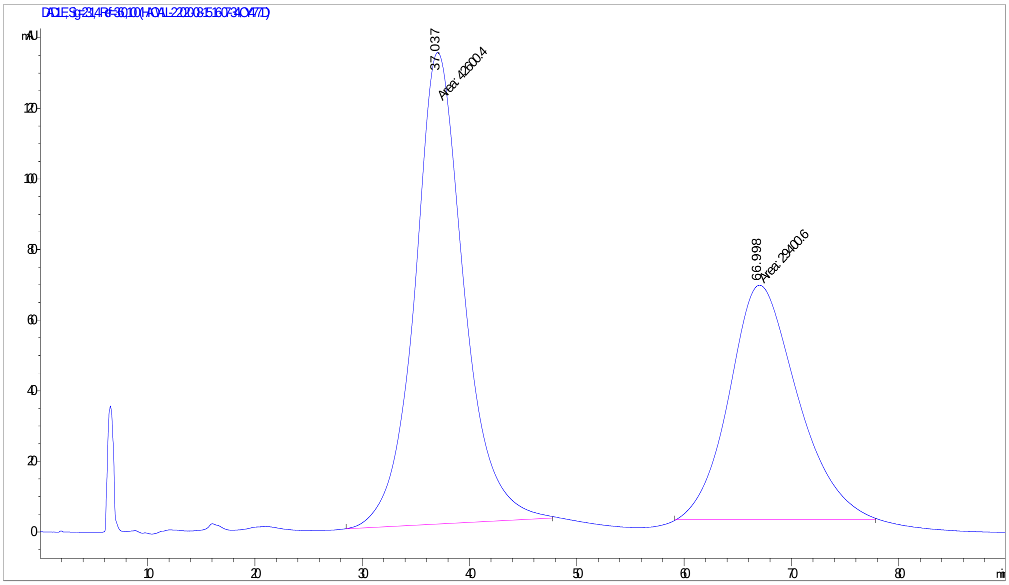

#	Time	Area	Height	Width	Area%	Symmetry
1	37.037	42600.4	133.6	5.3143	59.166	0.862
2	66.998	29400.6	66.4	7.381	40.834	0.767

Racemic mono cycloaddition product ***rac*-4a** was treated with 1.1 equiv. of iminoester **2g**. The course of reaction was observed over time. Two different products were observed, opposite enantiomer *endo, endo* **(-)3e** and diastereomer *endo, exo* **6b** with varying ratio and change in enantioselectivity over time. Both products were isolated after certain time points and enantiomeric excess was determined by HPLC analysis on chiral phase.

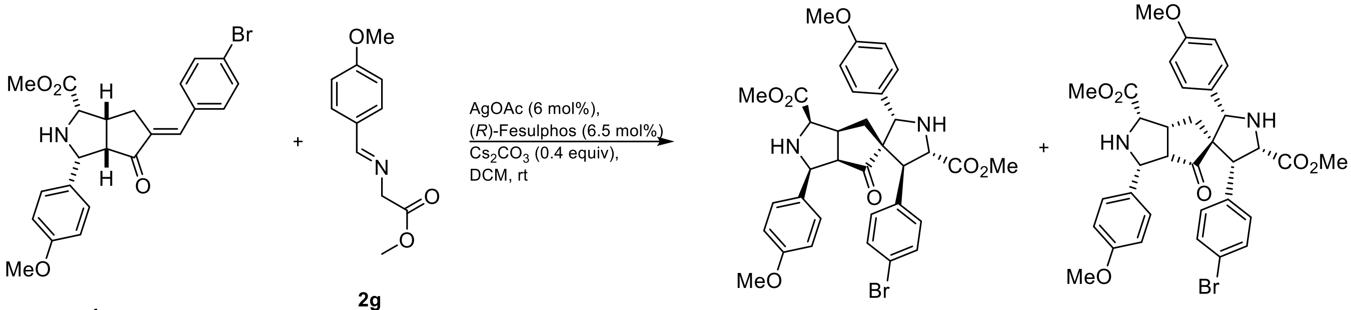

rac*-4c** **2g** **1.1 equiv.** **(-)3e** ***endo, endo **6b** ***endo, exo***

HPLC traces of enantiomer **(-)3e** after 3 min

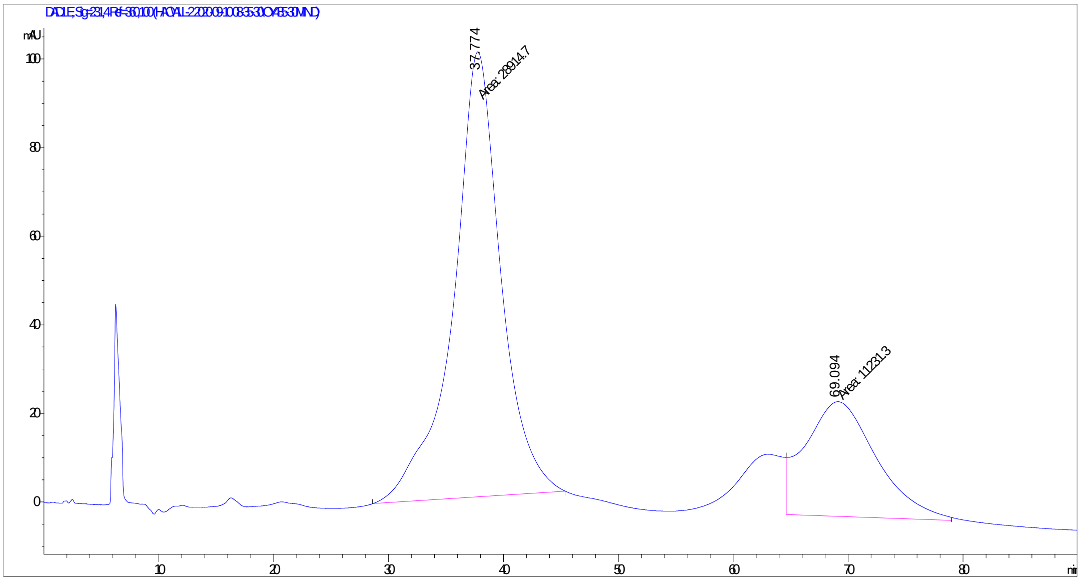

#	Time	Area	Height	Width	Area%	Symmetry
1	37.774	28914.7	100.5	4.797	72.024	1.05
2	69.094	11231.3	25.9	7.2254	27.976	0.816

HPLC traces of diastreomer **6b** after 3 min

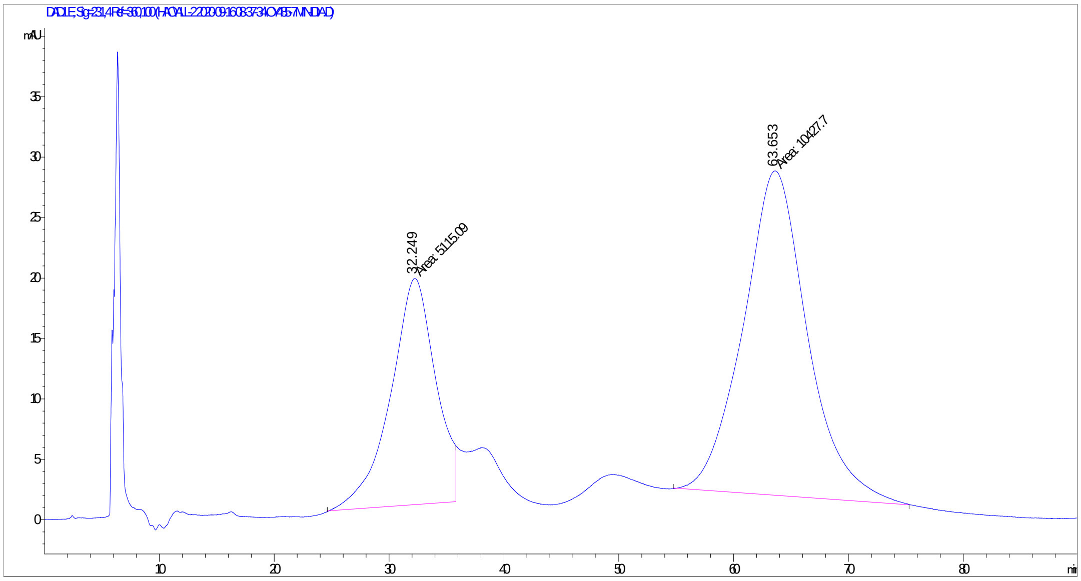

#	Time	Area	Height	Width	Area%	Symmetry
1	32.249	3870.4	16.2	3.9939	27.070	1.259
2	63.653	10427.7	26.8	6.4768	72.930	0.944

HPLC traces of enantiomer **(-)3e** after 7 min

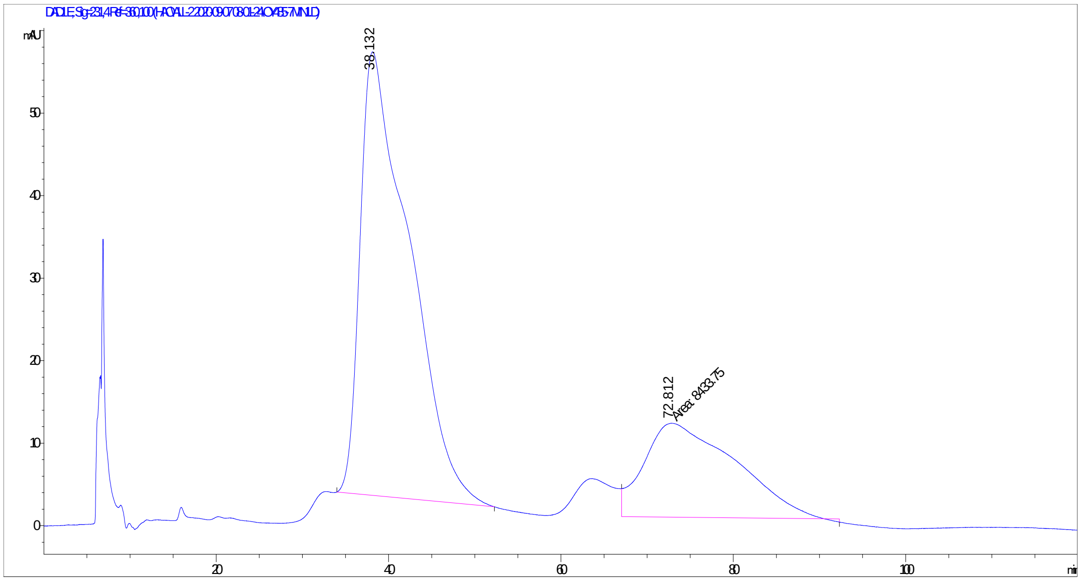

#	Time	Area	Height	Width	Area%	Symmetry
1	38.132	21398	53.7	4.6589	71.729	0.331
2	72.812	8433.8	11.4	12.3469	28.271	0.426

HPLC traces of diastereomer **6b** after 7 min

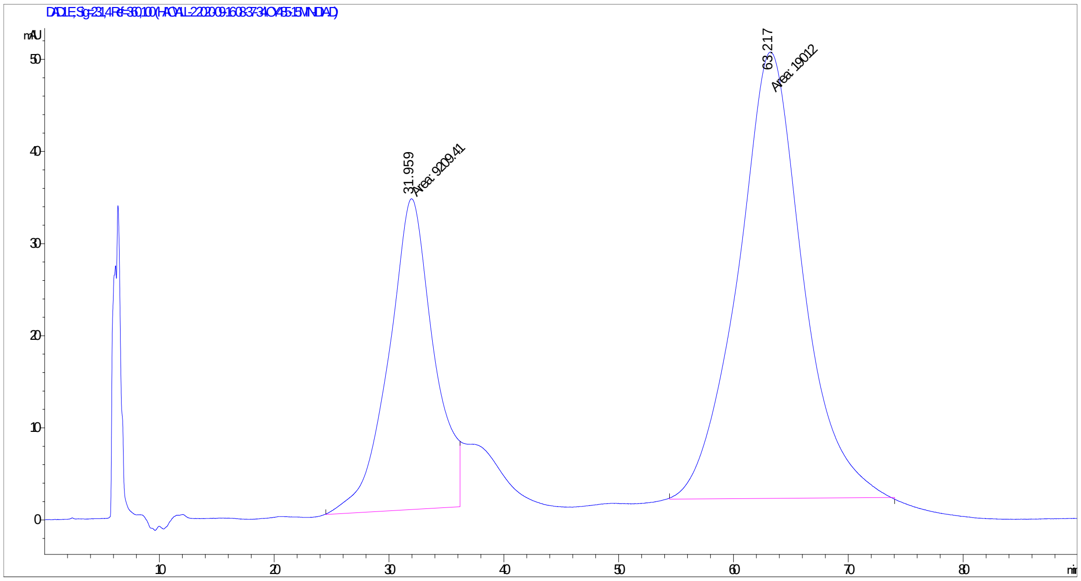

#	Time	Area	Height	Width	Area%	Symmetry
1	31.959	9209.4	33.7	4.549	32.633	1.019
2	63.217	19012	48.4	6.5407	67.367	0.975

HPLC traces of enantiomer **(-)3e** after 15 min

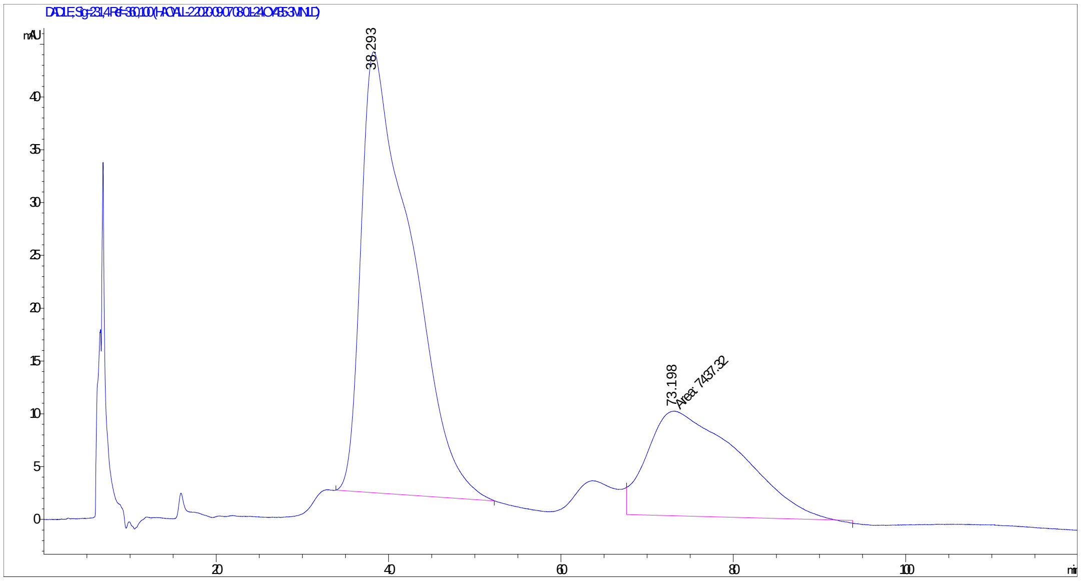

#	Time	Area	Height	Width	Area%	Symmetry
1	38.293	16861.3	41.7	4.7405	69.392	0.337
2	73.198	7437.3	9.9	12.5048	30.608	0.416

HPLC traces of diastereomer **6b** after 15 min

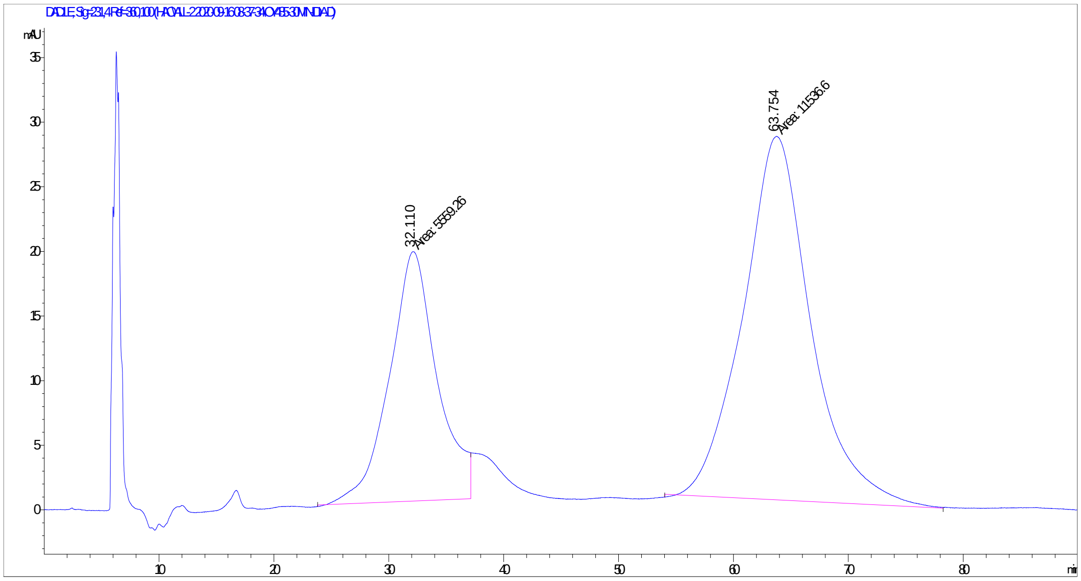

#	Time	Area	Height	Width	Area%	Symmetry
1	32.11	5559.3	19.3	4.8004	32.518	0.961
2	63.754	11536.6	28.1	6.8361	67.482	0.94

HPLC traces of enantiomer **(-)3e** after 30 min

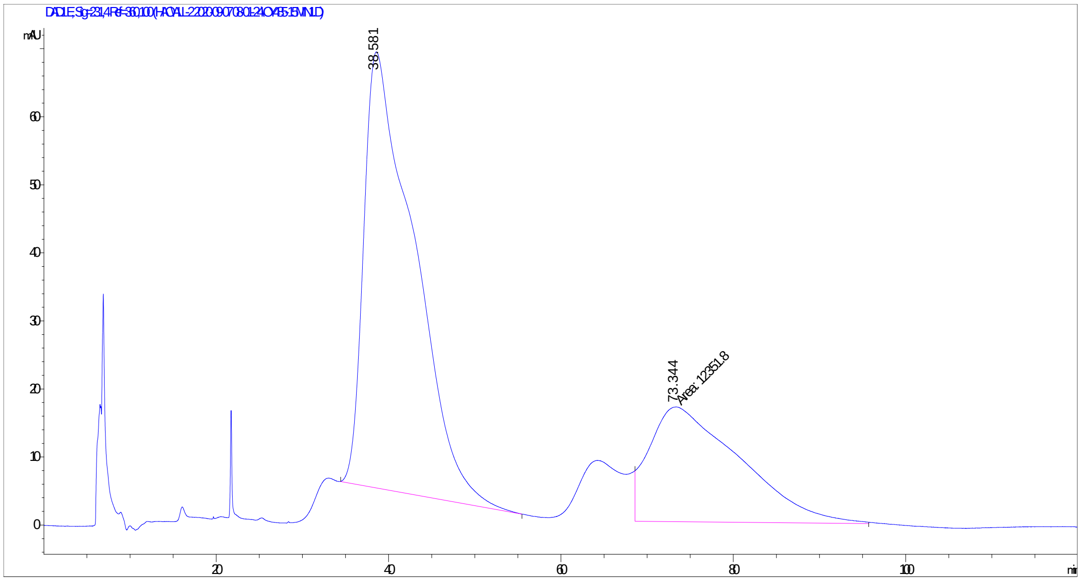

#	Time	Area	Height	Width	Area%	Symmetry
1	38.581	26134.6	64.1	4.7659	67.906	0.328
2	73.344	12351.8	16.9	12.205	32.094	0.411

HPLC traces of diastereomer **6b** after 15 min

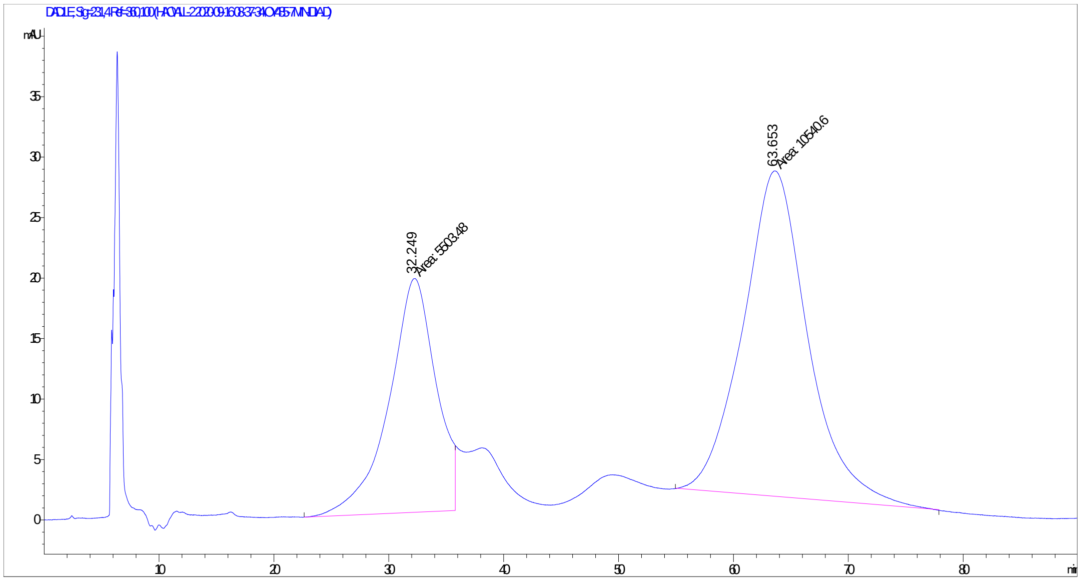

#	Time	Area	Height	Width	Area%	Symmetry
1	32.249	5503.5	19.3	4.7432	34.302	1.191
2	63.653	10540.6	26.9	6.5301	65.698	0.929

HPLC traces of enantiomer **(-)3e** after 1h

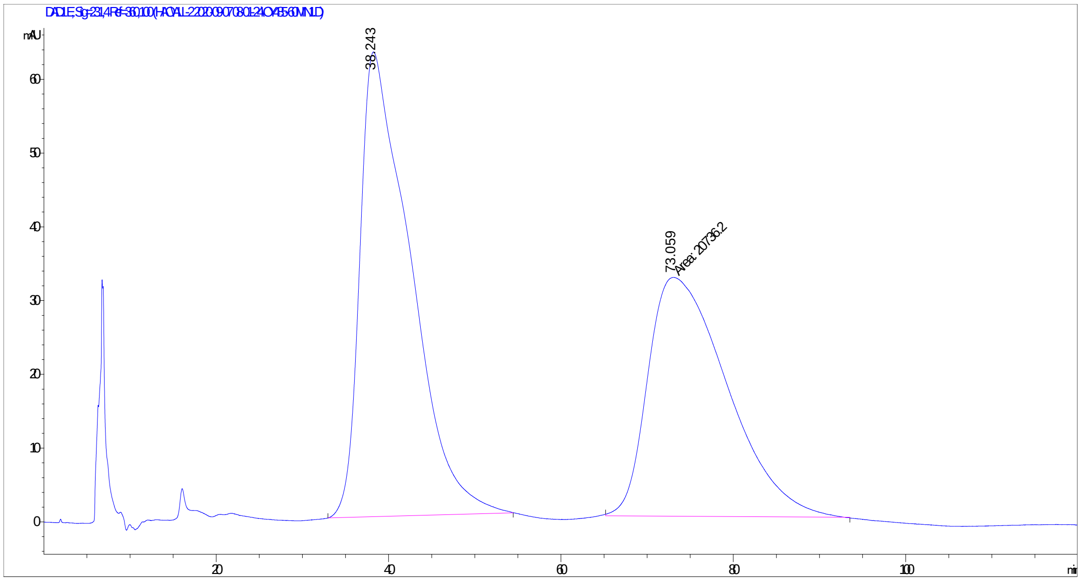

#	Time	Area	Height	Width	Area%	Symmetry
1	38.243	25631.3	63	4.7588	55.279	0.366
2	73.059	20736.2	32.4	10.674	44.721	0.47

Highly enantioenriched *endo, exo* **6a** (95% e.e.) was treated under chiral conditions and converted to *endo, endo* **(+) 3a.** Product was isolated and enantiomeric excess was determined by HPLC analysis on chiral phase.

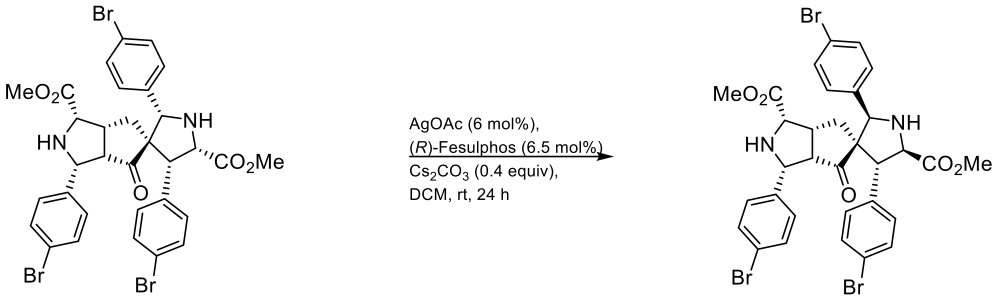

6a
endo, exo
95% e.e.

(+)3a
endo, endo
92%, 95% e.e.

HPLC traces of enantiomer *endo, exo* **6a (SM)** on chiral **IA**-column

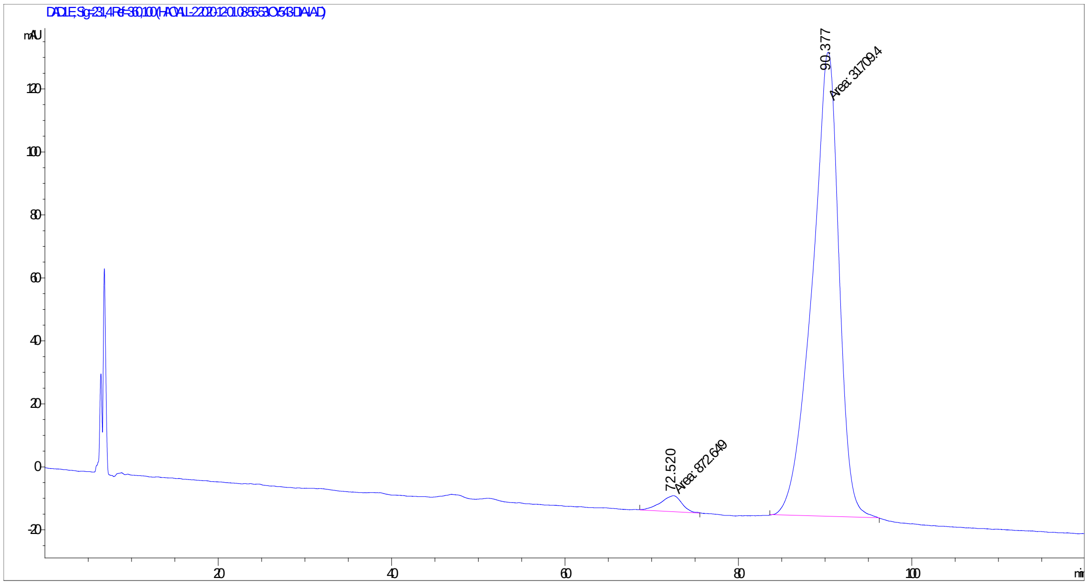

#	Time	Area	Height	Width	Area%	Symmetry
1	72.52	872.6	5.1	2.8608	2.678	1.482
2	90.377	31709.4	147.4	3.5865	97.322	1.451

HPLC traces of enantiomer *endo, endo* **(+)3a** on chiral **IA**-column

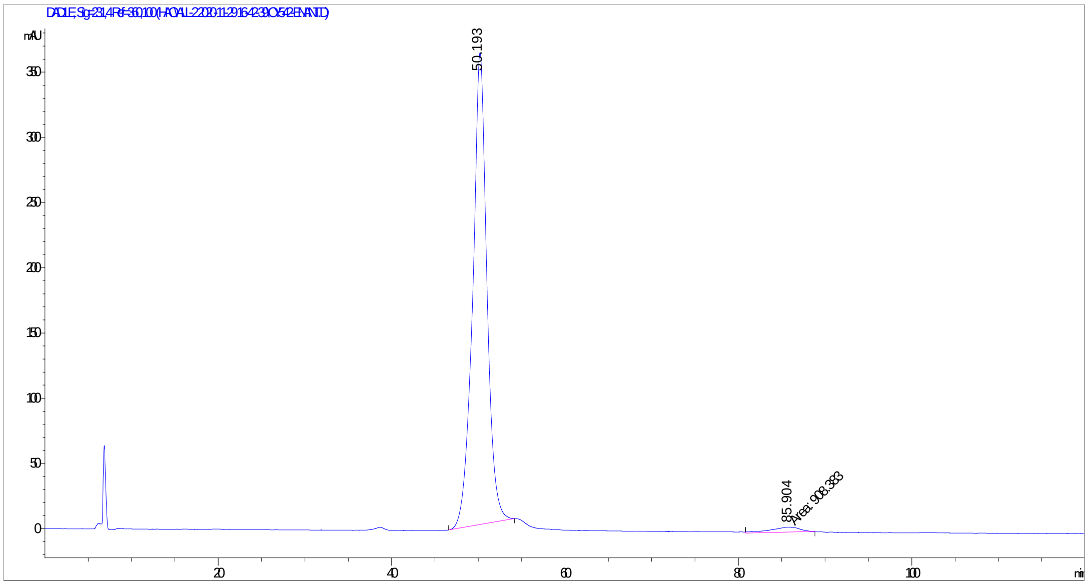

#	Time	Area	Height	Width	Area%	Symmetry
1	50.193	41127.6	362	1.6195	97.839	1.036
2	85.904	908.4	3.8	4.0007	2.161	1.982

HPLC traces of racemic *endo, endo* **(+)3a** on chiral **IA**-column

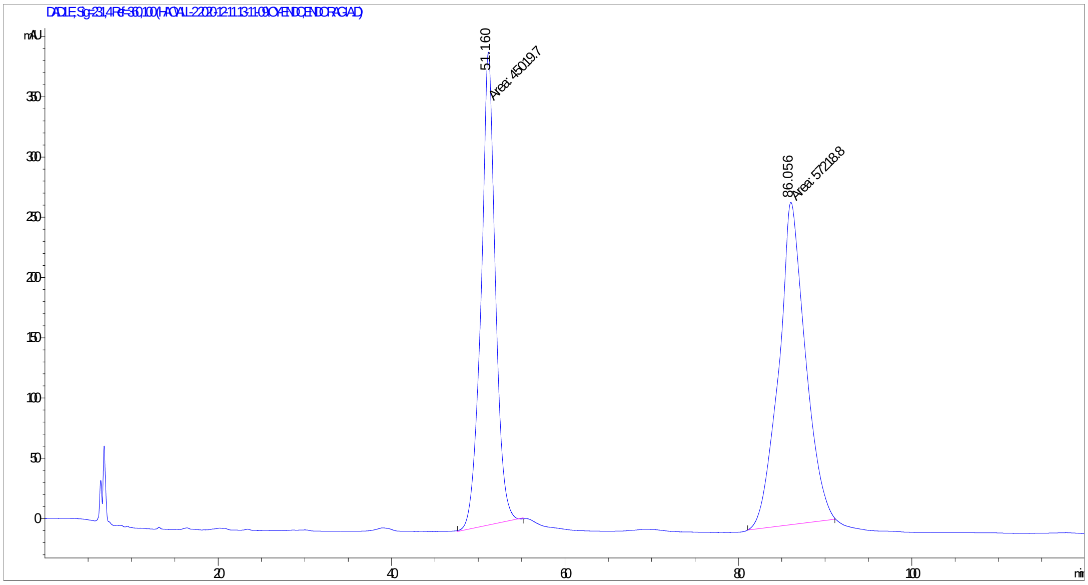

#	Time	Area	Height	Width	Area%	Symmetry
1	51.16	48163.6	397	2.022	49.107	0.919
2	86.056	49914.9	253.9	3.2762	50.893	0.873

Slightly enantioenriched *endo, exo* **6a** (37% e.e.) was treated under chiral conditions and converted to *endo, endo* **(+) 3a.** Both products were isolated and enantiomeric excess was determined by HPLC analysis on chiral phase

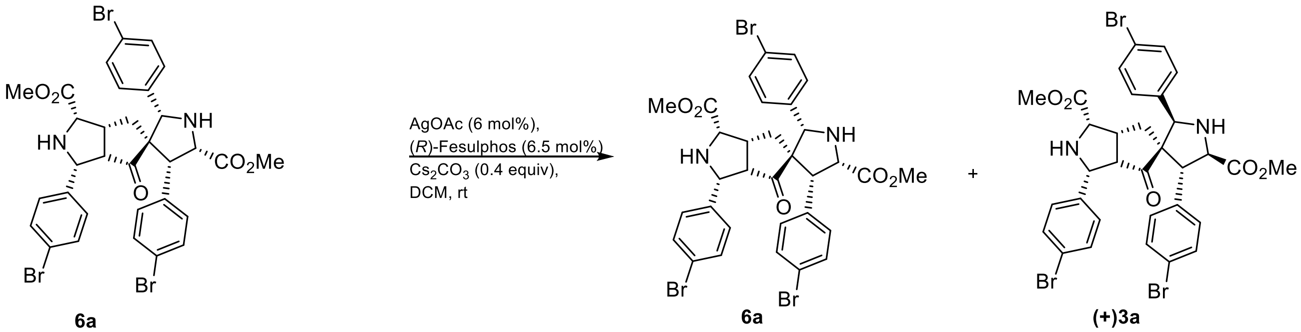

6a *endo, exo* 37% e.e. → **6a** *endo, exo* 51% (recovered), 20% e.e. + **(+)3a** *endo, endo* 37%, 59% e.e.

HPLC traces of *endo, exo* **6a** on chiral IC-column

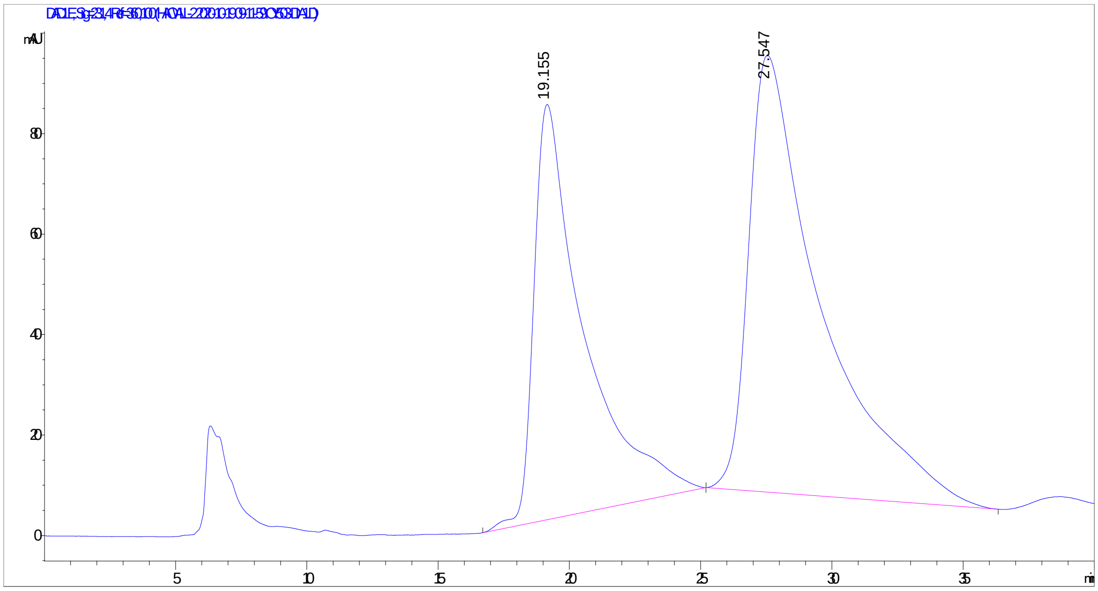

#	Time	Area	Height	Width	Area%	Symmetry
1	19.155	10903.9	82.6	1.7713	39.801	0.343
2	27.547	16492.4	86.9	2.5531	60.199	0.333

HPLC traces of enantiomer *endo, endo* (+)**3a**

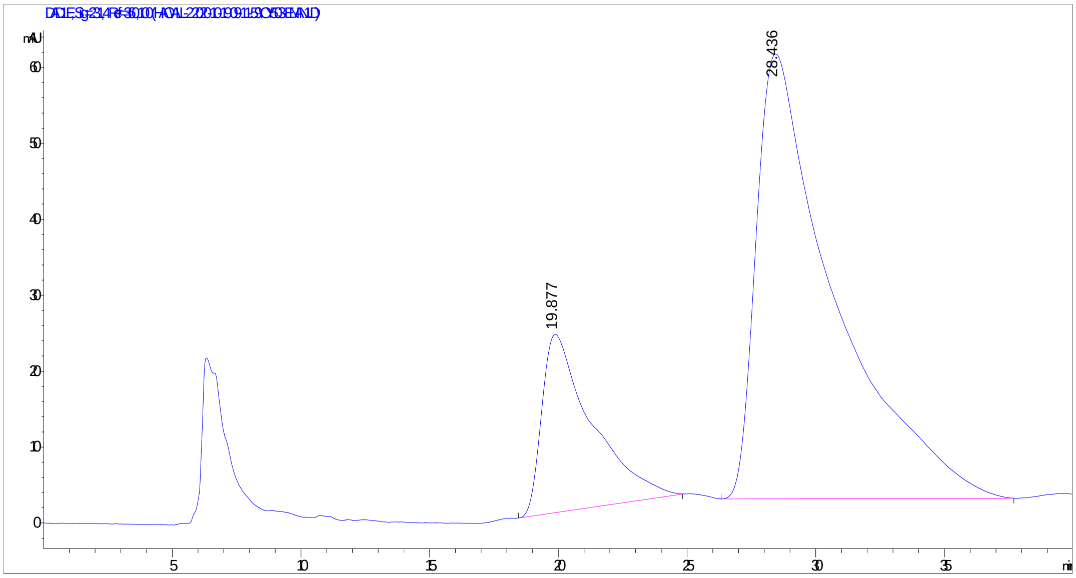

#	Time	Area	Height	Width	Area%	Symmetry
1	19.877	3161	23.5	1.6441	20.295	0.358
2	28.436	12414.2	58.6	2.7557	79.705	0.304

Enantioenriched *endo, exo* **6a** (37% e.e.) was treated with iminoester **2g**.and converted to imine exchanged product *endo, endo* **5k.** Product was isolated and enantiomeric excess was determined by HPLC analysis on chiral phase

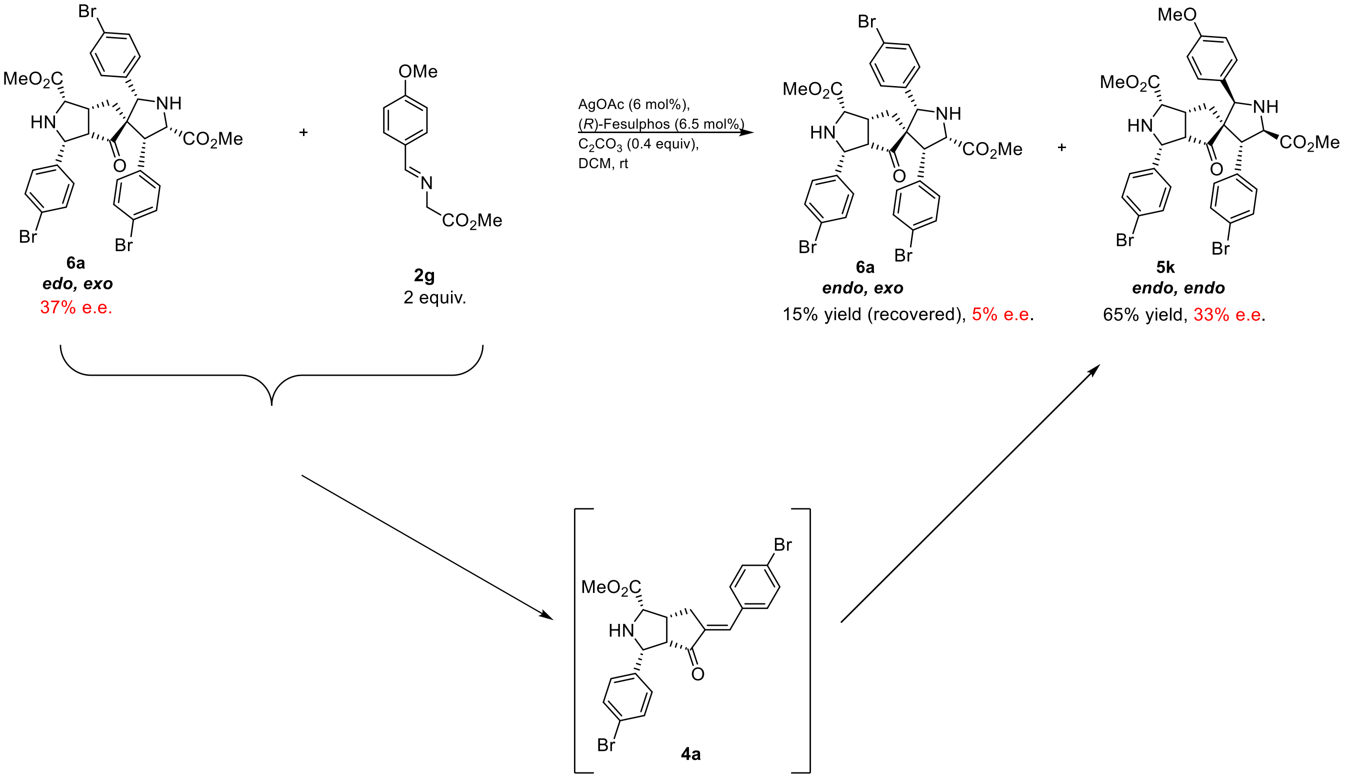

Imine exchange through mono adduct as intermediate

HPLC traces of enantiomer **5p**

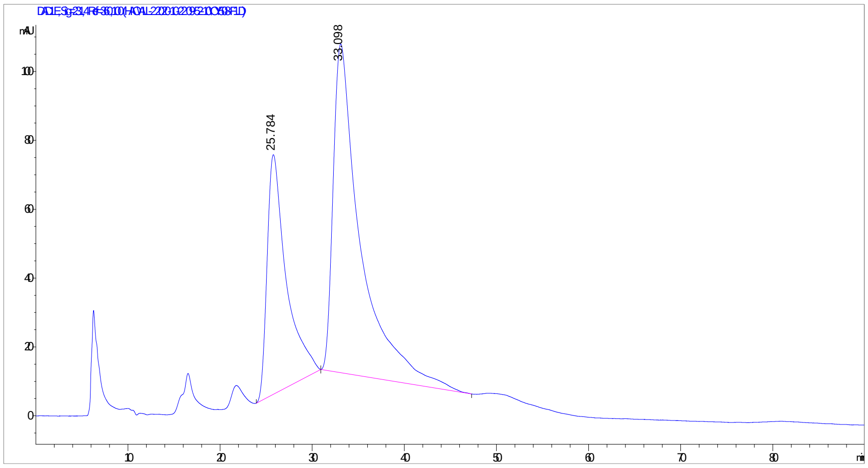

#	Time	Area	Height	Width	Area%	Symmetry
1	25.784	10076.8	69.6	2.0076	33.880	0.447
2	33.098	19666	95.5	2.7229	66.120	0.352

Racemic double cycloaddition product *endo, endo* ***rac*-3a** was treated with iminoester **2a** in order to investigate if it can be converted to its stereoisomer *endo, exo* **6a.** Racemic double cycloaddition product ***rac*-3a** was not able to be converted to diastereomer **6a** and was recovered as racemic product again.

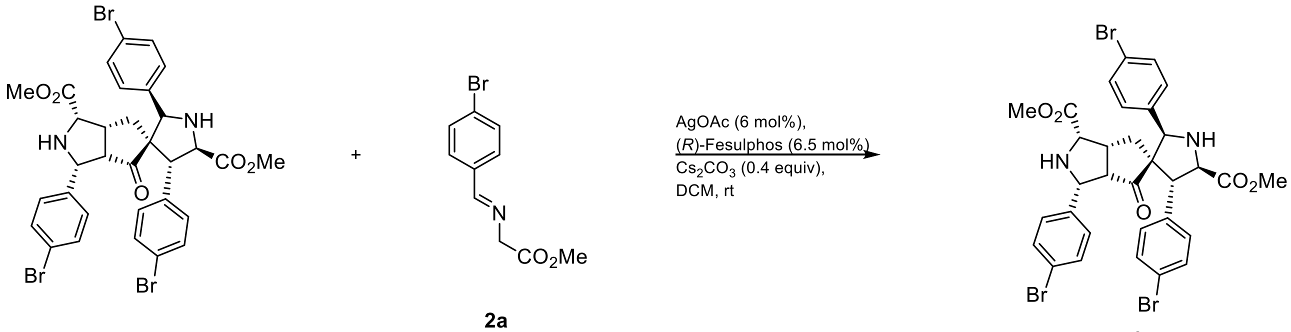

rac-3a + **2a** 2 equiv. → **rac-3a** 100% yield

HPLC traces of recovered racemic ***rac*-3a**

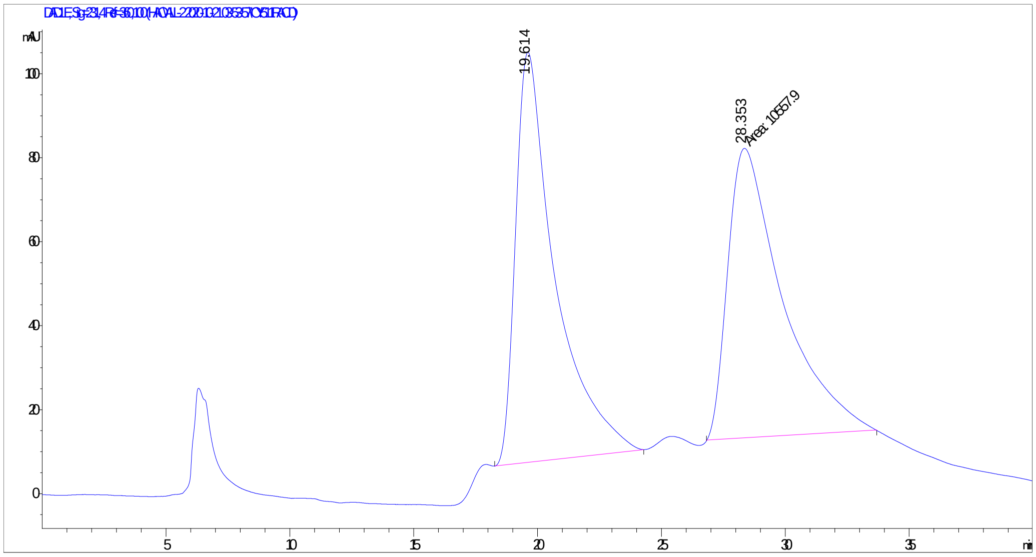

#	Time	Area	Height	Width	Area%	Symmetry
1	19.614	10826.2	97.7	1.565	50.627	0.416
2	28.353	10557.9	68.9	2.5536	49.373	0.426

Crystal data and structure for (+)3a

CCDC Number: 1952338

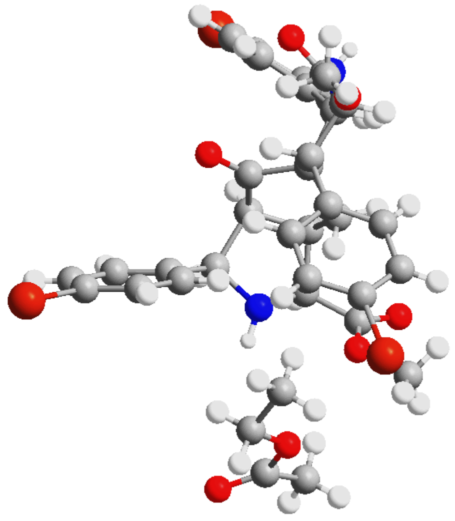

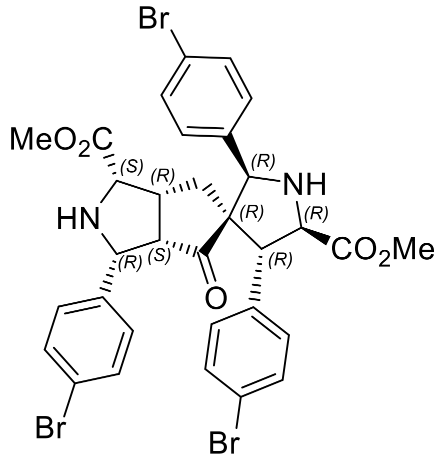

3a

Compound	3a
Formula	$C_{32}H_{29}Br_3N_2O_5$
Formula weight [g·mol^{-1}]	761.29
Temperature [K]	100
Wave length [Å]	0.71073
Space group	P-21 21 21
Cell dimensions	
a, *b*, *c* (Å)	9.7769, 10.2614, 34.8336
α, β, γ (°)	90, 90, 90
Volume [Å^3]	3494.7 (3)
Z	4
R-Factor%	3.03
Calc. density [Mg·m^3]	1.614
Θ(max) [°]	30.99
Θ(min) [°]	2.5
F(000)	1712
Index ranges	- 14 ≤ h ≤ 14
	- 14 ≤ k ≤ 14
	-50 ≤ l ≤ 50
Reflections collected	11129
Independent reflections	10791 [R(int) = 0.027]
$R[F2 > 2\sigma(F2)]$	0.027
$wR(F2)$	0.0725
S	1.086

Crystal data and structure for (+)3e

CCDC Number: 2042023

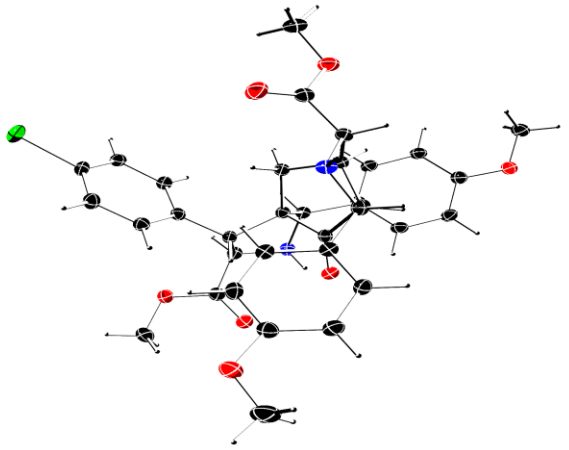

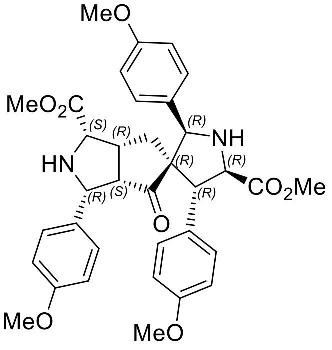

(+)3e

Compound	(+)3e
Formula	$C_{34}H_{35}BrN_2O_7$
Formula weight [g·mol^{-1}]	663.55
Temperature [K]	100
Wave length [Å]	0.71073
Space group	P-21 21 21
Cell dimensions	
a, *b*, *c* (Å)	13.4421, 13.6093, 16.705
α, β, γ (°)	90, 90, 90
Volume [Å^3]	3056.0 (6)
Z	4
R-Factor%	3.03
Calc. density [Mg·m^3]	1.442
Θ(max) [°]	28.999
Θ(min) [°]	2.5
F(000)	1376
Index ranges	- 18 ≤ h ≤ 18
	- 18 ≤ k ≤ 18
	-22 ≤ l ≤ 22
Reflections collected	8127
Independent reflections	7792 [R(int) = 0.0265]
$R[F2 > 2\sigma(F2)]$	0.0265
$wR(F2)$	0.0709
S	1.053

Crystal data and structure for (-)3e

CCDC Number: 2042025

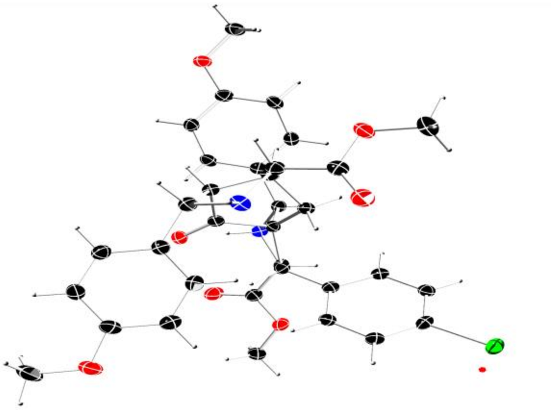

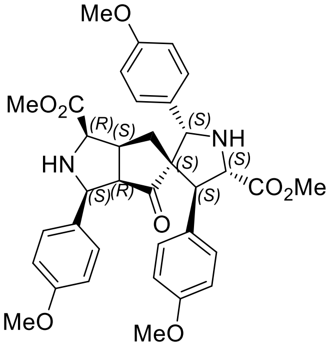

(-)3e

Compound	(-3)e
Formula	$C_{34}H_{35}BrN_2O_7$
Formula weight [g·mol^{-1}]	663.54
Temperature [K]	100
Wave length [Å]	0.71073
Space group	P-21 21 21
Cell dimensions	
a, *b*, *c* (Å)	13.437, 13.616, 16.674
α, β, γ (°)	90, 90, 90
Volume [Å^3]	3051(3)
Z	4
R-Factor%	3.03
Calc. density [Mg·m^3]	1.445
Θ(max) [°]	27.947
Θ(min) [°]	2.5
F(000)	1376
Index ranges	- 17 ≤ h ≤ 17
	- 17 ≤ k ≤ 17
	-21 ≤ l ≤ 21
Reflections collected	7292
Independent reflections	6501 [R(int) = 0.0317]
R[*F*2 > 2σ(*F*2)]	0.0317
wR(*F*2)	0.0699
S	1.028

Crystal data and structure for 6a

CCDC Number: 2042027

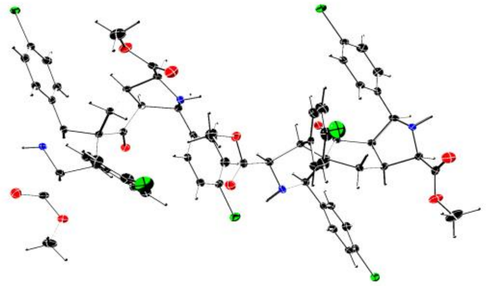

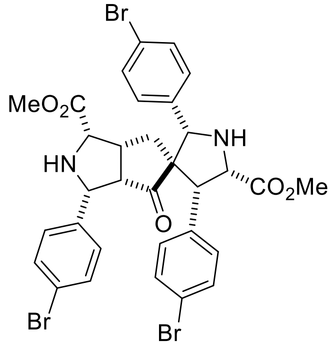

6a

Compound	6a
Formula	$C_{32}H_{29}Br_3N_2O_5$
Formula weight [g·mol^{-1}]	761.27
Temperature [K]	100
Wave length [Å]	0.71073
Space group	P -1
Cell dimensions	
a, *b*, *c* (Å)	11.2514, 15.8026, 19.7865
α, β, γ (°)	80.520, 81.241, 89.927
Volume [Å^3]	3428.5
Z	4
R-Factor%	3.03
Calc. density [Mg·m^3]	1.475
Θ(max) [°]	27.000
Θ(min) [°]	2.5
F(000)	1520.0
Index ranges	- 14 ≤ h ≤ 14
	- 20 ≤ k ≤ 20
	-25 ≤ l ≤ 25
Reflections collected	14970
Independent reflections	14219[R(int) = 0.0424]
R[*F*2 > 2σ(*F*2)]	0.0424
wR(*F*2)	0.1066
S	1.110

Crystal data and structure for 6b

CCDC Number: 2042026

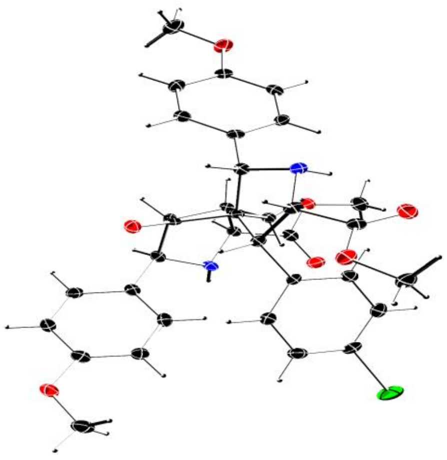

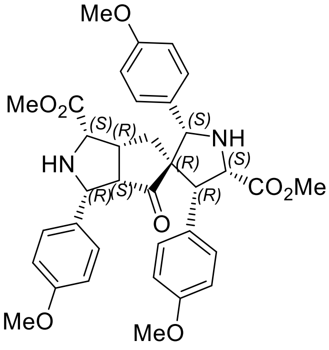

6b

Compound	6b
Formula	$C_{34}H_{35}BrN_2O_7$
Formula weight [g·mol^{-1}]	663.54
Temperature [K]	100
Wave length [Å]	0.71073
Space group	P-61
Cell dimensions	
a, *b*, *c* (Å)	11.267, 11.267, 47.013
α, β, γ (°)	90, 90, 120
Volume [Å^3]	5169
Z	6
R-Factor%	3.03
Calc. density [Mg·m^3]	1.391
Θ(max) [°]	25.996
Θ(min) [°]	2.5
F(000)	2256
Index ranges	- 13 ≤ h ≤ 13
	- 13 ≤ k ≤ 13
	-57 ≤ l ≤ 57
Reflections collected	6766
Independent reflections	6157[R(int) = 0.0329]
R[*F*2 > 2σ(*F*2)]	0.0329
wR(*F*2)	0.0657
S	1.046

References

[1] Takanami, T. *et al.* Utilization of PPh3-Ti(IV) reagents. An efficient α'-alkylidenation of cyclic enones. *Tetrahedron Lett.* **31,** 677-680 (1990).

[2] Costas, M. *et al.* Iron catalyzed highly enantioselective epoxidation of cyclic aliphatic enones with aqueous H_2O_2. *J. Am. Chem. Soc.* **138,** 2732–2738 (2016).

[3]. J. M. Longmire, B. Wang and X. Zhang, Highly Enantioselective Ag(I)-catalyzed [3+2] Cycloaddition of Azomethine Ylides. *J. Am. Chem. Soc.* **124**, 13400–13401 (2002).

[4] Cabrera, S. *et al*. CuI-Fesulphos complexes: efficient chiral catalysts for asymmetric 1, 3-dipolar cycloaddition of azomethine ylides. *Tetrahedron Lett.* **63***,* 6587-6602 (2007).

[5] López-Pérez, A. *et al.* The Phenylsulfonyl Group as a Temporal Regiochemical Controller in the Catalytic Asymmetric 1, 3-Dipolar Cycloaddition of Azomethine Ylides. *Angew. Chem. Int. Ed.* **48**, 340-343 (2009)

[6] Seashore-Ludlow, B. *et al*. Addition of Azomethine Ylides to Aldehydes: Mechanistic Dichotomy of Differentially Substituted α-Imino Esters. *Eur. J. Org. Chem.* **20,** 3927-3933 (2010).

Copies of spectral data

^{1}H NMR

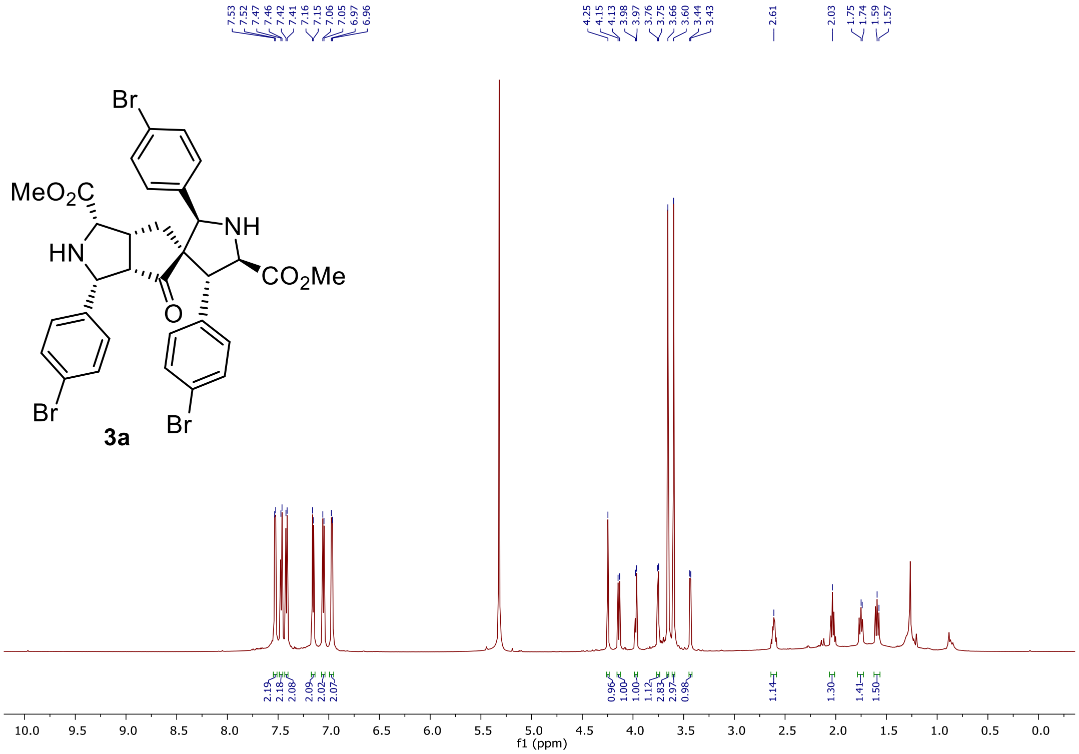

^{13}C NMR

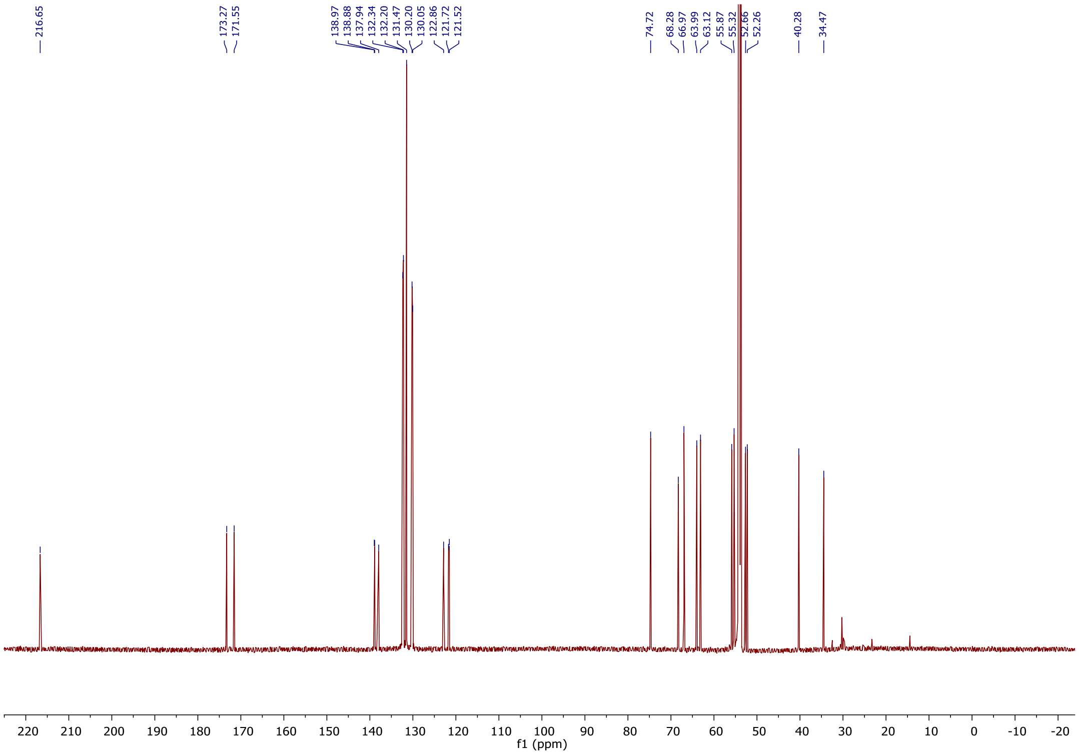

HPLC traces for **3a**: racemic top, enantiomer 1 bottom

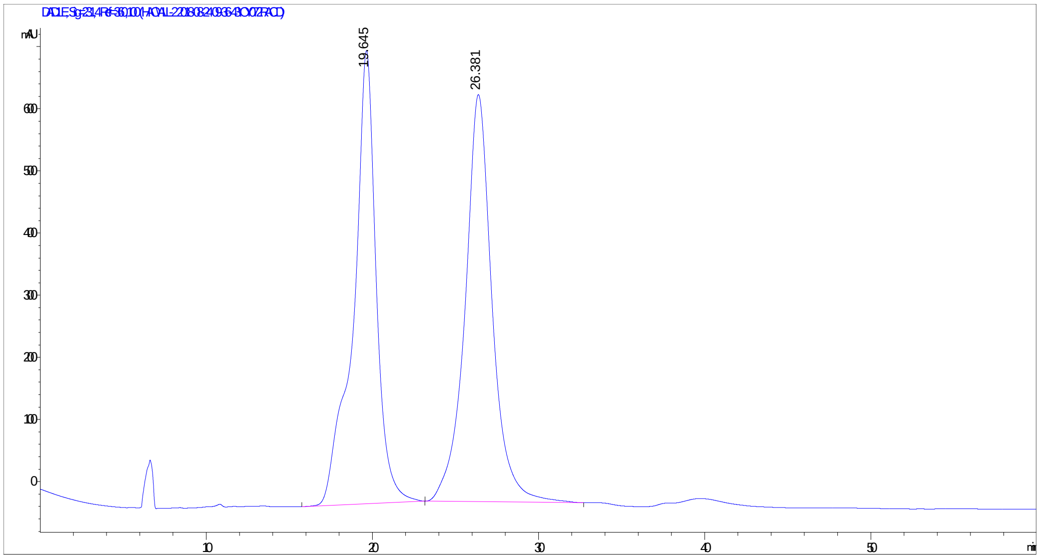

#	Time	Area	Height	Width	Area%	Symmetry
1	19.645	69686.1	729.6	1.3464	48.371	1.34
2	26.381	74380.5	655.4	1.656	51.629	0.932

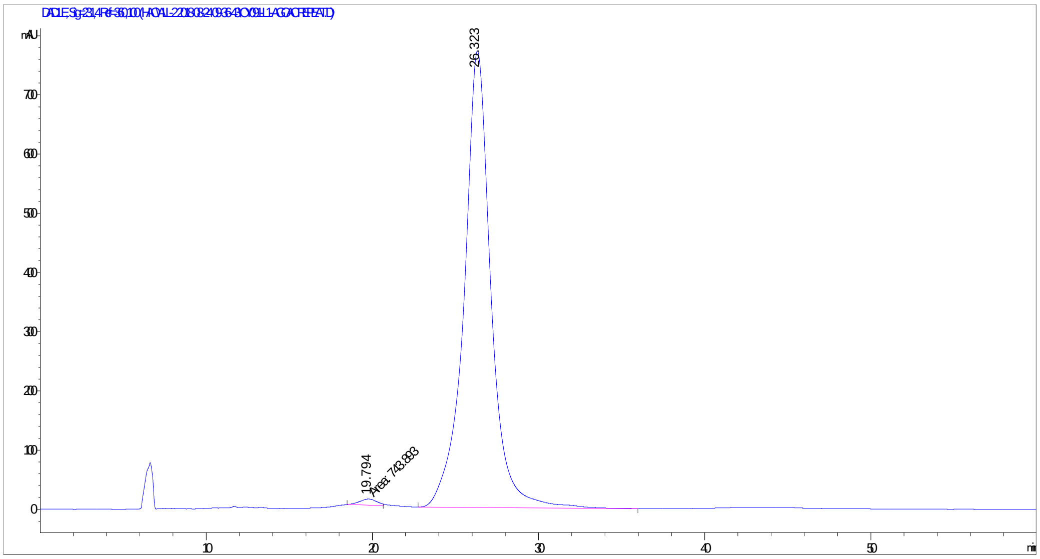

#	Time	Area	Height	Width	Area%	Symmetry
1	19.794	743.9	10.4	1.1902	0.851	0.966
2	26.323	86685	771.2	1.6334	99.149	0.95

^{1}H NMR

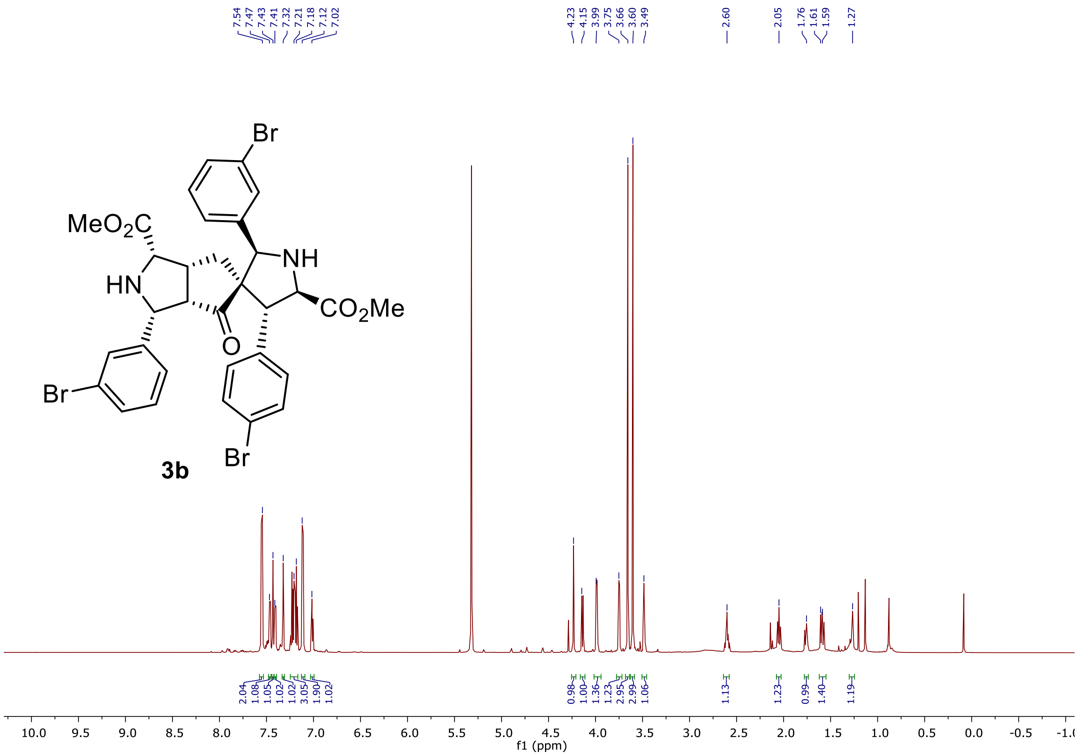

^{13}C NMR

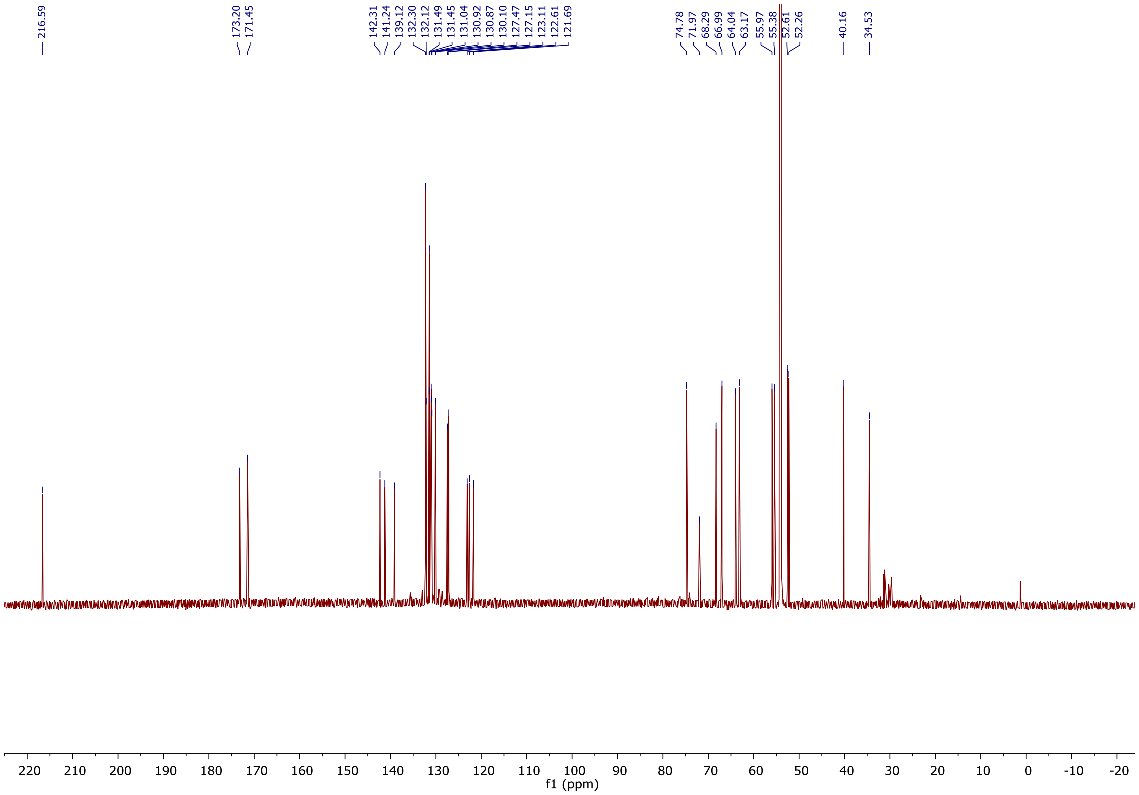

HPLC traces for **3b**: racemic top, enantiomer 1 bottom

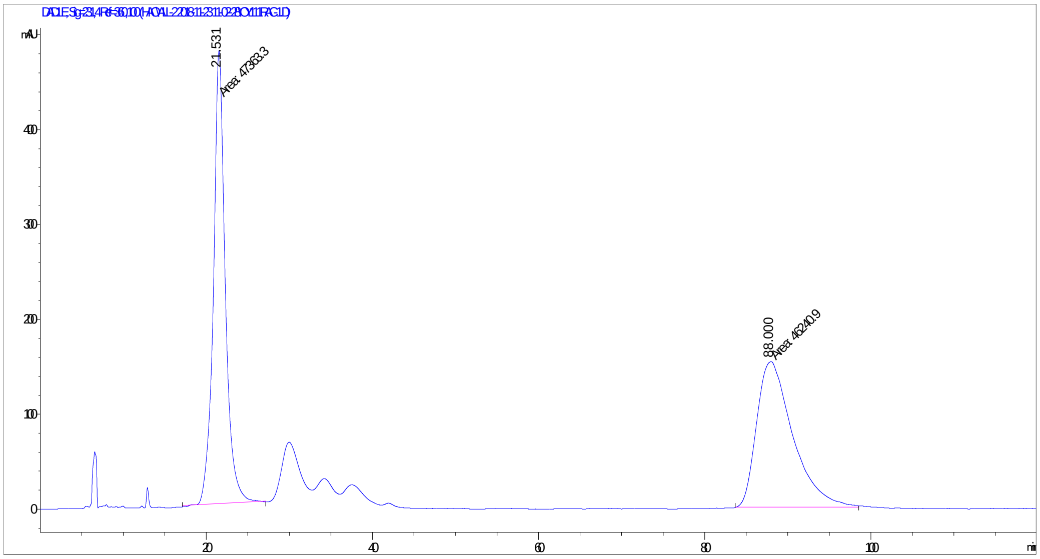

#	Time	Area	Height	Width	Area%	Symmetry
1	21.531	47363.3	477.6	1.6527	50.600	0.861
2	88	46240.9	153.2	5.031	49.400	0.638

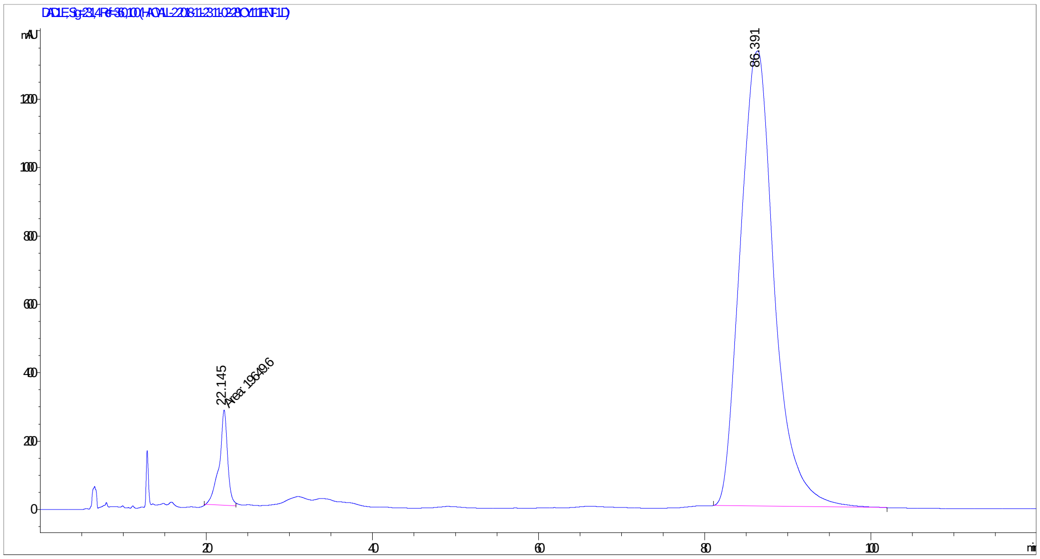

#	Time	Area	Height	Width	Area%	Symmetry
1	22.145	19649.6	278.5	1.1759	4.929	1.36
2	86.391	378987.2	1331.1	3.5357	95.071	1.011

^{1}H NMR

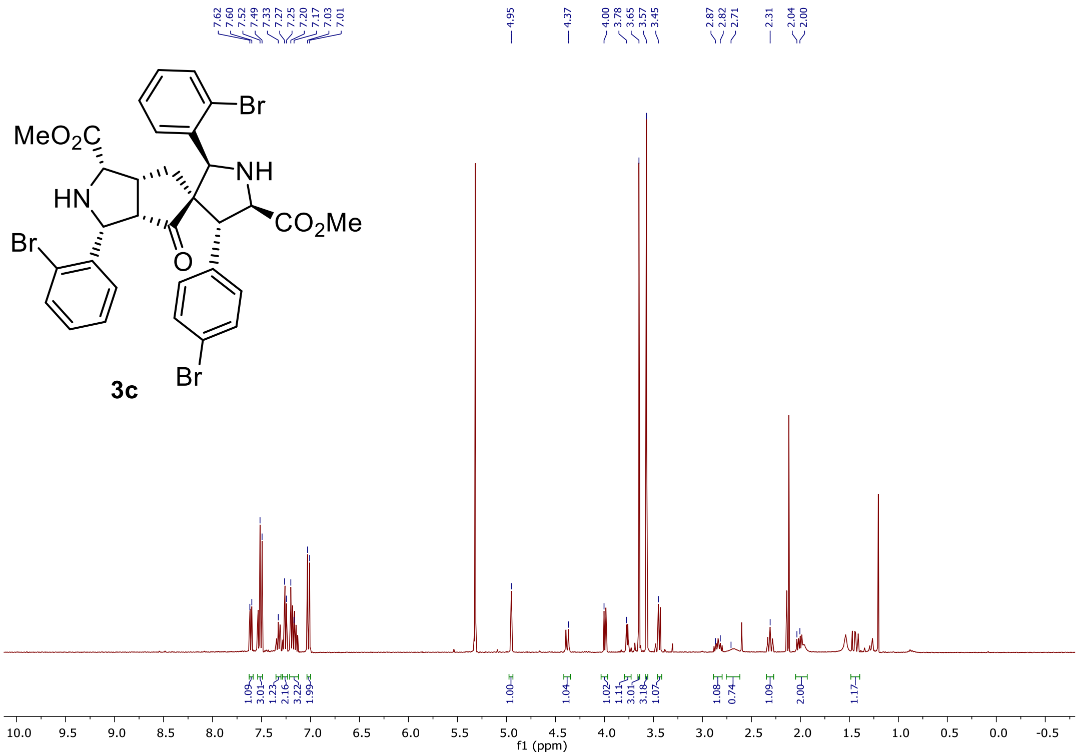

^{13}C NMR

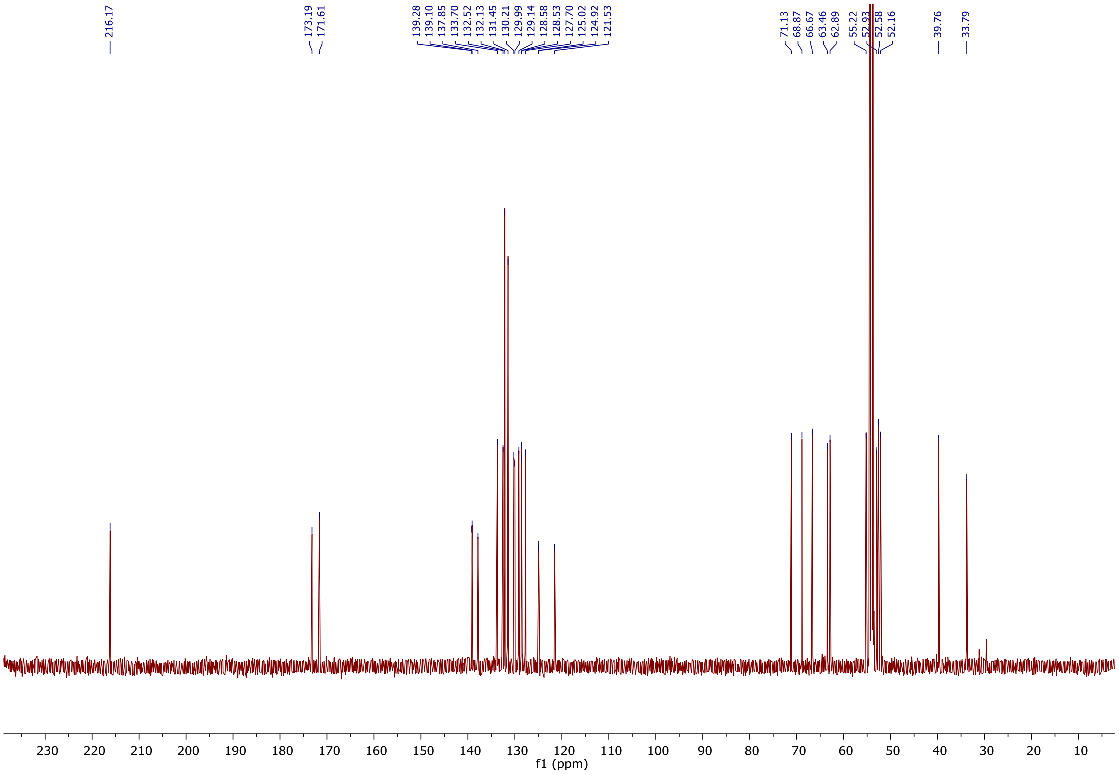

^{1}H NMR HPLC traces for **3c**: racemic top, enantiomer 1 bottom

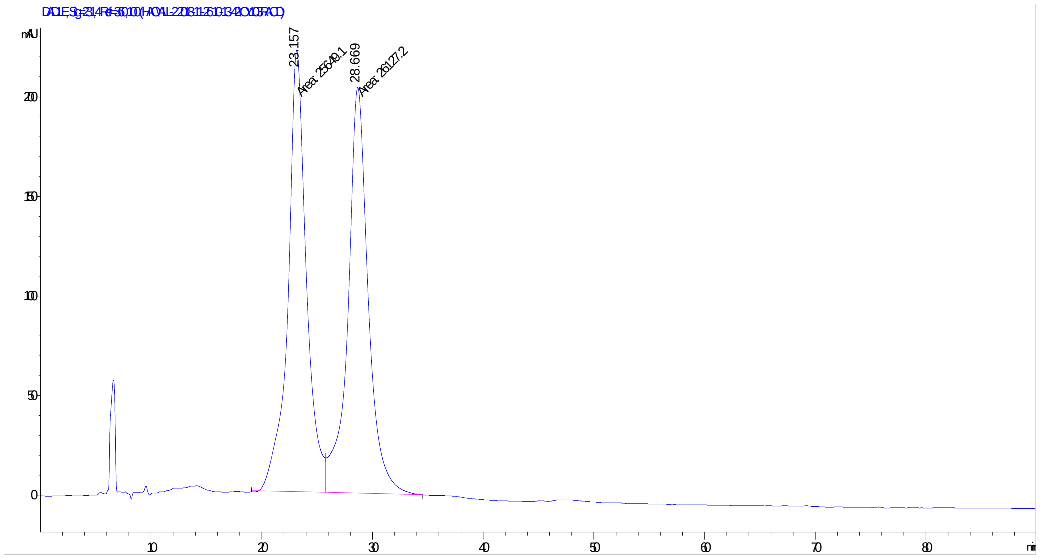

#	Time	Area	Height	Width	Area%	Symmetry
1	23.157	25649.1	221.5	1.9295	49.538	0.901
2	28.669	26127.2	203.7	2.1376	50.462	0.92

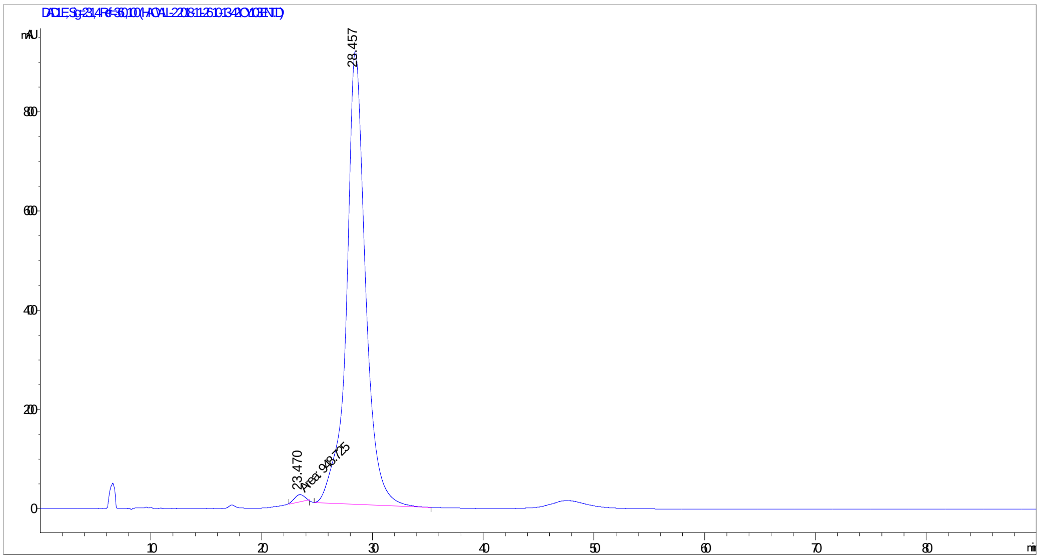

#	Time	Area	Height	Width	Area%	Symmetry
1	23.47	948.7	14.8	1.0699	0.862	1.447
2	28.457	109162.8	913.5	1.7302	99.138	0.864

^{1}H NMR

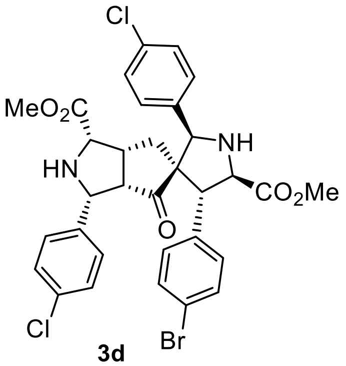

3d

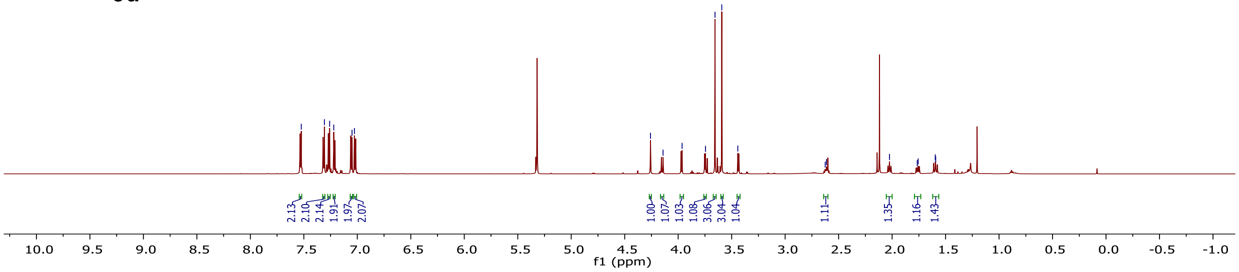

^{13}C NMR

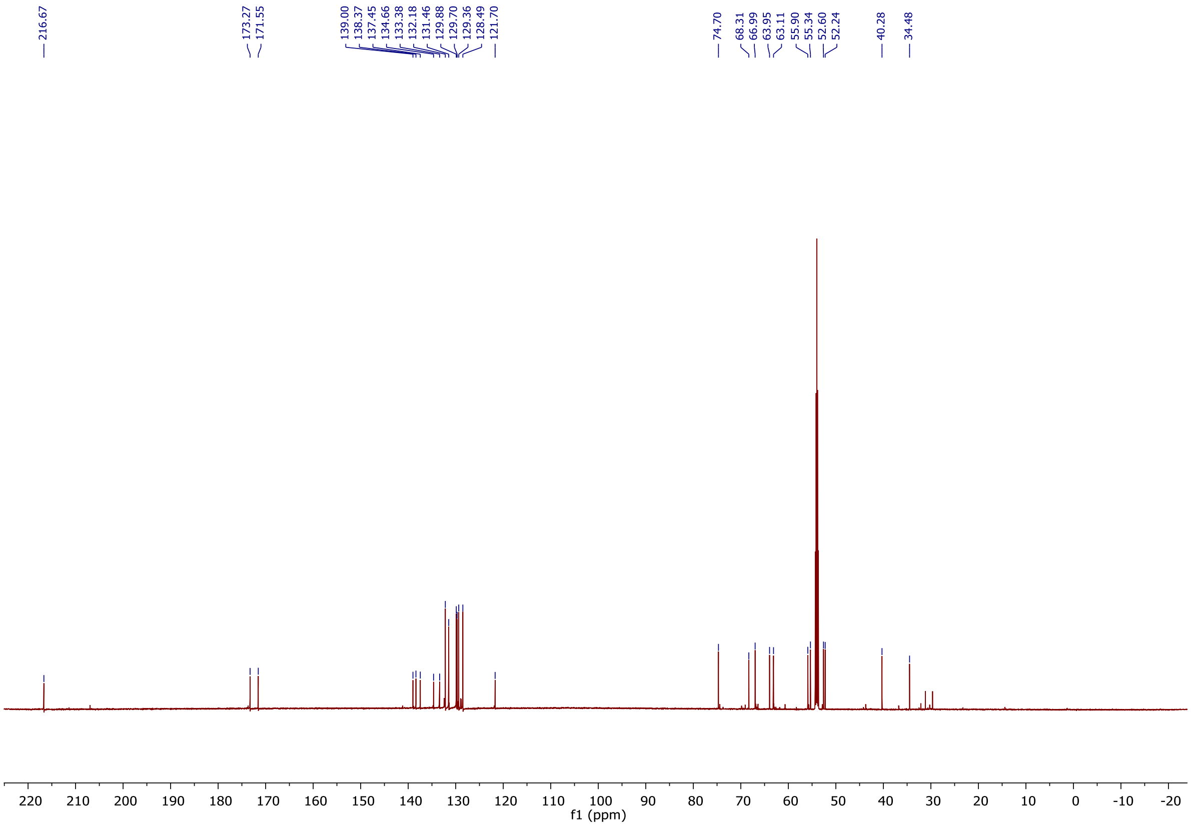

HPLC traces for **3d**: racemic top, enantiomer 1 bottom

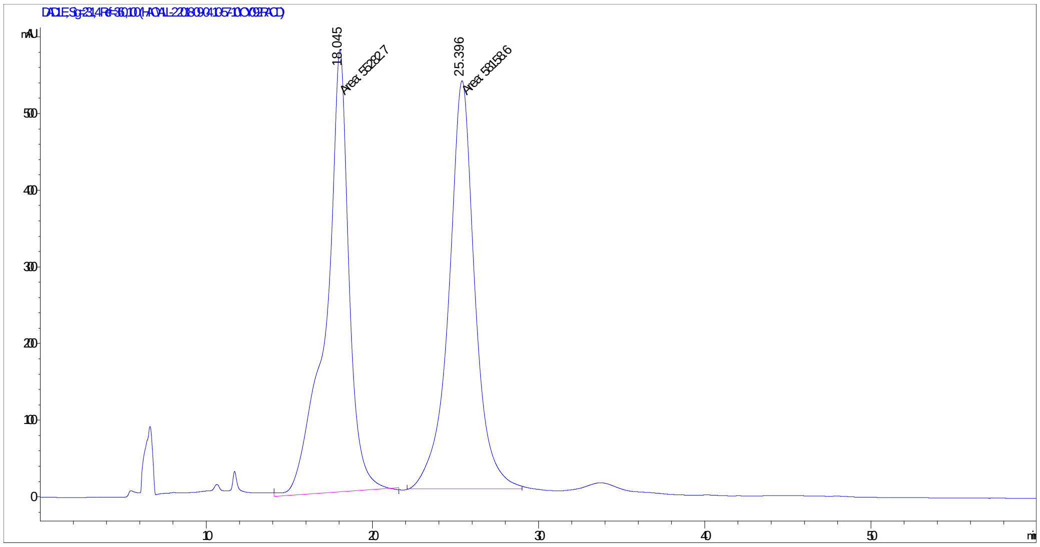

#	Time	Area	Height	Width	Area%	Symmetry
1	18.045	55282.7	576.7	1.5977	48.732	1.485
2	25.396	58158.6	532.5	1.8202	51.268	1.008

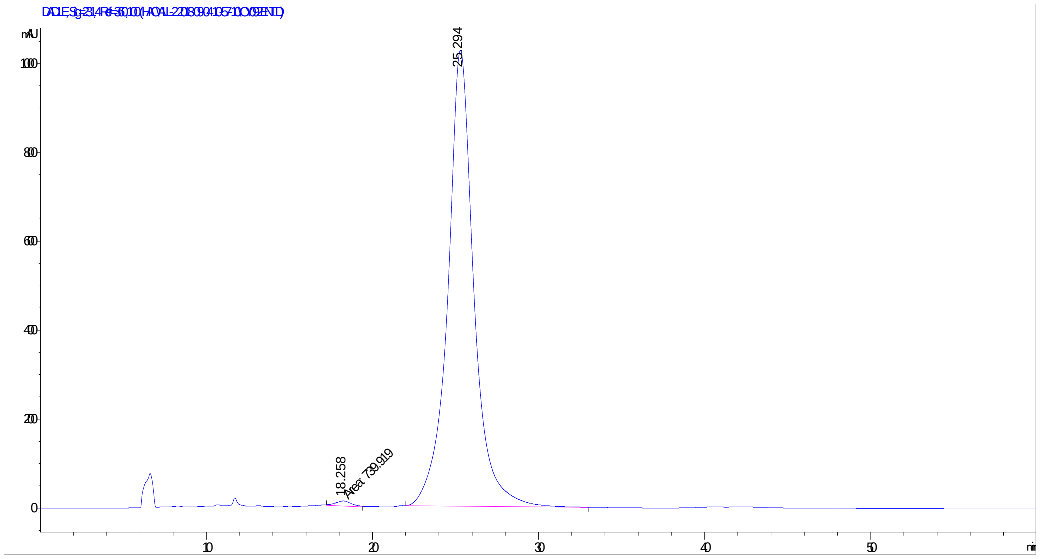

#	Time	Area	Height	Width	Area%	Symmetry
1	18.258	739.9	11.2	1.1031	0.658	0.894
2	25.294	111749.3	1025	1.5819	99.342	0.901

^{1}H NMR

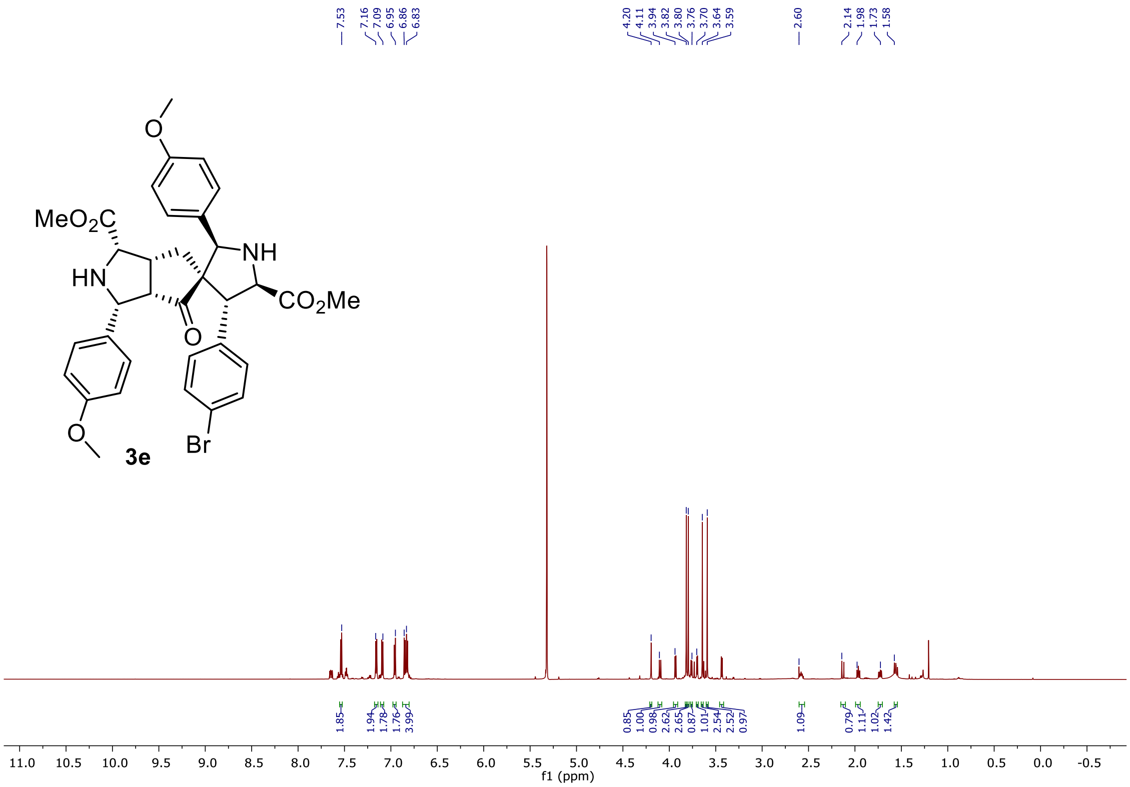

^{13}C NMR

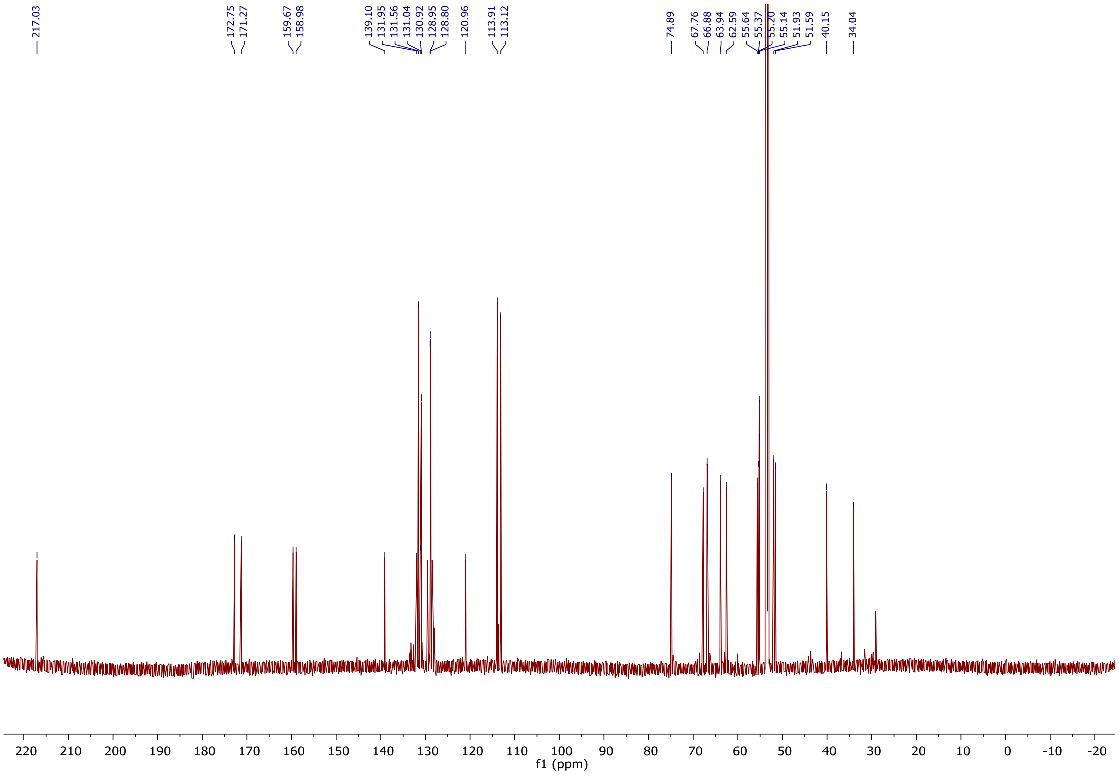

HPLC traces for **3e**: racemic top, enantiomer 1 bottom

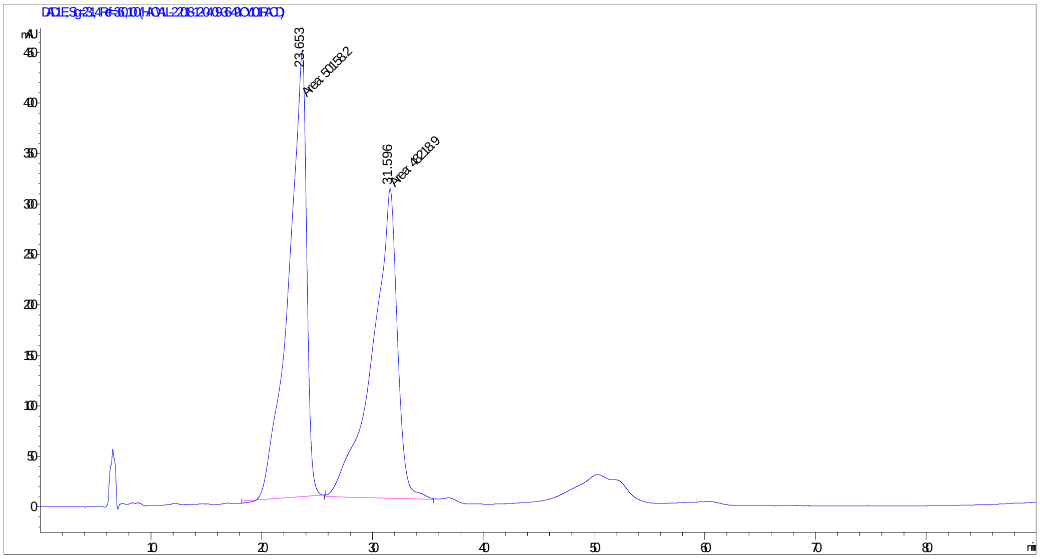

#	Time	Area	Height	Width	Area%	Symmetry
1	23.653	50158.2	441.9	1.8918	50.986	2.834
2	31.596	48218.9	306.5	2.6216	49.014	2.279

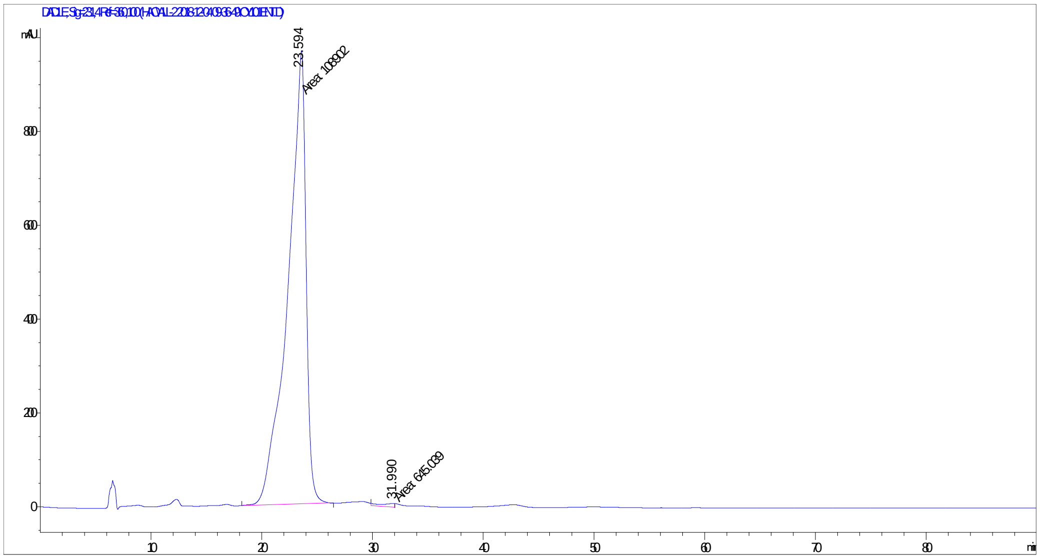

#	Time	Area	Height	Width	Area%	Symmetry
1	23.594	108902.2	965.7	1.8796	99.411	2.742
2	31.99	645	7.7	1.398	0.589	11.277

^{1}H NMR

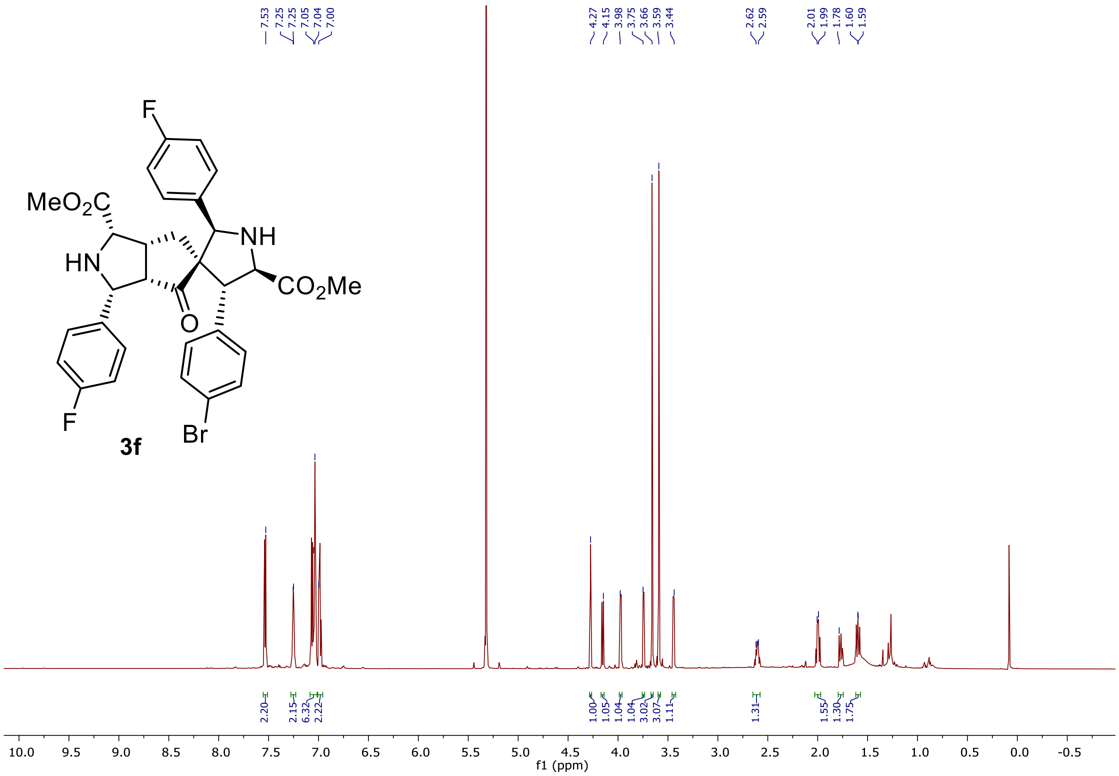

^{13}C NMR

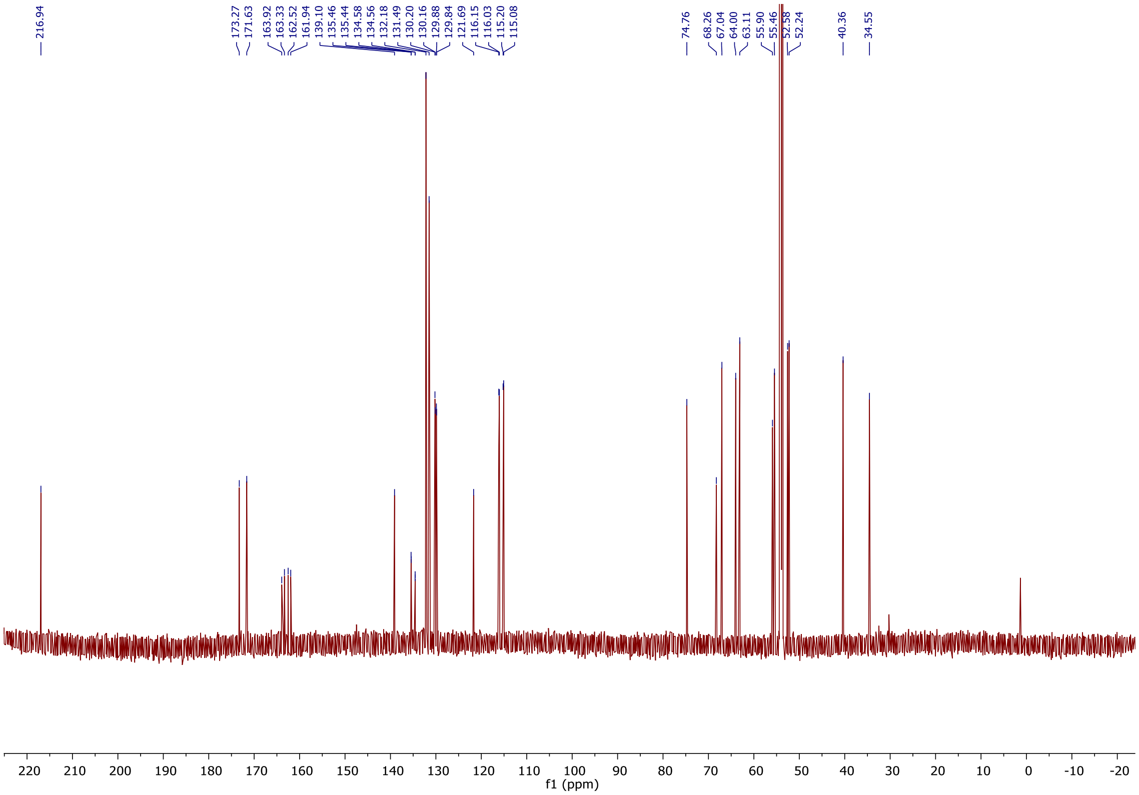

^{19}F NMR

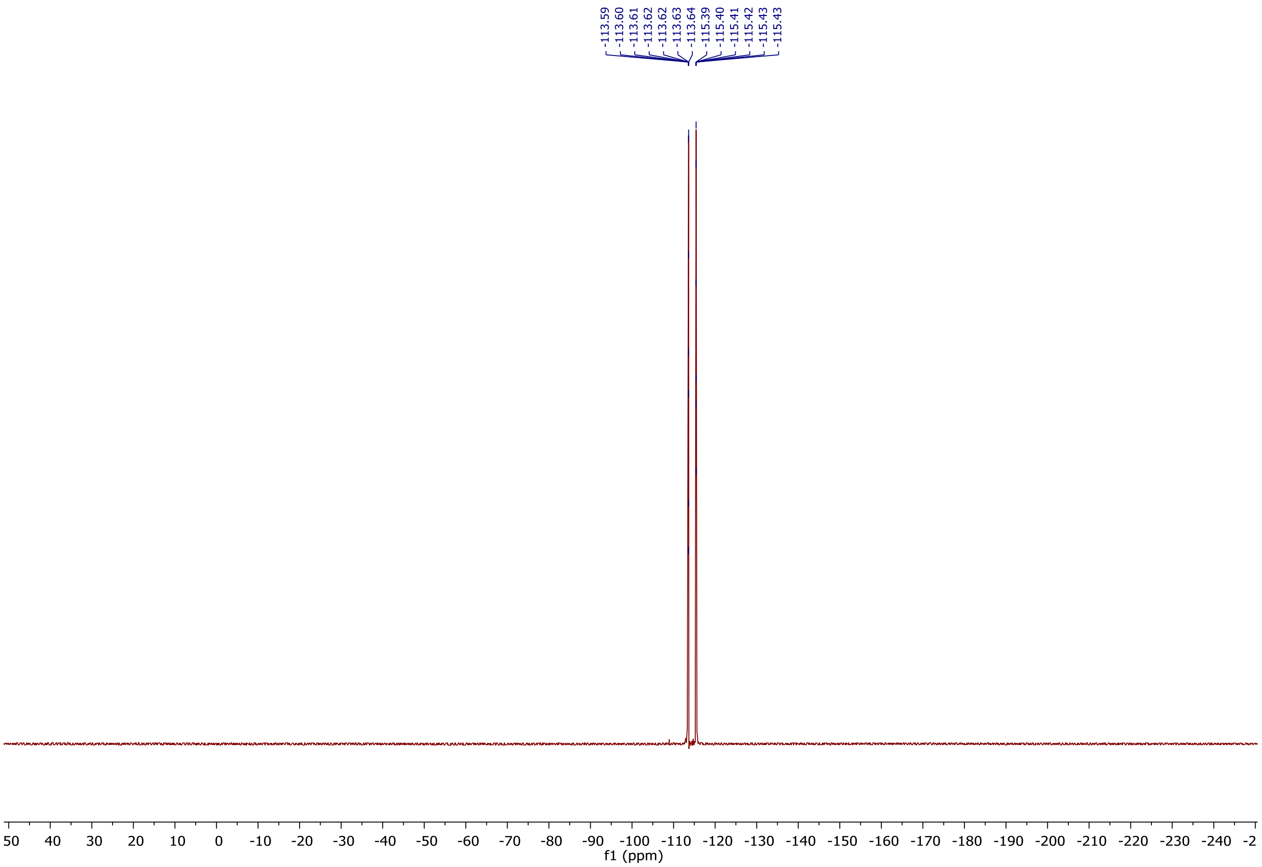
-113.59
-113.60
-113.61
-113.62
-113.62
-113.63
-113.64
-115.39
-115.40
-115.41
-115.42
-115.43
-115.43
50 40 30 20 10 0 -10 -20 -30 -40 -50 -60 -70 -80 -90 -100 -110 -120 -130 -140 -150 -160 -170 -180 -190 -200 -210 -220 -230 -240 -2
f1 (ppm)

HPLC traces for **3f**: racemic top, enantiomer 1 bottom

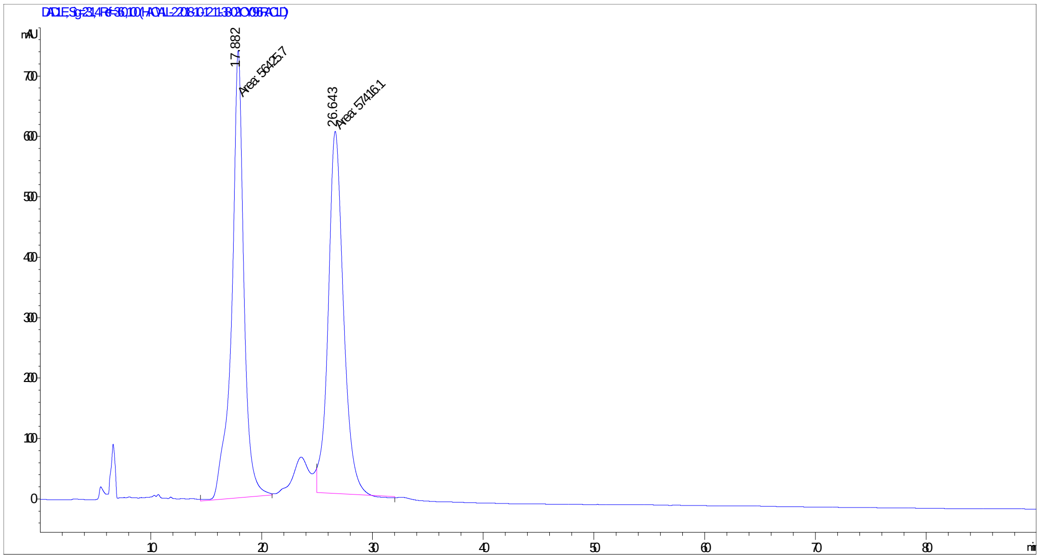

#	Time	Area	Height	Width	Area%	Symmetry
1	17.882	56425.7	740.4	1.2701	49.565	1.101
2	26.643	57416.1	599.8	1.5955	50.435	0.82

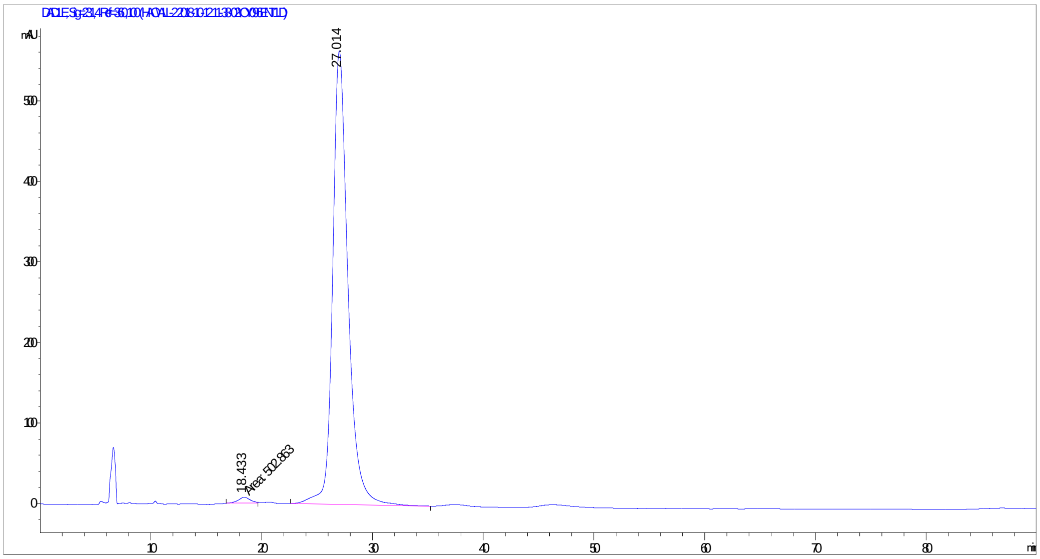

#	Time	Area	Height	Width	Area%	Symmetry
1	18.433	502.9	7	1.1939	0.920	0.931
2	27.014	54165.8	562.8	1.4348	99.080	0.735

^{1}H NMR

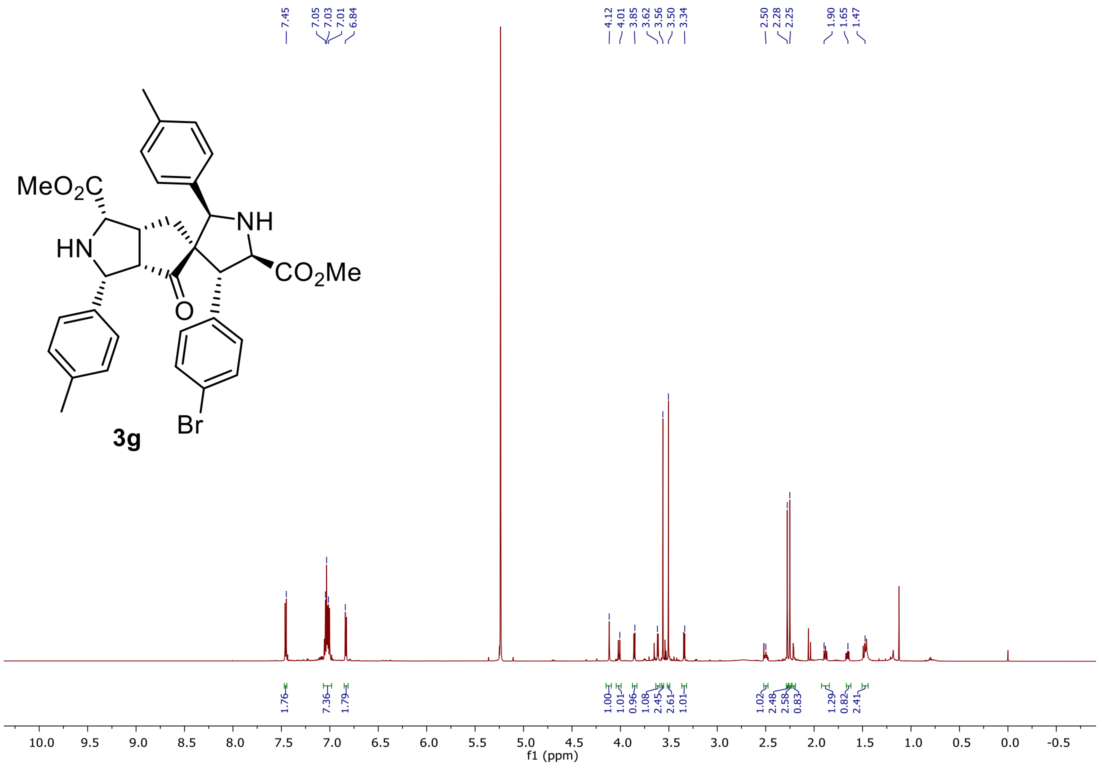

^{13}C NMR

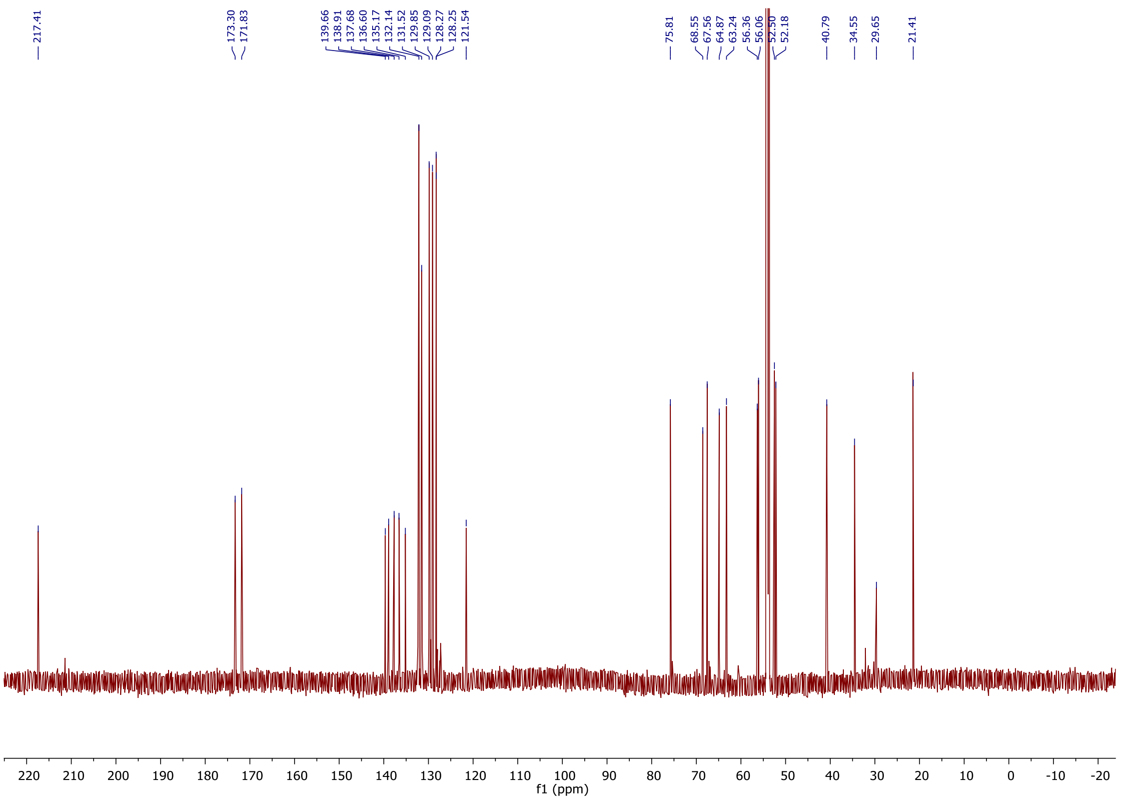

HPLC traces for **3g**: racemic top, enantiomer 1 bottom

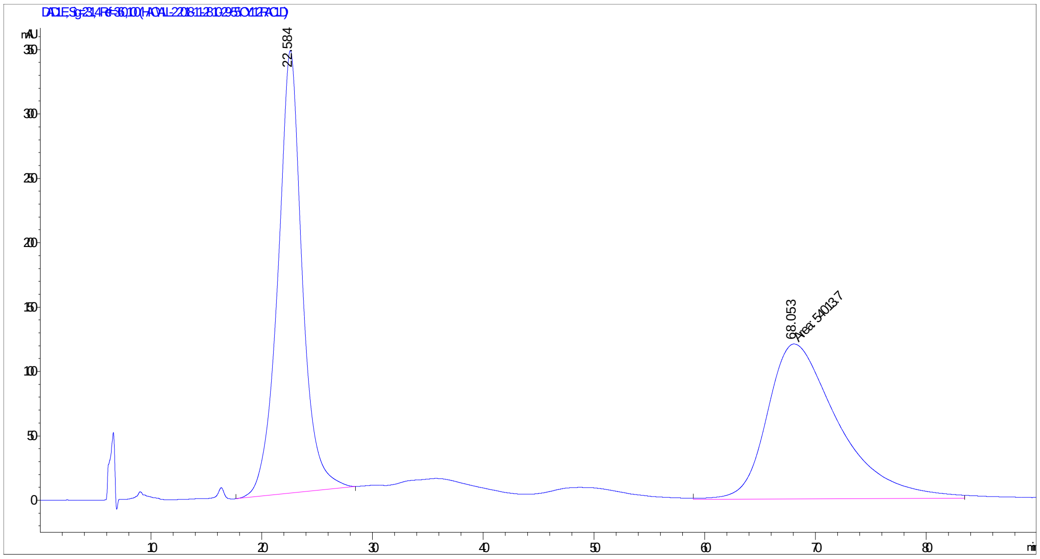

#	Time	Area	Height	Width	Area%	Symmetry
1	22.584	52551.8	343.8	2.2035	49.314	0.996
2	68.053	54013.7	120.4	7.4752	50.686	0.582

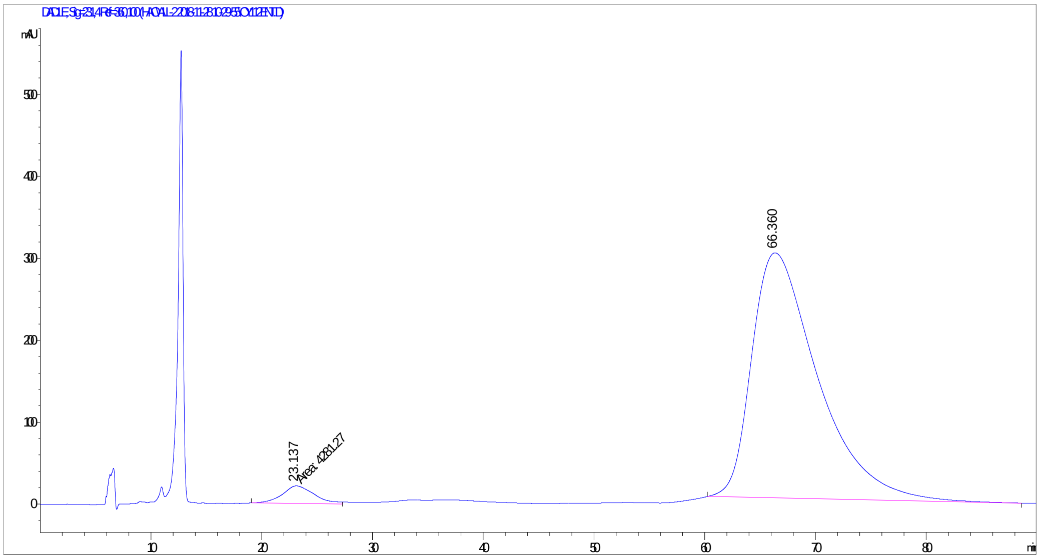

#	Time	Area	Height	Width	Area%	Symmetry
1	23.137	4281.3	21.4	3.3363	3.336	0.742
2	66.36	124063.2	299.1	5.2751	96.664	0.504

^{1}H NMR

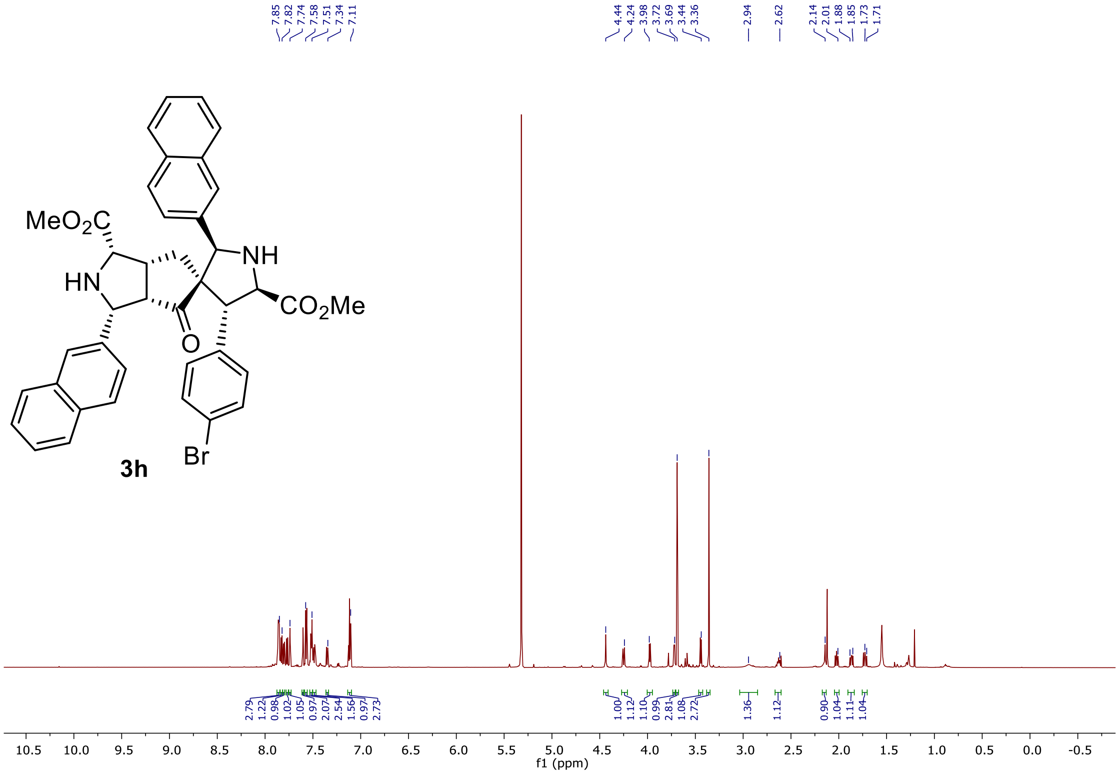

3h

^{13}C NMR

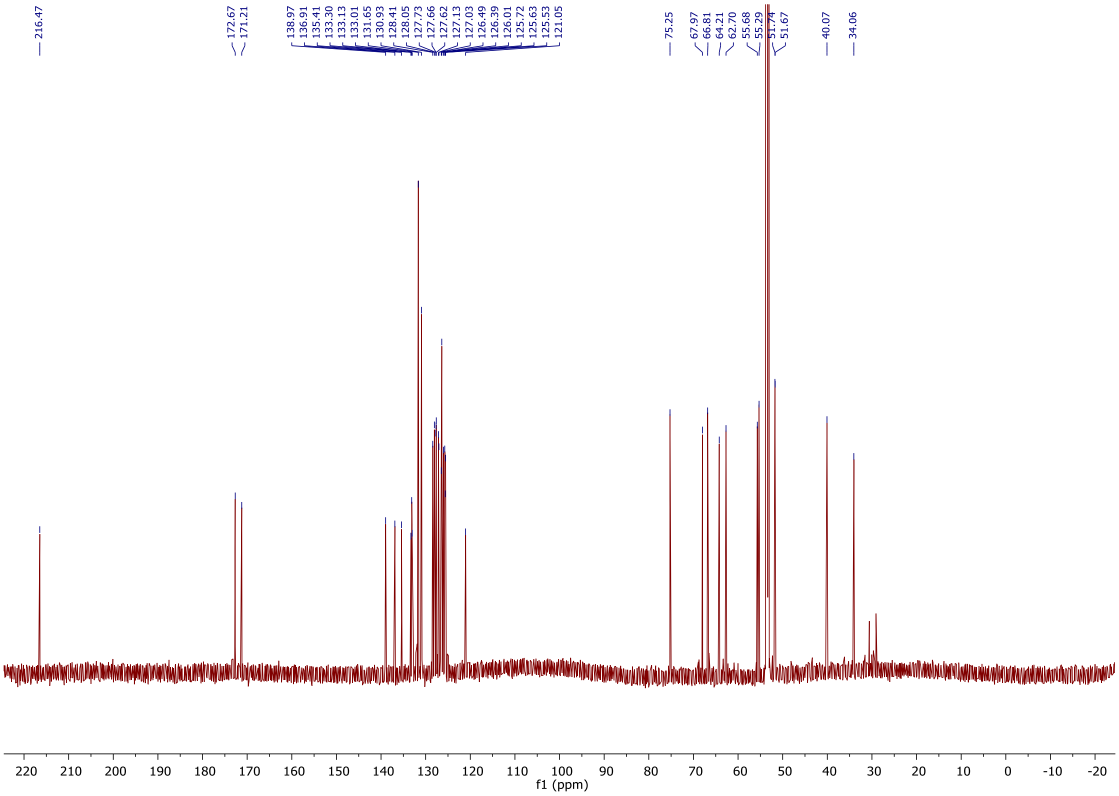

HPLC traces for **3h**: racemic top, enantiomer 1 bottom

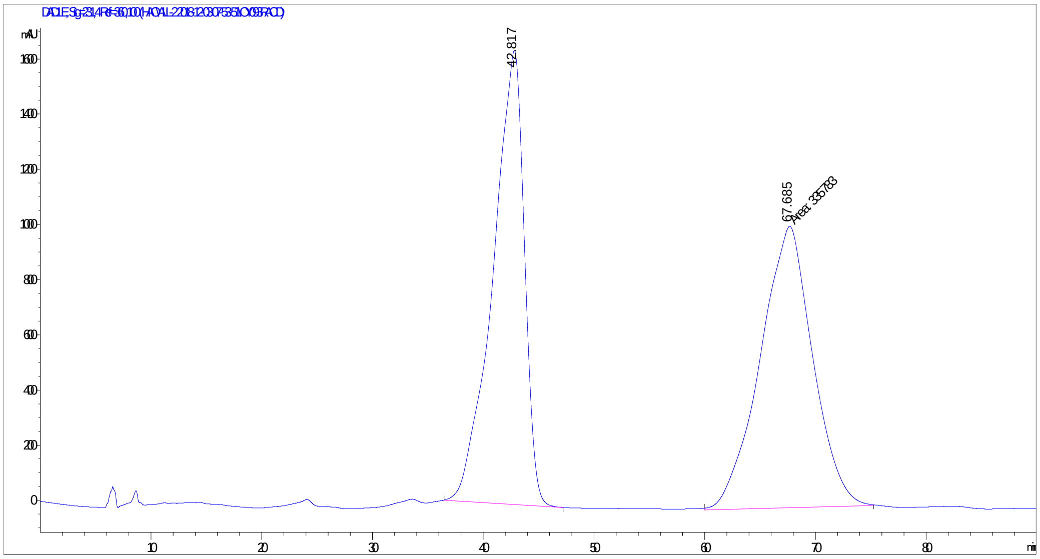

#	Time	Area	Height	Width	Area%	Symmetry
1	42.817	324564.6	1645	2.6639	49.151	2.067
2	67.685	335783.2	1019.9	5.4871	50.849	1.232

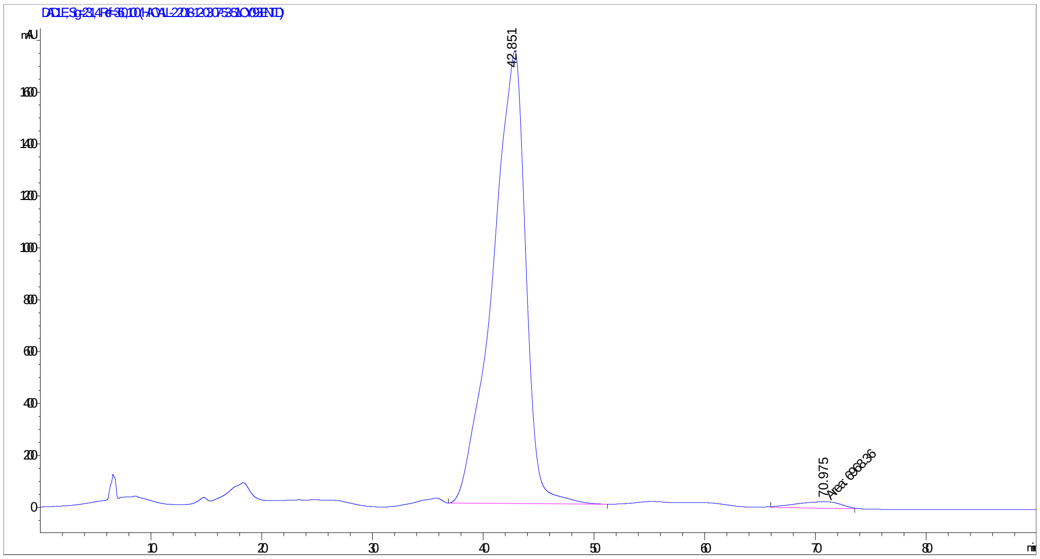

#	Time	Area	Height	Width	Area%	Symmetry
1	42.851	361646.3	1744.2	2.741	98.110	1.892
2	70.975	6968.4	25.2	4.6147	1.890	1.71

^{1}H NMR

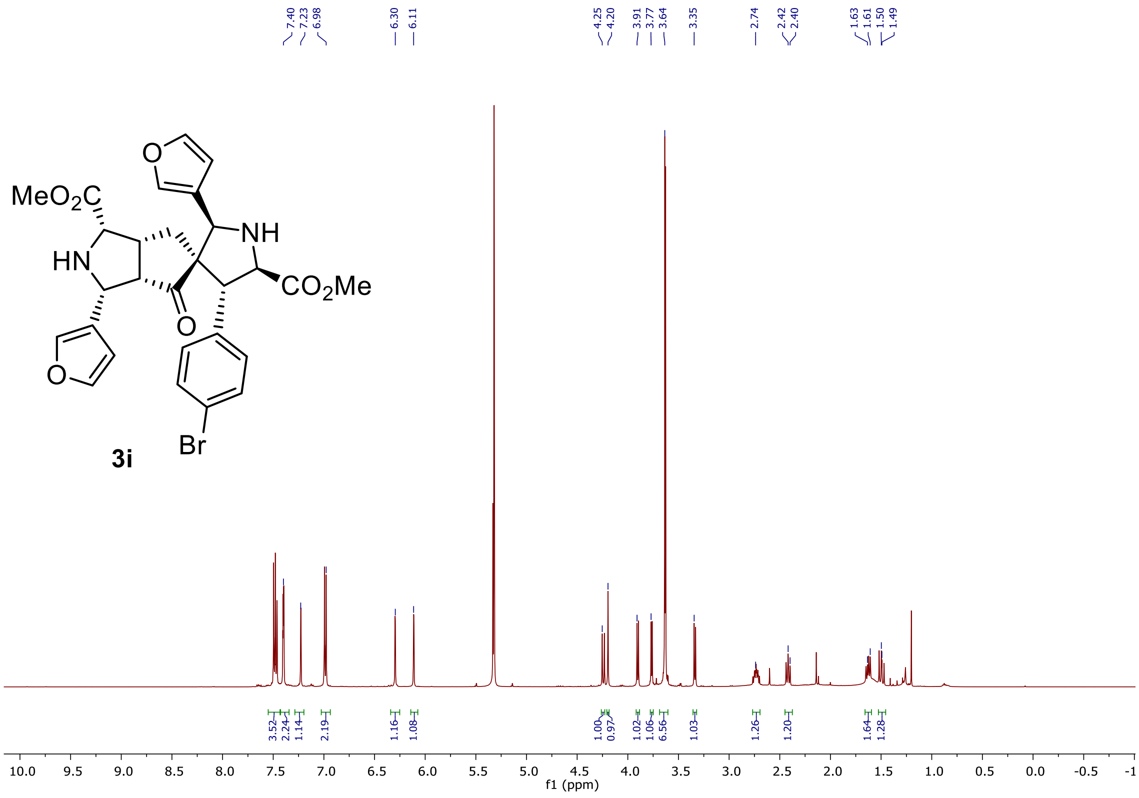

^{13}C NMR

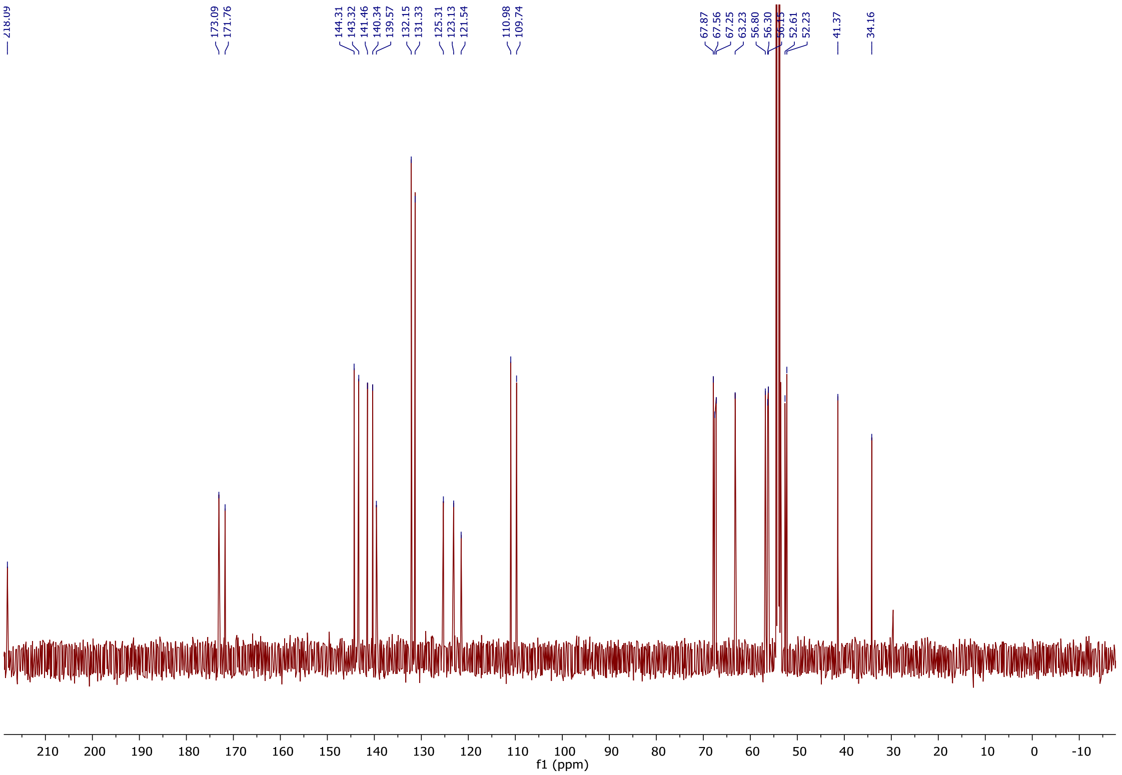

HPLC traces for **3i**: racemic top, enantiomer 1 bottom

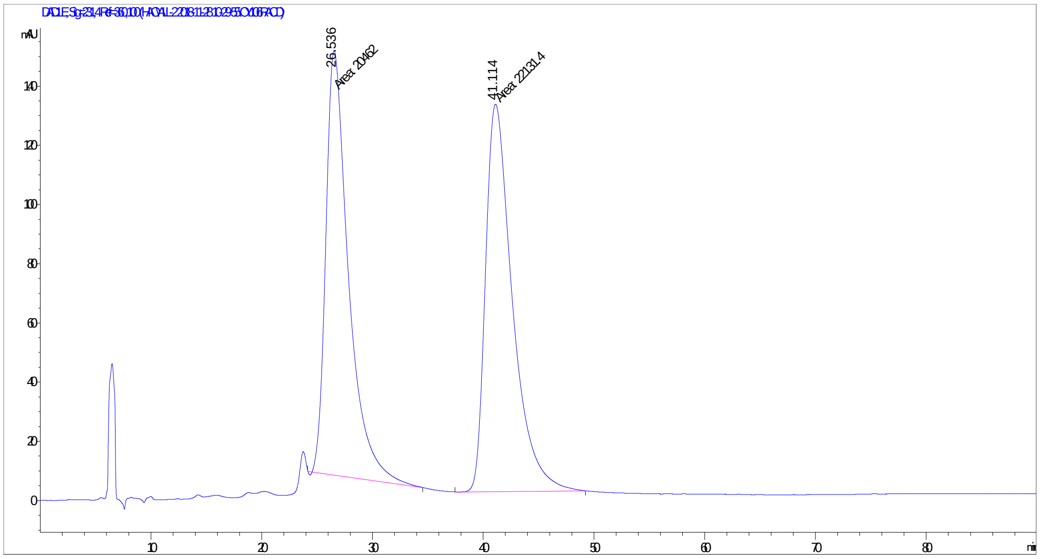

#	Time	Area	Height	Width	Area%	Symmetry
1	26.536	20462	143.5	2.3768	48.040	0.549
2	41.114	22131.4	130.9	2.817	51.960	0.568

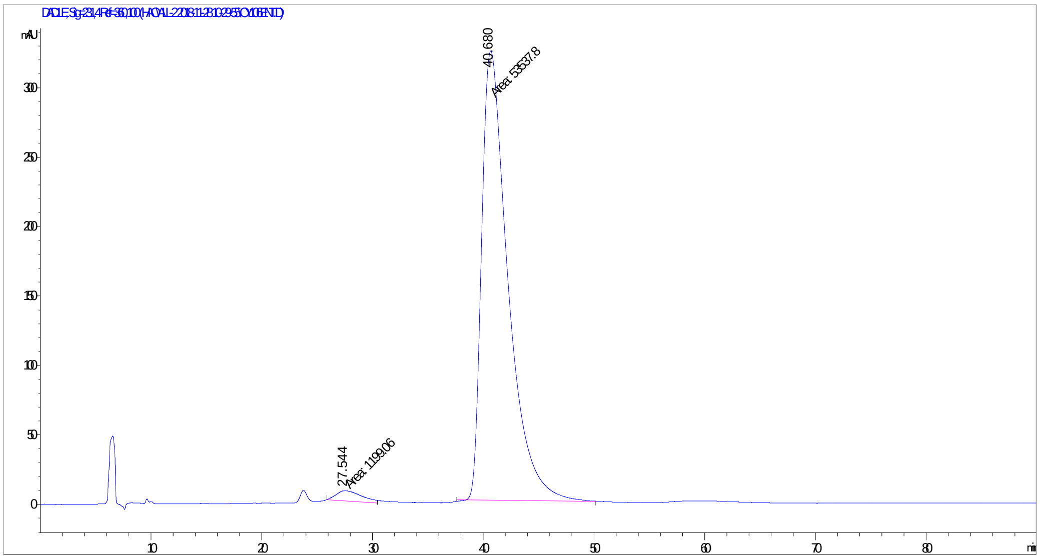

#	Time	Area	Height	Width	Area%	Symmetry
1	27.544	1199.1	7.4	2.7173	2.191	0.476
2	40.68	53537.8	323.6	2.7574	97.809	0.5

^{1}H NMR

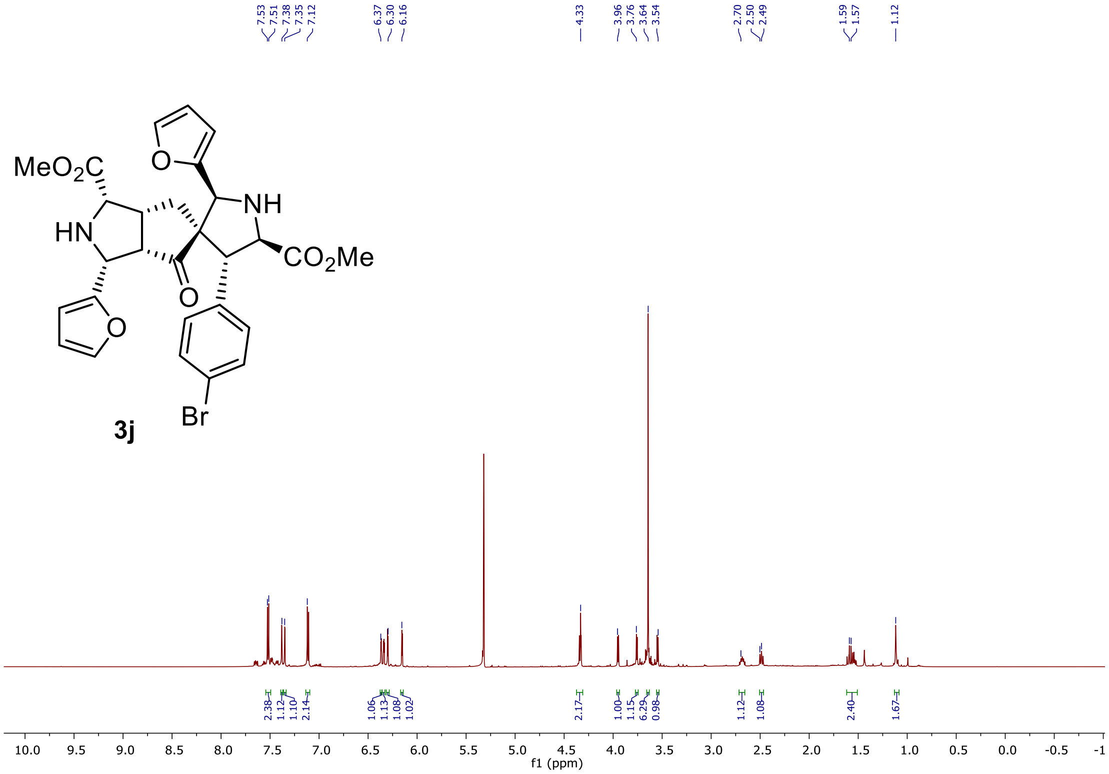

^{13}C NMR

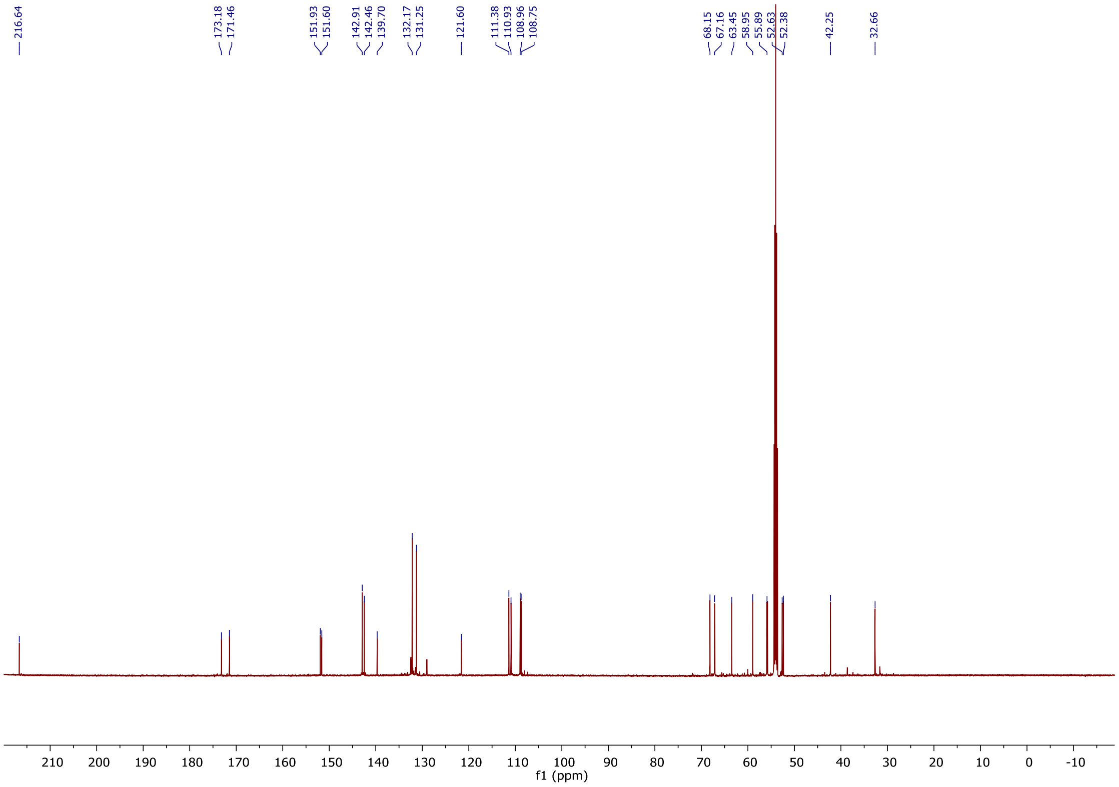

HPLC traces for **3j**: racemic top, enantiomer 1 bottom

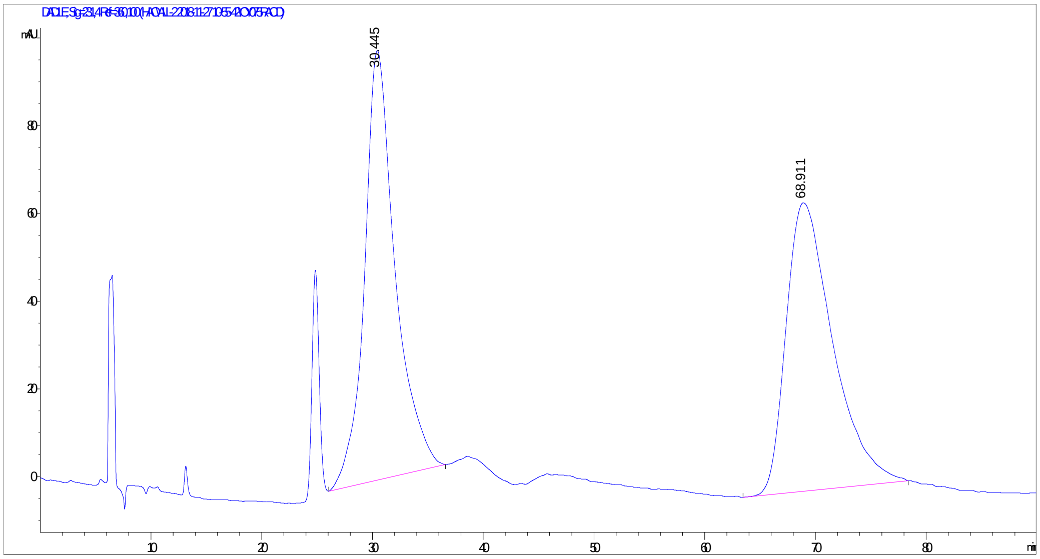

#	Time	Area	Height	Width	Area%	Symmetry
1	30.445	18867.8	97.9	2.7285	49.497	0.693
2	68.911	19250.9	65.8	3.7418	50.503	0.546

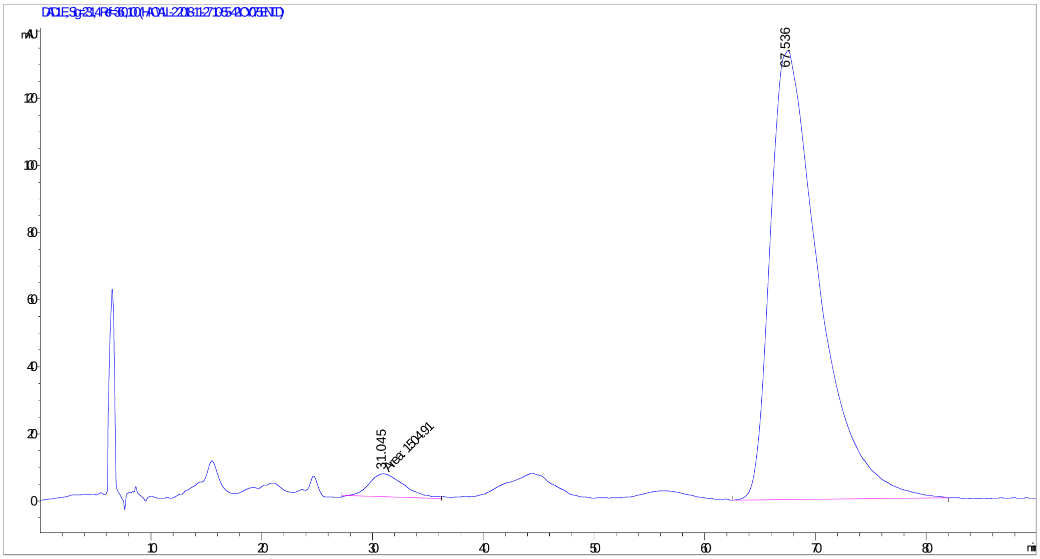

#	Time	Area	Height	Width	Area%	Symmetry
1	31.042	1084.8	6.8	2.6645	2.154	0.799
2	67.544	49278.5	161.5	3.6813	97.846	0.557

^{1}H NMR

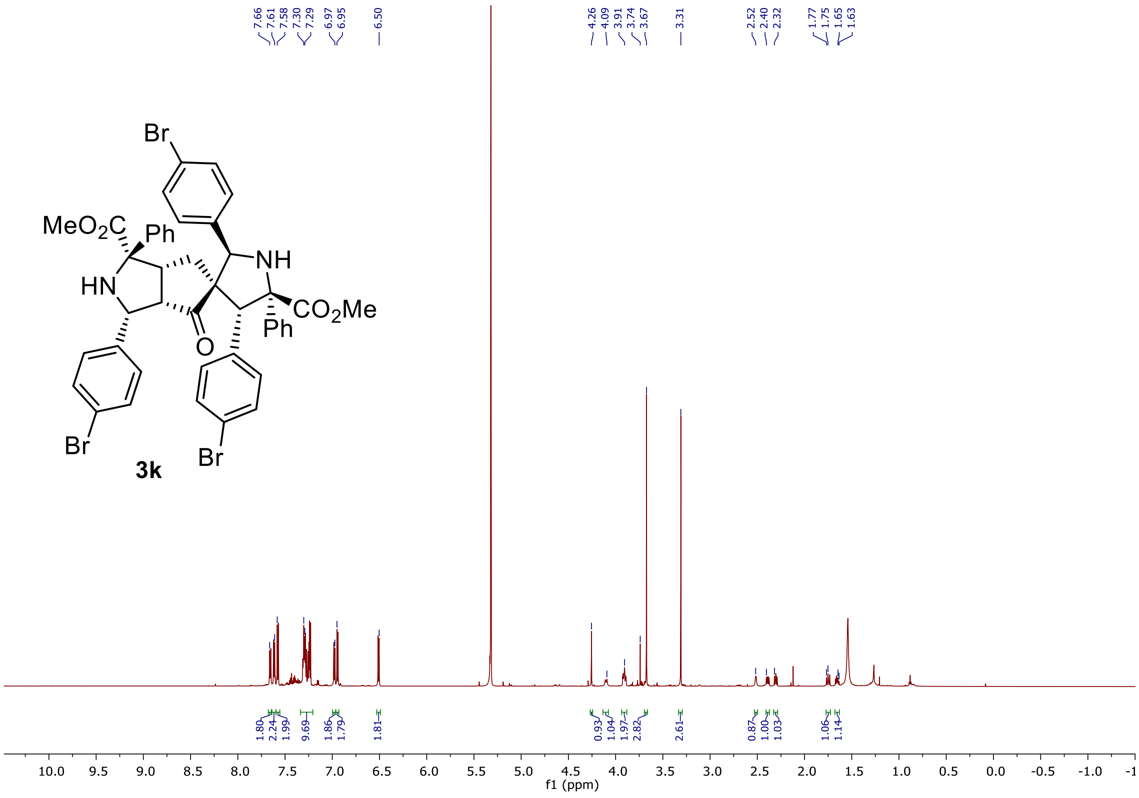

^{13}C NMR

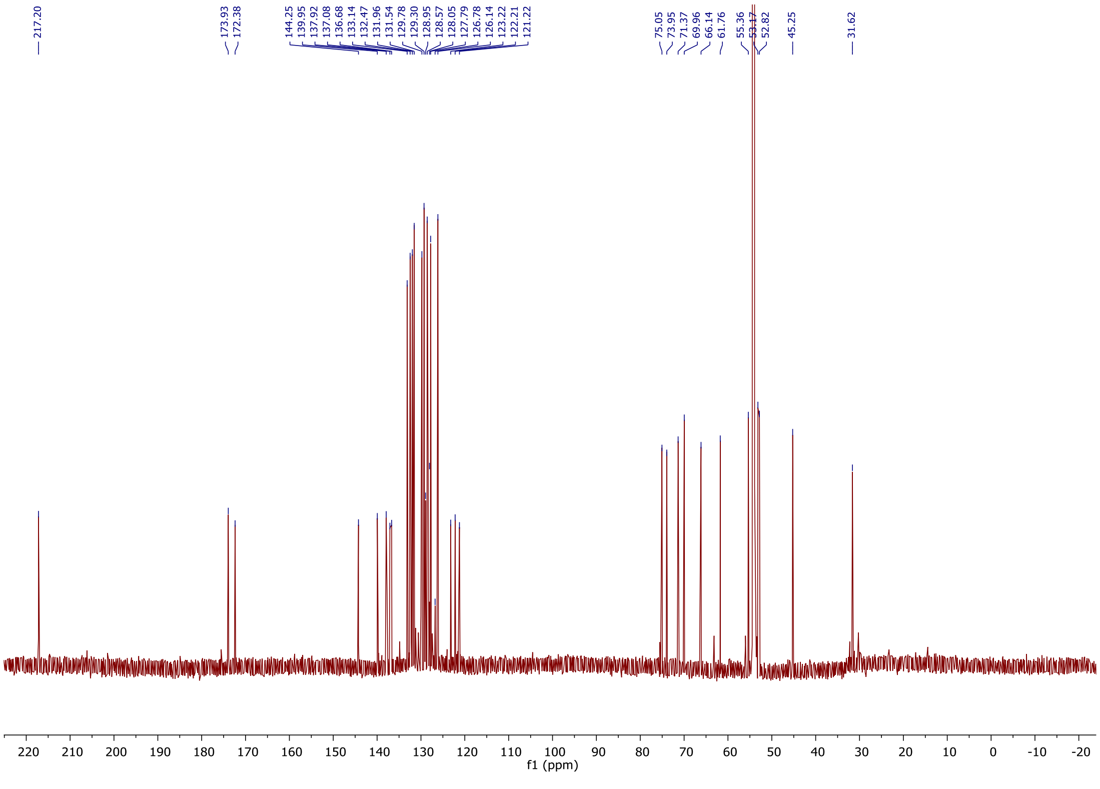

HPLC traces for **3k**: racemic top, enantiomer 1 bottom

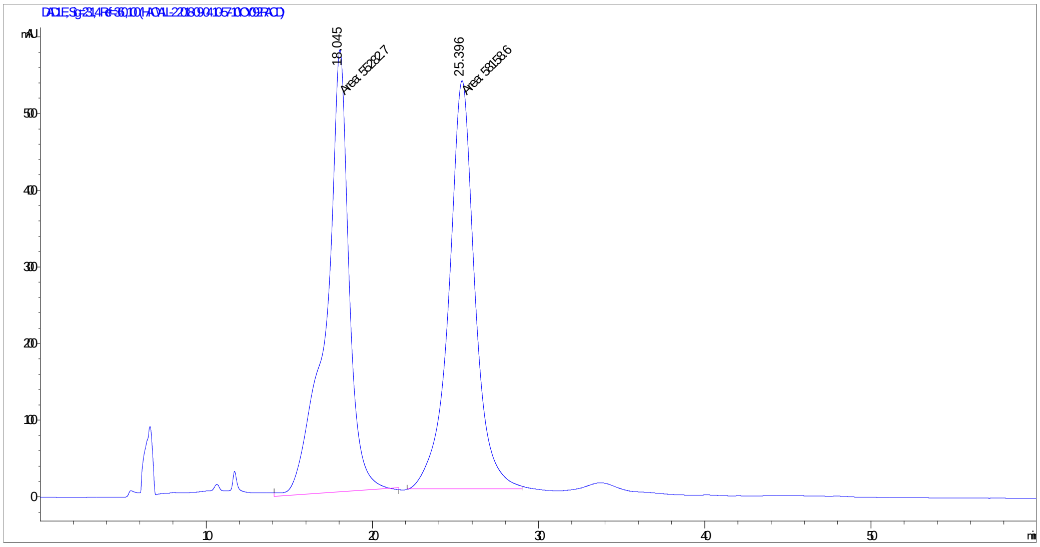

#	Time	Area	Height	Width	Area%	Symmetry
1	18.045	55282.7	576.7	1.5977	48.732	1.485
2	25.396	58158.6	532.5	1.8202	51.268	1.008

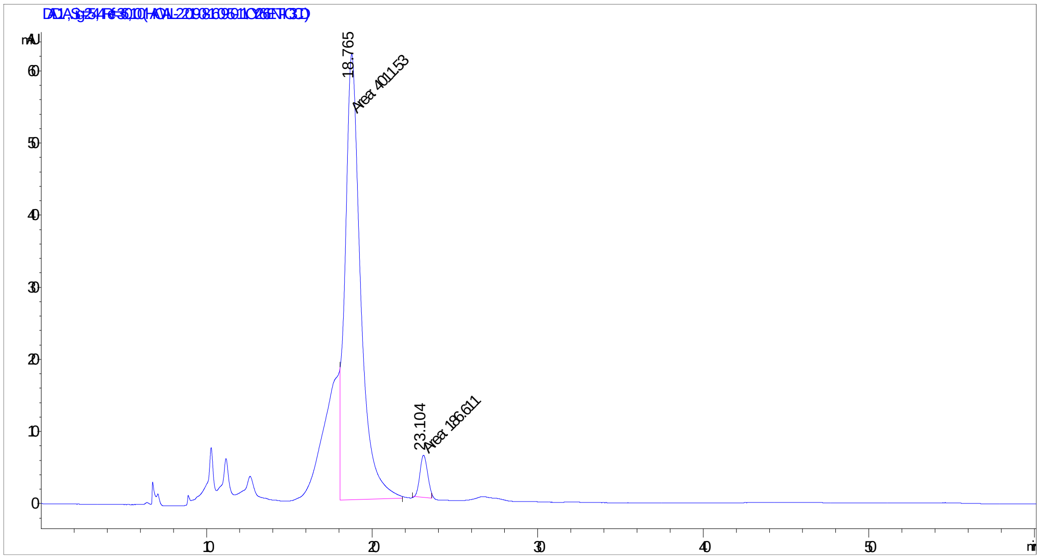

#	Time	Area	Height	Width	Area%	Symmetry
1	18.767	31013.5	370.5	1.1685	95.660	1.242
2	26.611	1407.2	7.4	3.1552	4.340	1.266

^{1}H NMR

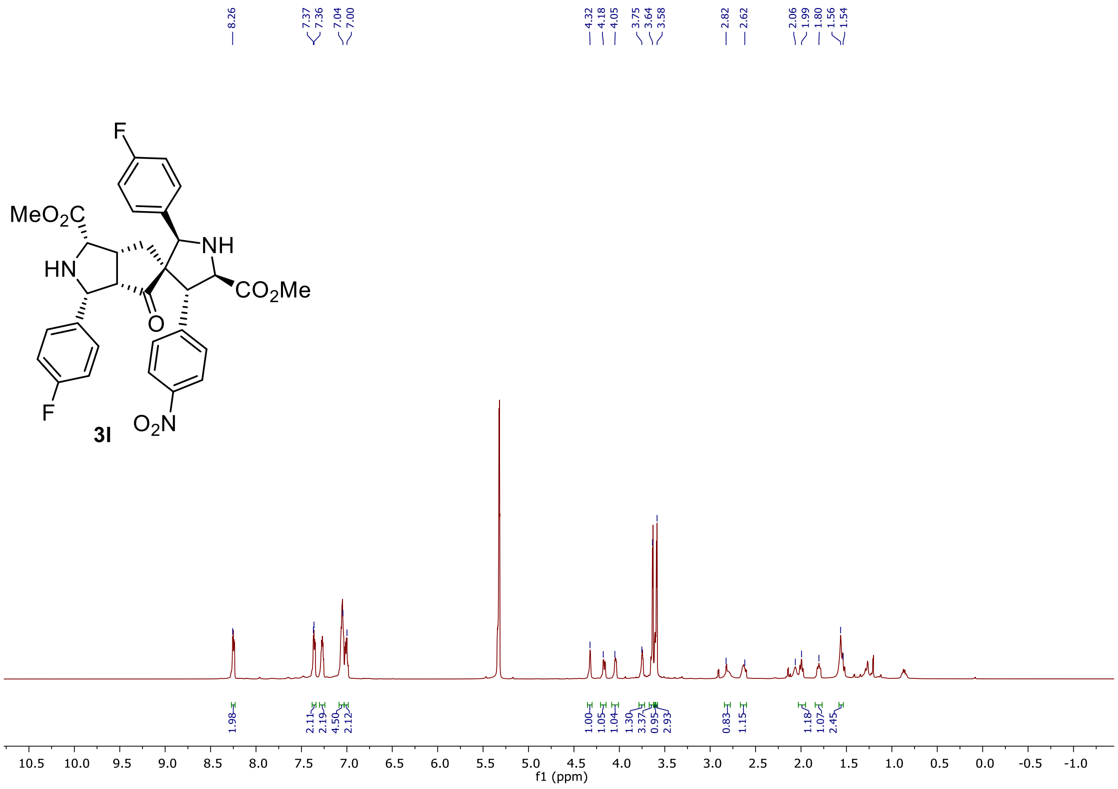

^{13}C NMR

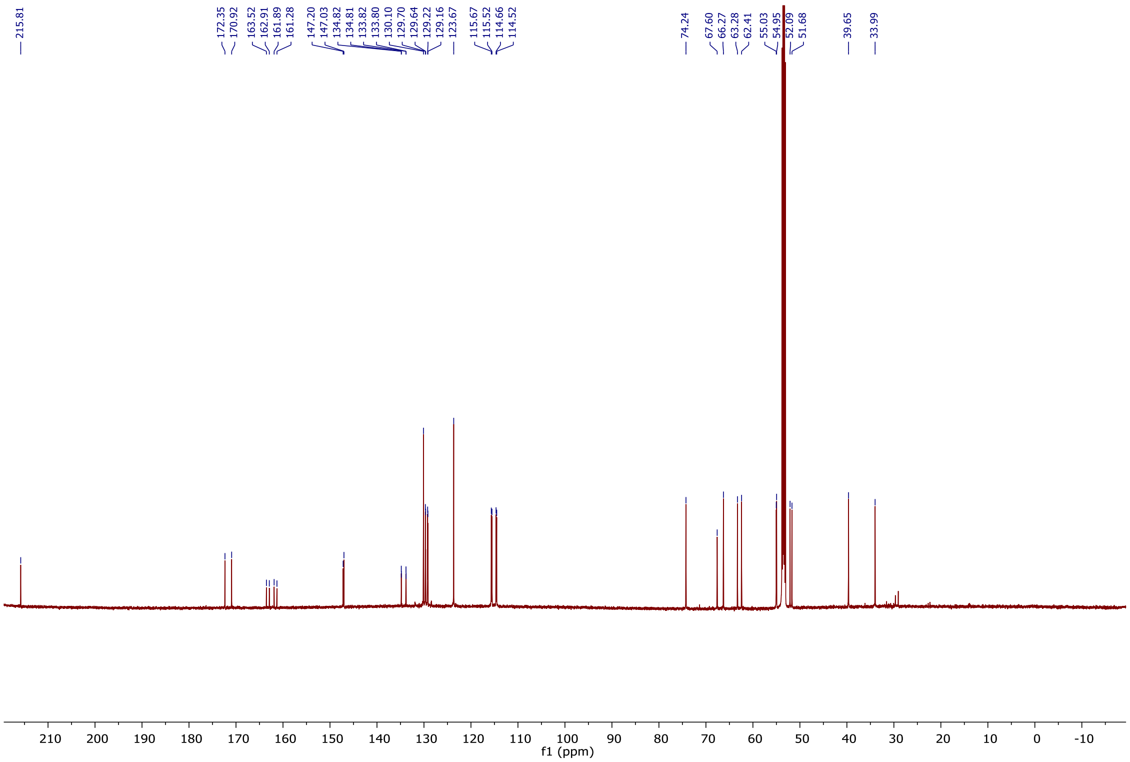

^{19}F NMR

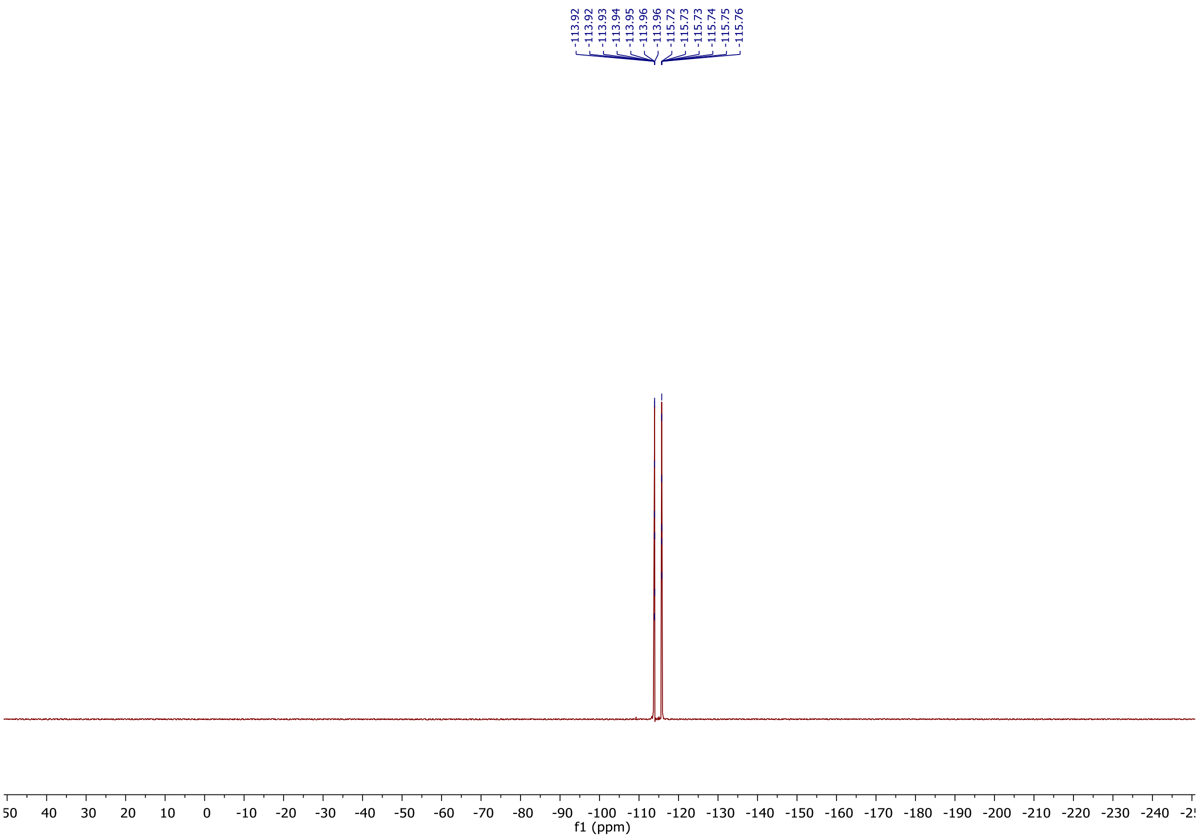

-113.92
-113.92
-113.93
-113.94
-113.95
-113.96
-113.96
-115.72
-115.73
-115.73
-115.74
-115.75
-115.76
f1 (ppm)

HPLC traces for **3l**: racemic top, enantiomer 1 bottom

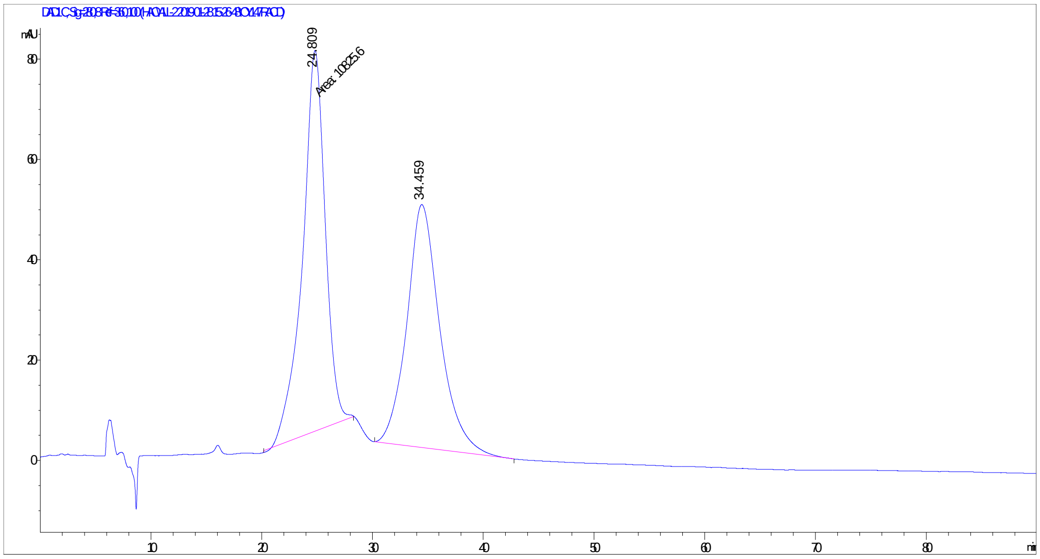

#	Time	Area	Height	Width	Area%	Symmetry
1	24.809	10825.6	75.9	2.3765	51.939	1.186
2	34.459	10017.3	48.5	2.788	48.061	0.802

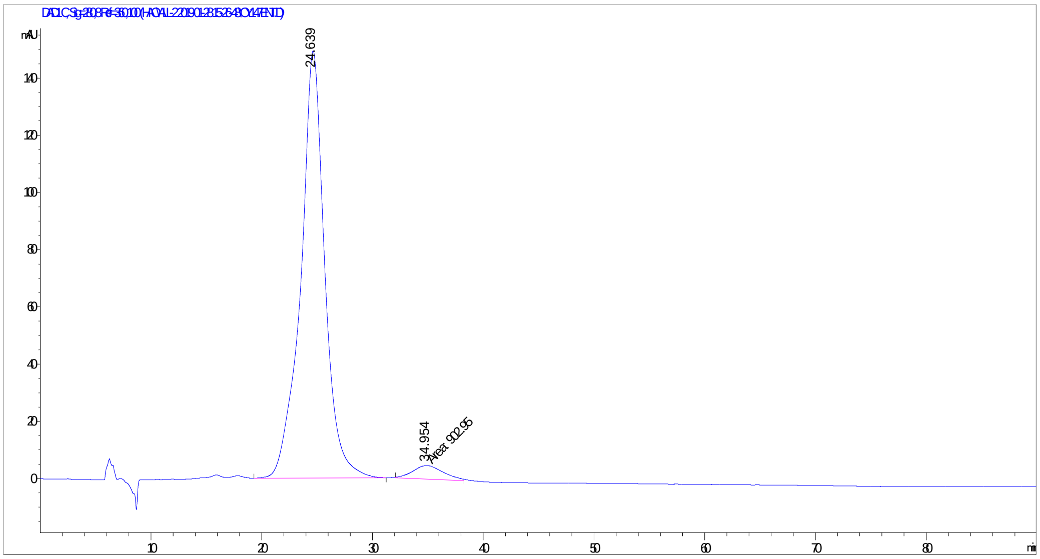

#	Time	Area	Height	Width	Area%	Symmetry
1	24.639	22495.4	149.3	2.1161	96.141	1.048
2	34.954	903	4.7	3.1844	3.859	0.813

^{1}H NMR

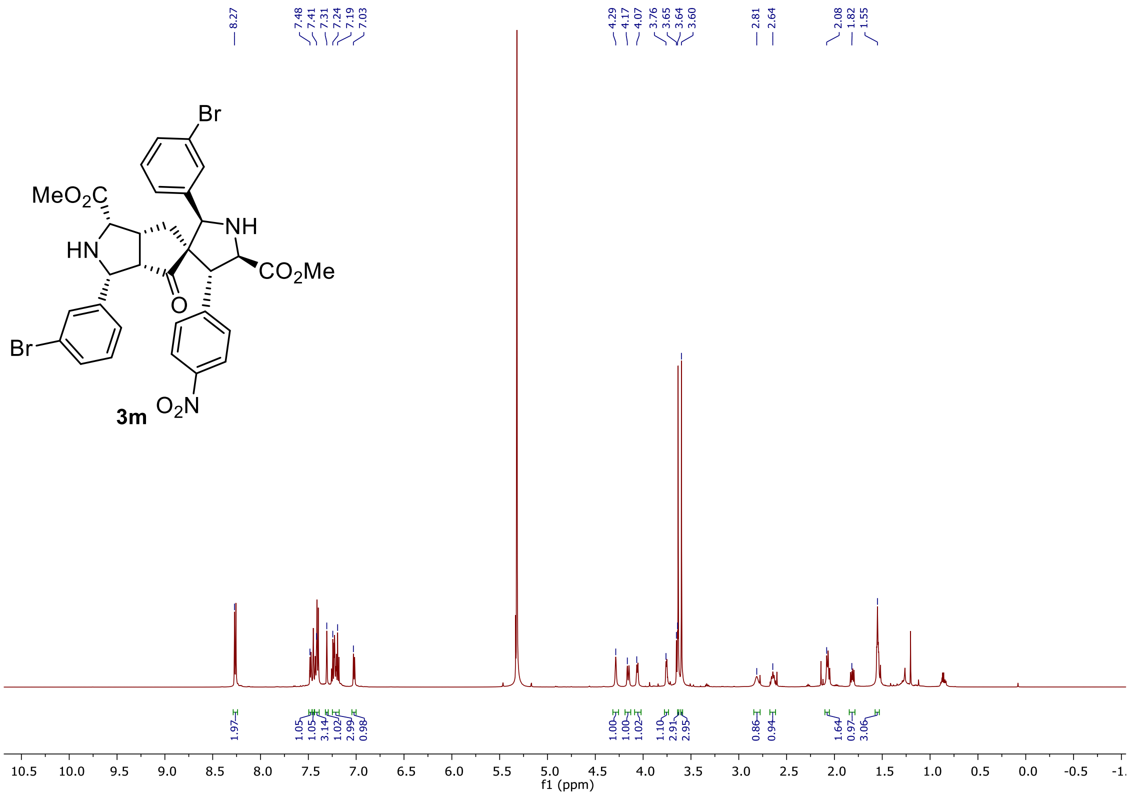

^{13}C NMR

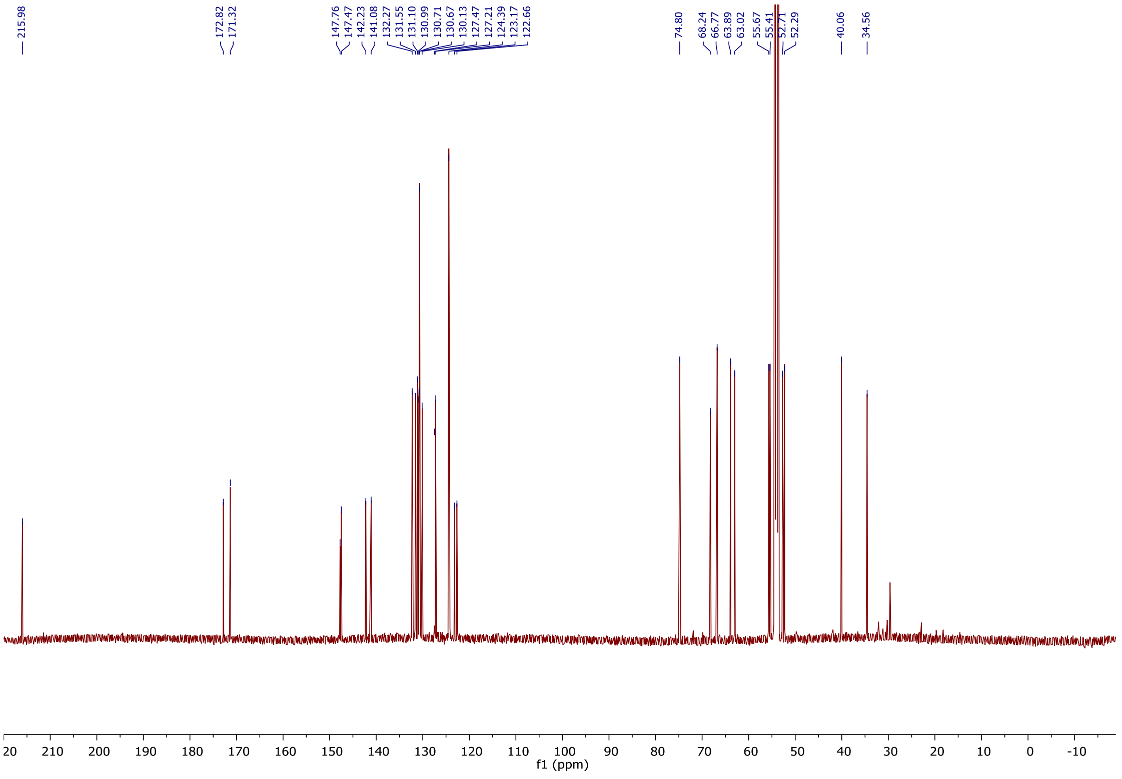

HPLC traces for **3m**: racemic top, enantiomer 1 bottom

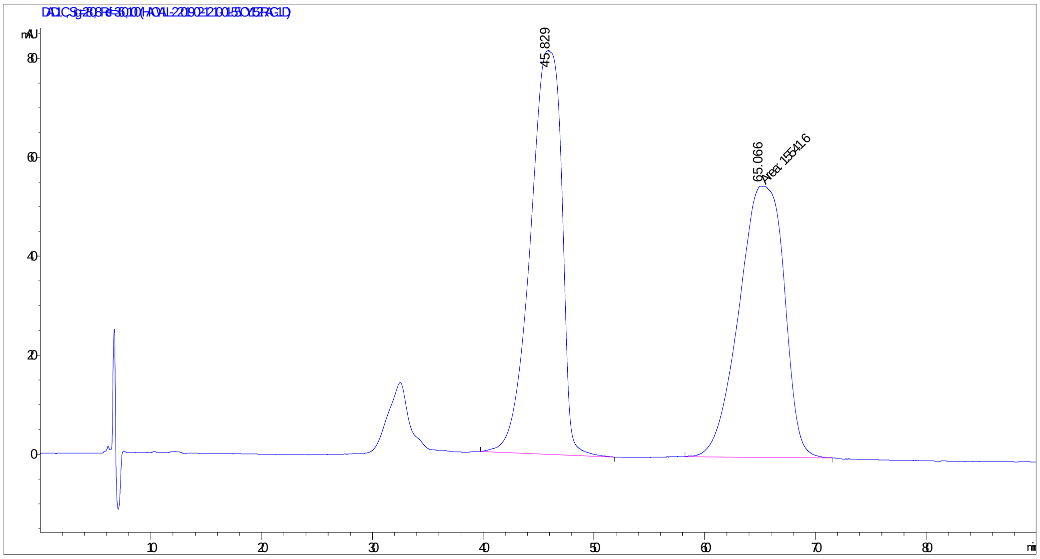

#	Time	Area	Height	Width	Area%	Symmetry
1	45.829	16067.6	81.5	2.4231	50.832	1.209
2	65.066	15541.6	54.8	4.728	49.168	0.934

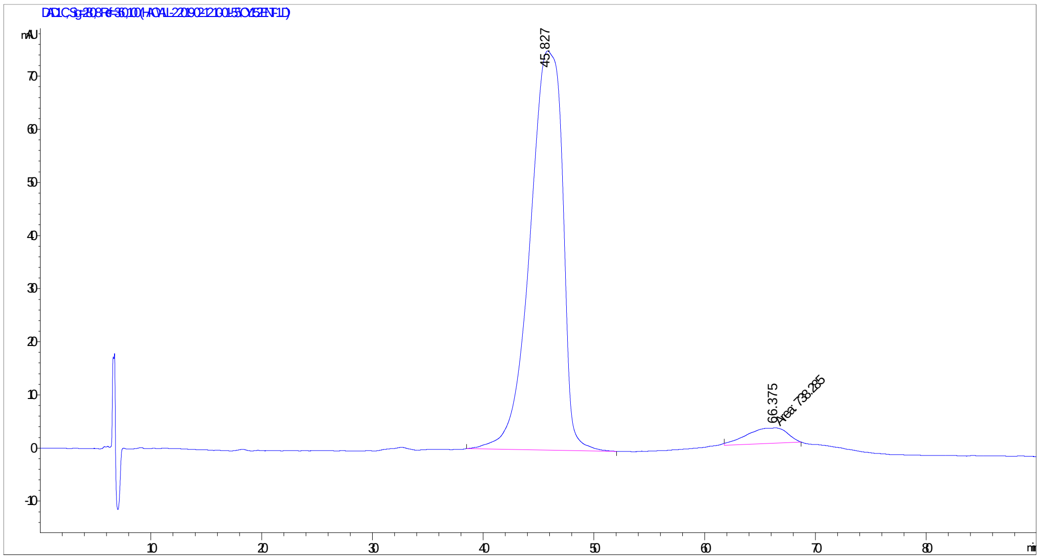

#	Time	Area	Height	Width	Area%	Symmetry
1	45.827	15480.3	75.1	2.4613	95.448	1.176
2	66.375	738.3	2.9	4.2593	4.552	2.292

^{1}H NMR

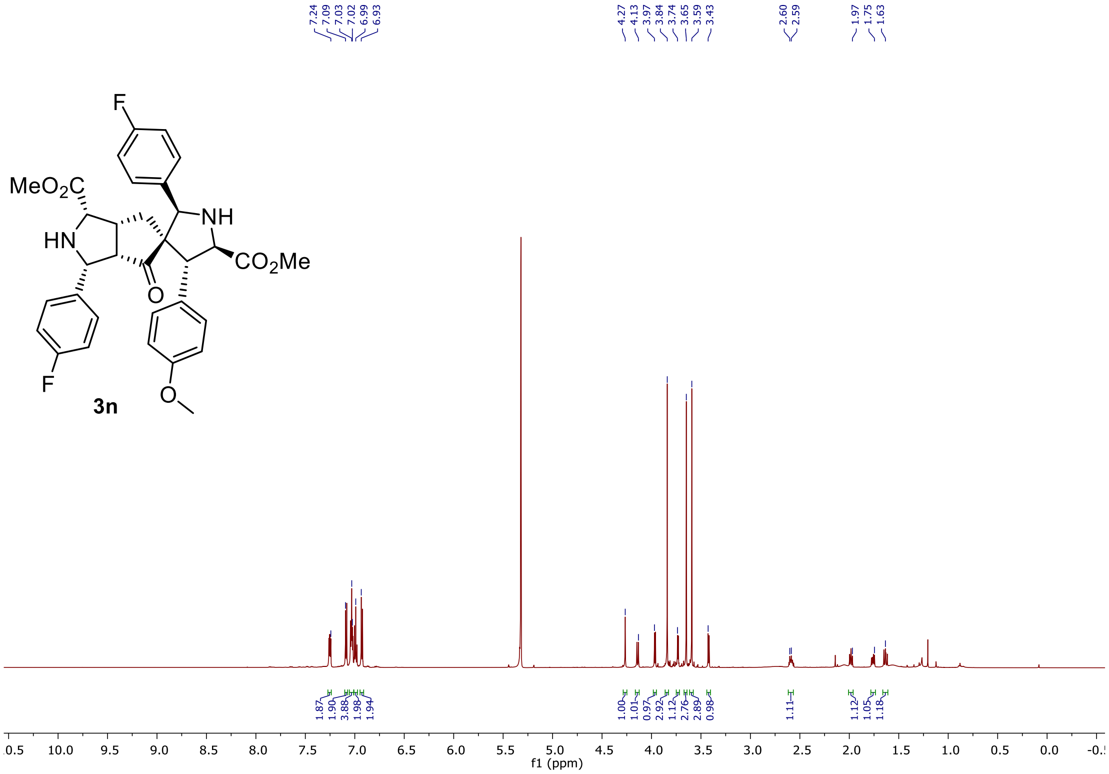

3n

^{13}C NMR

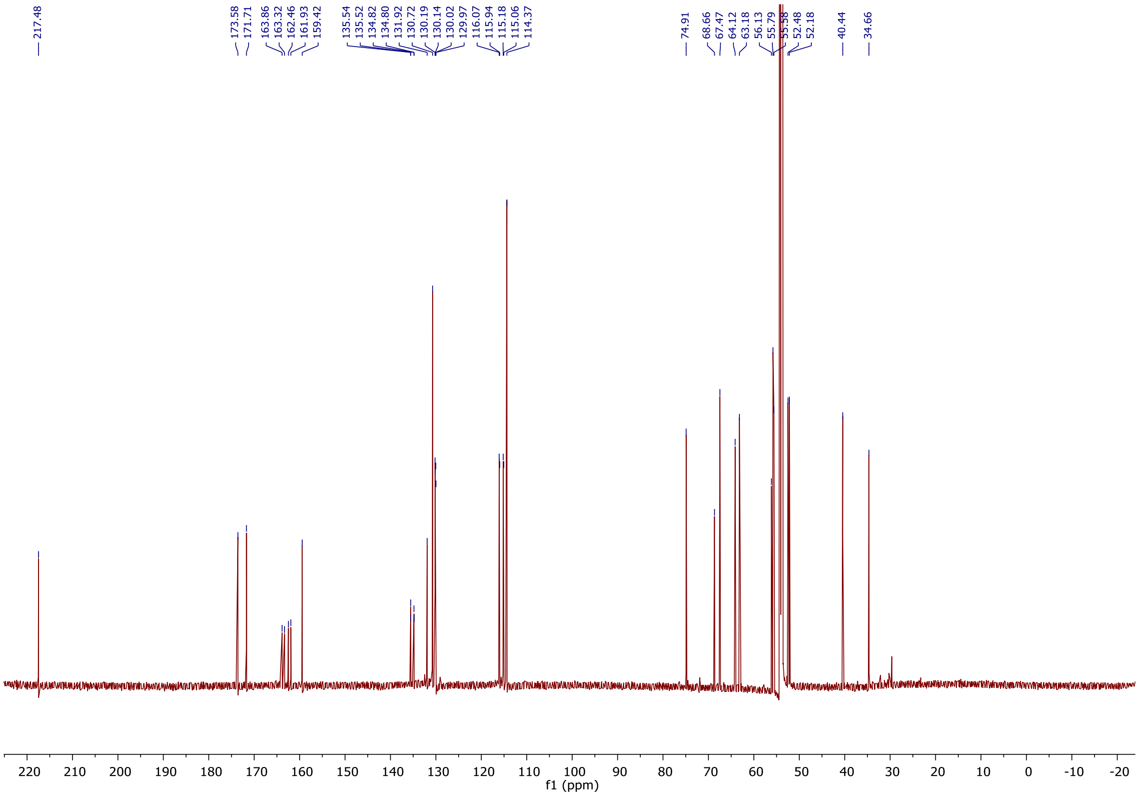

^{19}F NMR

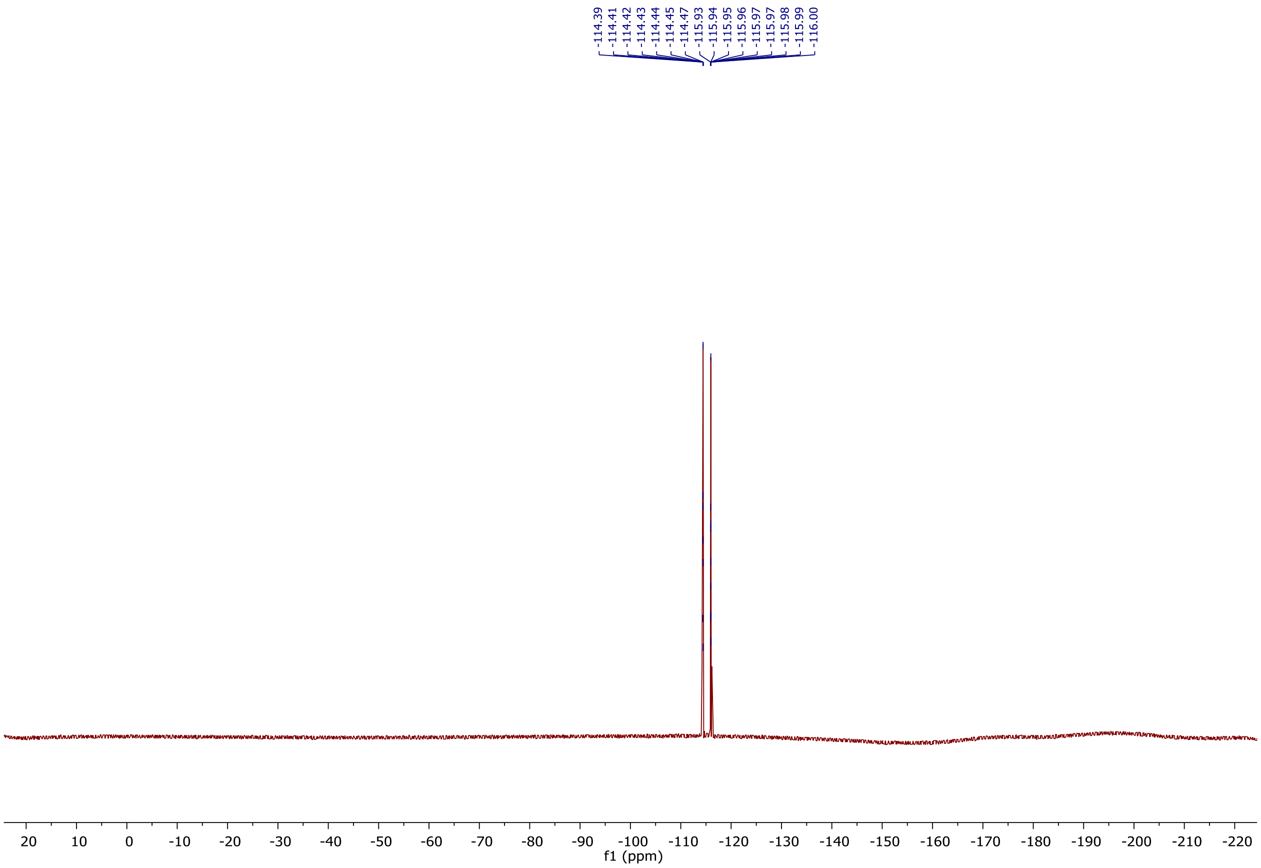

-114.39
-114.41
-114.42
-114.43
-114.44
-114.45
-114.47
-115.93
-115.94
-115.95
-115.96
-115.97
-115.98
-115.99
-116.00
20 10 0 -10 -20 -30 -40 -50 -60 -70 -80 -90 -100 -110 -120 -130 -140 -150 -160 -170 -180 -190 -200 -210 -220
f1 (ppm)

HPLC traces for **3n**: racemic top, enantiomer 1 bottom

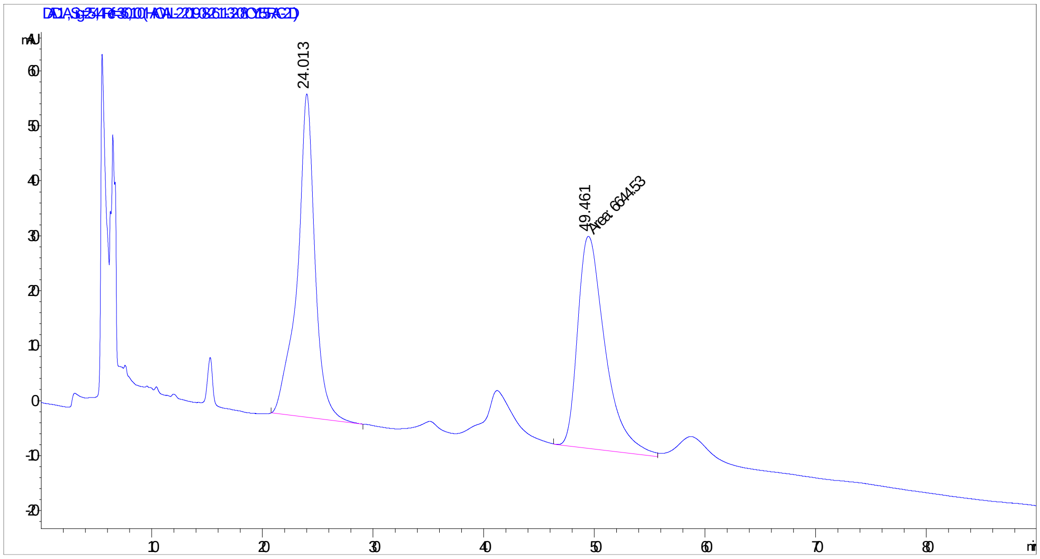

#	Time	Area	Height	Width	Area%	Symmetry
1	24.494	1923.3	15.2	1.7255	50.452	1.125
2	50.982	1888.8	9.8	3.2187	49.548	0.579

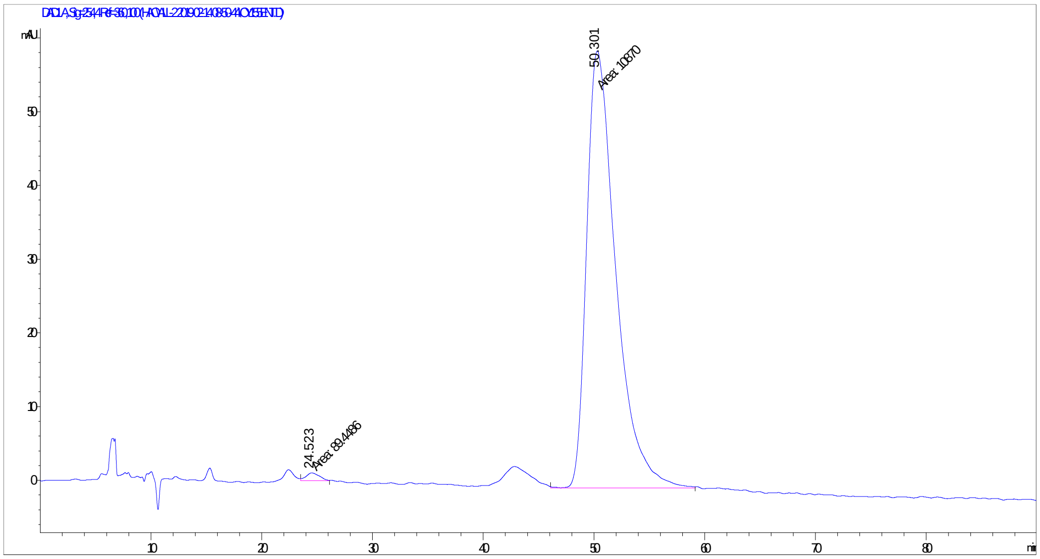

#	Time	Area	Height	Width	Area%	Symmetry
1	24.523	89.4	1.1	1.415	0.816	0.752
2	50.301	10870	59.3	3.0551	99.184	0.552

^{1}H NMR

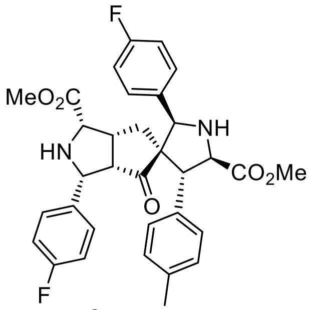

3o

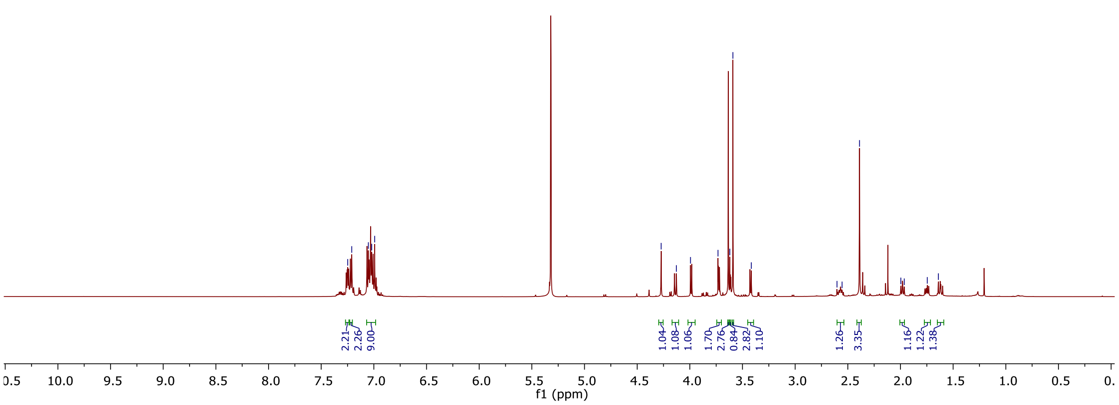

^{13}C NMR

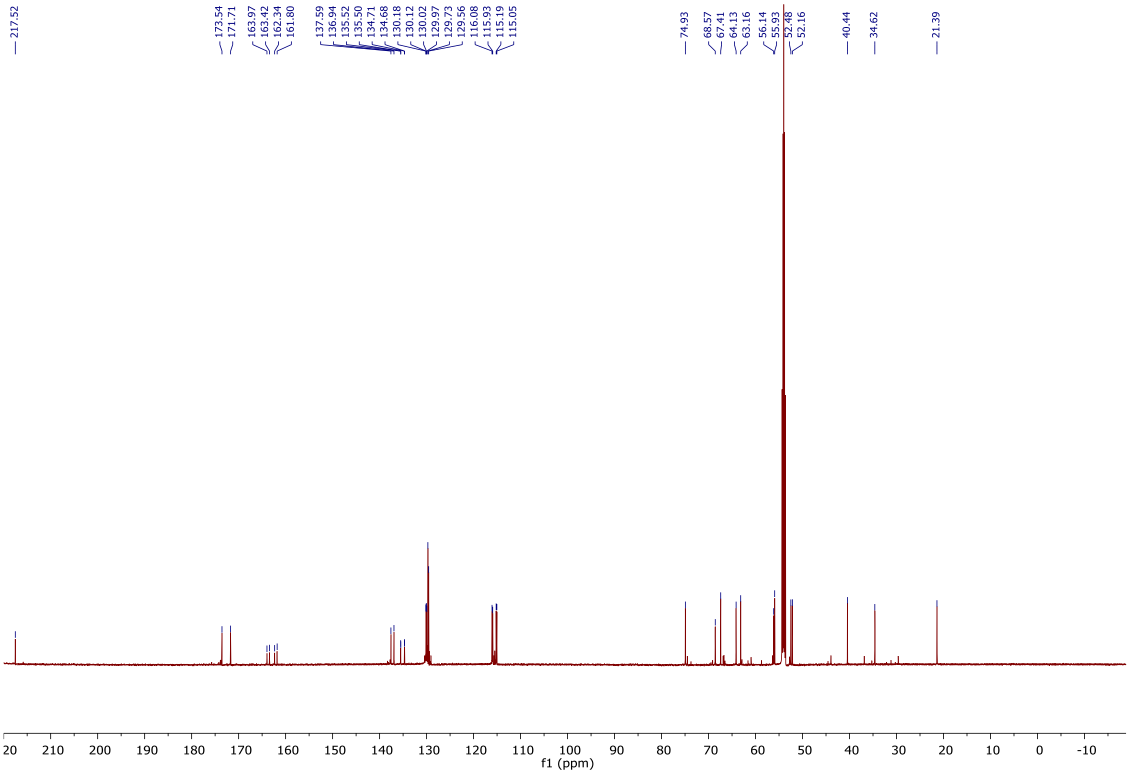

^{19}F NMR

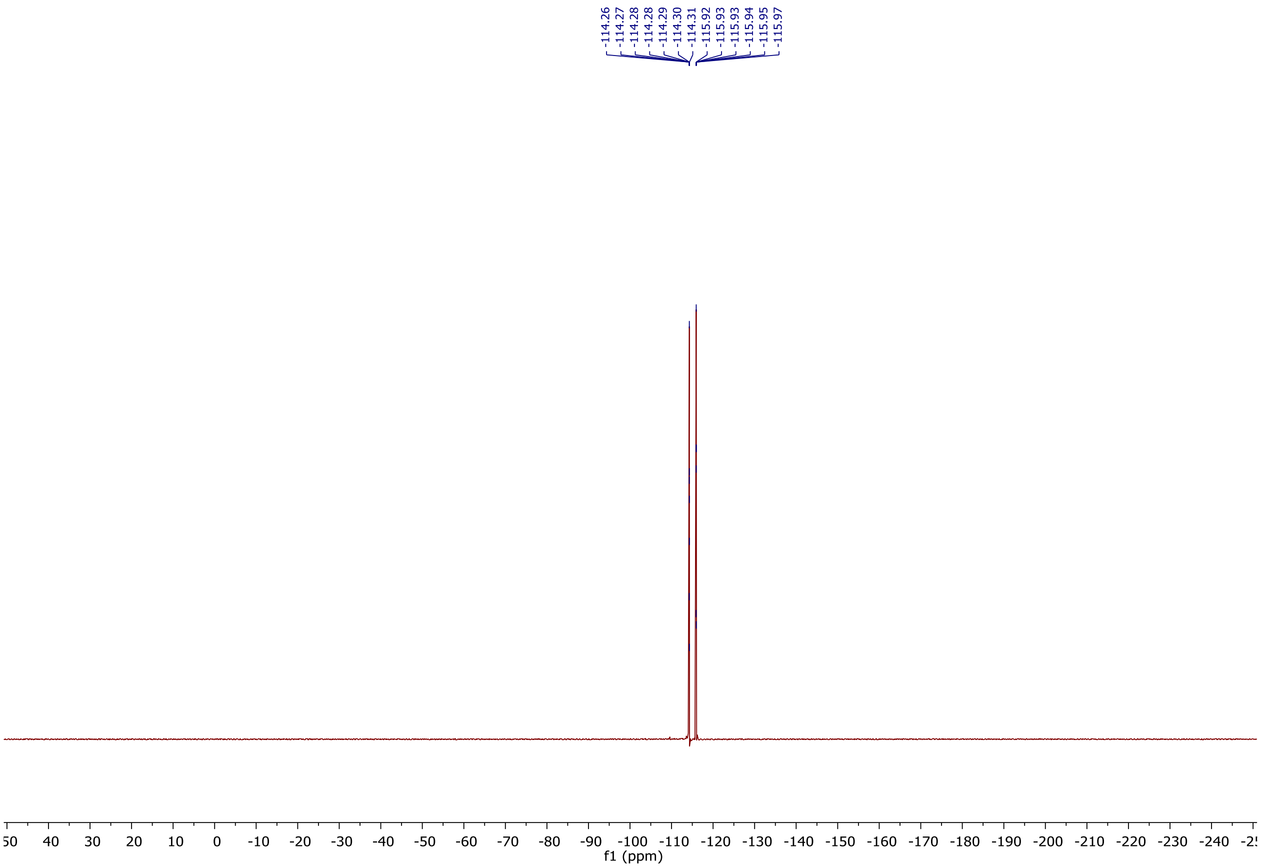

-114.26
-114.27
-114.28
-114.28
-114.29
-114.30
-114.31
-115.92
-115.93
-115.93
-115.94
-115.95
-115.97
50 40 30 20 10 0 -10 -20 -30 -40 -50 -60 -70 -80 -90 -100 -110 -120 -130 -140 -150 -160 -170 -180 -190 -200 -210 -220 -230 -240
f1 (ppm)

HPLC traces for **3o**: racemic top, enantiomer 1 bottom

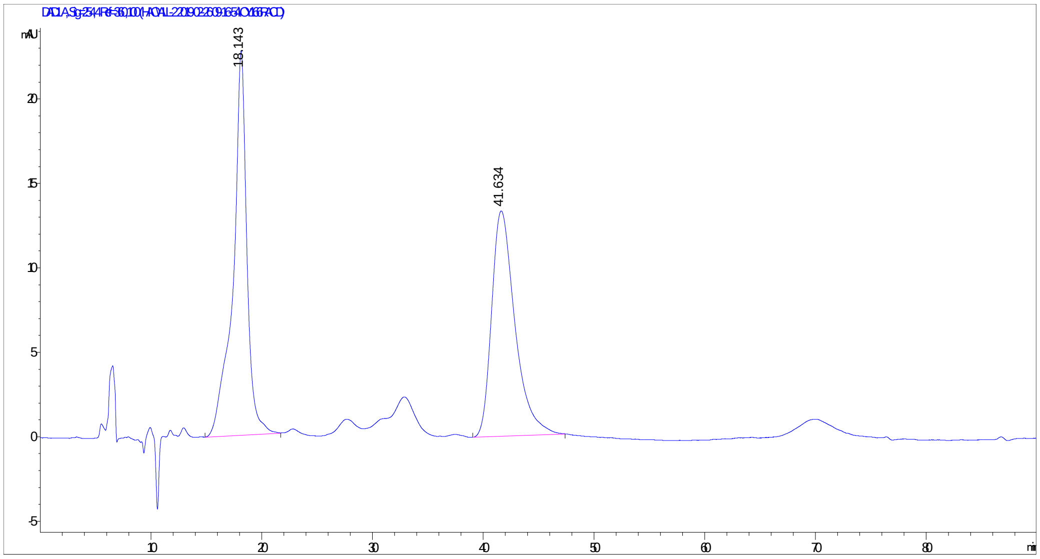

#	Time	Area	Height	Width	Area%	Symmetry
1	18.143	1971.4	22.8	1.2042	50.219	1.378
2	41.634	1954.2	13.3	1.7926	49.781	0.663

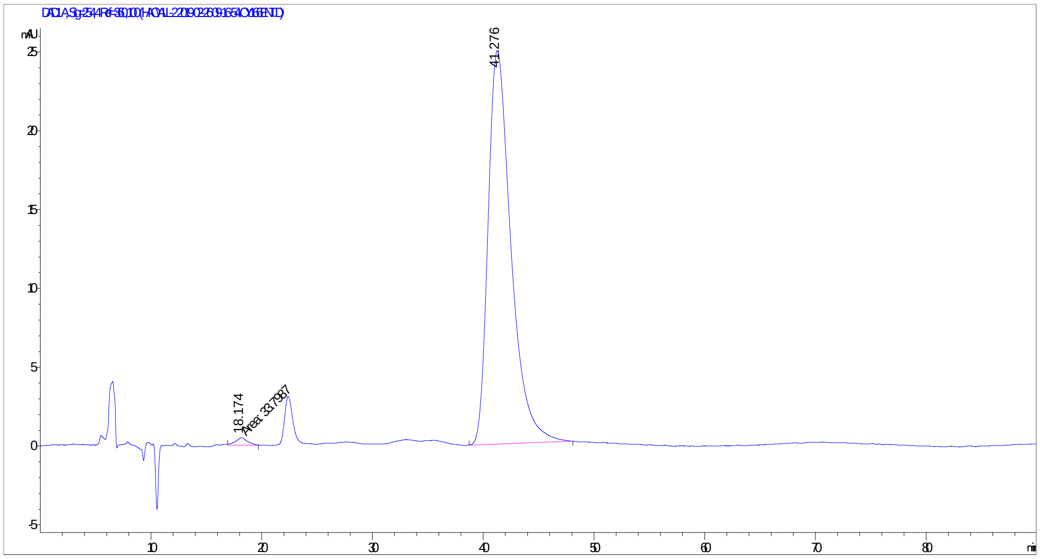

#	Time	Area	Height	Width	Area%	Symmetry
1	18.23	34.4	4.7E-1	1.2304	0.949	0.806
2	41.276	3591.5	25	2.0393	99.051	0.625

^{1}H NMR

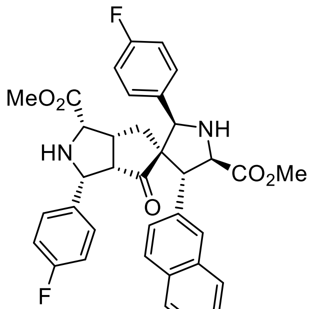

3p

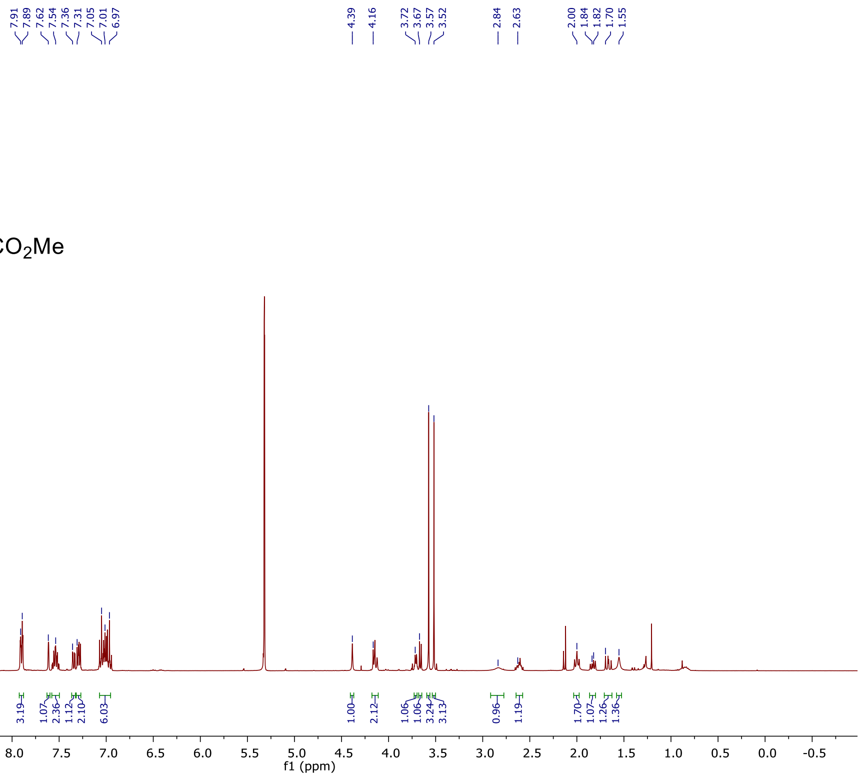

^{13}C NMR

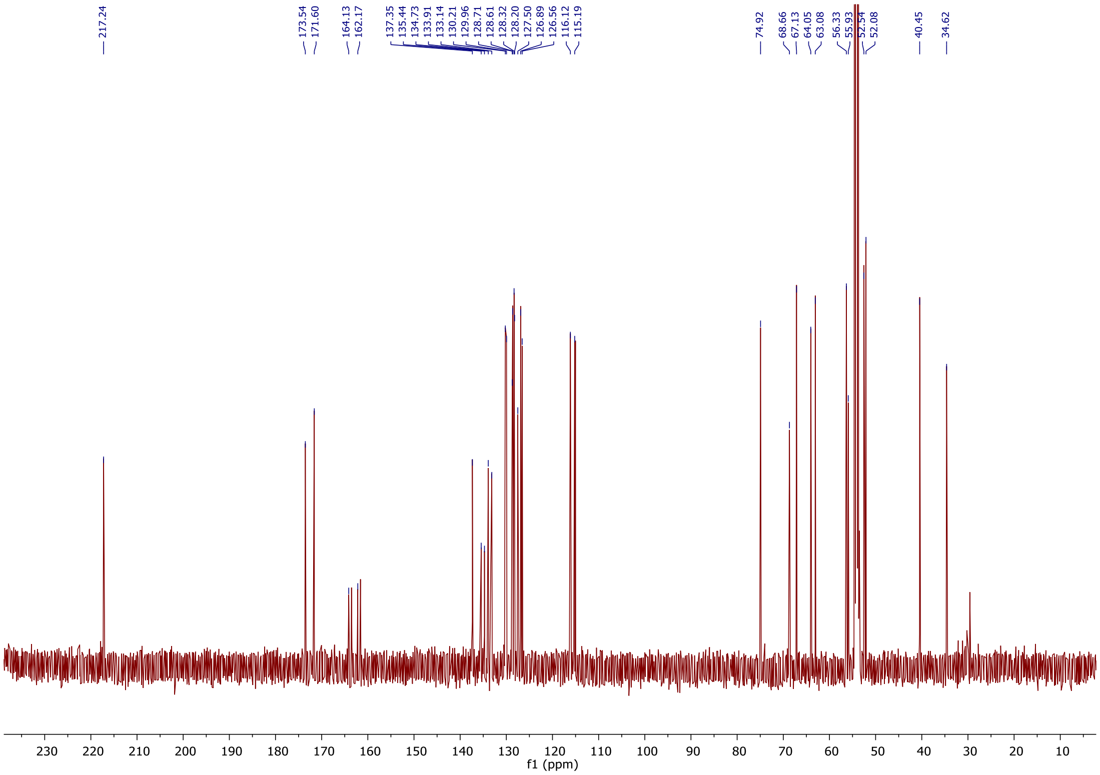

¹⁹F NMR

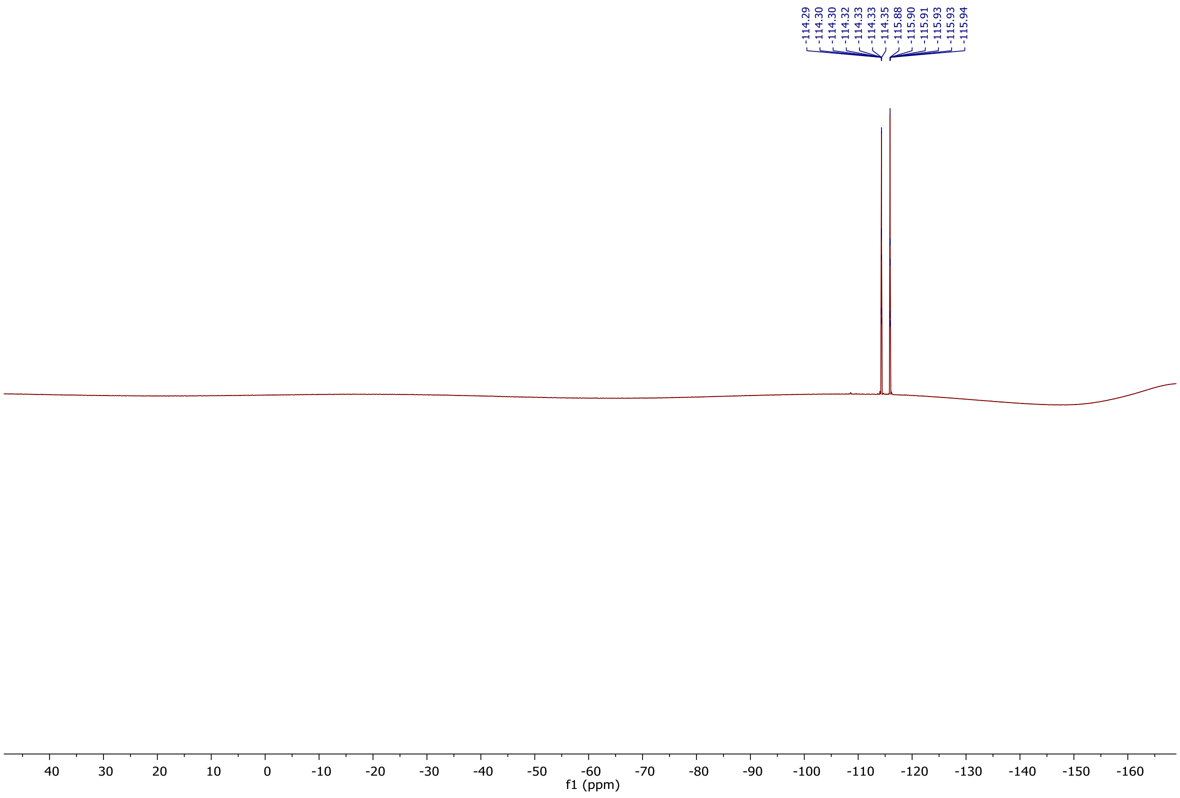
-114.29
-114.30
-114.30
-114.32
-114.33
-114.33
-114.35
-115.88
-115.90
-115.91
-115.93
-115.93
-115.94
40 30 20 10 0 -10 -20 -30 -40 -50 -60 -70 -80 -90 -100 -110 -120 -130 -140 -150 -160
f1 (ppm)

HPLC traces for **3p**: racemic top, enantiomer 1 bottom

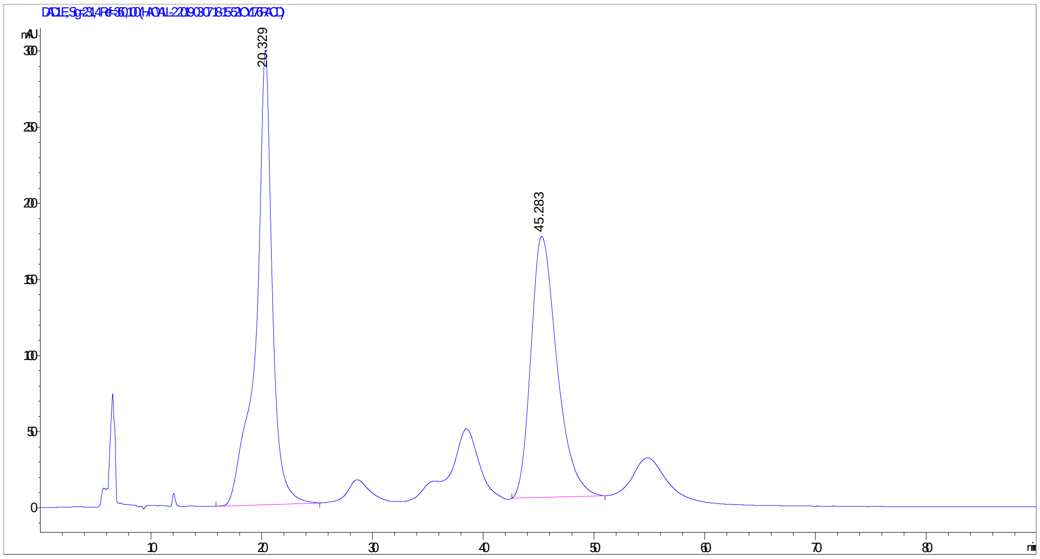

#	Time	Area	Height	Width	Area%	Symmetry
1	20.329	30458.1	298.3	1.4171	52.010	1.322
2	45.283	28104.4	171.5	2.2357	47.990	0.668

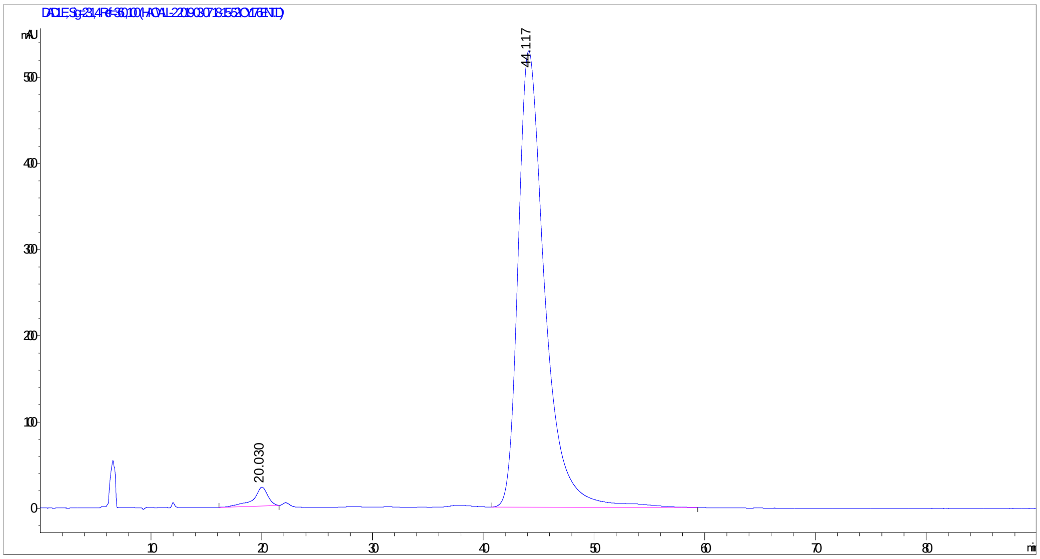

#	Time	Area	Height	Width	Area%	Symmetry
1	20.03	1991	21.8	1.2644	2.194	1.568
2	44.117	88738.3	529.6	2.3468	97.806	0.608

^{1}H NMR

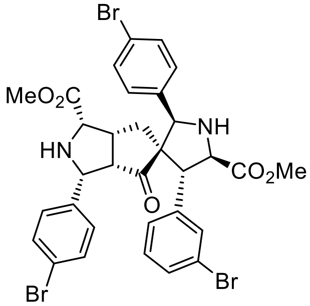

3q

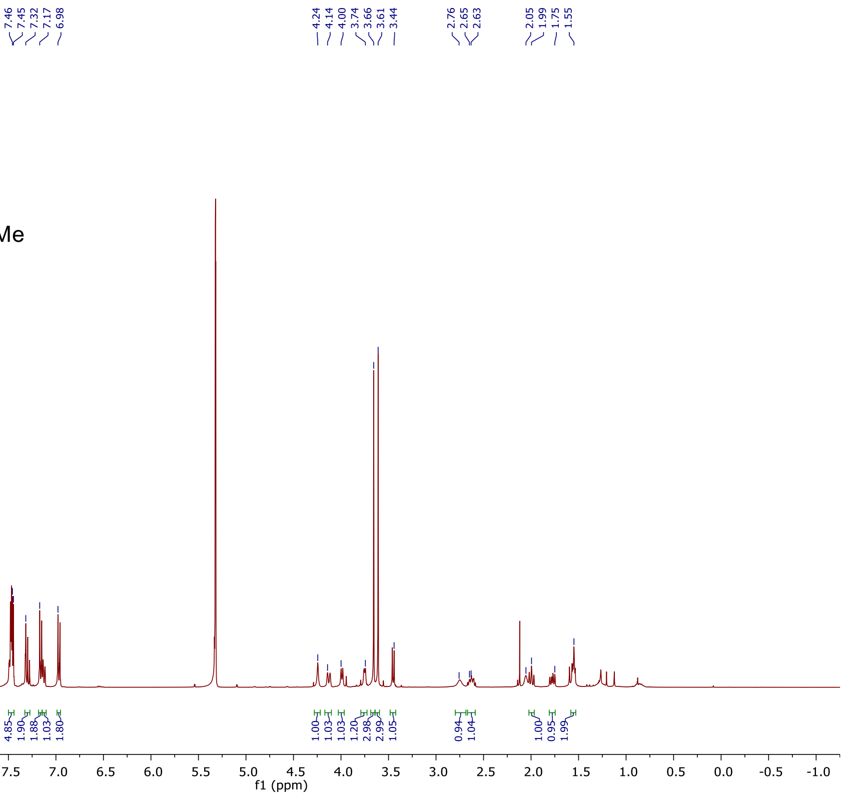

^{13}C NMR

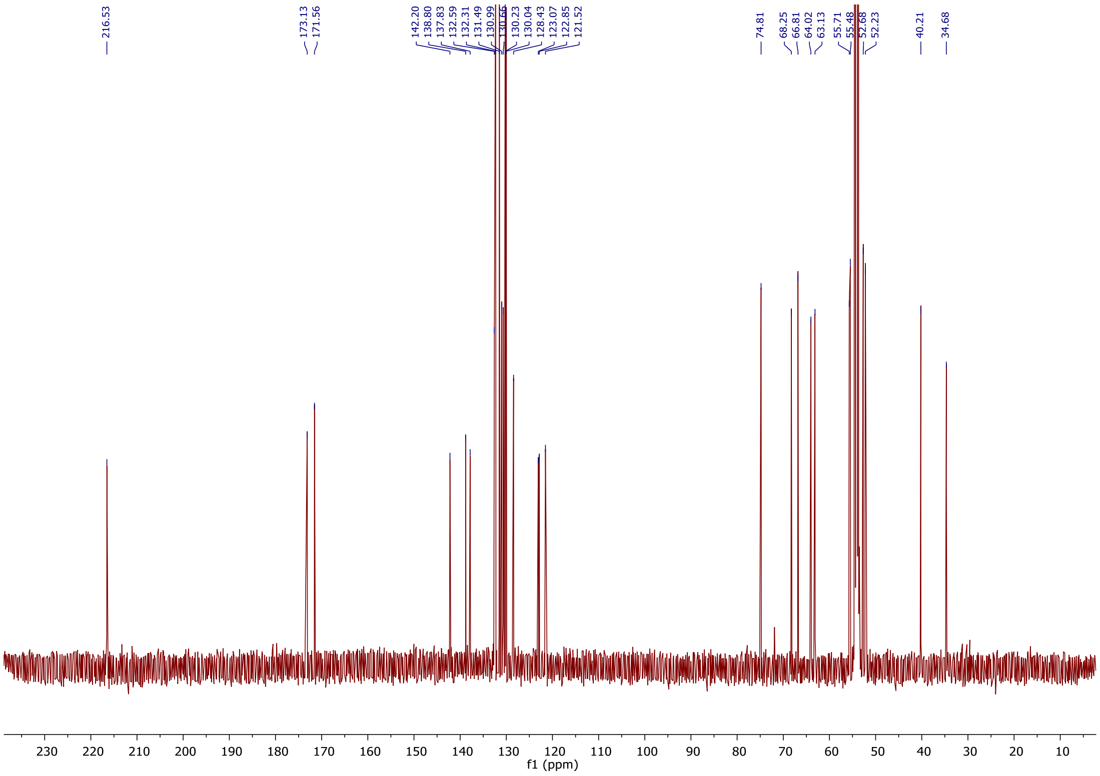

HPLC traces for **3q**: racemic top, enantiomer 1 bottom

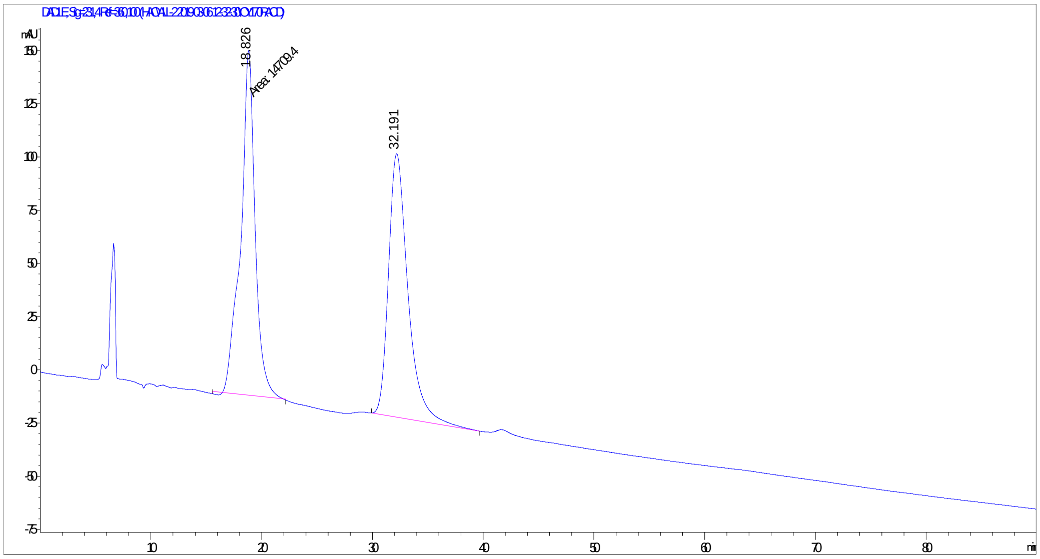

#	Time	Area	Height	Width	Area%	Symmetry
1	18.826	14709.4	161.9	1.5146	49.335	1.184
2	32.191	15106.2	123.8	1.7958	50.665	0.667

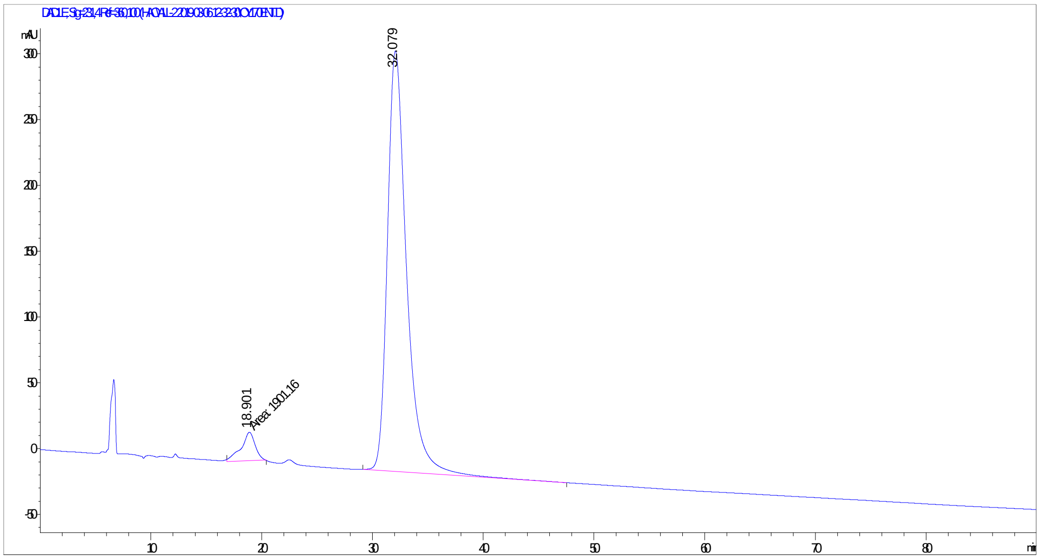

#	Time	Area	Height	Width	Area%	Symmetry
1	18.901	1901.2	21.5	1.4704	4.713	1.448
2	32.079	38435.4	319.6	1.7789	95.287	0.656

^{1}H NMR

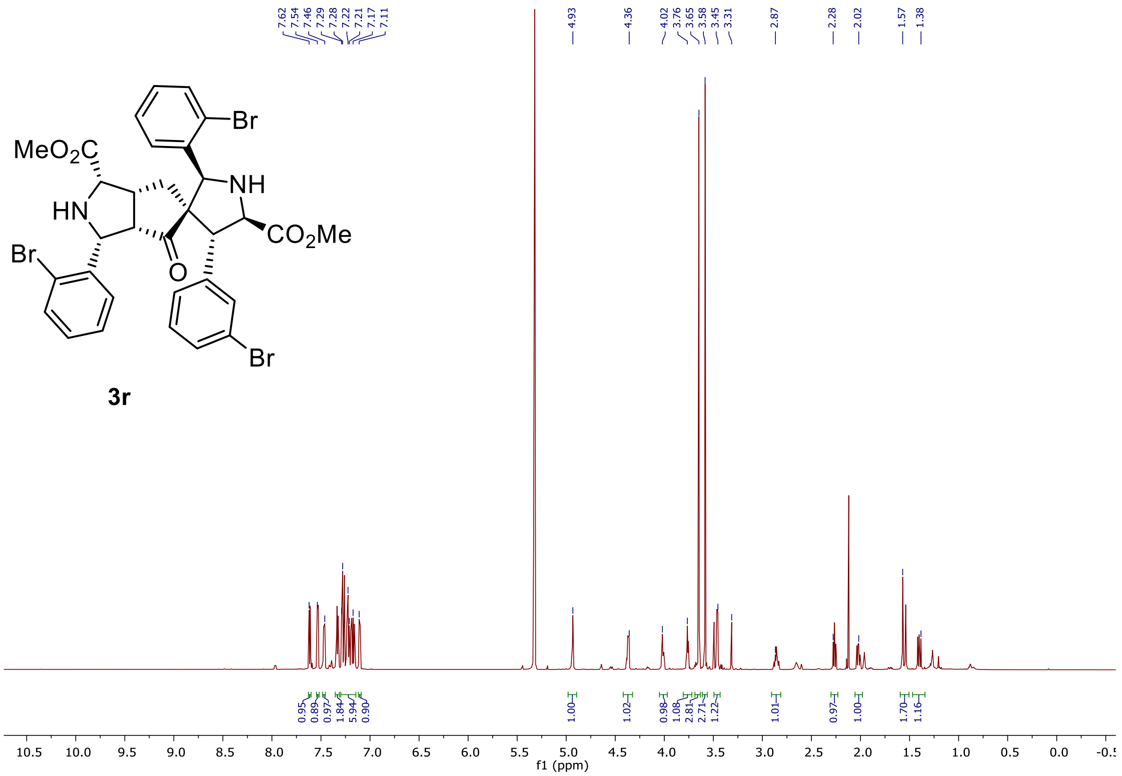

^{13}C NMR

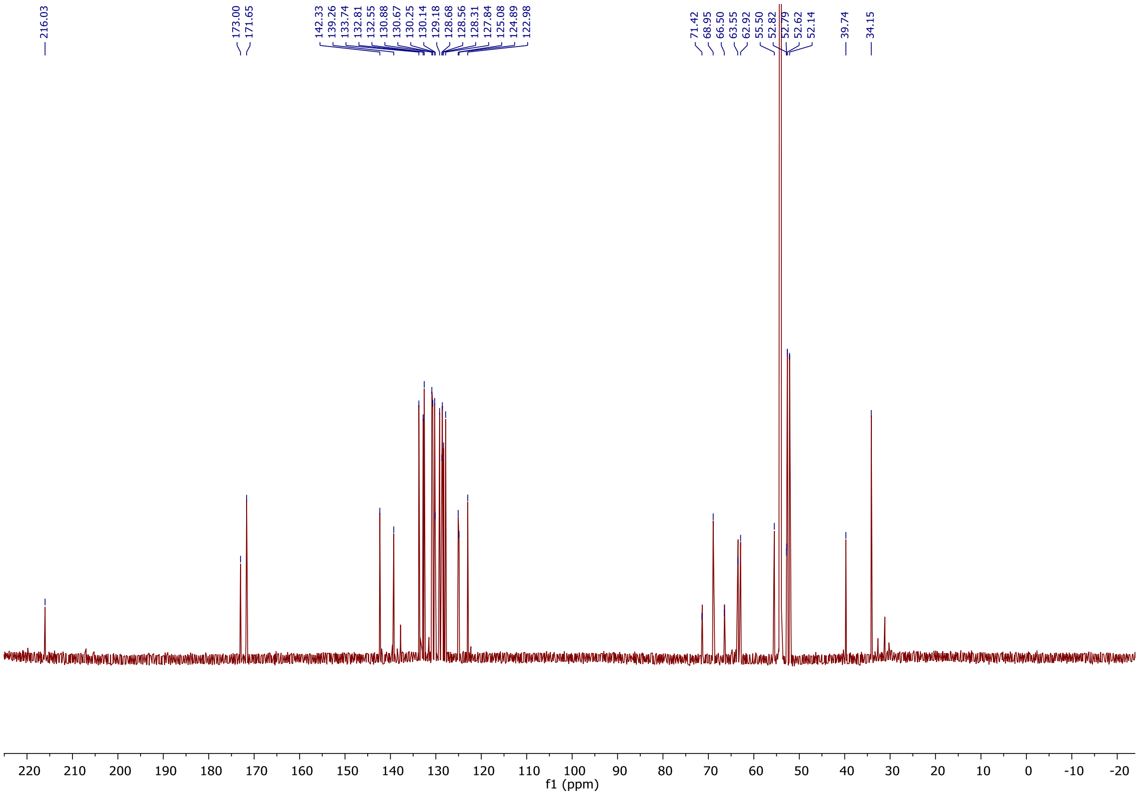

HPLC traces for **3r**: racemic top, enantiomer 1 bottom

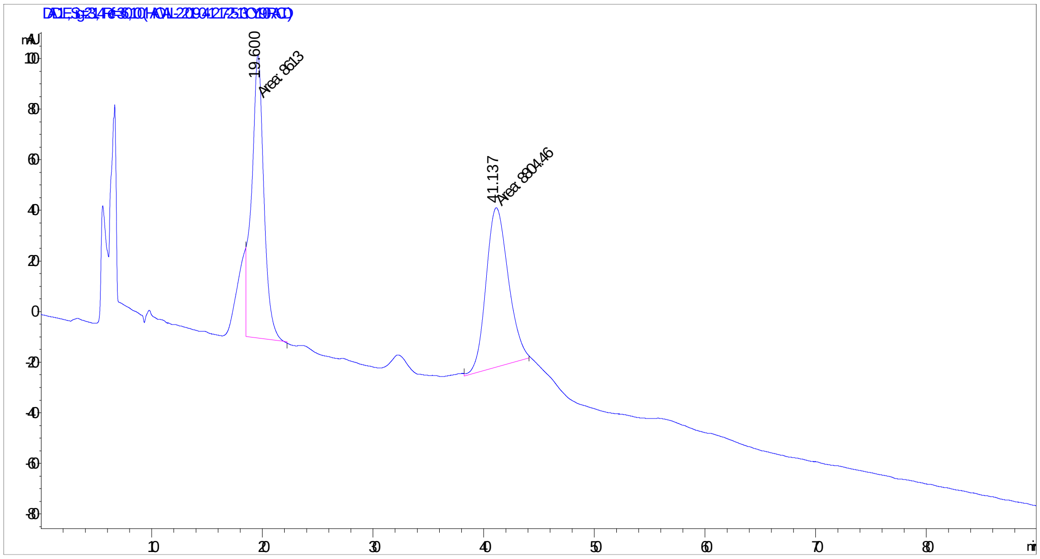

#	Time	Area	Height	Width	Area%	Symmetry
1	19.6	8613	112.1	1.2801	49.450	1.02
2	41.137	8804.5	63	2.3284	50.550	0.833

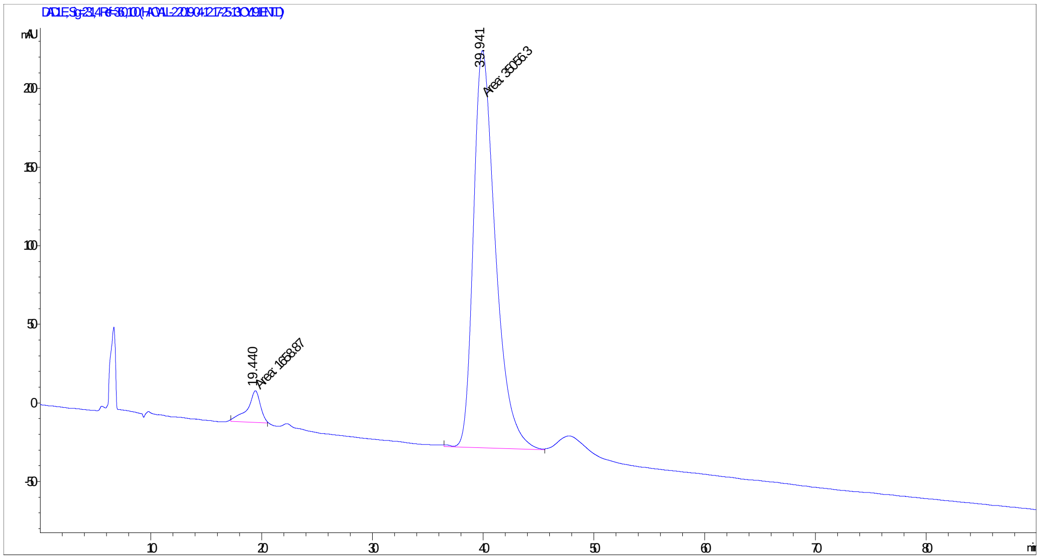

#	Time	Area	Height	Width	Area%	Symmetry
1	19.44	1658.9	20.2	1.3672	4.518	1.595
2	39.941	35056.3	252.7	2.312	95.482	0.669

^{1}H NMR

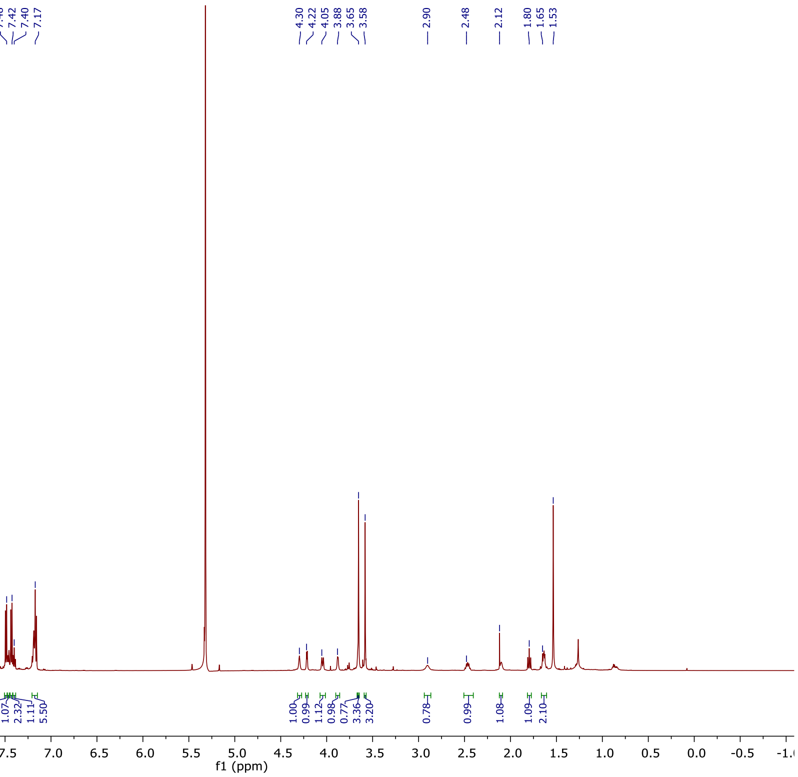

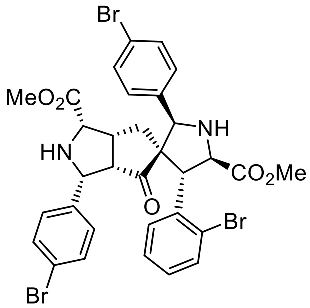

3s

^{13}C NMR

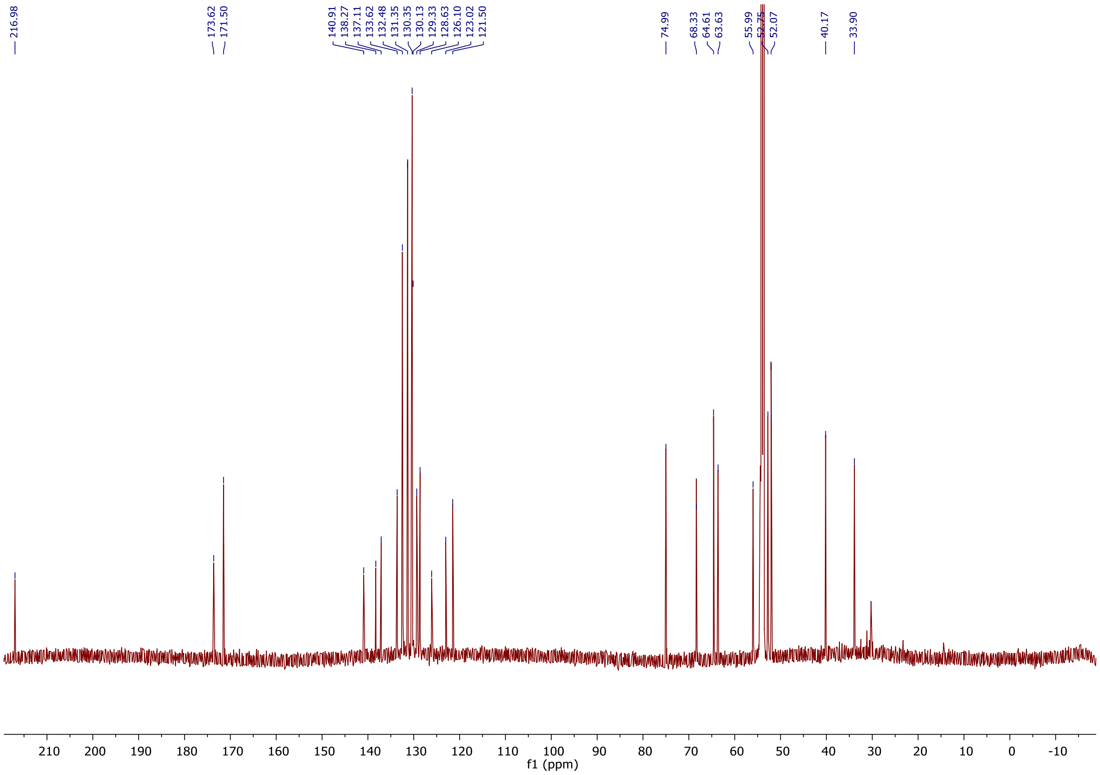

HPLC traces for **3s**: racemic top, enantiomer 1 bottom

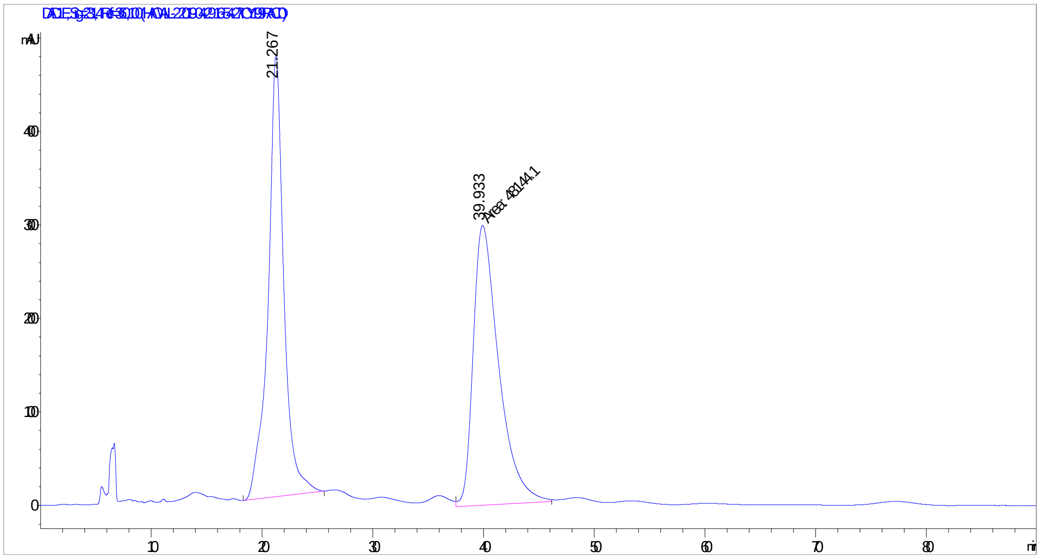

#	Time	Area	Height	Width	Area%	Symmetry
1	21.267	46853.8	473.5	1.4084	49.321	0.976
2	39.933	48144.1	299.4	2.6799	50.679	0.567

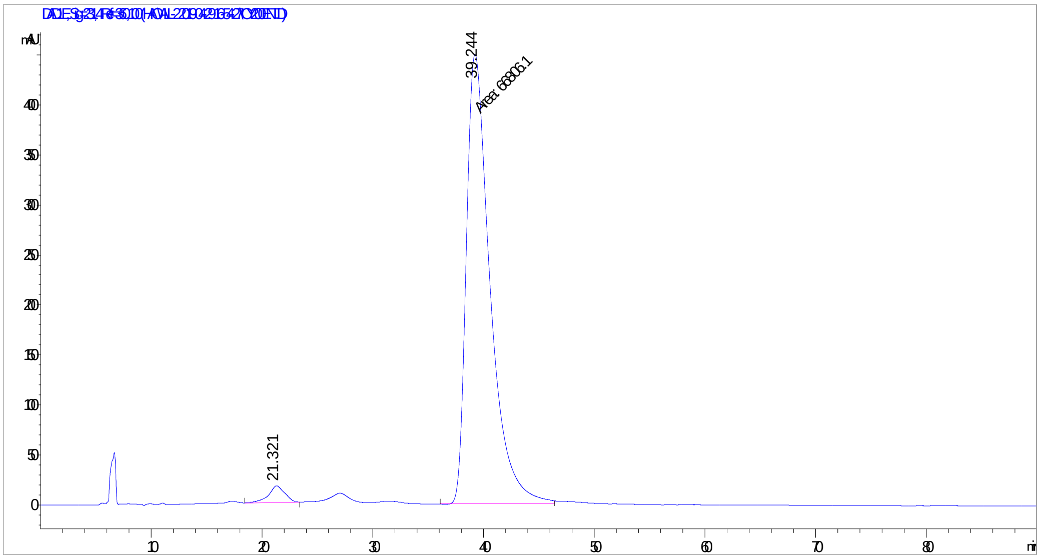

#	Time	Area	Height	Width	Area%	Symmetry
1	21.321	1650.9	16.5	1.3783	2.412	1.013
2	39.244	66806.1	448.9	2.4804	97.588	0.549

^{1}H NMR

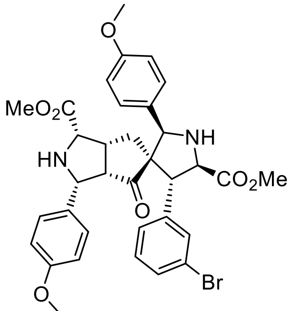

3t

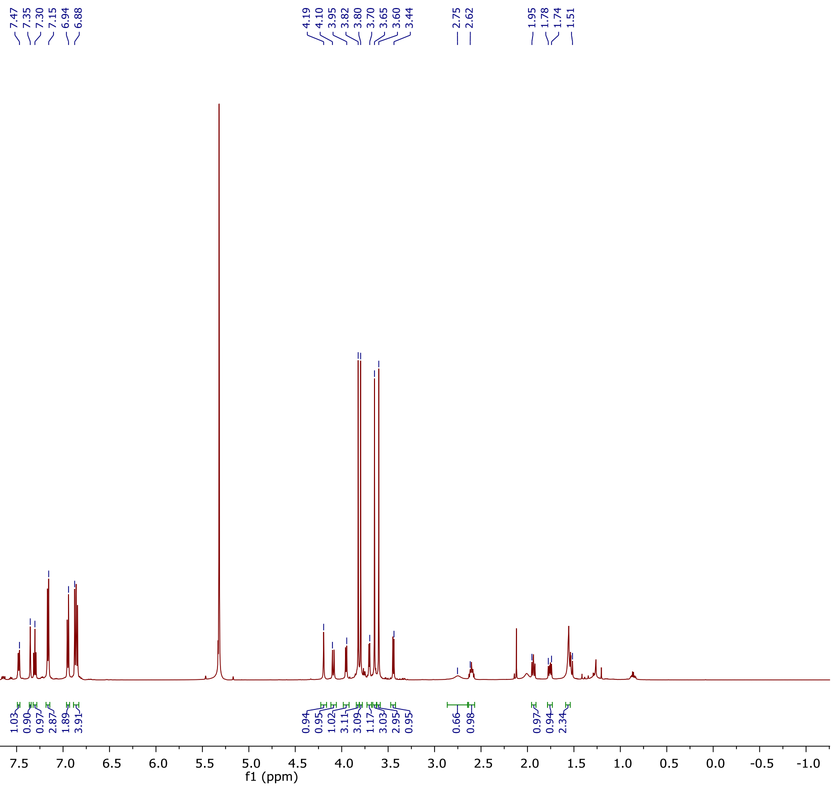

^{1}C NMR

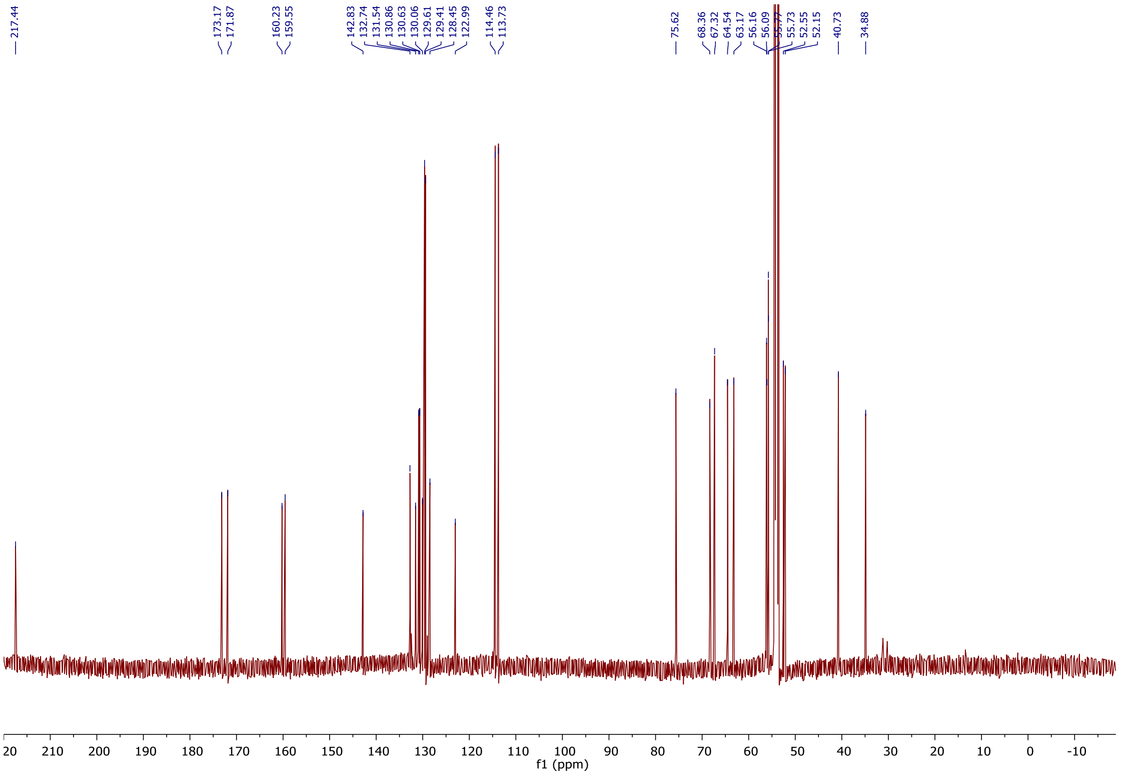

HPLC traces for **3t**: racemic top, enantiomer 1 bottom

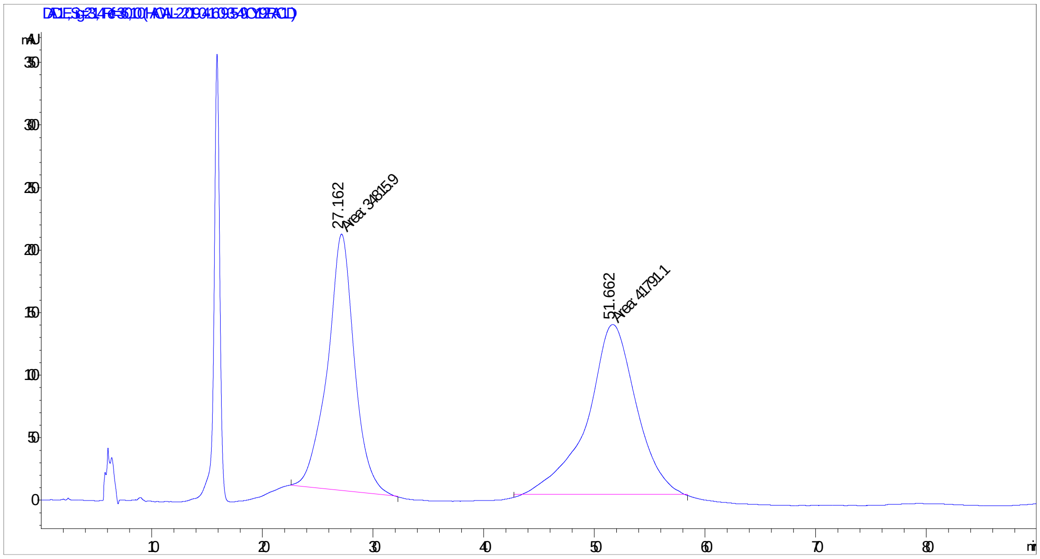

#	Time	Area	Height	Width	Area%	Symmetry
1	27.157	40958	212.9	2.6882	48.665	1.223
2	51.662	43205.2	137.1	5.2526	51.335	1.105

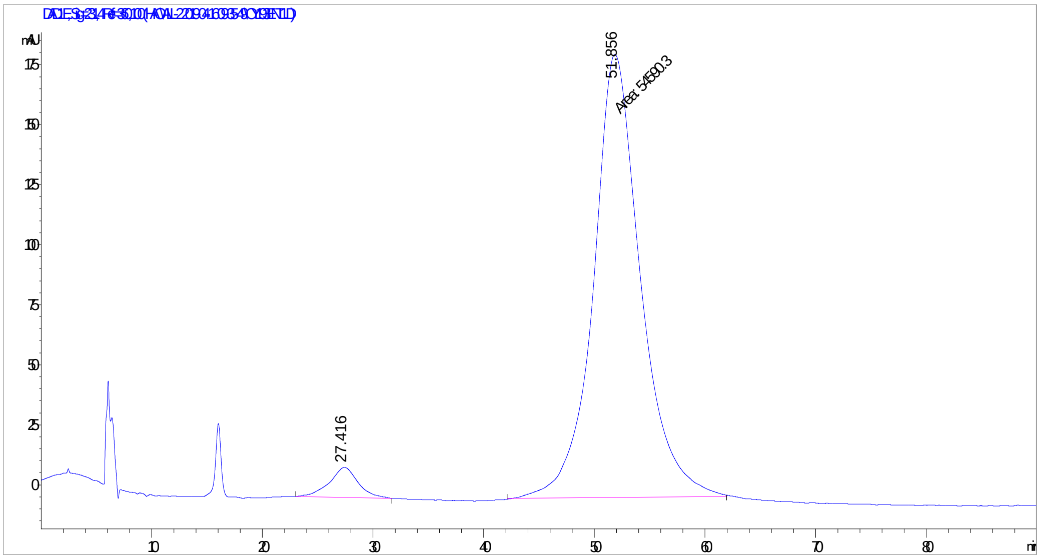

#	Time	Area	Height	Width	Area%	Symmetry
1	27.416	2242.2	12.5	2.1188	4.231	1.075
2	51.856	50746.5	179.8	3.7242	95.769	0.761

^{1}H NMR

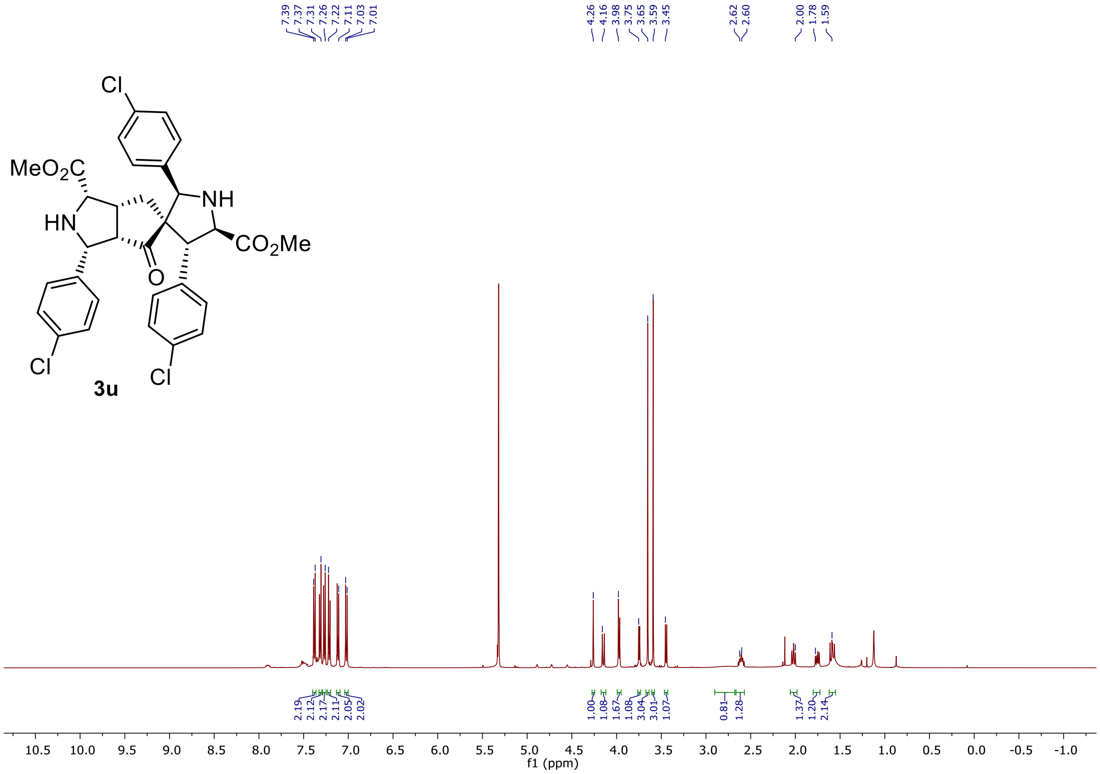

^{13}C NMR

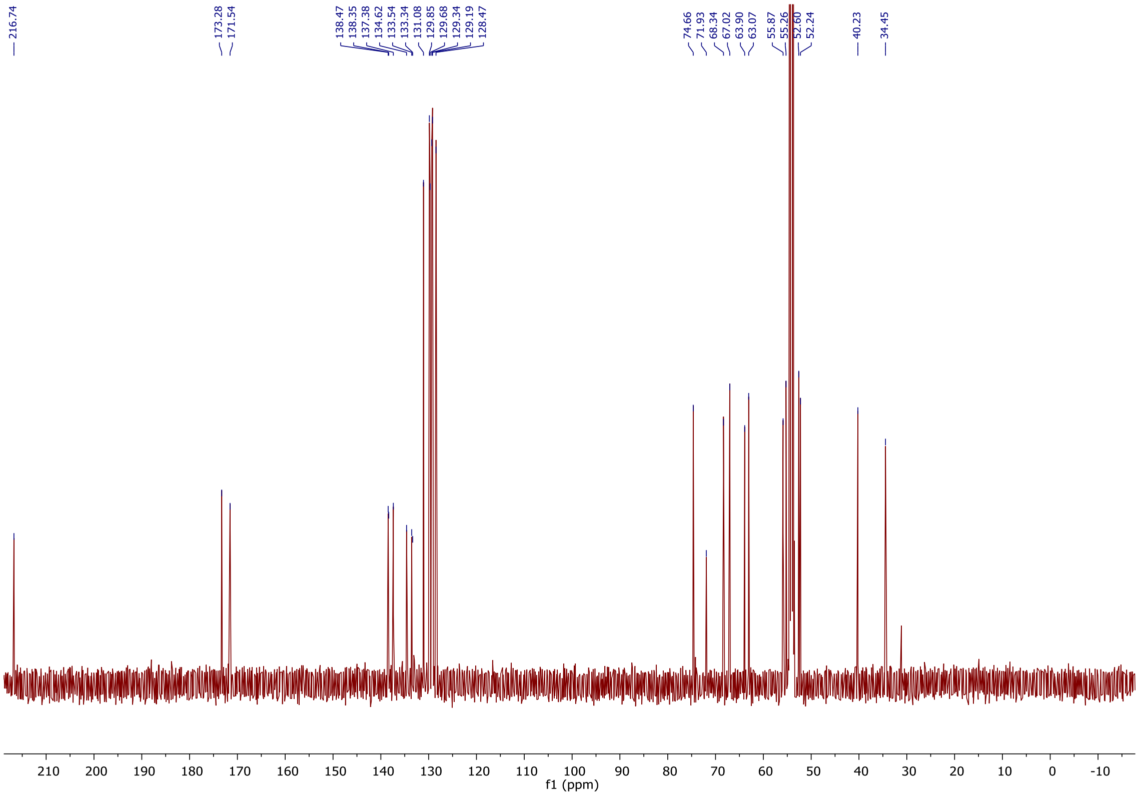

HPLC traces for **3u**: racemic top, enantiomer 1 bottom

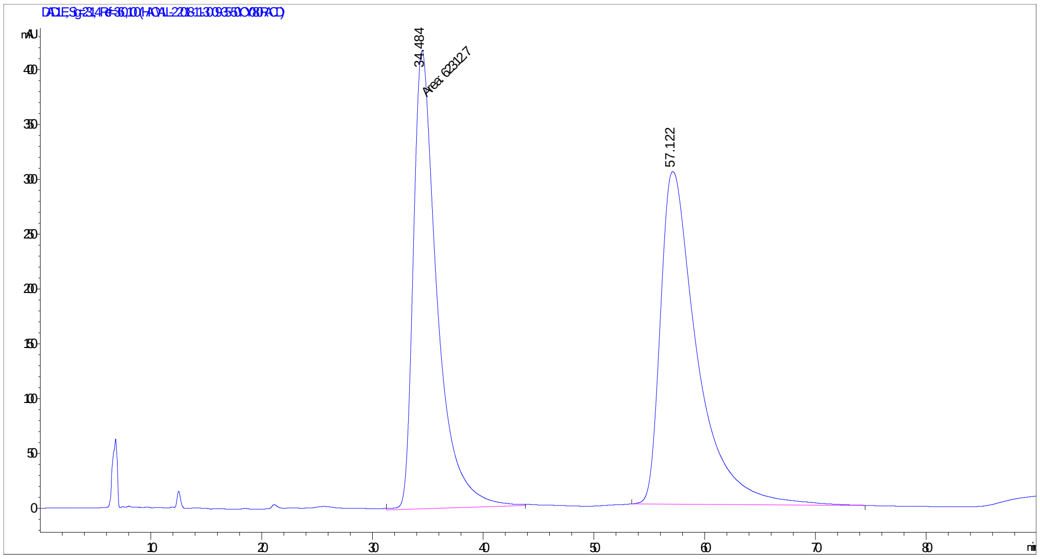

#	Time	Area	Height	Width	Area%	Symmetry
1	34.484	62312.7	417.8	2.4859	47.182	0.551
2	57.122	69756.4	303.2	3.1705	52.818	0.46

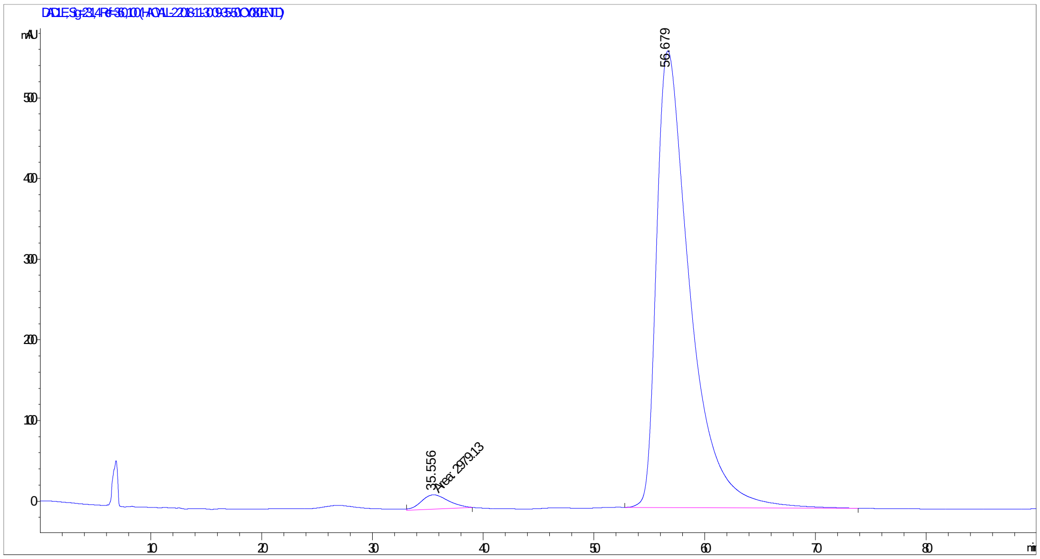

#	Time	Area	Height	Width	Area%	Symmetry
1	35.556	2979.1	17.6	2.0574	2.444	0.887
2	56.679	118932.6	566	2.9657	97.556	0.467

^{1}H NMR

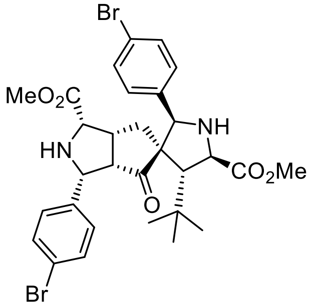

3v

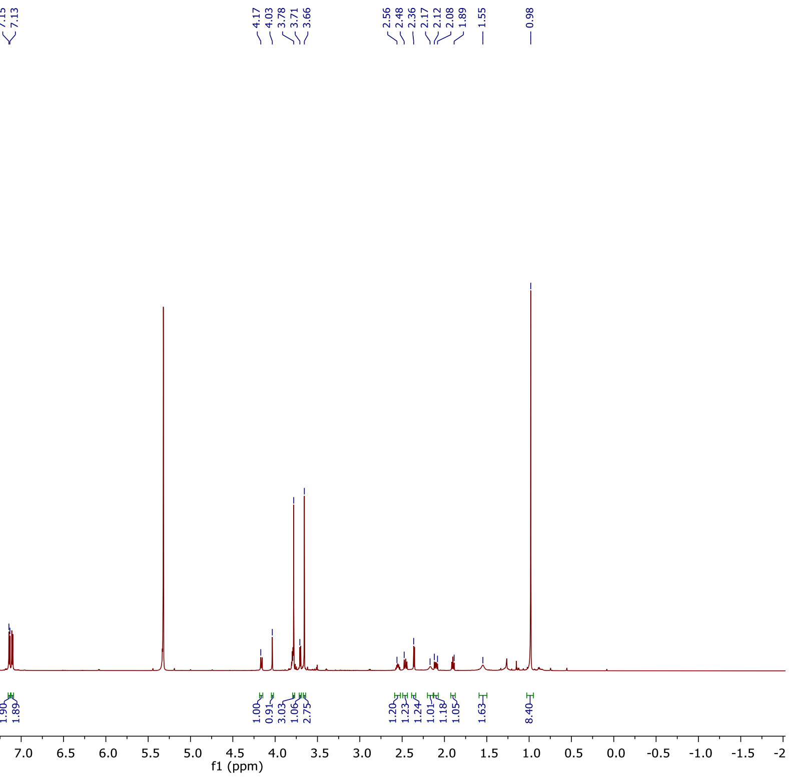

^{13}C NMR

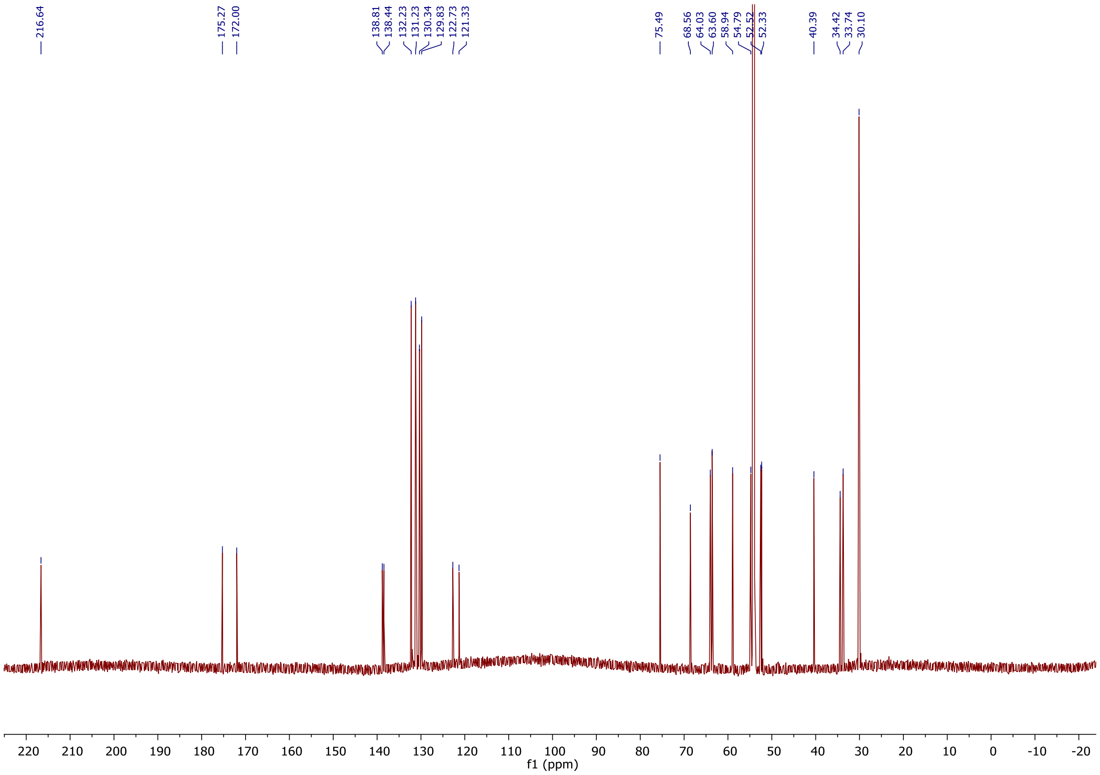

HPLC traces for **3v**: racemic top, enantiomer 1 bottom

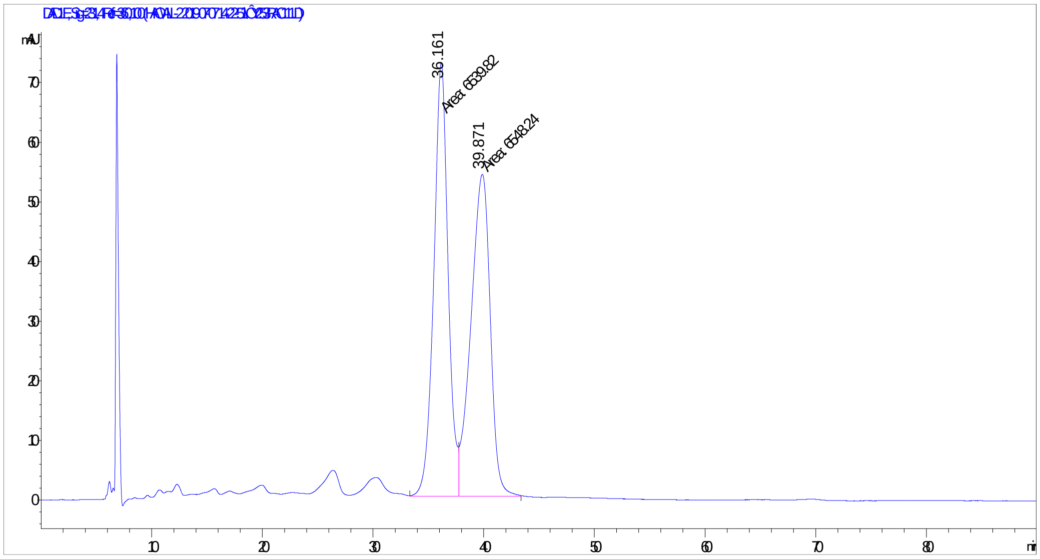

#	Time	Area	Height	Width	Area%	Symmetry
1	36.161	5511.8	67.7	1.2039	50.839	1.162
2	39.871	5329.8	49.4	1.6054	49.161	1.254

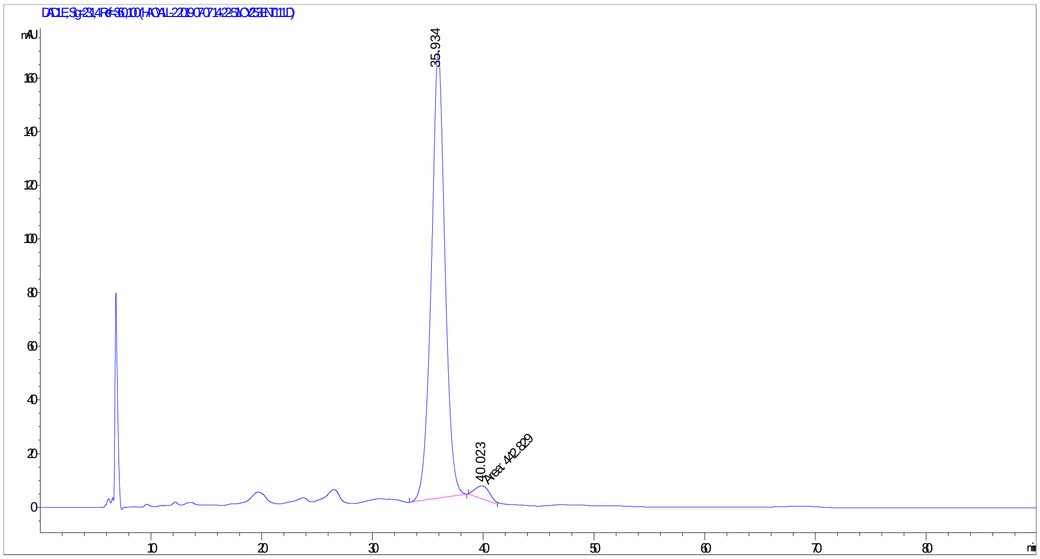

#	Time	Area	Height	Width	Area%	Symmetry
1	35.934	14297.2	166.7	1.2524	96.996	0.976
2	40.023	442.8	4.9	1.5112	3.004	0.774

^{1}H NMR

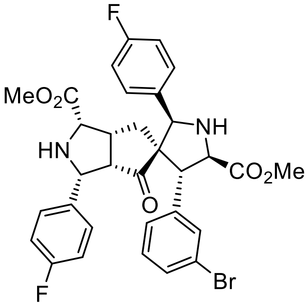

3w

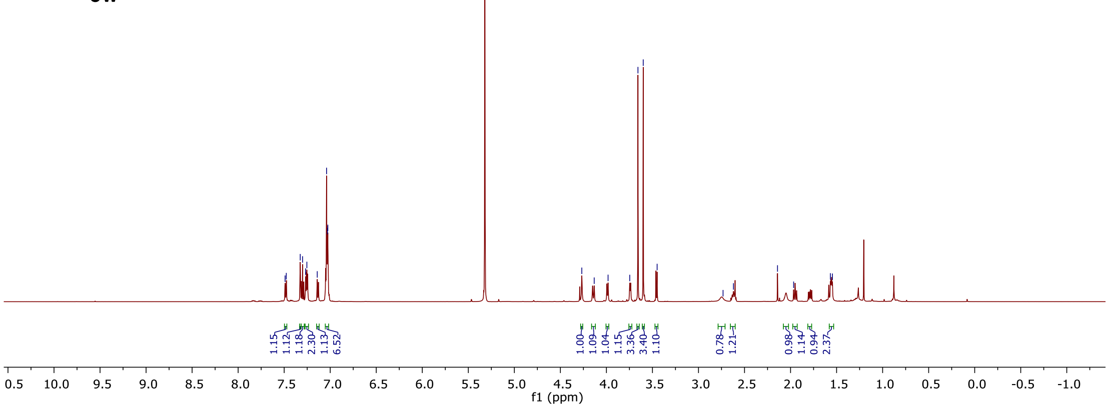

^{13}C NMR

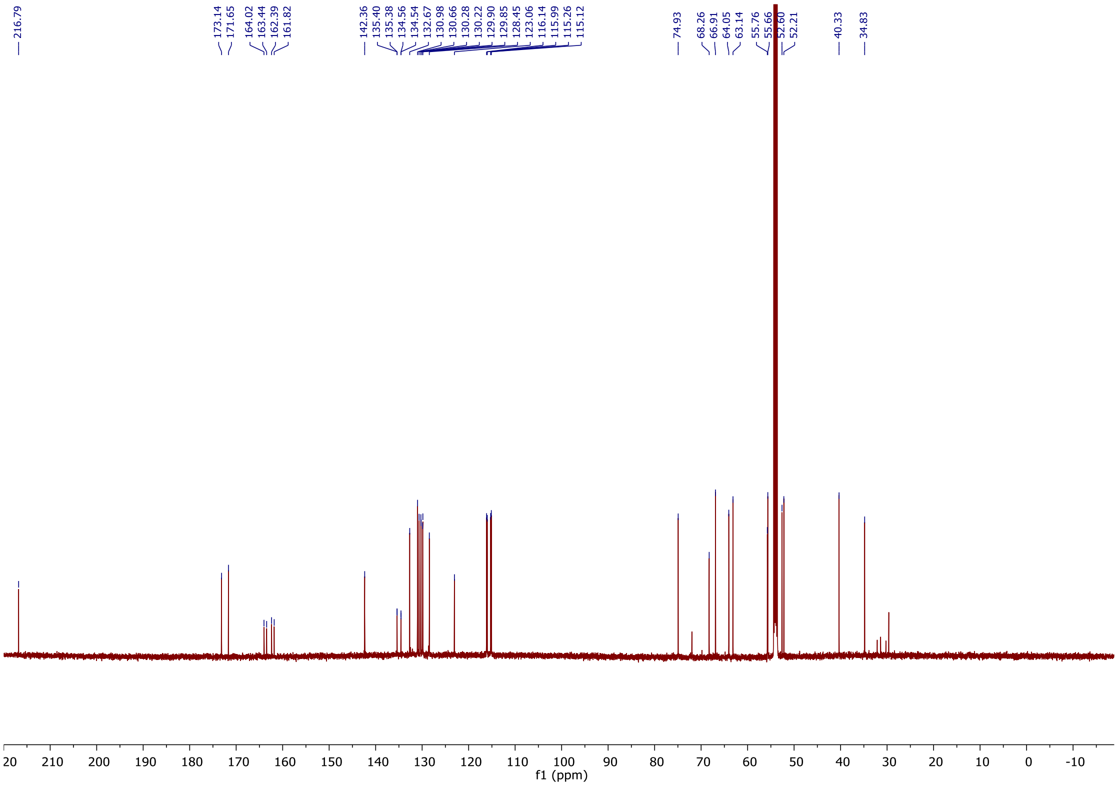

^{19}F NMR

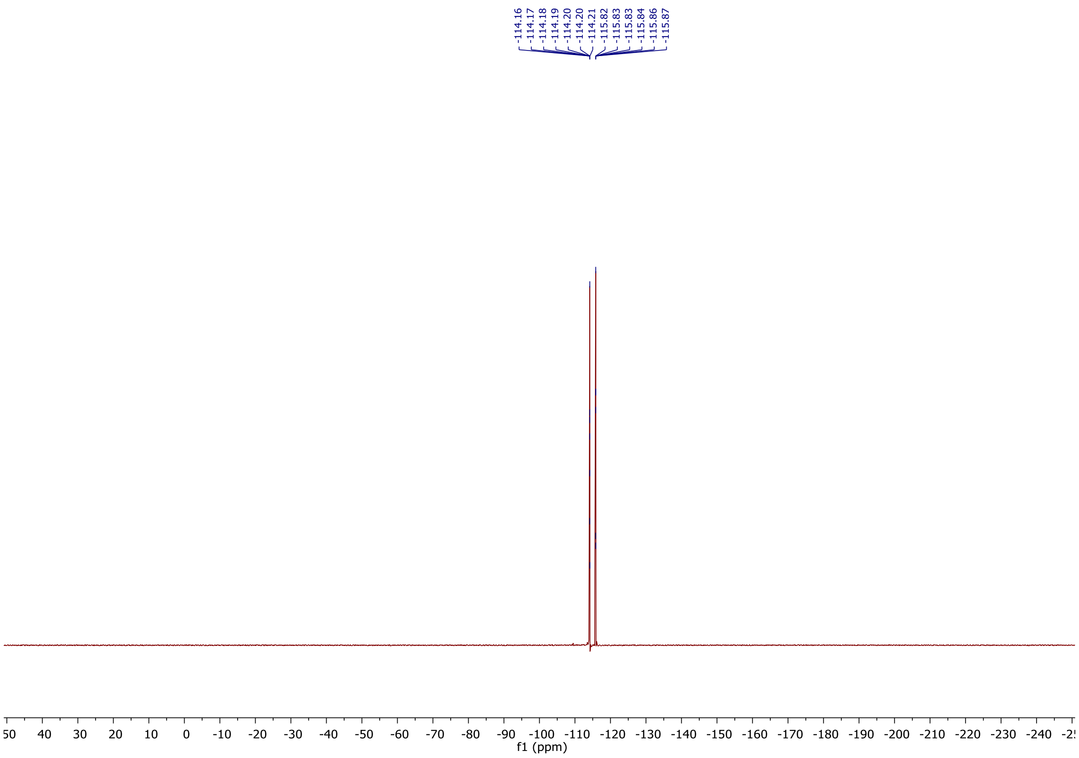

-114.16
-114.17
-114.18
-114.19
-114.20
-114.20
-114.21
-115.82
-115.83
-115.83
-115.84
-115.86
-115.87
f1 (ppm)

HPLC traces for **3w**: racemic top, enantiomer 1 bottom

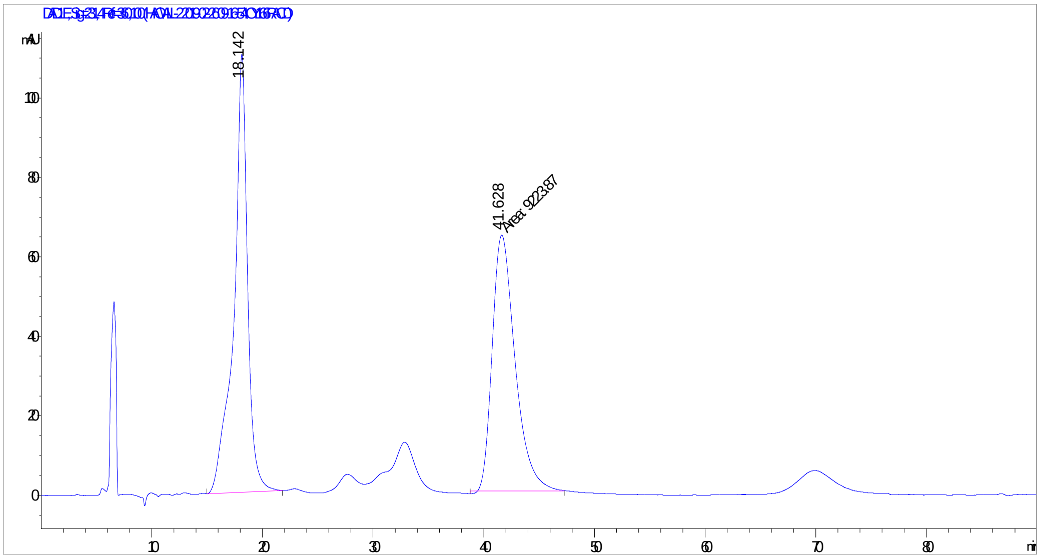

#	Time	Area	Height	Width	Area%	Symmetry
1	18.142	9481.7	110.2	1.1975	50.689	1.384
2	41.628	9223.9	64.3	2.3899	49.311	0.678

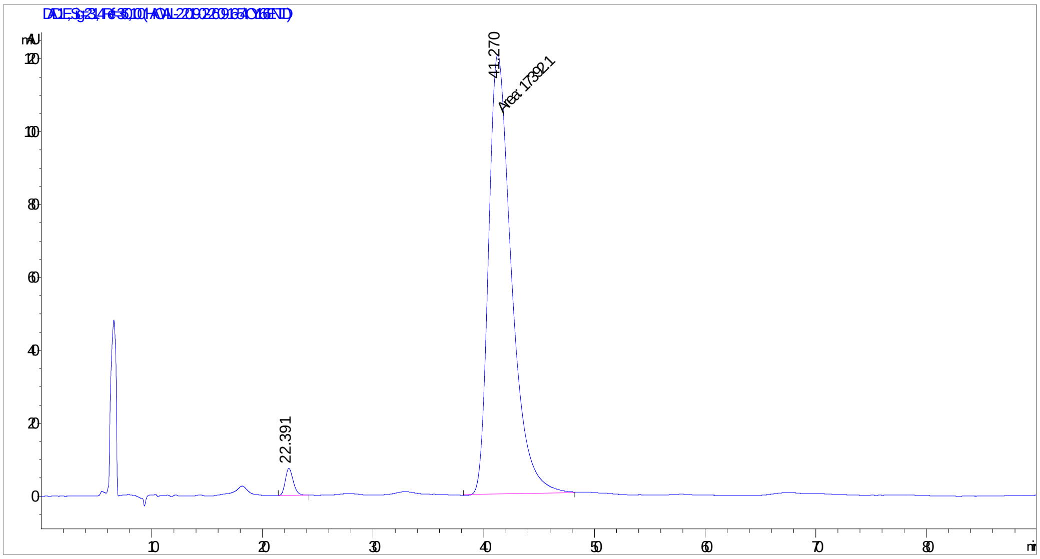

#	Time	Area	Height	Width	Area%	Symmetry
1	22.391	370	7.4	0.7572	2.083	0.767
2	41.27	17392.1	120.6	2.4035	97.917	0.636

^{1}H NMR

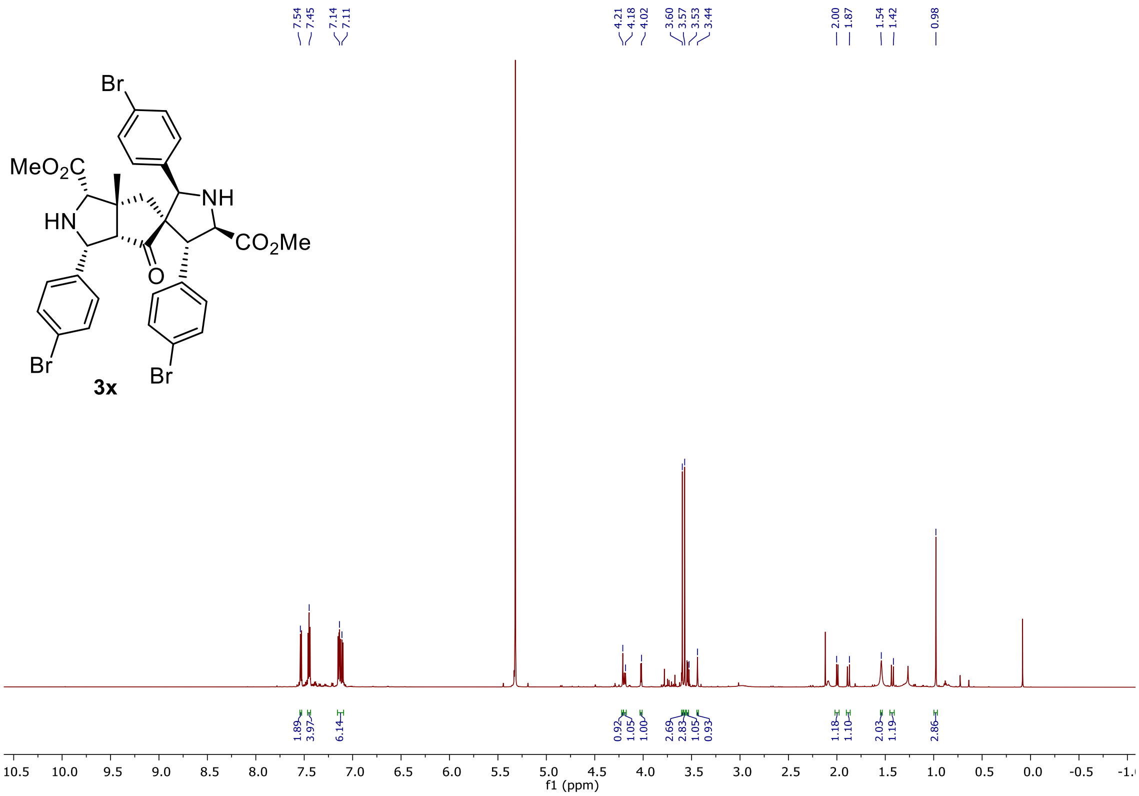

^{13}C NMR

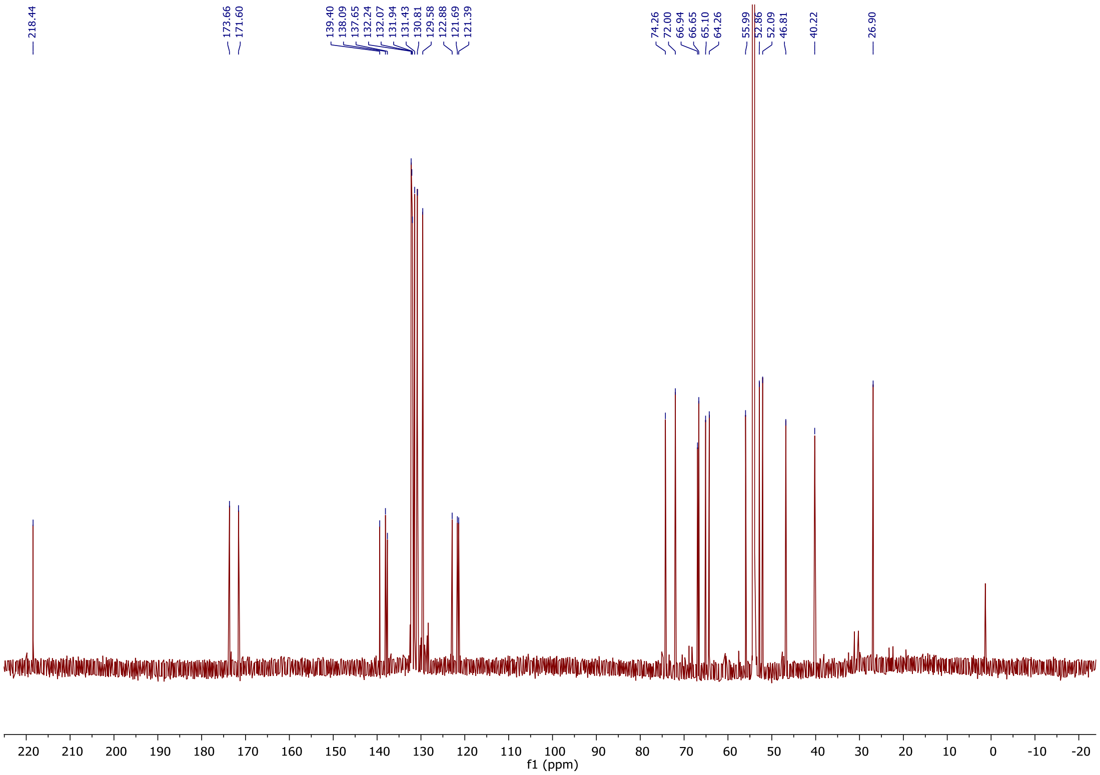

HPLC traces for **3x**: racemic top, enantiomer 1 bottom

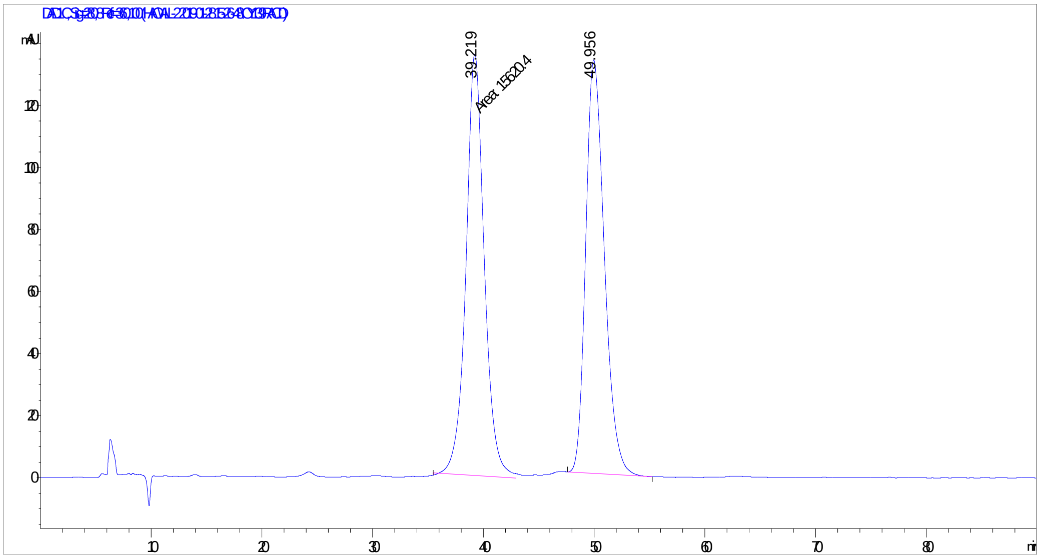

#	Time	Area	Height	Width	Area%	Symmetry
1	39.219	15620.4	135.8	1.9168	50.014	0.9
2	49.956	15611.7	133.2	1.8011	49.986	0.707

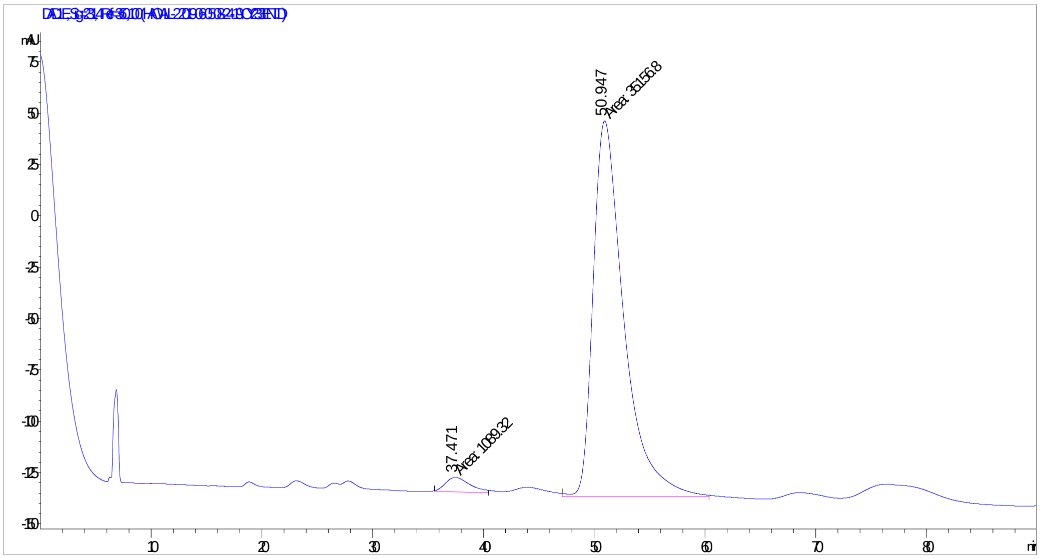

#	Time	Area	Height	Width	Area%	Symmetry
1	37.471	1089.3	7	2.5817	3.005	0.632
2	50.947	35156.8	182.8	3.2053	96.995	0.574

^{1}H NMR

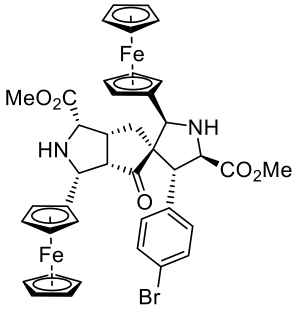

3y

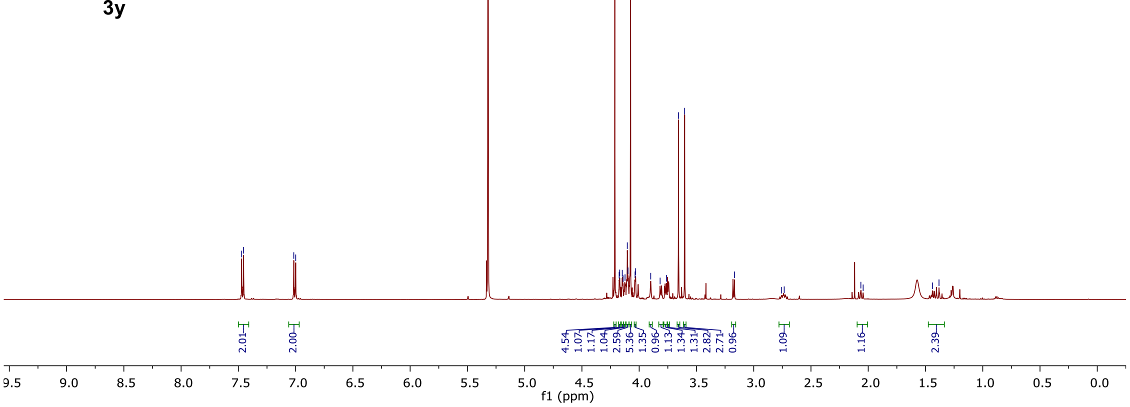

^{13}C NMR

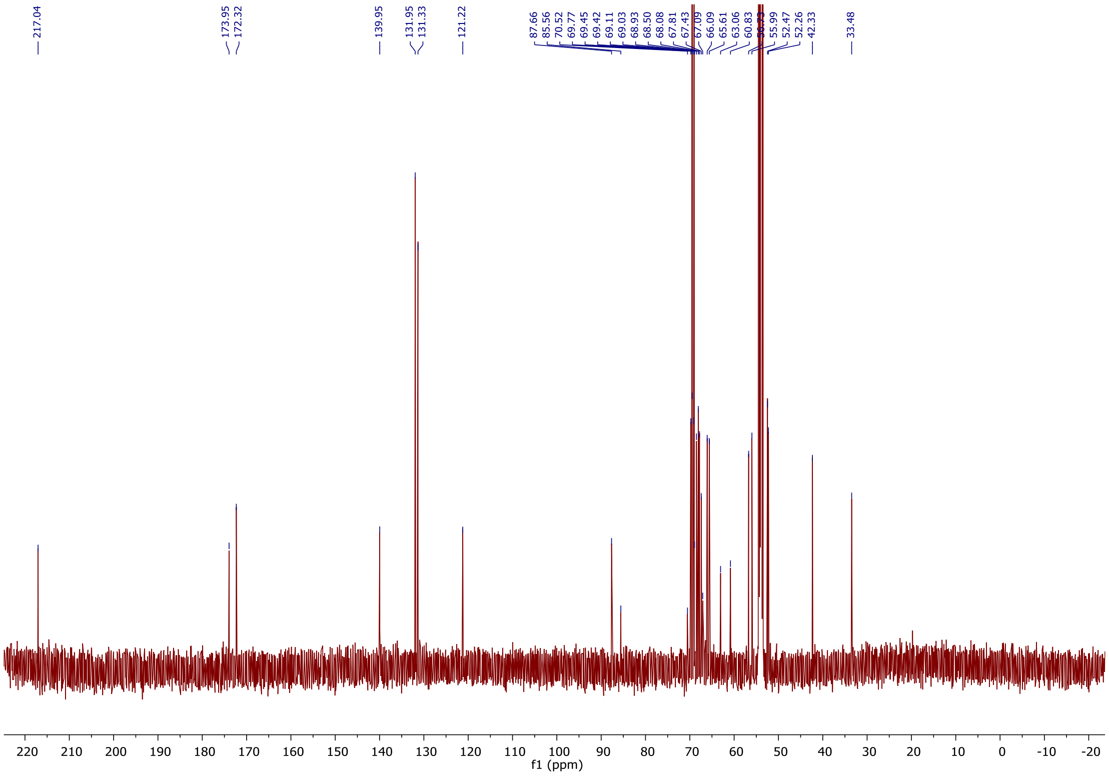

HPLC traces for **3y**: racemic top, enantiomer 1 bottom

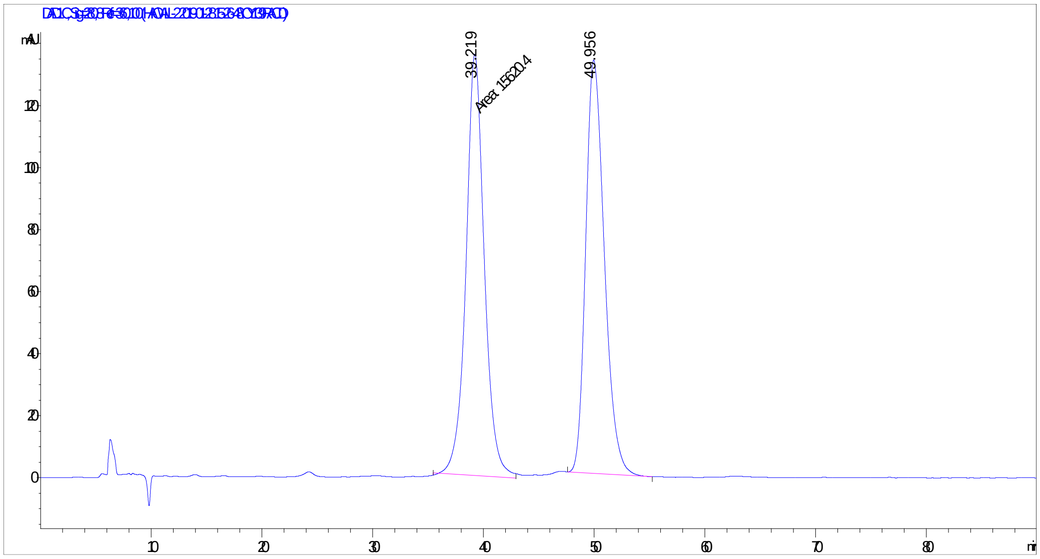

#	Time	Area	Height	Width	Area%	Symmetry
1	39.219	15620.4	135.8	1.9168	50.014	0.9
2	49.956	15611.7	133.2	1.8011	49.986	0.707

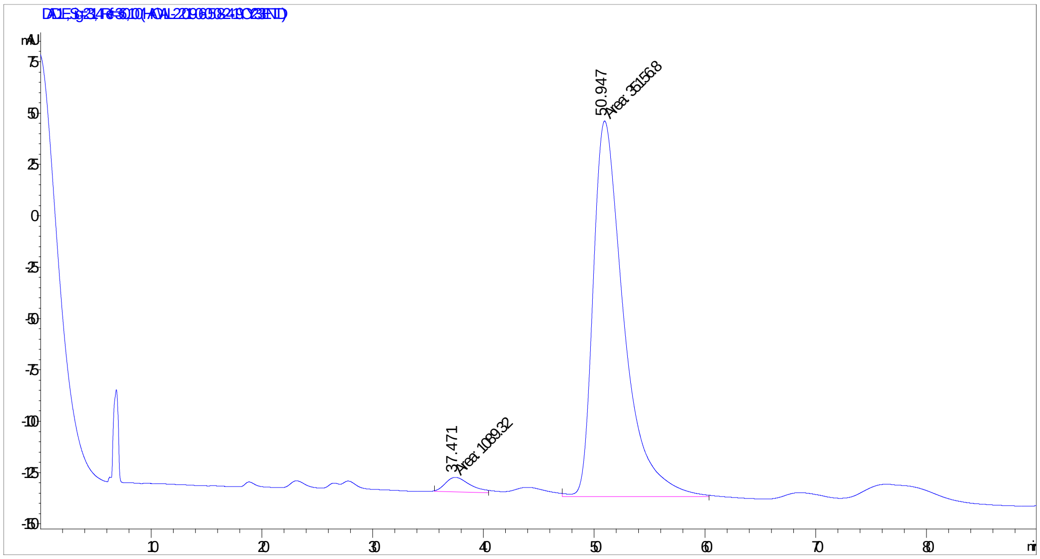

#	Time	Area	Height	Width	Area%	Symmetry
1	37.471	1089.3	7	2.5817	3.005	0.632
2	50.947	35156.8	182.8	3.2053	96.995	0.574

^{1}H NMR

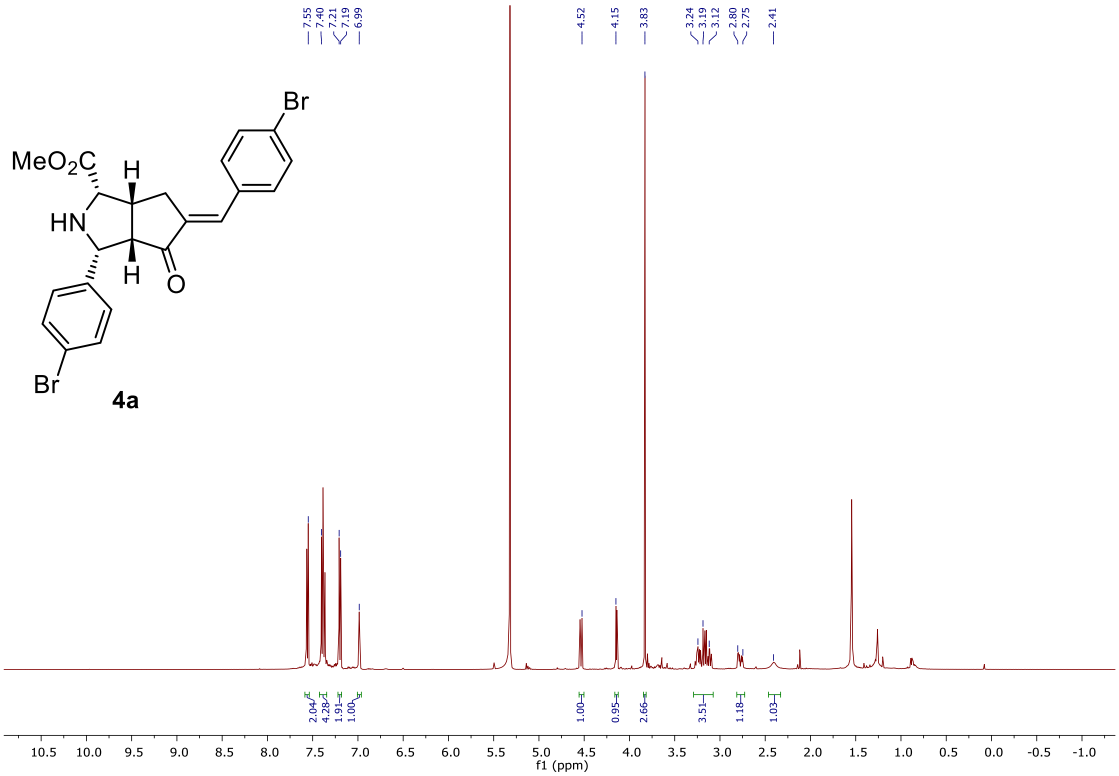

^{13}C NMR

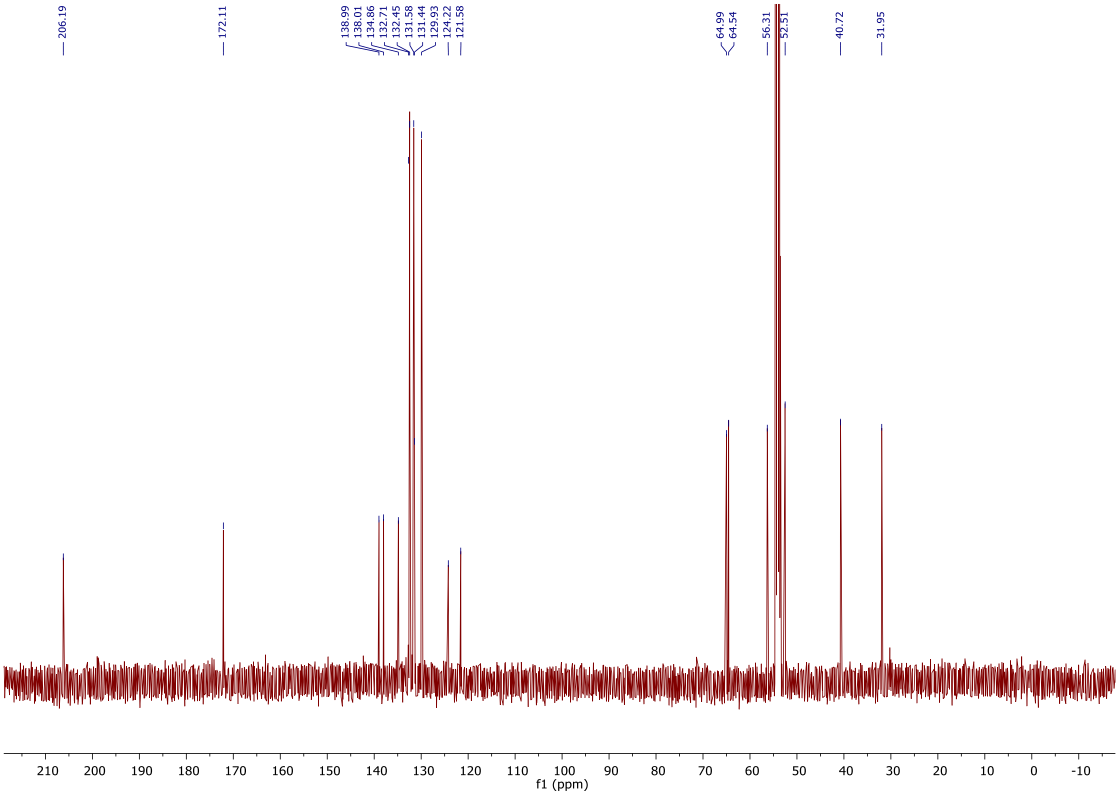

HPLC traces for **4a**: racemic top, enantiomer 1 bottom

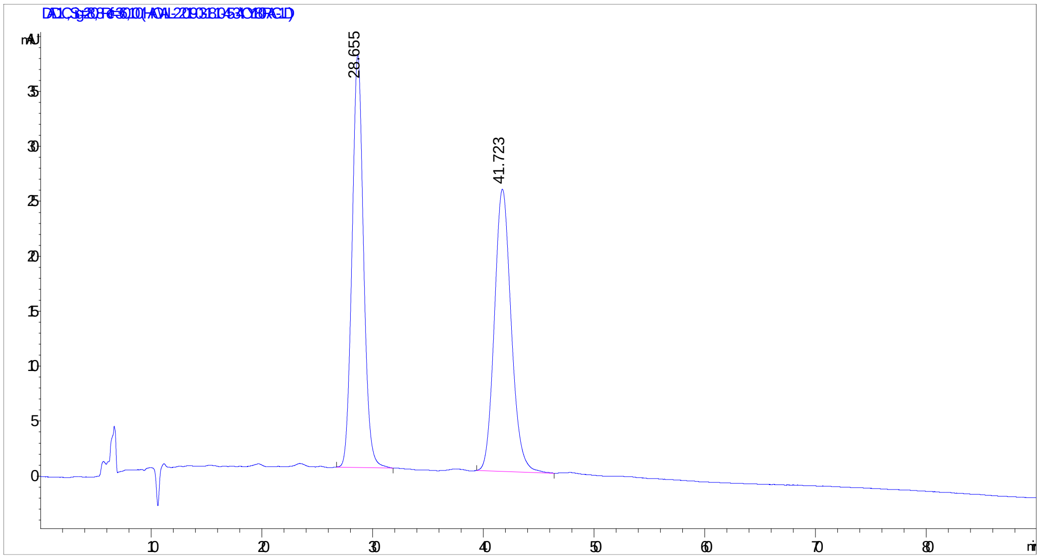

#	Time	Area	Height	Width	Area%	Symmetry
1	28.655	2699.8	37.6	1.0637	49.914	0.93
2	41.723	2709.2	25.7	1.5346	50.086	0.867

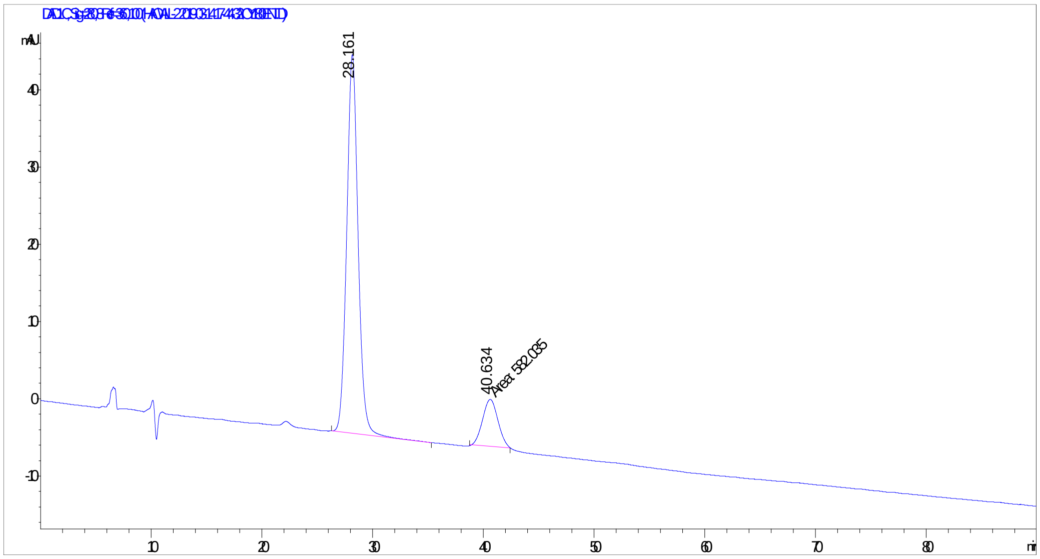

#	Time	Area	Height	Width	Area%	Symmetry
1	28.161	3503	49	1.0695	85.752	0.912
2	40.634	582	6.1	1.597	14.248	0.964

^{1}H NMR

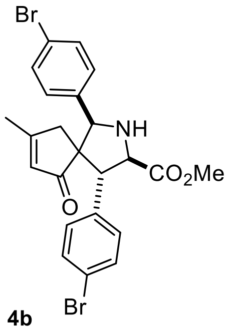

4b

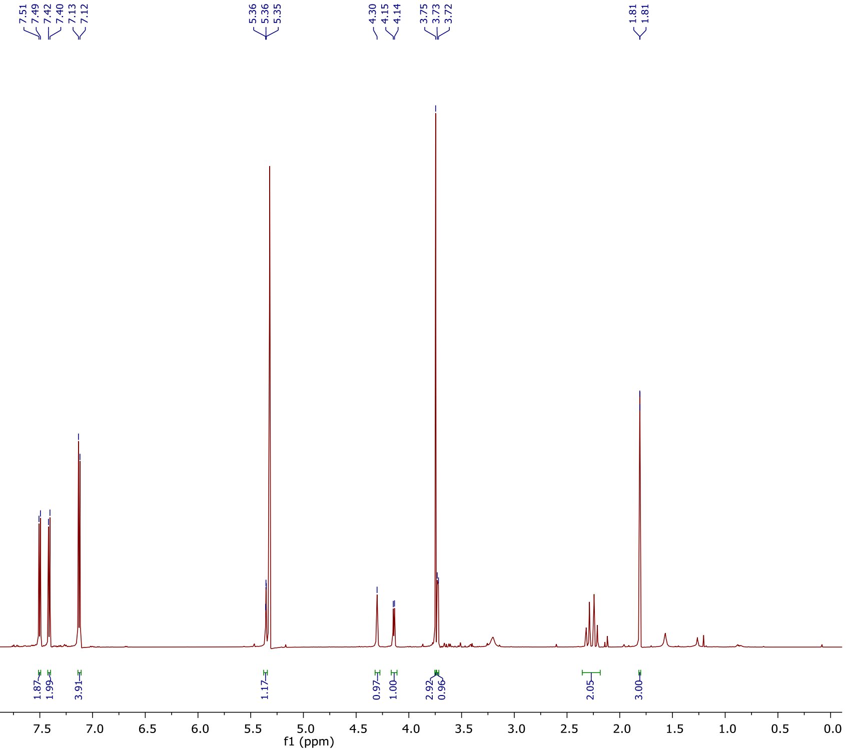

^{13}C NMR

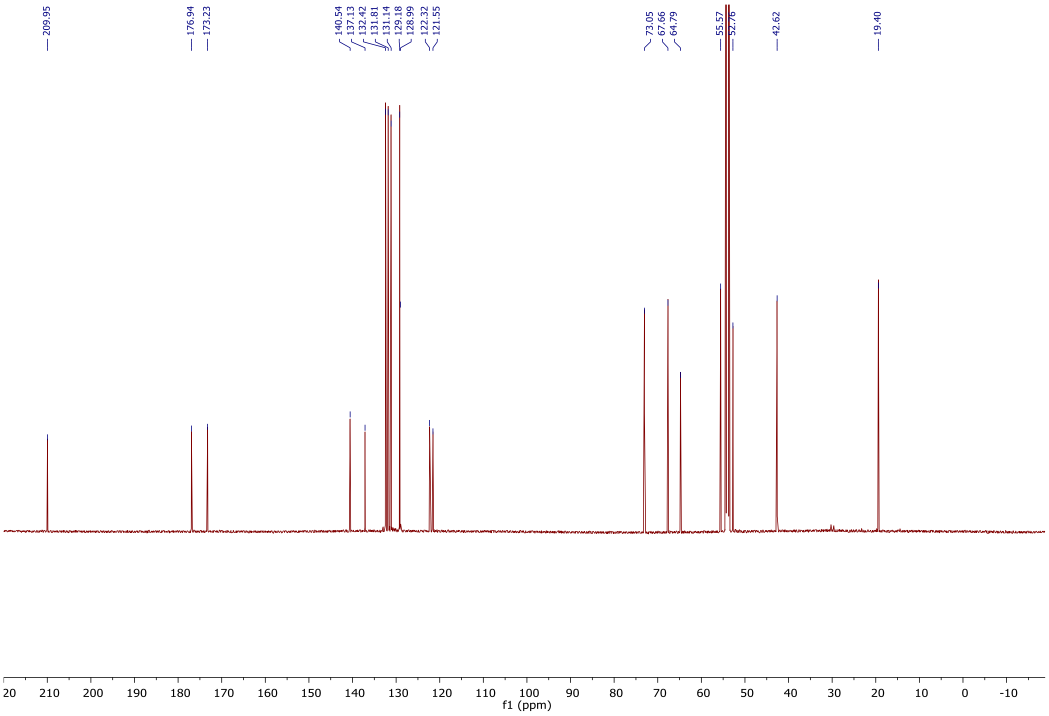

^{1}H NMR

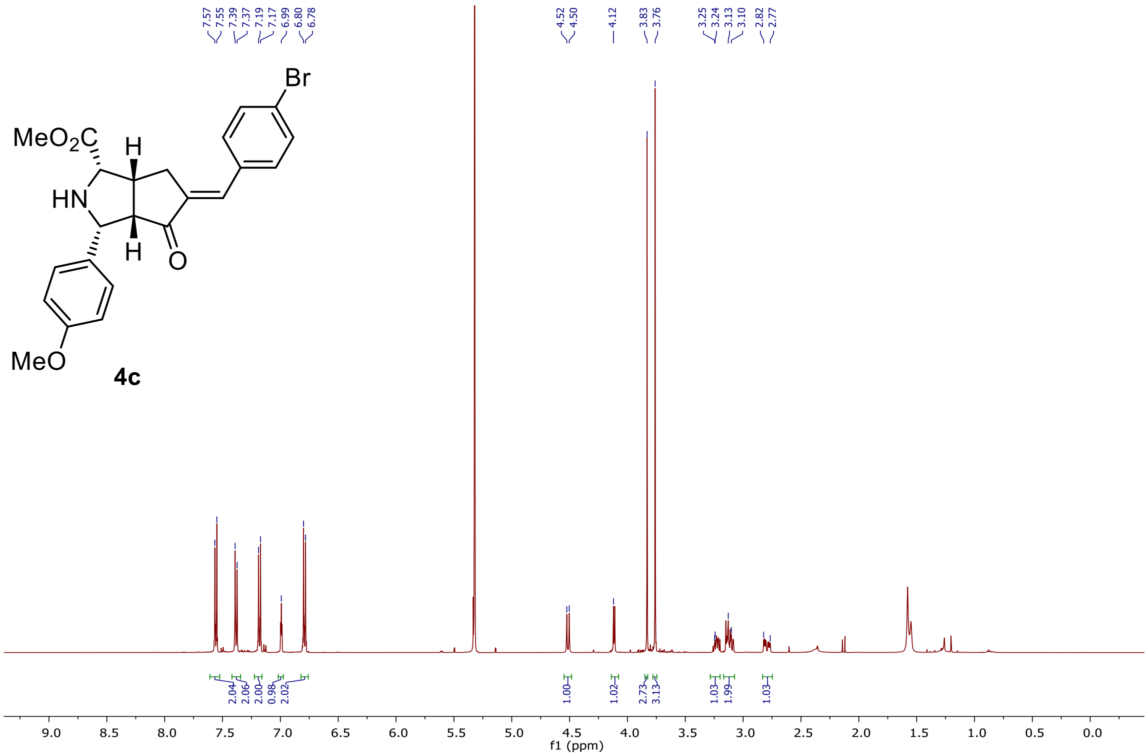

^{13}C NMR

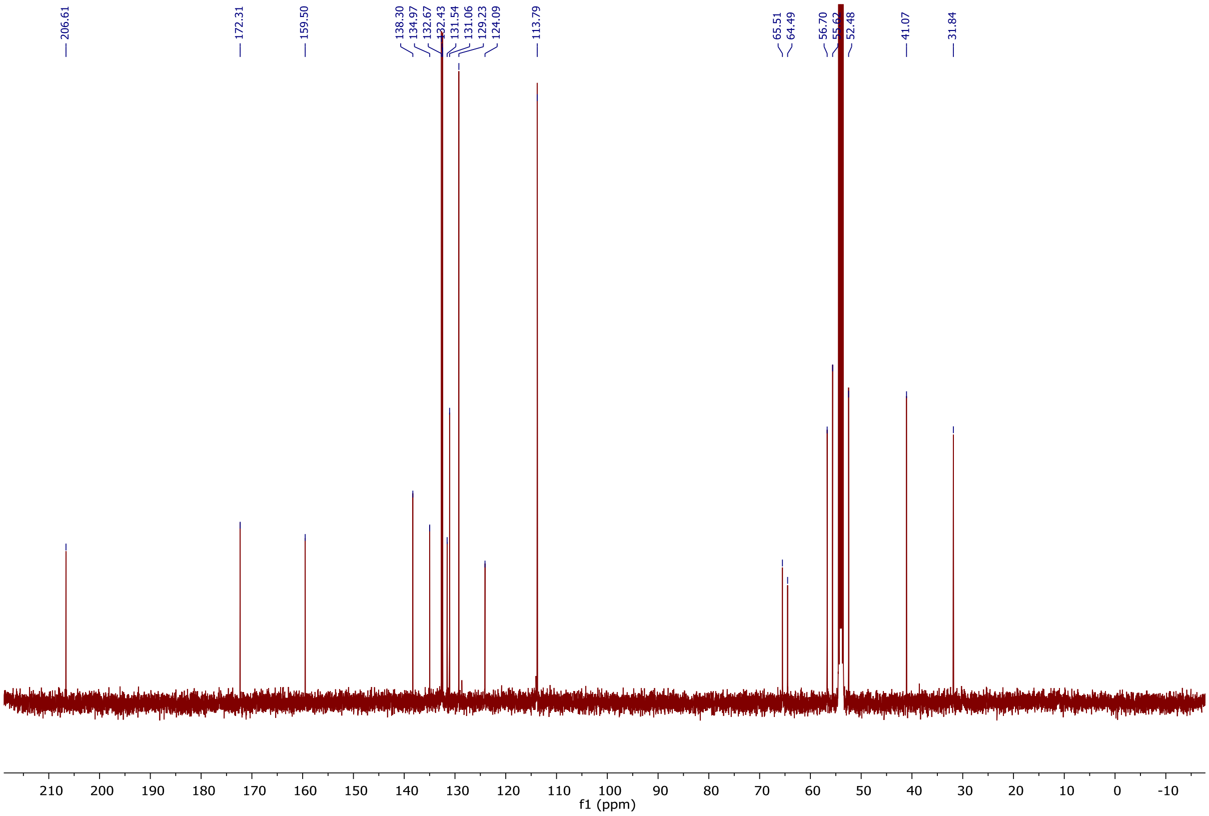

HPLC traces for **4c**: racemic top, enantiomer 1 bottom

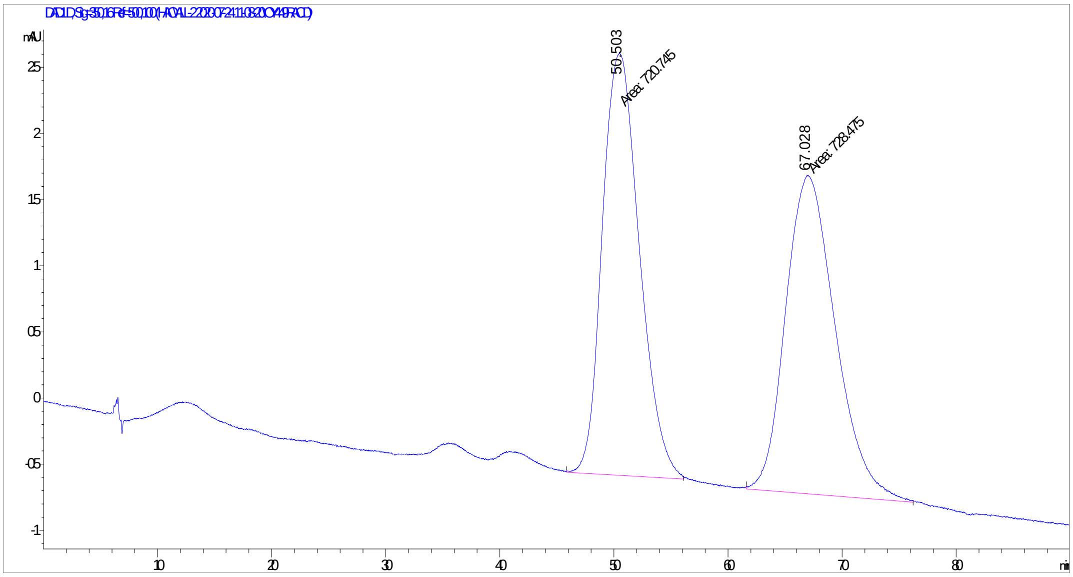

#	Time	Area	Height	Width	Area%	Symmetry
1	50.503	720.7	3.2	3.7647	49.733	0.839
2	67.028	728.5	2.4	5.0412	50.267	0.795

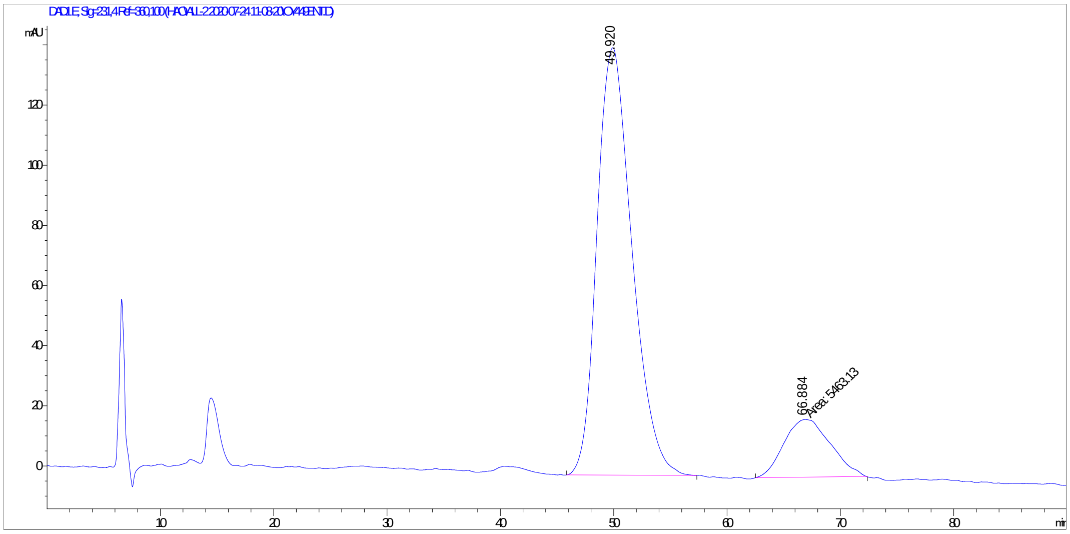

#	Time	Area	Height	Width	Area%	Symmetry
1	49.92	30572.2	142.1	2.6904	84.840	0.798
2	66.884	5463.1	19.2	4.7532	15.160	0.814

^{1}H NMR

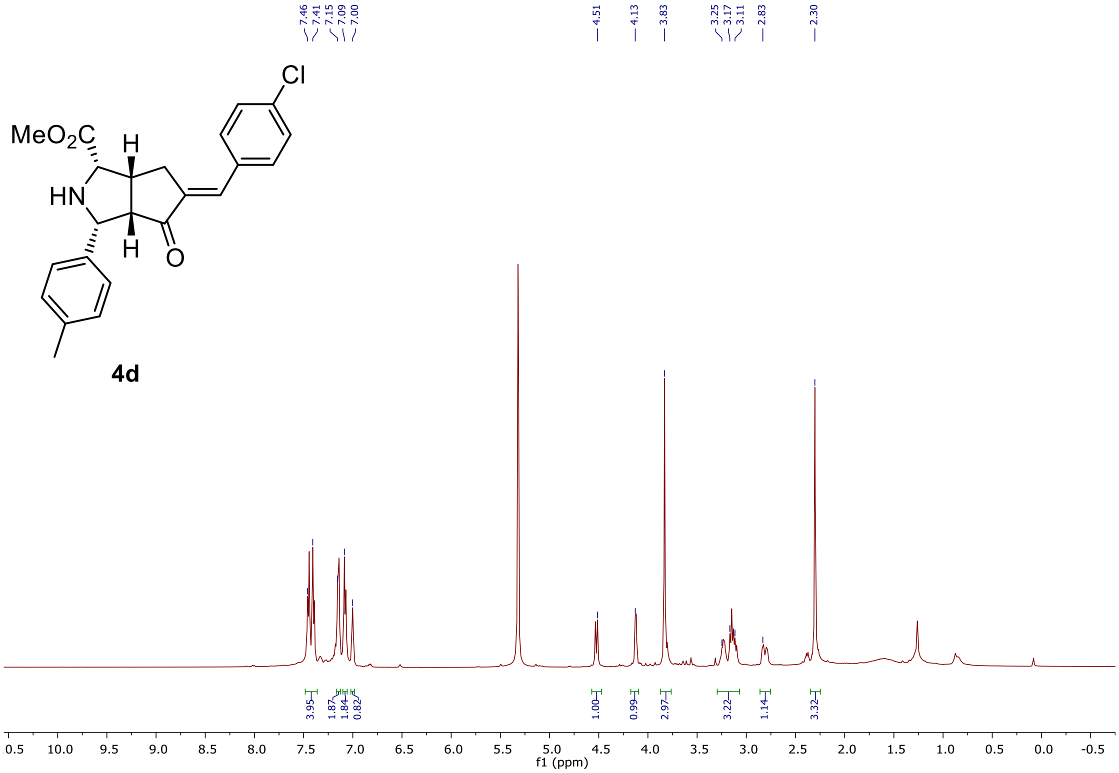

^{13}C NMR

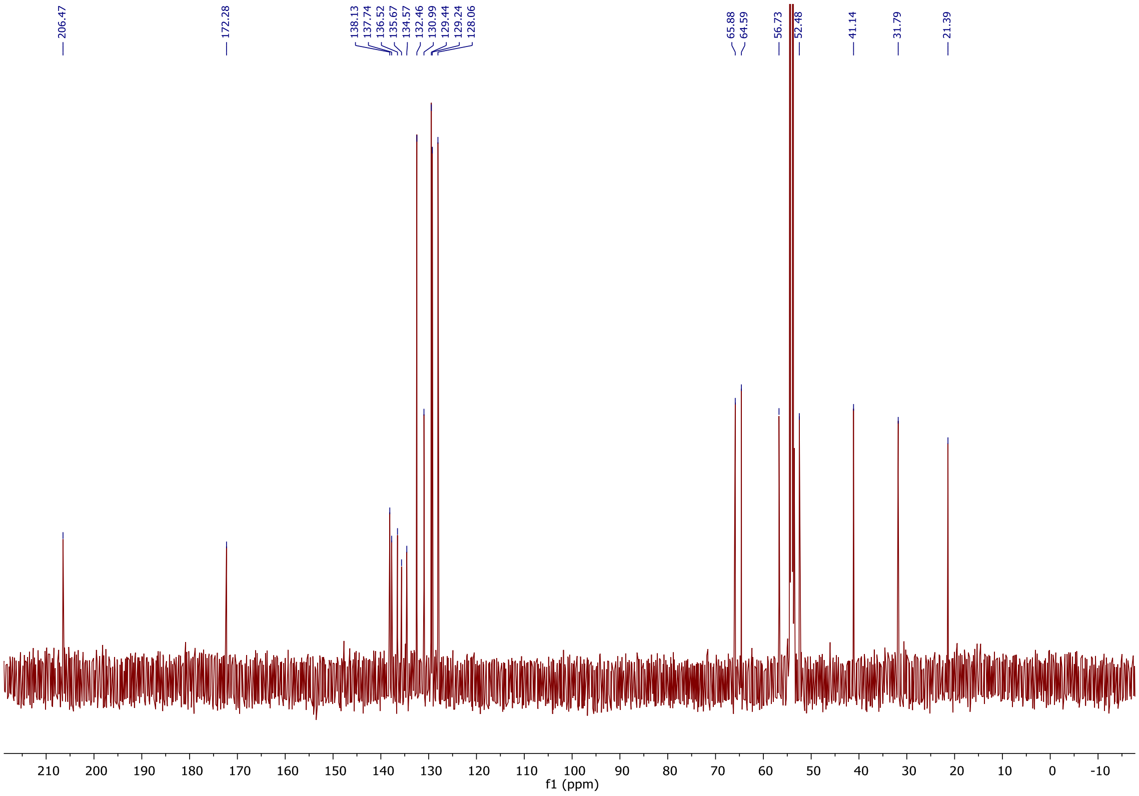

HPLC traces for **4d**: racemic top, enantiomer 1 bottom

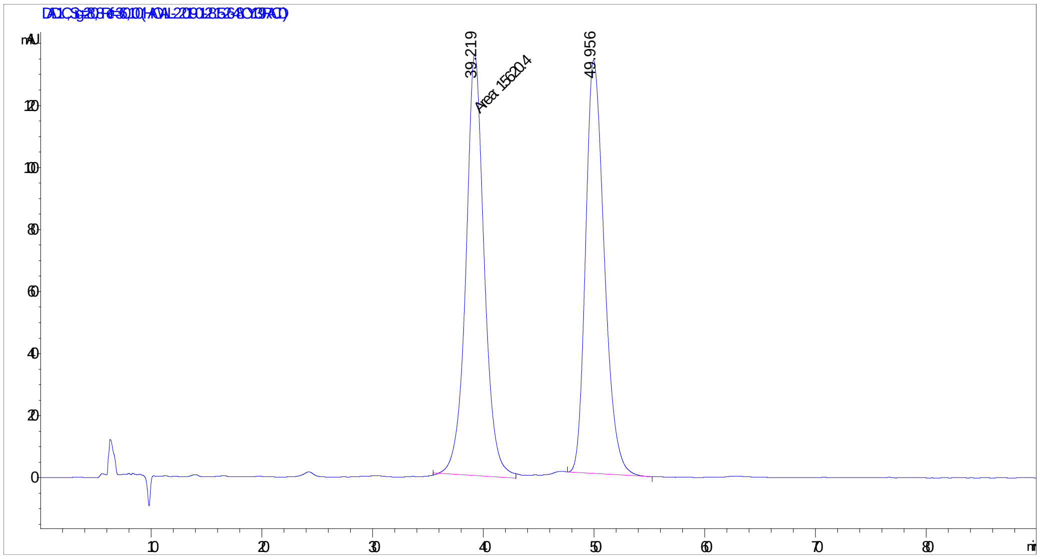

#	Time	Area	Height	Width	Area%	Symmetry
1	39.219	15620.4	135.8	1.9168	50.014	0.9
2	49.956	15611.7	133.2	1.8011	49.986	0.707

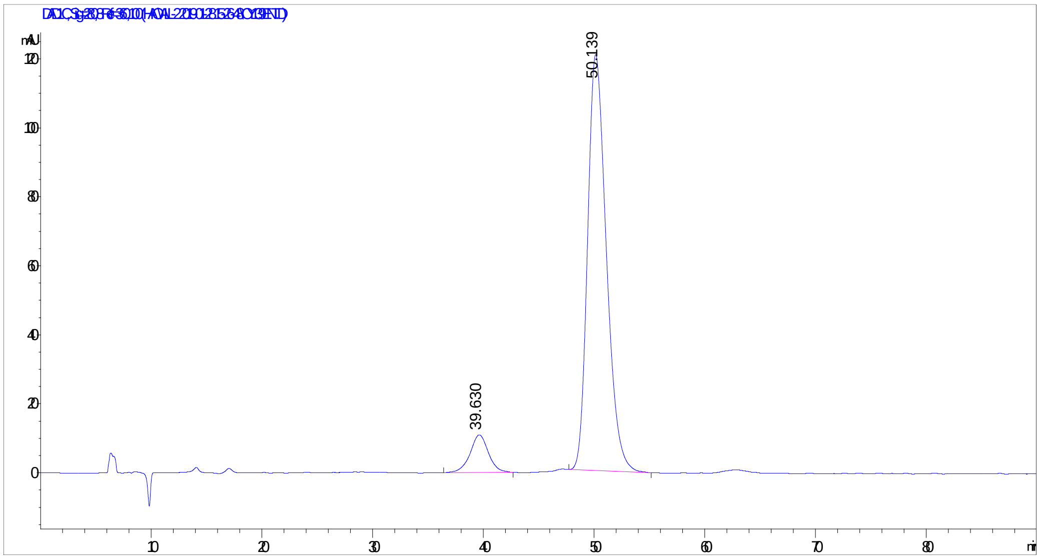

#	Time	Area	Height	Width	Area%	Symmetry
1	39.63	1260	10.9	1.5658	8.162	1
2	50.139	14176.5	120.6	1.7513	91.838	0.715

^{1}H NMR

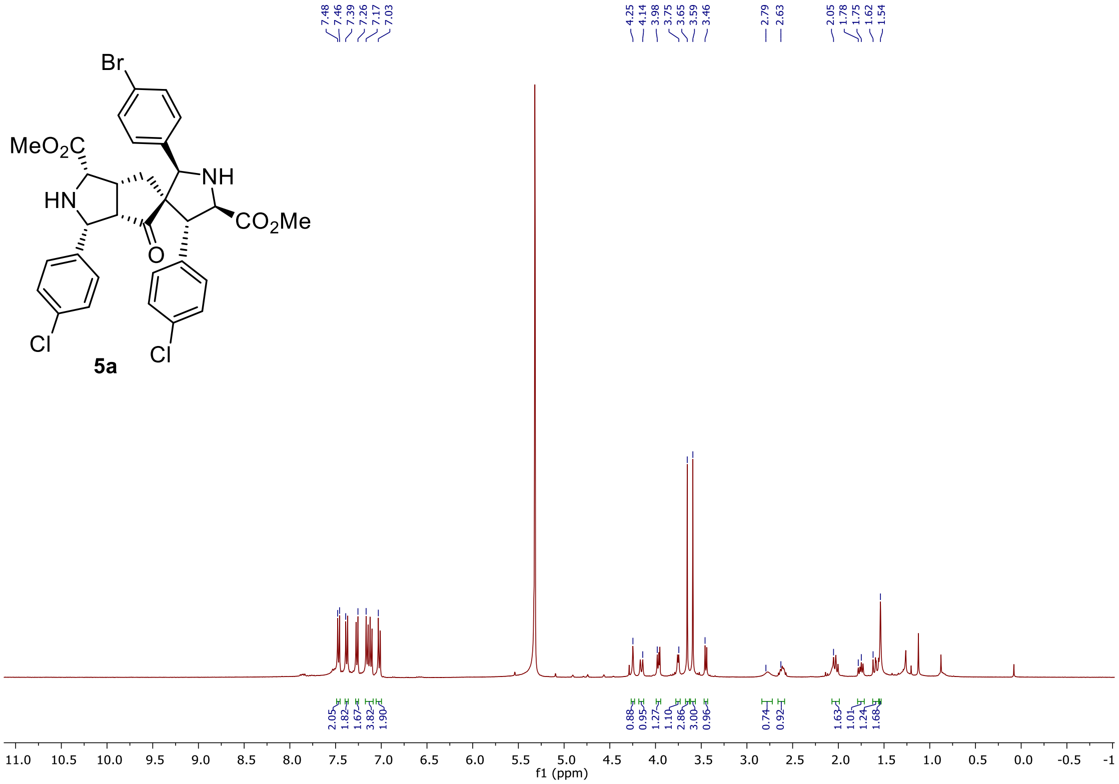

5a

^{13}C NMR

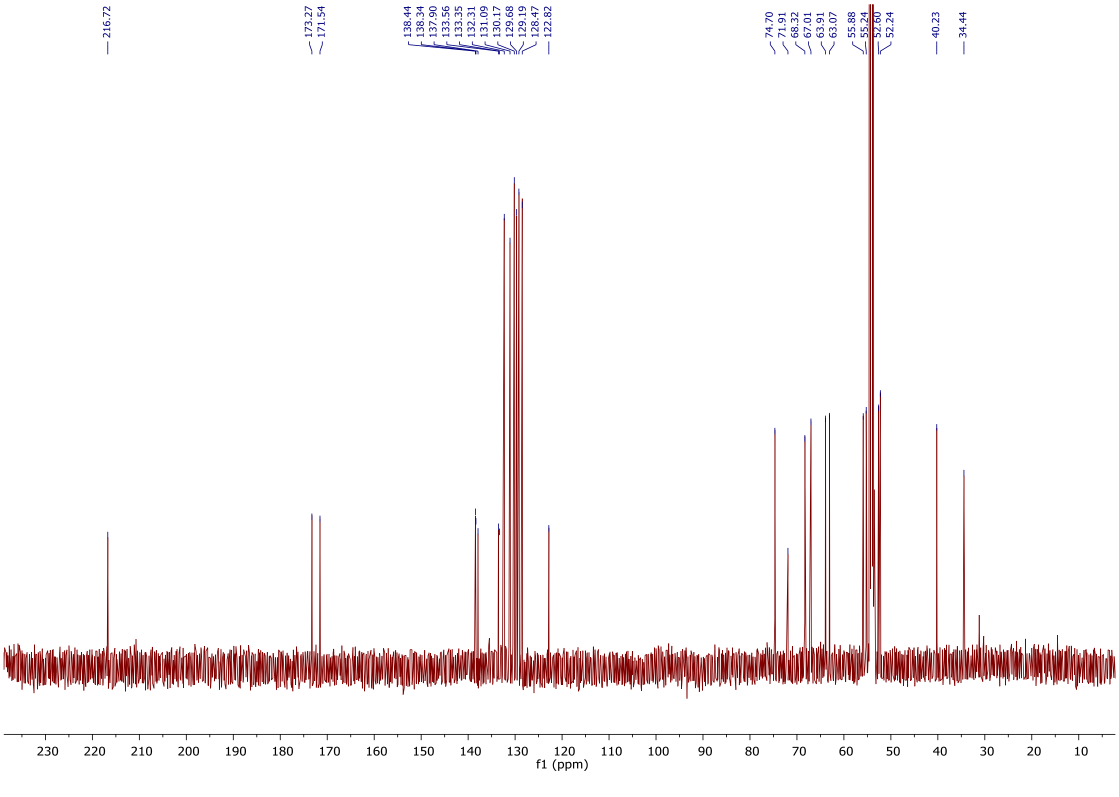

HPLC traces for **5a**: racemic top, enantiomer 1 bottom

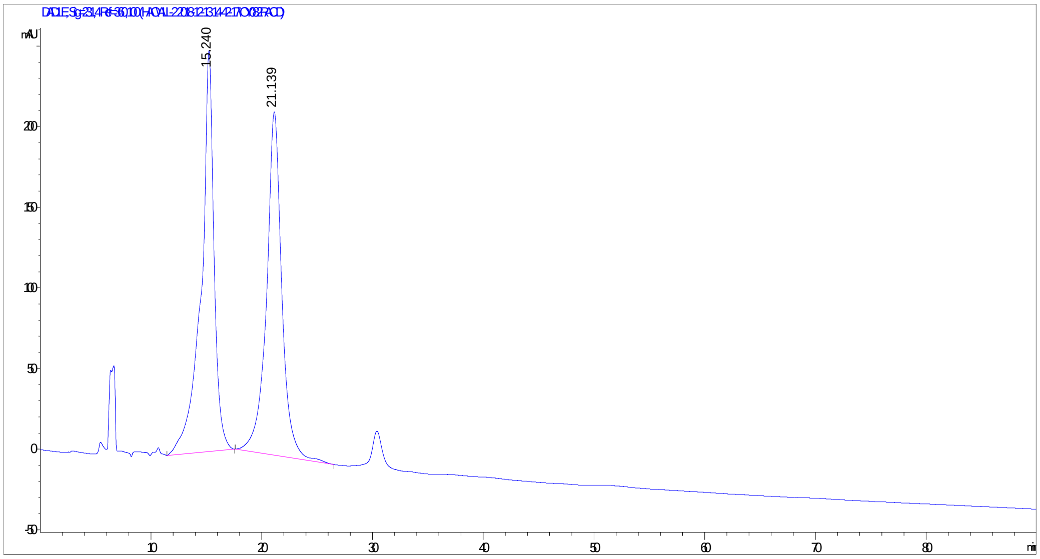

#	Time	Area	Height	Width	Area%	Symmetry
1	15.24	20157.6	248.7	1.1156	49.582	1.57
2	21.139	20497.7	212.9	1.3813	50.418	1.013

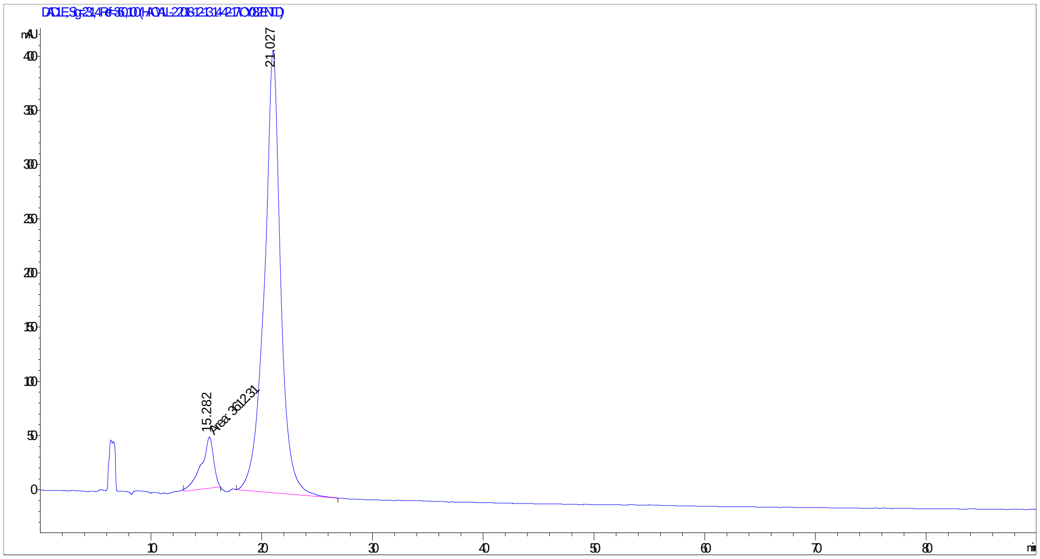

#	Time	Area	Height	Width	Area%	Symmetry
1	15.289	3346.5	47.1	1.1842	7.422	1.514
2	21.027	41743.9	407.8	1.4483	92.578	1.107

^{1}H NMR

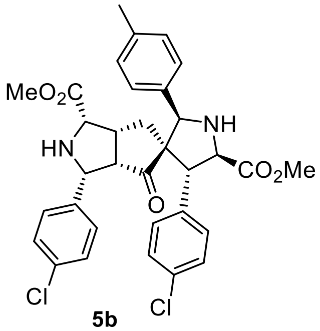

5b

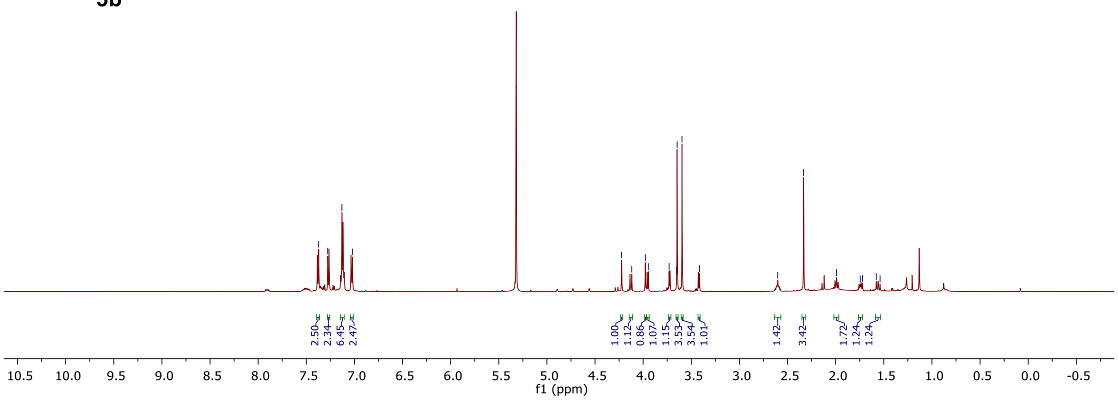

^{13}C NMR

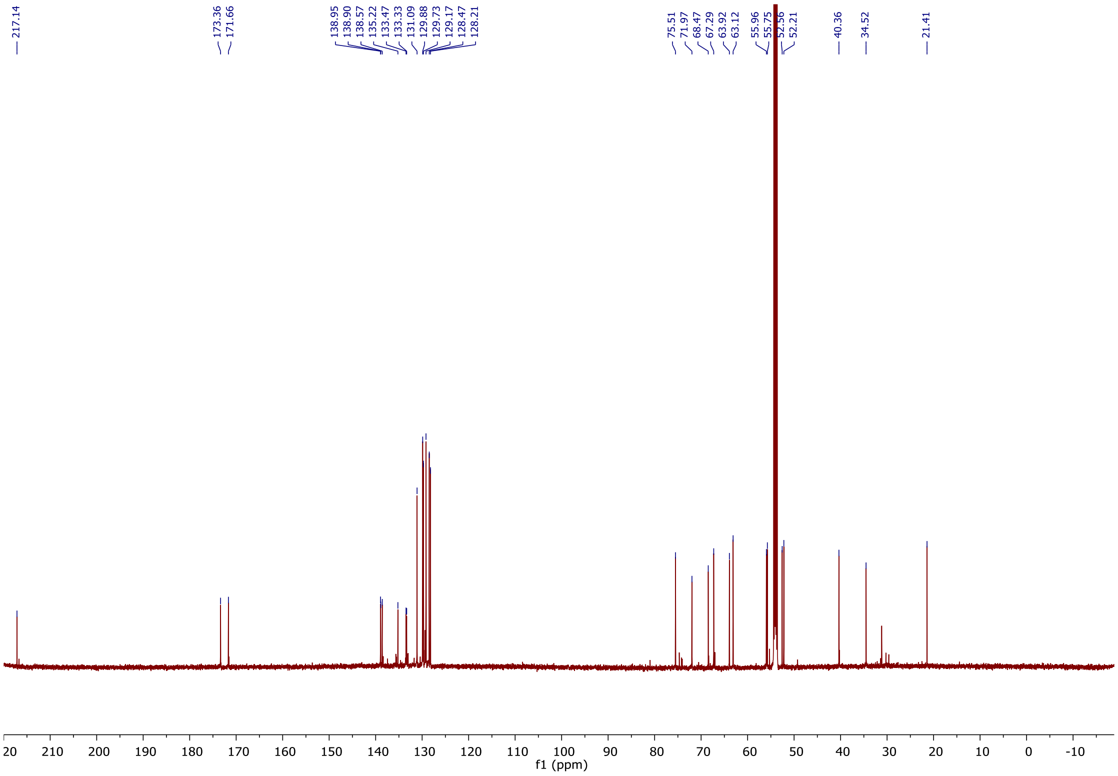

HPLC traces for **5b**: racemic top, enantiomer 1 bottom

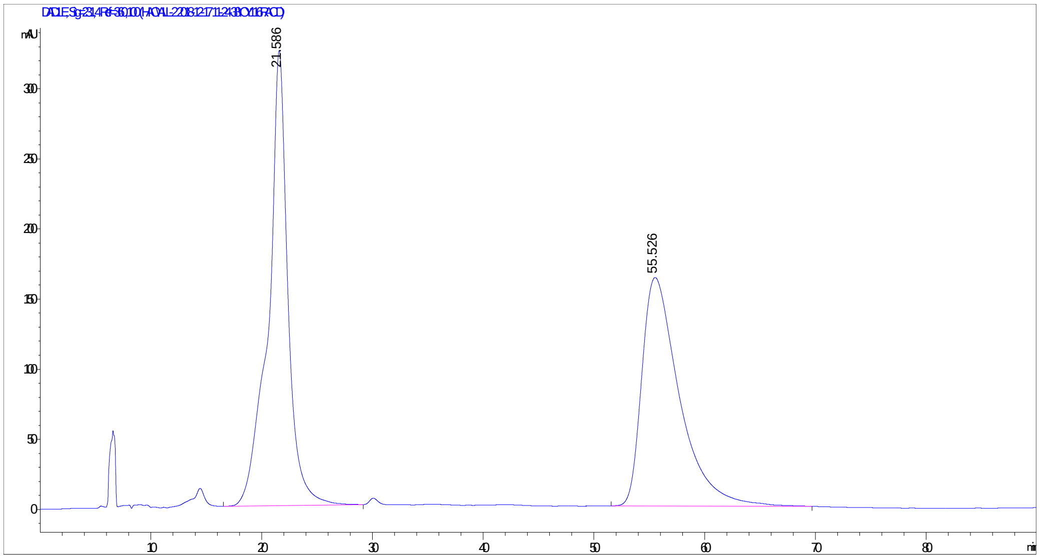

#	Time	Area	Height	Width	Area%	Symmetry
1	21.586	39707.2	324.7	1.7026	50.791	1.222
2	55.526	38471	163	3.1509	49.209	0.503

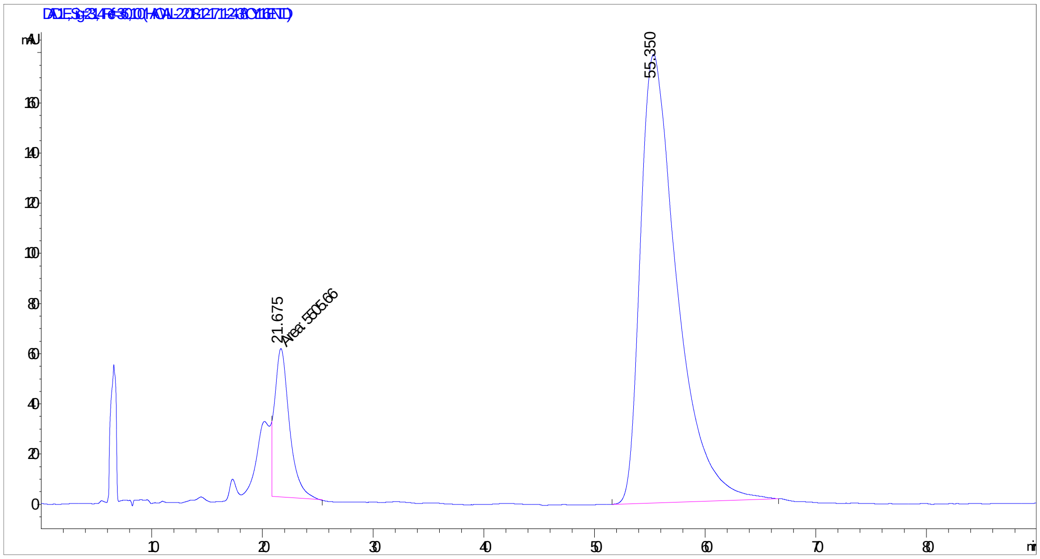

#	Time	Area	Height	Width	Area%	Symmetry
1	21.671	4670.4	60.2	1.2931	9.856	0.368
2	55.35	42713.9	179.7	3.9618	90.144	0.52

^{1}H NMR

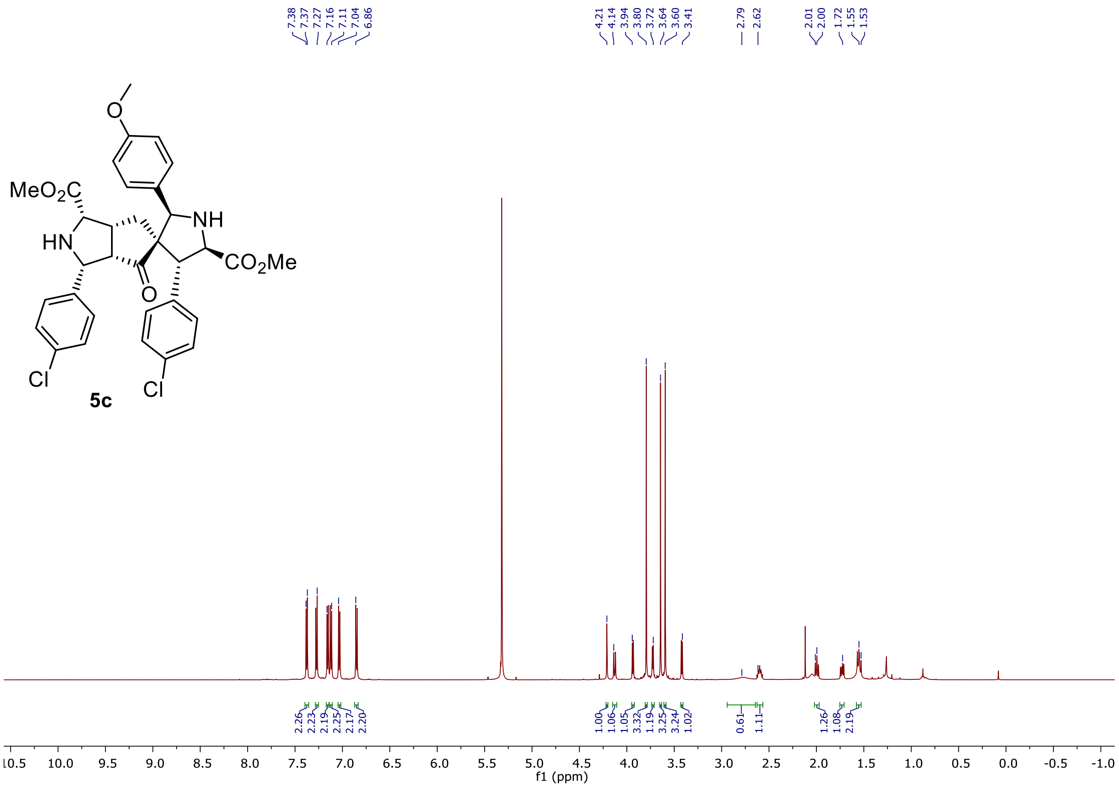

^{13}C HMR

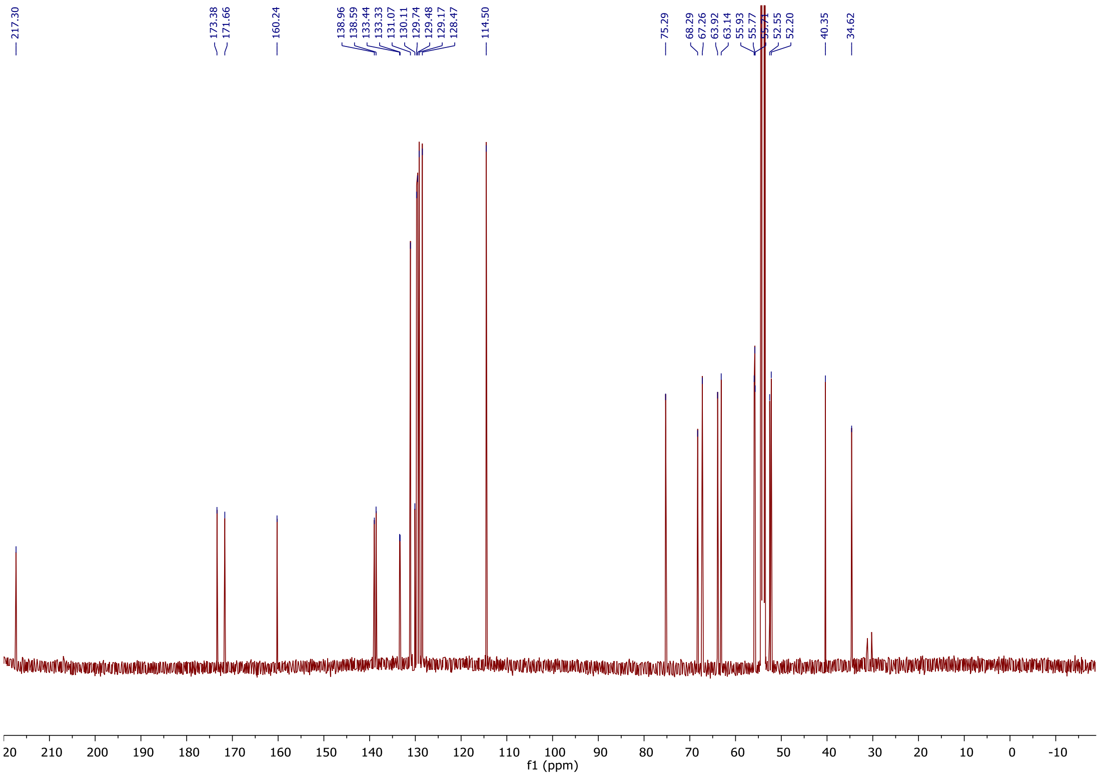

HPLC traces for **5c**: racemic top, enantiomer 1 bottom

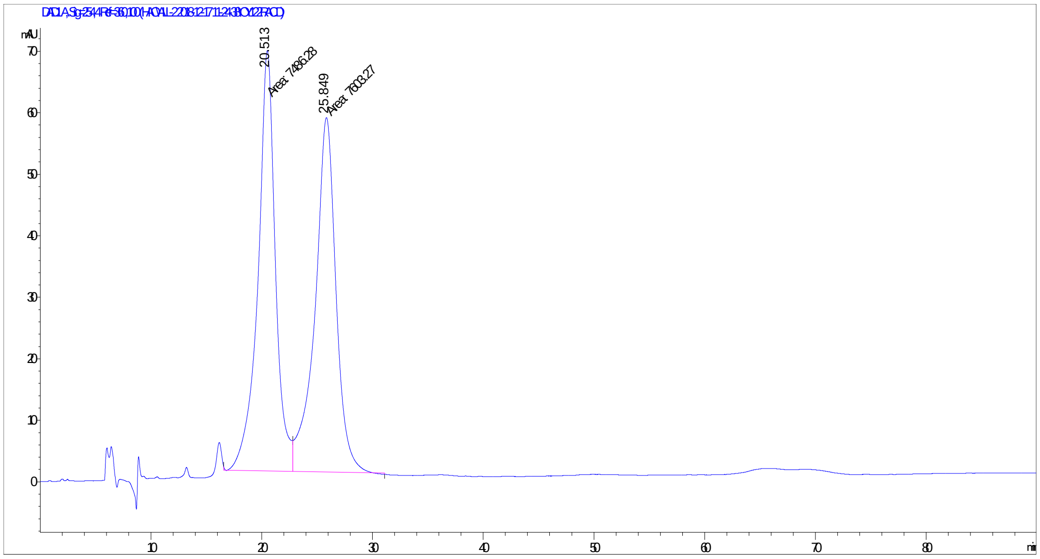

#	Time	Area	Height	Width	Area%	Symmetry
1	20.513	7486.3	68.3	1.8263	49.612	0
2	25.849	7603.3	57.6	2.2	50.388	1.162

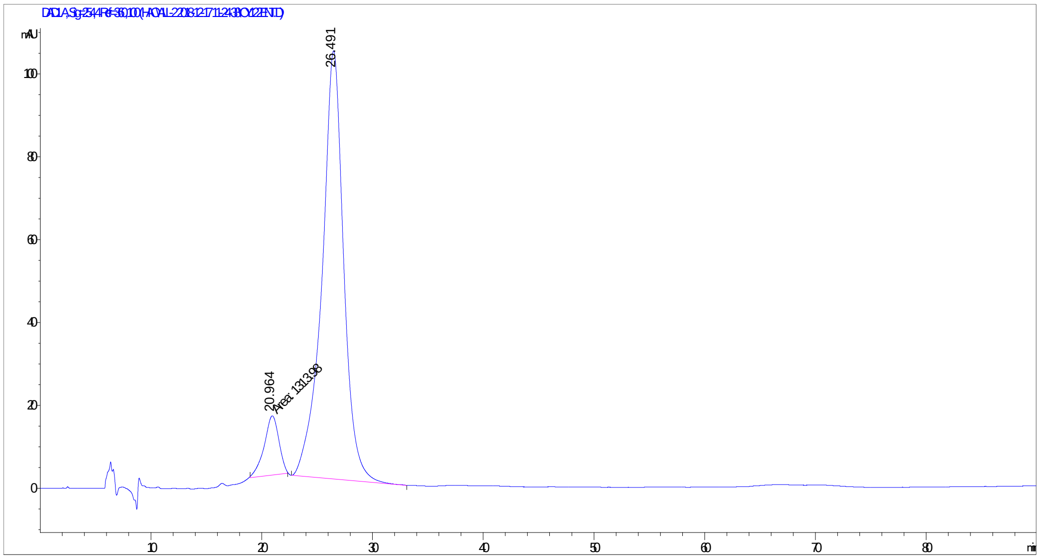

#	Time	Area	Height	Width	Area%	Symmetry
1	20.964	1314	14.3	1.5344	8.551	1.235
2	26.491	14053.2	103.3	1.9589	91.449	1.123

^{1}H NMR

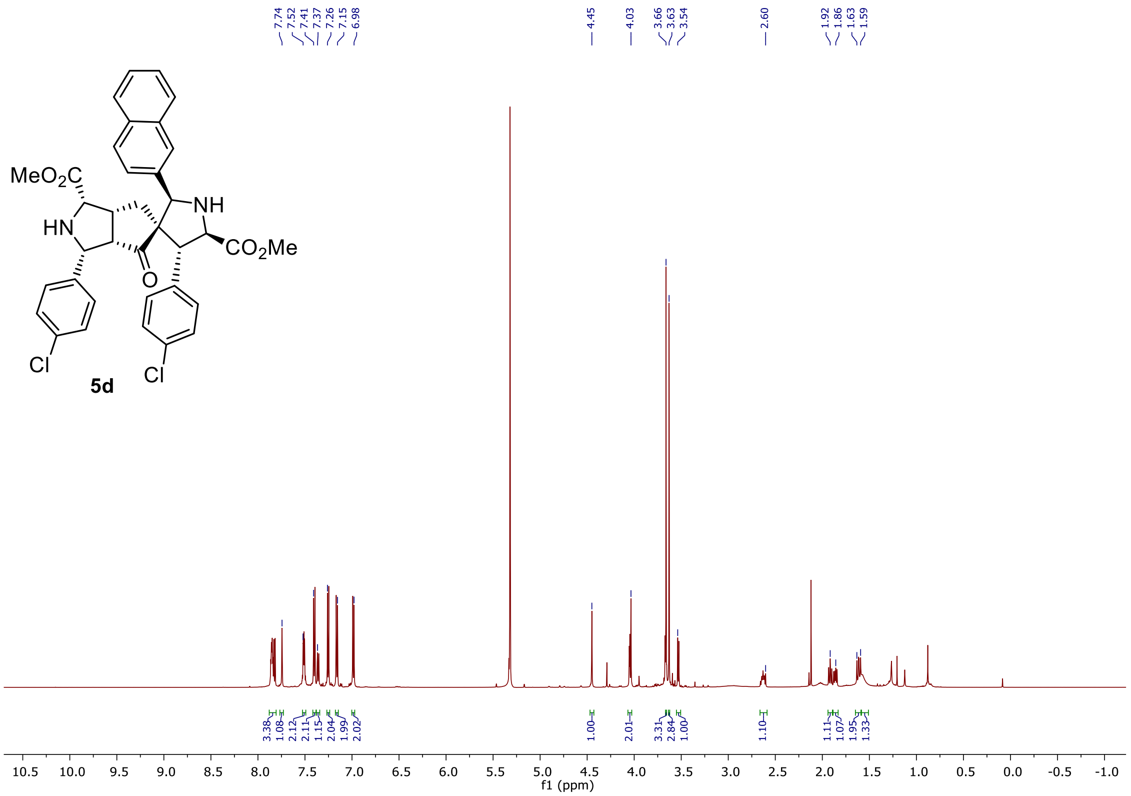

^{13}C NMR

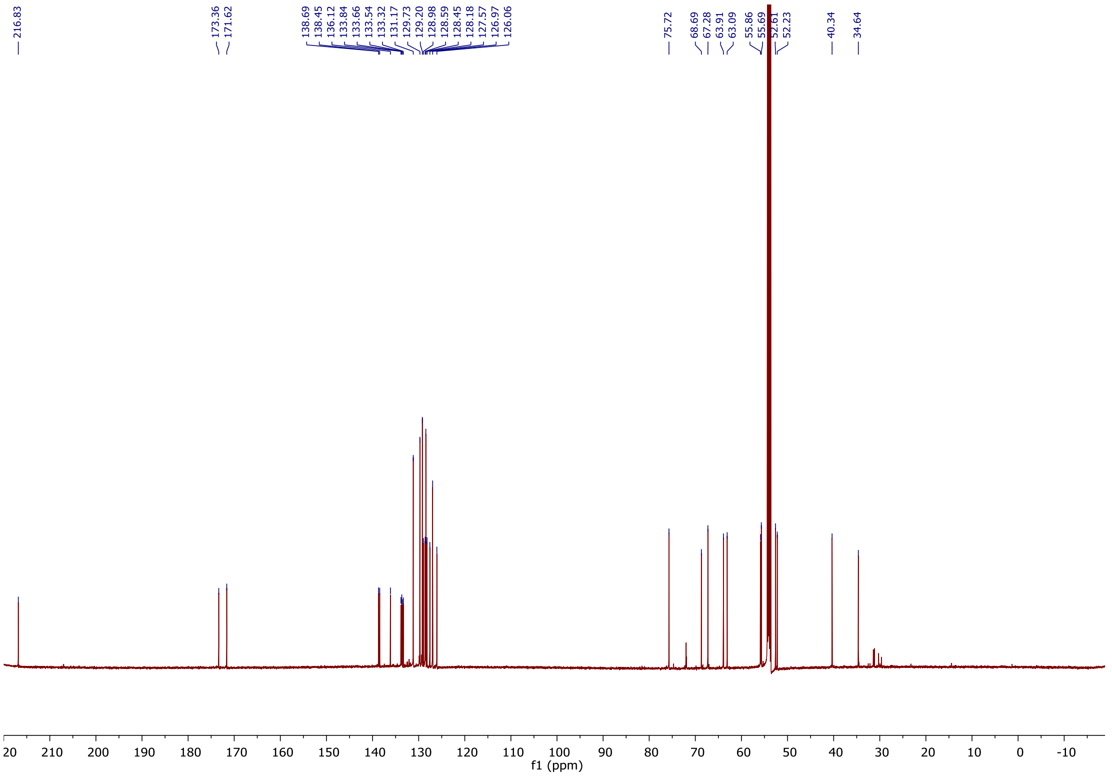

HPLC traces for **5d**: racemic top, enantiomer 1 bottom

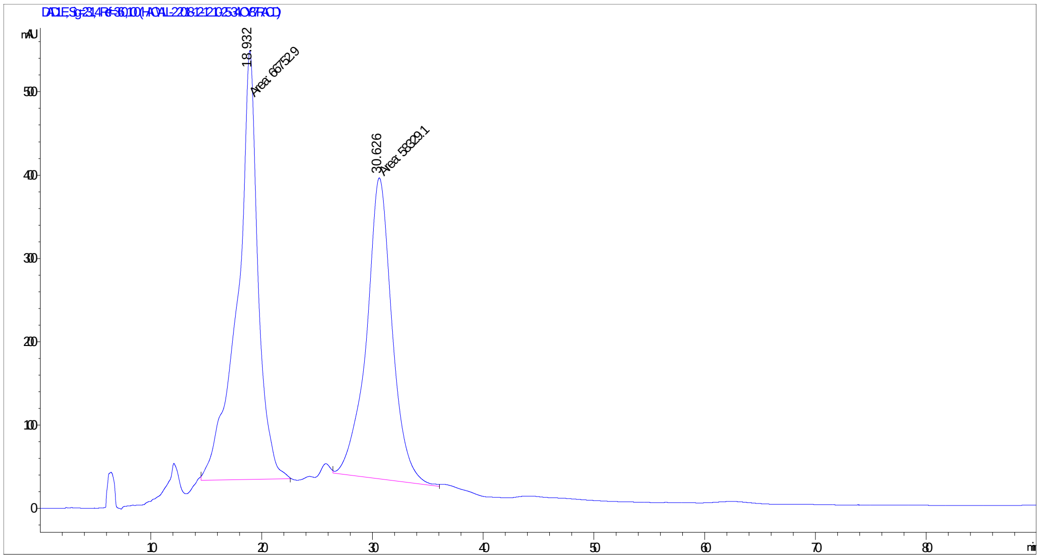

#	Time	Area	Height	Width	Area%	Symmetry
1	18.932	66752.9	514.7	2.1615	53.367	1.433
2	30.626	58329.1	361.1	2.6925	46.633	0.98

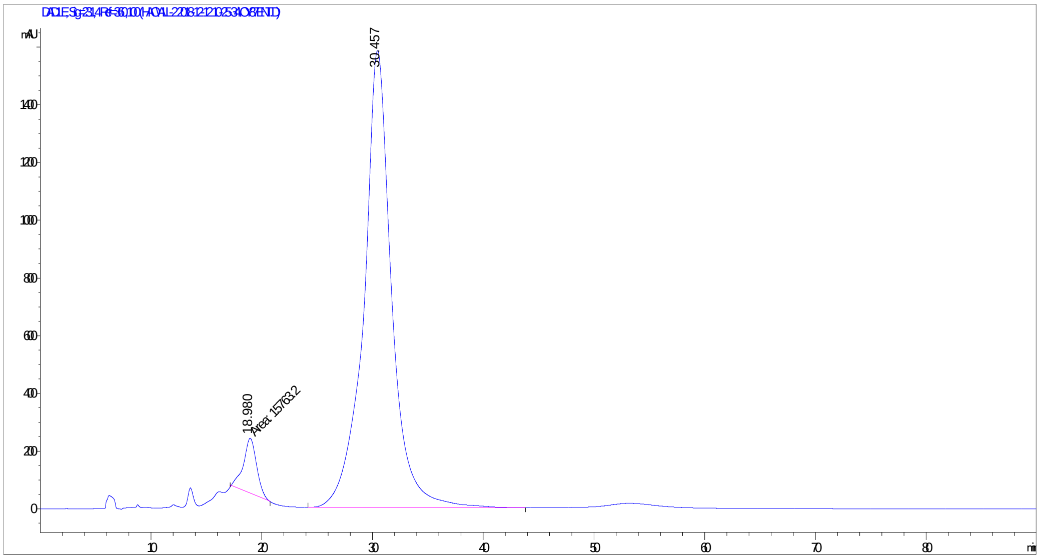

#	Time	Area	Height	Width	Area%	Symmetry
1	18.98	15763.2	190.1	1.3823	5.396	1.019
2	30.457	276356.2	1582.8	2.431	94.604	0.941

^{1}H NMR

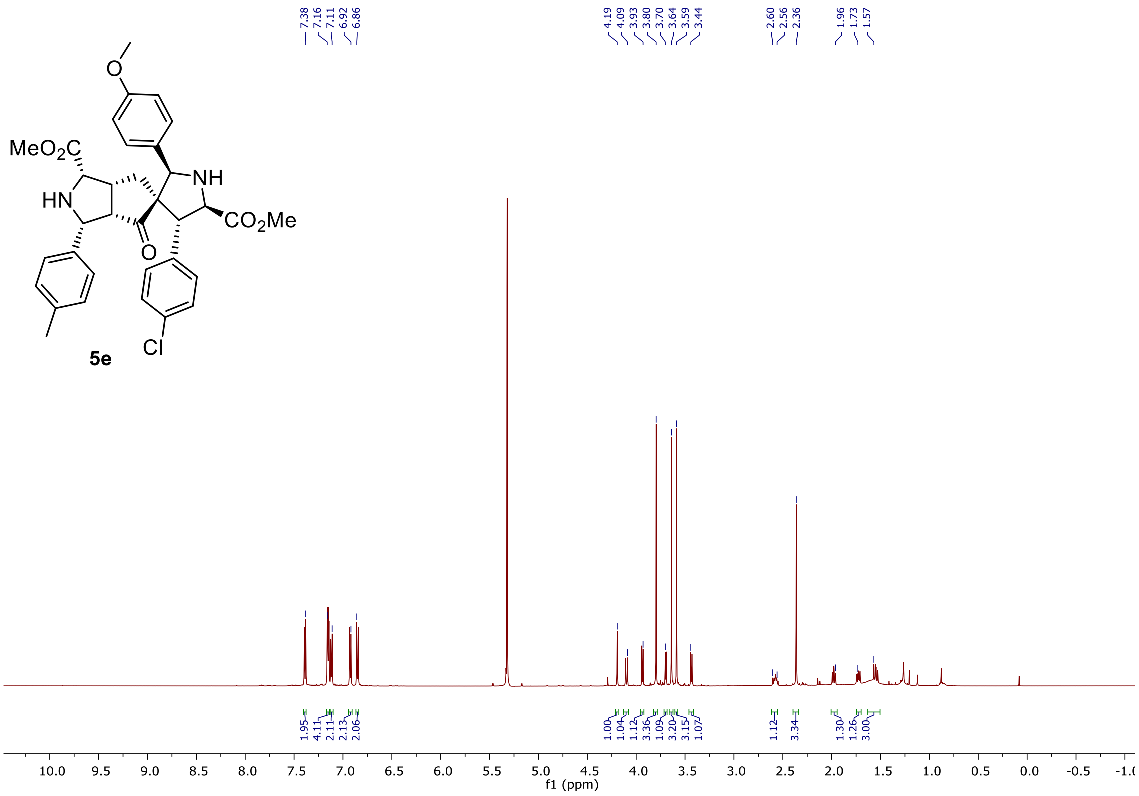

^{13}C NMR

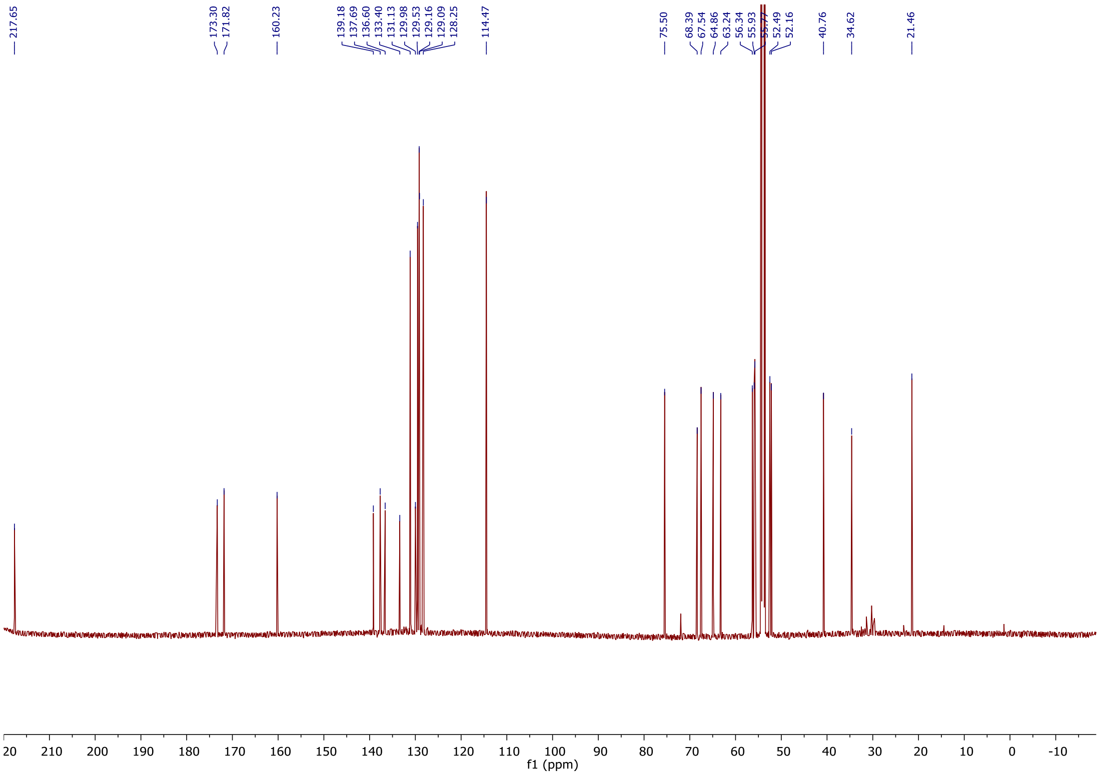

HPLC traces for **5e**: racemic top, enantiomer 1 bottom

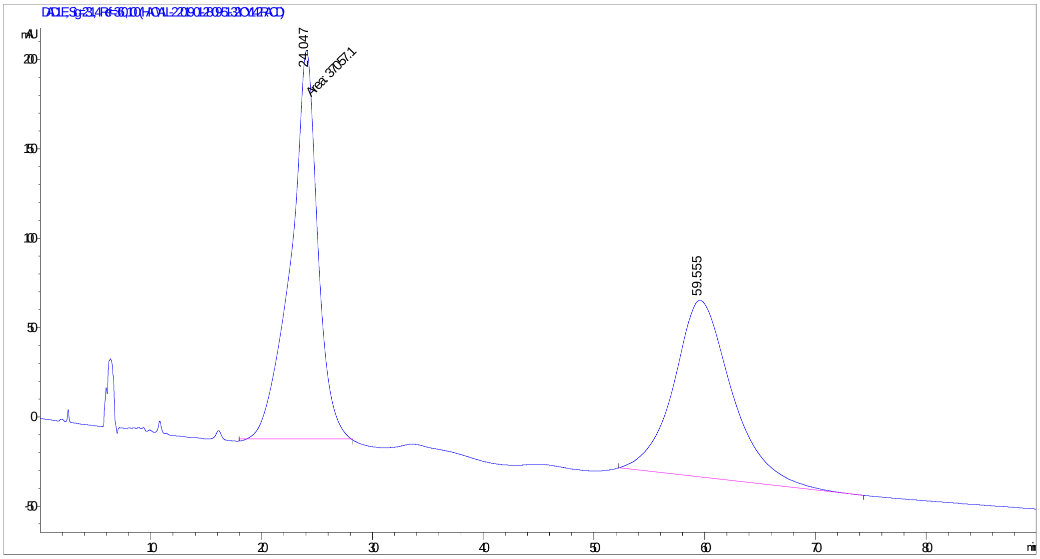

#	Time	Area	Height	Width	Area%	Symmetry
1	24.047	37057.1	217.4	2.8412	50.575	1.359
2	59.555	36215.1	98.9	4.3174	49.425	0.779

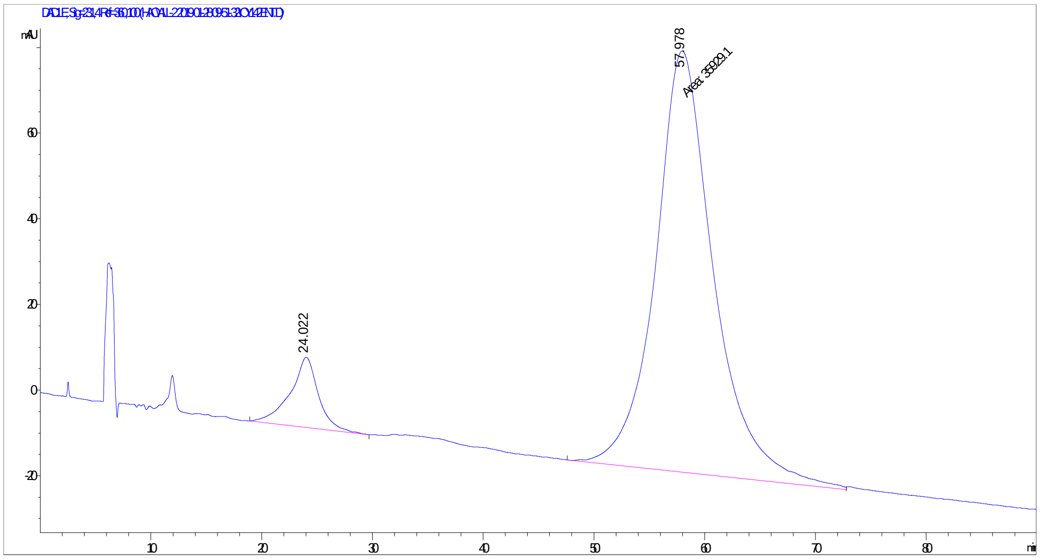

#	Time	Area	Height	Width	Area%	Symmetry
1	24.022	2889.6	16.4	2.1611	7.444	1.274
2	57.978	35929.1	98.4	6.0881	92.556	0.822

^{1}H NMR

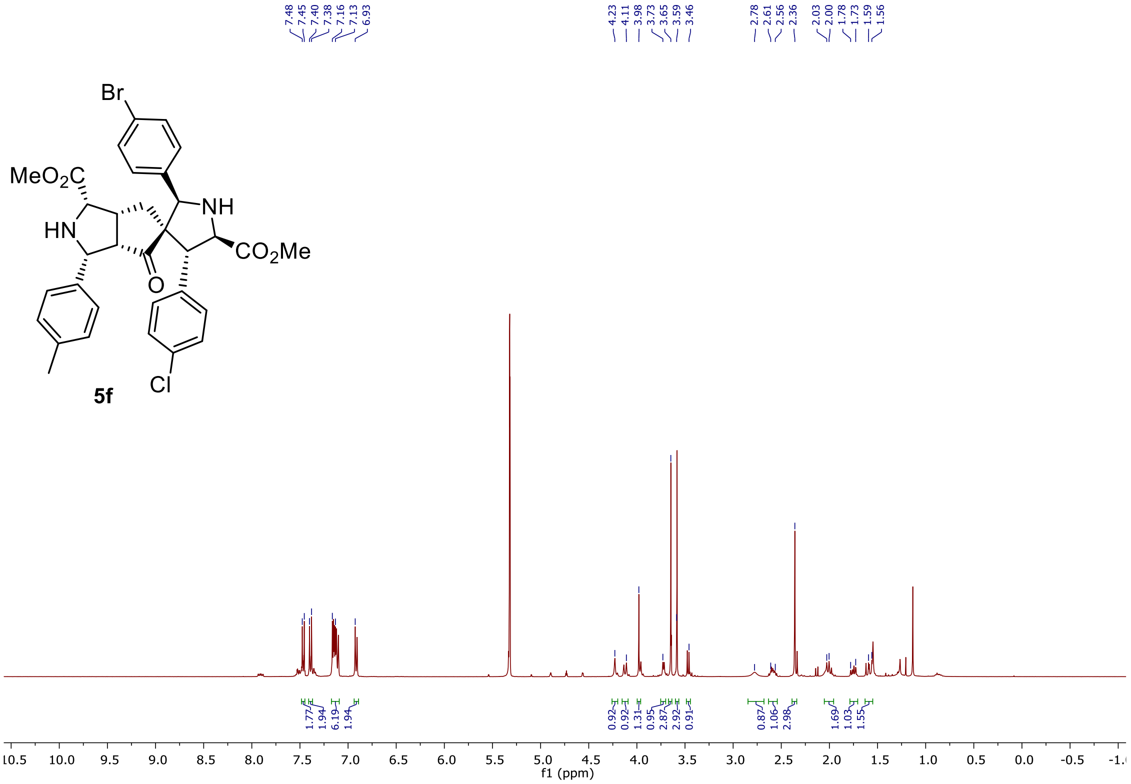

^{13}C NMR

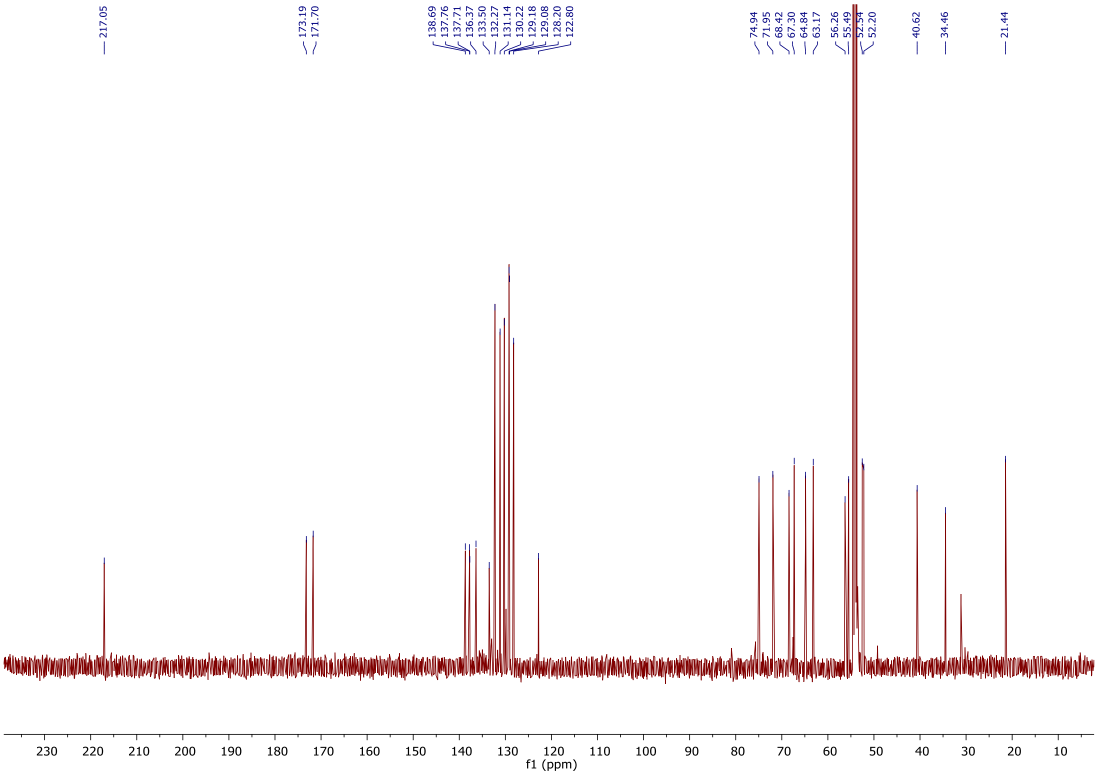

HPLC traces for **5f**: racemic top, enantiomer 1 bottom

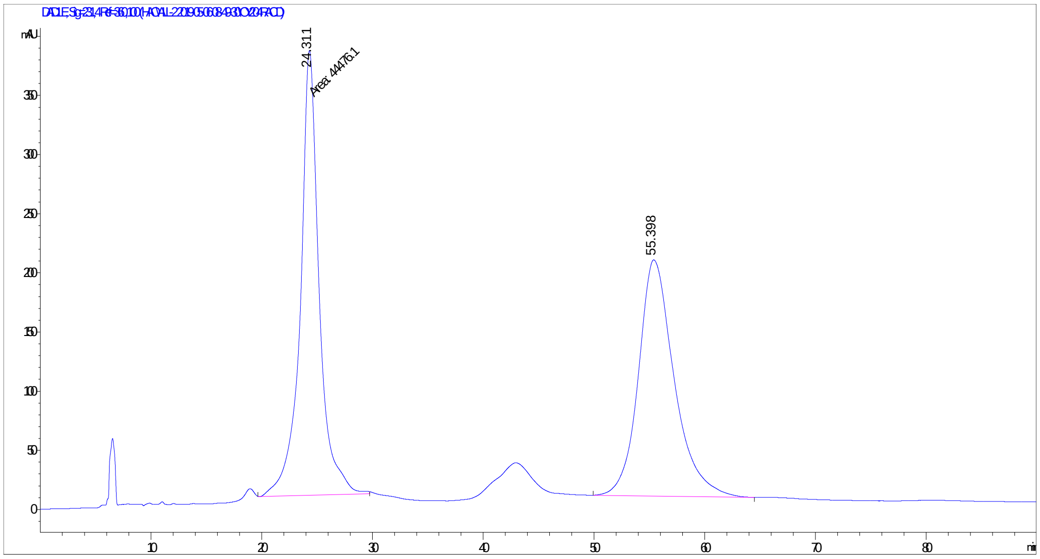

#	Time	Area	Height	Width	Area%	Symmetry
1	24.311	44476.1	375.9	1.9721	49.821	0.914
2	55.398	44795.4	199.7	2.7714	50.179	0.708

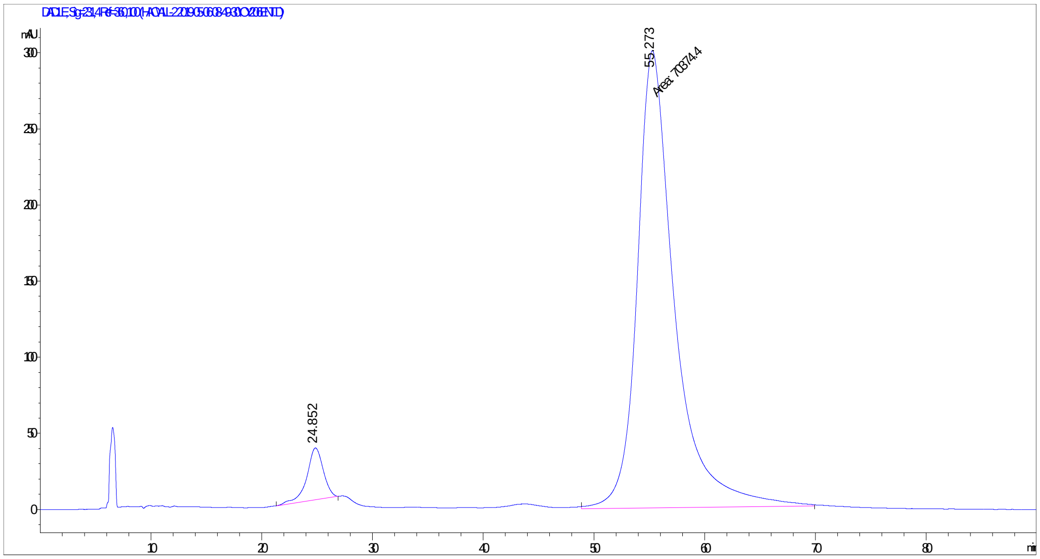

#	Time	Area	Height	Width	Area%	Symmetry
1	24.852	3532.5	34	1.4893	4.780	1.129
2	55.273	70374.4	300.5	3.903	95.220	0.658

^{1}H NMR

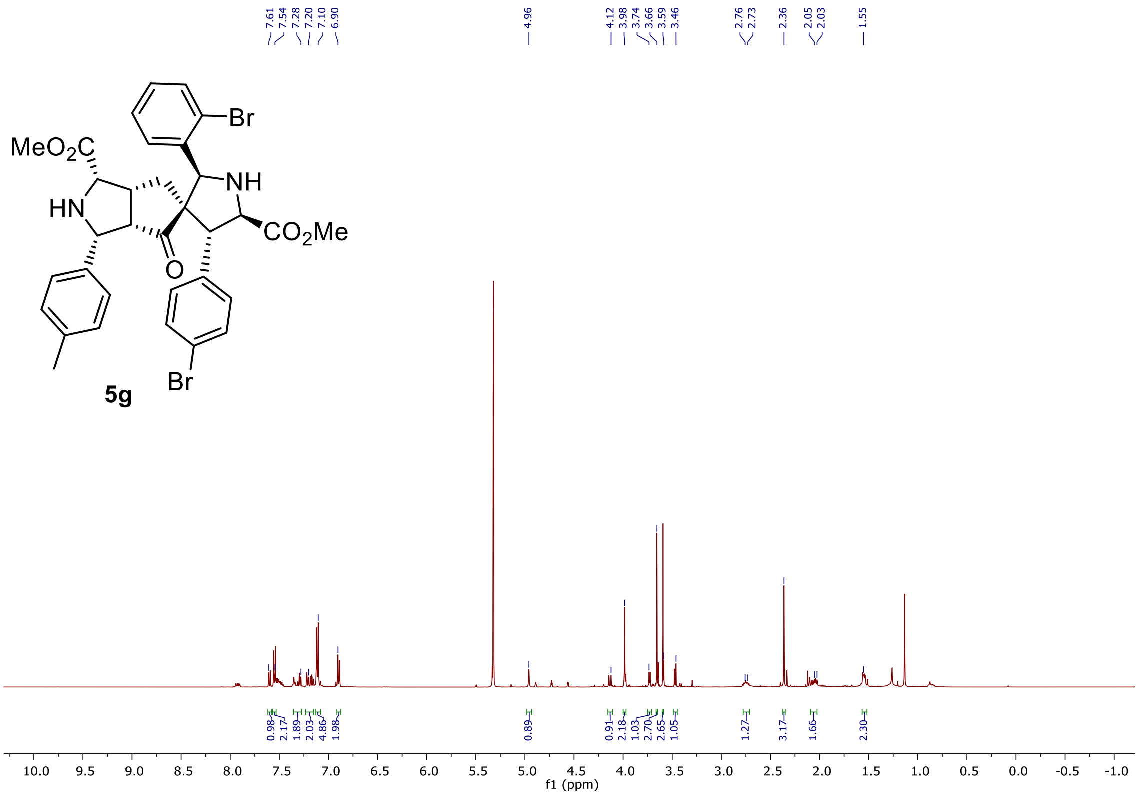

5g

^{13}C NMR

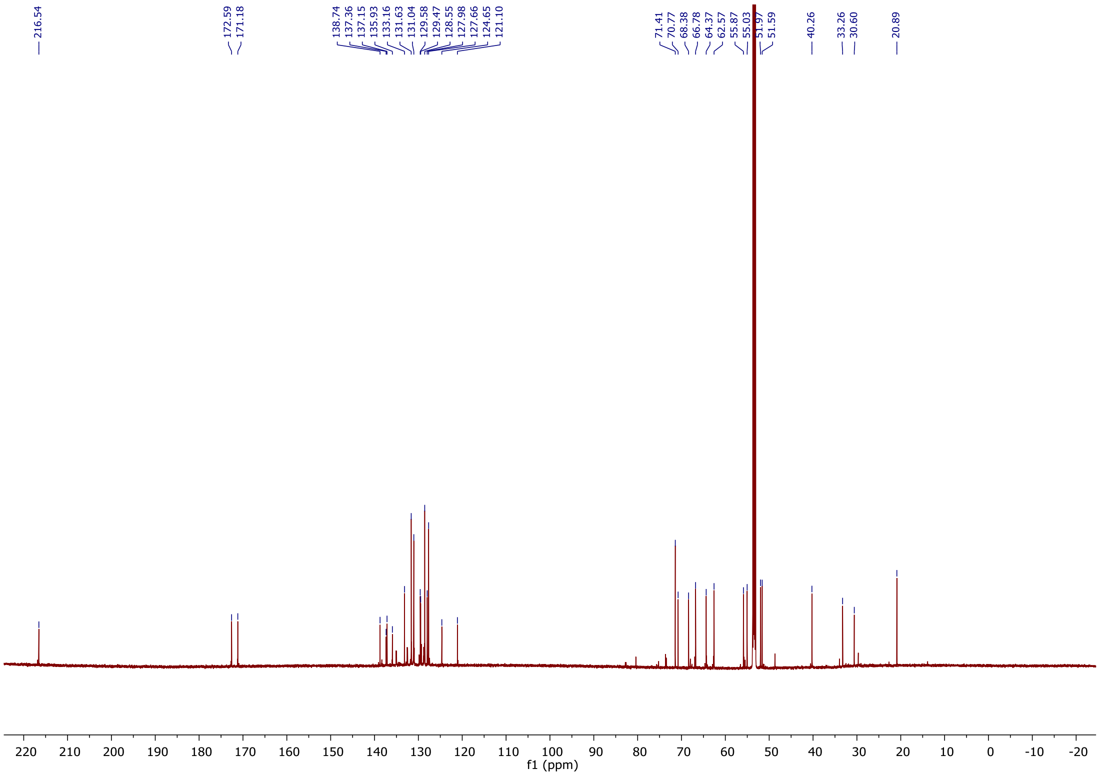

HPLC traces for **5g**: racemic top, enantiomer 1 bottom

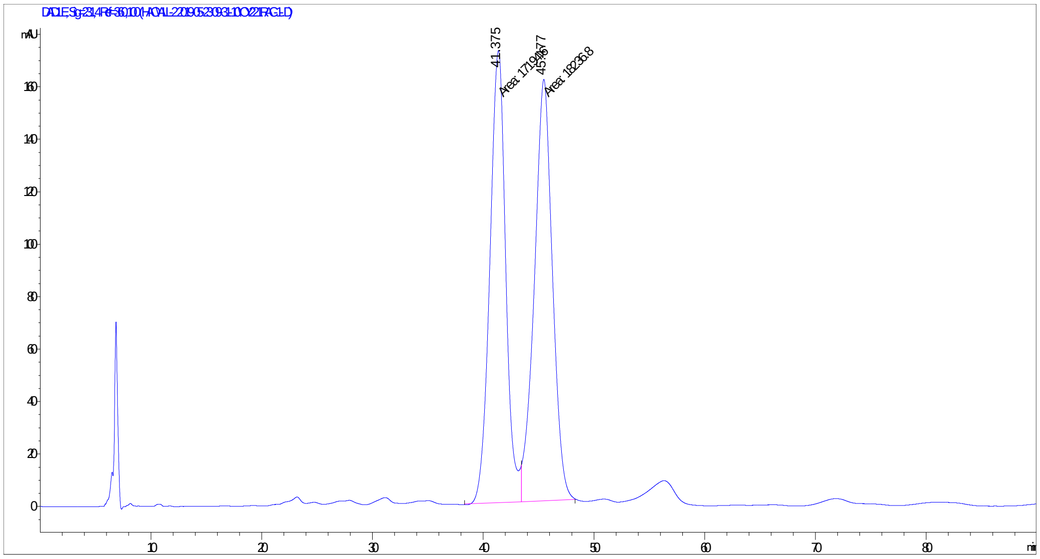

#	Time	Area	Height	Width	Area%	Symmetry
1	41.375	17194.6	172.4	1.6626	48.529	0
2	45.477	18236.8	160.7	1.891	51.471	1.065

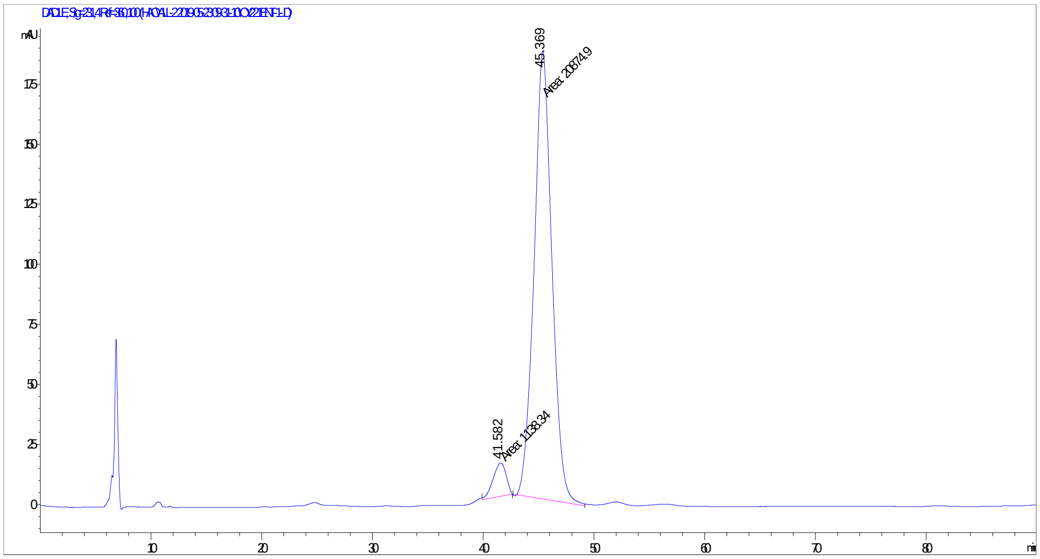

#	Time	Area	Height	Width	Area%	Symmetry
1	41.582	1138.3	13.8	1.3722	5.171	1.433
2	45.369	20874.9	186.5	1.8656	94.829	0.903

^{1}H NMR

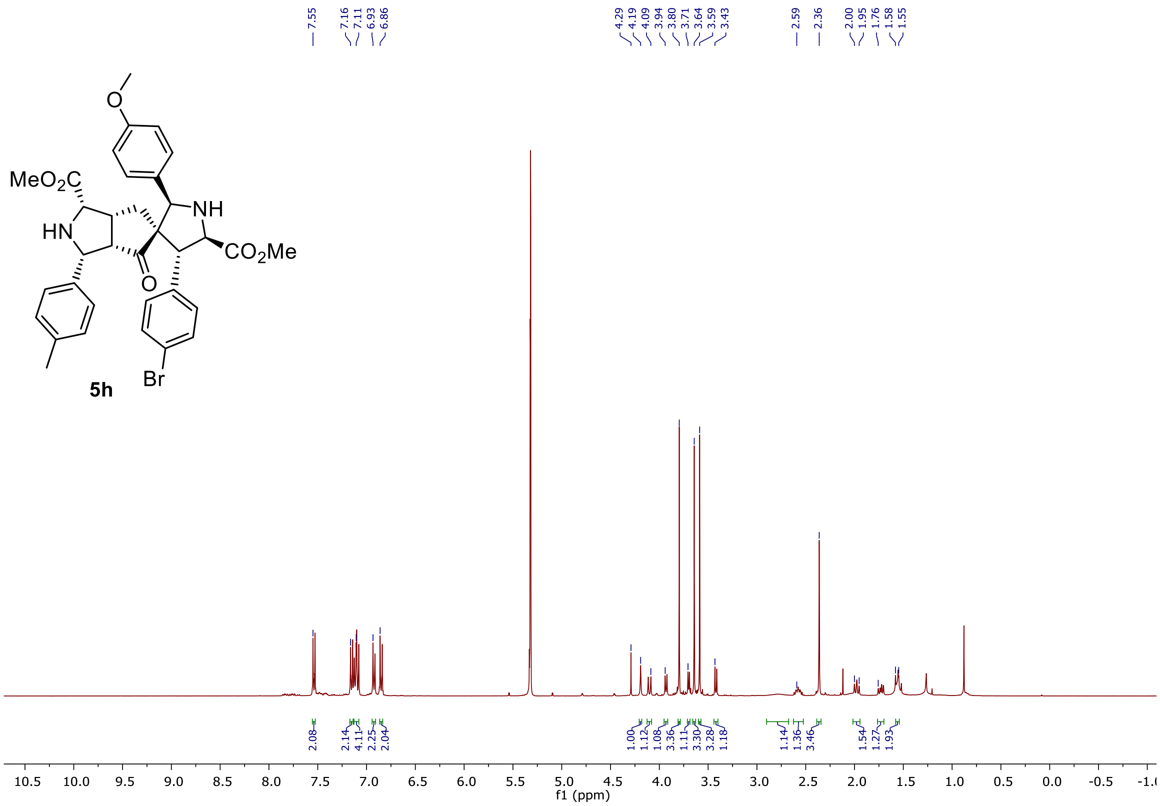

^{13}C NMR

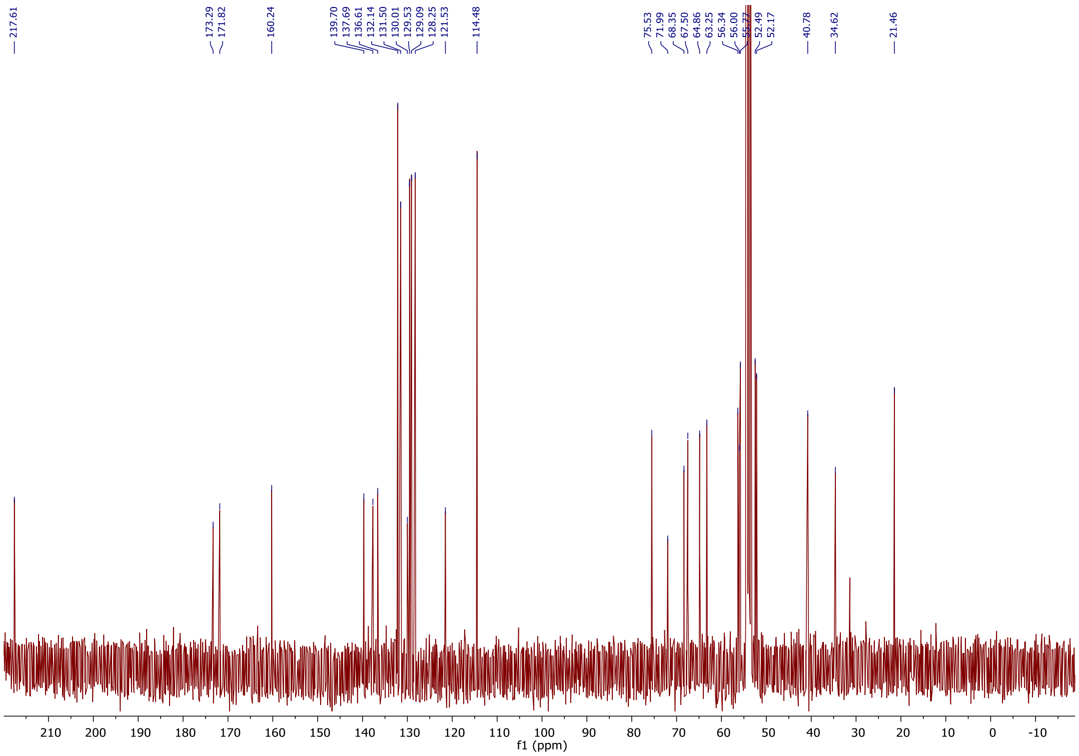

HPLC traces for **5h**: racemic top, enantiomer 1 bottom

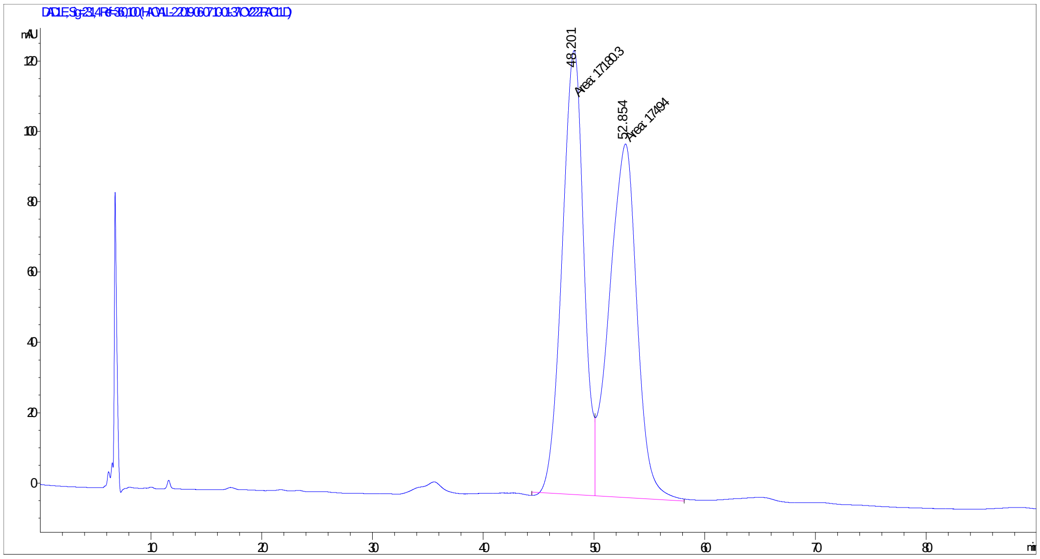

#	Time	Area	Height	Width	Area%	Symmetry
1	48.201	17180.3	126.3	2.268	49.548	1.167
2	52.854	17494	100.5	2.9001	50.452	1.305

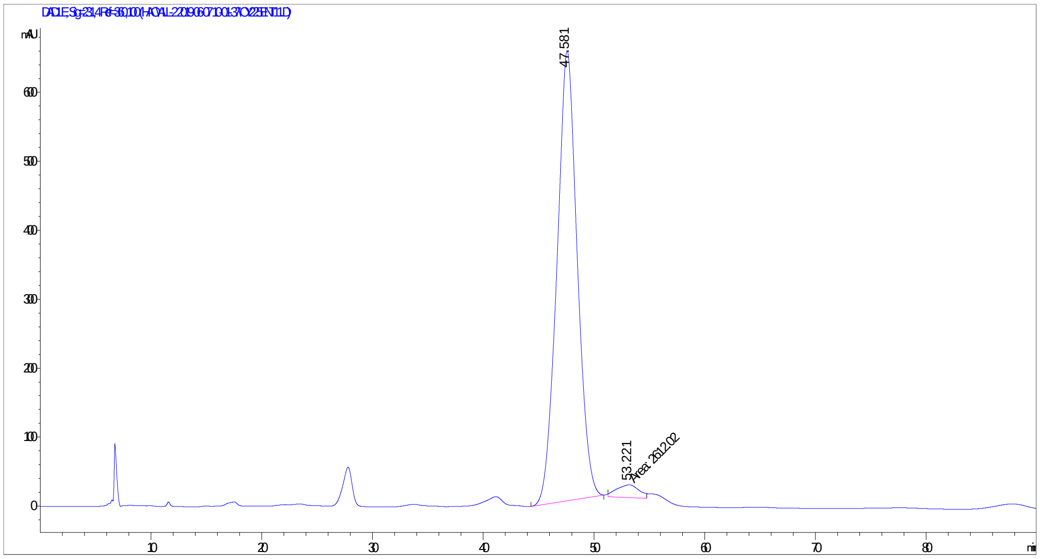

#	Time	Area	Height	Width	Area%	Symmetry
1	47.581	83091.4	652.6	1.8194	96.952	0.979
2	53.221	2612	18.5	2.3534	3.048	1.234

^{1}H NMR

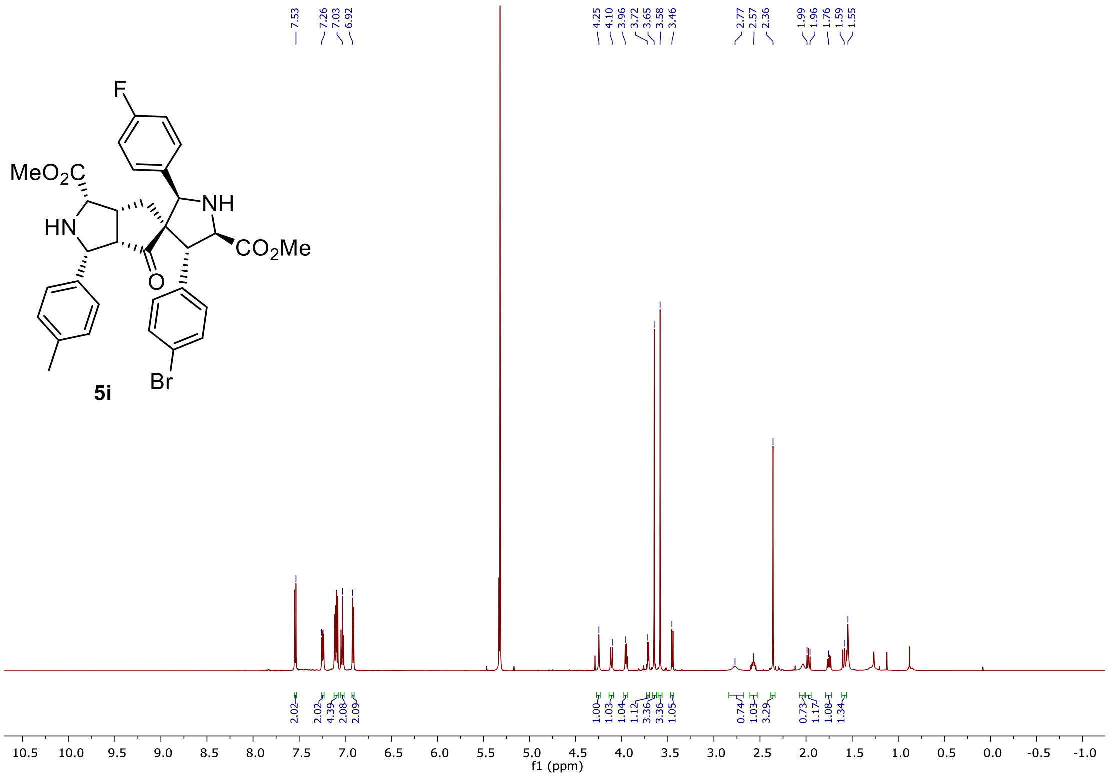

^{13}C NMR

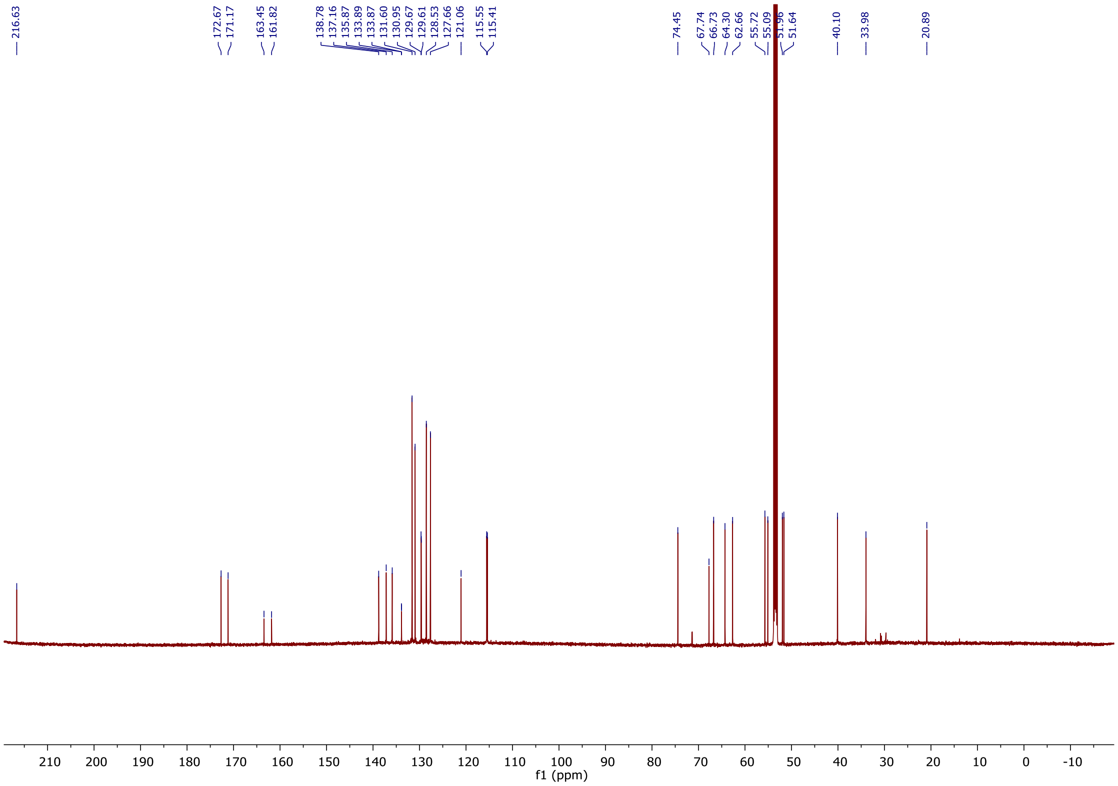

^{19}F NMR

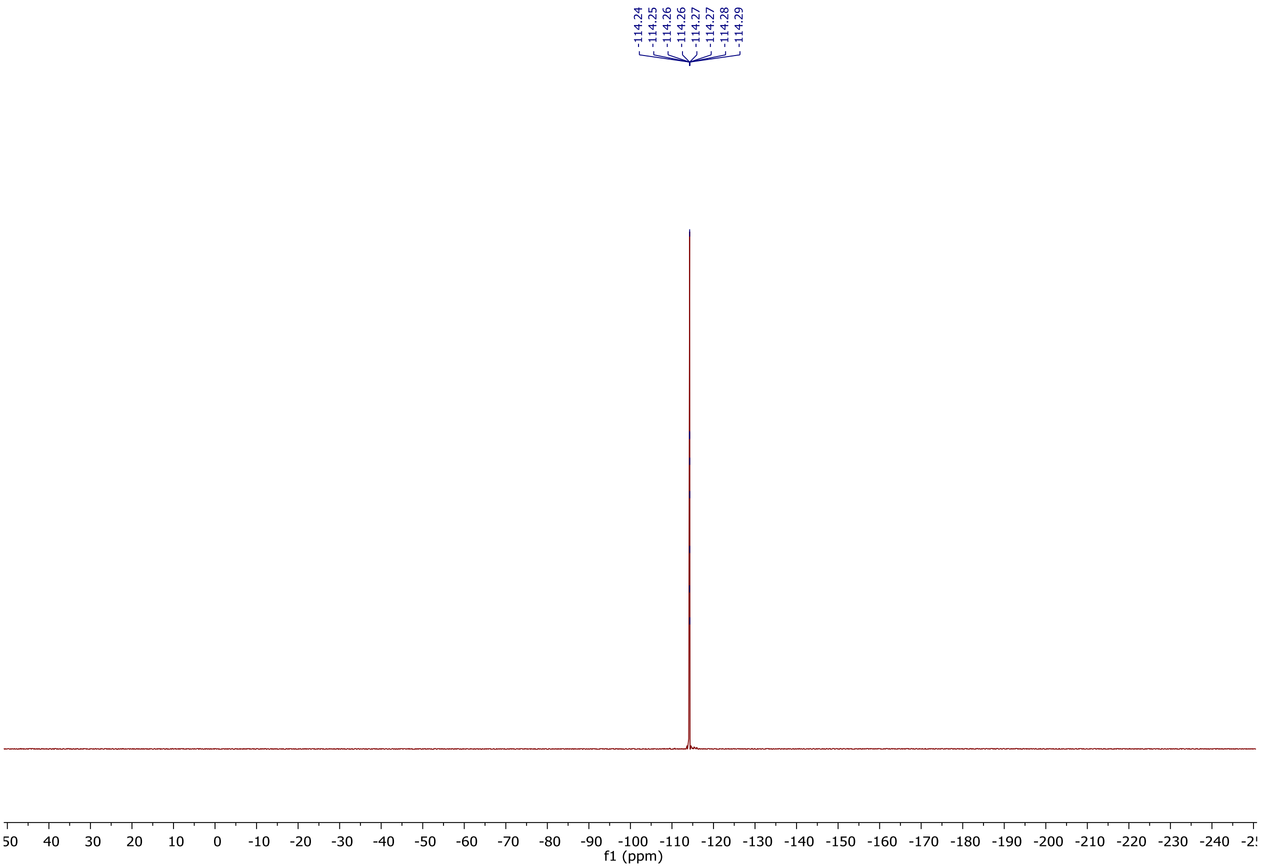

-114.24
-114.25
-114.26
-114.26
-114.27
-114.27
-114.28
-114.29
50 40 30 20 10 0 -10 -20 -30 -40 -50 -60 -70 -80 -90 -100 -110 -120 -130 -140 -150 -160 -170 -180 -190 -200 -210 -220 -230 -240
f1 (ppm)

HPLC traces for **5i**: racemic top, enantiomer 1 bottom

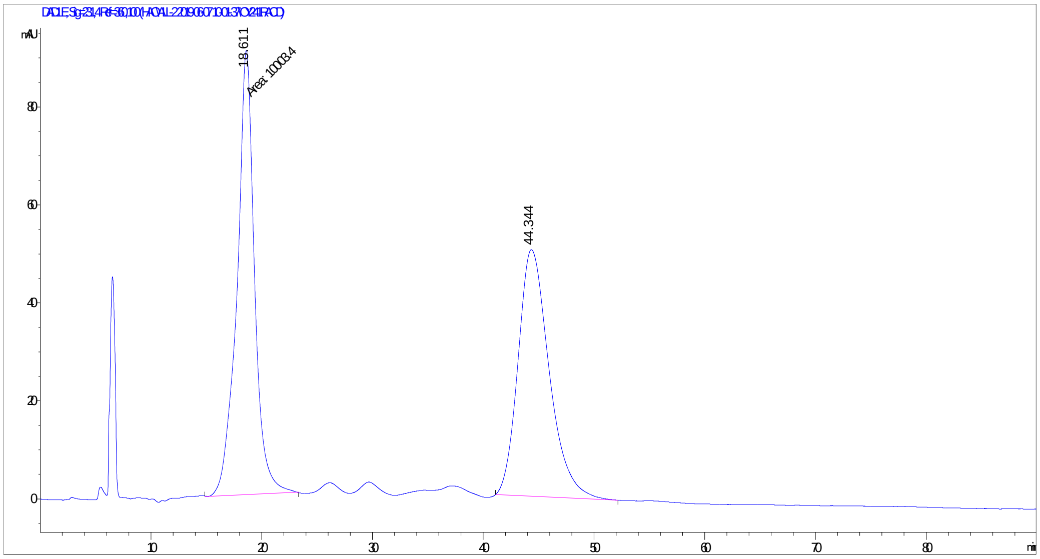

#	Time	Area	Height	Width	Area%	Symmetry
1	18.611	10003.4	90.7	1.839	50.456	1.084
2	44.344	9822.6	50.4	2.5439	49.544	0.701

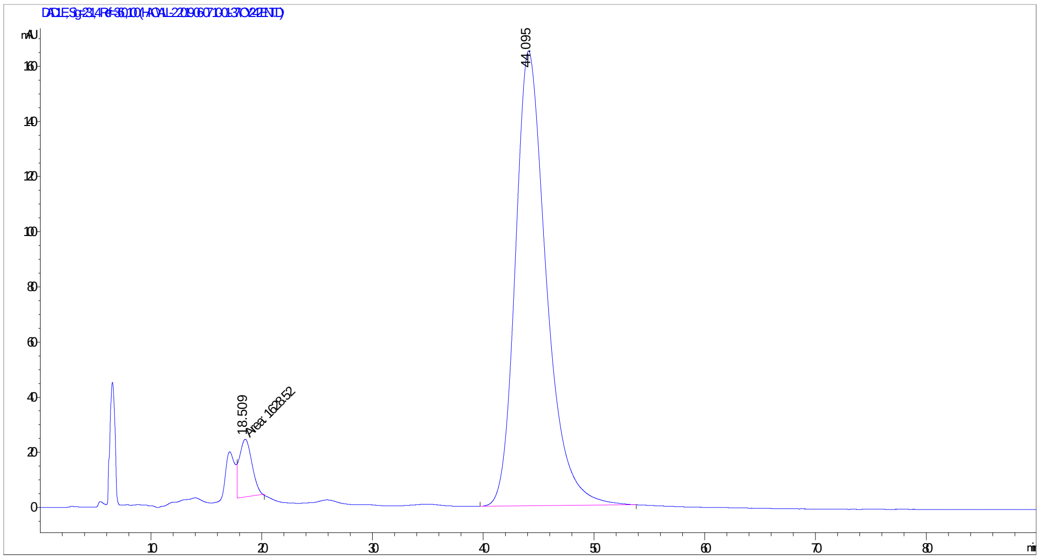

#	Time	Area	Height	Width	Area%	Symmetry
1	18.509	1628.5	20.9	1.2974	4.829	0.823
2	44.095	32097.3	165	2.6535	95.171	0.703

^{1}H NMR

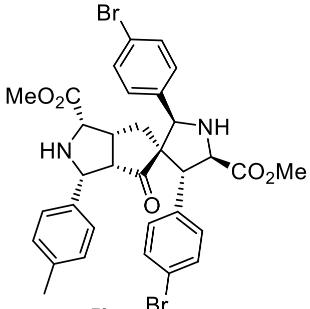

5j

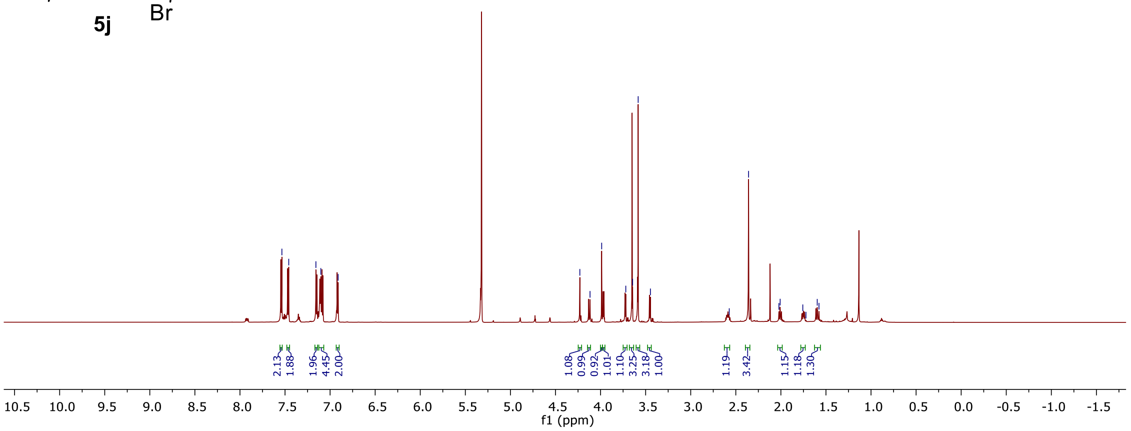

^{13}C NMR

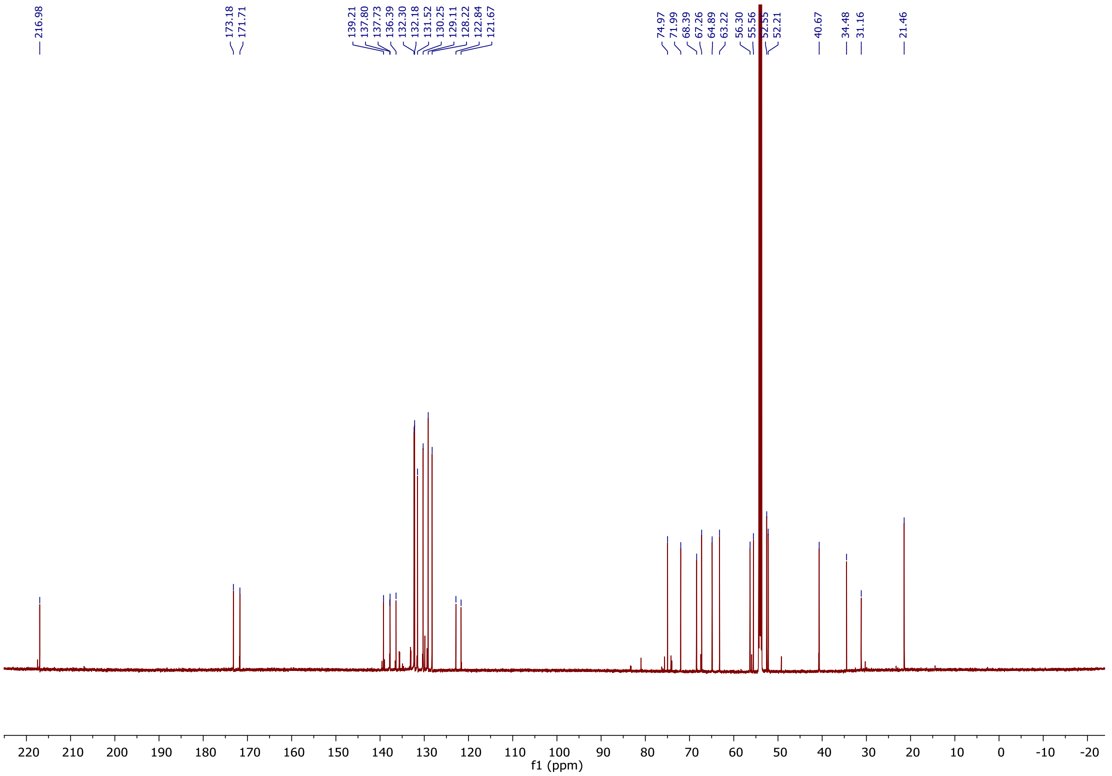

HPLC traces for **5j**: racemic top, enantiomer 1 bottom

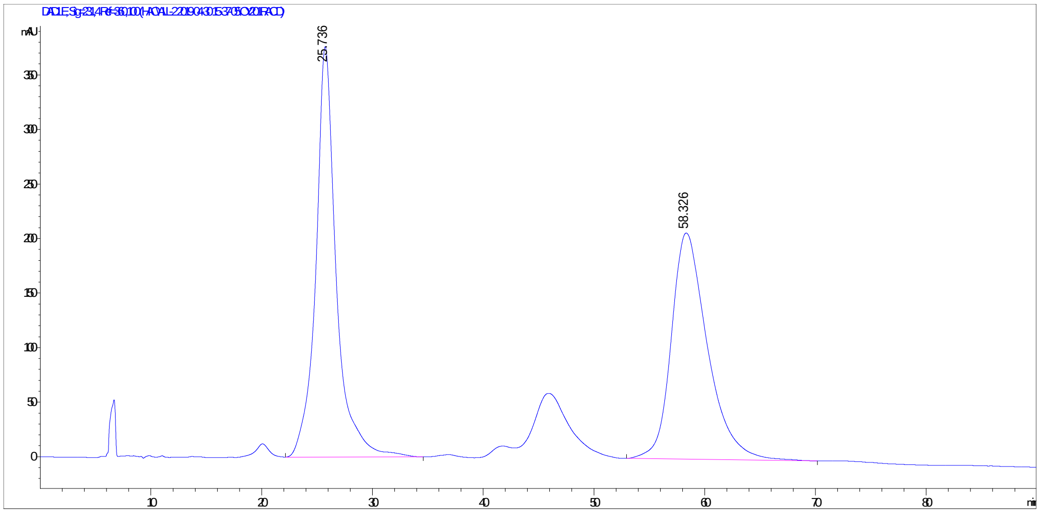

#	Time	Area	Height	Width	Area%	Symmetry
1	25.736	48258.3	376.2	1.8127	50.547	0.805
2	58.326	47213.7	207.4	3.18	49.453	0.666

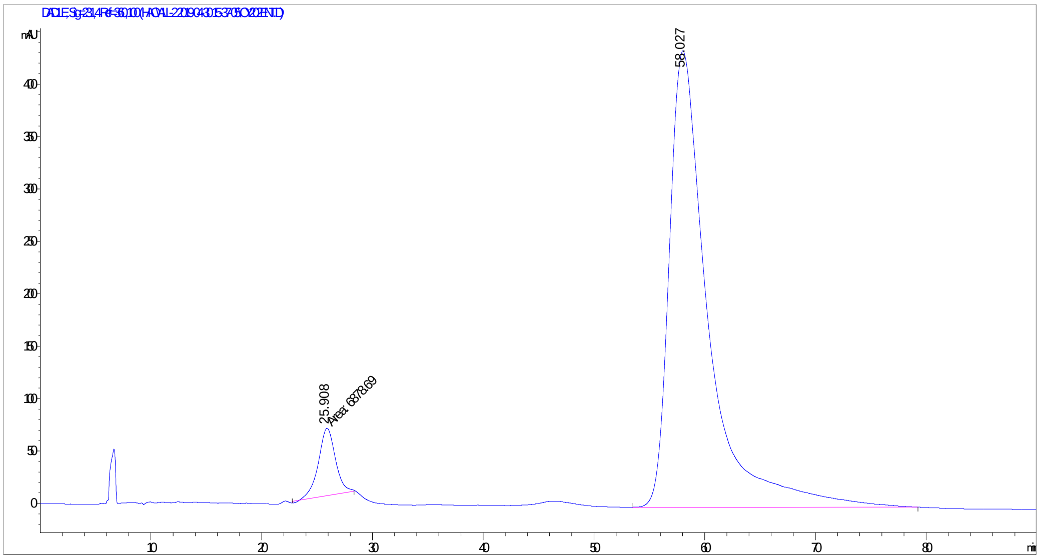

#	Time	Area	Height	Width	Area%	Symmetry
1	25.908	7364.6	65.3	1.8804	6.469	1.216
2	58.027	106484.5	435.3	3.376	93.531	0.488

^{1}H NMR

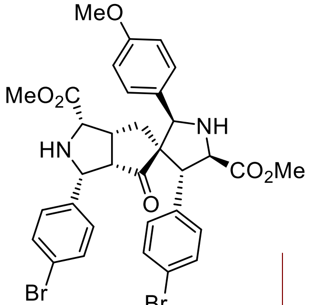

5k

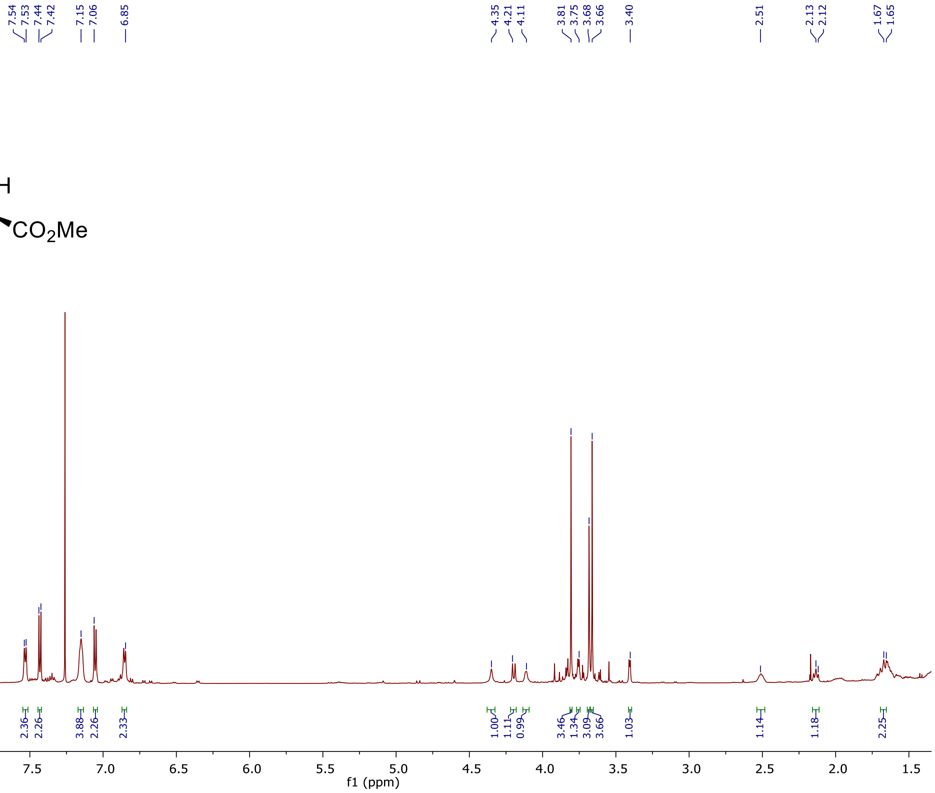

^{13}C NMR

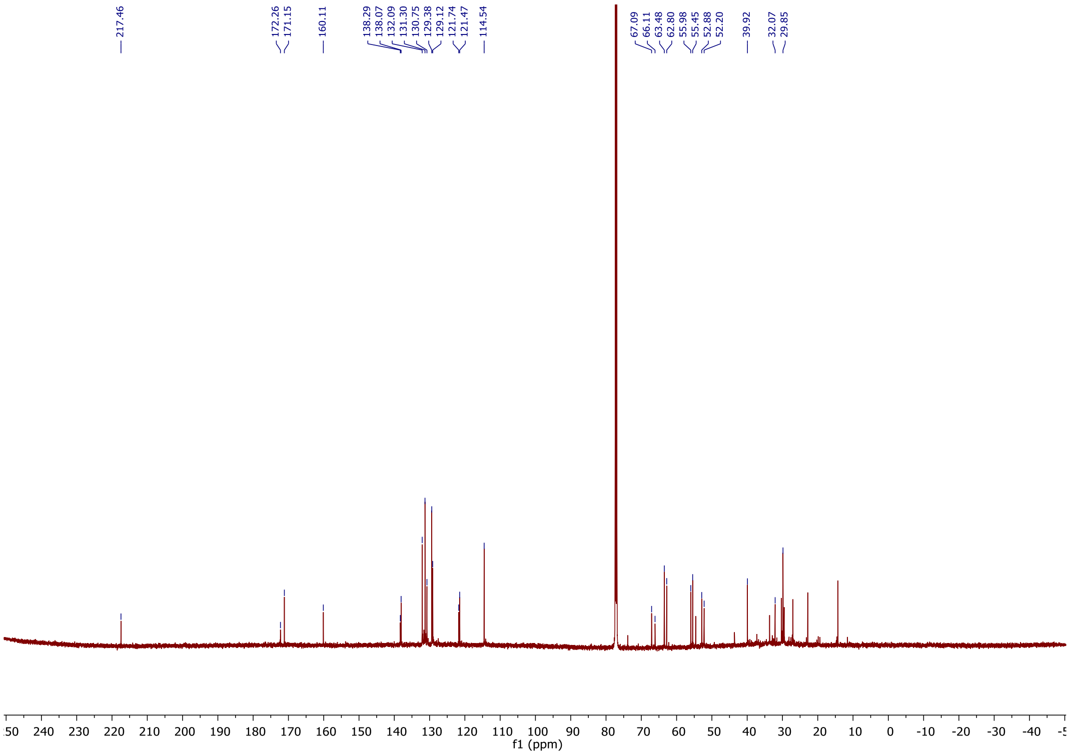

HPLC traces for **5k**

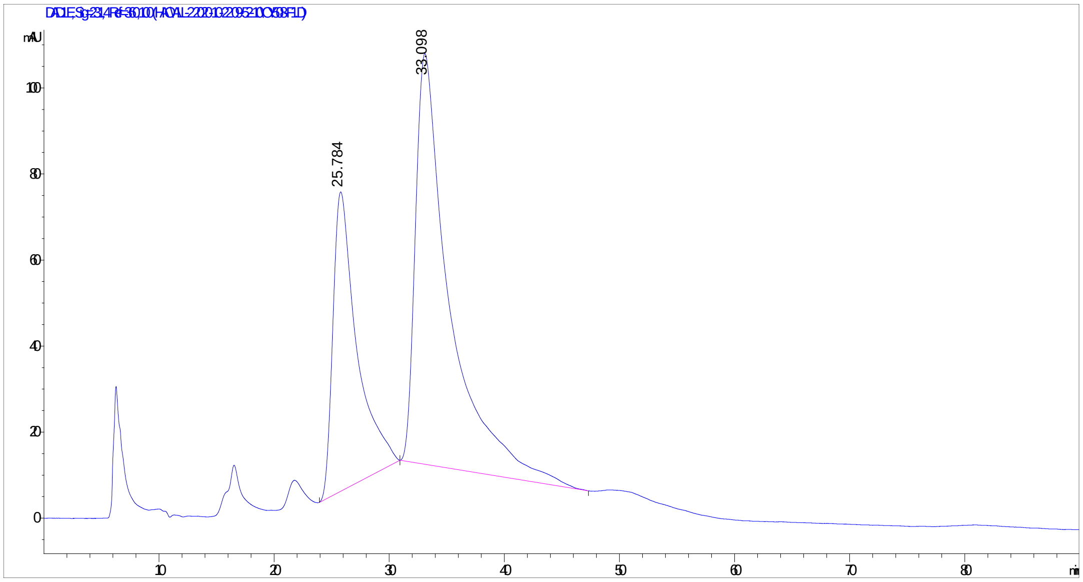

#	Time	Area	Height	Width	Area%	Symmetry
1	25.784	10076.8	69.6	2.0076	33.880	0.447
2	33.098	19666	95.5	2.7229	66.120	0.352

^{1}H NMR

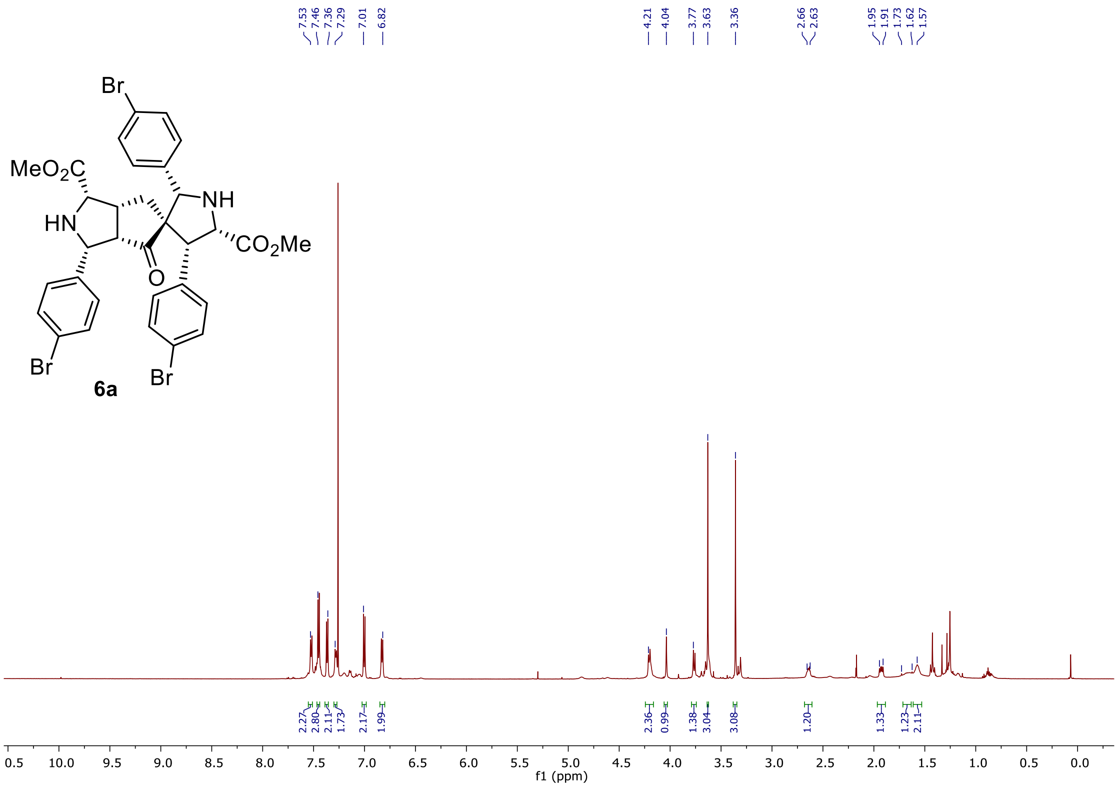

^{13}C NMR

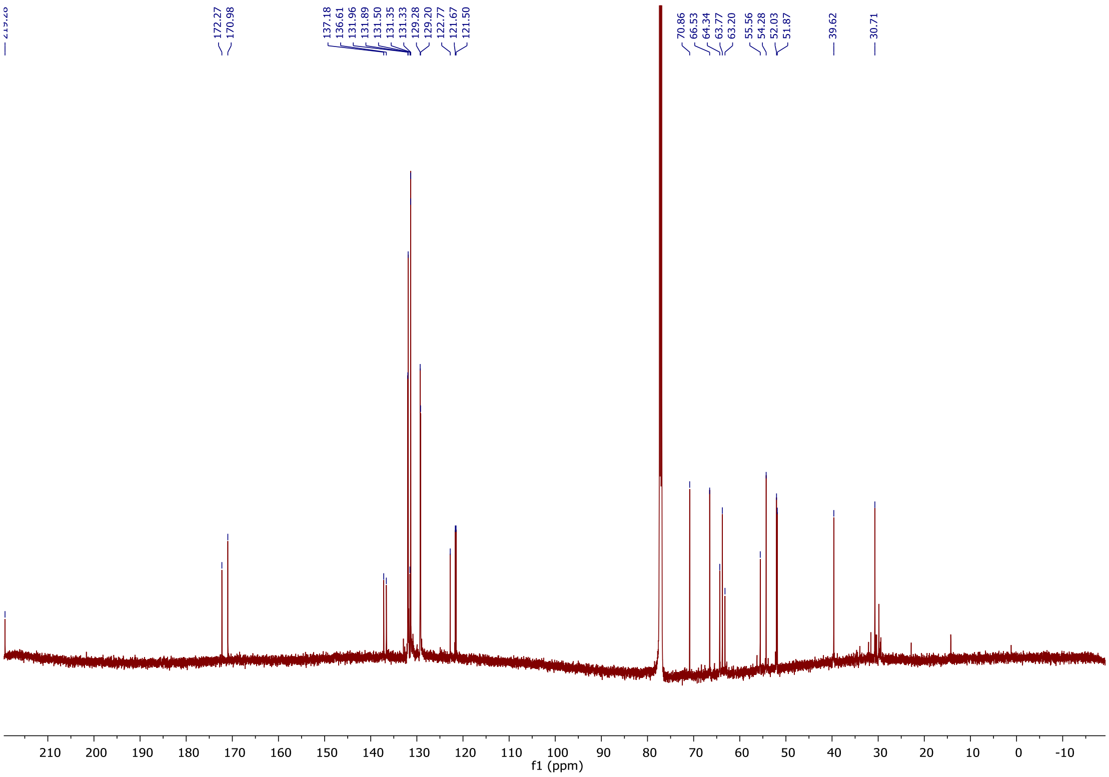

HPLC traces for **6a** on chiral **IA-column,** racemic top enantiomer 1 bottom

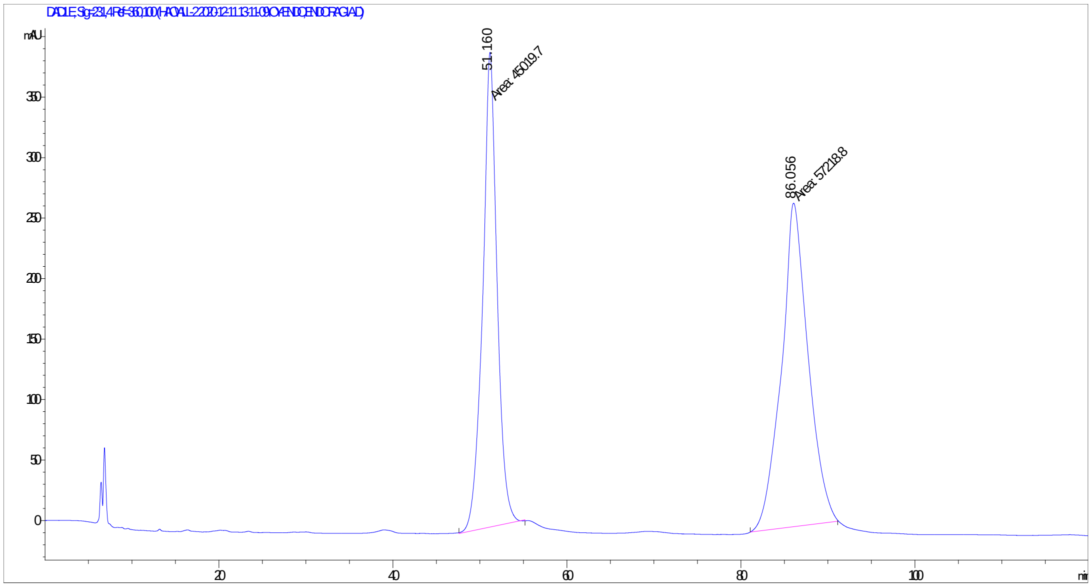

#	Time	Area	Height	Width	Area%	Symmetry
1	51.16	48163.6	397	2.022	49.107	0.919
2	86.056	49914.9	253.9	3.2762	50.893	0.873

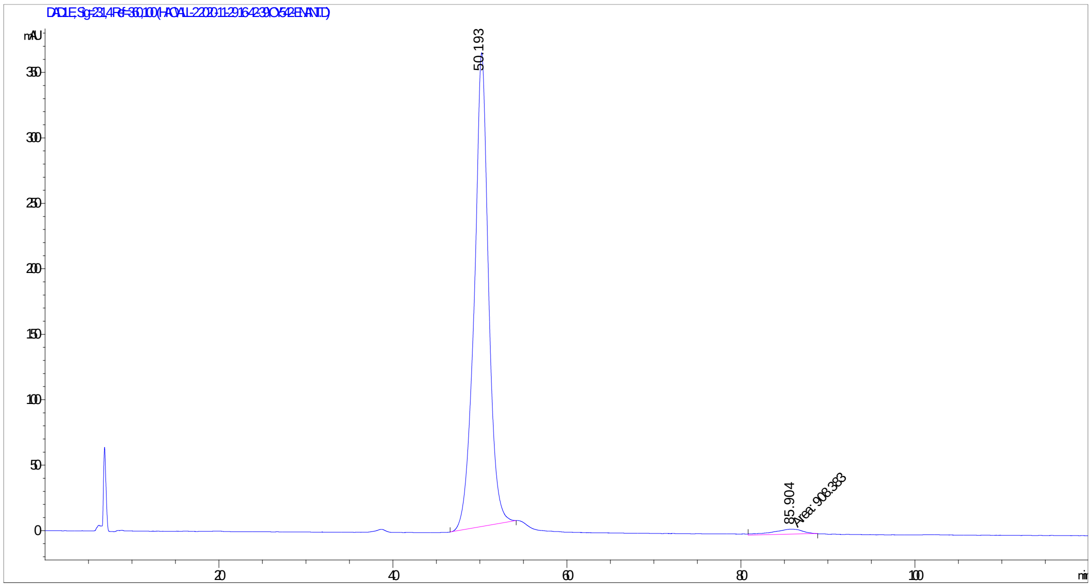

#	Time	Area	Height	Width	Area%	Symmetry
1	50.193	41127.6	362	1.6195	97.839	1.036
2	85.904	908.4	3.8	4.0007	2.161	1.982

HPLC traces for **6a** on chiral **IC-column,** racemic top enantiomer 1 bottom

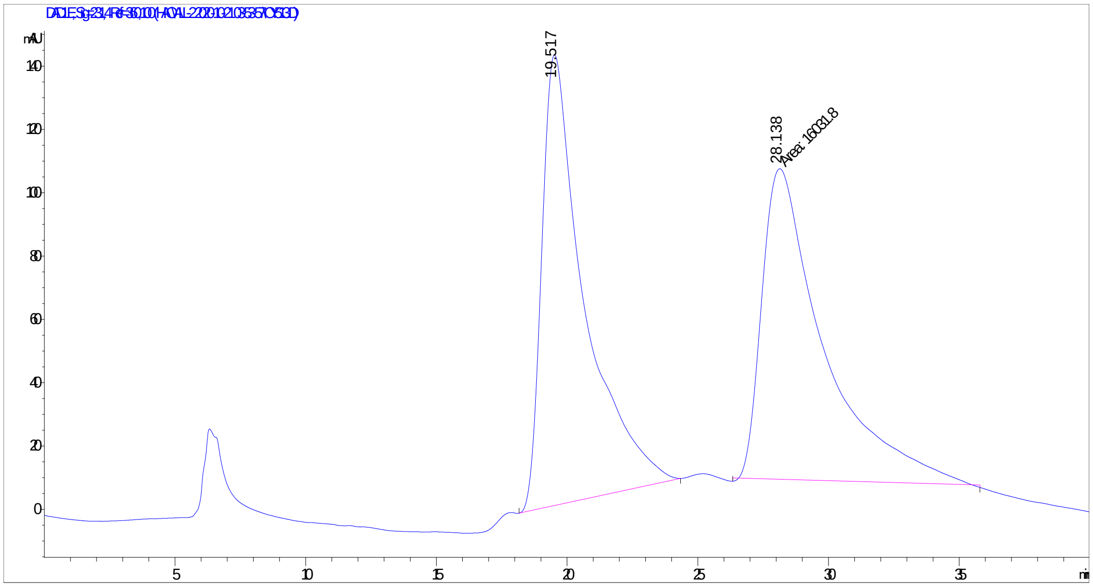

#	Time	Area	Height	Width	Area%	Symmetry
1	19.517	16121.4	142.4	1.5448	50.139	0.386
2	28.138	16031.8	98.1	2.7238	49.861	0.395

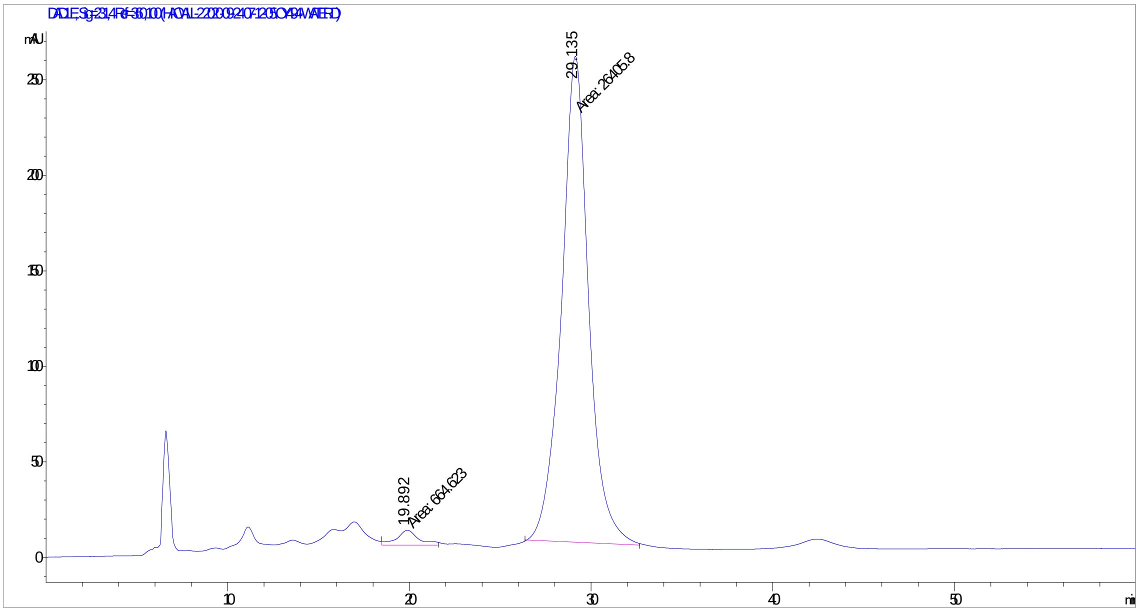

#	Time	Area	Height	Width	Area%	Symmetry
1	19.892	664.6	7.8	1.4272	2.455	0.922
2	29.135	26405.8	254.3	1.7303	97.545	1.002

^{1}H NMR

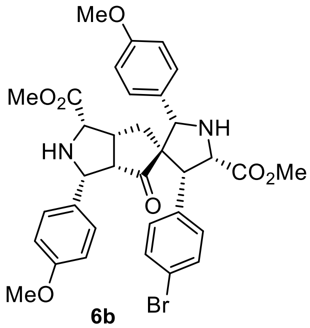

6b

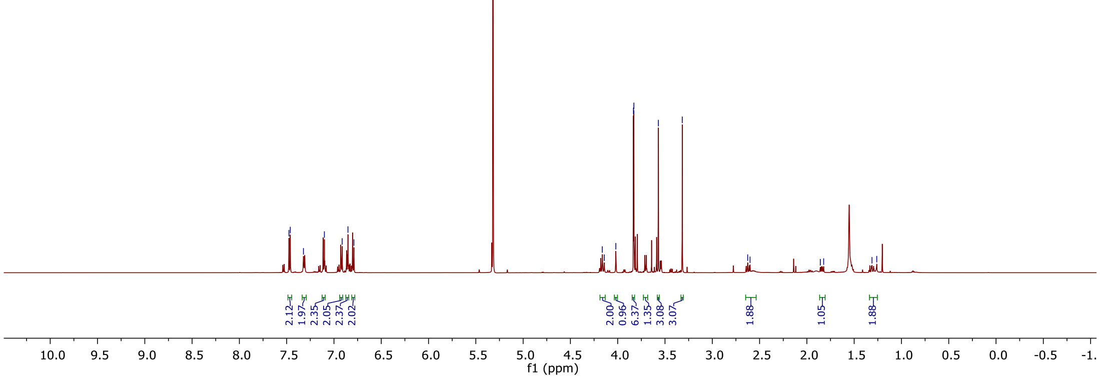

^{13}C NMR

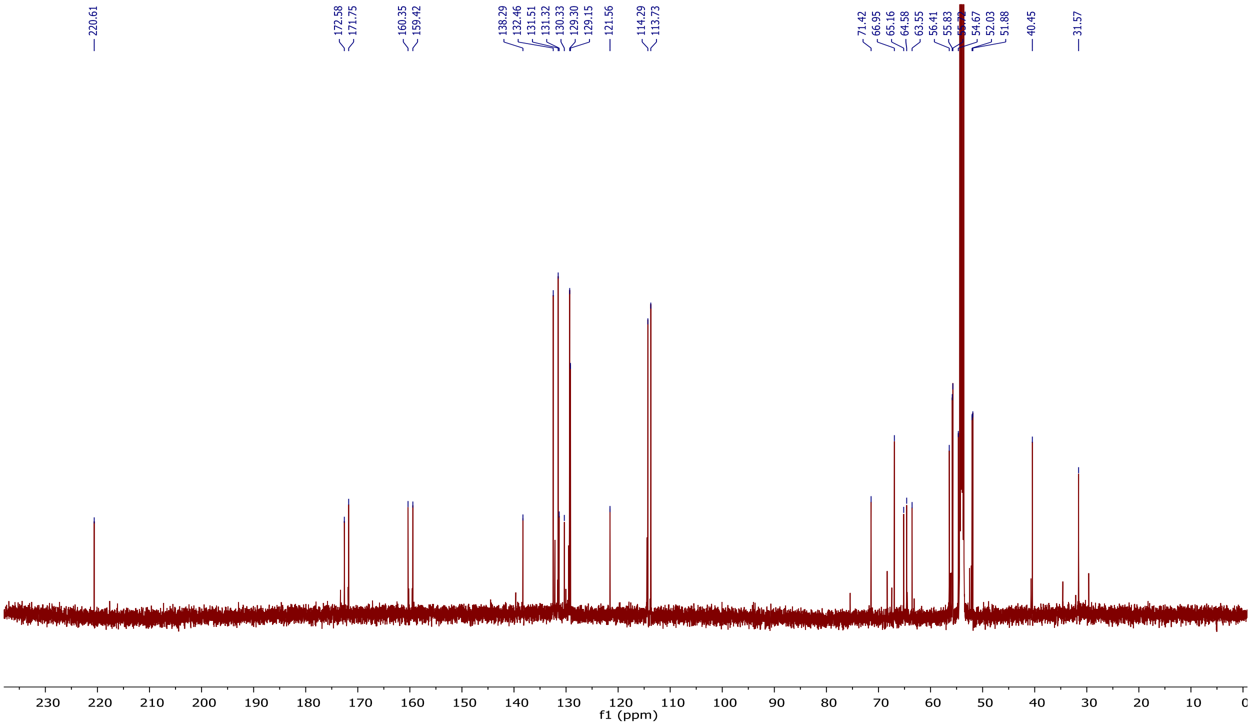

HPLC traces for **6b**, racemic top enantiomer 1 bottom

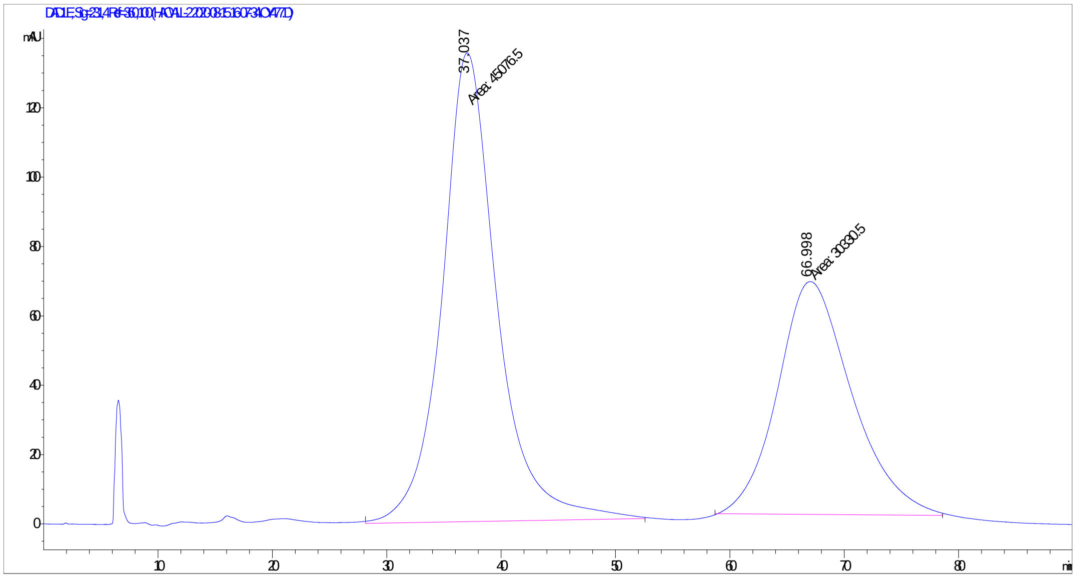

#	Time	Area	Height	Width	Area%	Symmetry
1	37.037	45076.5	135.2	5.5564	59.778	0.822
2	66.998	30330.5	67.1	7.5291	40.222	0.758

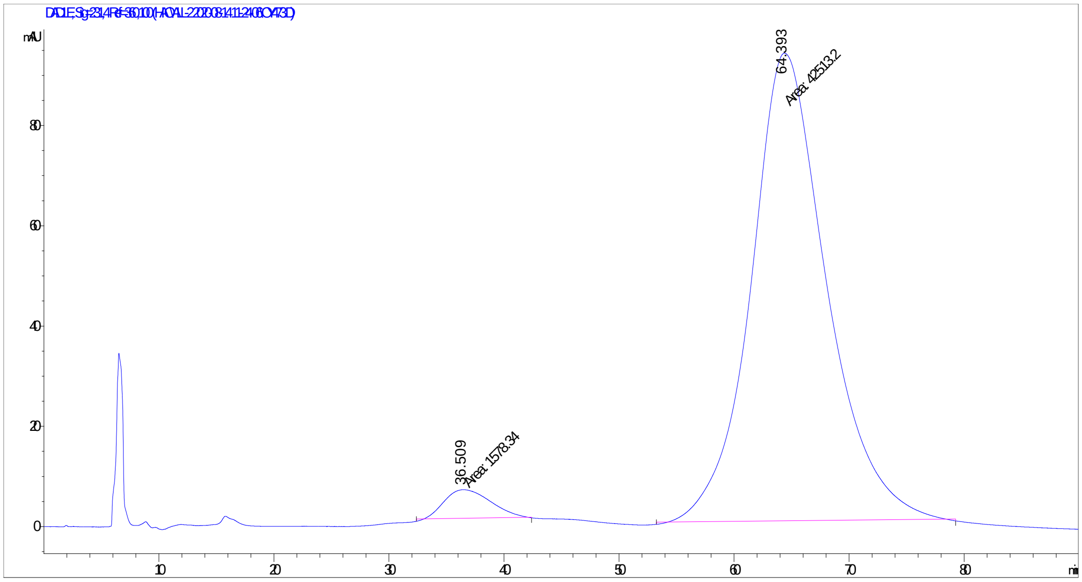

#	Time	Area	Height	Width	Area%	Symmetry
1	36.509	1578.3	5.7	4.6147	3.580	0.719
2	64.393	42513.2	93.3	7.5957	96.420	0.788

^{1}H NMR

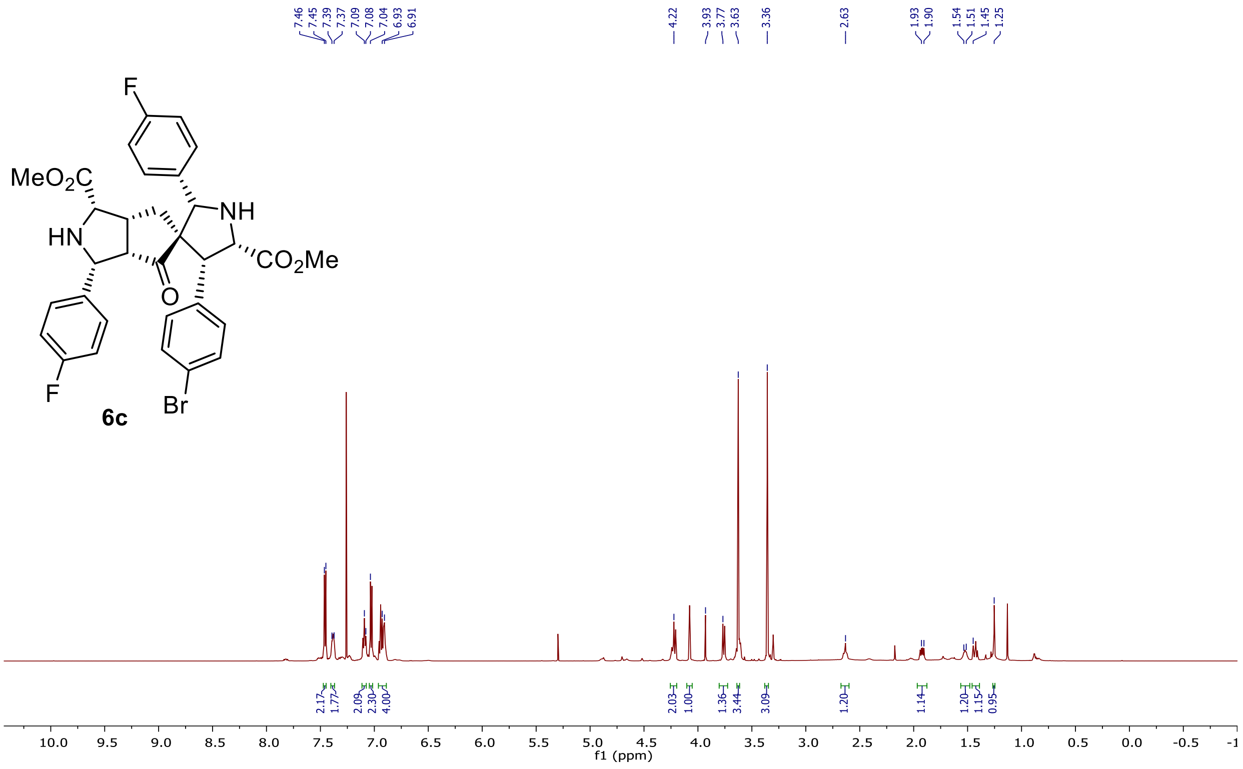

^{13}C NMR

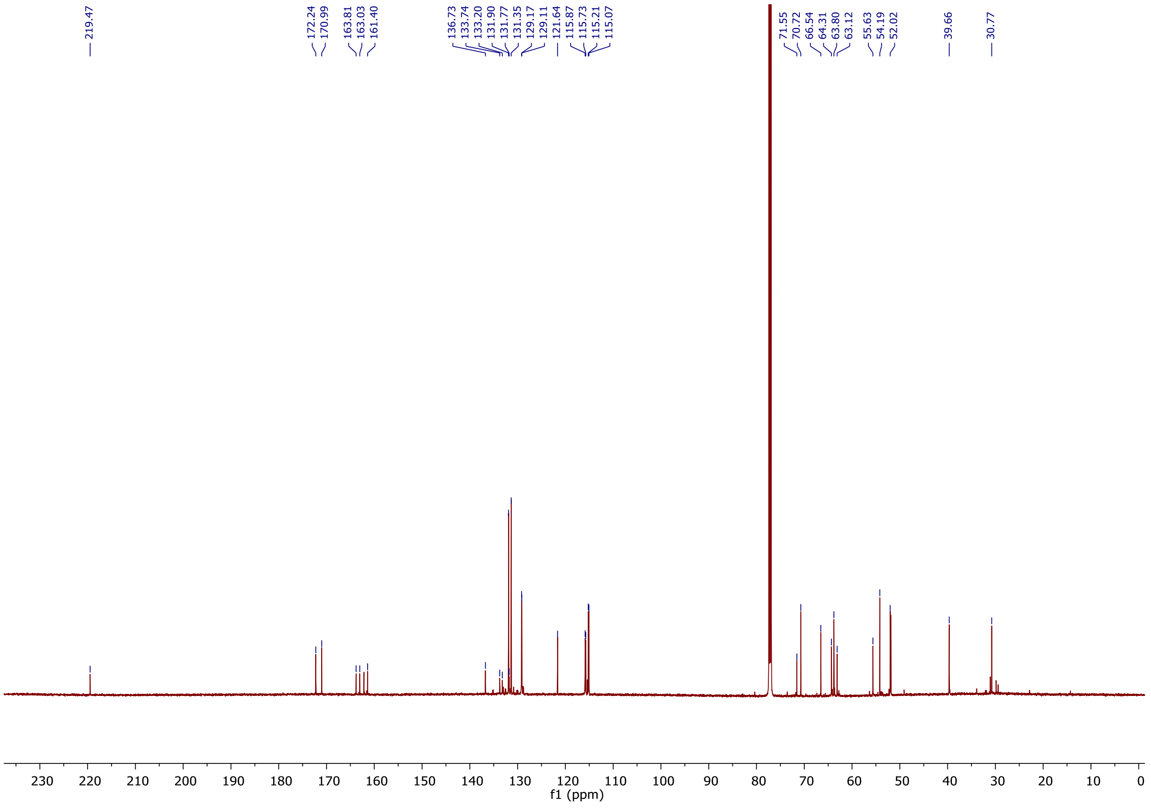

HPLC traces for **6c**, racemic top enantiomer 1 bottom

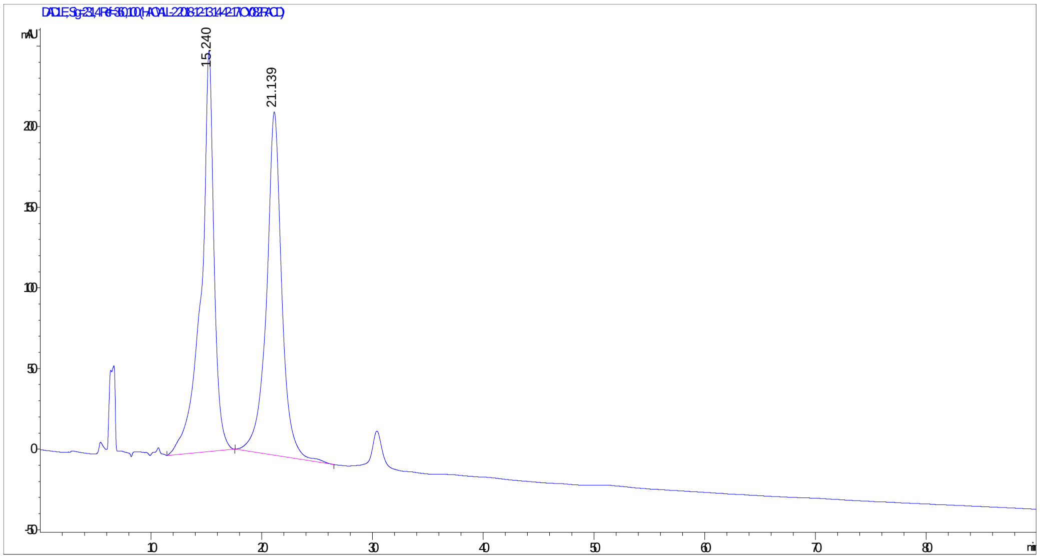

#	Time	Area	Height	Width	Area%	Symmetry
1	15.24	20157.6	248.7	1.1156	49.582	1.57
2	21.139	20497.7	212.9	1.3813	50.418	1.013

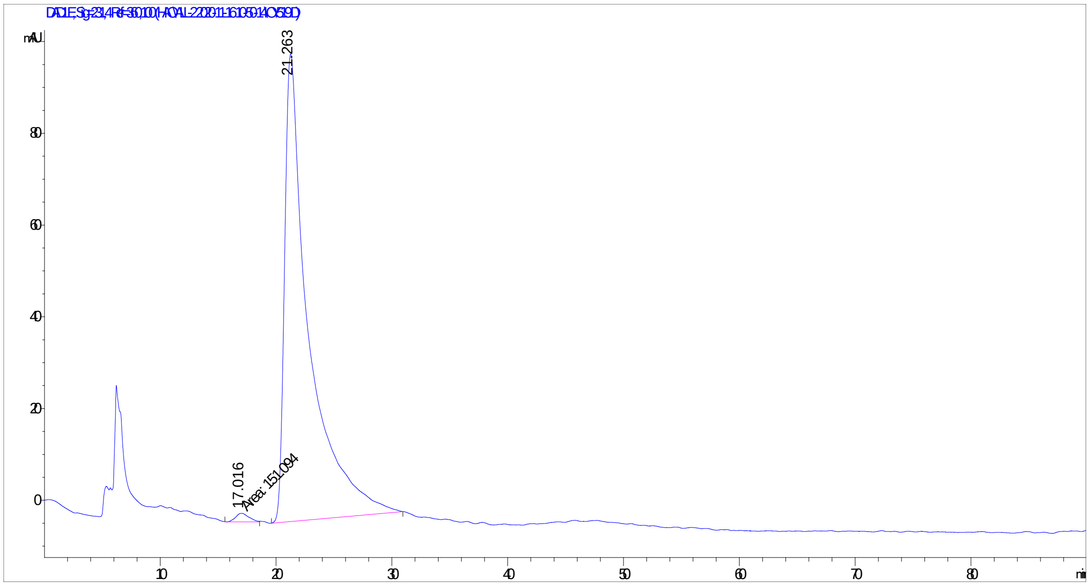

#	Time	Area	Height	Width	Area%	Symmetry
1	17.016	151.1	1.9	1.3344	1.011	0.764
2	21.263	14801.2	101.9	1.9522	98.989	0.301

^{1}H NMR

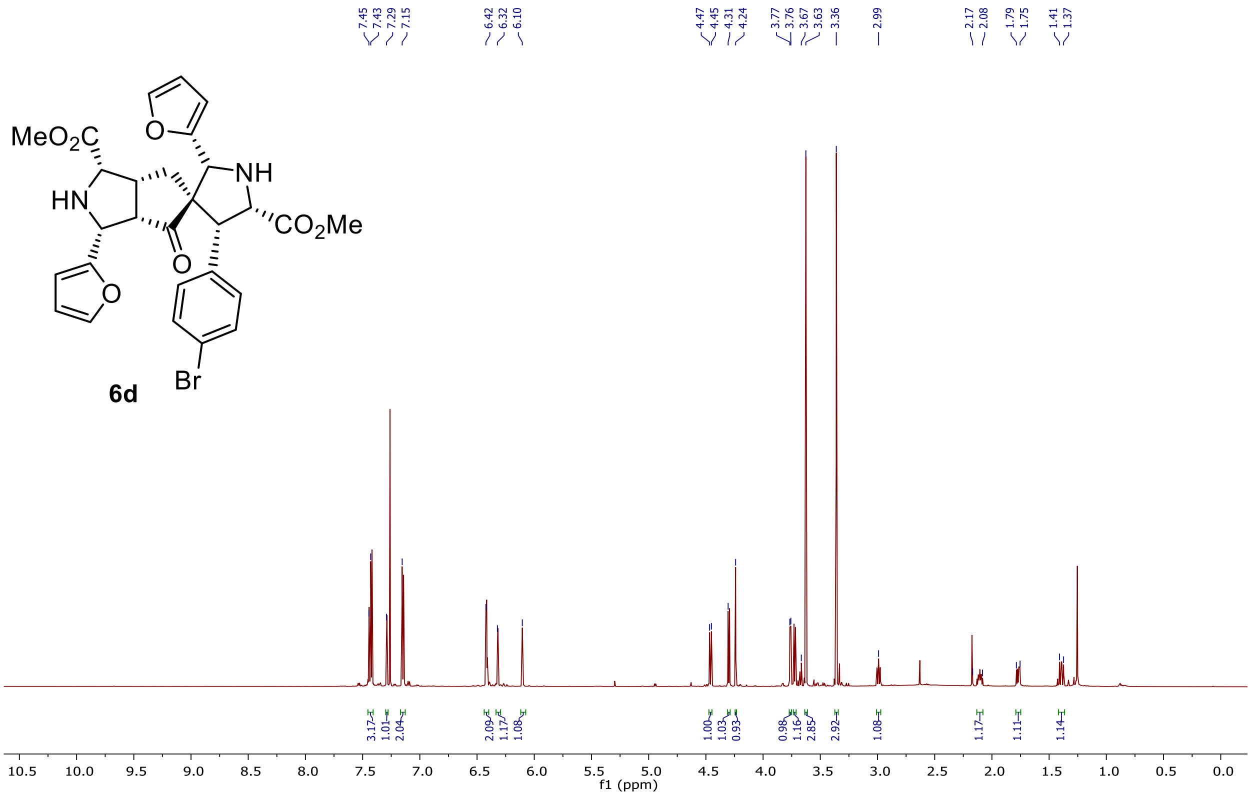

^{13}C NMR

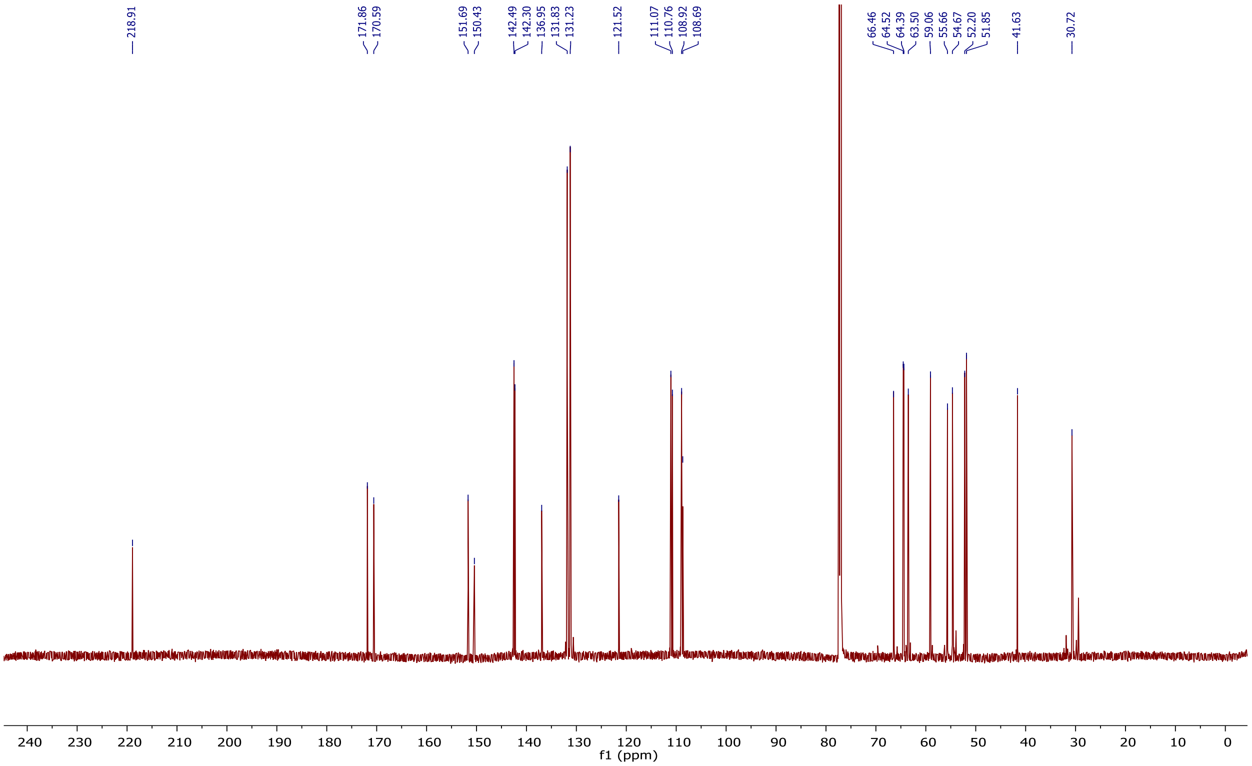

HPLC traces for **6d**, racemic top enantiomer 1 bottom

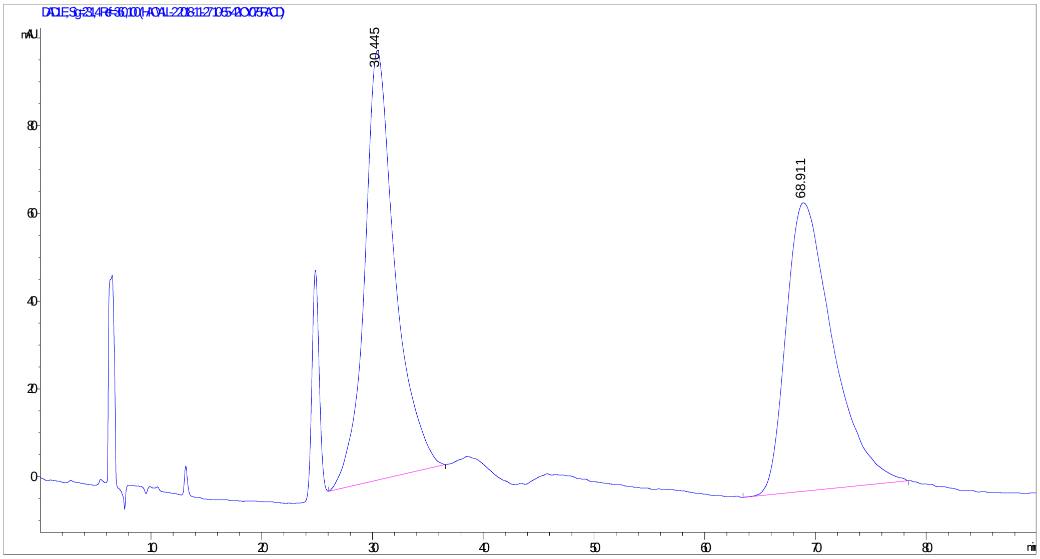

#	Time	Area	Height	Width	Area%	Symmetry
1	30.445	18867.8	97.9	2.7285	49.497	0.693
2	68.911	19250.9	65.8	3.7418	50.503	0.546

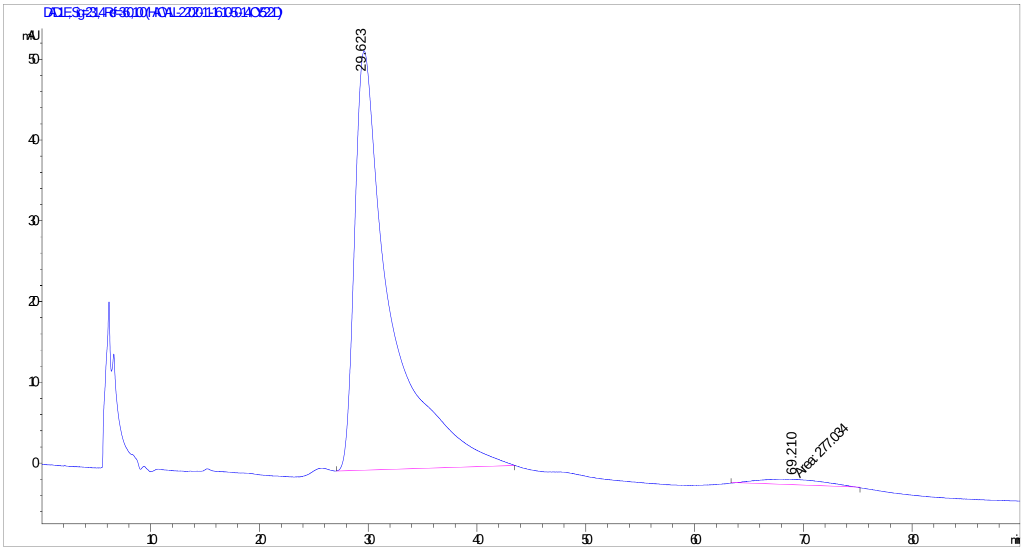

#	Time	Area	Height	Width	Area%	Symmetry
1	29.623	11920.7	51.9	2.9671	97.729	0.33
2	69.21	277	6.7E-1	6.8472	2.271	0.798

^{1}H NMR

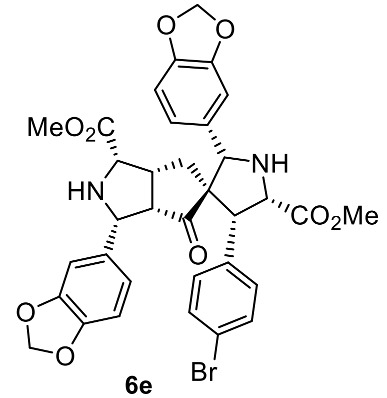

6e

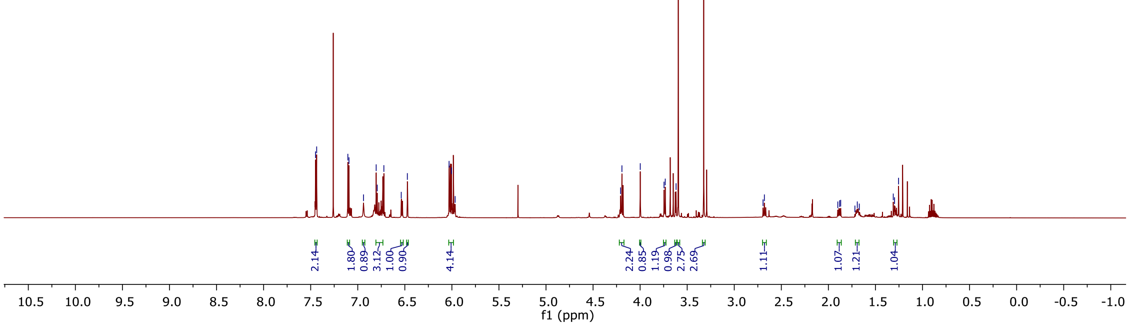

^{13}C NMR

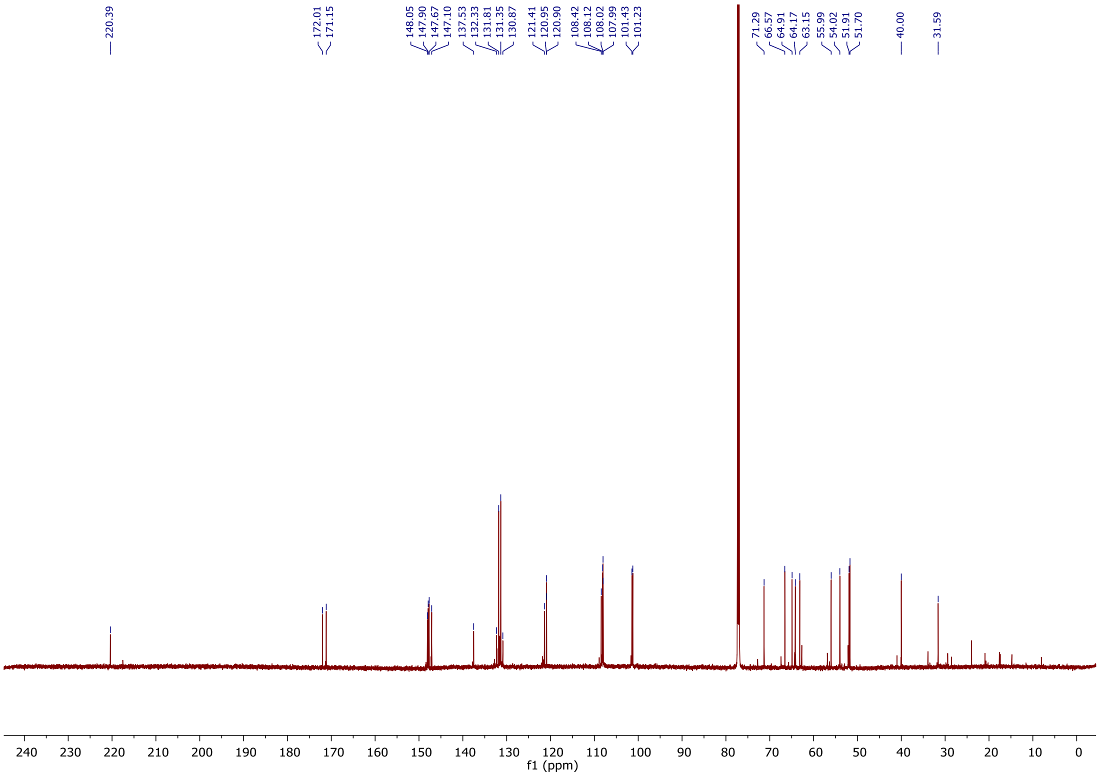

HPLC traces for **6e**, racemic top enantiomer 1 bottom

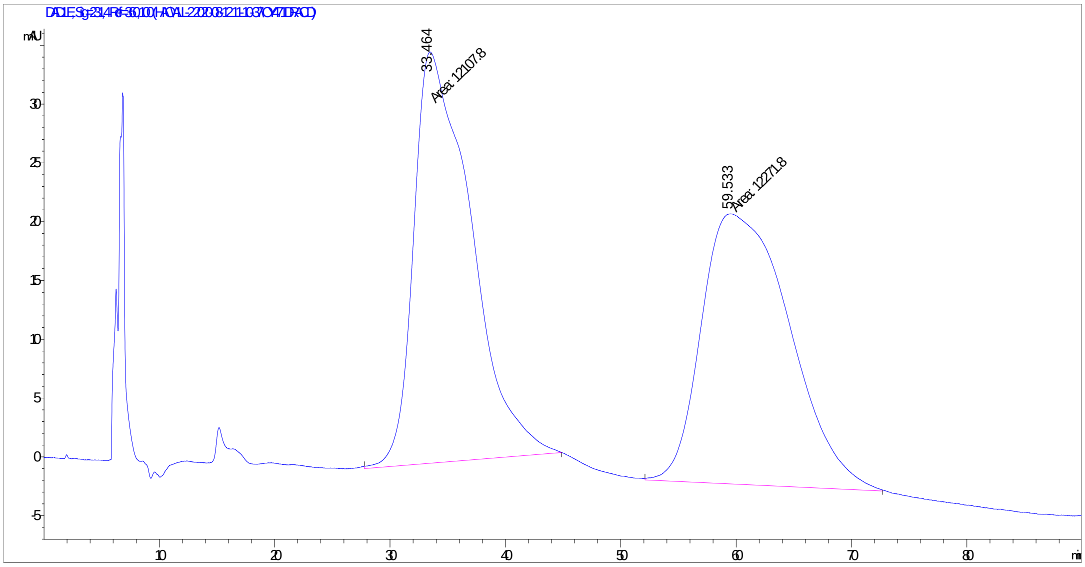

#	Time	Area	Height	Width	Area%	Symmetry
1	33.464	12107.8	35	5.7676	49.664	0.405
2	59.533	12271.8	23	8.9028	50.336	0.497

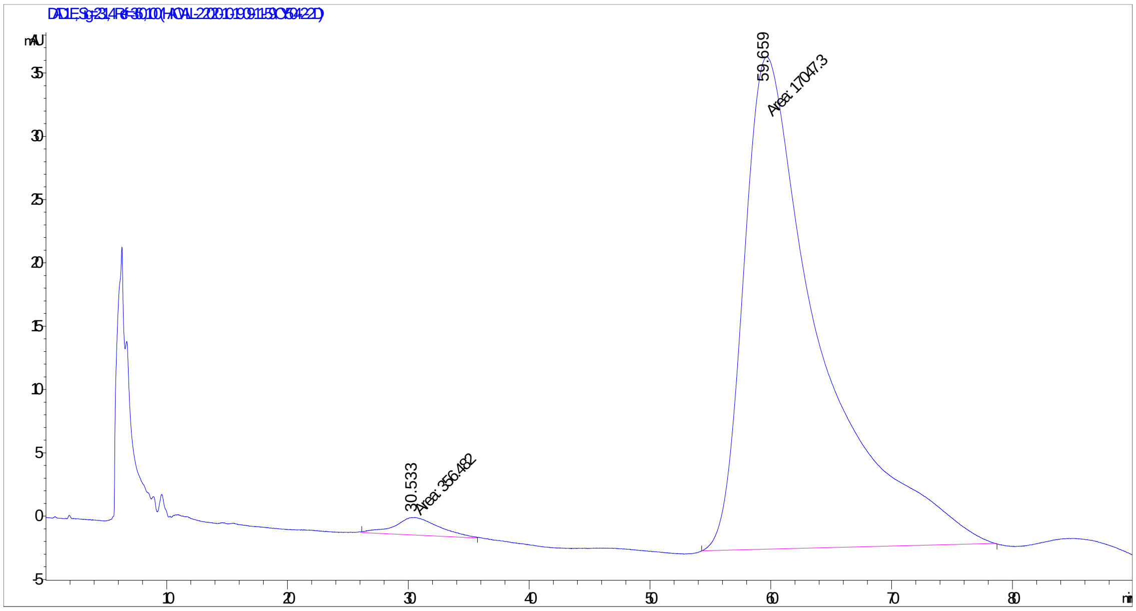

#	Time	Area	Height	Width	Area%	Symmetry
1	30.533	356.5	1.4	4.2745	2.048	0.801
2	59.659	17047.3	38.9	7.3107	97.952	0.417

^{1}H NMR

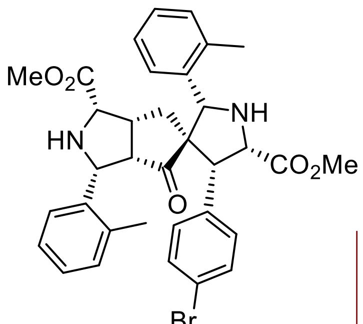

6f

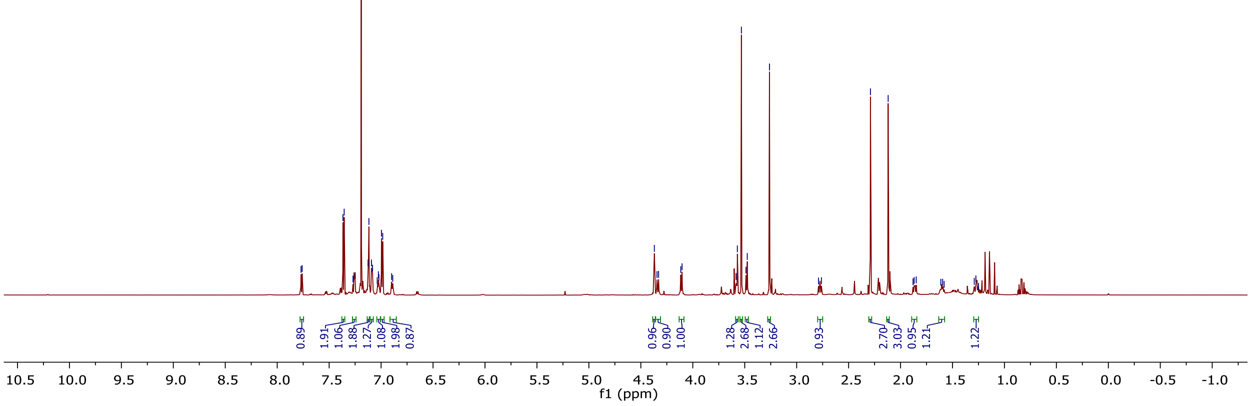

^{13}C NMR

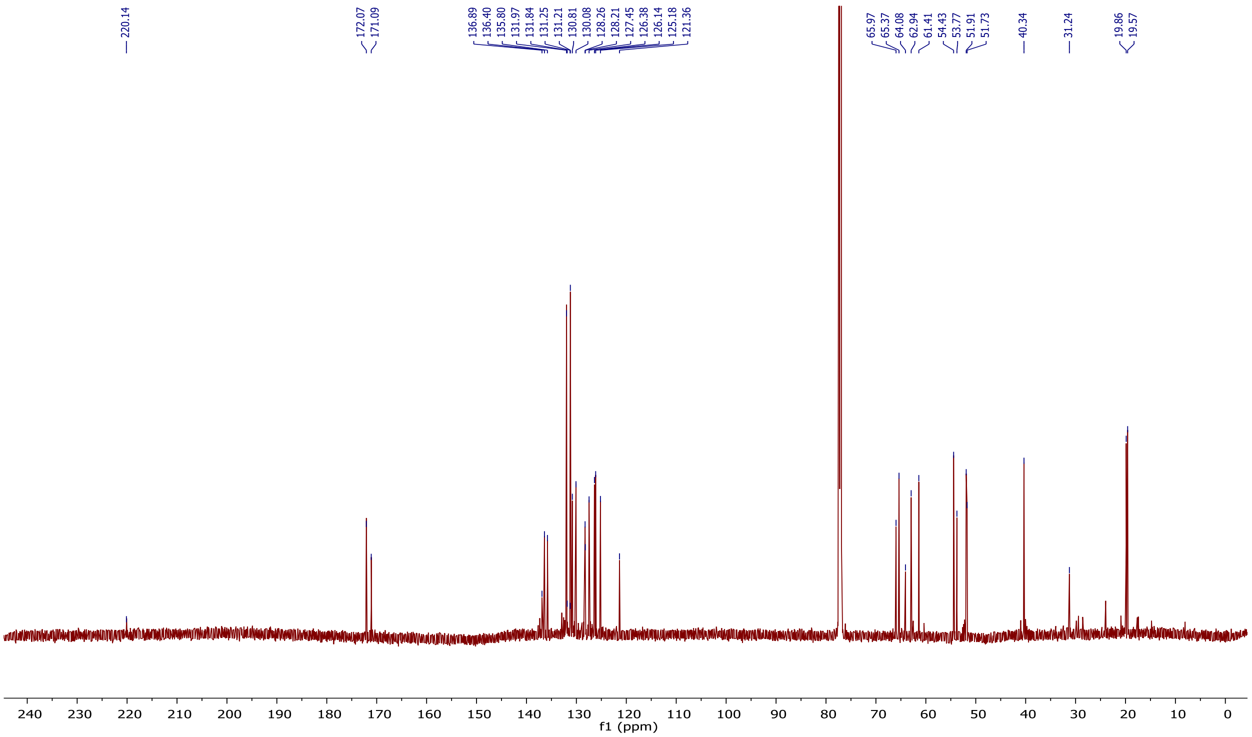

HPLC traces for **6f**, racemic top enantiomer 1 bottom

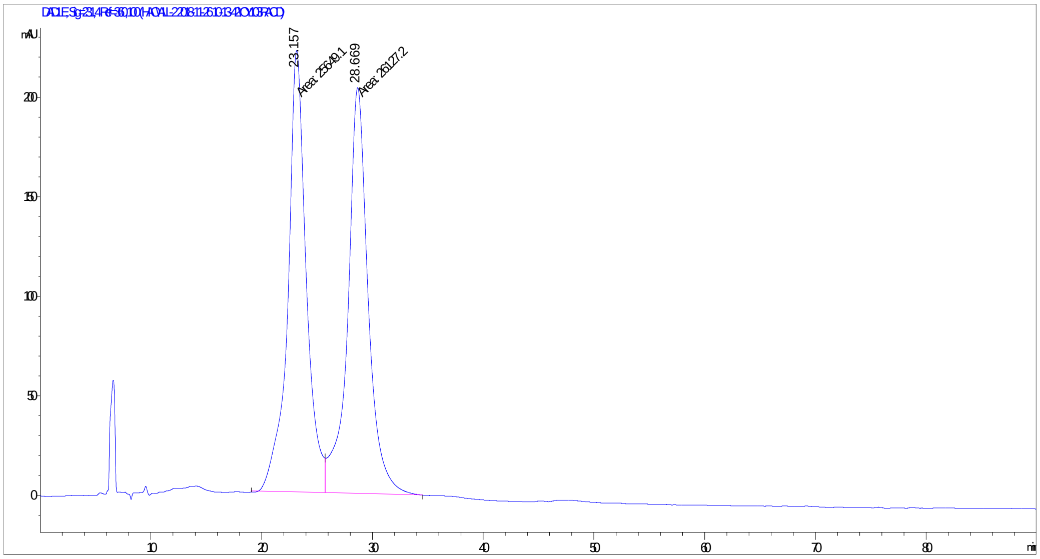

#	Time	Area	Height	Width	Area%	Symmetry
1	23.157	25649.1	221.5	1.9295	49.538	0.901
2	28.669	26127.2	203.7	2.1376	50.462	0.92

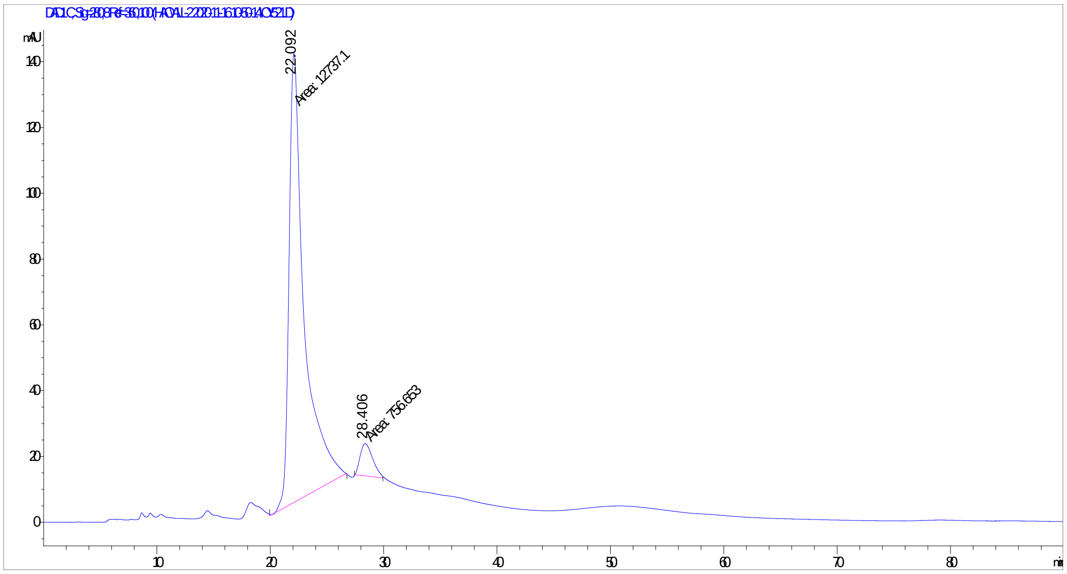

#	Time	Area	Height	Width	Area%	Symmetry
1	22.092	12737.1	136.5	1.5548	94.393	0.458
2	28.406	756.7	9.8	1.2842	5.607	0.64

^{1}H NMR

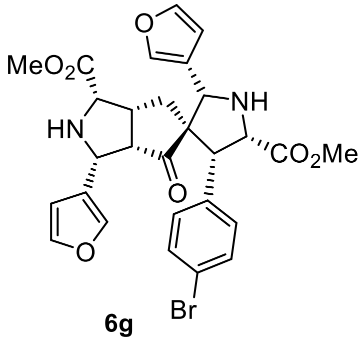

6g

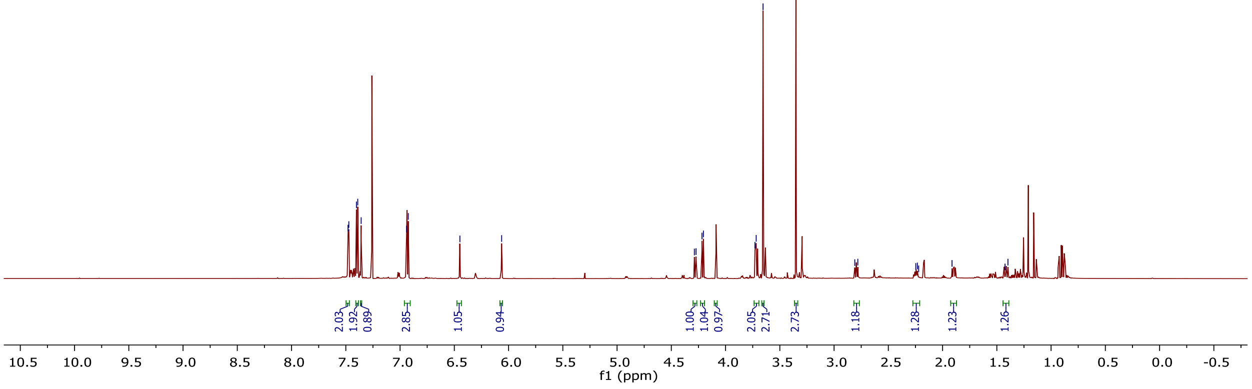

^{13}C NMR

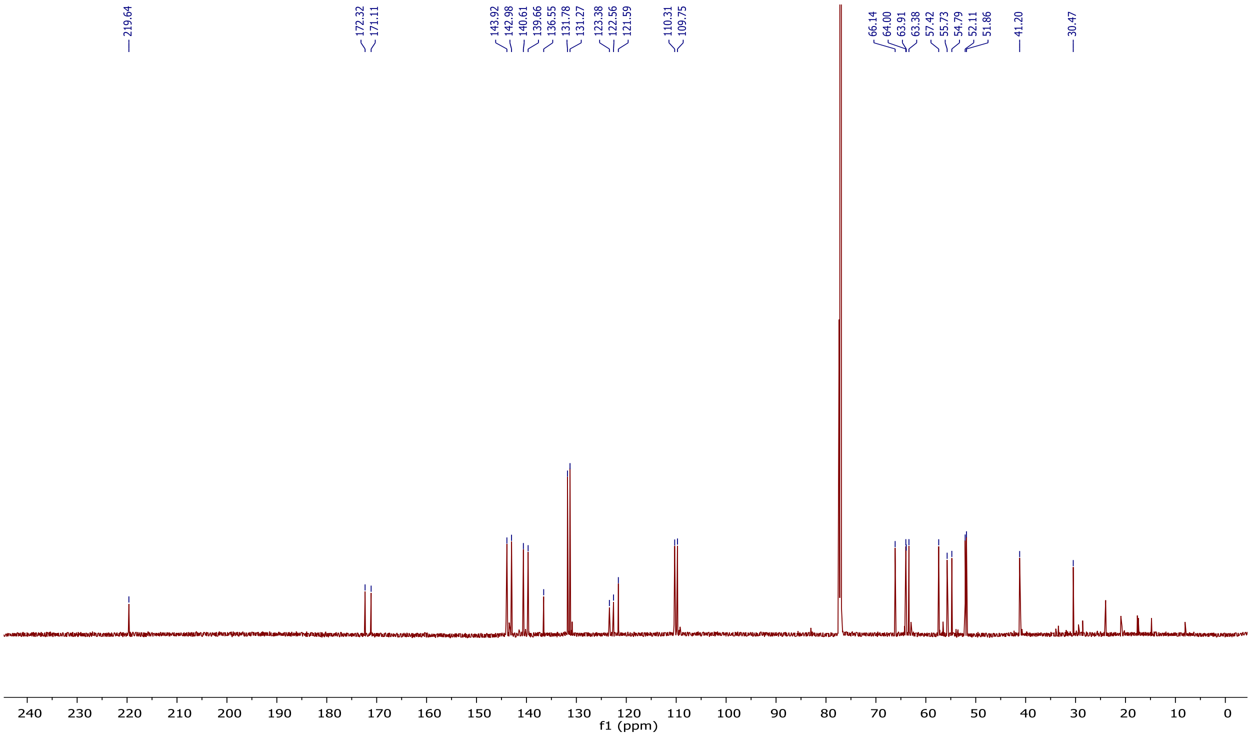

HPLC traces for **6g**, racemic top enantiomer 1 bottom

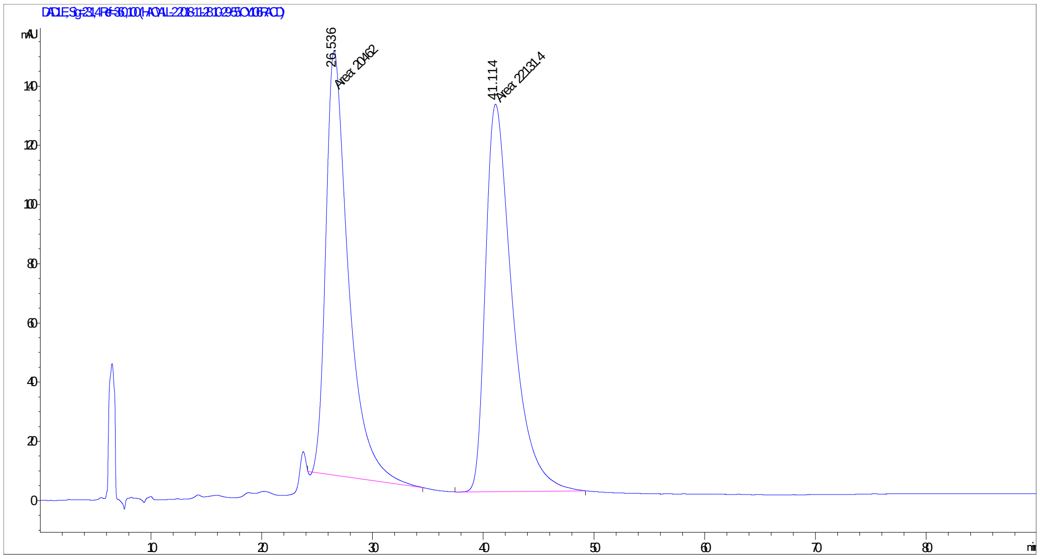

#	Time	Area	Height	Width	Area%	Symmetry
1	26.536	20462	143.5	2.3768	48.040	0.549
2	41.114	22131.4	130.9	2.817	51.960	0.568

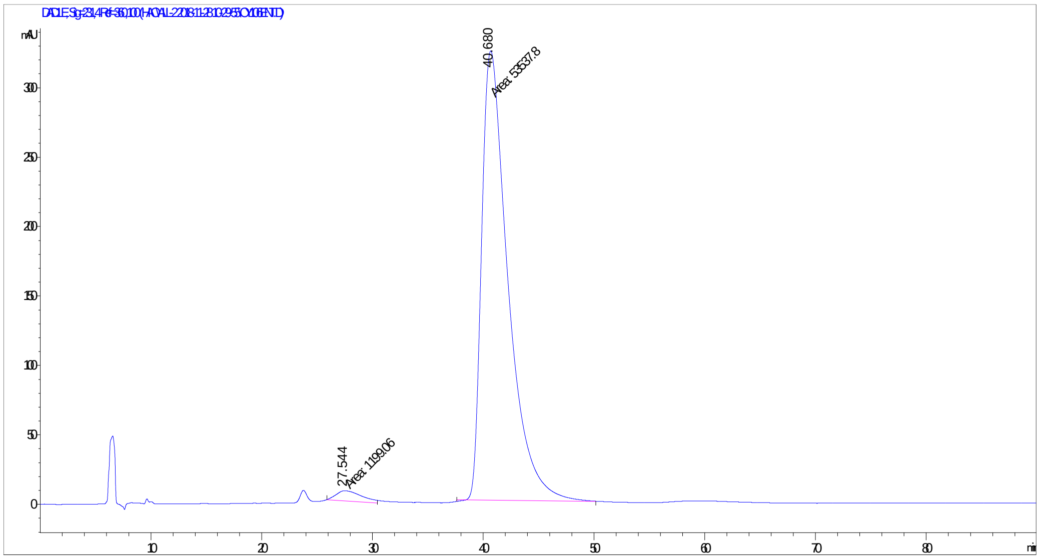

#	Time	Area	Height	Width	Area%	Symmetry
1	27.544	1199.1	7.4	2.7173	2.191	0.476
2	40.68	53537.8	323.6	2.7574	97.809	0.5
